# Supplementary figures and images for: Development of 50 InDel-based barcode system for genetic identification of tartary buckwheat resources
Source: PLoS One. 2021 Jun 3;16(6):e0250786. doi: 10.1371/journal.pone.0250786 (PMC8174720; doi:10.1371/journal.pone.0250786)

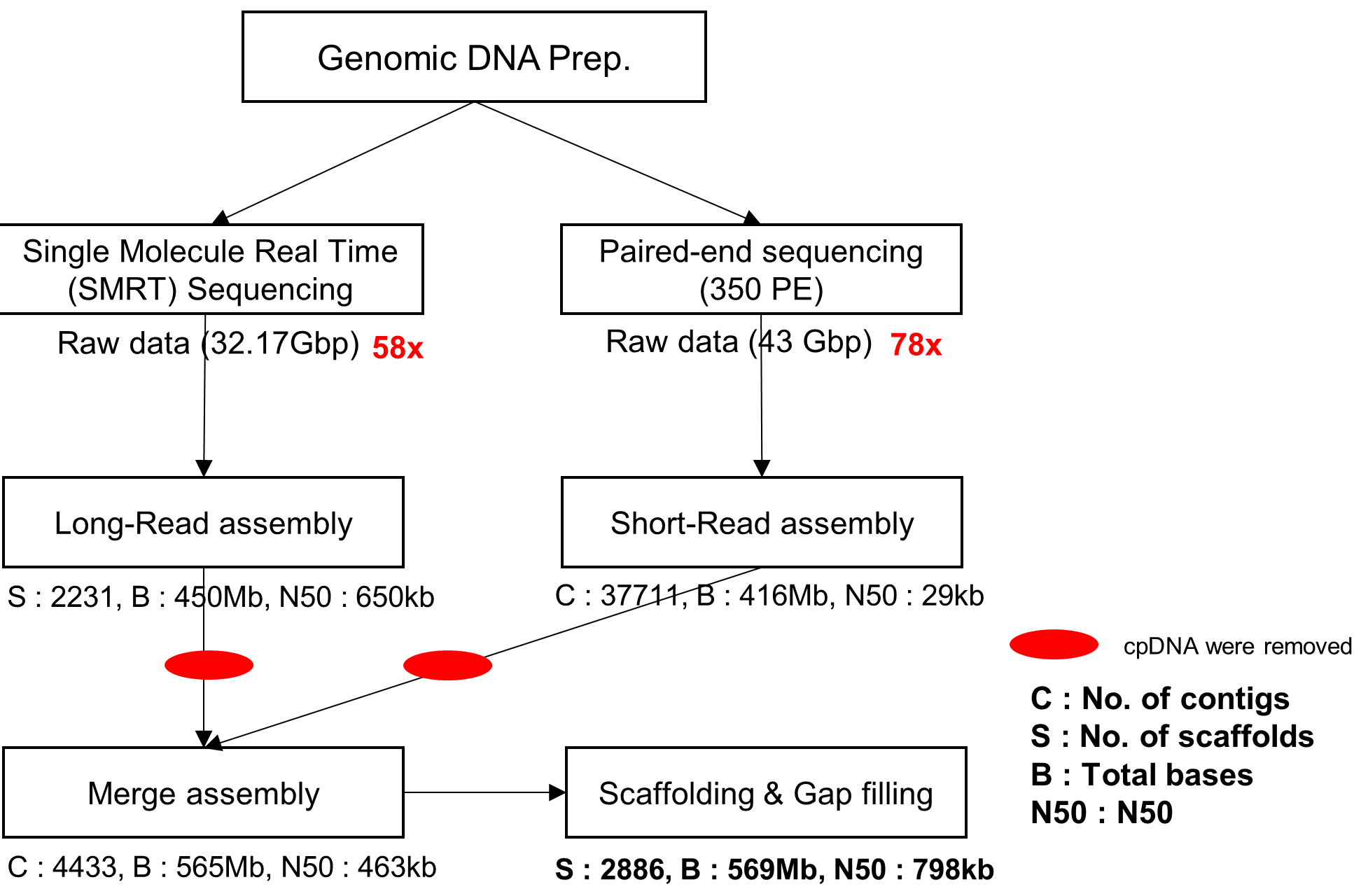

Supplement: S1 Fig — (TIF) [file pone.0250786.s001.tif]

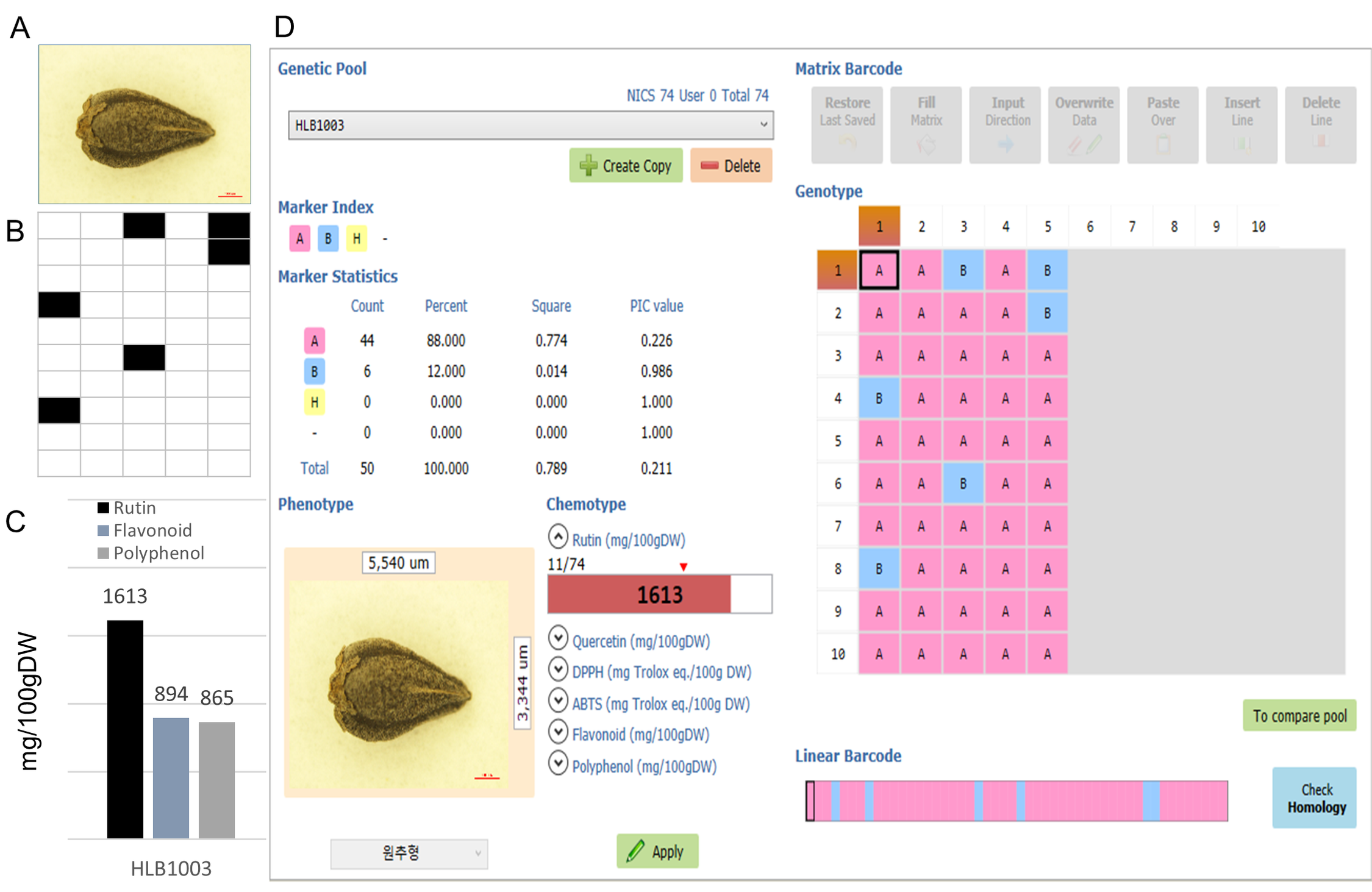

Supplement: S3 Fig — (TIF) [file pone.0250786.s003.tif]
