# Supplementary material for: Development of 50 InDel-based barcode system for genetic identification of tartary buckwheat resources
Source: PLoS One. 2021 Jun 3;16(6):e0250786. doi: 10.1371/journal.pone.0250786 (PMC8174720; doi:10.1371/journal.pone.0250786)
Supplement: S2 Fig — (PDF) [file pone.0250786.s002.pdf]

Supplementary materials and methods

Raw images of agarose gels

For preparing 3% agarose gel, 3 g of agarose were completely dissolved in 100 mL 0.5x TBE using a microwave. After cooling down to about 60 °C, it was poured into a gel tray. Once solidified at room temperature, the agarose gel was placed into the gel box with 0.5x TBE. DNA samples were prepared by mixing 1 volume LoadingSTAR (Dynebio, Republic of Korea) with 5 volume PCR product. The samples were loaded, and then the gel was run at 80–150 V until the dye line was approximately 75-80% of the way down the gel. After the run, the gel placed on UV transilluminator (Azure^{TM} C150, CA, USA) to immediately visualize bands under a detection wavelength of 302 nm. Using an image software, Gimp 2.10.22, original tiff images were annotated and then saved as tiff files with LZW compression. We built a single PDF file compiled from the multiple tiff image files (S2 Fig). The bands were confirmed via 100 bp DNA ladder (Dynebio, Republic of Korea) as a guide and the e-PCR products in 26 tartary buckwheat genomes (S5 Table). The numbers of the genotypes are listed in S1 Table. M, 100 bp DNA ladder; Ref, *Fagopyrum tataricum* cv. "Daegwan 3-7."

S2 Fig. Agarose gels showing the separation of alleles of 50 InDels in 73 tartary buckwheat accessions.

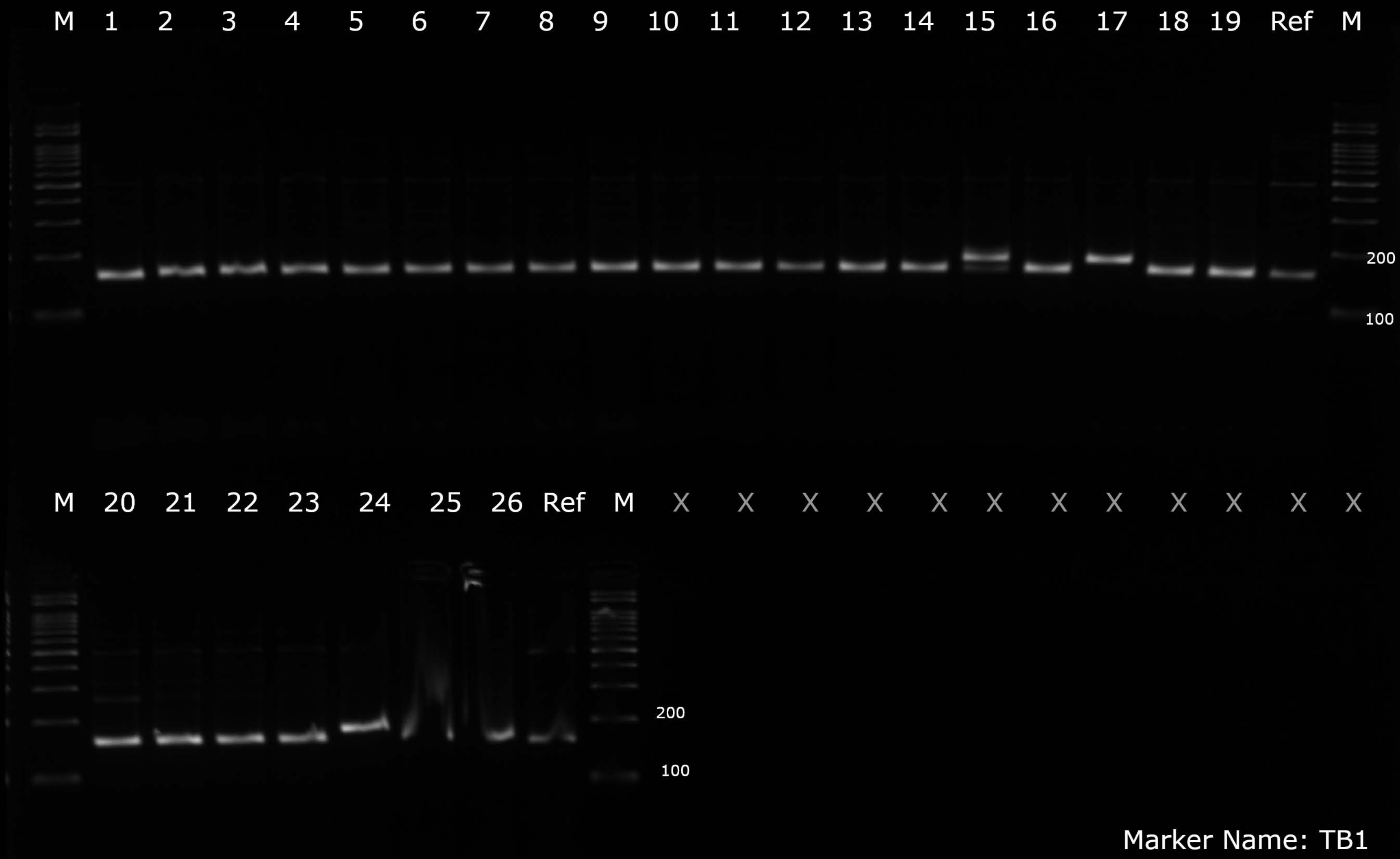
M 1 2 3 4 5 6 7 8 9 10 11 12 13 14 15 16 17 18 19 Ref M
200
100
M 20 21 22 23 24 25 26 Ref M X X X X X X X X X X X X
200
100
Marker Name: TB1

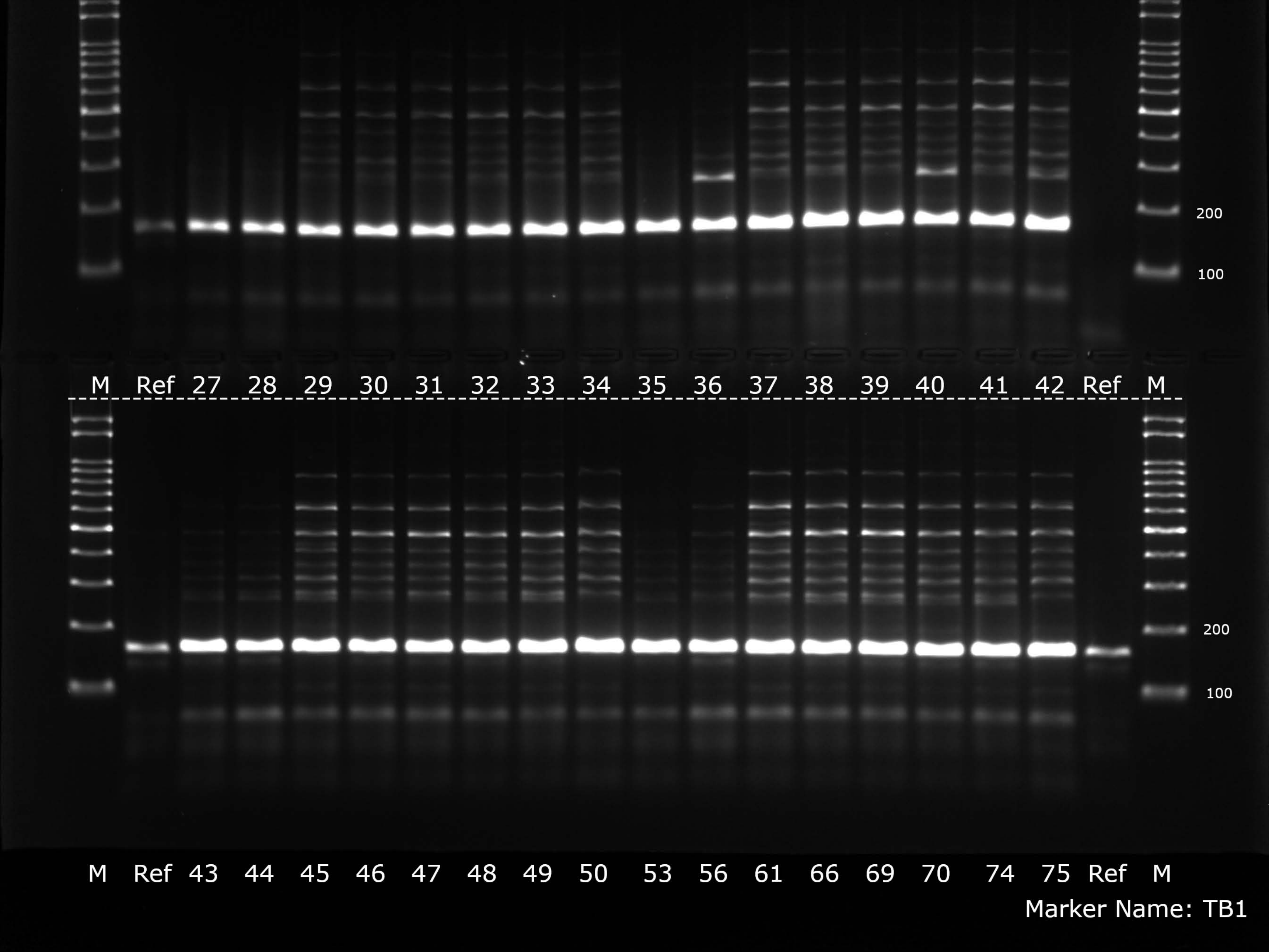
200
100
M Ref 27 28 29 30 31 32 33 34 35 36 37 38 39 40 41 42 Ref M
200
100
M Ref 43 44 45 46 47 48 49 50 53 56 61 66 69 70 74 75 Ref M
Marker Name: TB1

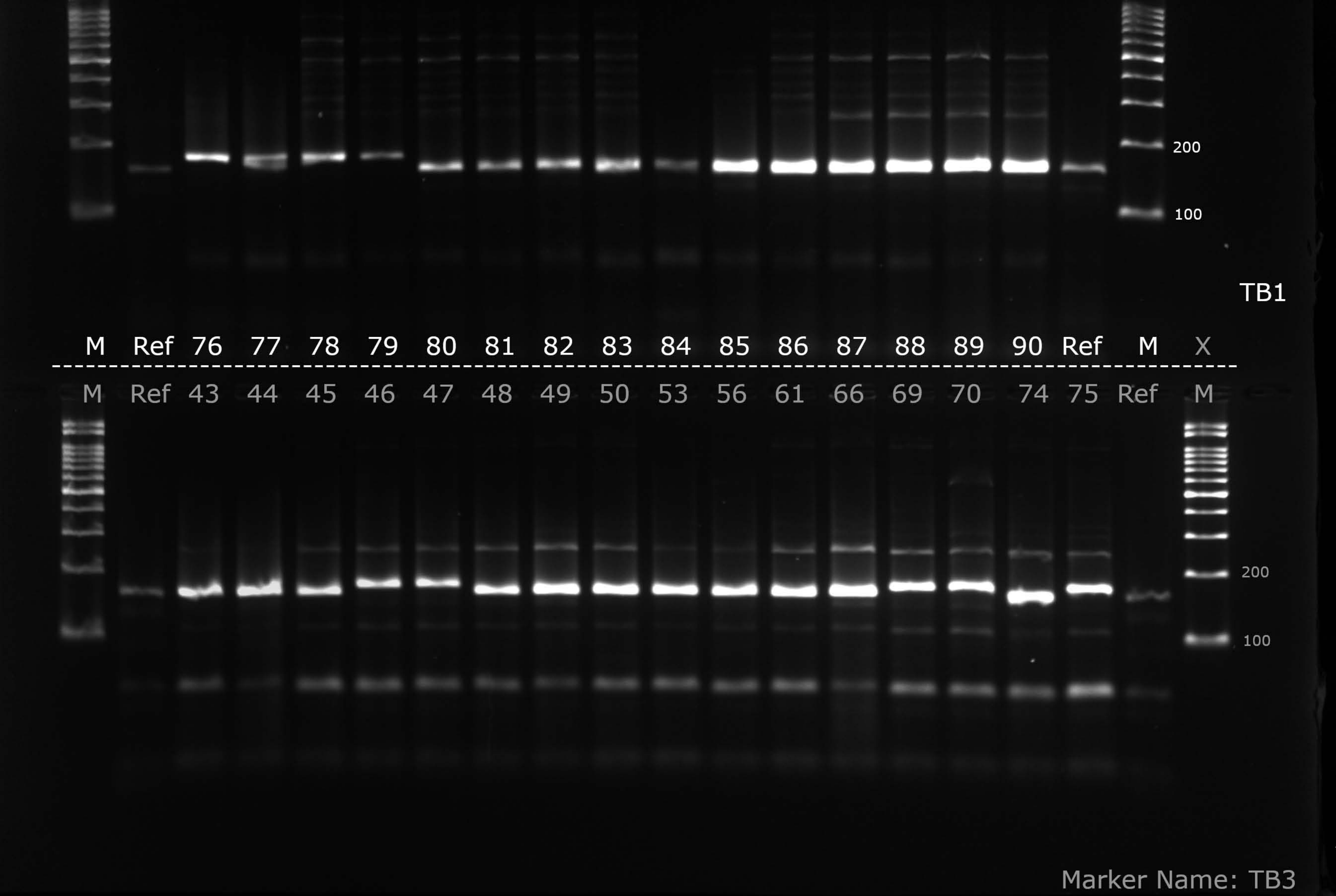
200
100
TB1
M Ref 76 77 78 79 80 81 82 83 84 85 86 87 88 89 90 Ref M X
M Ref 43 44 45 46 47 48 49 50 53 56 61 66 69 70 74 75 Ref M
200
100
Marker Name: TB3

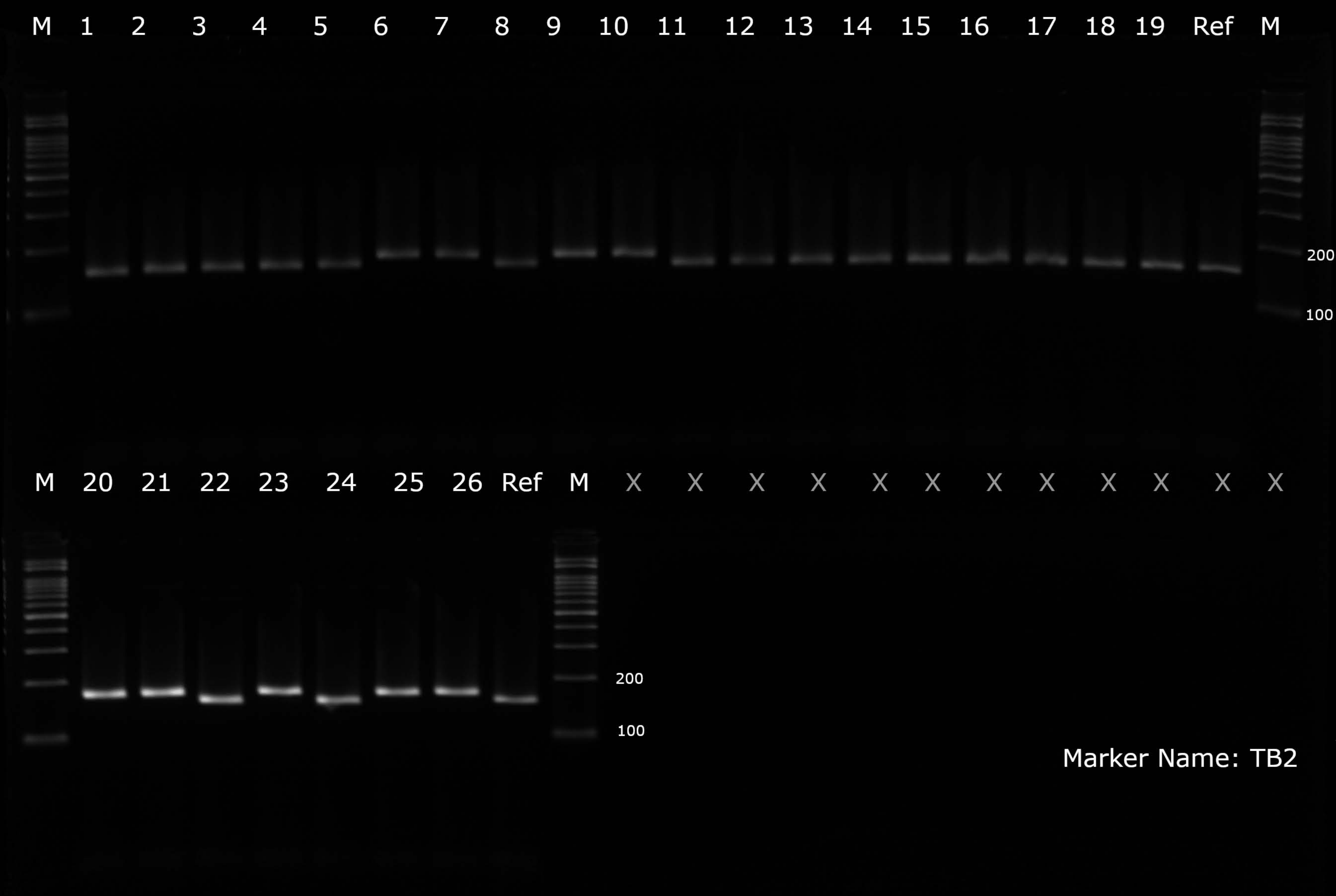
M 1 2 3 4 5 6 7 8 9 10 11 12 13 14 15 16 17 18 19 Ref M
200
100
M 20 21 22 23 24 25 26 Ref M X X X X X X X X X X X X
200
100
Marker Name: TB2

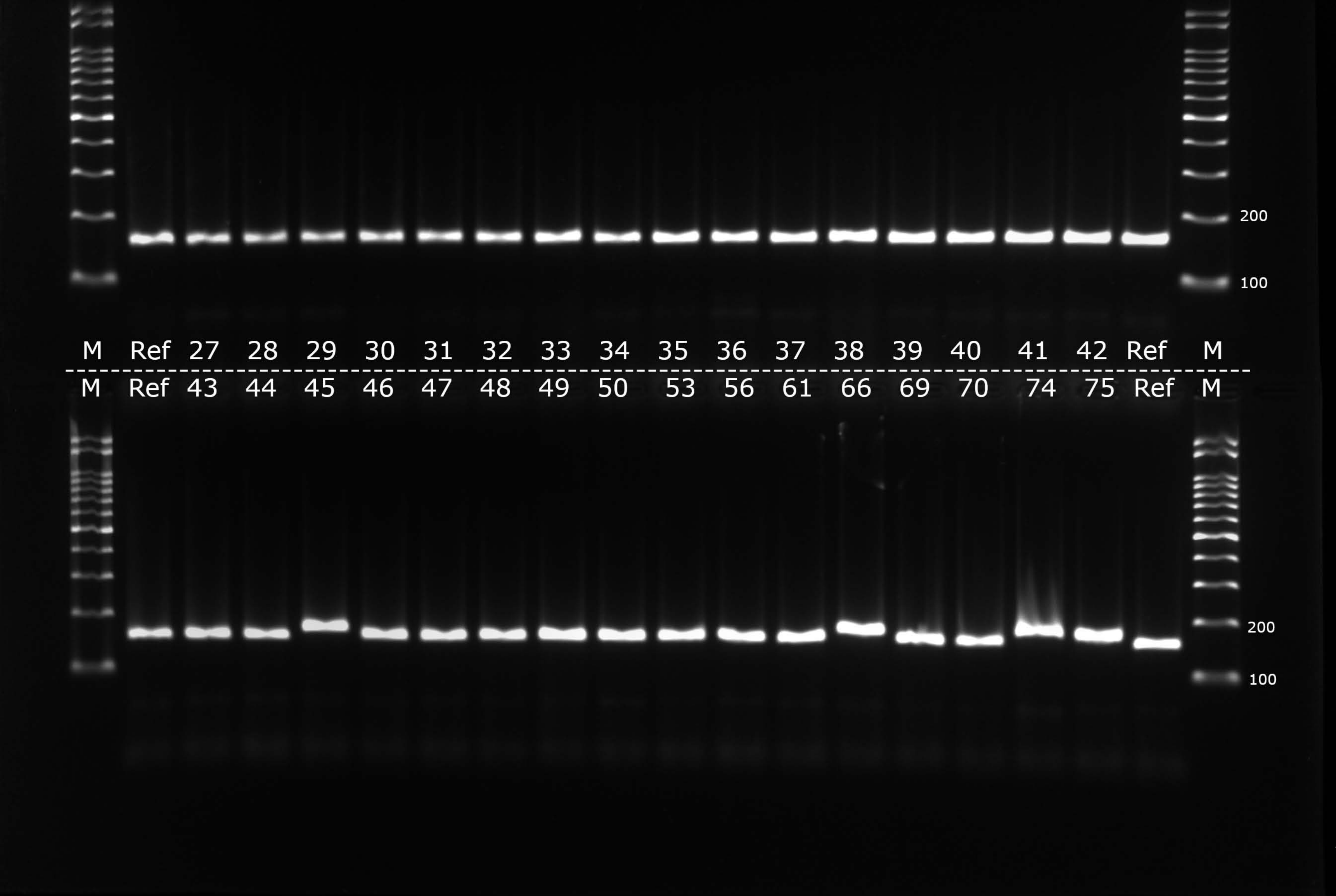

Marker Name: TB2

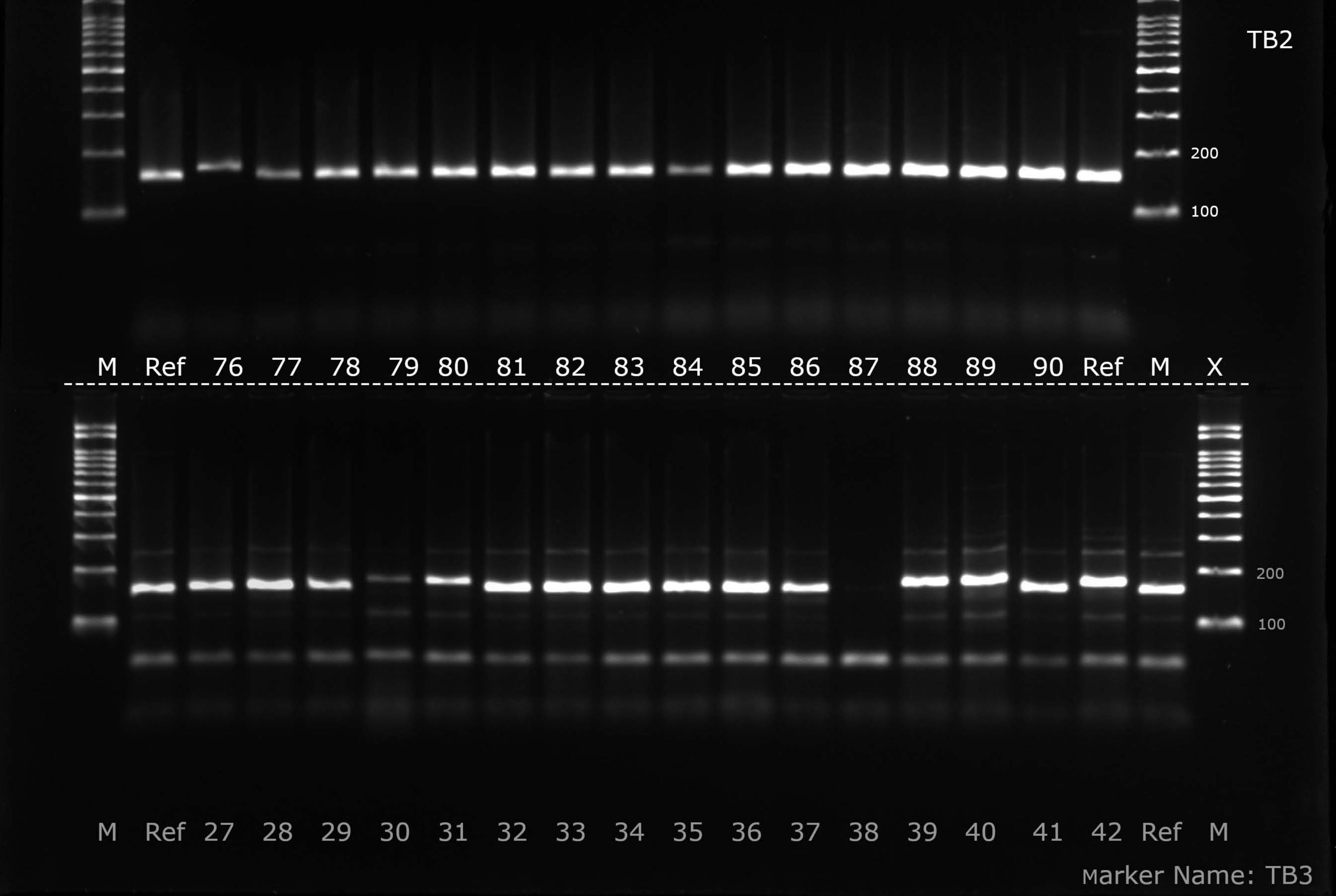
TB2
200
100
M Ref 76 77 78 79 80 81 82 83 84 85 86 87 88 89 90 Ref M X
200
100
M Ref 27 28 29 30 31 32 33 34 35 36 37 38 39 40 41 42 Ref M
Marker Name: TB3

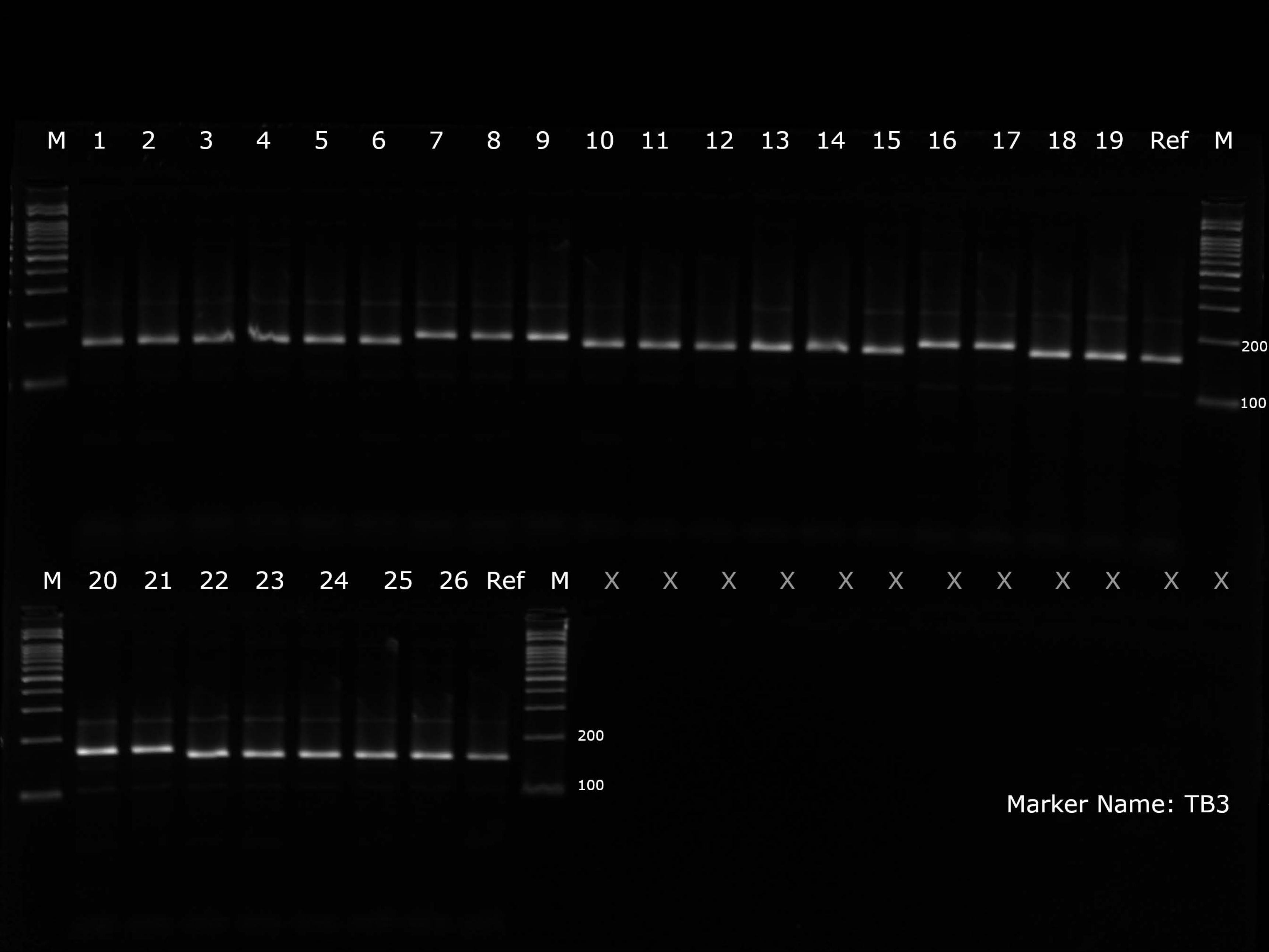
M 1 2 3 4 5 6 7 8 9 10 11 12 13 14 15 16 17 18 19 Ref M
200
100
M 20 21 22 23 24 25 26 Ref M X X X X X X X X X X X X
200
100
Marker Name: TB3

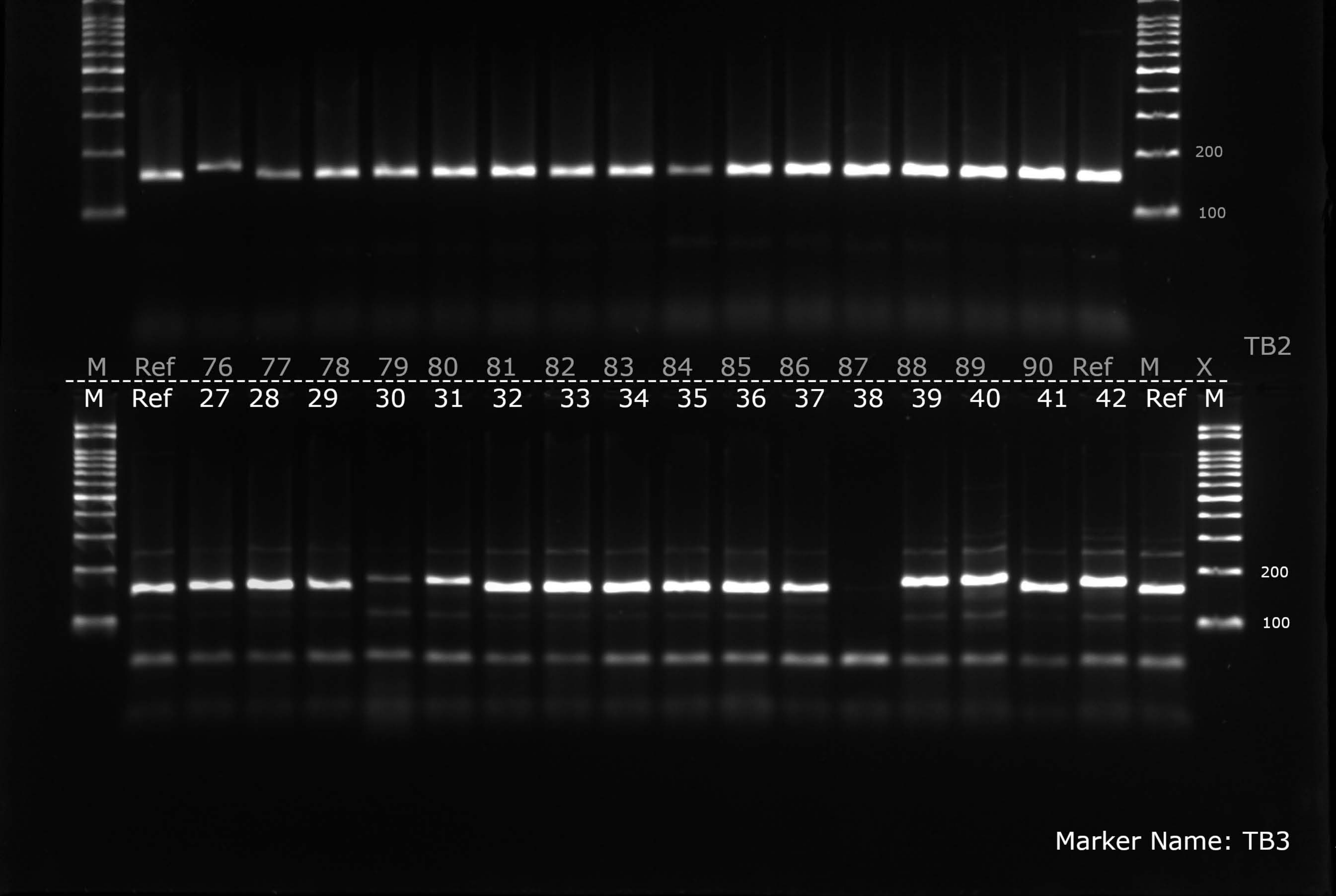
200
100
TB2
M Ref 76 77 78 79 80 81 82 83 84 85 86 87 88 89 90 Ref M X
M Ref 27 28 29 30 31 32 33 34 35 36 37 38 39 40 41 42 Ref M
200
100
Marker Name: TB3

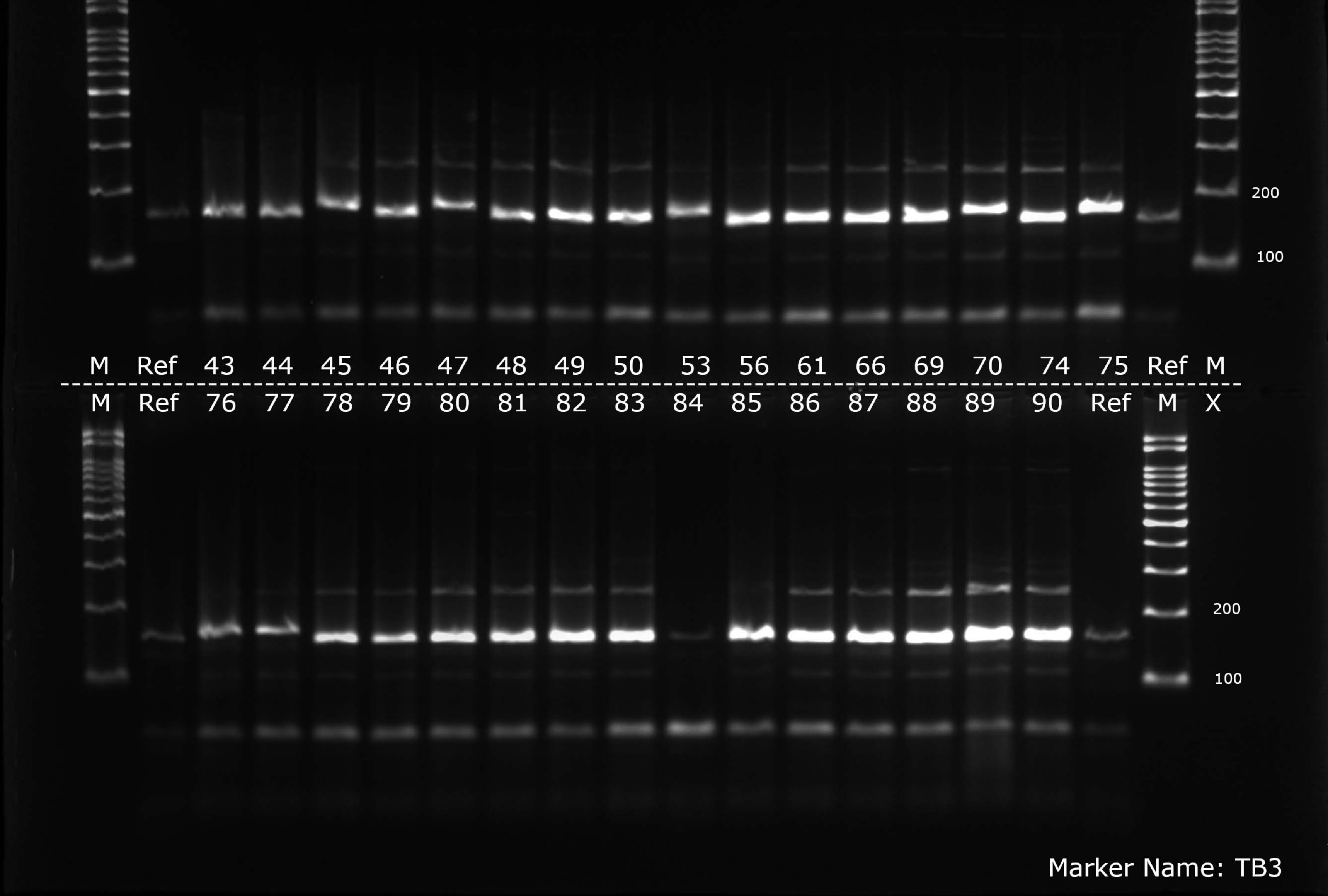
M Ref 43 44 45 46 47 48 49 50 53 56 61 66 69 70 74 75 Ref M
200
100
M Ref 76 77 78 79 80 81 82 83 84 85 86 87 88 89 90 Ref M X
200
100
Marker Name: TB3

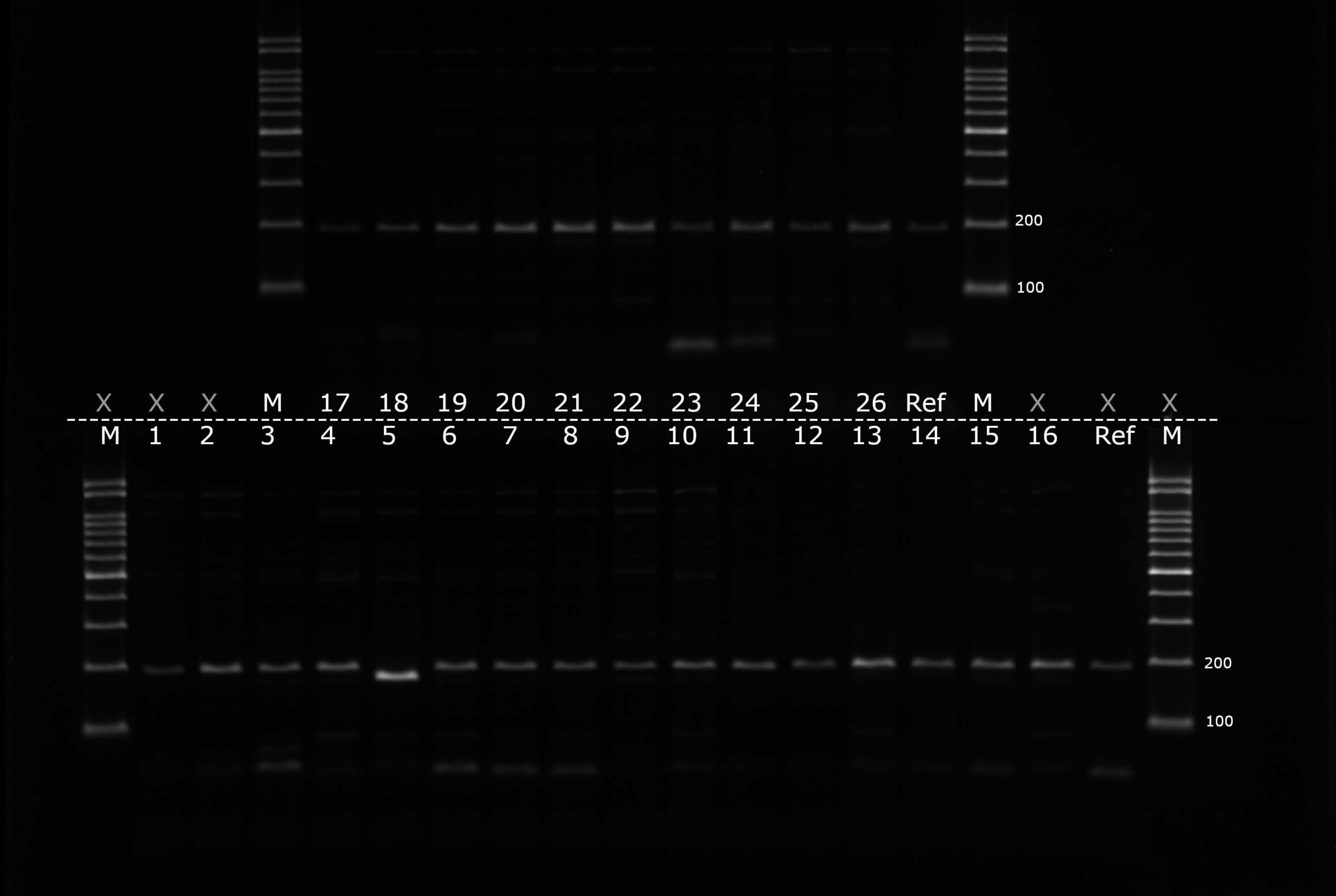

Marker Name: TB4

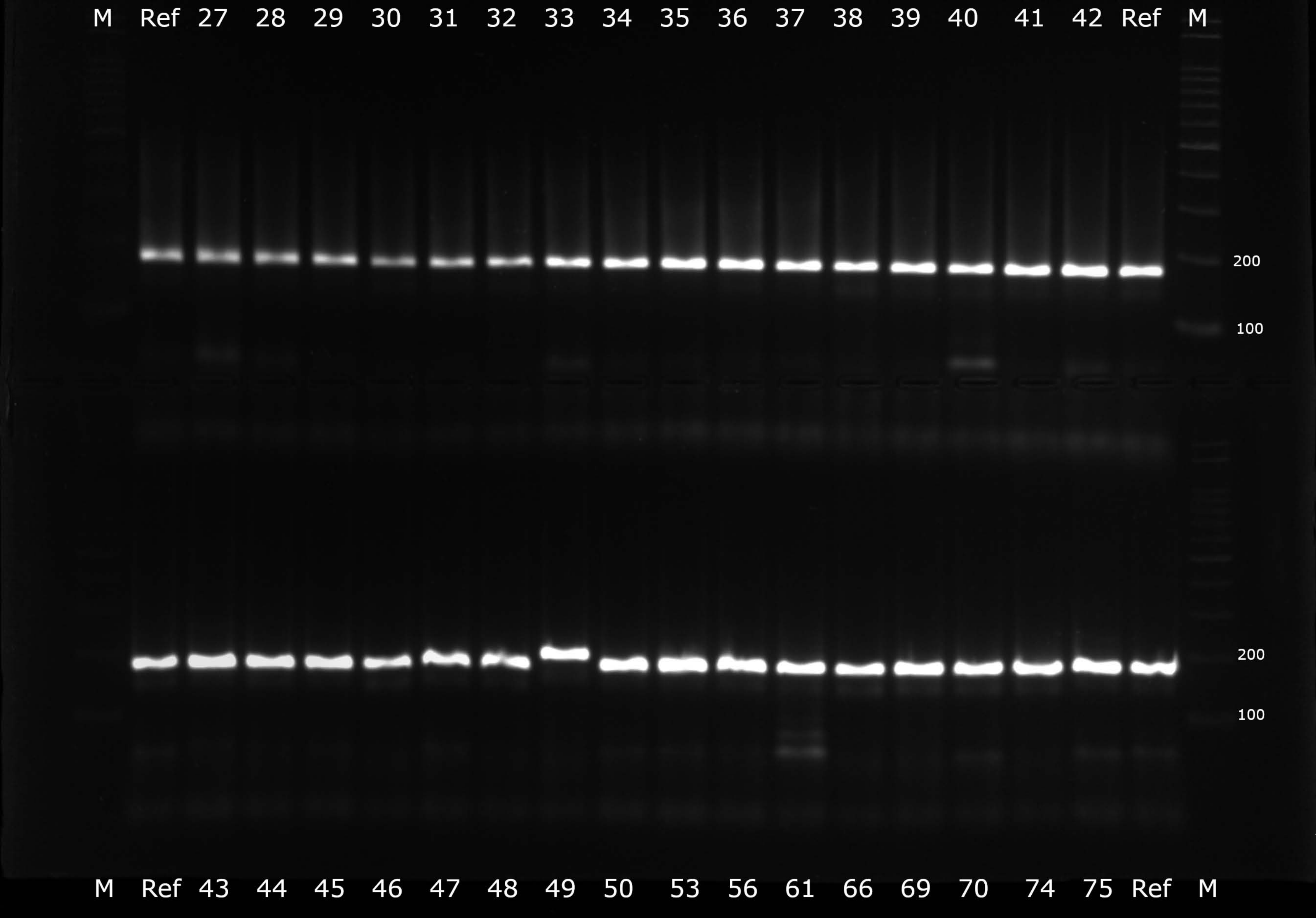

Marker Name: TB4

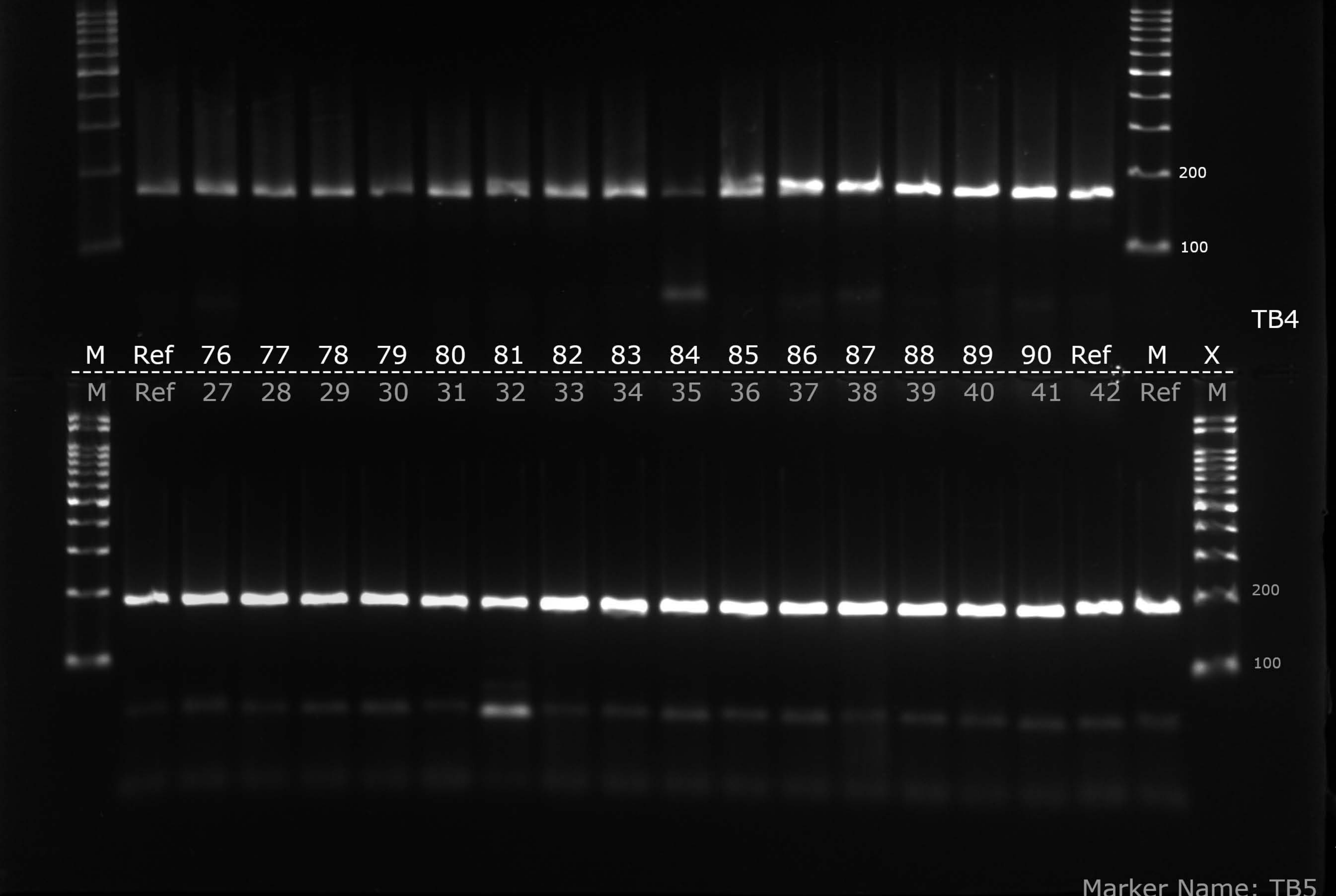
200
100
TB4
M Ref 76 77 78 79 80 81 82 83 84 85 86 87 88 89 90 Ref M X
M Ref 27 28 29 30 31 32 33 34 35 36 37 38 39 40 41 42 Ref M
200
100
Marker Name: TB5

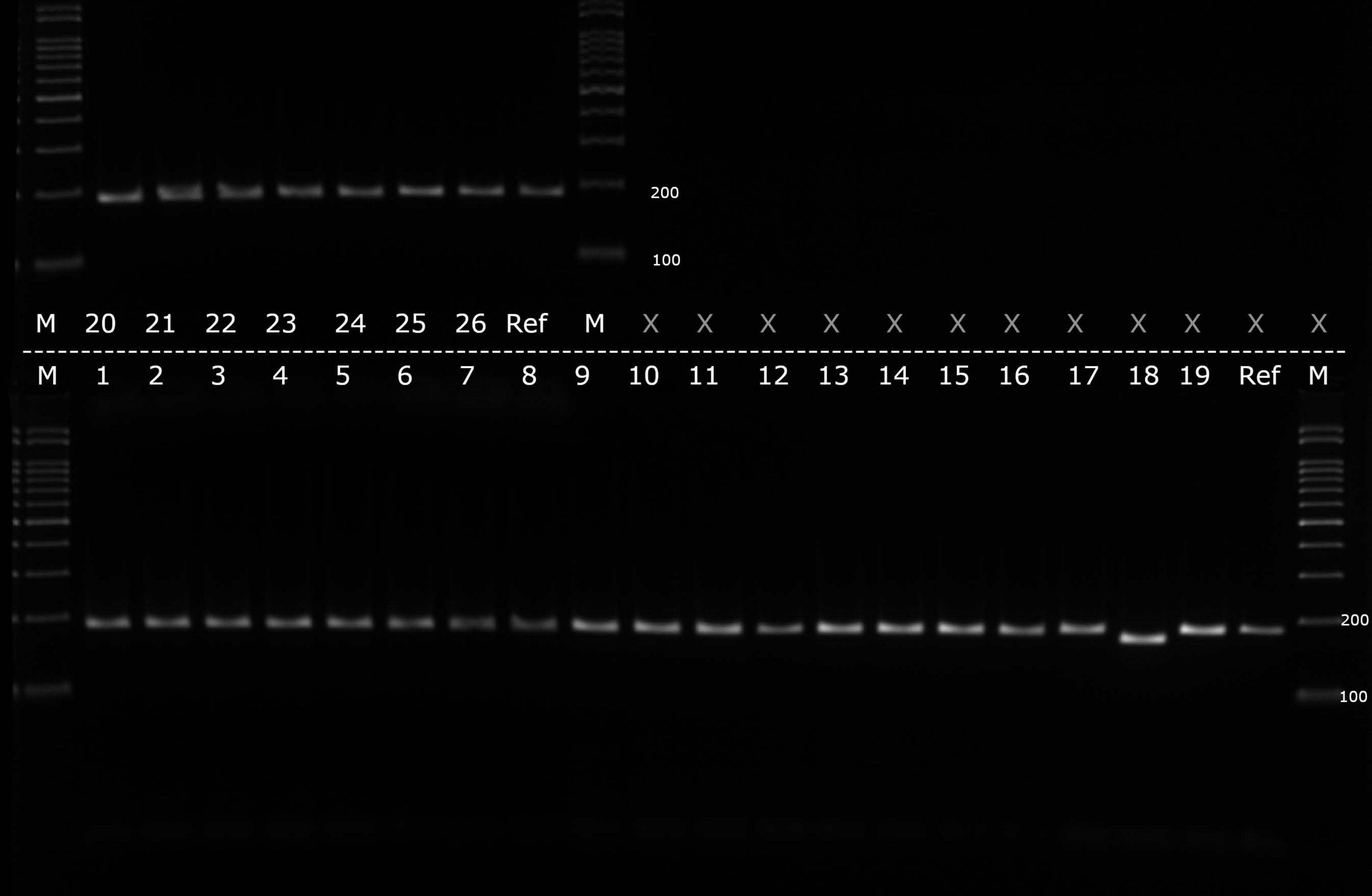

Marker Name: TB5

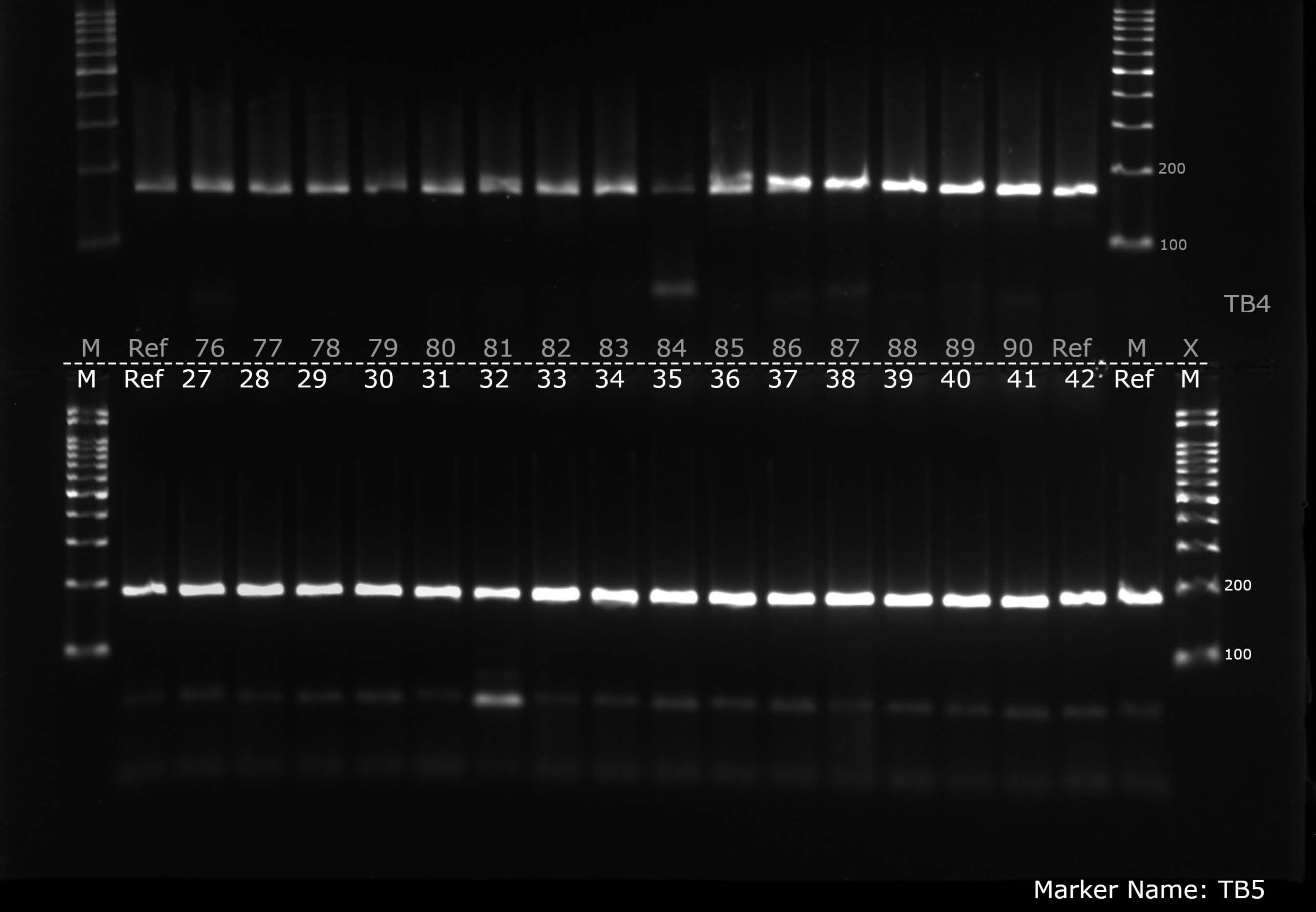
200
100
TB4
M Ref 76 77 78 79 80 81 82 83 84 85 86 87 88 89 90 Ref M X
M Ref 27 28 29 30 31 32 33 34 35 36 37 38 39 40 41 42 Ref M
200
100
Marker Name: TB5

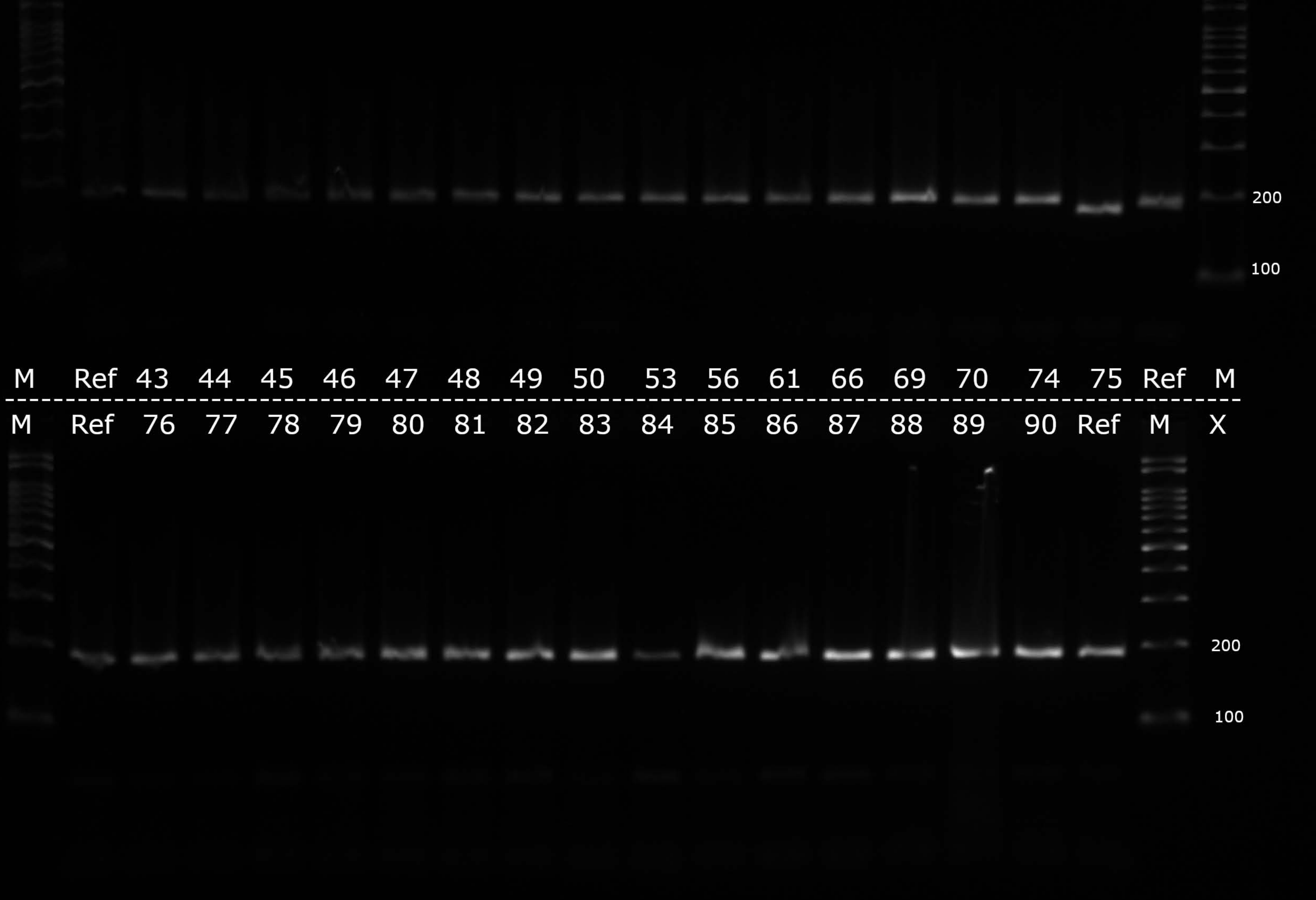

Marker Name: TB5

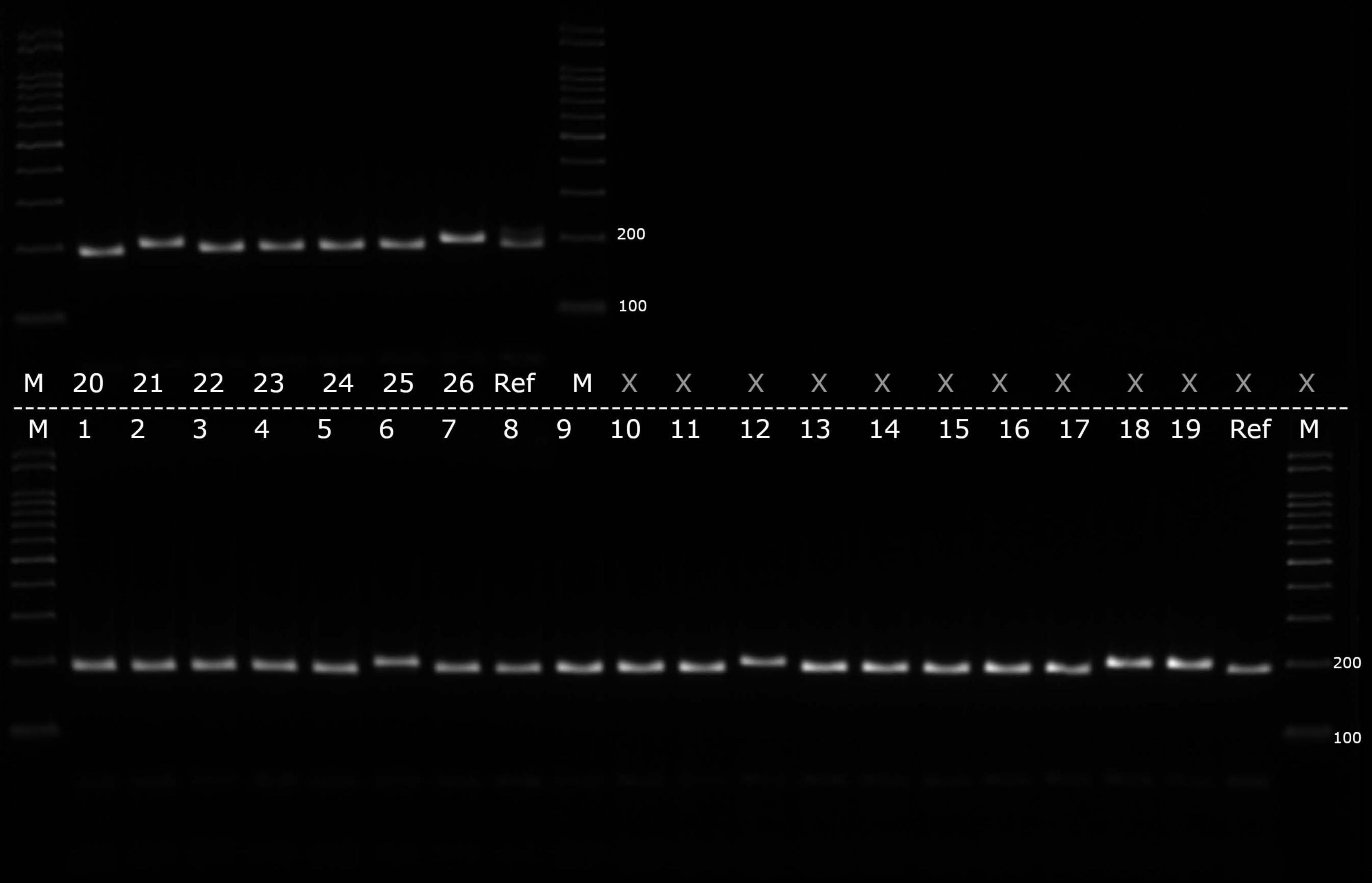

Marker Name: TB6

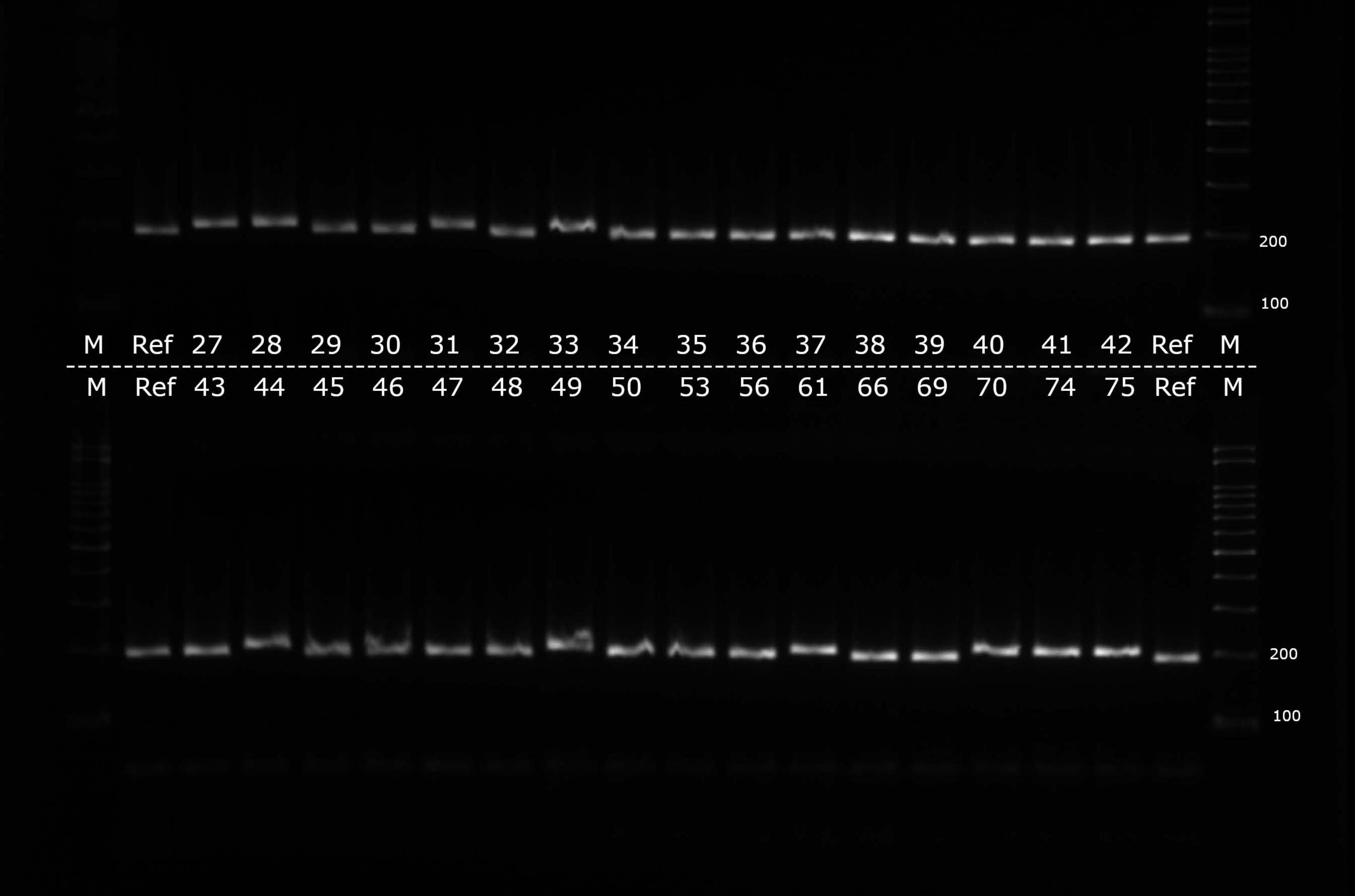

Marker Name: TB6

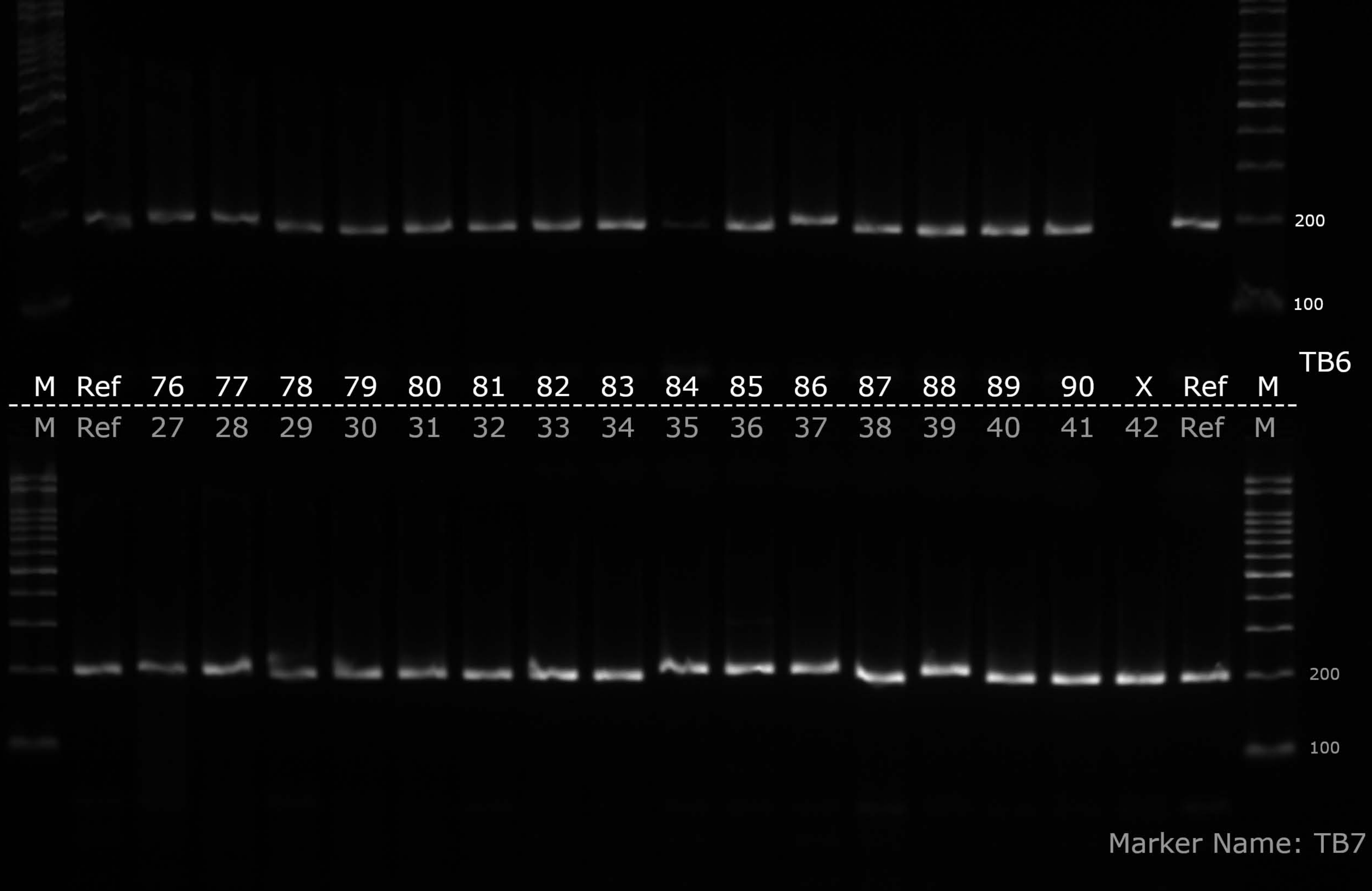
200
100
TB6
M Ref 76 77 78 79 80 81 82 83 84 85 86 87 88 89 90 X Ref M
M Ref 27 28 29 30 31 32 33 34 35 36 37 38 39 40 41 42 Ref M
200
100
Marker Name: TB7

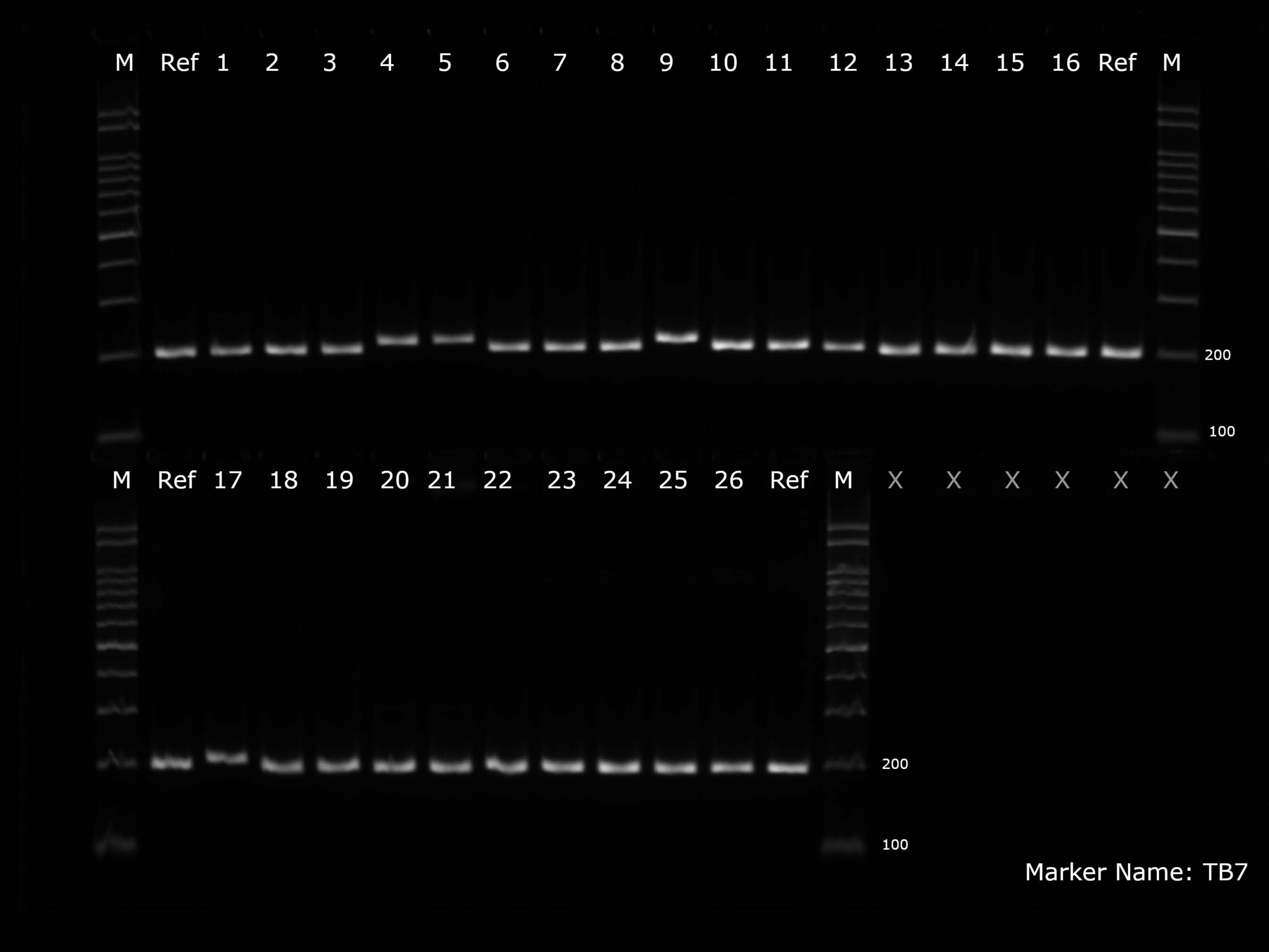
M Ref 1 2 3 4 5 6 7 8 9 10 11 12 13 14 15 16 Ref M
200
100
M Ref 17 18 19 20 21 22 23 24 25 26 Ref M X X X X X X
200
100
Marker Name: TB7

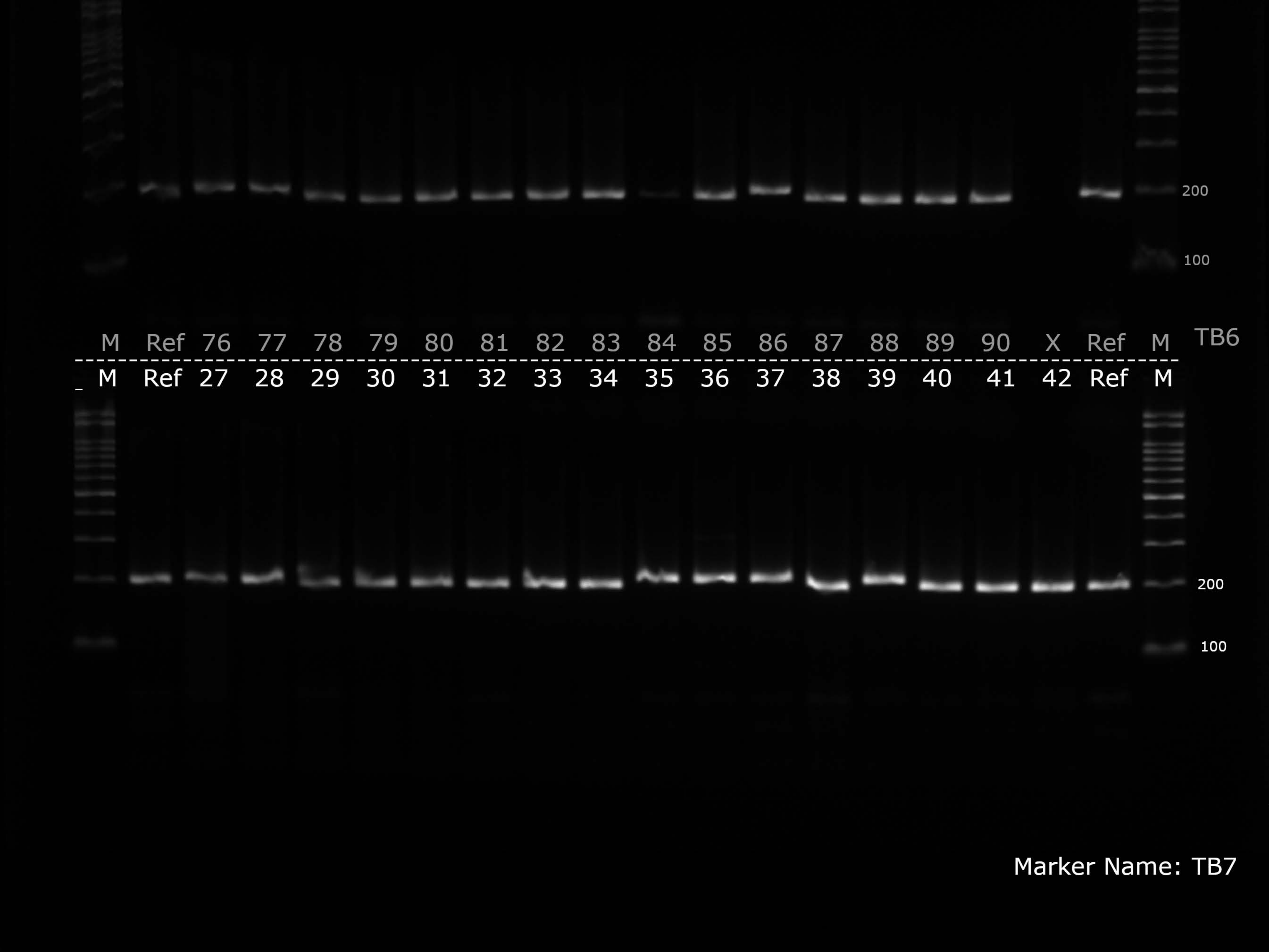
M Ref 76 77 78 79 80 81 82 83 84 85 86 87 88 89 90 X Ref M
TB6
200
100
M Ref 27 28 29 30 31 32 33 34 35 36 37 38 39 40 41 42 Ref M
200
100
Marker Name: TB7

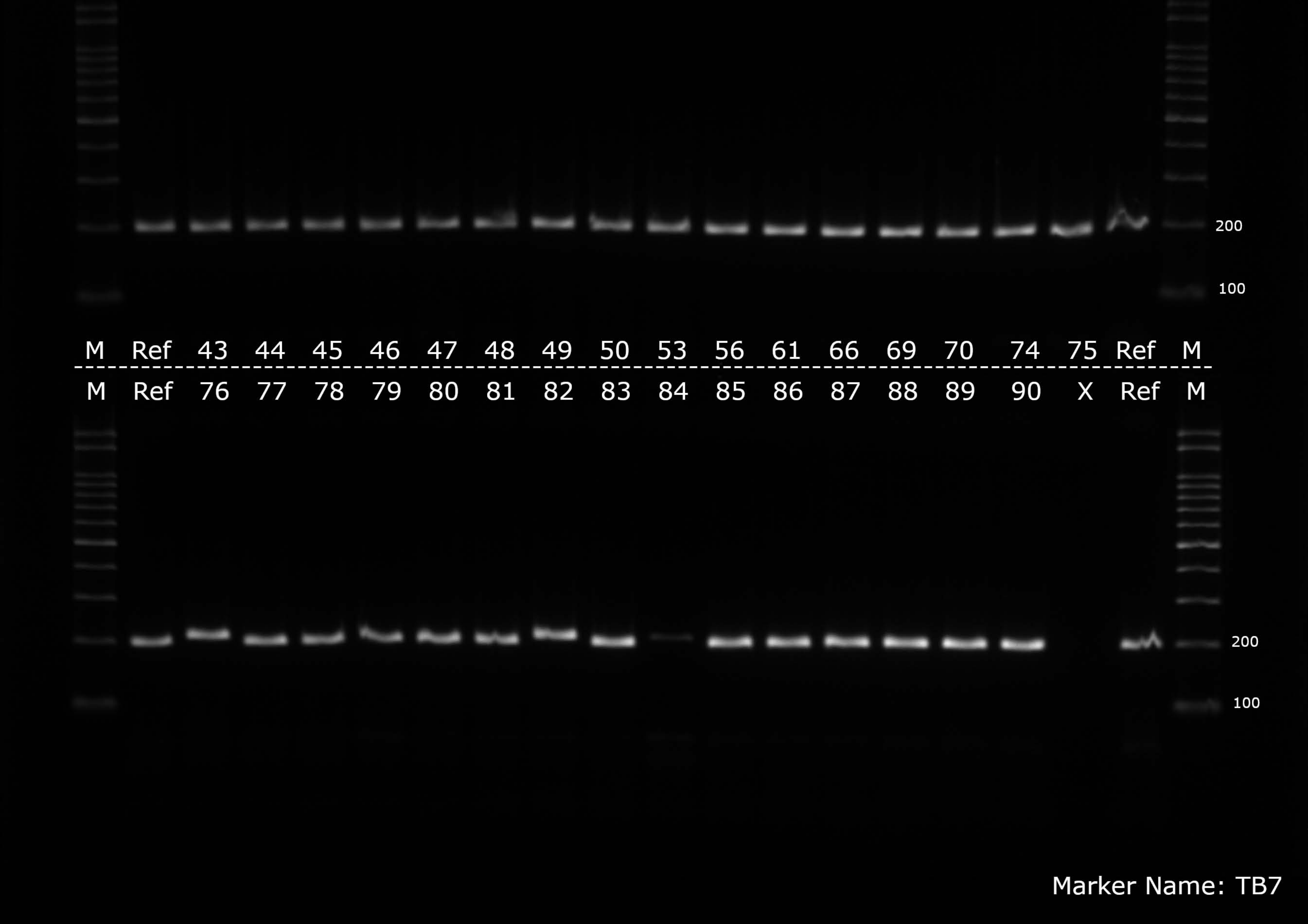
200
100
M Ref 43 44 45 46 47 48 49 50 53 56 61 66 69 70 74 75 Ref M
M Ref 76 77 78 79 80 81 82 83 84 85 86 87 88 89 90 X Ref M
200
100
Marker Name: TB7

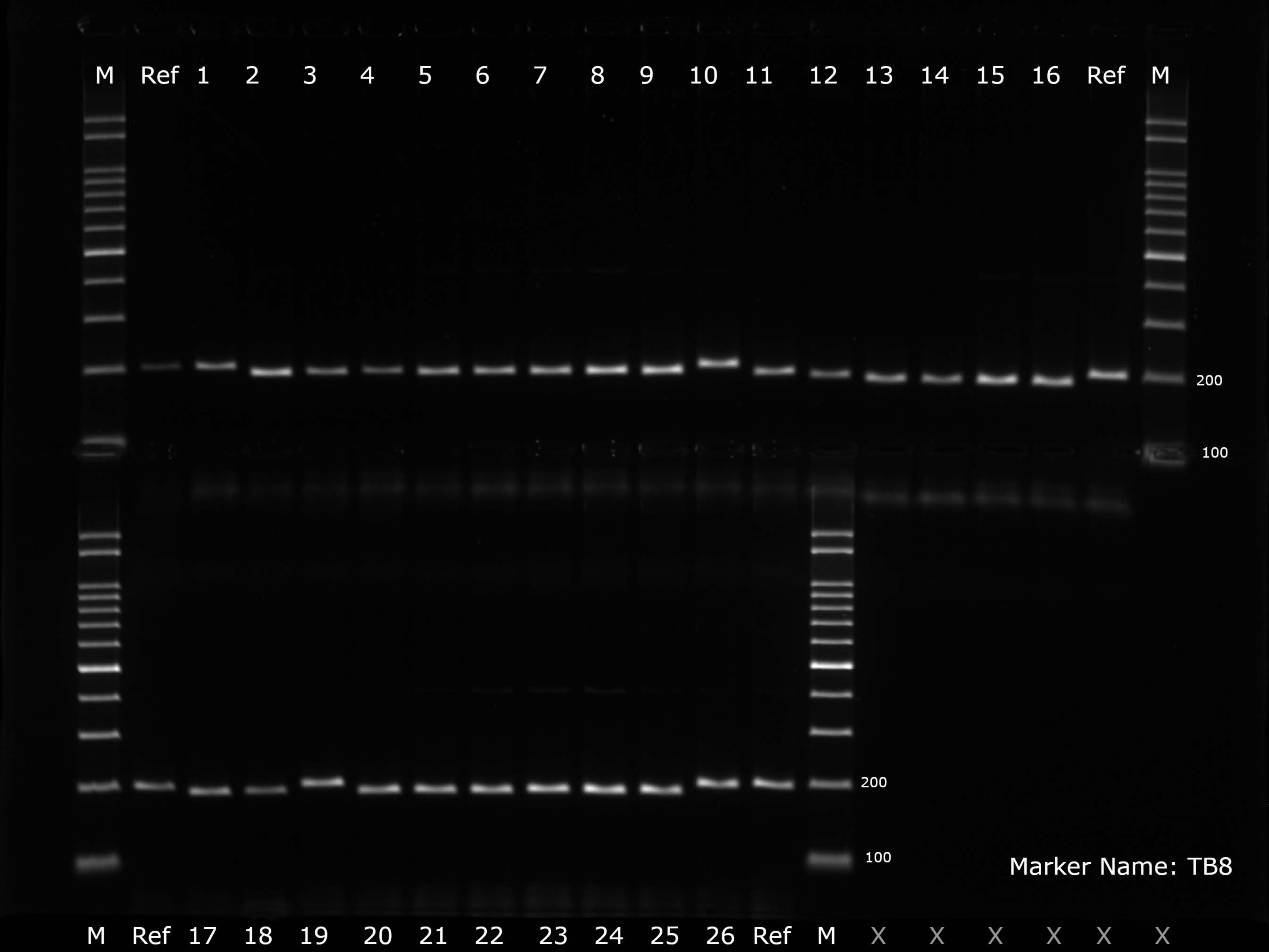
M Ref 1 2 3 4 5 6 7 8 9 10 11 12 13 14 15 16 Ref M
200
100
200
100
Marker Name: TB8
M Ref 17 18 19 20 21 22 23 24 25 26 Ref M X X X X X X

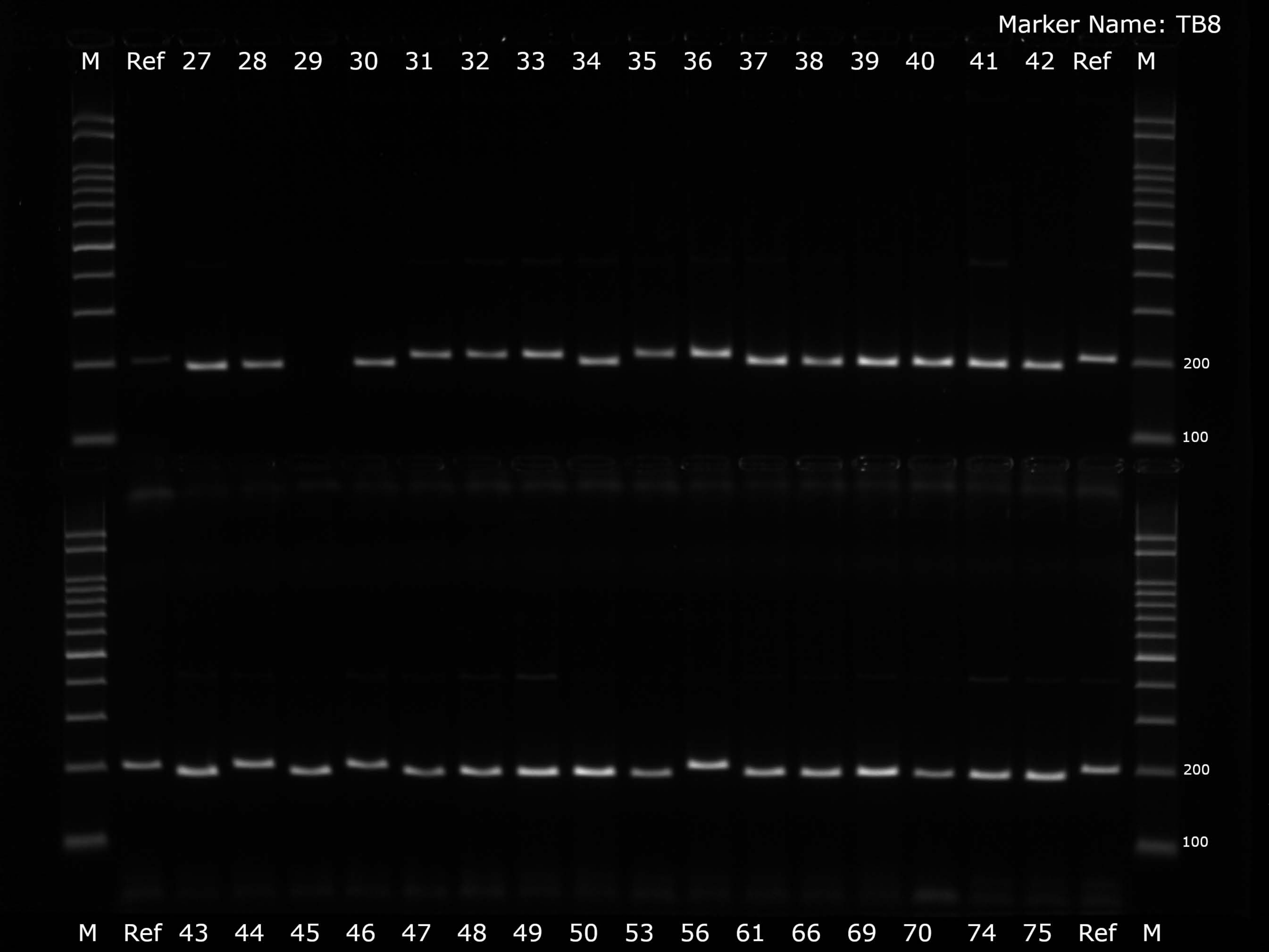
Marker Name: TB8
M Ref 27 28 29 30 31 32 33 34 35 36 37 38 39 40 41 42 Ref M
200
100
200
100
M Ref 43 44 45 46 47 48 49 50 53 56 61 66 69 70 74 75 Ref M

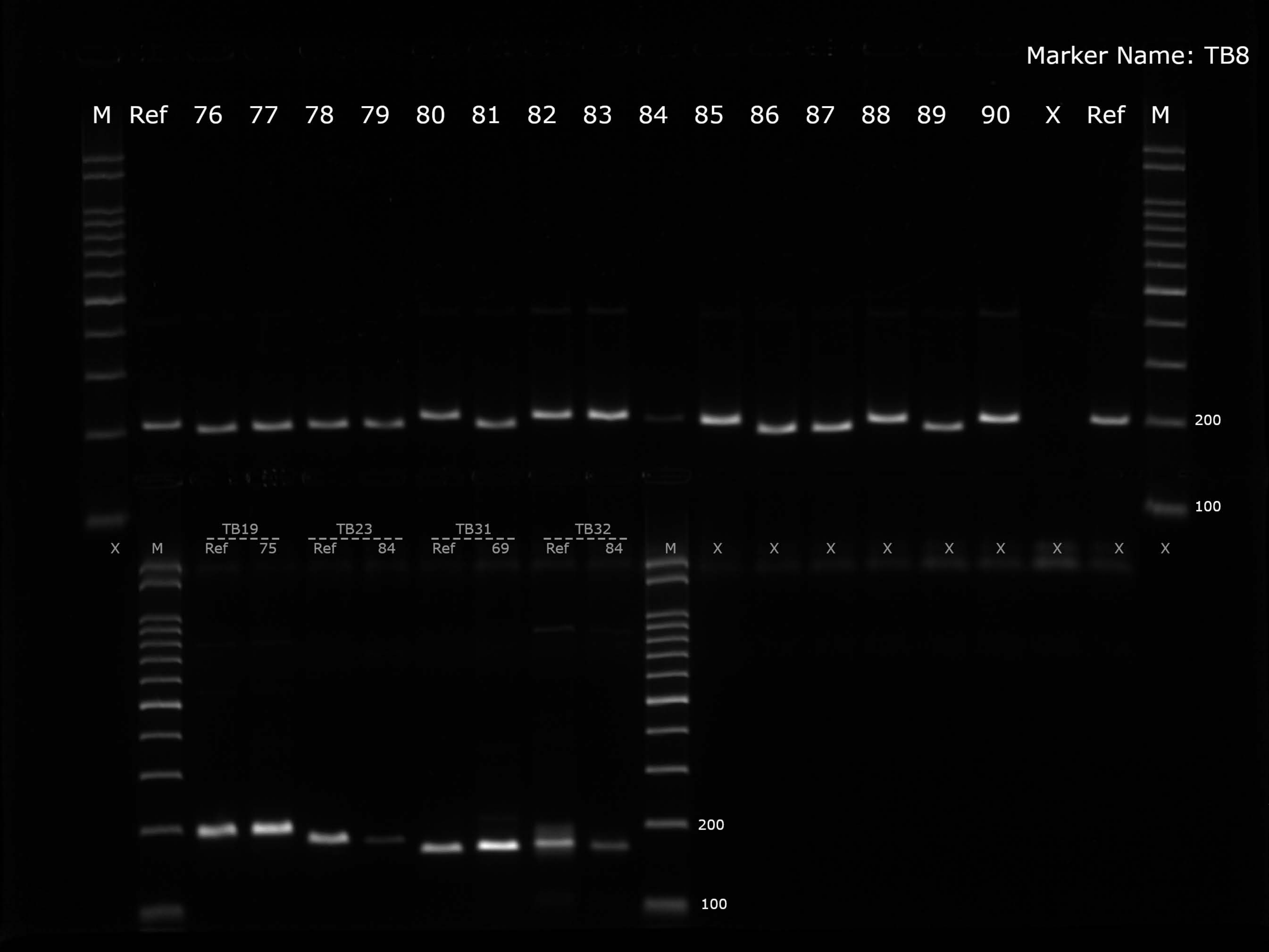
Marker Name: TB8
M Ref 76 77 78 79 80 81 82 83 84 85 86 87 88 89 90 X Ref M
200
100
TB19 TB23 TB31 TB32
X M Ref 75 Ref 84 Ref 69 Ref 84 M X X X X X X X X X
200
100

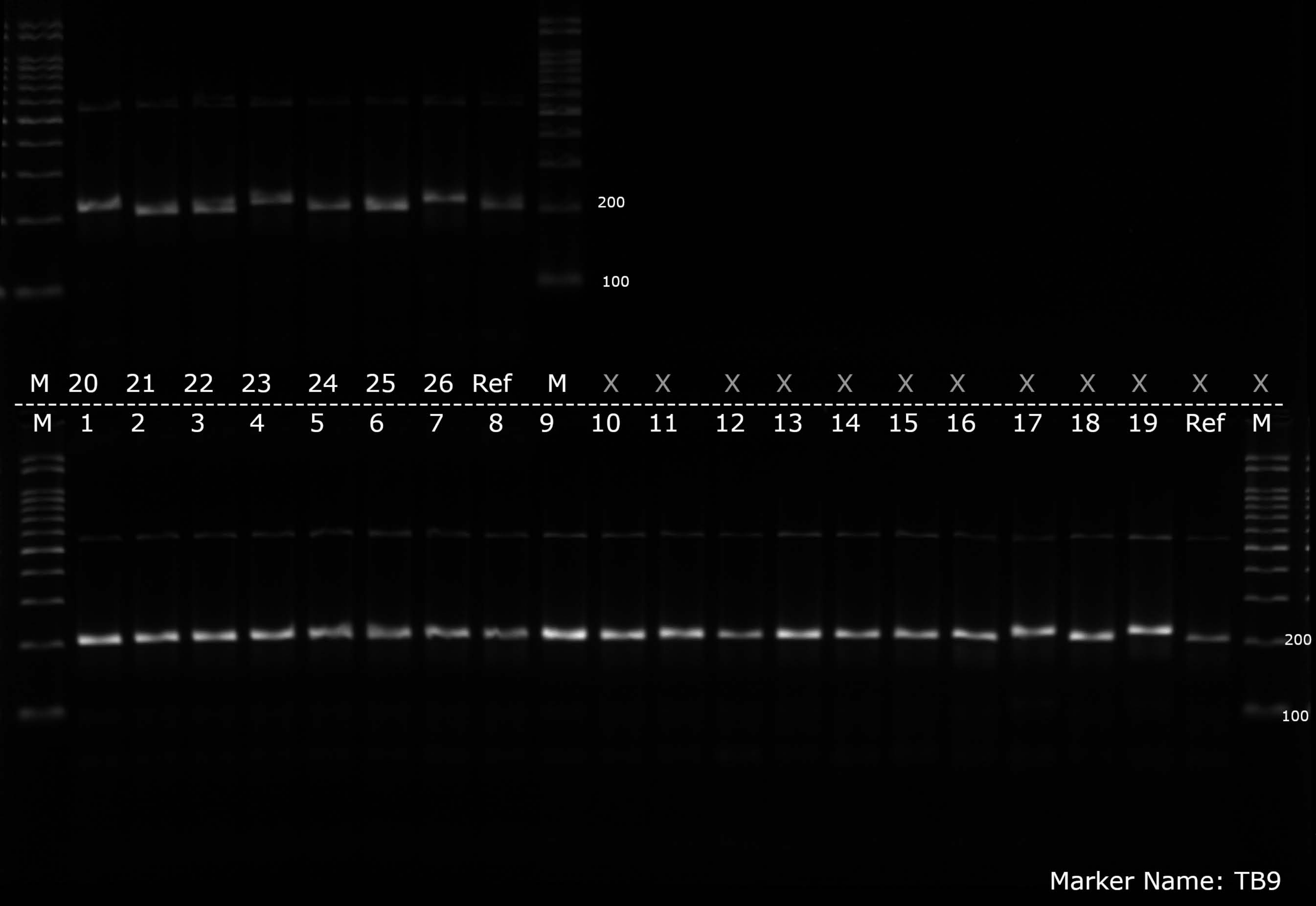
200
100
M 20 21 22 23 24 25 26 Ref M X X X X X X X X X X X X
M 1 2 3 4 5 6 7 8 9 10 11 12 13 14 15 16 17 18 19 Ref M
200
100
Marker Name: TB9

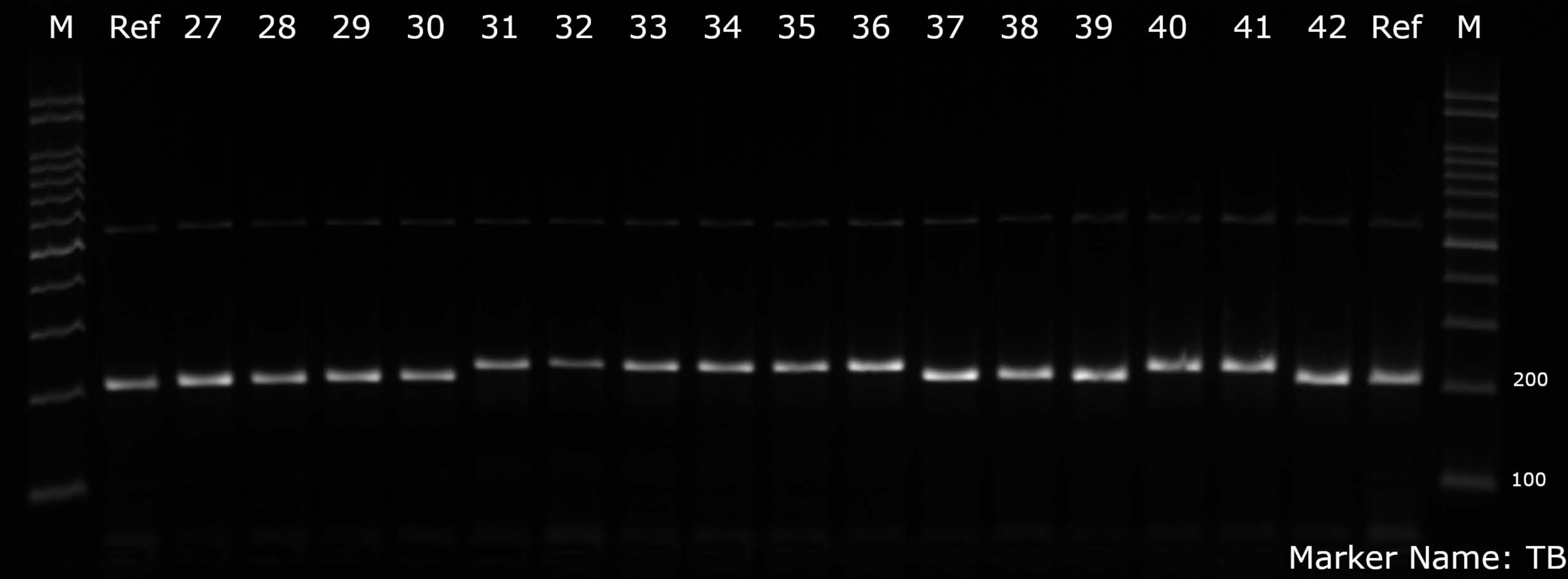

Marker Name: TB9

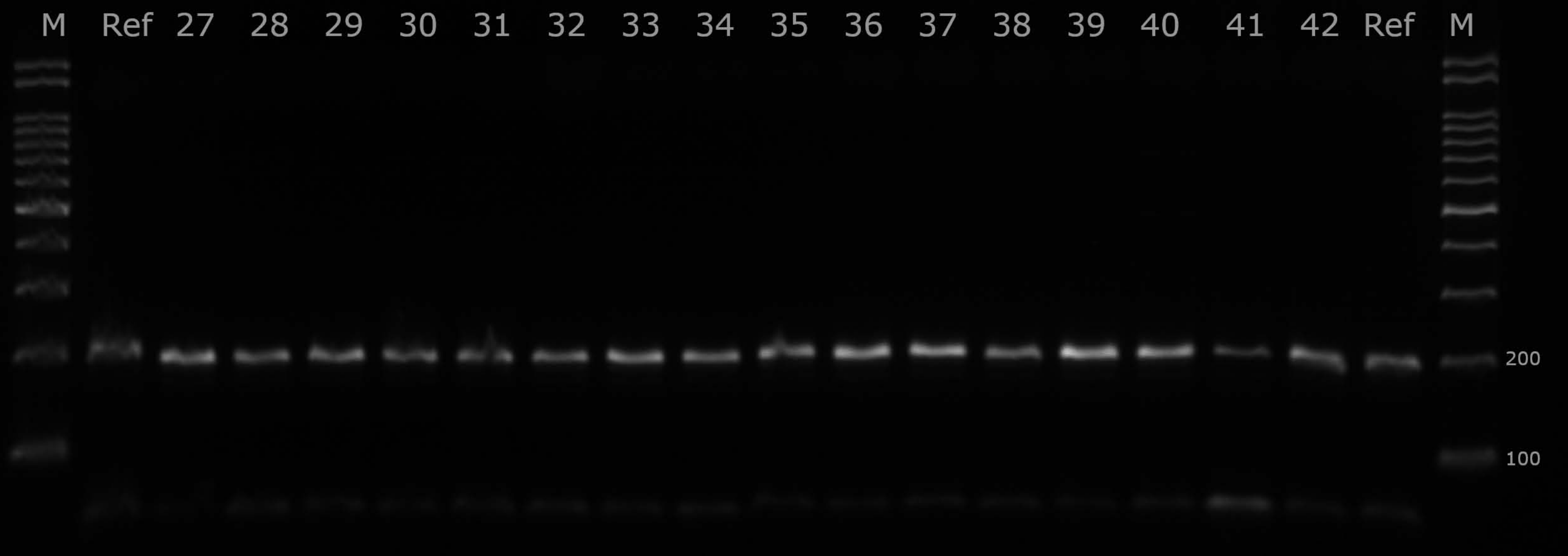

Marker Name: TB22

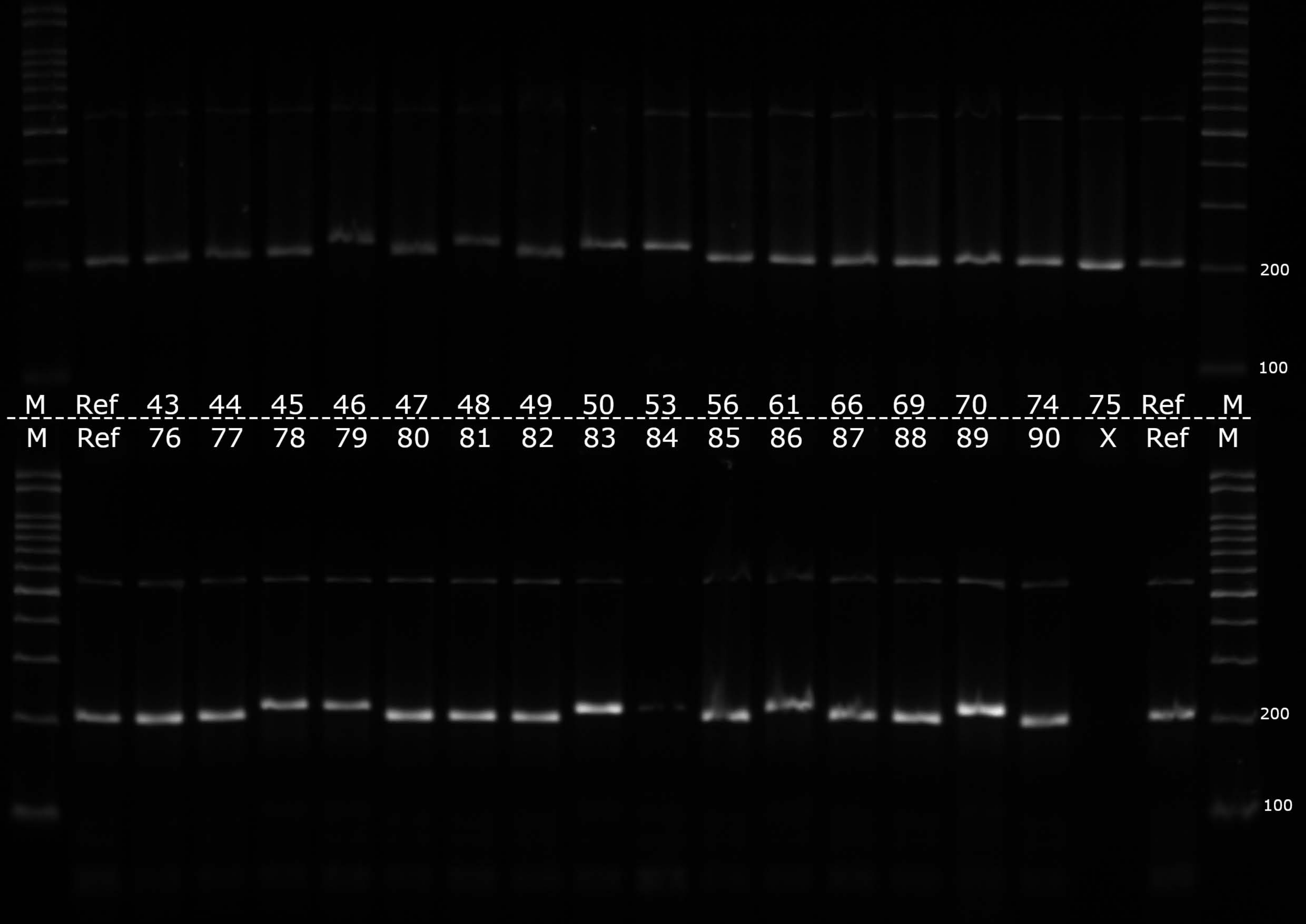

Marker Name: TB9

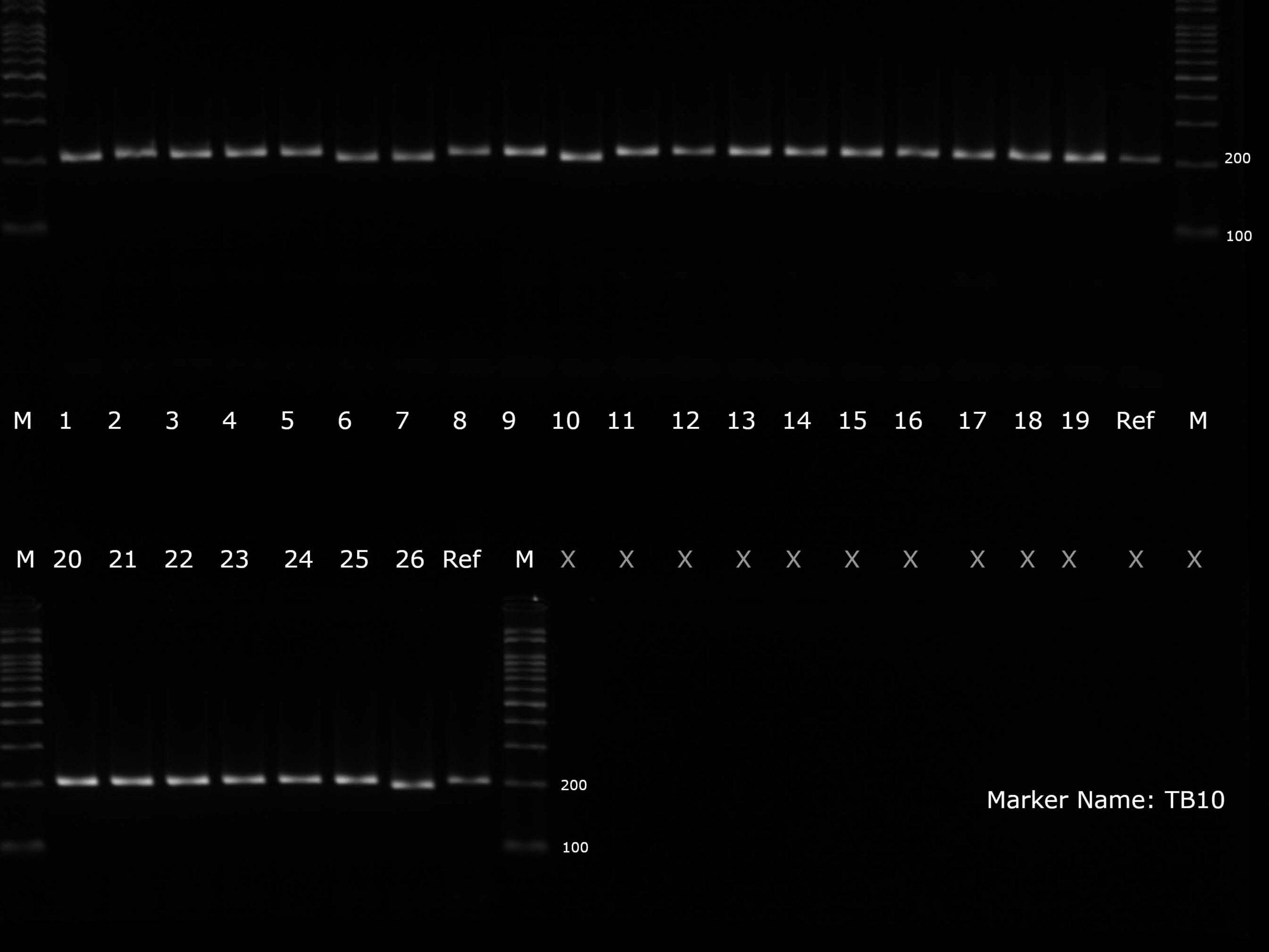
200
100
M 1 2 3 4 5 6 7 8 9 10 11 12 13 14 15 16 17 18 19 Ref M
M 20 21 22 23 24 25 26 Ref M X X X X X X X X X X X X
200
100
Marker Name: TB10

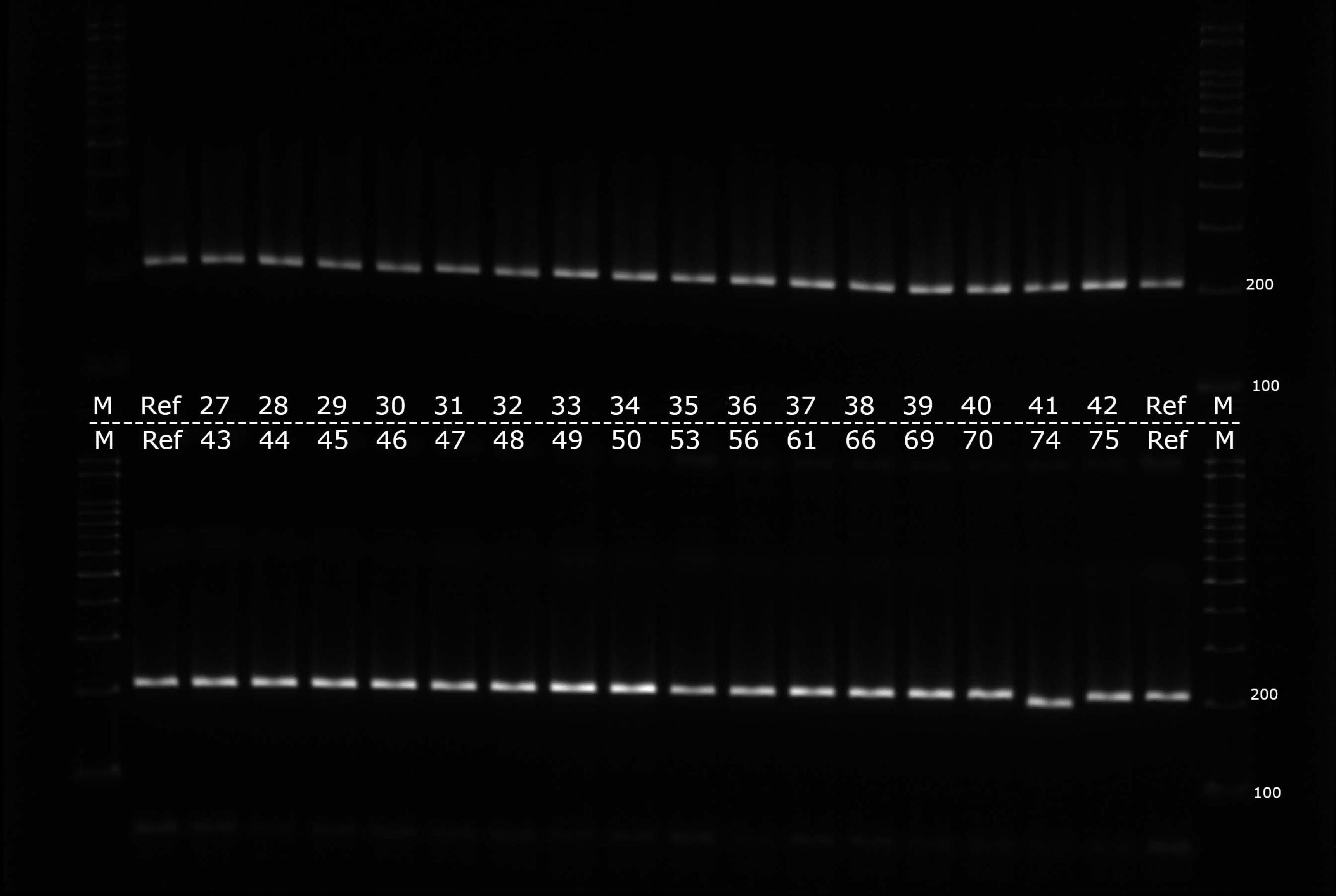

Marker Name: TB10

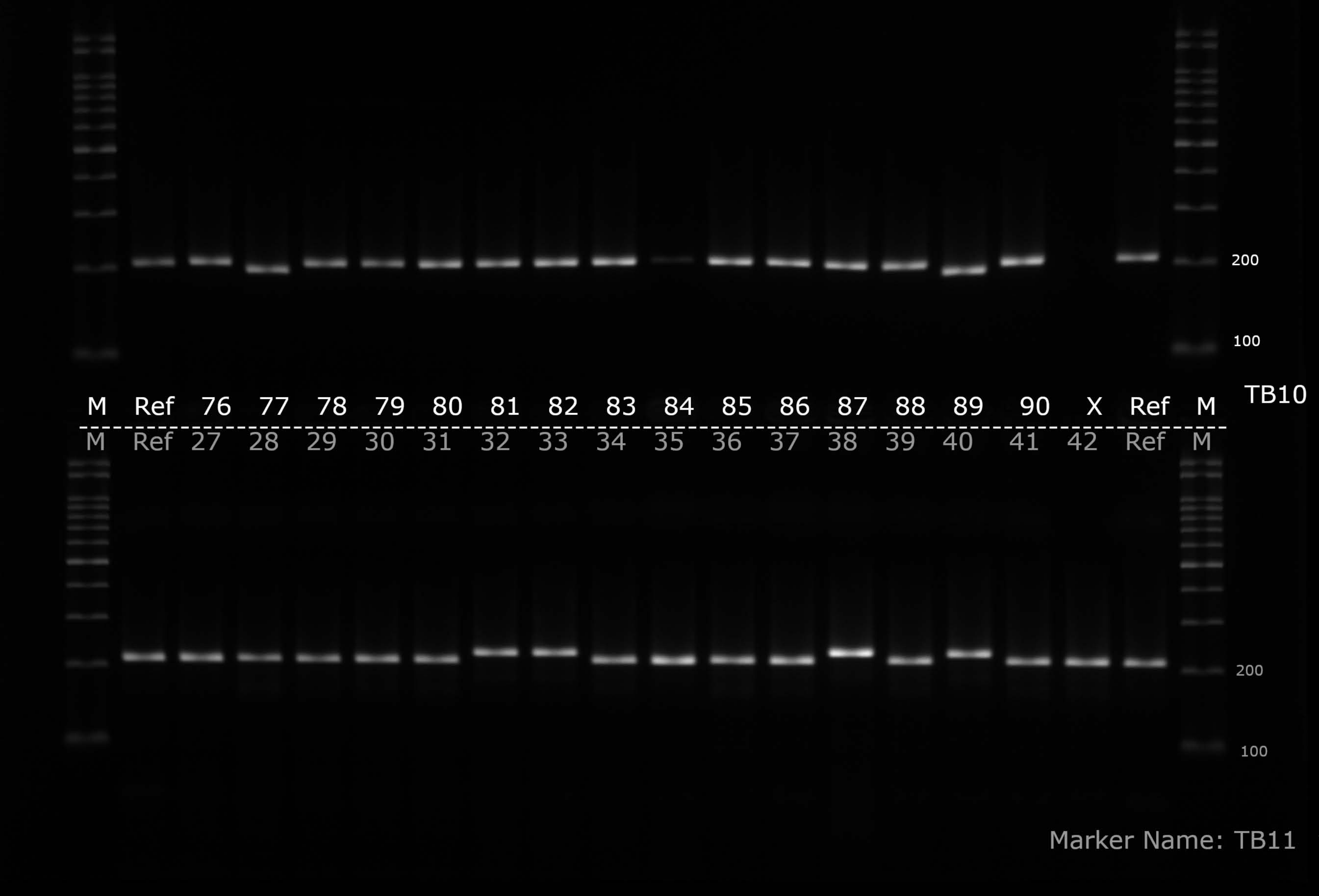

200
100
M Ref 76 77 78 79 80 81 82 83 84 85 86 87 88 89 90 X Ref M
TB10
M Ref 27 28 29 30 31 32 33 34 35 36 37 38 39 40 41 42 Ref M
200
100
Marker Name: TB11

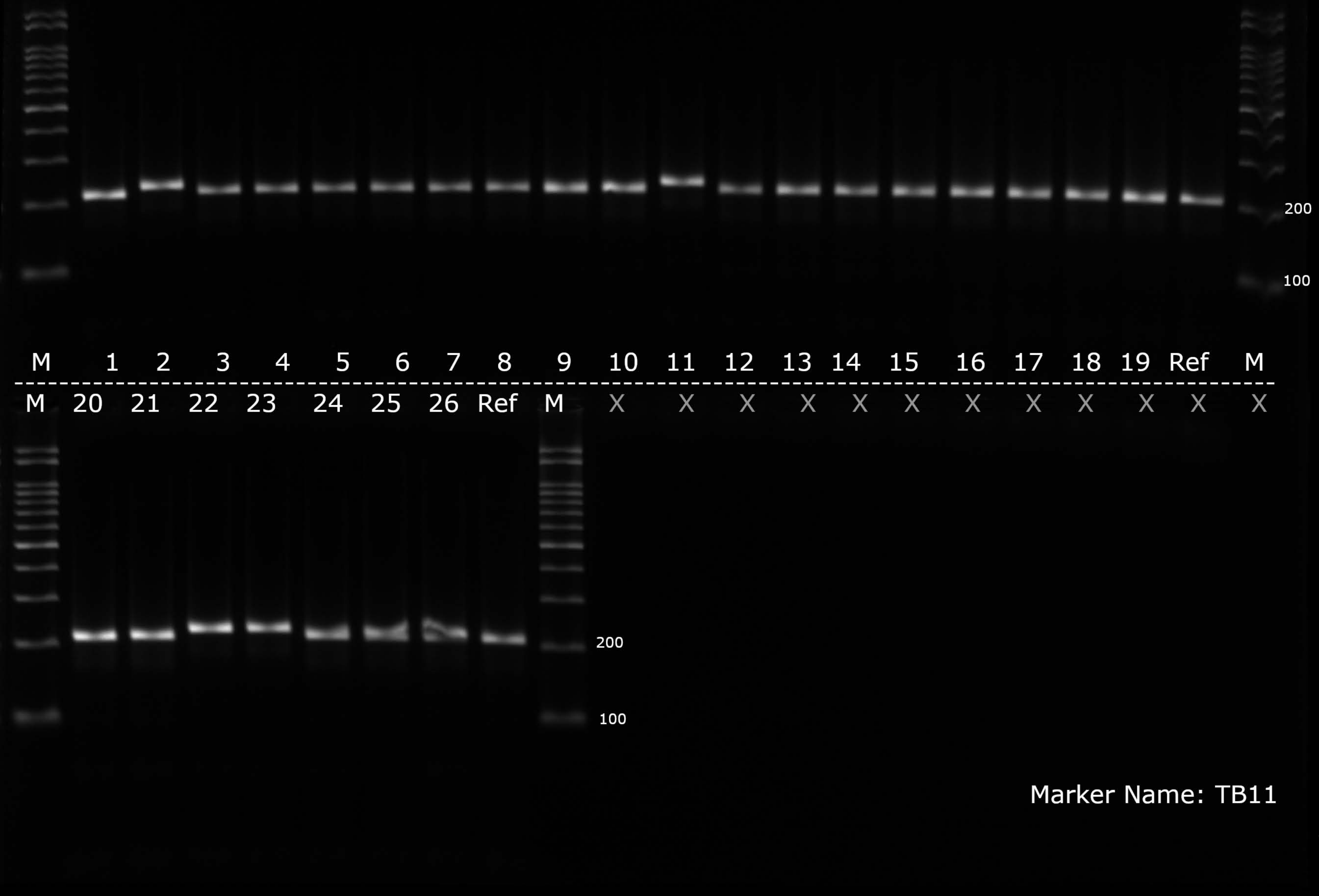
M 1 2 3 4 5 6 7 8 9 10 11 12 13 14 15 16 17 18 19 Ref M
200
100
M 20 21 22 23 24 25 26 Ref M X X X X X X X X X X X X
200
100
Marker Name: TB11

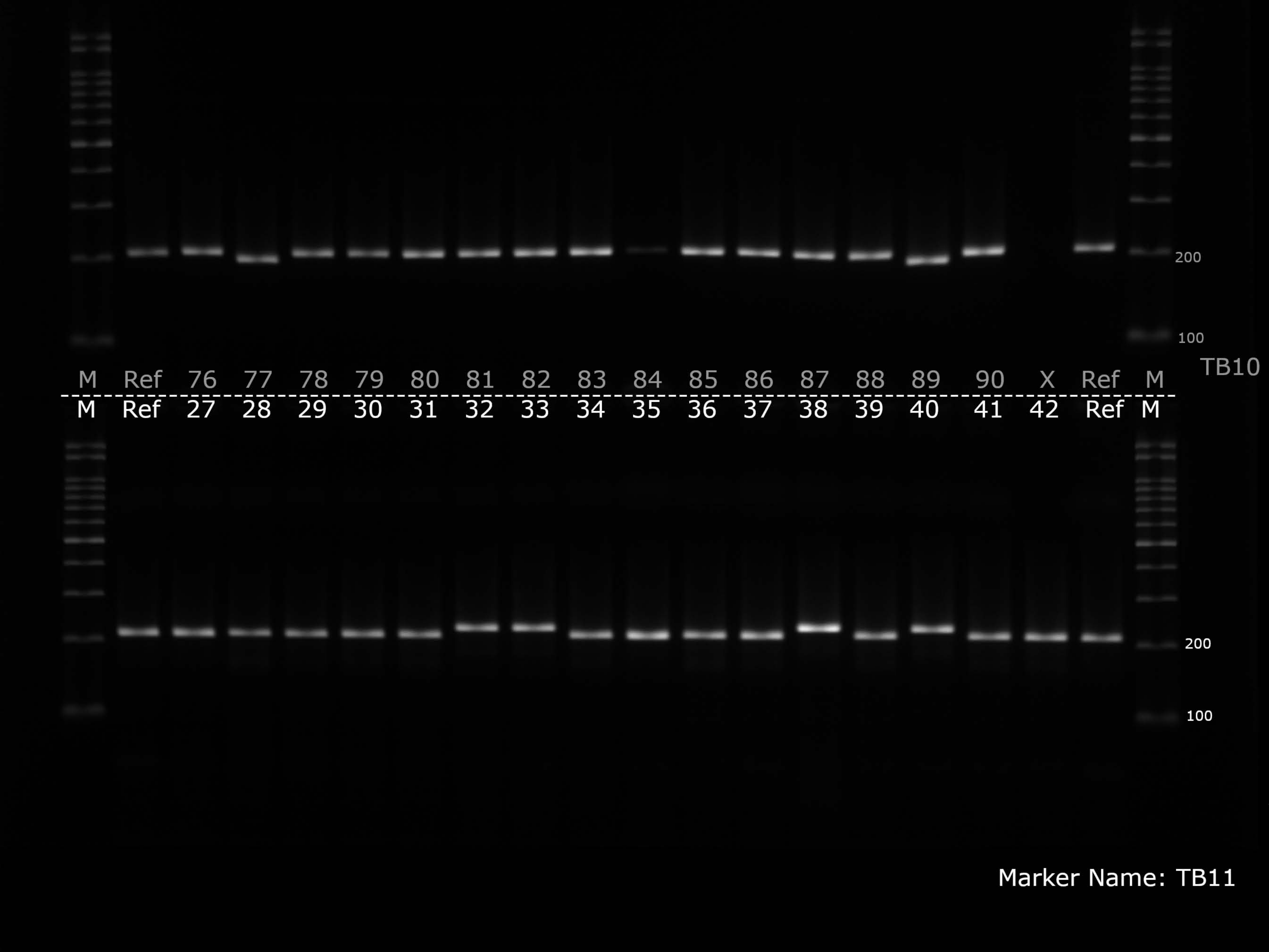
200
100
TB10
M Ref 76 77 78 79 80 81 82 83 84 85 86 87 88 89 90 X Ref M
M Ref 27 28 29 30 31 32 33 34 35 36 37 38 39 40 41 42 Ref M
200
100
Marker Name: TB11

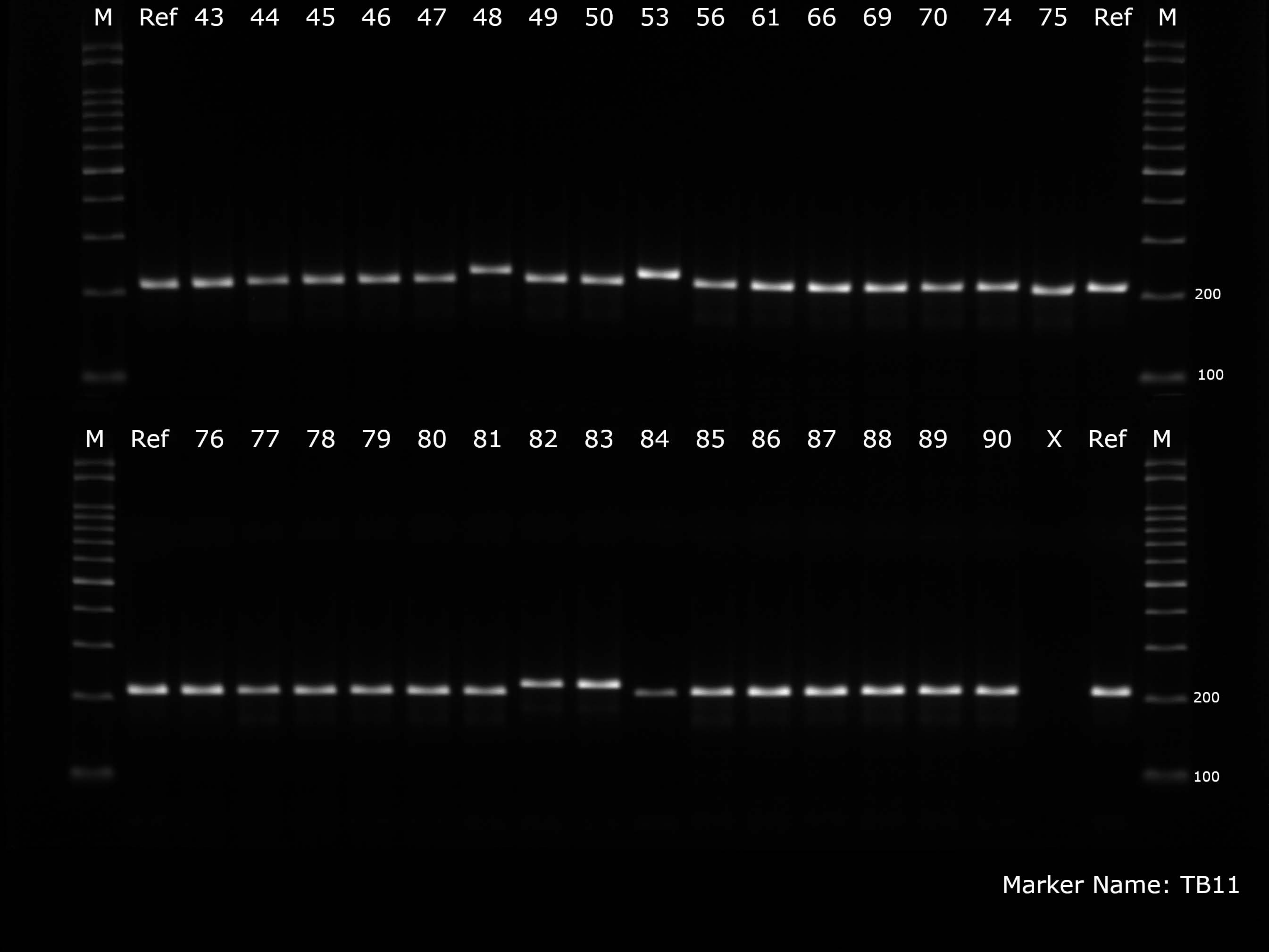
M Ref 43 44 45 46 47 48 49 50 53 56 61 66 69 70 74 75 Ref M
200
100
M Ref 76 77 78 79 80 81 82 83 84 85 86 87 88 89 90 X Ref M
200
100
Marker Name: TB11

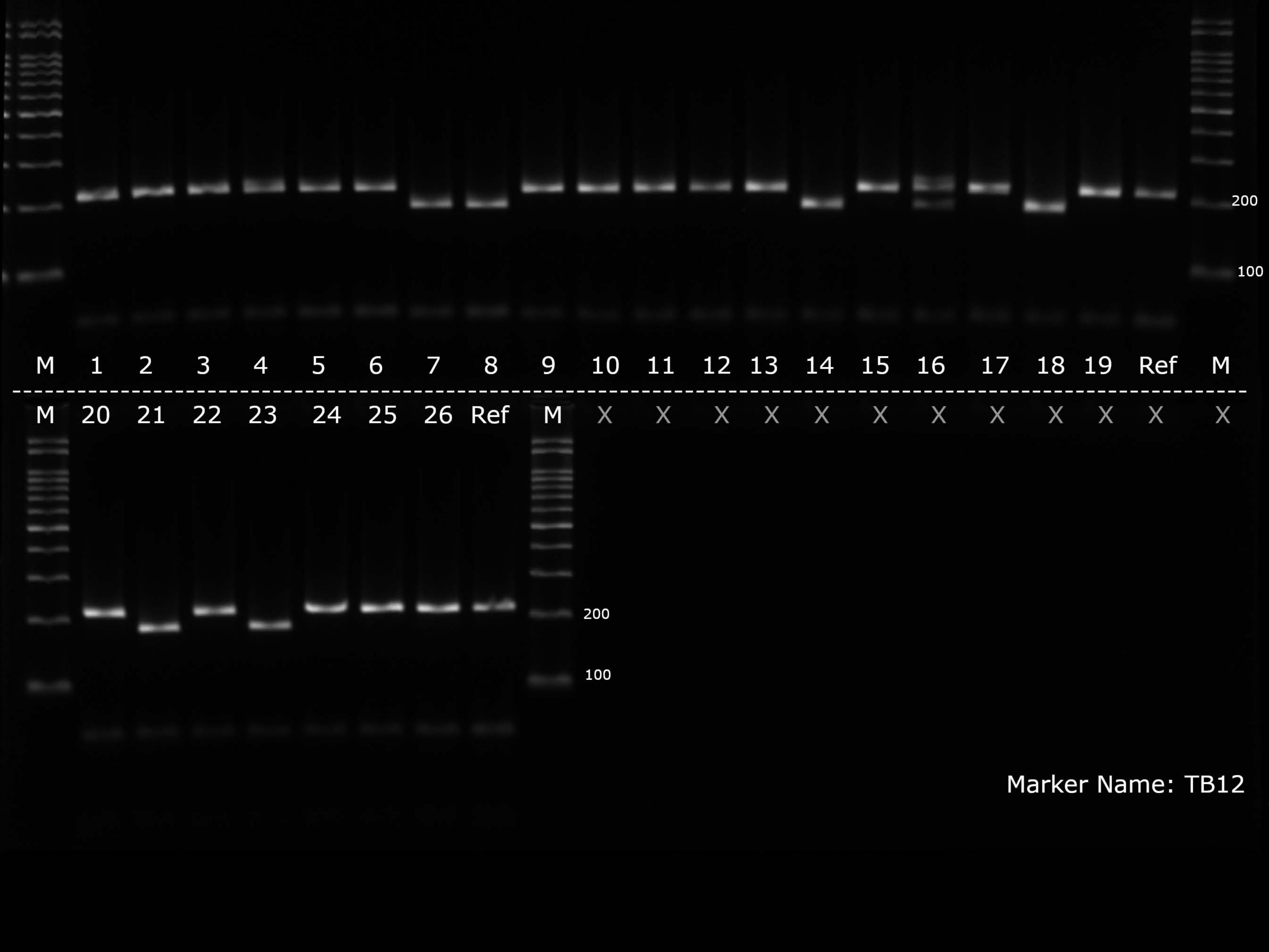
M 1 2 3 4 5 6 7 8 9 10 11 12 13 14 15 16 17 18 19 Ref M
200
100
M 20 21 22 23 24 25 26 Ref M X X X X X X X X X X X X
200
100
Marker Name: TB12

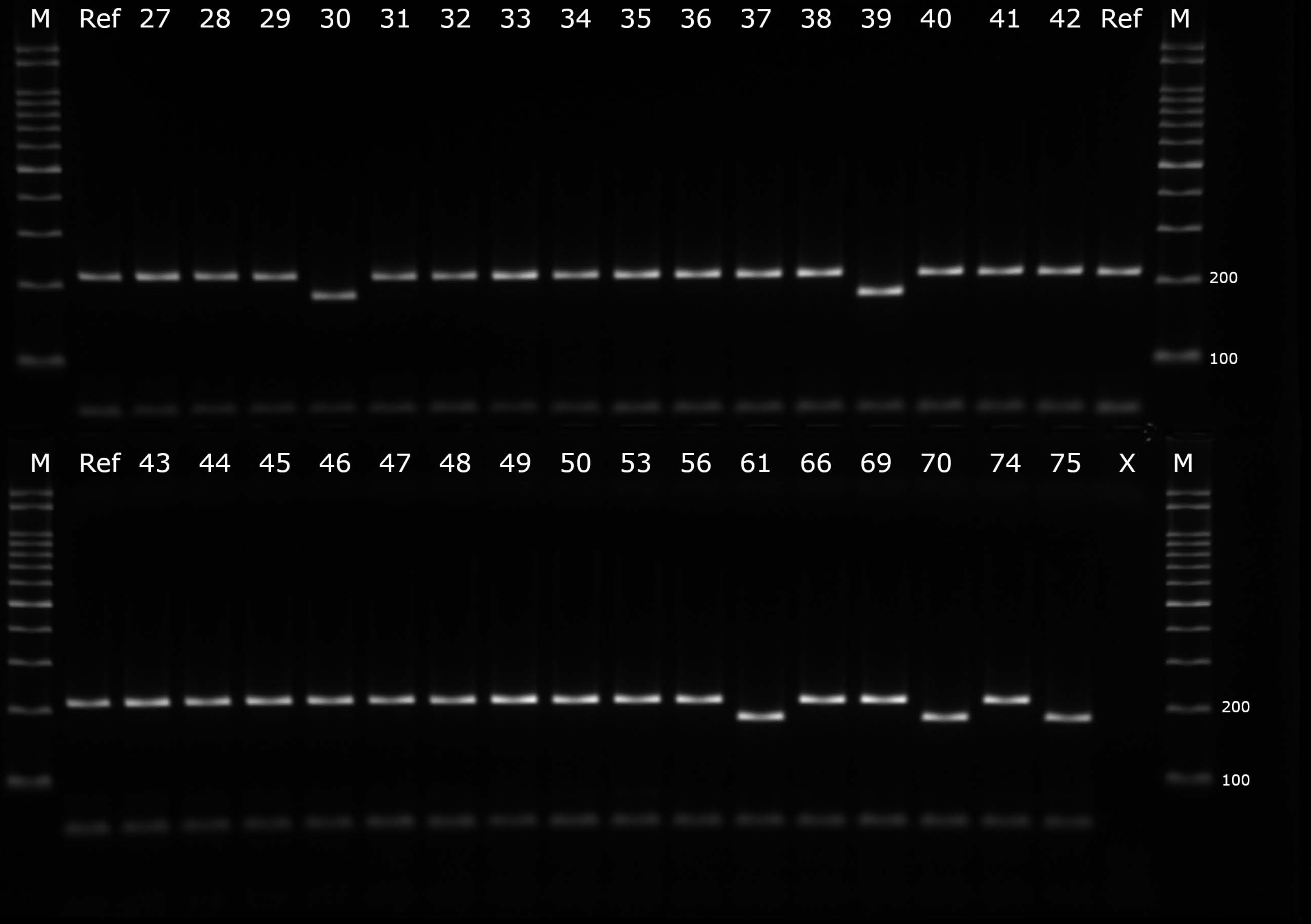

Marker Name: TB12

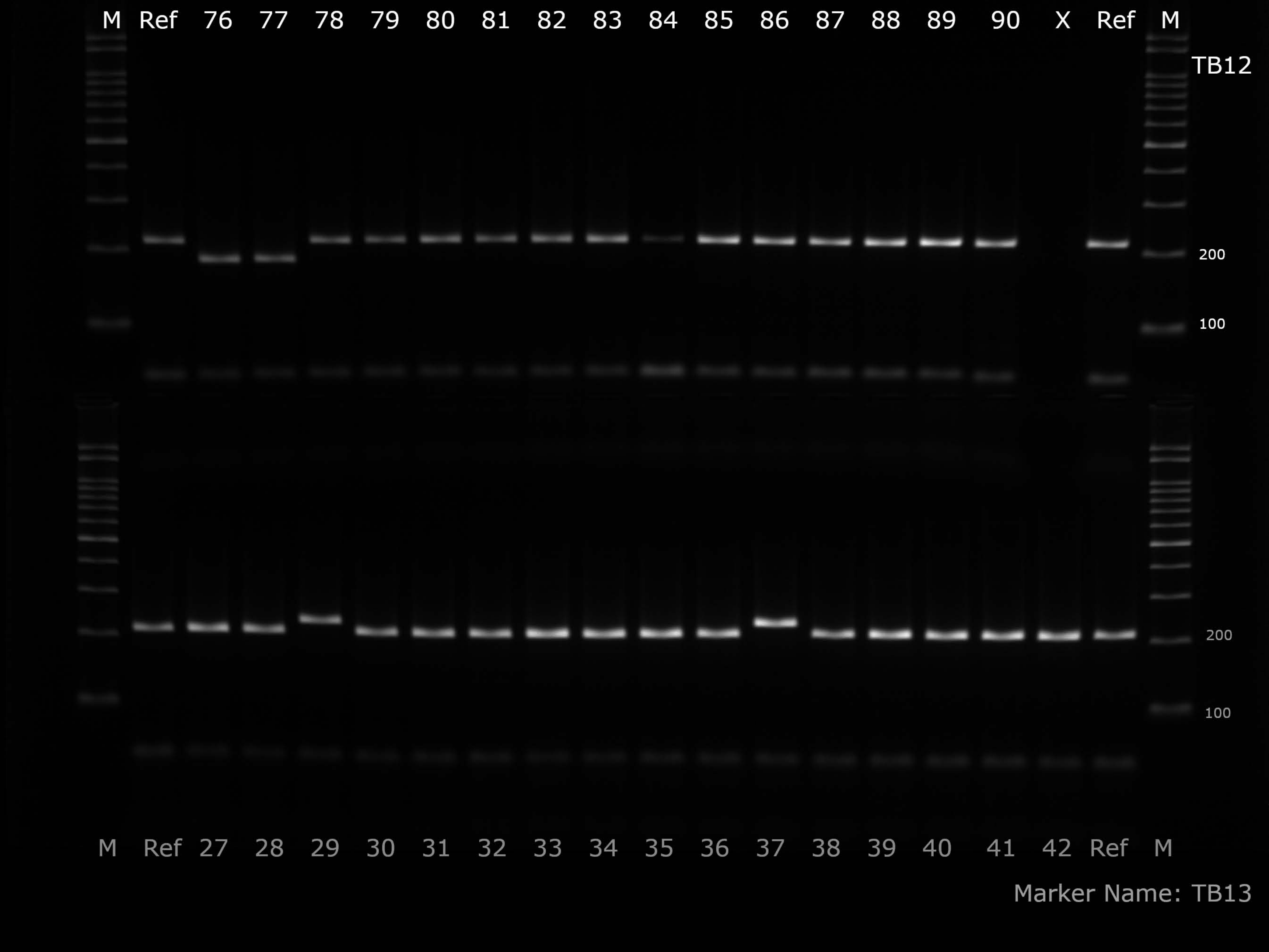
M Ref 76 77 78 79 80 81 82 83 84 85 86 87 88 89 90 X Ref M
TB12
200
100
200
100
M Ref 27 28 29 30 31 32 33 34 35 36 37 38 39 40 41 42 Ref M
Marker Name: TB13

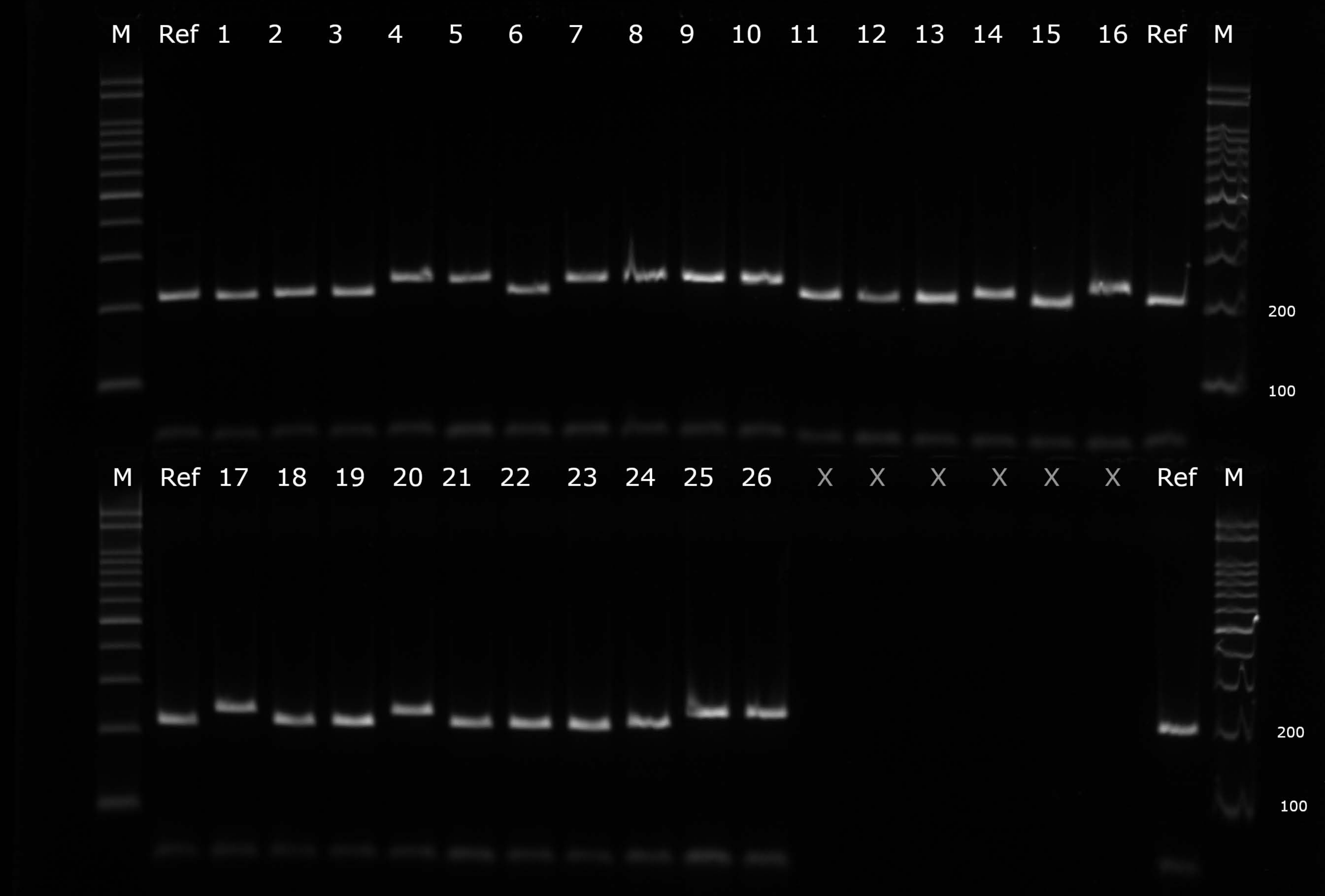

Marker Name: TB13

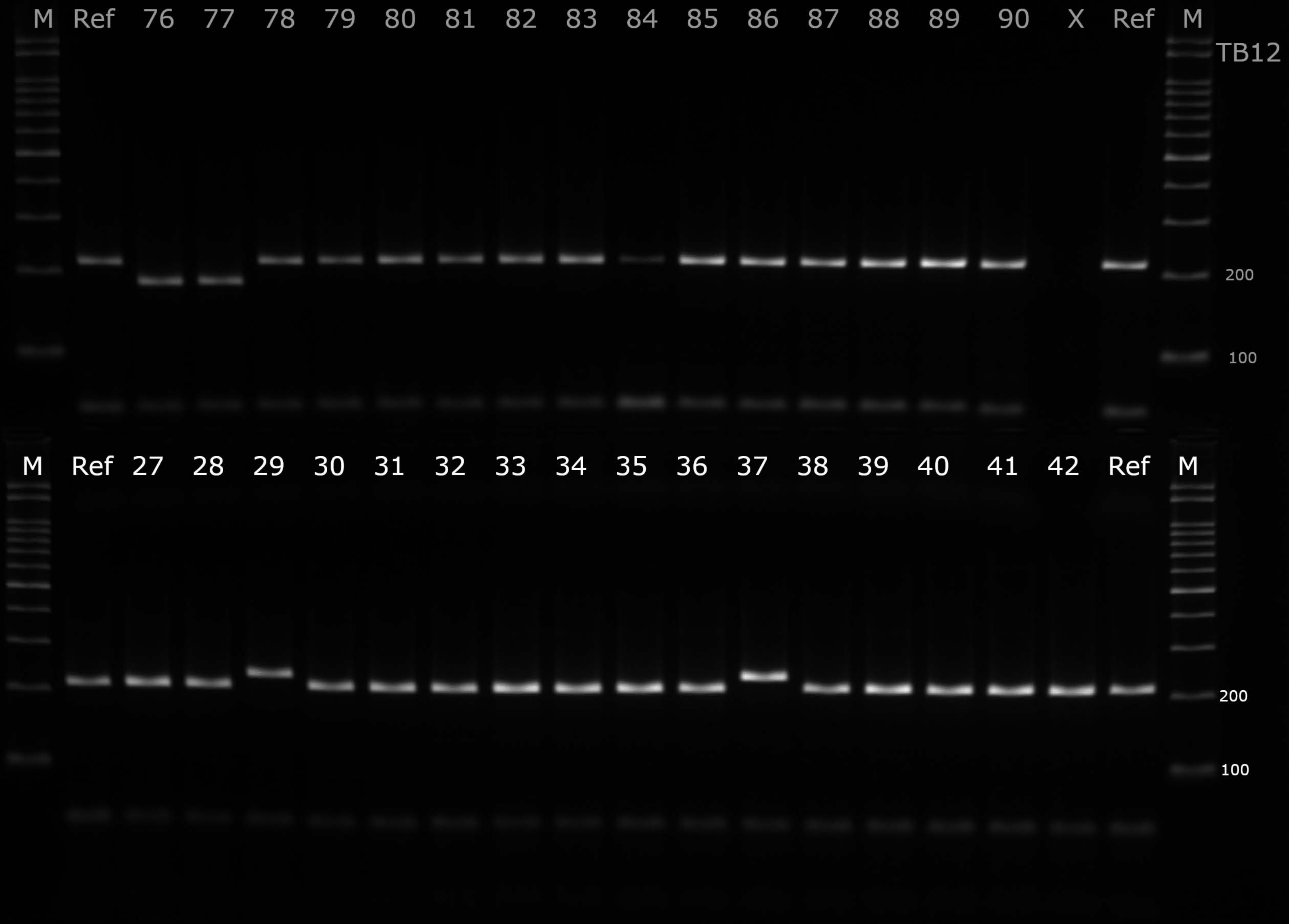

Marker Name: TB13

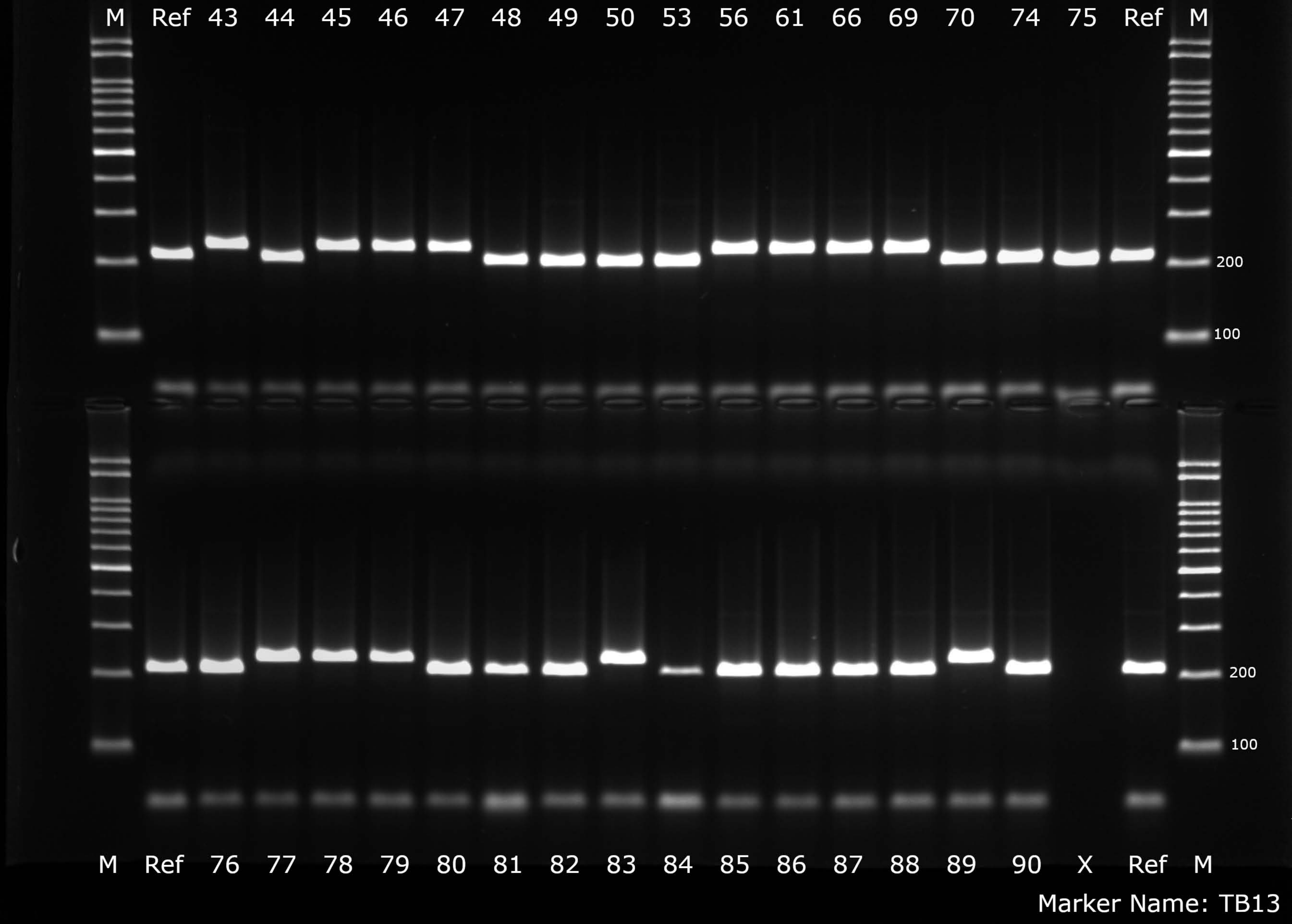
M Ref 43 44 45 46 47 48 49 50 53 56 61 66 69 70 74 75 Ref M
200
100
200
100
M Ref 76 77 78 79 80 81 82 83 84 85 86 87 88 89 90 X Ref M
Marker Name: TB13

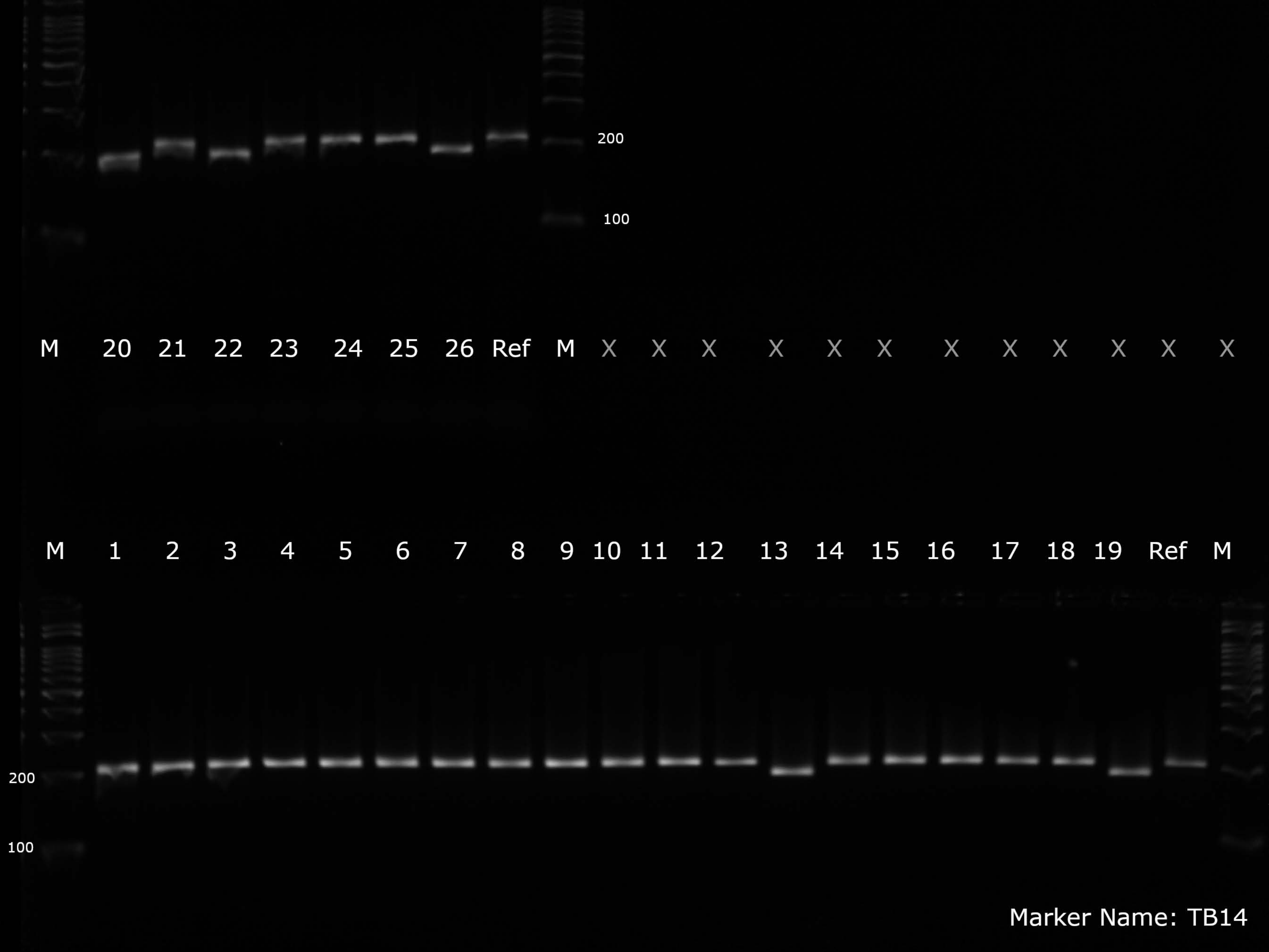
200
100
M 20 21 22 23 24 25 26 Ref M X X X X X X X X X X X X
M 1 2 3 4 5 6 7 8 9 10 11 12 13 14 15 16 17 18 19 Ref M
200
100
Marker Name: TB14

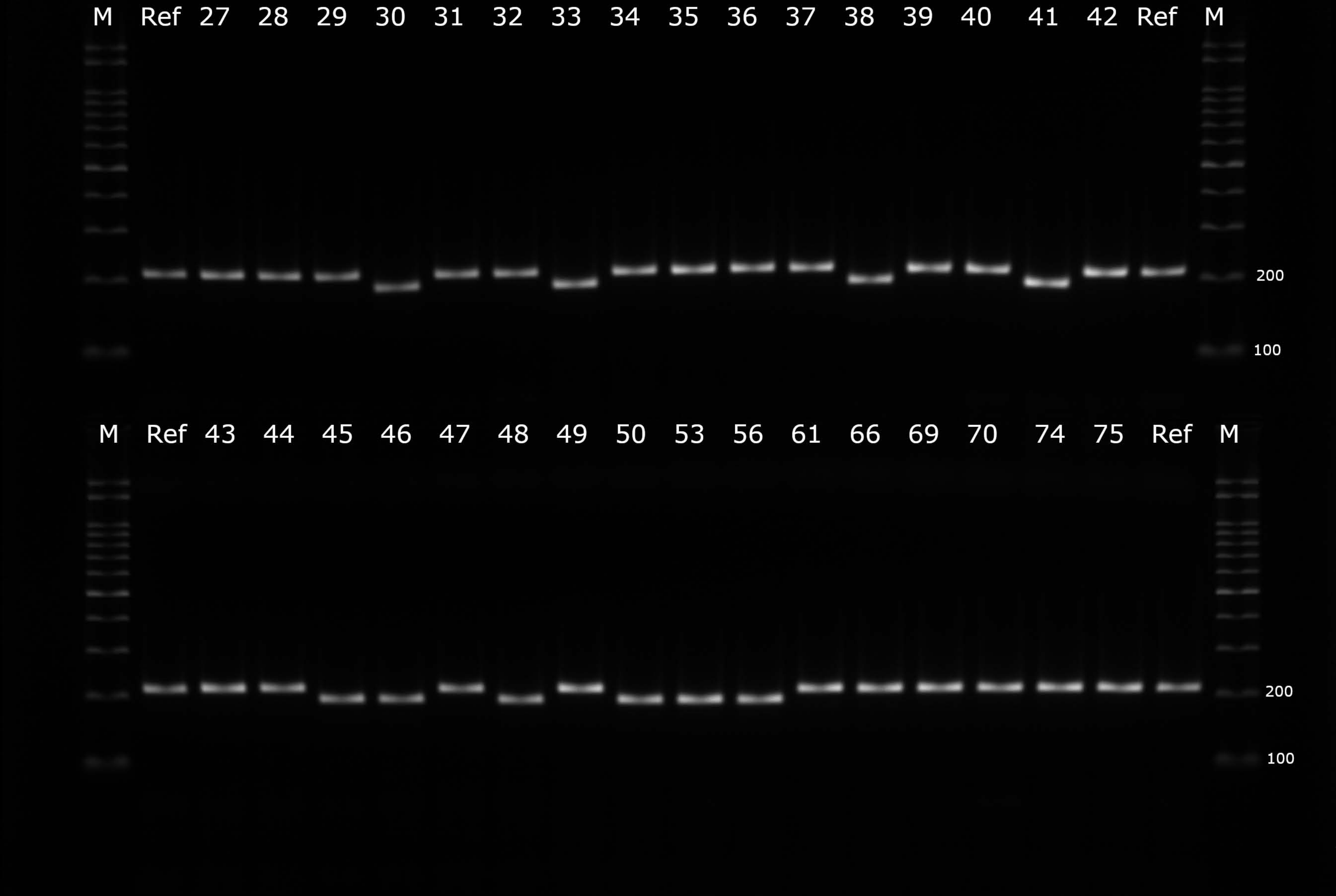

Marker Name: TB14

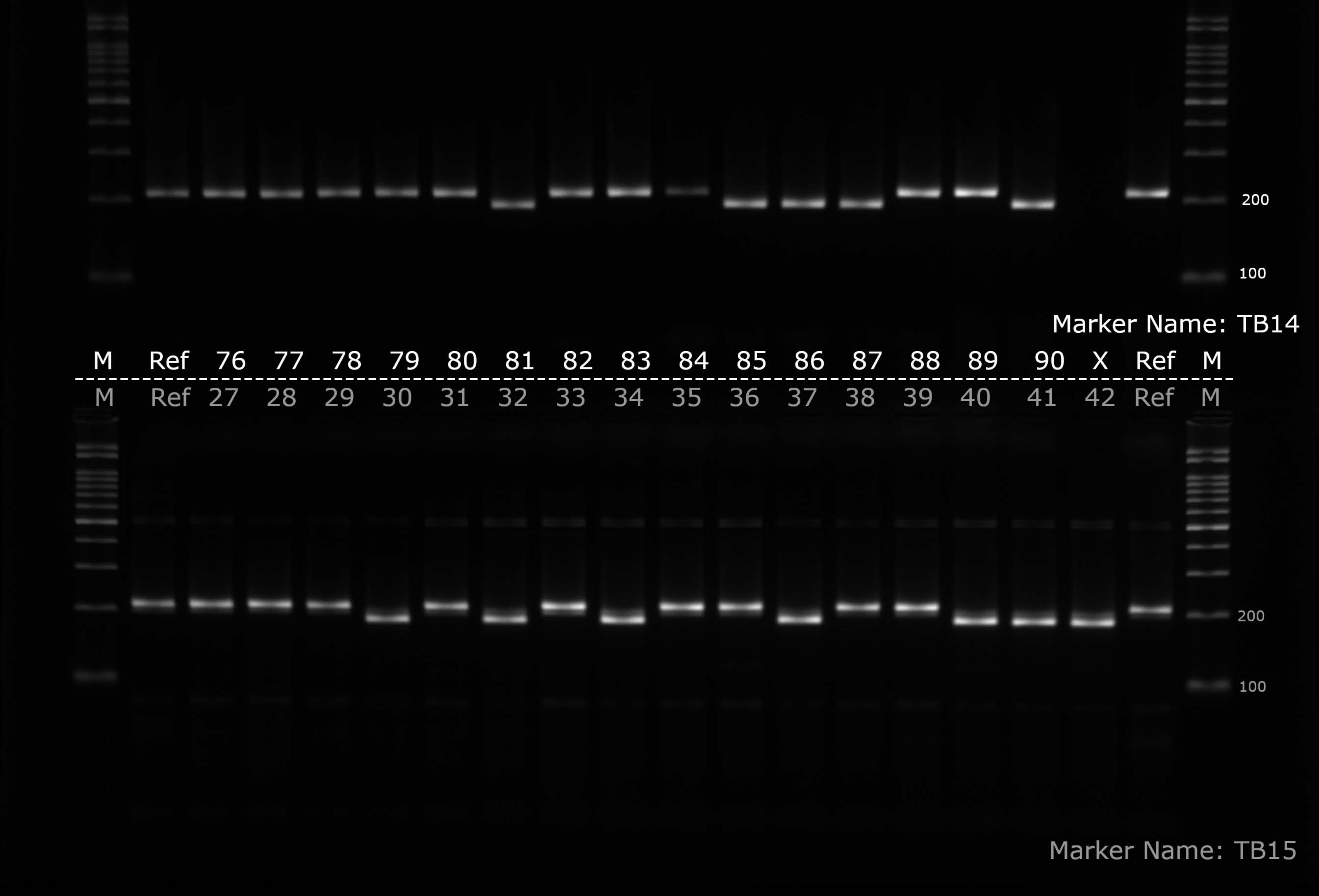
200
100
Marker Name: TB14
M Ref 76 77 78 79 80 81 82 83 84 85 86 87 88 89 90 X Ref M
M Ref 27 28 29 30 31 32 33 34 35 36 37 38 39 40 41 42 Ref M
200
100
Marker Name: TB15

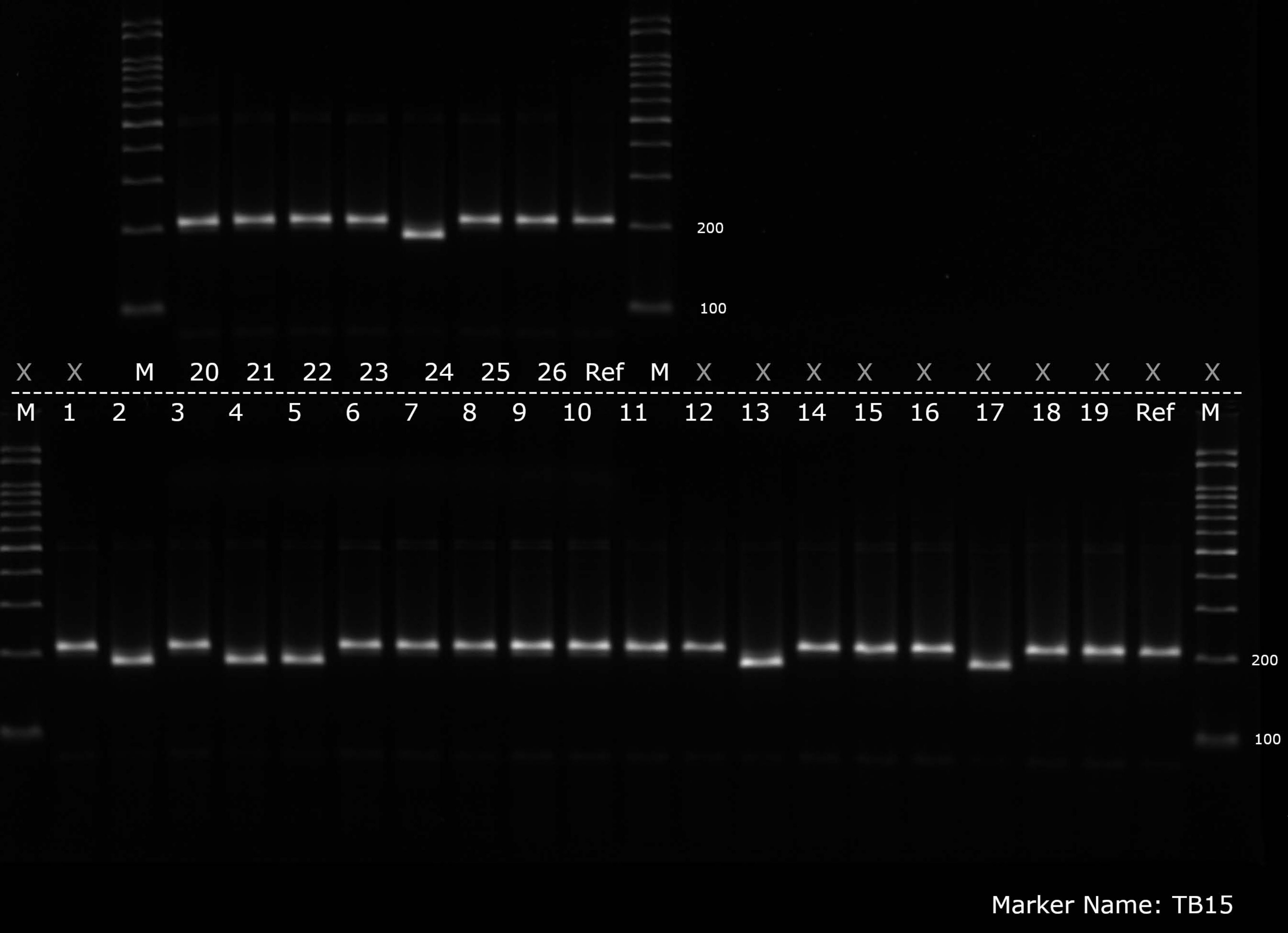
200
100
X X M 20 21 22 23 24 25 26 Ref M X X X X X X X X X X
M 1 2 3 4 5 6 7 8 9 10 11 12 13 14 15 16 17 18 19 Ref M
200
100
Marker Name: TB15

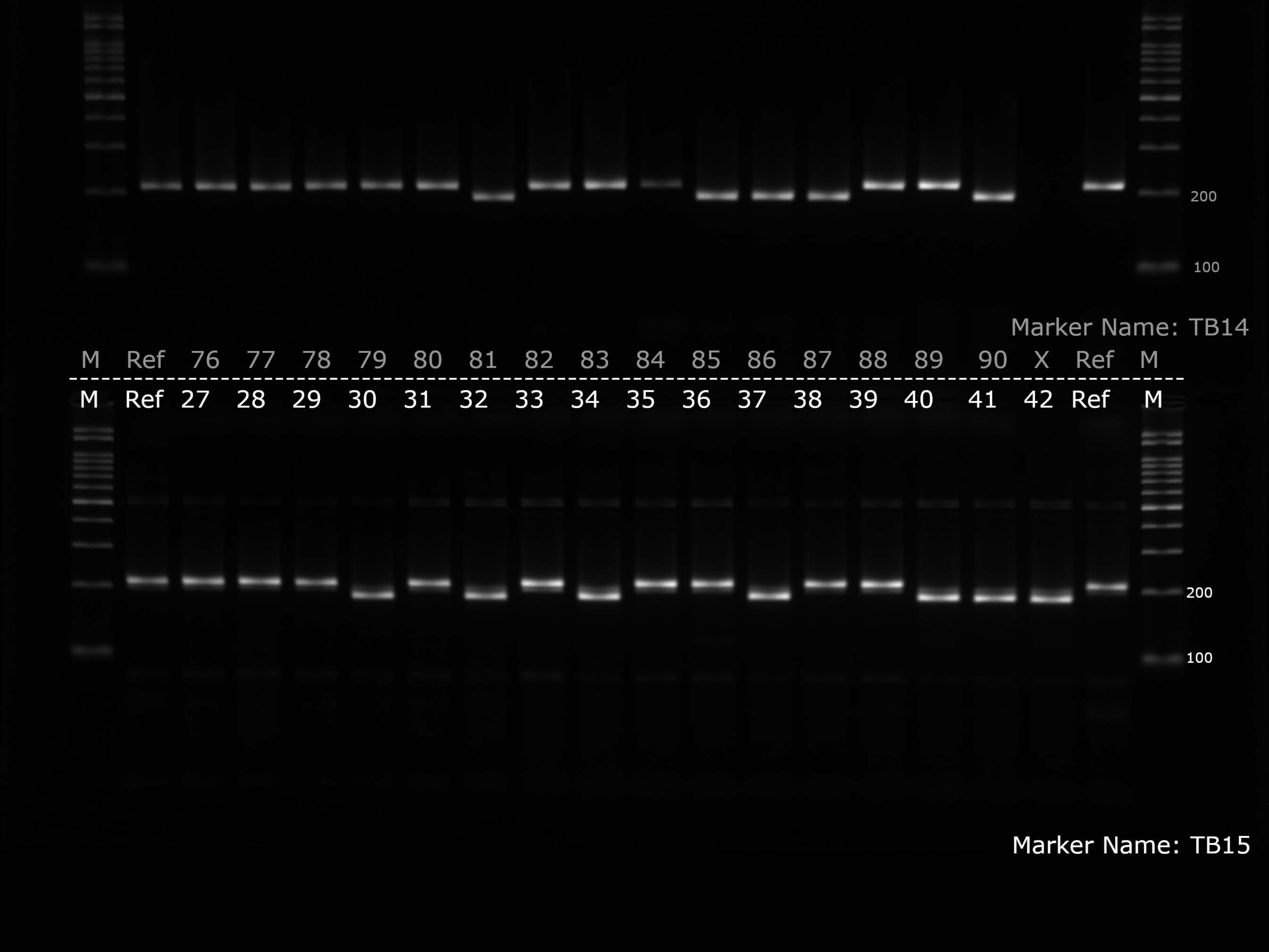
200
100
Marker Name: TB14
M Ref 76 77 78 79 80 81 82 83 84 85 86 87 88 89 90 X Ref M
M Ref 27 28 29 30 31 32 33 34 35 36 37 38 39 40 41 42 Ref M
200
100
Marker Name: TB15

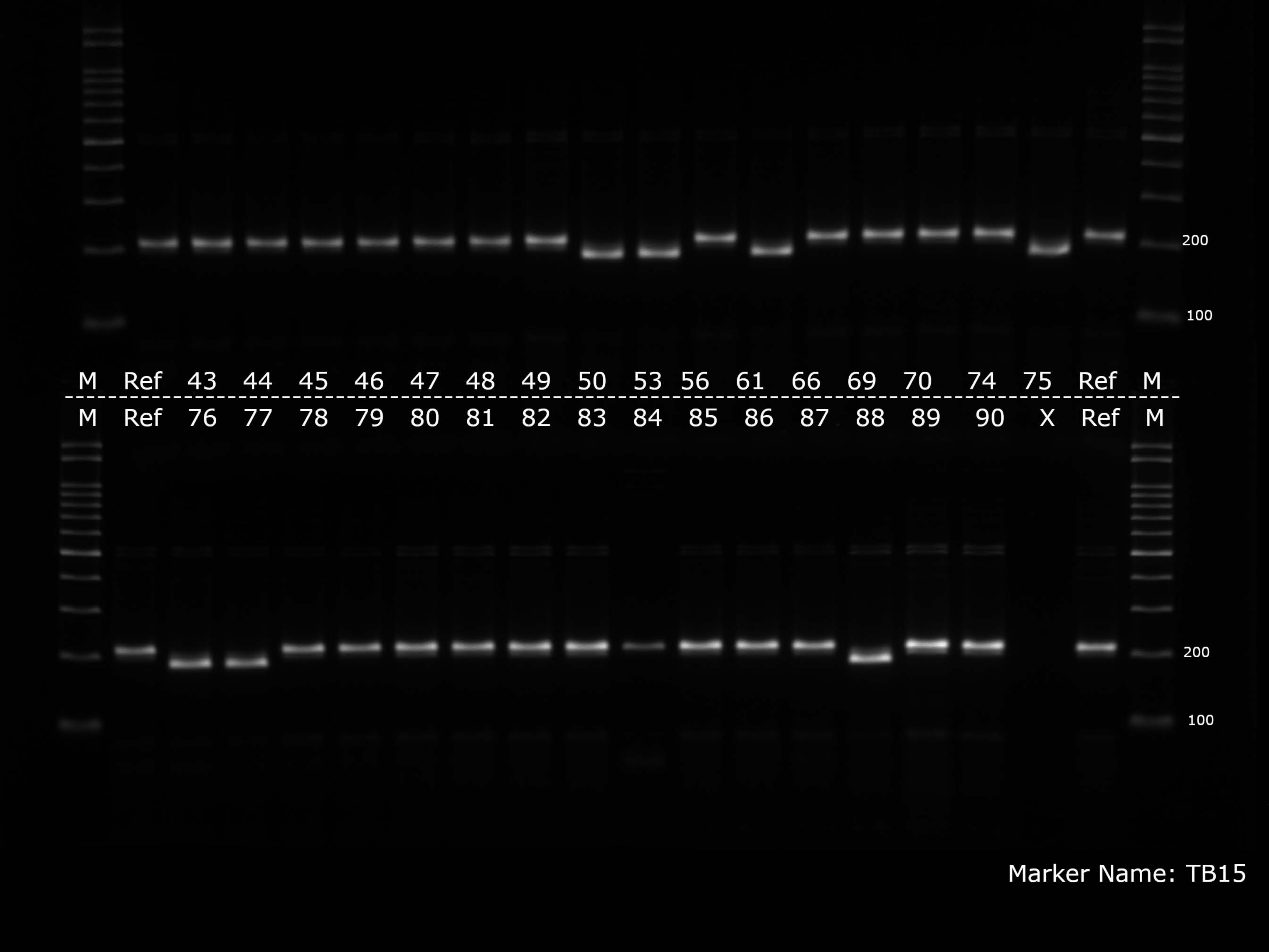
200
100
M Ref 43 44 45 46 47 48 49 50 53 56 61 66 69 70 74 75 Ref M
M Ref 76 77 78 79 80 81 82 83 84 85 86 87 88 89 90 X Ref M
200
100
Marker Name: TB15

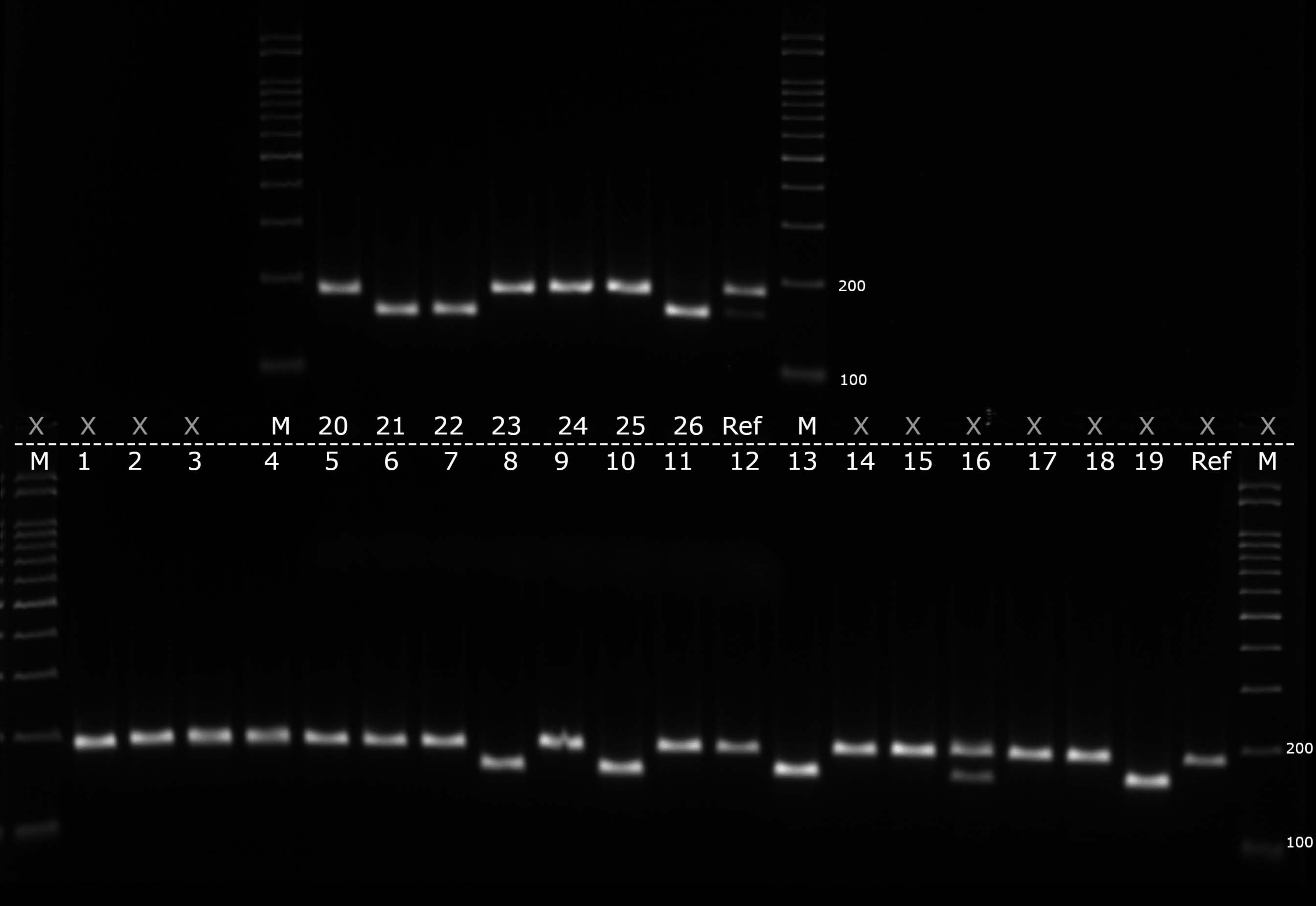

Marker Name: TB16

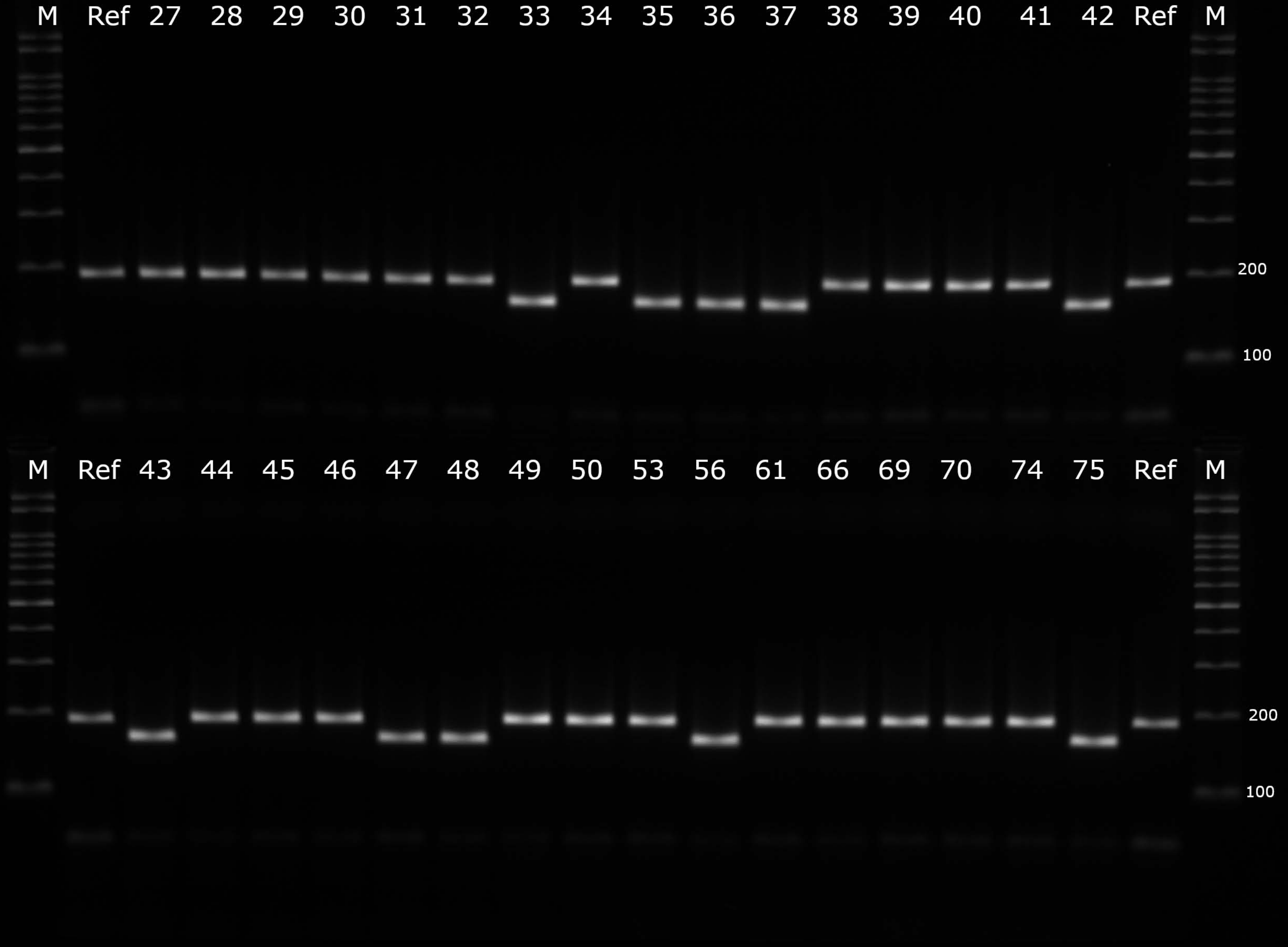

Marker Name: TB16

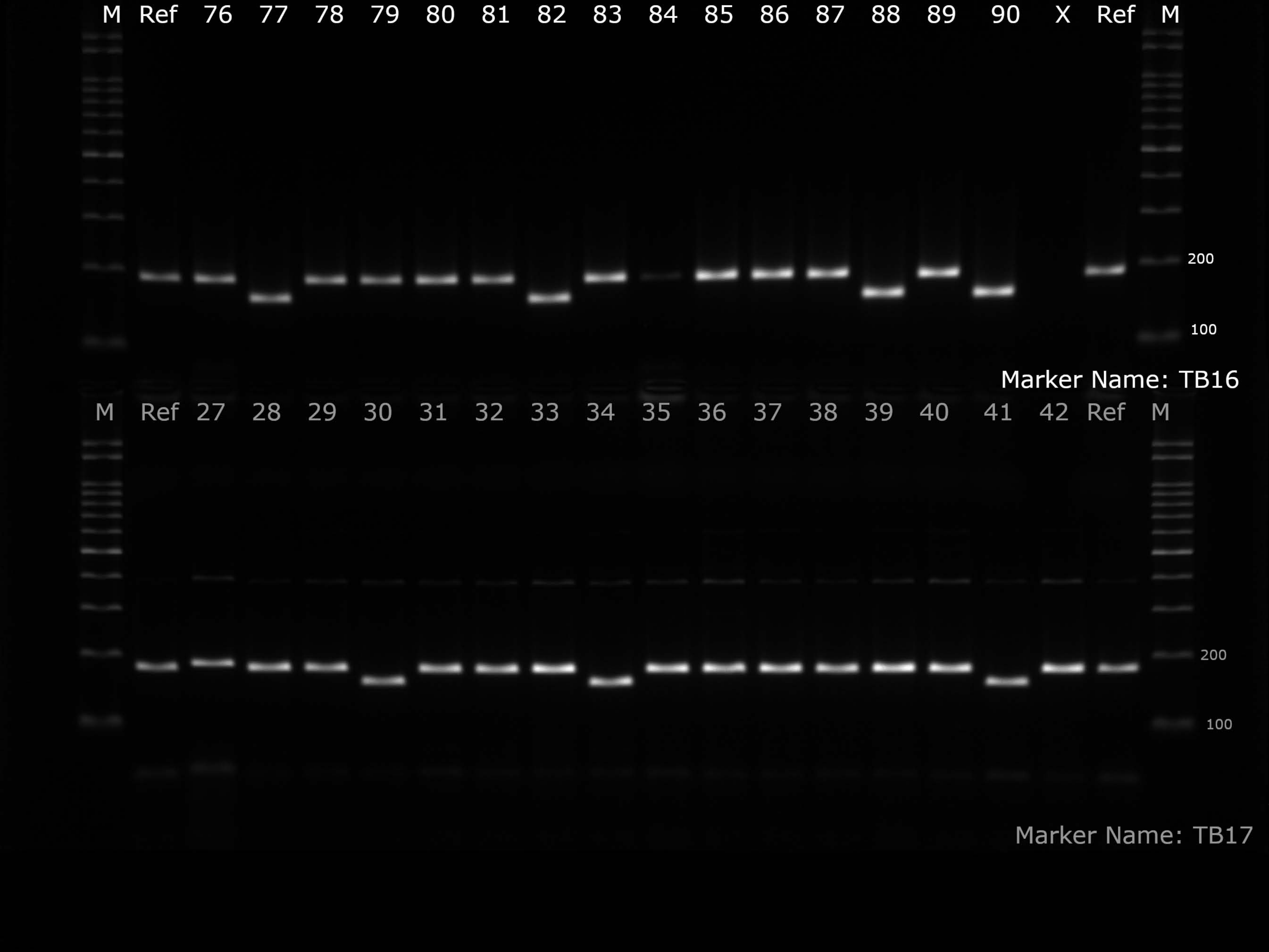
M Ref 76 77 78 79 80 81 82 83 84 85 86 87 88 89 90 X Ref M
200
100
Marker Name: TB16
M Ref 27 28 29 30 31 32 33 34 35 36 37 38 39 40 41 42 Ref M
200
100
Marker Name: TB17

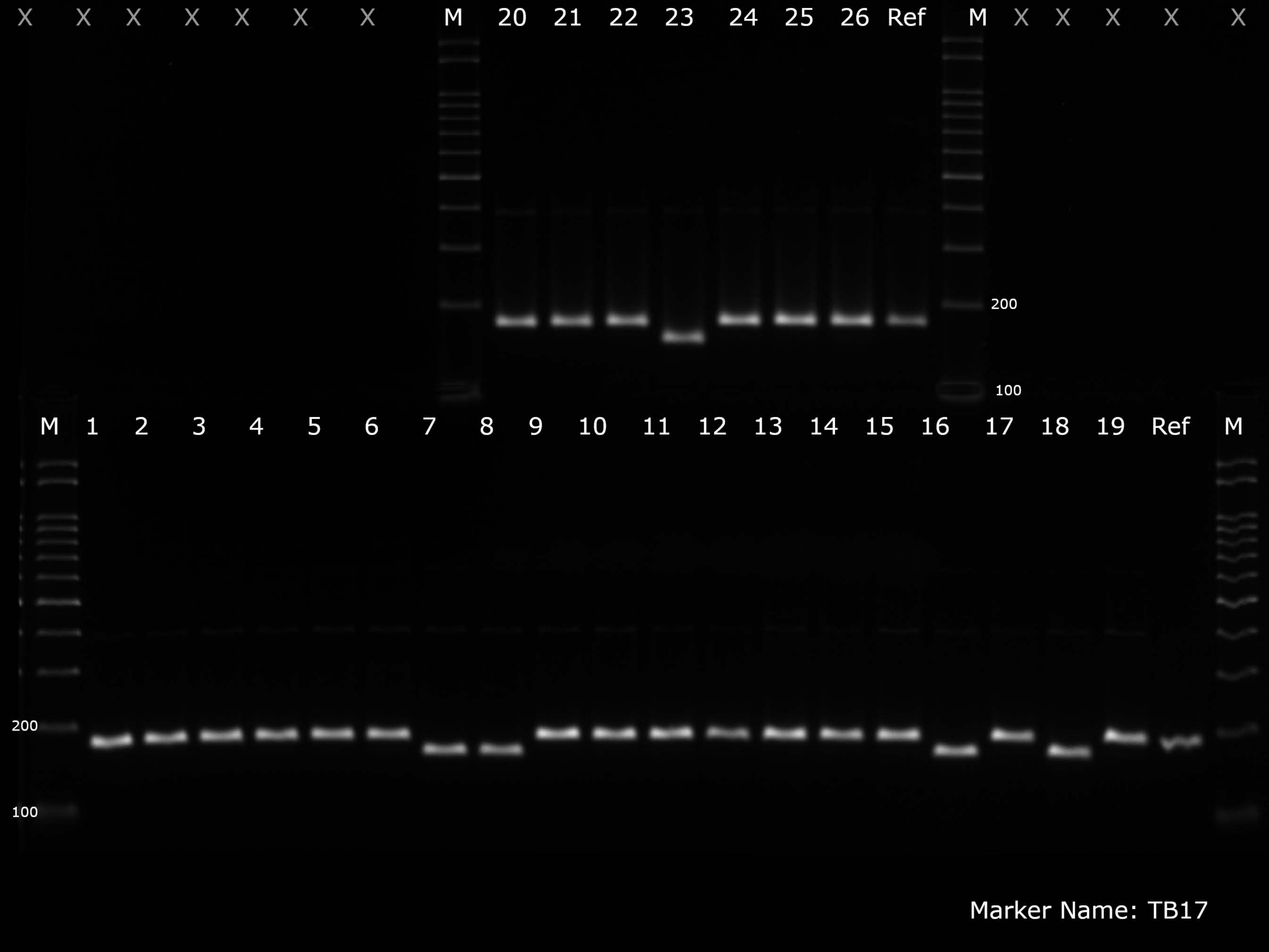
X X X X X X X M 20 21 22 23 24 25 26 Ref M X X X X X
200
100
M 1 2 3 4 5 6 7 8 9 10 11 12 13 14 15 16 17 18 19 Ref M
200
100
Marker Name: TB17

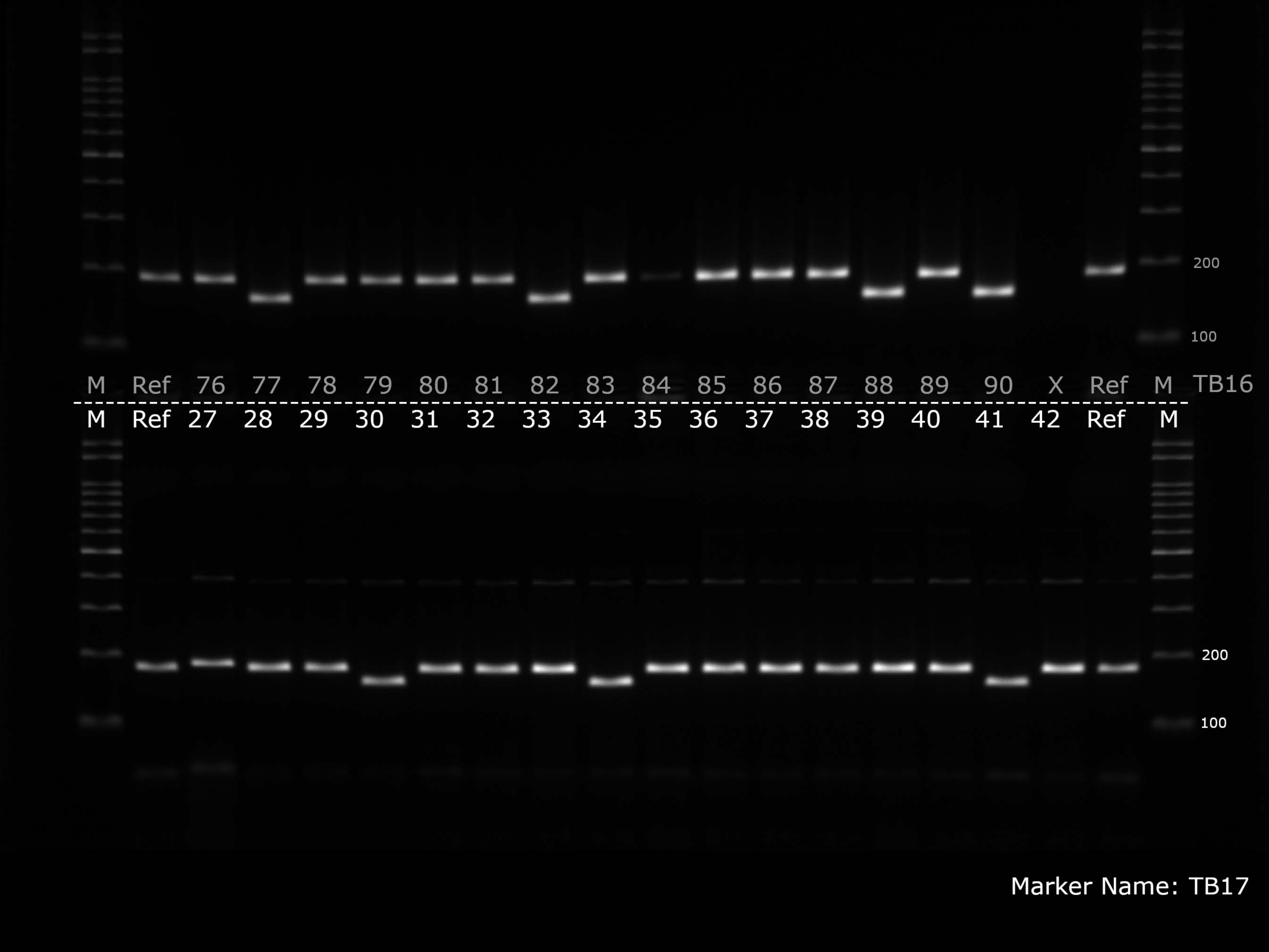
M Ref 76 77 78 79 80 81 82 83 84 85 86 87 88 89 90 X Ref M TB16
M Ref 27 28 29 30 31 32 33 34 35 36 37 38 39 40 41 42 Ref M
200
100
200
100
Marker Name: TB17

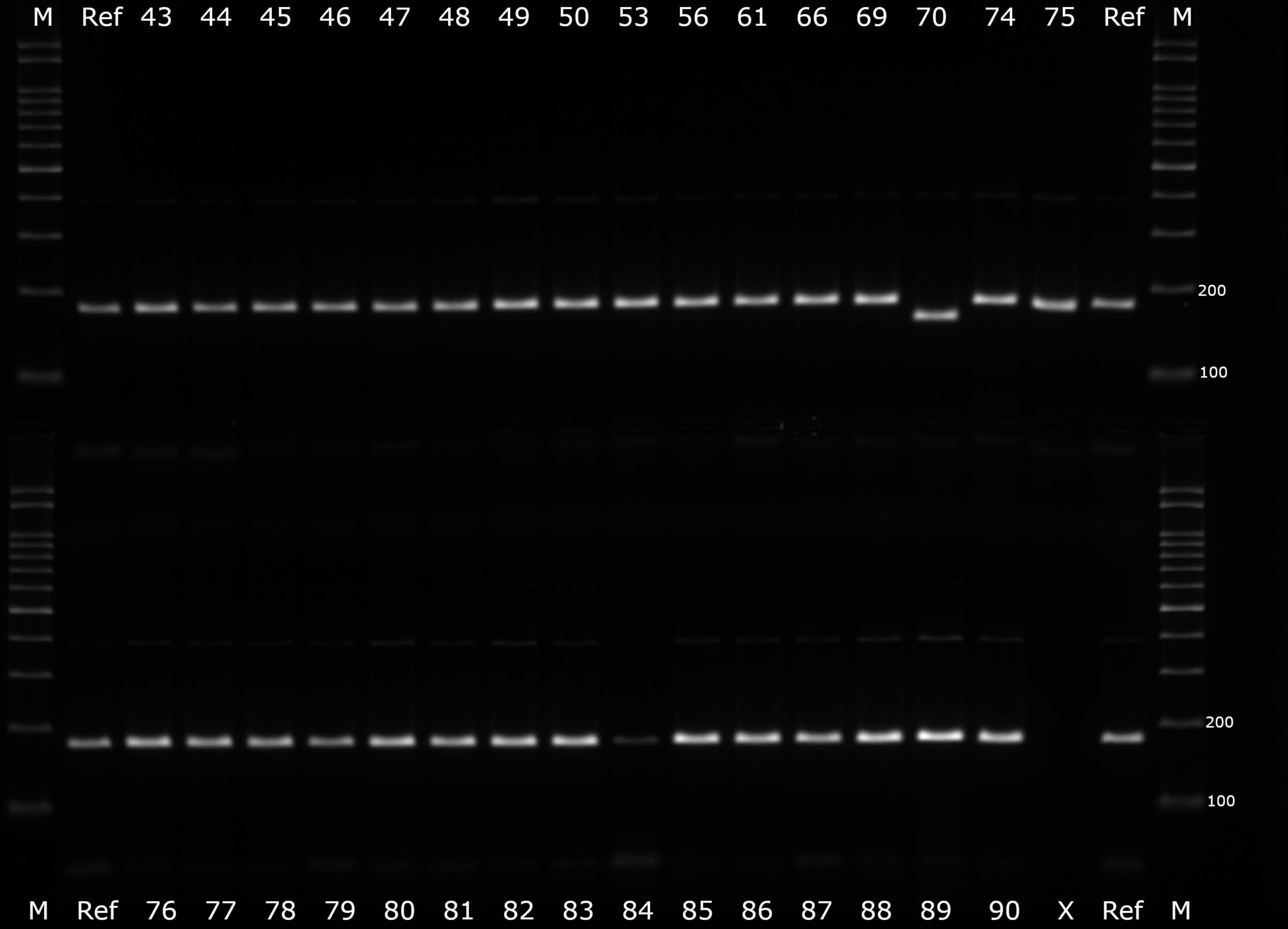

Marker Name: TB17

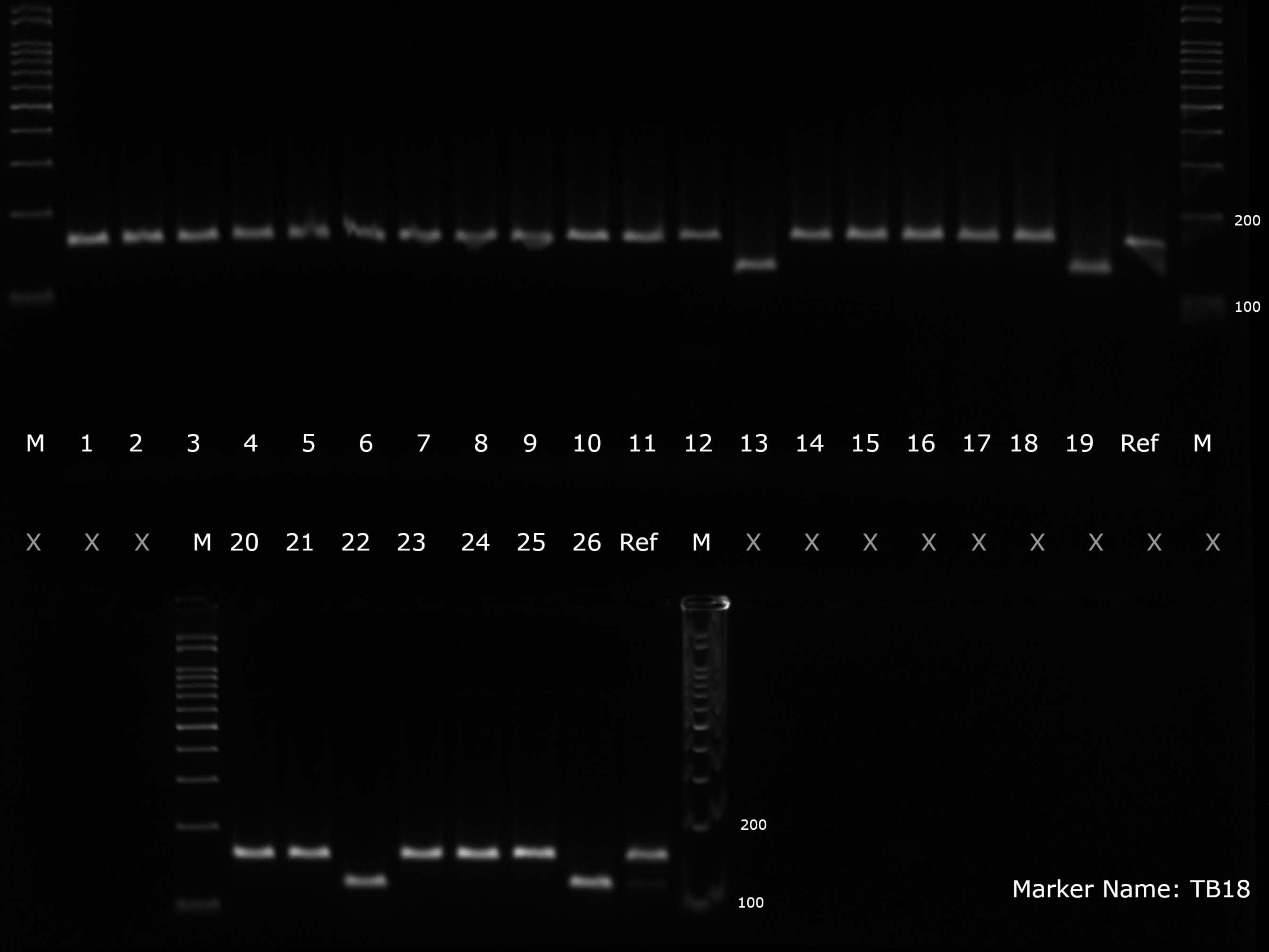
200
100
M 1 2 3 4 5 6 7 8 9 10 11 12 13 14 15 16 17 18 19 Ref M
X X X M 20 21 22 23 24 25 26 Ref M X X X X X X X X X
200
100
Marker Name: TB18

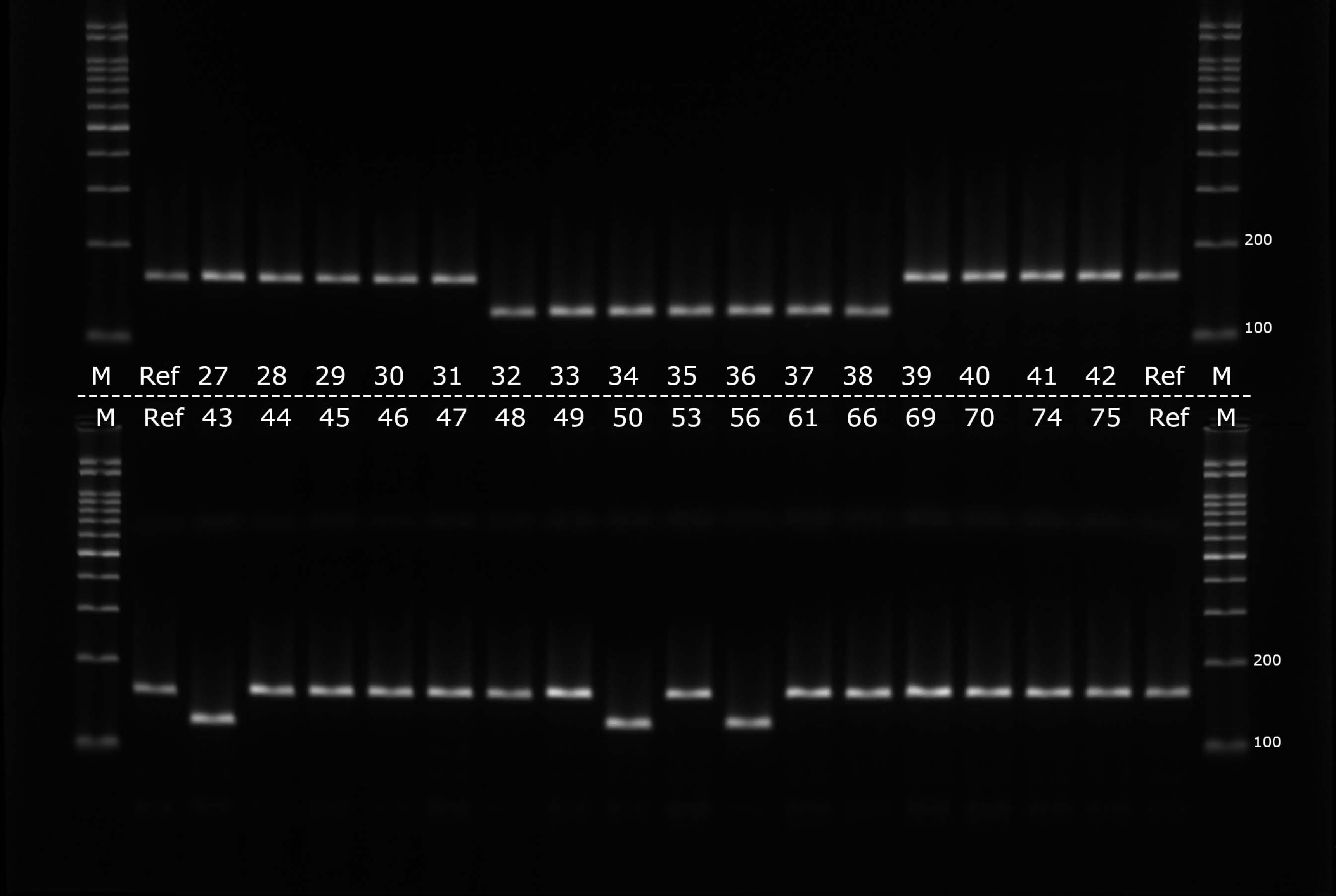

Marker Name: TB18

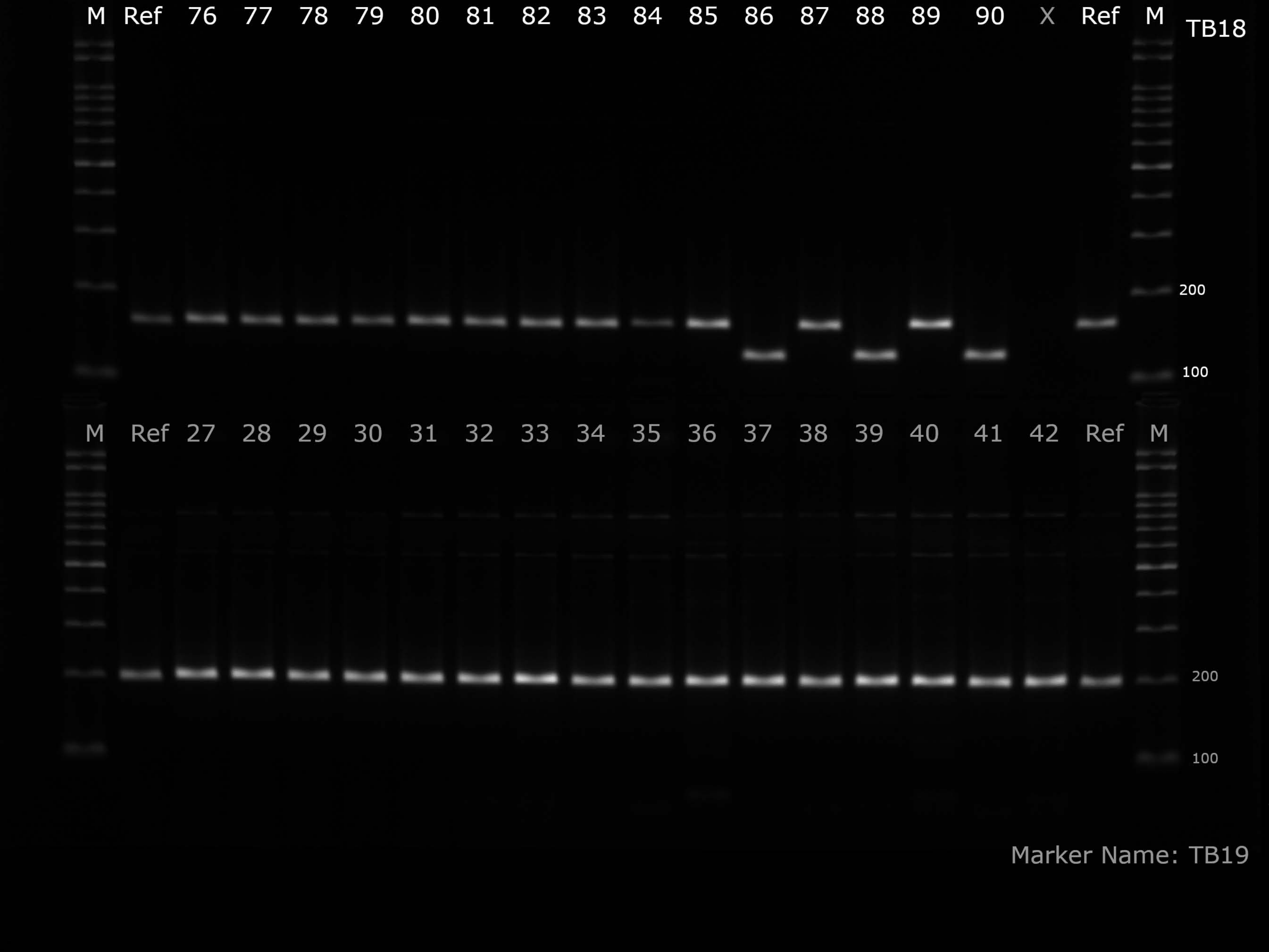

M Ref 76 77 78 79 80 81 82 83 84 85 86 87 88 89 90 X Ref M
TB18
200
100
M Ref 27 28 29 30 31 32 33 34 35 36 37 38 39 40 41 42 Ref M
200
100
Marker Name: TB19

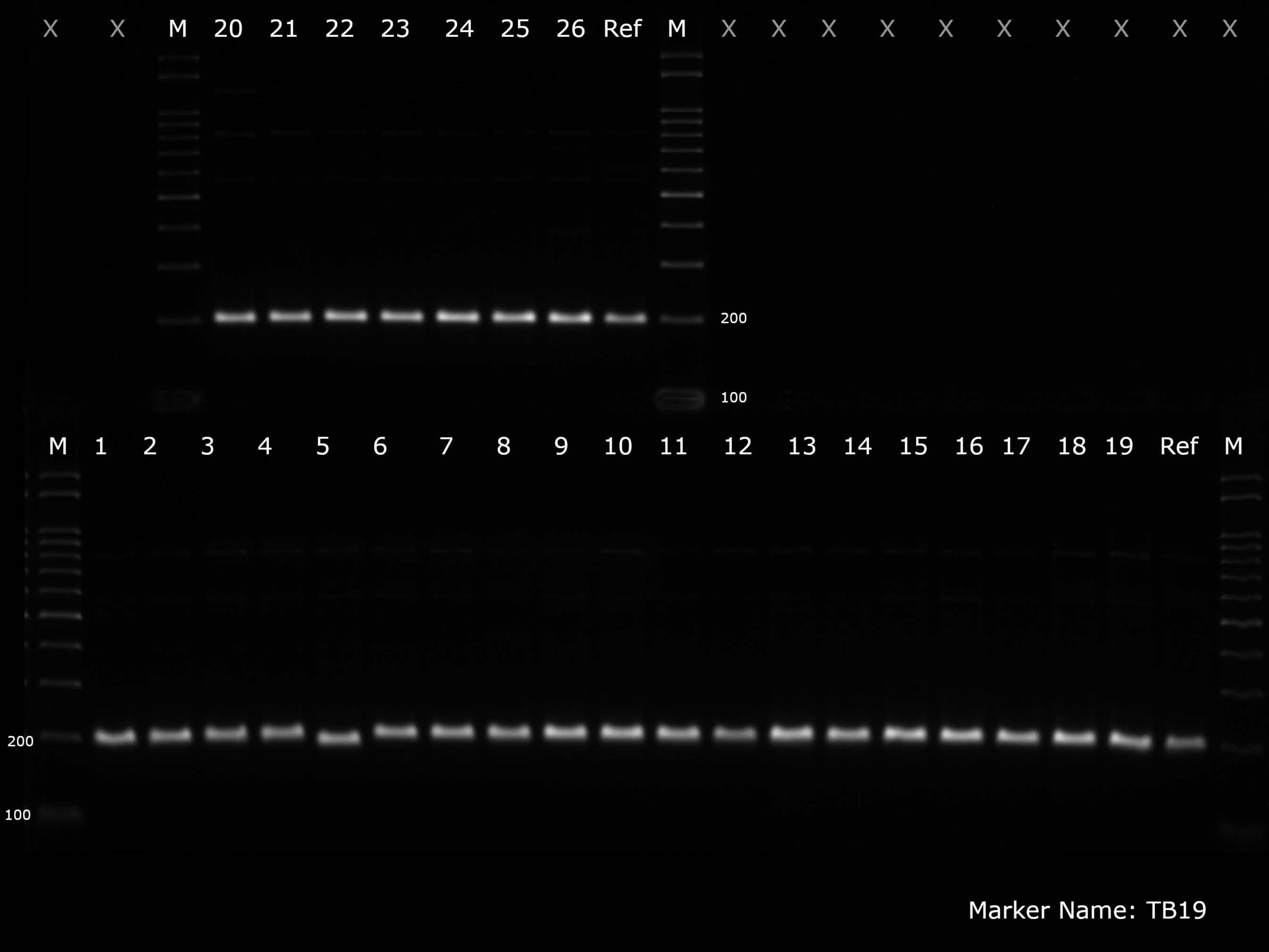
X X M 20 21 22 23 24 25 26 Ref M X X X X X X X X X X
200
100
M 1 2 3 4 5 6 7 8 9 10 11 12 13 14 15 16 17 18 19 Ref M
200
100
Marker Name: TB19

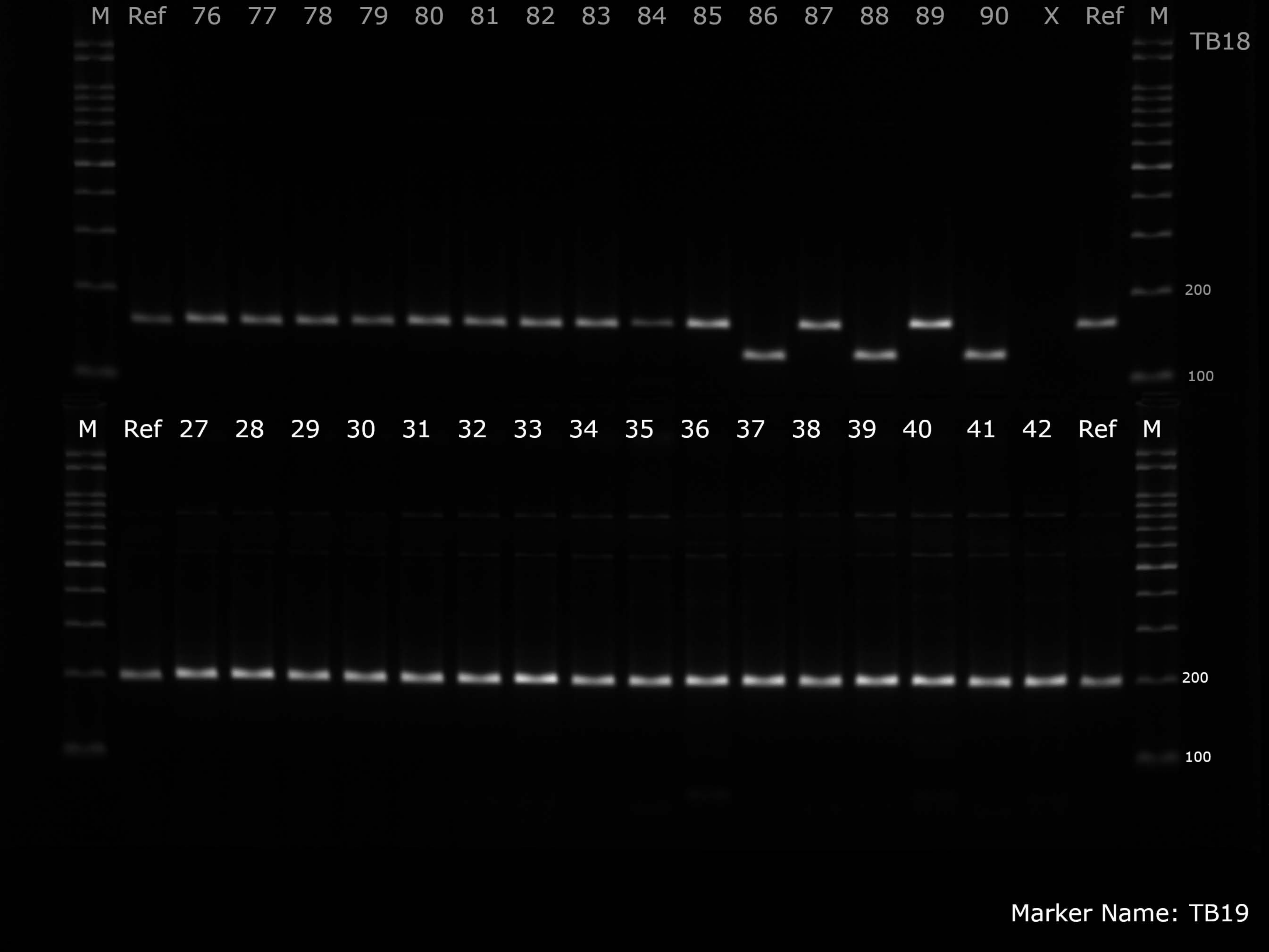
M Ref 76 77 78 79 80 81 82 83 84 85 86 87 88 89 90 X Ref M
TB18
200
100
M Ref 27 28 29 30 31 32 33 34 35 36 37 38 39 40 41 42 Ref M
200
100
Marker Name: TB19

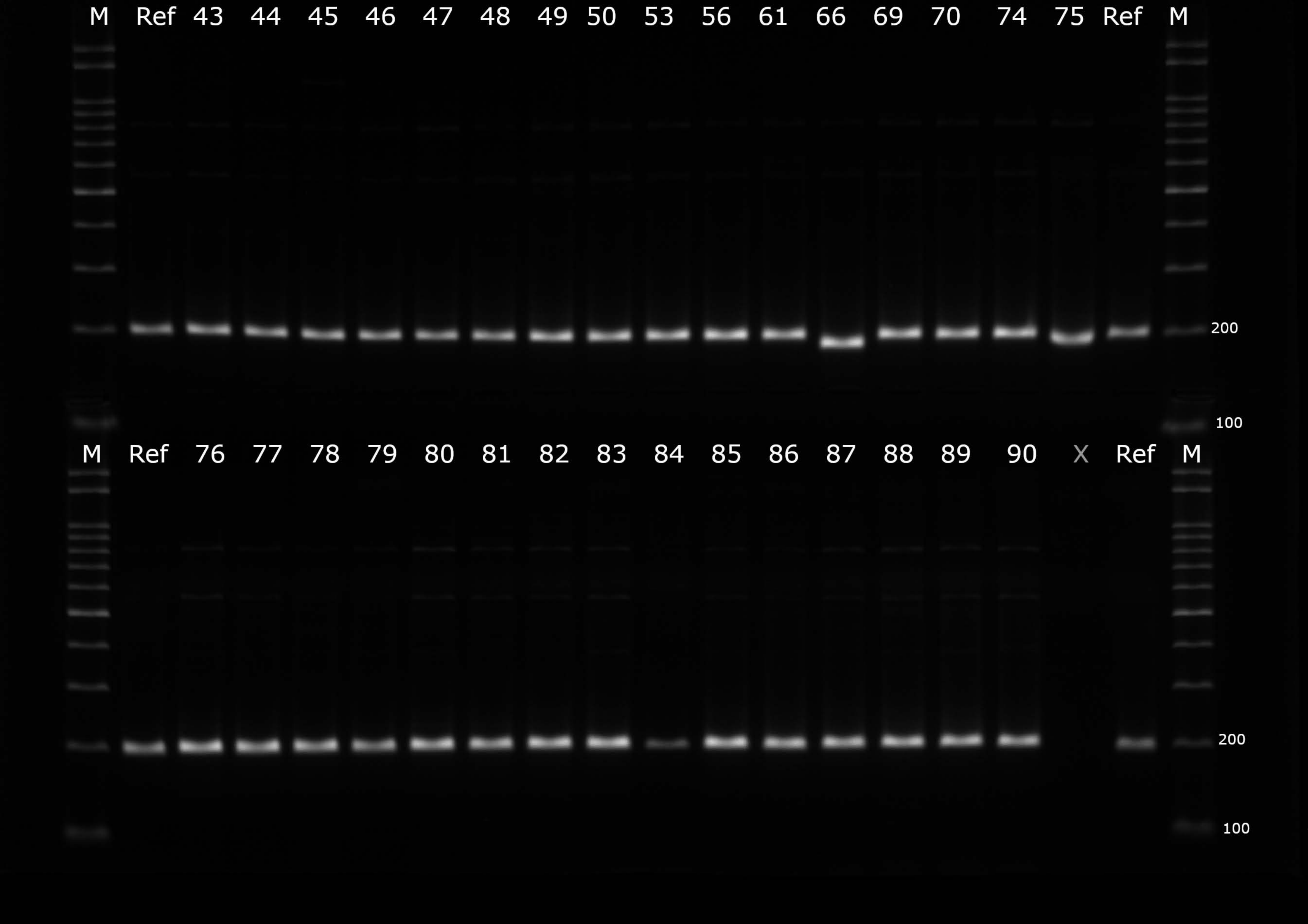

Marker Name: TB19

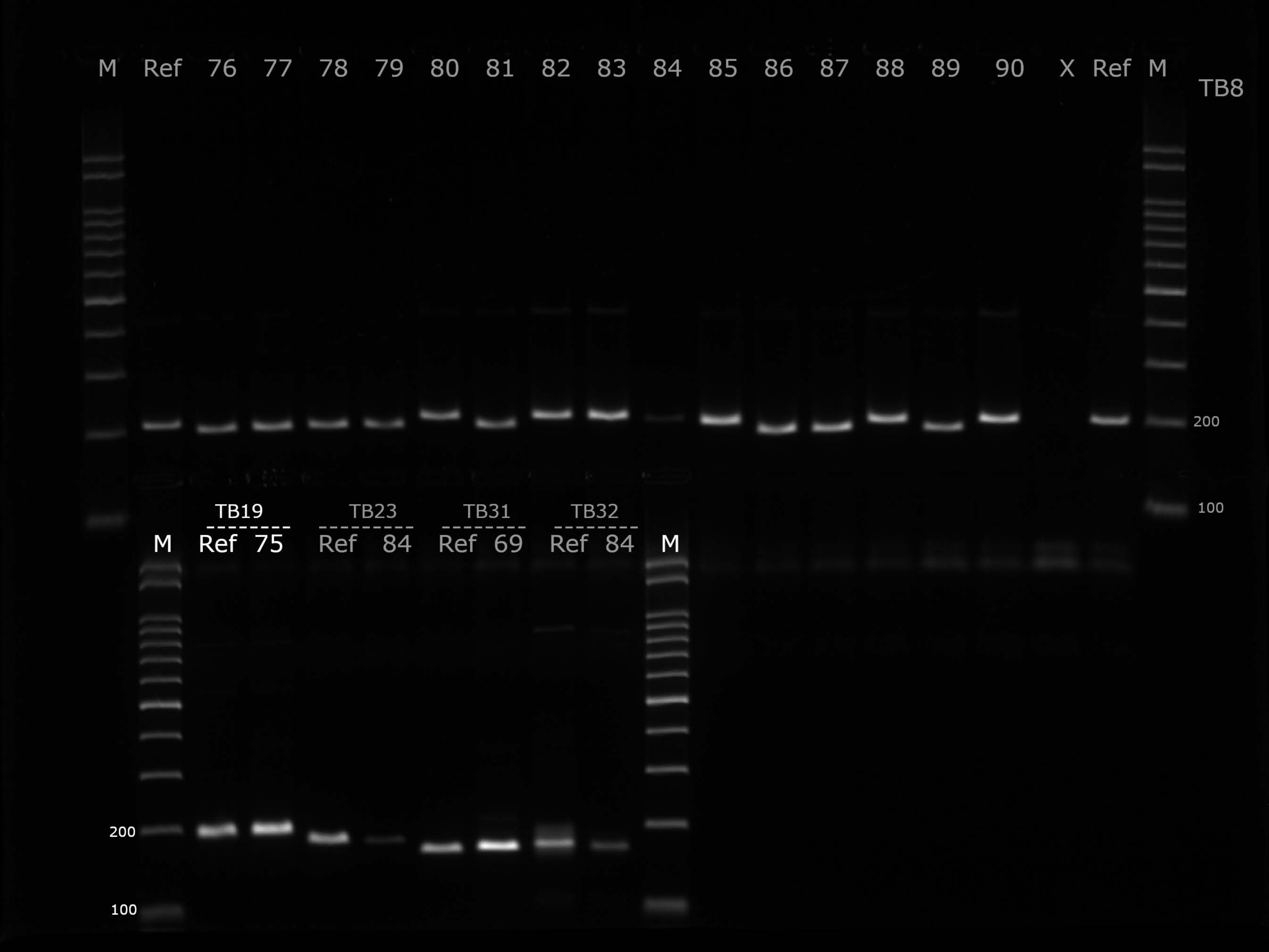
M Ref 76 77 78 79 80 81 82 83 84 85 86 87 88 89 90 X Ref M
TB8
200
100
TB19
TB23
TB31
TB32
M Ref 75 Ref 84 Ref 69 Ref 84 M
200
100

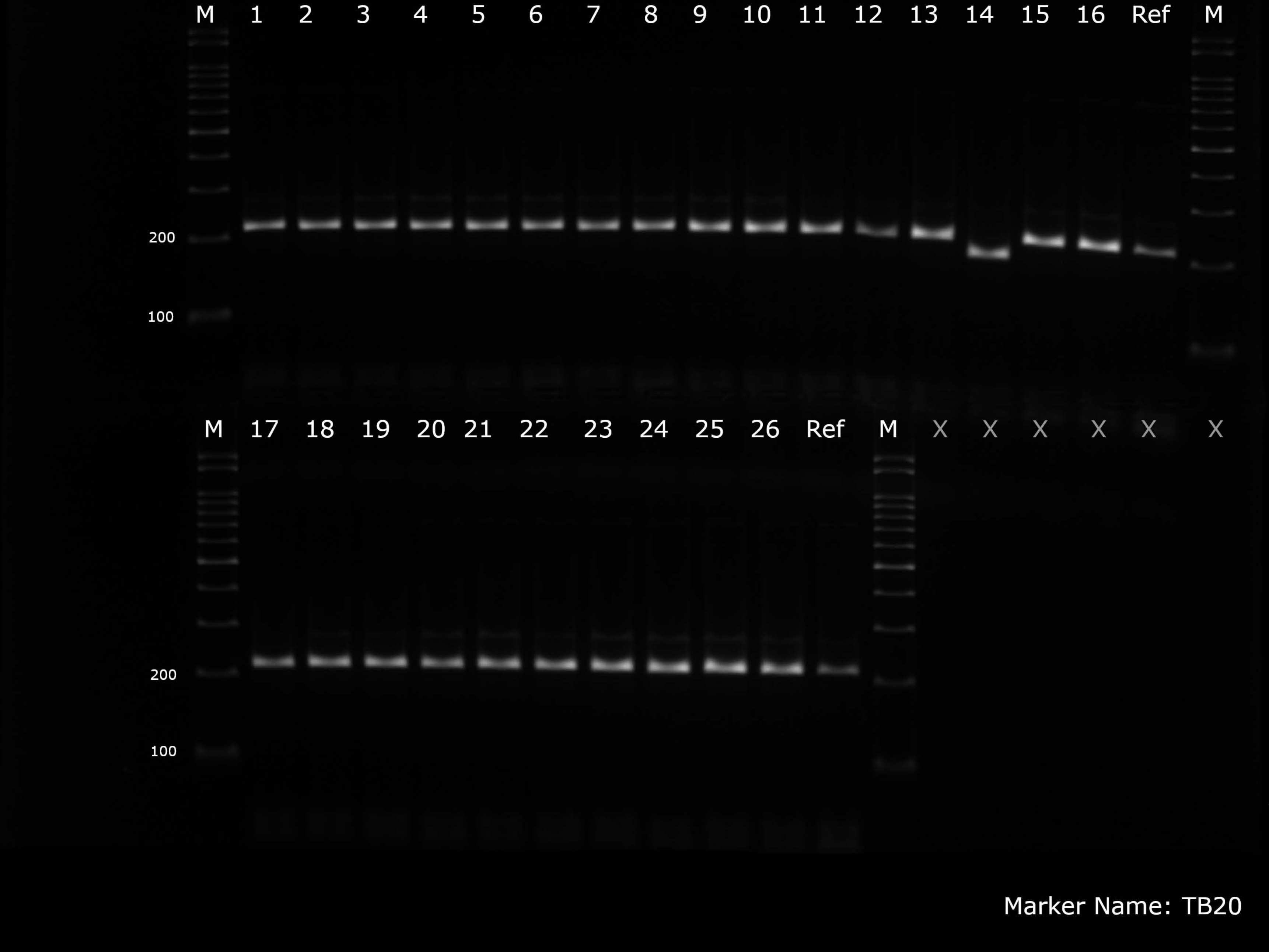
M 1 2 3 4 5 6 7 8 9 10 11 12 13 14 15 16 Ref M
200
100
M 17 18 19 20 21 22 23 24 25 26 Ref M X X X X X X
200
100
Marker Name: TB20

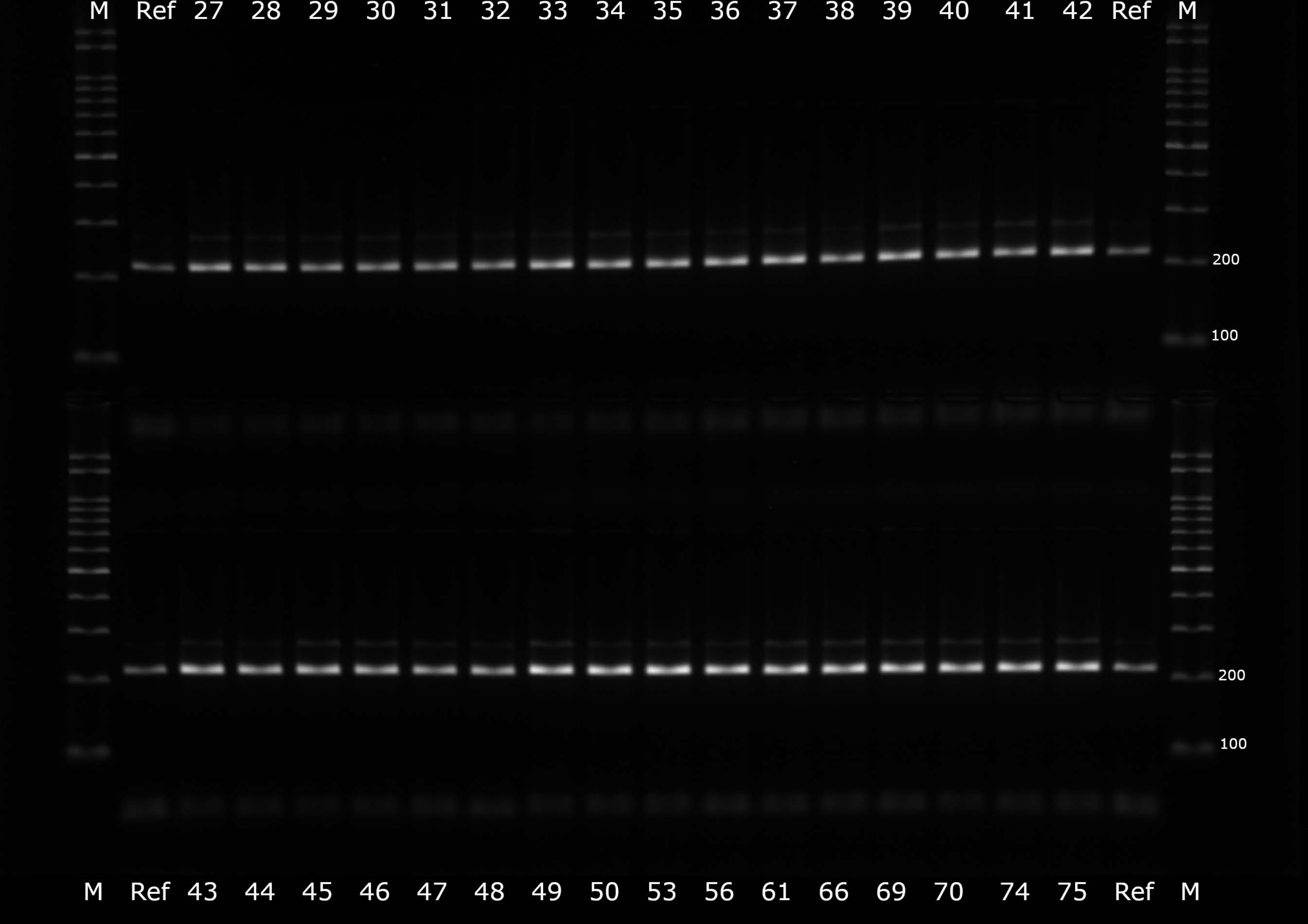

Marker Name: TB20

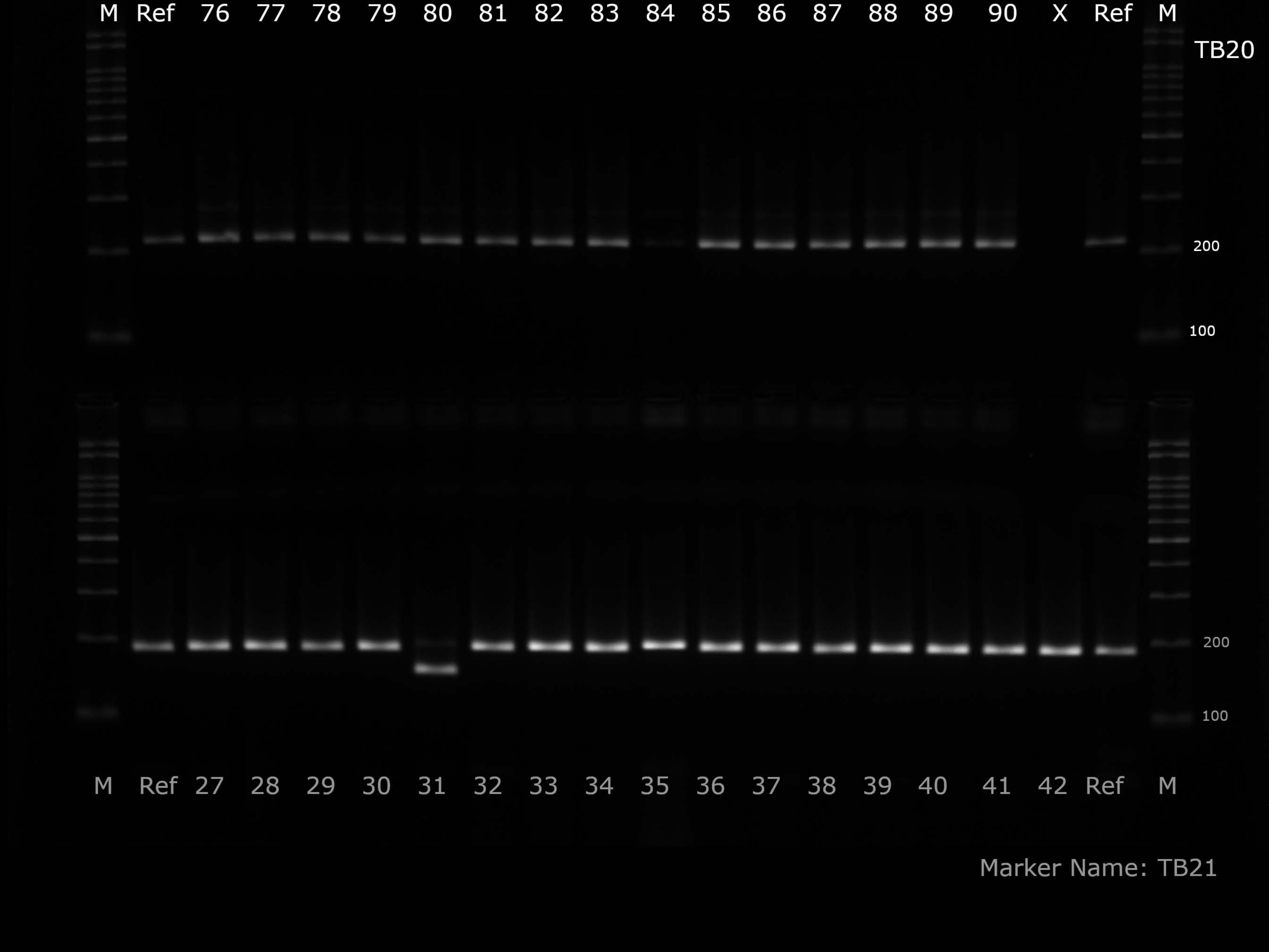

M Ref 76 77 78 79 80 81 82 83 84 85 86 87 88 89 90 X Ref M
TB20
200
100
200
100
M Ref 27 28 29 30 31 32 33 34 35 36 37 38 39 40 41 42 Ref M
Marker Name: TB21

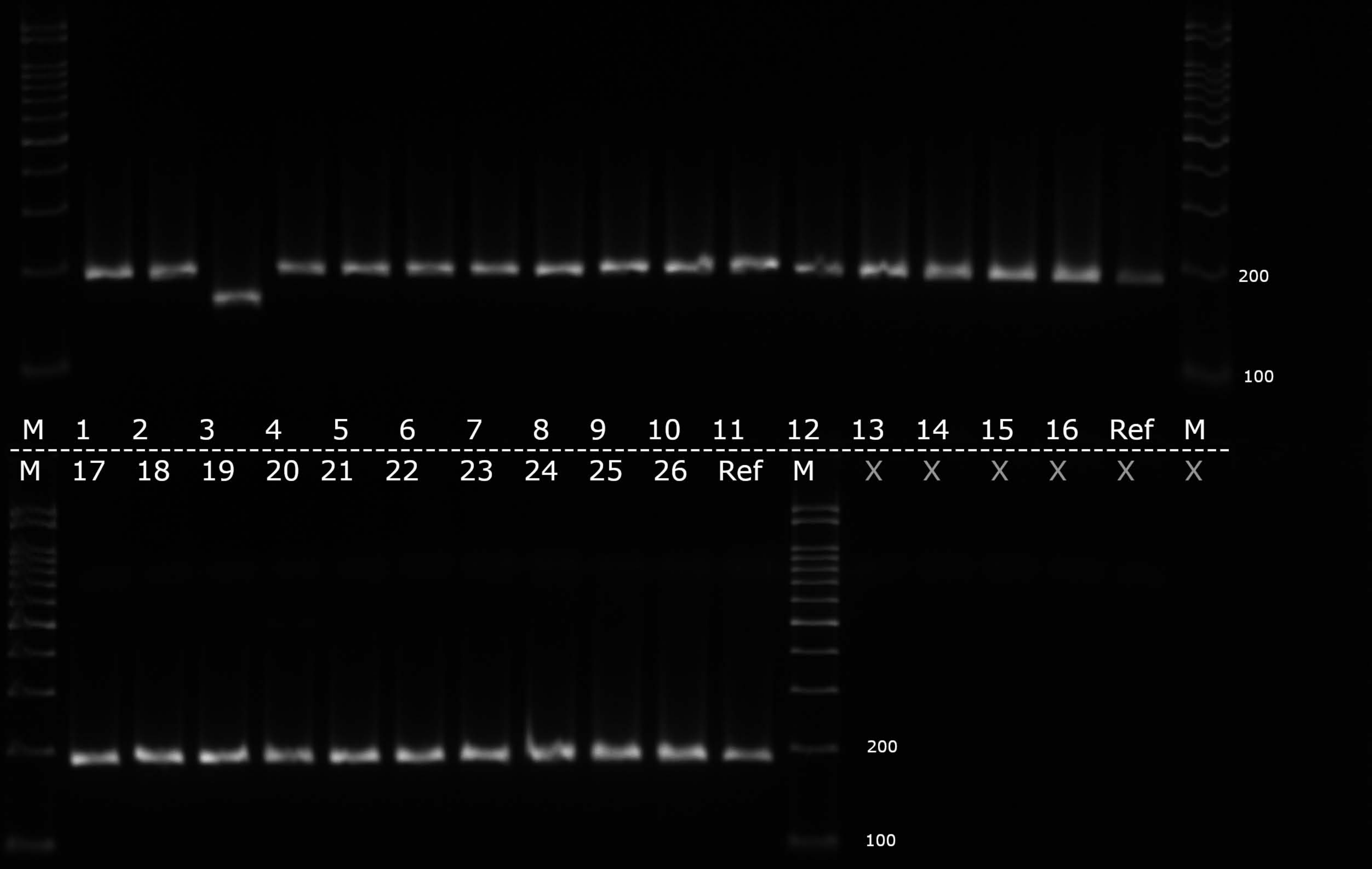

Marker Name: TB21

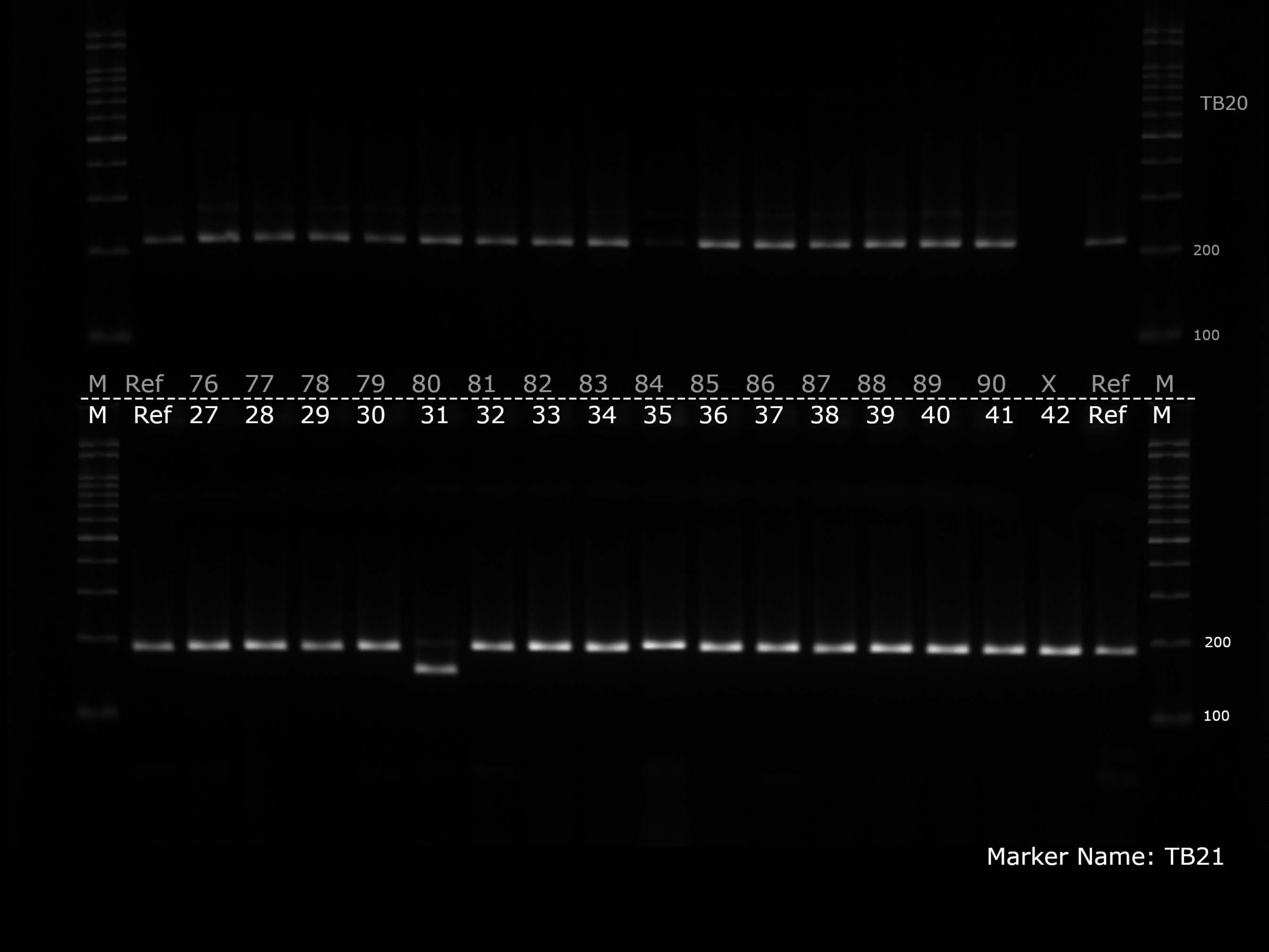
TB20
200
100
M Ref 76 77 78 79 80 81 82 83 84 85 86 87 88 89 90 X Ref M
M Ref 27 28 29 30 31 32 33 34 35 36 37 38 39 40 41 42 Ref M
200
100
Marker Name: TB21

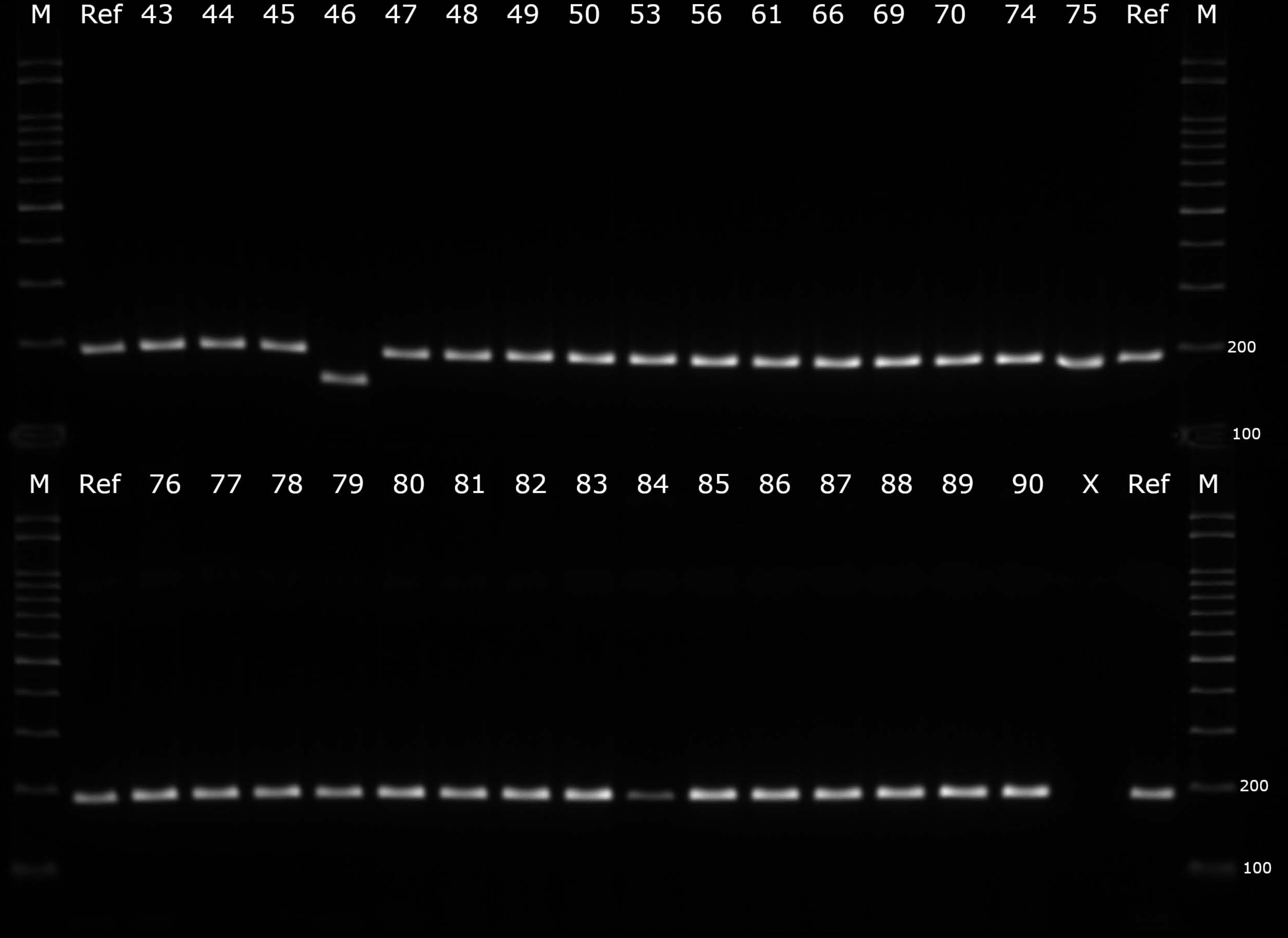

Marker Name: TB21

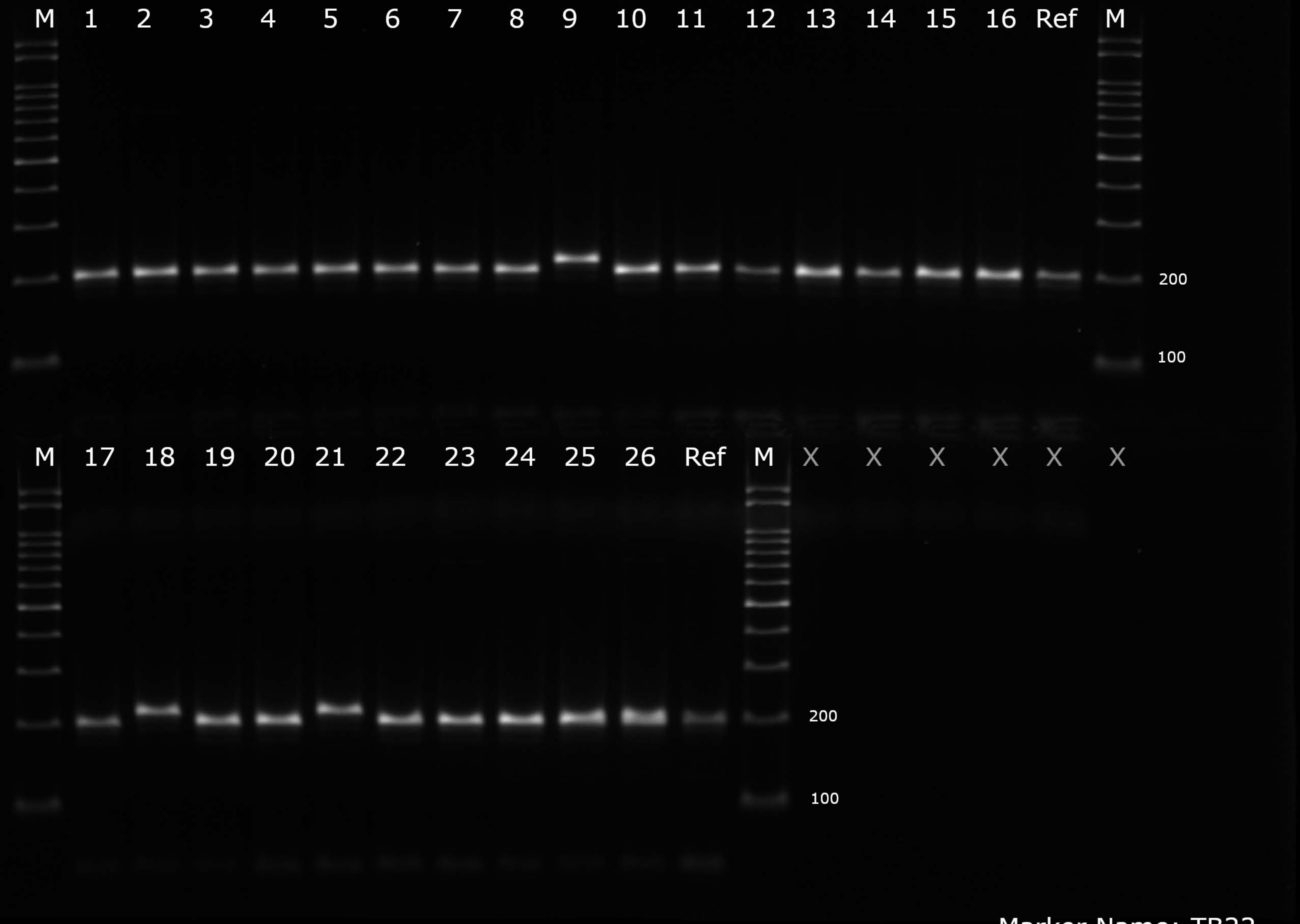

Marker Name: TB22

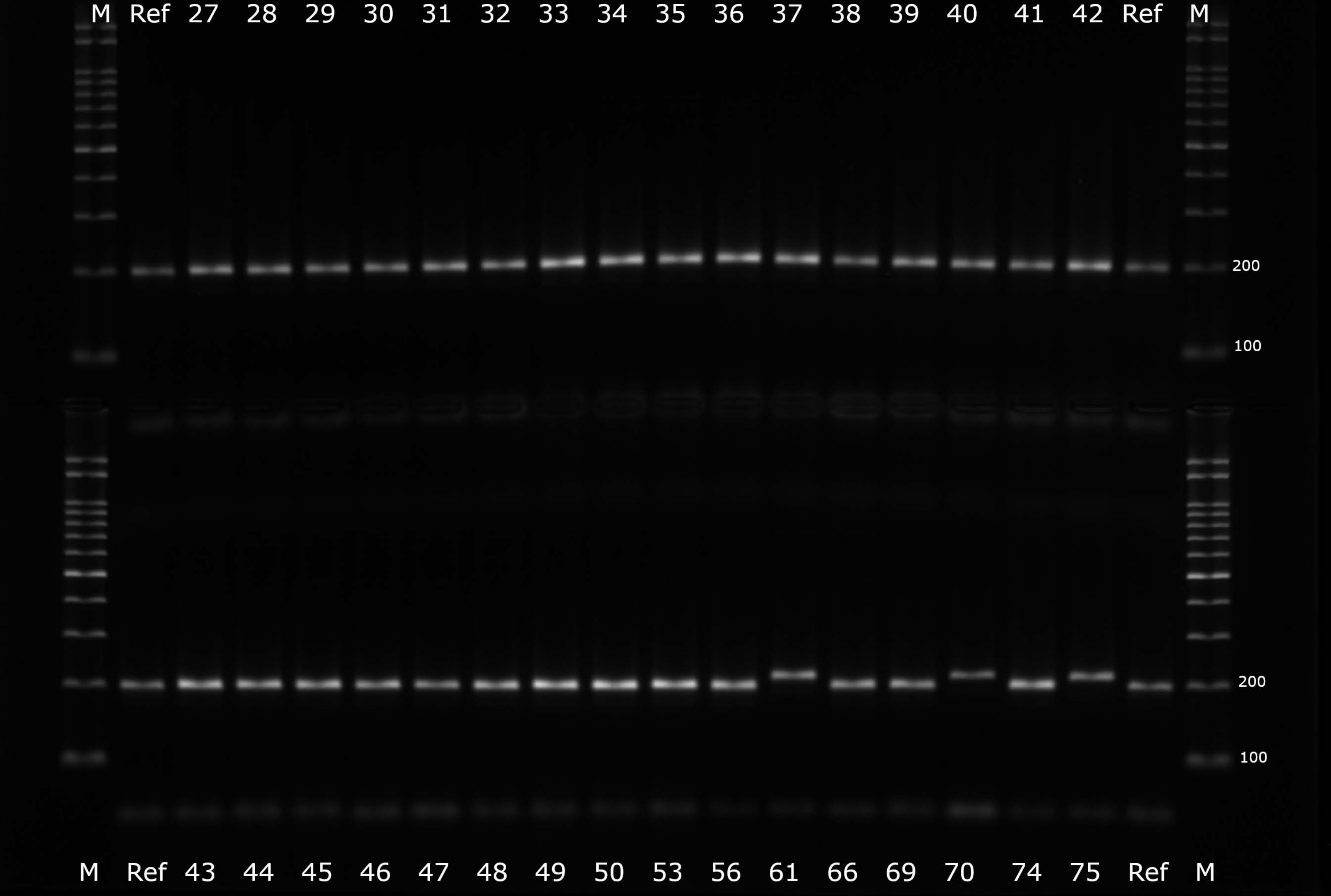

Marker Name: TB22

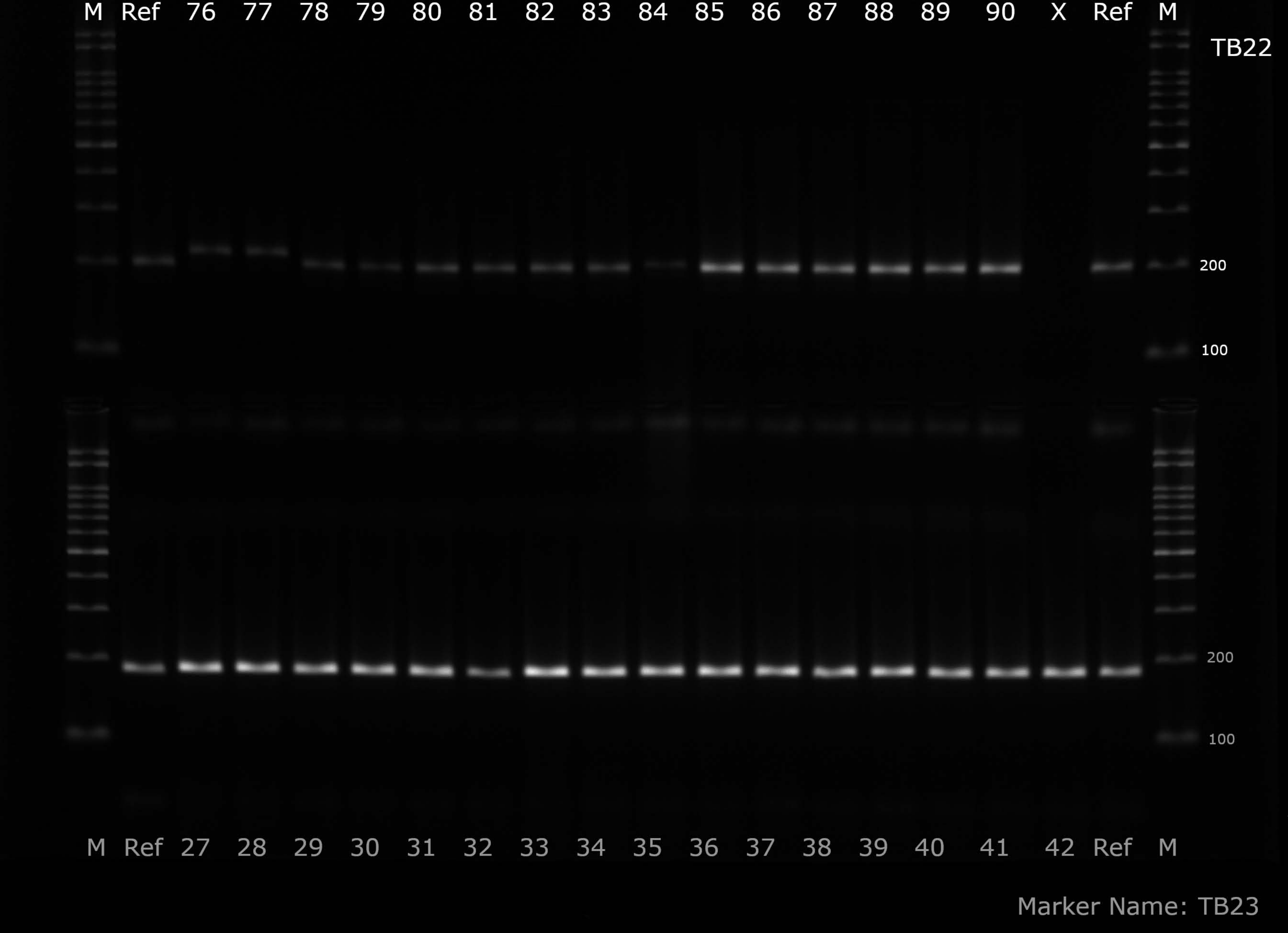
M Ref 76 77 78 79 80 81 82 83 84 85 86 87 88 89 90 X Ref M
TB22
200
100
200
100
M Ref 27 28 29 30 31 32 33 34 35 36 37 38 39 40 41 42 Ref M
Marker Name: TB23

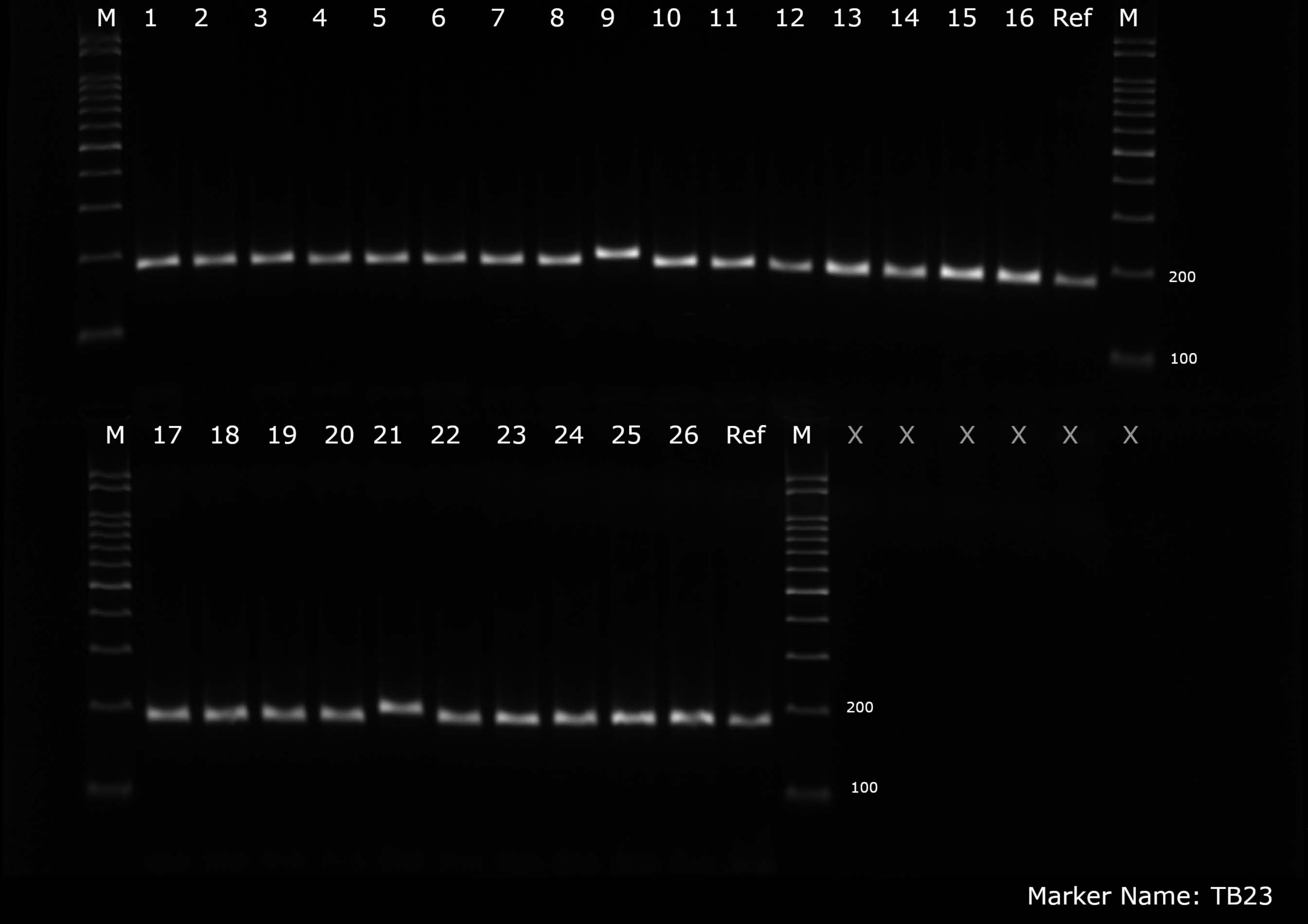
M 1 2 3 4 5 6 7 8 9 10 11 12 13 14 15 16 Ref M
200
100
M 17 18 19 20 21 22 23 24 25 26 Ref M X X X X X X
200
100
Marker Name: TB23

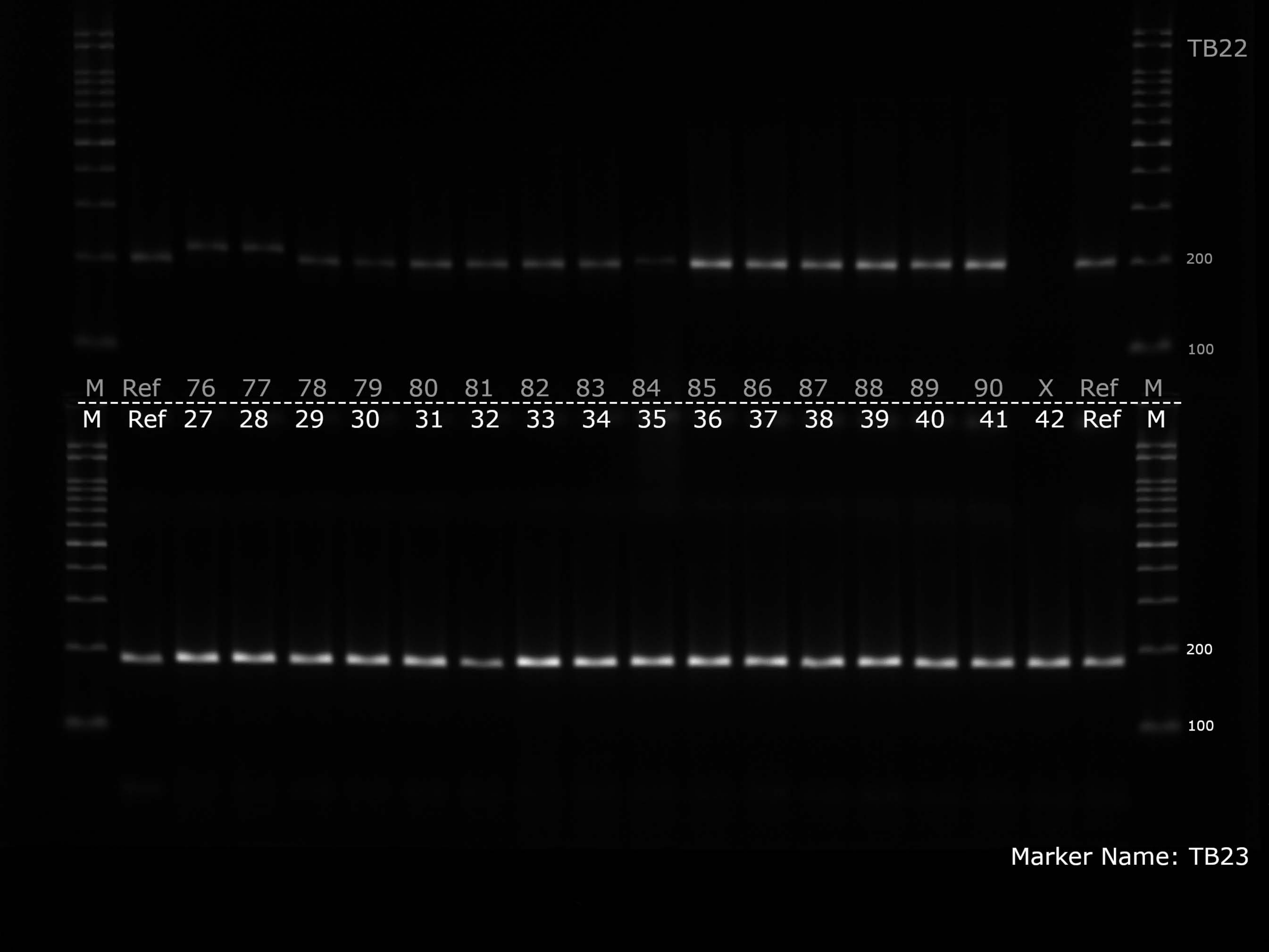
TB22
200
100
M Ref 76 77 78 79 80 81 82 83 84 85 86 87 88 89 90 X Ref M
M Ref 27 28 29 30 31 32 33 34 35 36 37 38 39 40 41 42 Ref M
200
100
Marker Name: TB23

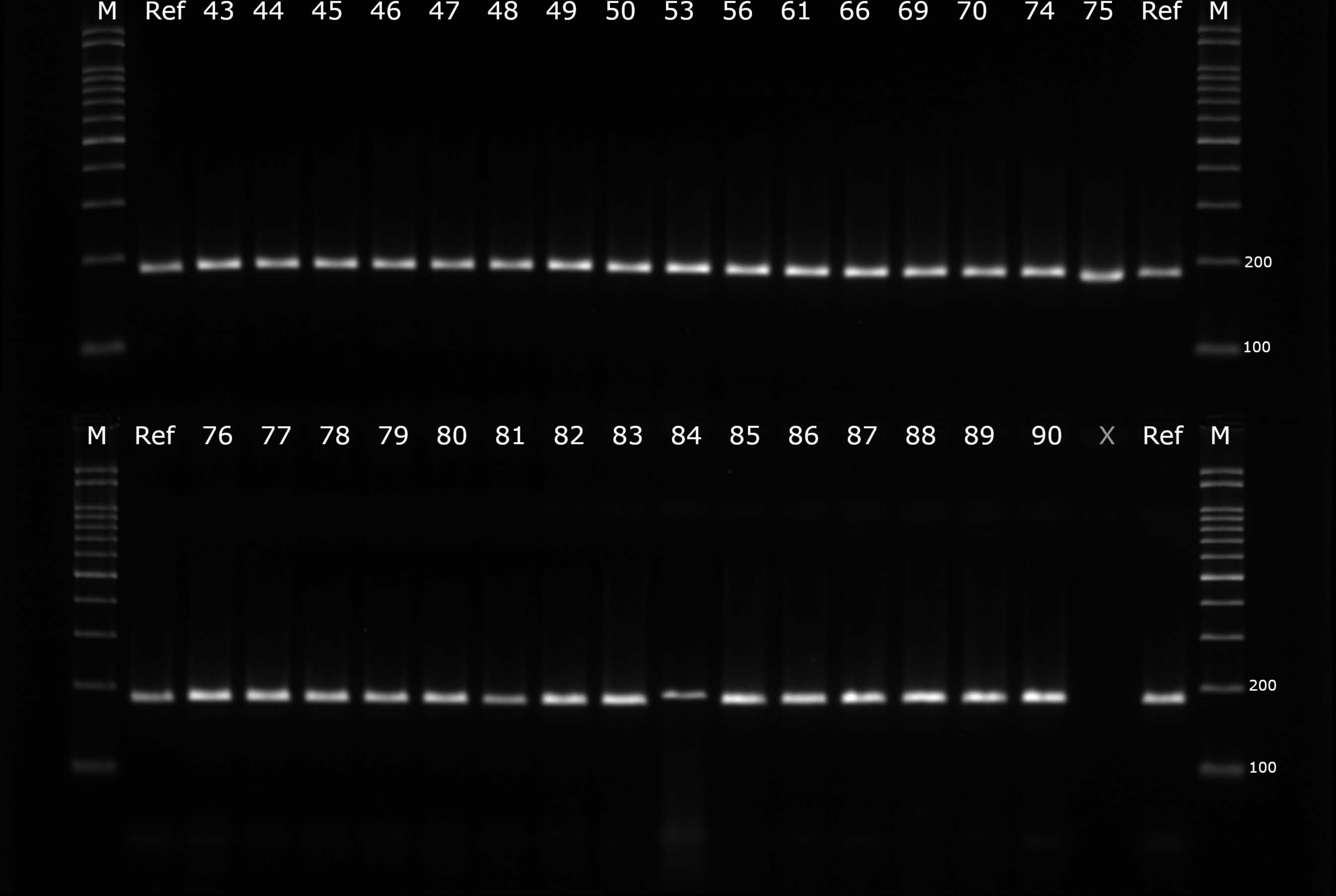

Marker Name: TB23

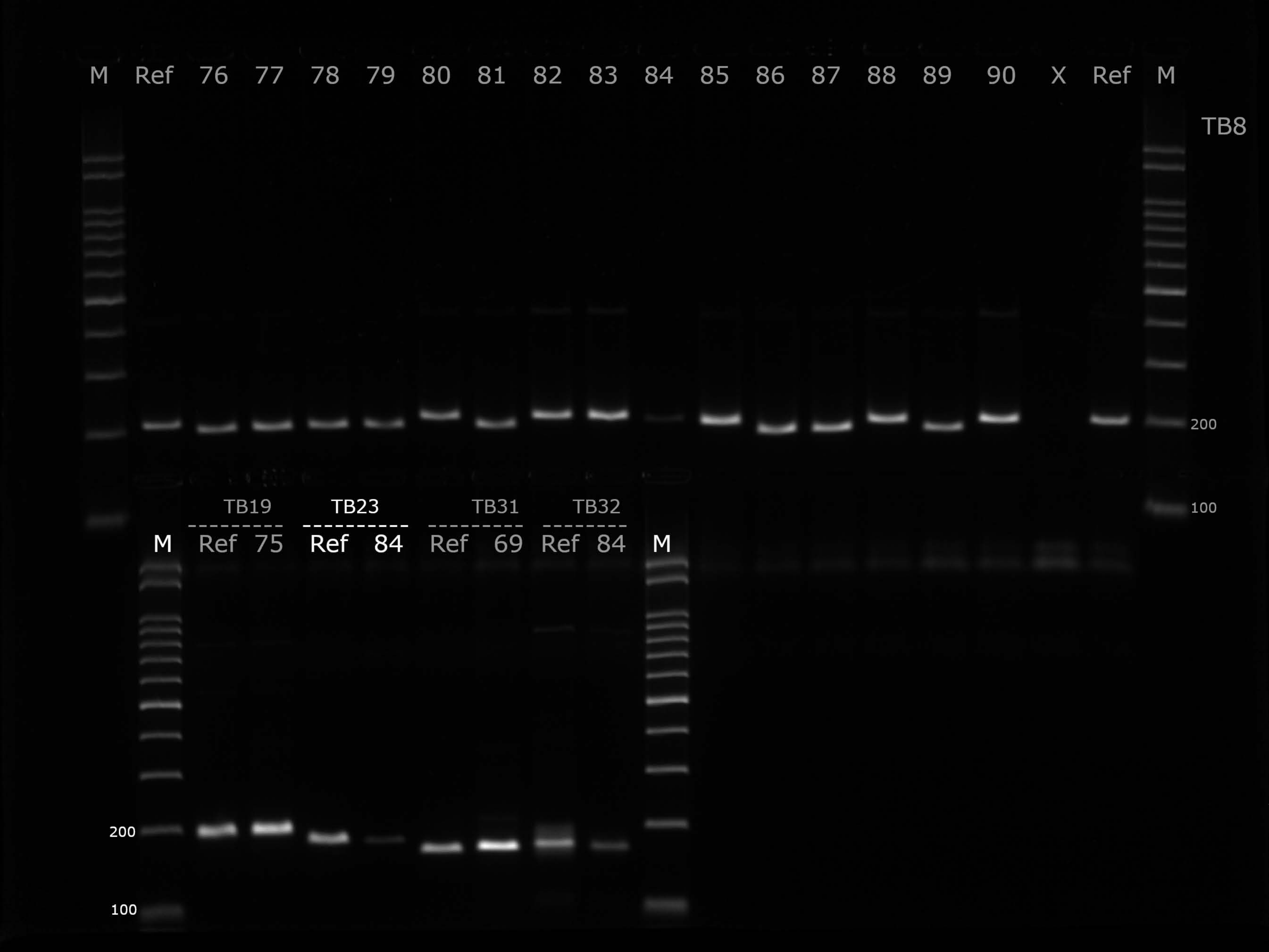
M Ref 76 77 78 79 80 81 82 83 84 85 86 87 88 89 90 X Ref M
TB8
200
100
TB19
TB23
TB31
TB32
M Ref 75 Ref 84 Ref 69 Ref 84 M
200
100

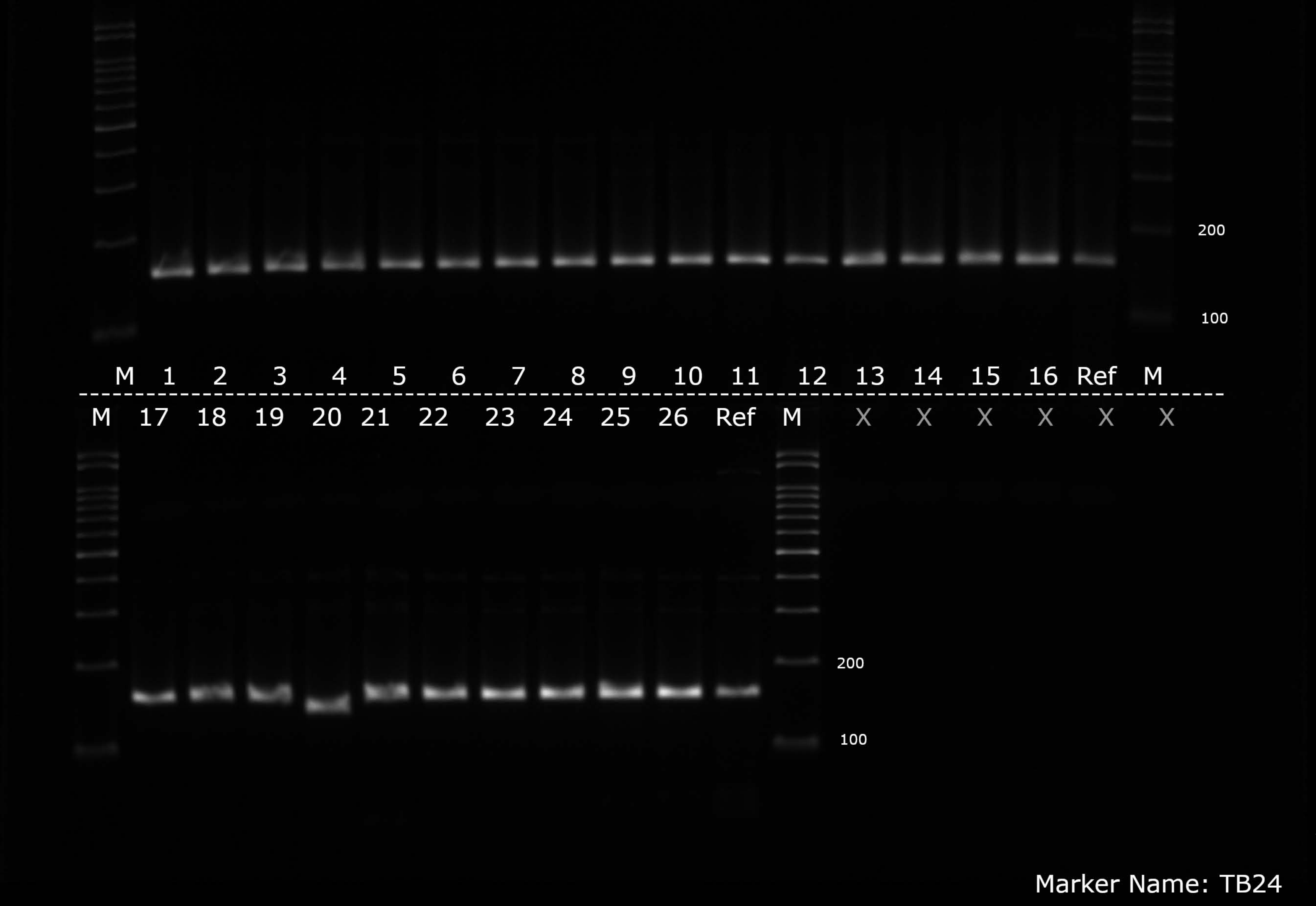
200
100
M 1 2 3 4 5 6 7 8 9 10 11 12 13 14 15 16 Ref M
M 17 18 19 20 21 22 23 24 25 26 Ref M X X X X X X
200
100
Marker Name: TB24

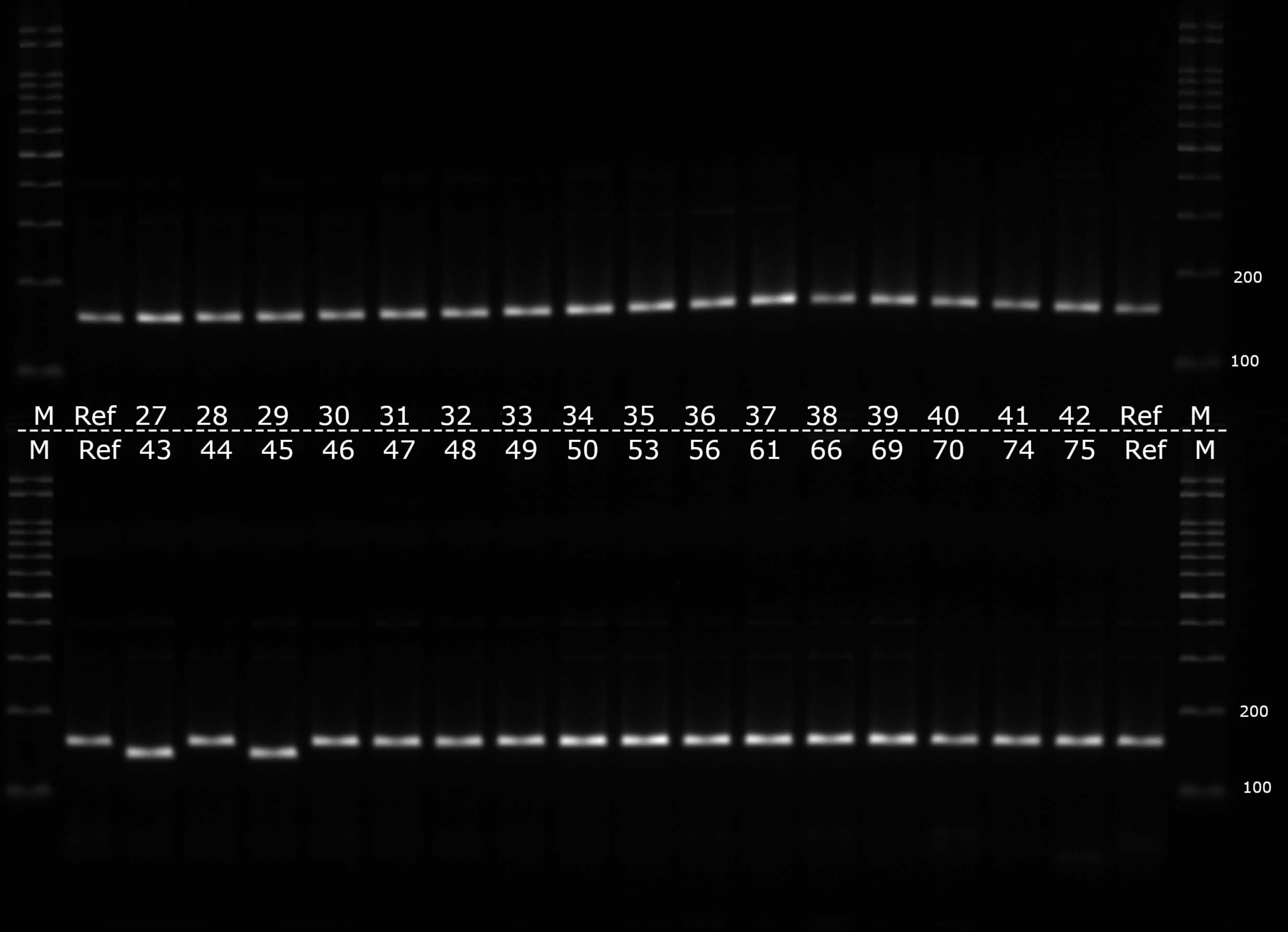

Marker Name: TB24

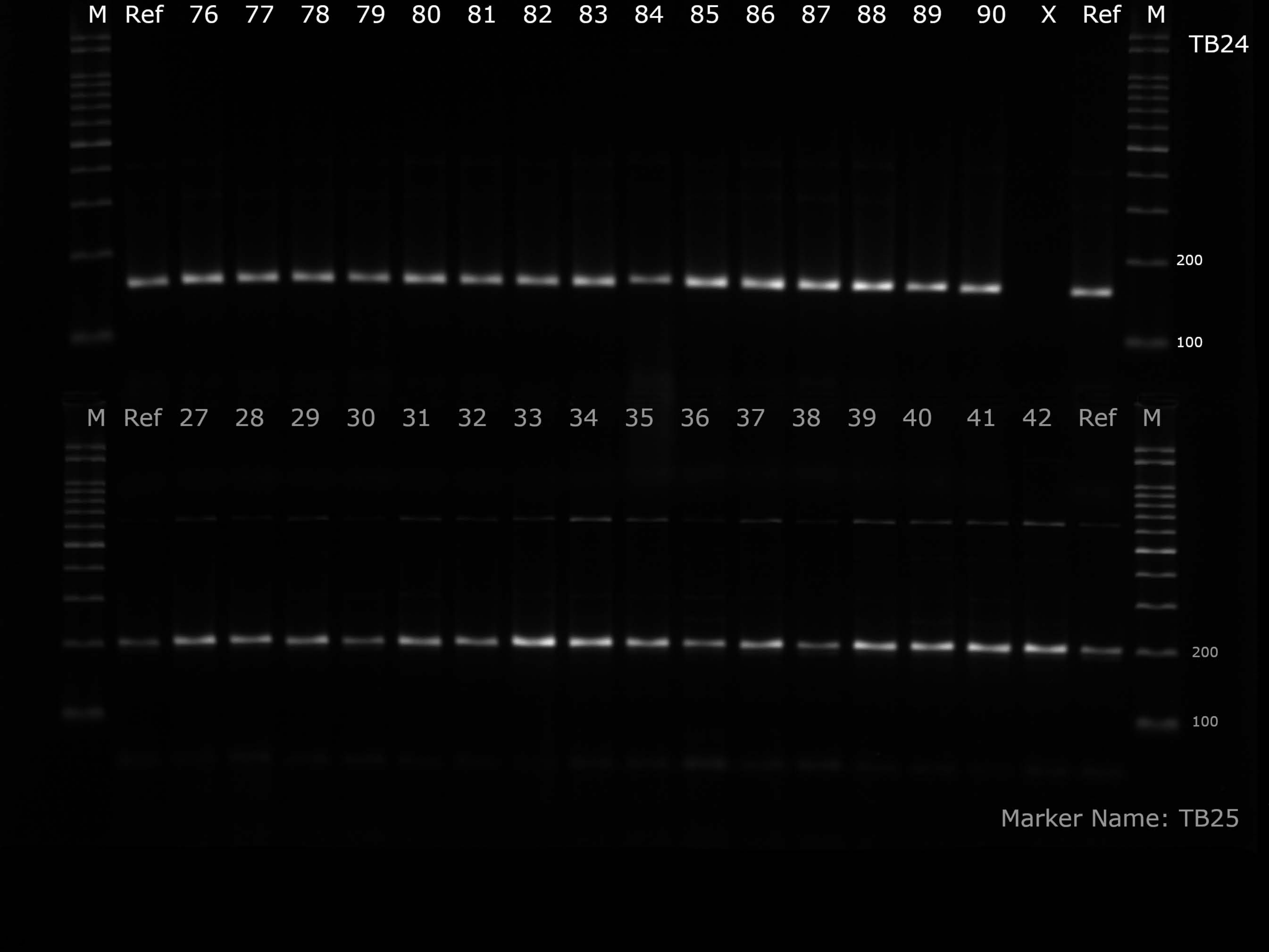
M Ref 76 77 78 79 80 81 82 83 84 85 86 87 88 89 90 X Ref M
TB24
200
100
M Ref 27 28 29 30 31 32 33 34 35 36 37 38 39 40 41 42 Ref M
200
100
Marker Name: TB25

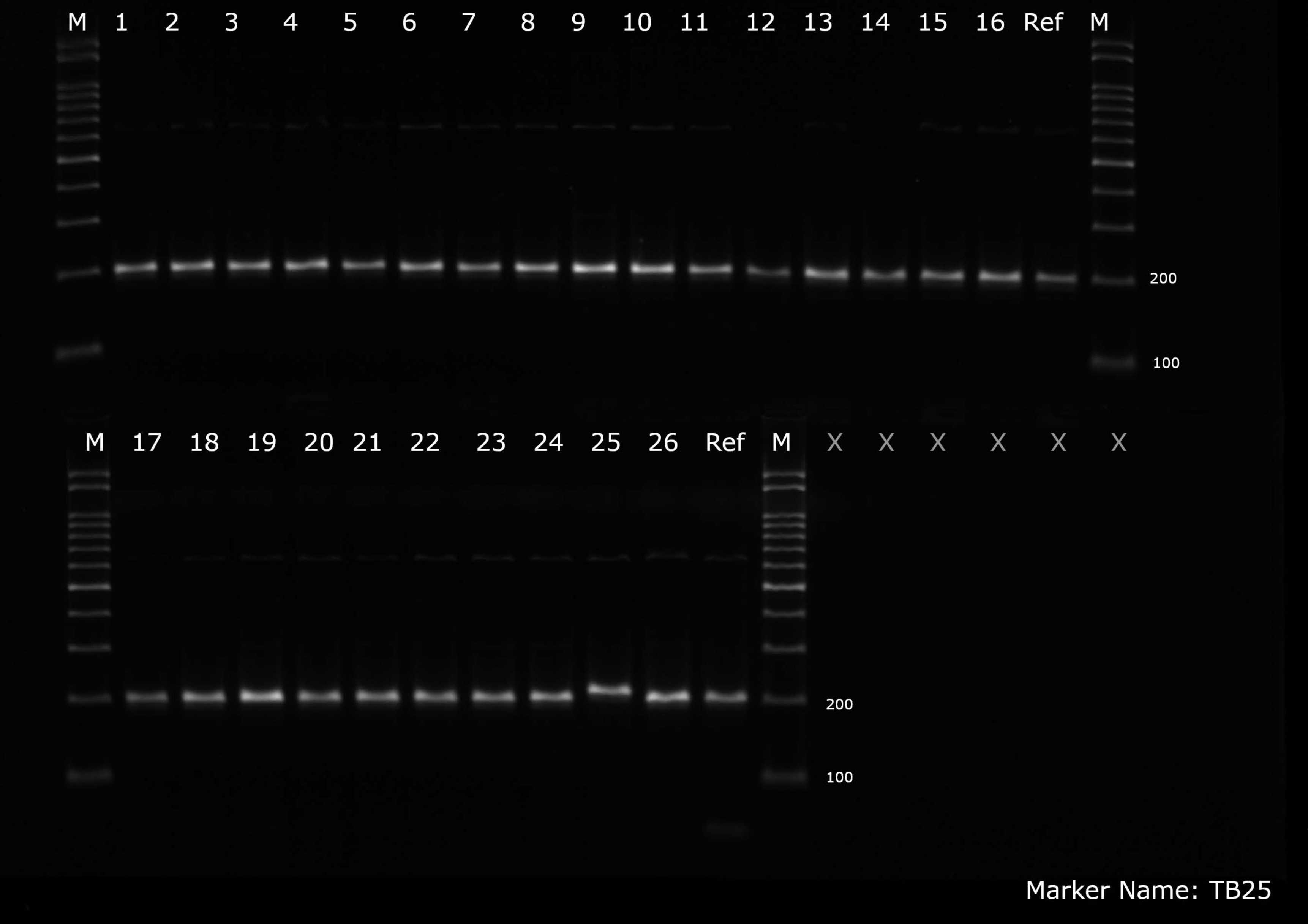
M 1 2 3 4 5 6 7 8 9 10 11 12 13 14 15 16 Ref M
200
100
M 17 18 19 20 21 22 23 24 25 26 Ref M X X X X X X
200
100
Marker Name: TB25

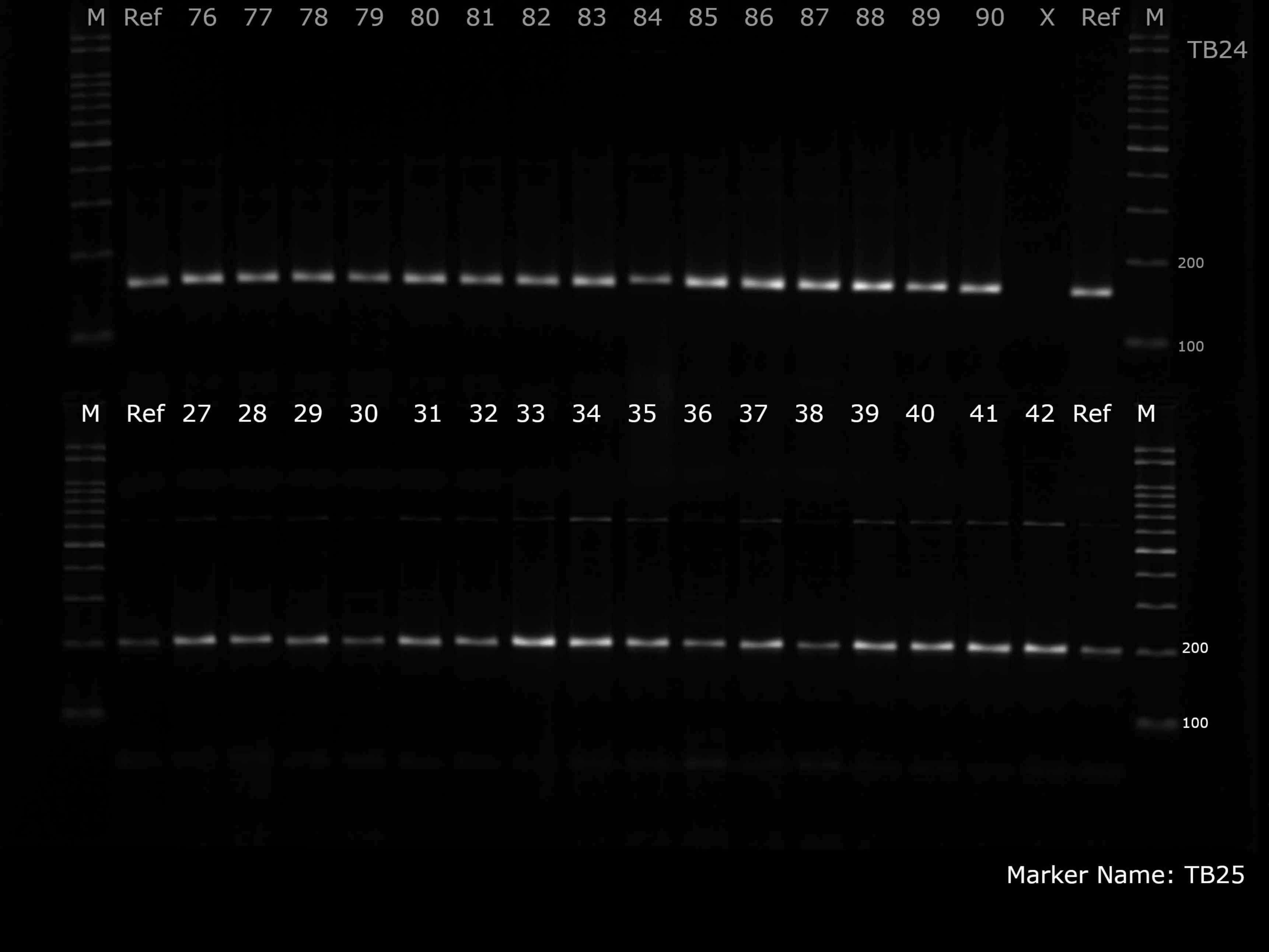
M Ref 76 77 78 79 80 81 82 83 84 85 86 87 88 89 90 X Ref M
TB24
200
100
M Ref 27 28 29 30 31 32 33 34 35 36 37 38 39 40 41 42 Ref M
200
100
Marker Name: TB25

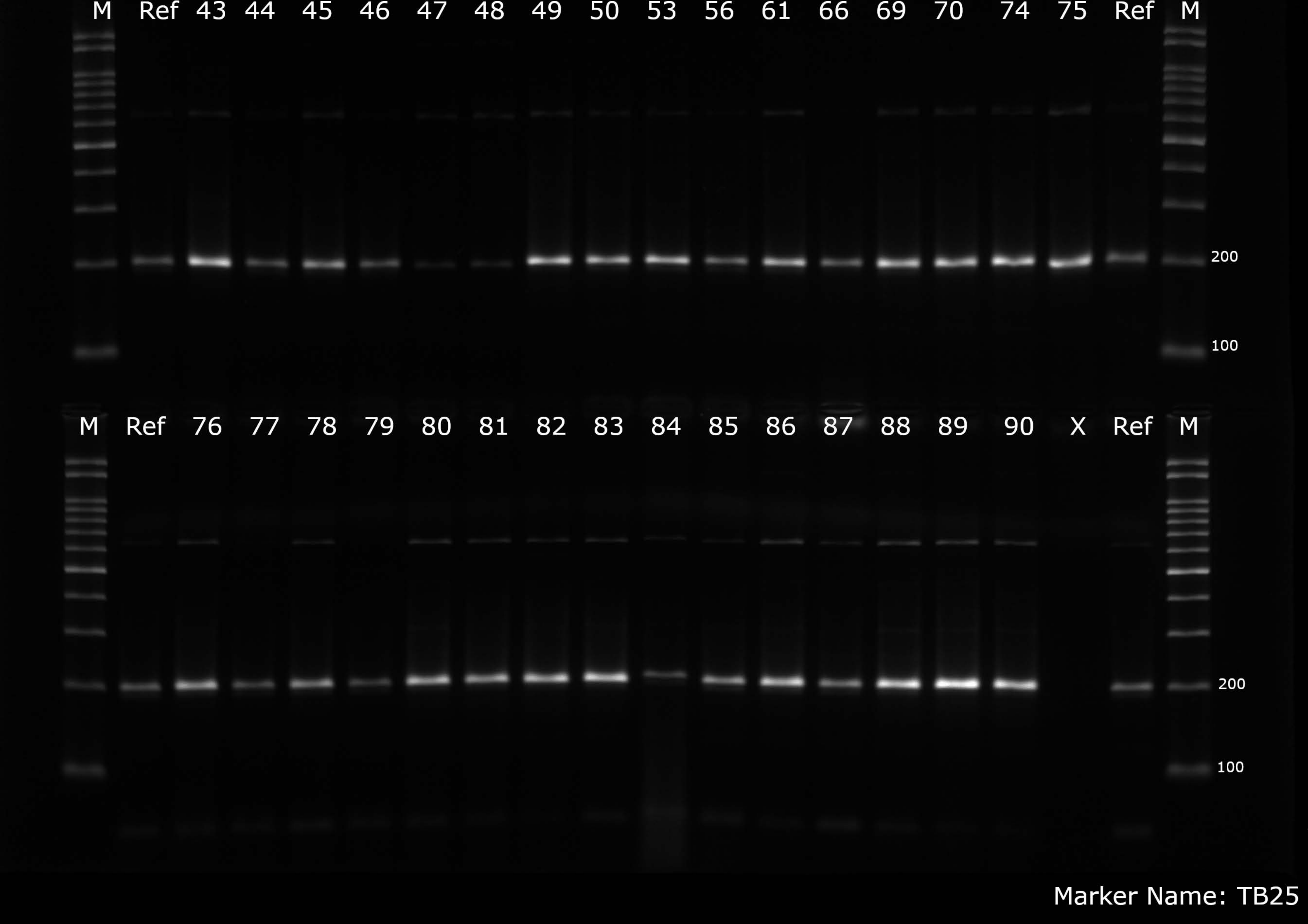
M Ref 43 44 45 46 47 48 49 50 53 56 61 66 69 70 74 75 Ref M
200
100
M Ref 76 77 78 79 80 81 82 83 84 85 86 87 88 89 90 X Ref M
200
100
Marker Name: TB25

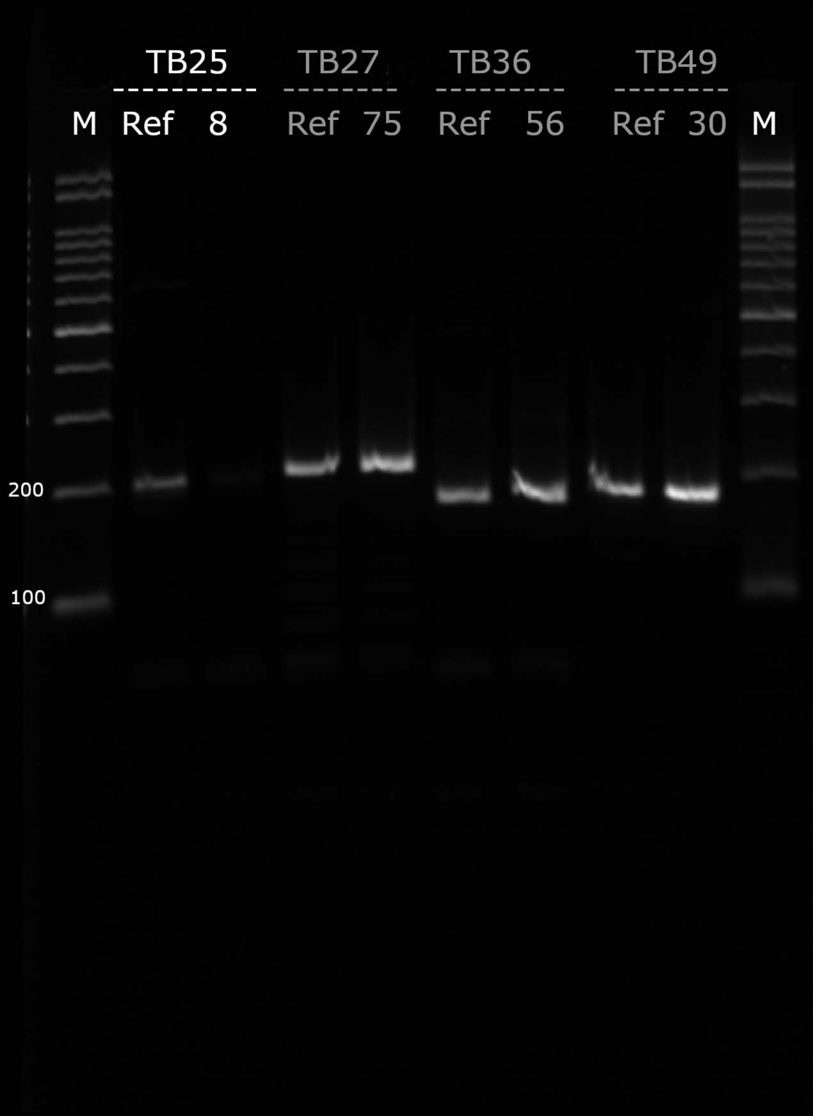
TB25
TB27
TB36
TB49
M
Ref
8
Ref
75
Ref
56
Ref
30
M
200
100

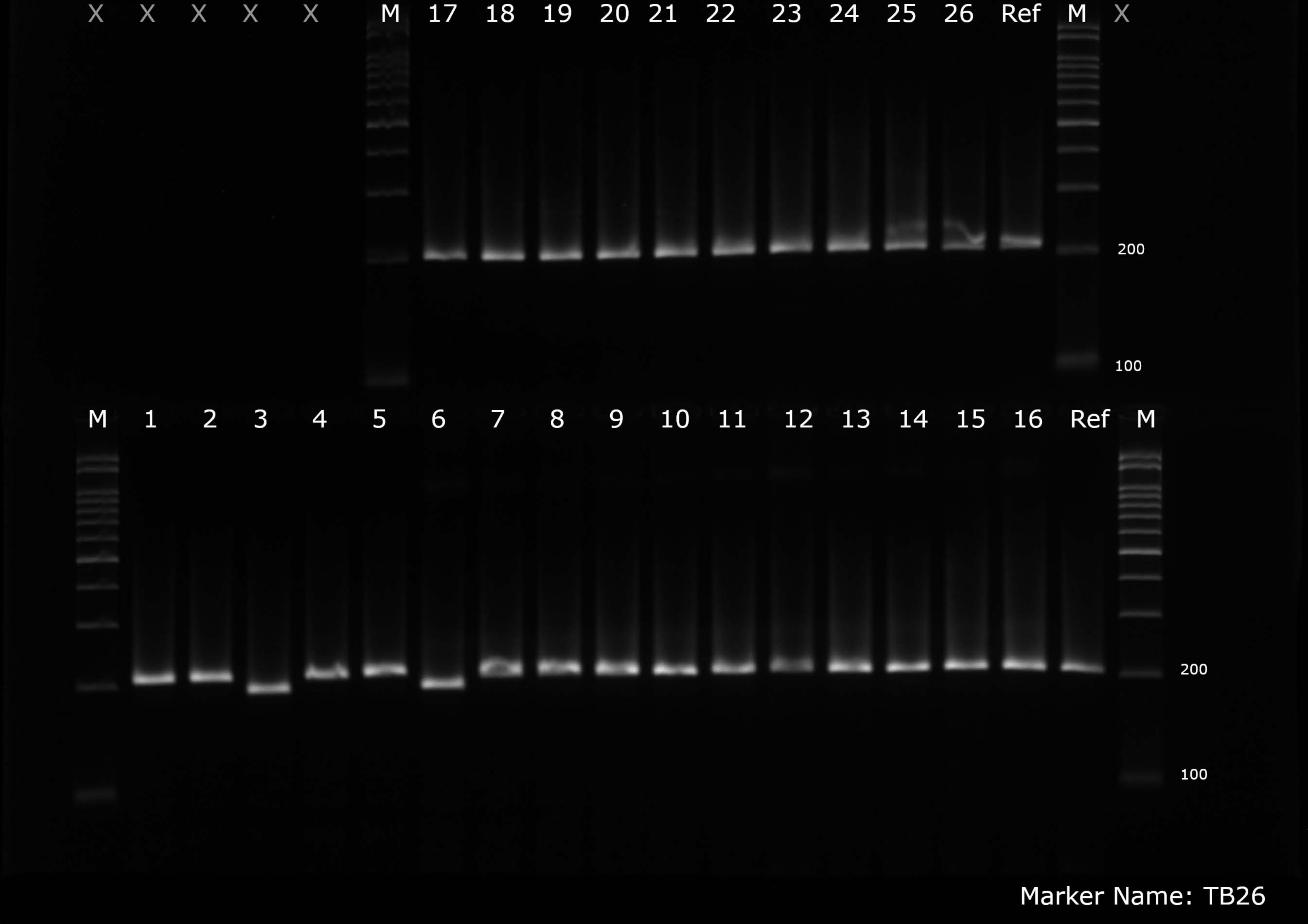

X X X X X M 17 18 19 20 21 22 23 24 25 26 Ref M X
200
100
M 1 2 3 4 5 6 7 8 9 10 11 12 13 14 15 16 Ref M
200
100
Marker Name: TB26

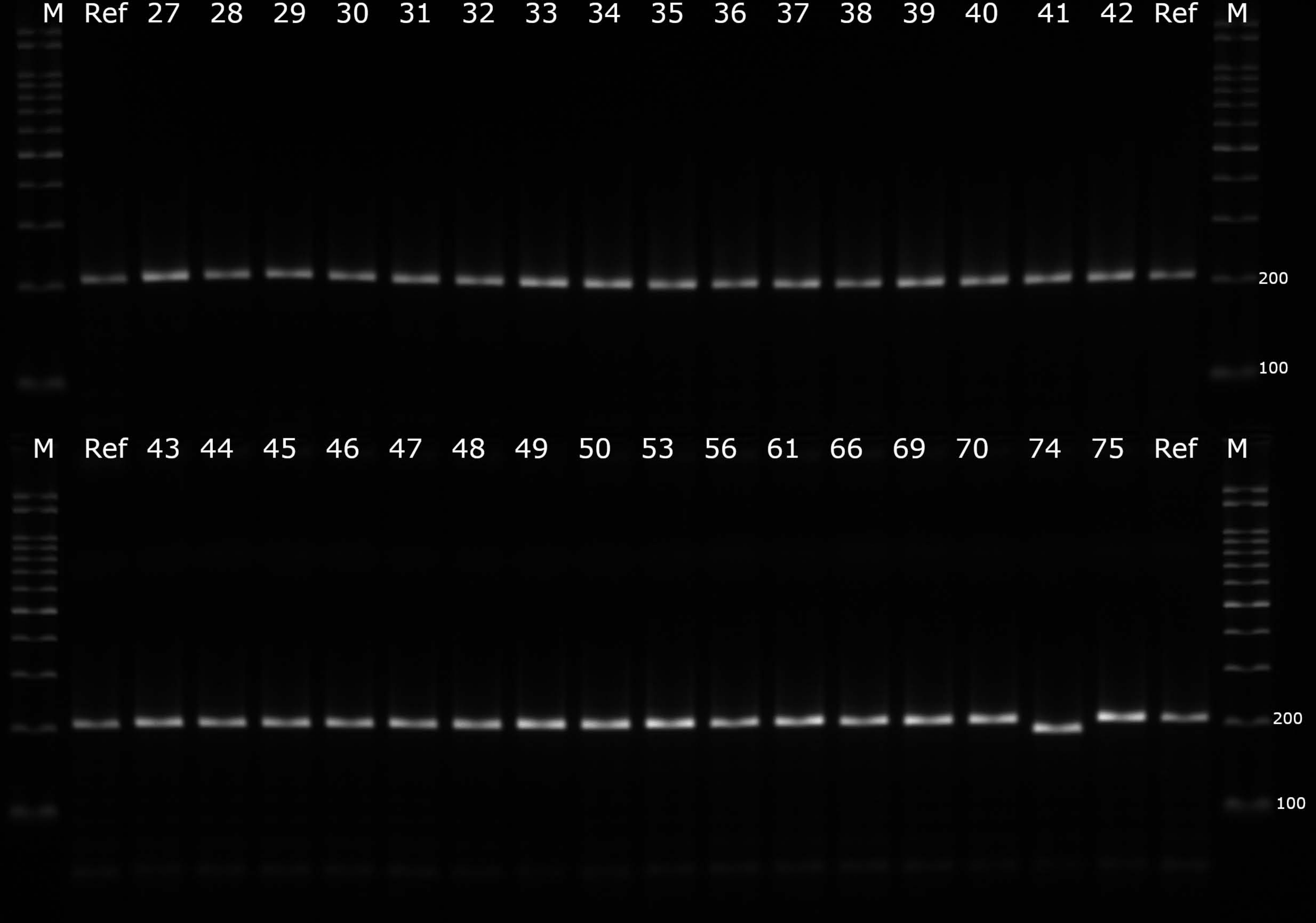

Marker Name: TB26

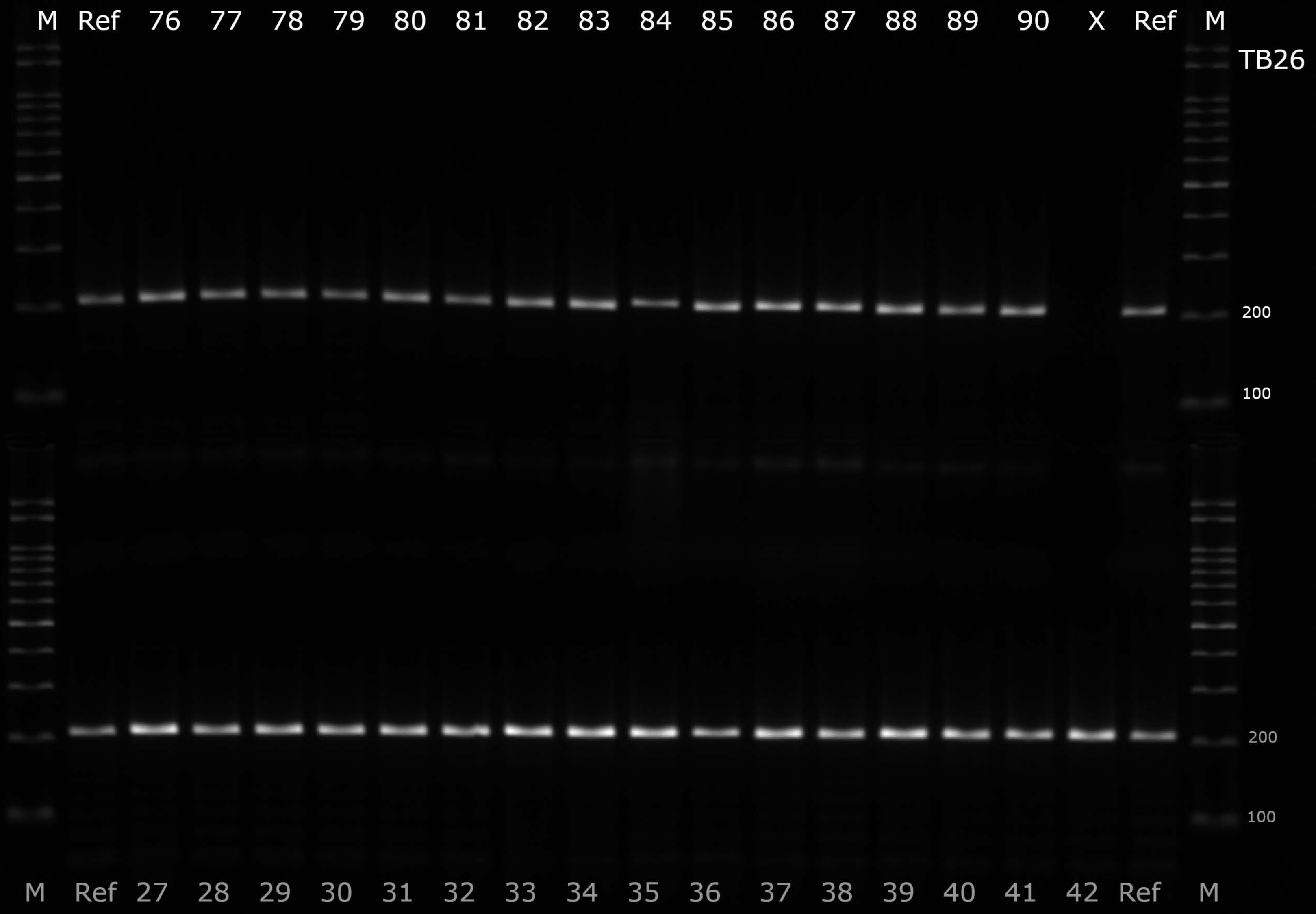

Marker Name: TB27

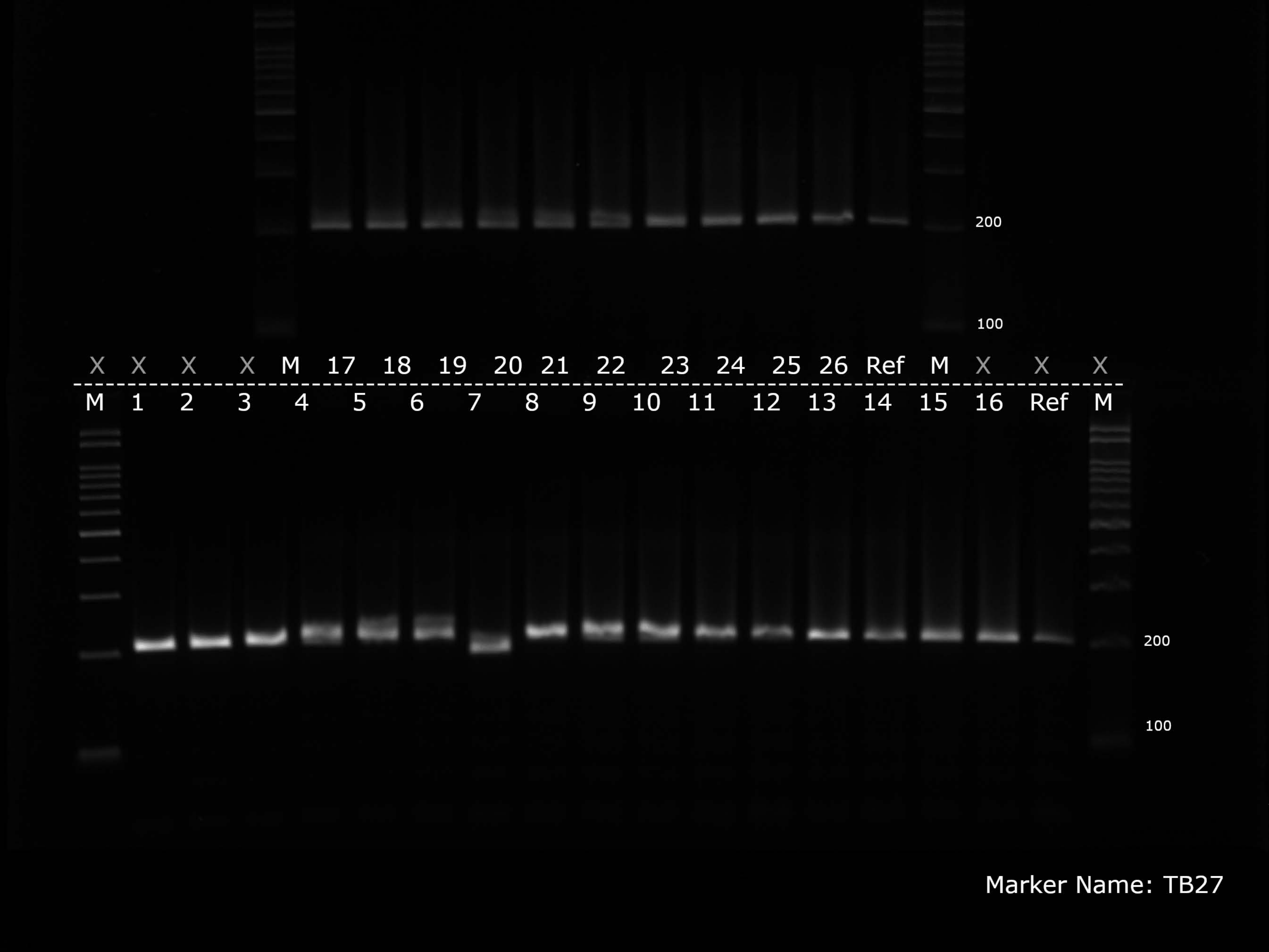

200
100
X X X X M 17 18 19 20 21 22 23 24 25 26 Ref M X X X
M 1 2 3 4 5 6 7 8 9 10 11 12 13 14 15 16 Ref M
200
100
Marker Name: TB27

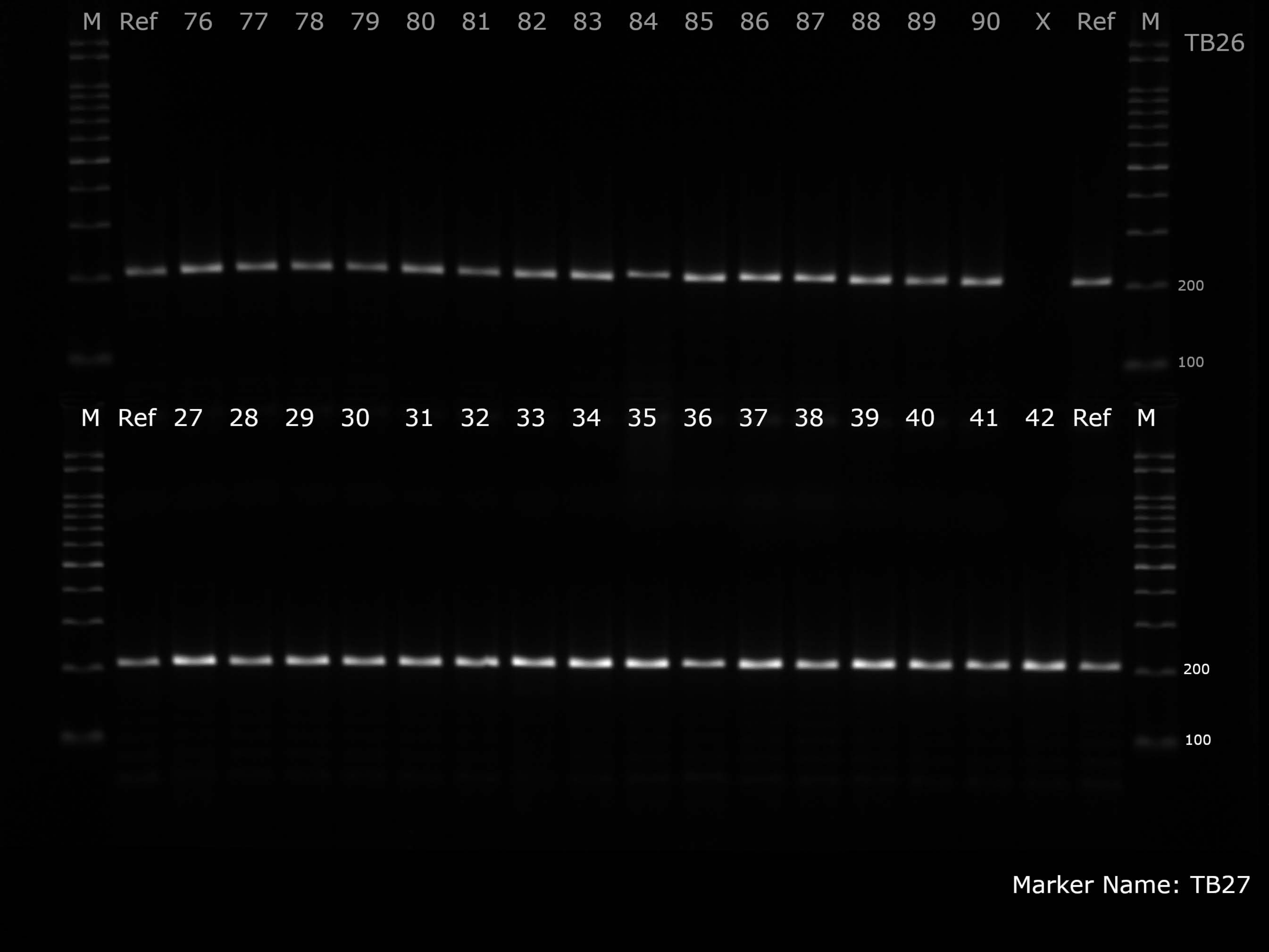
M Ref 76 77 78 79 80 81 82 83 84 85 86 87 88 89 90 X Ref M
TB26
200
100
M Ref 27 28 29 30 31 32 33 34 35 36 37 38 39 40 41 42 Ref M
200
100
Marker Name: TB27

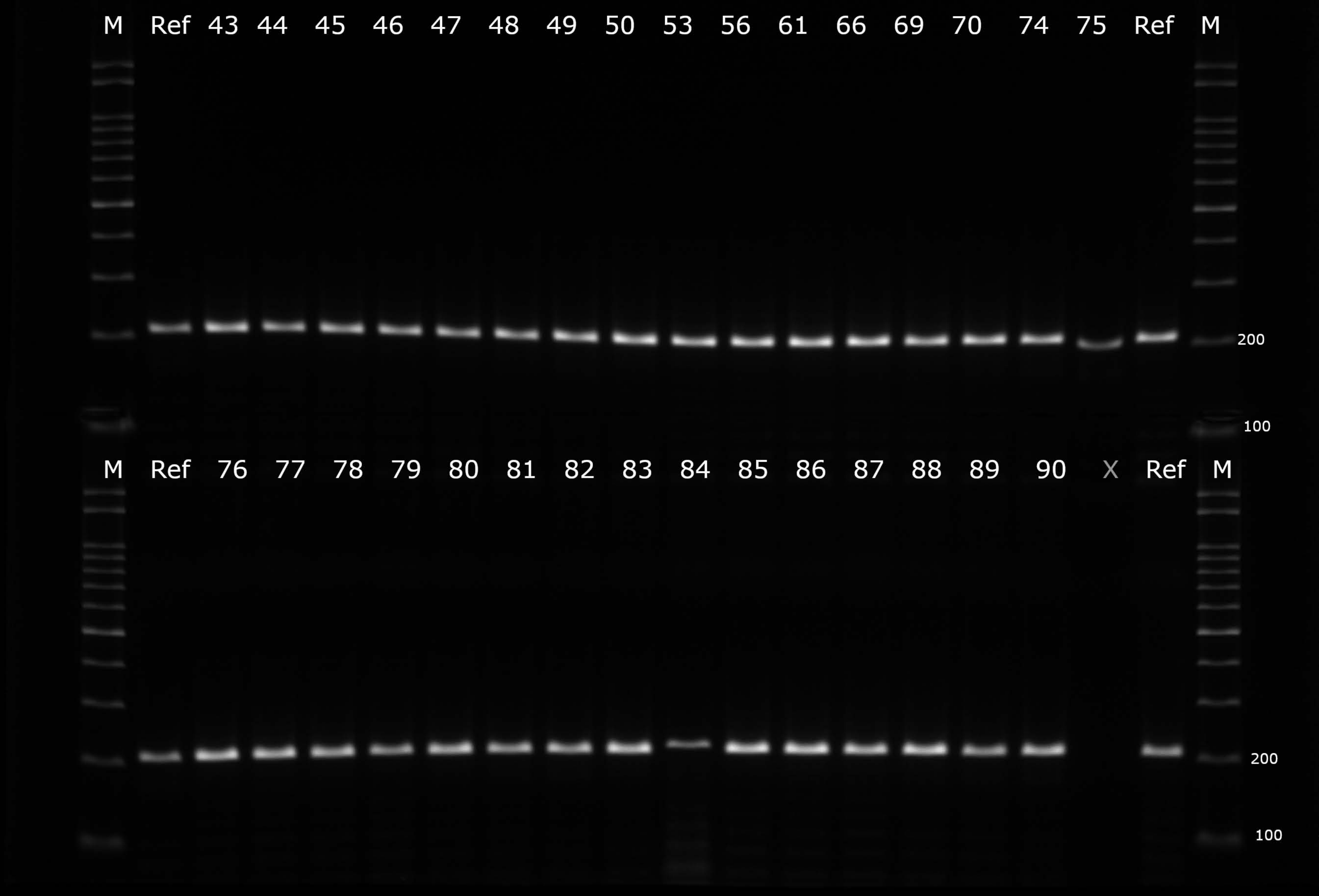

Marker Name: TB27

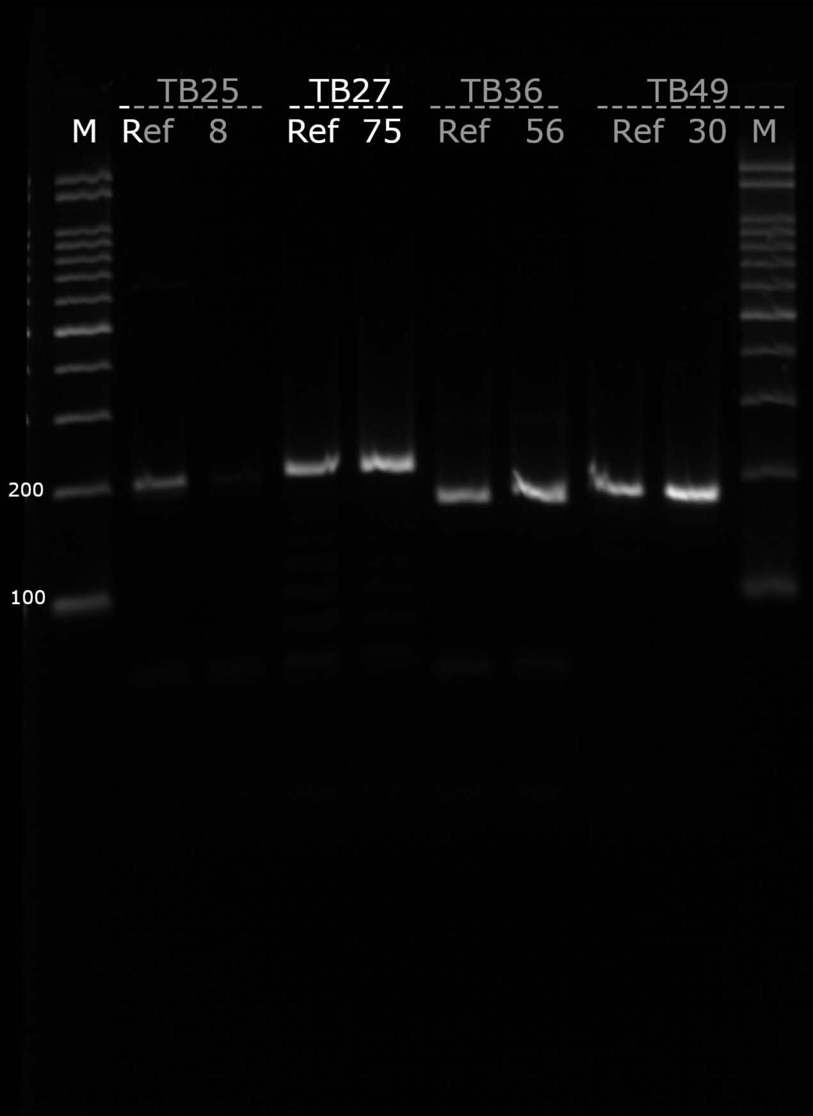
TB25
TB27
TB36
TB49
M
Ref
8
Ref
75
Ref
56
Ref
30
M
200
100

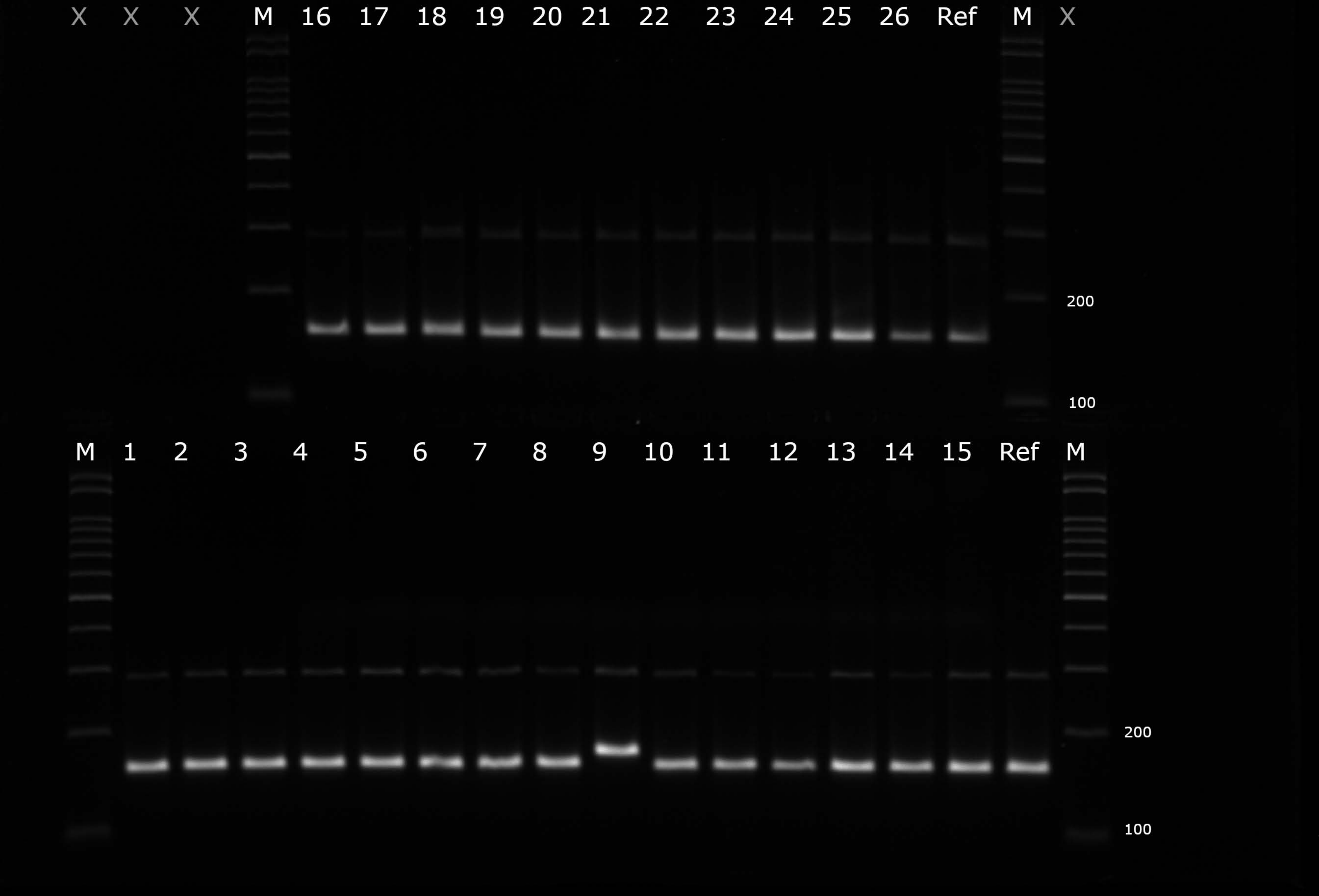

Marker Name: TB28

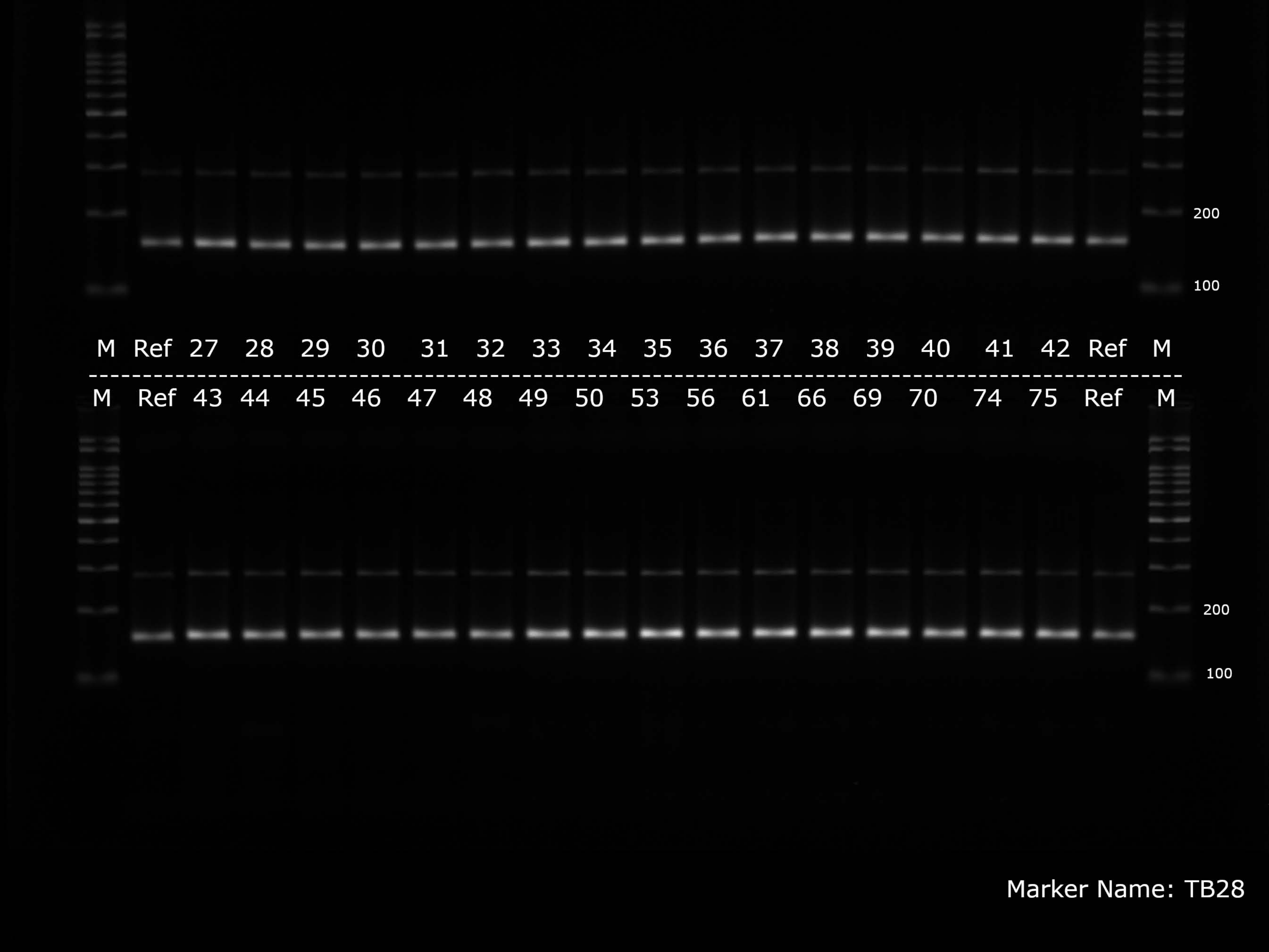
M Ref 27 28 29 30 31 32 33 34 35 36 37 38 39 40 41 42 Ref M
M Ref 43 44 45 46 47 48 49 50 53 56 61 66 69 70 74 75 Ref M
200
100
200
100
Marker Name: TB28

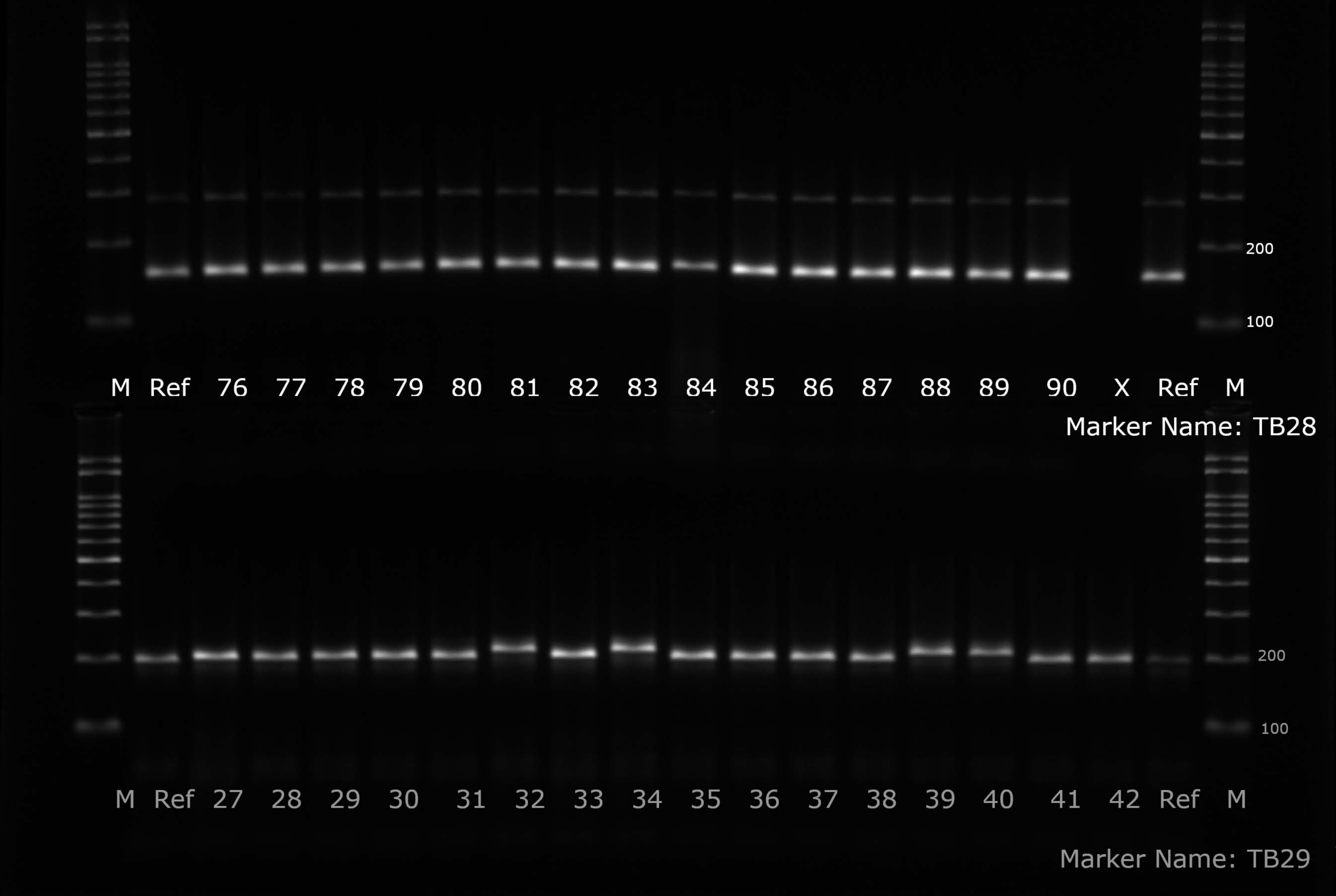
200
100
M Ref 76 77 78 79 80 81 82 83 84 85 86 87 88 89 90 X Ref M
Marker Name: TB28
200
100
M Ref 27 28 29 30 31 32 33 34 35 36 37 38 39 40 41 42 Ref M
Marker Name: TB29

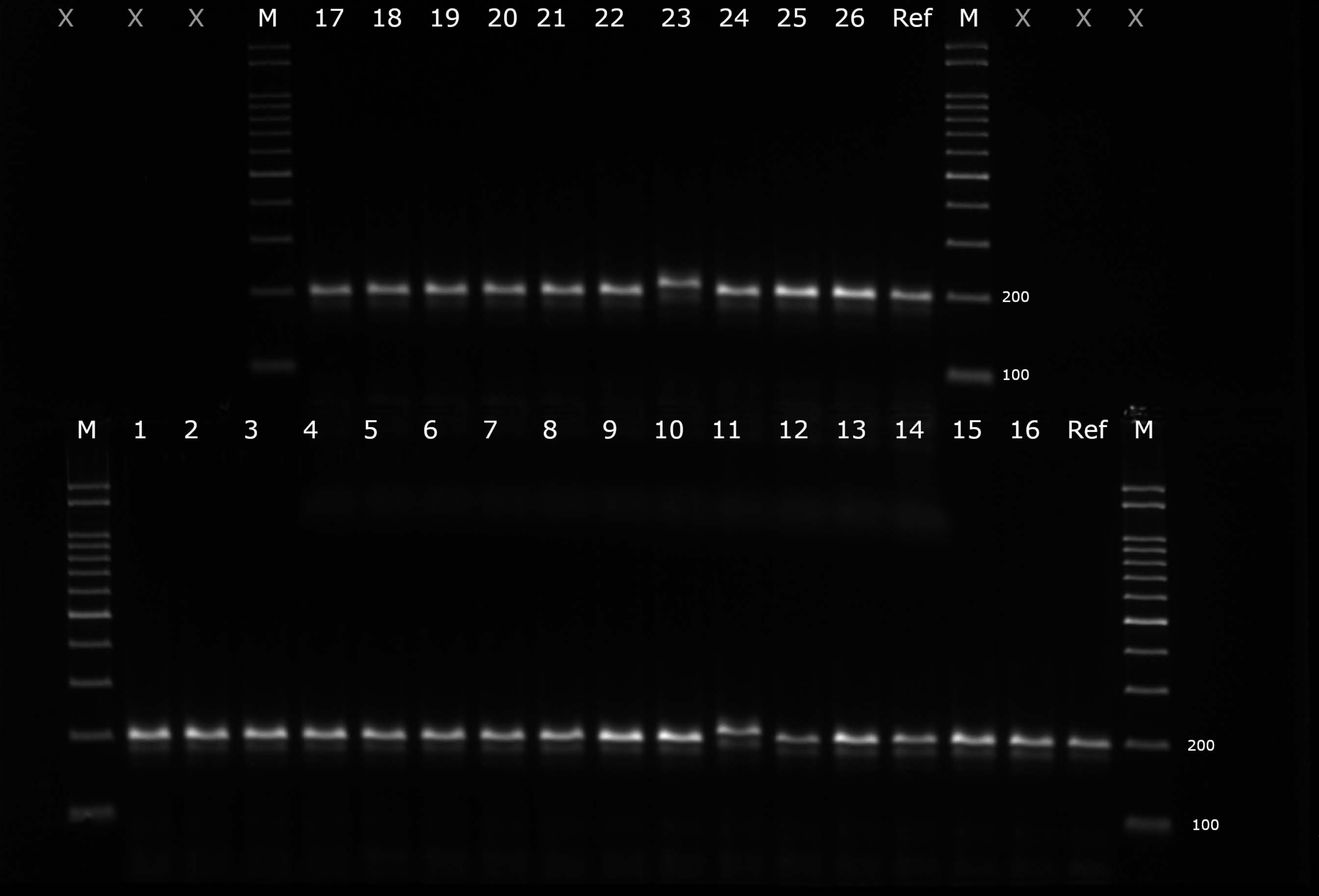

Marker Name: TB29

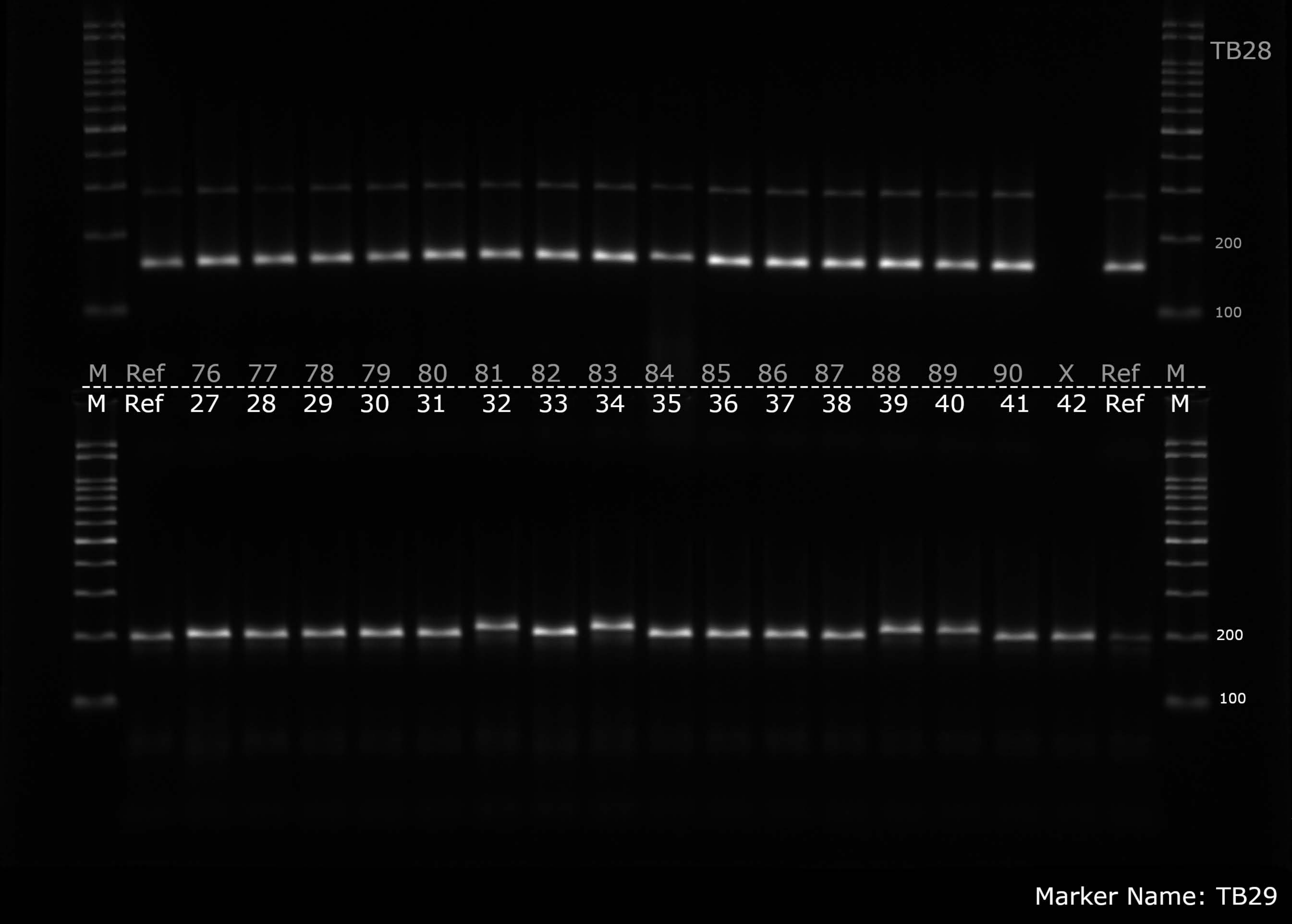
TB28
200
100
M Ref 76 77 78 79 80 81 82 83 84 85 86 87 88 89 90 X Ref M
M Ref 27 28 29 30 31 32 33 34 35 36 37 38 39 40 41 42 Ref M
200
100
Marker Name: TB29

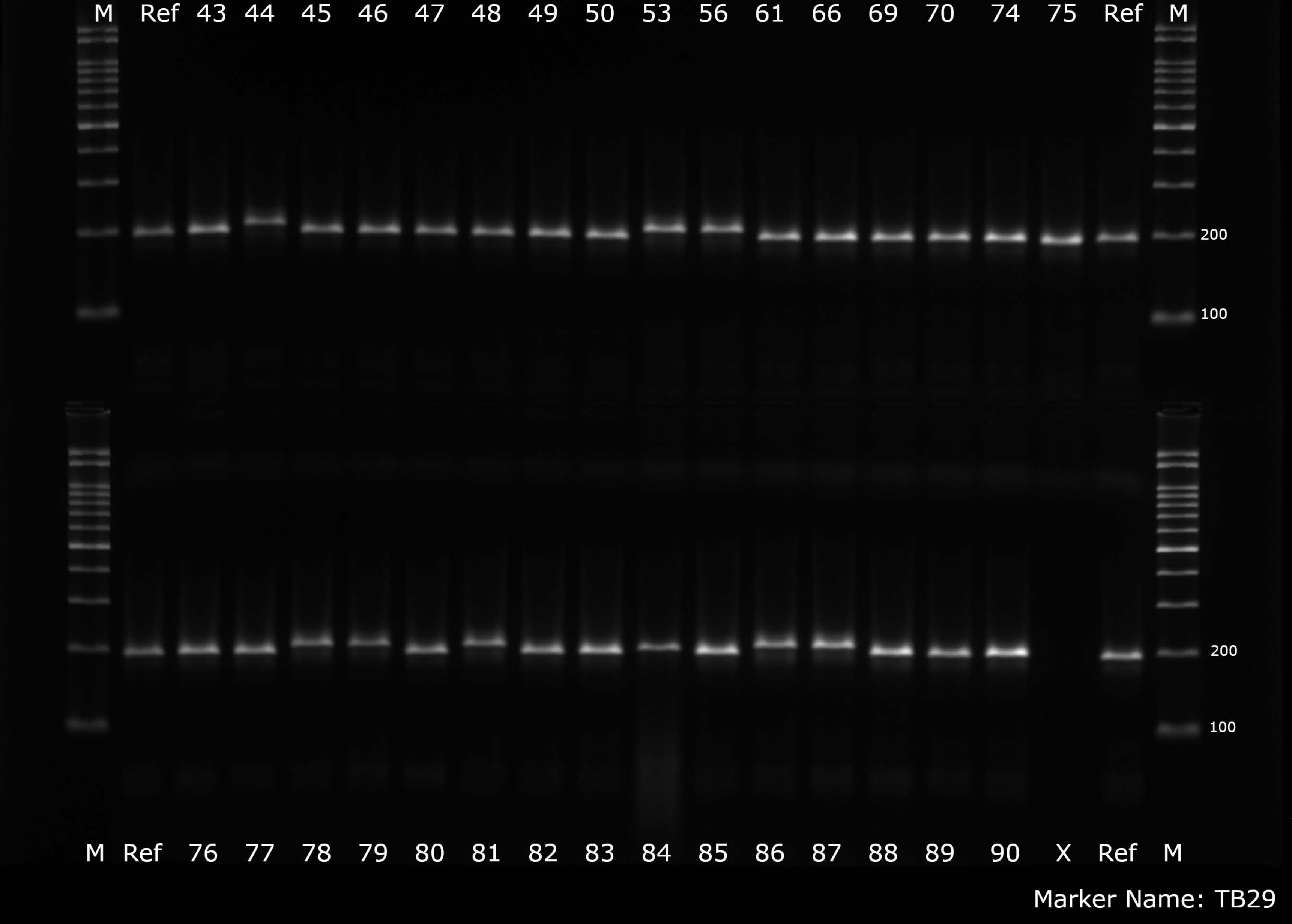
M Ref 43 44 45 46 47 48 49 50 53 56 61 66 69 70 74 75 Ref M
200
100
200
100
M Ref 76 77 78 79 80 81 82 83 84 85 86 87 88 89 90 X Ref M
Marker Name: TB29

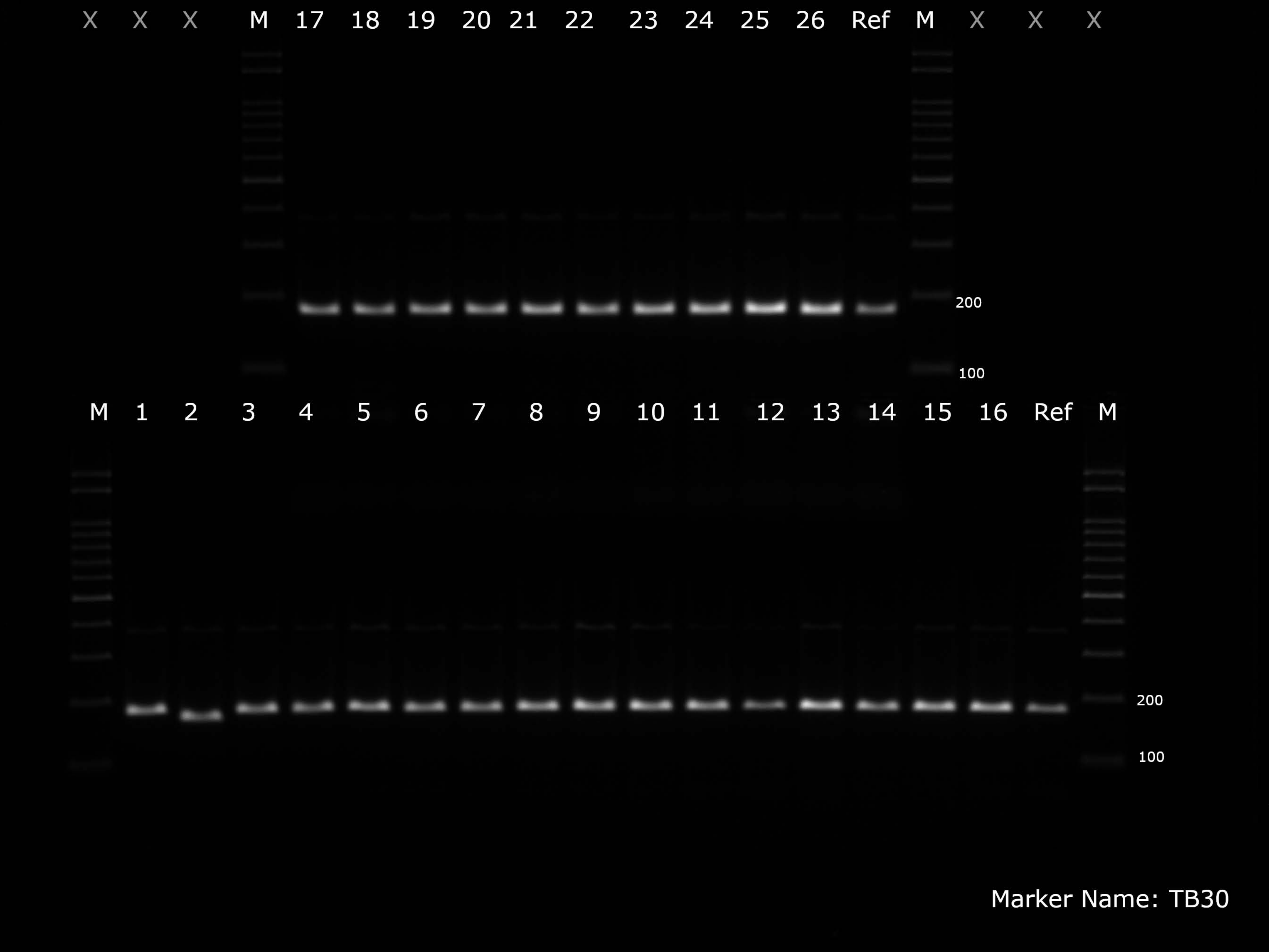
X X X M 17 18 19 20 21 22 23 24 25 26 Ref M X X X
200
100
M 1 2 3 4 5 6 7 8 9 10 11 12 13 14 15 16 Ref M
200
100
Marker Name: TB30

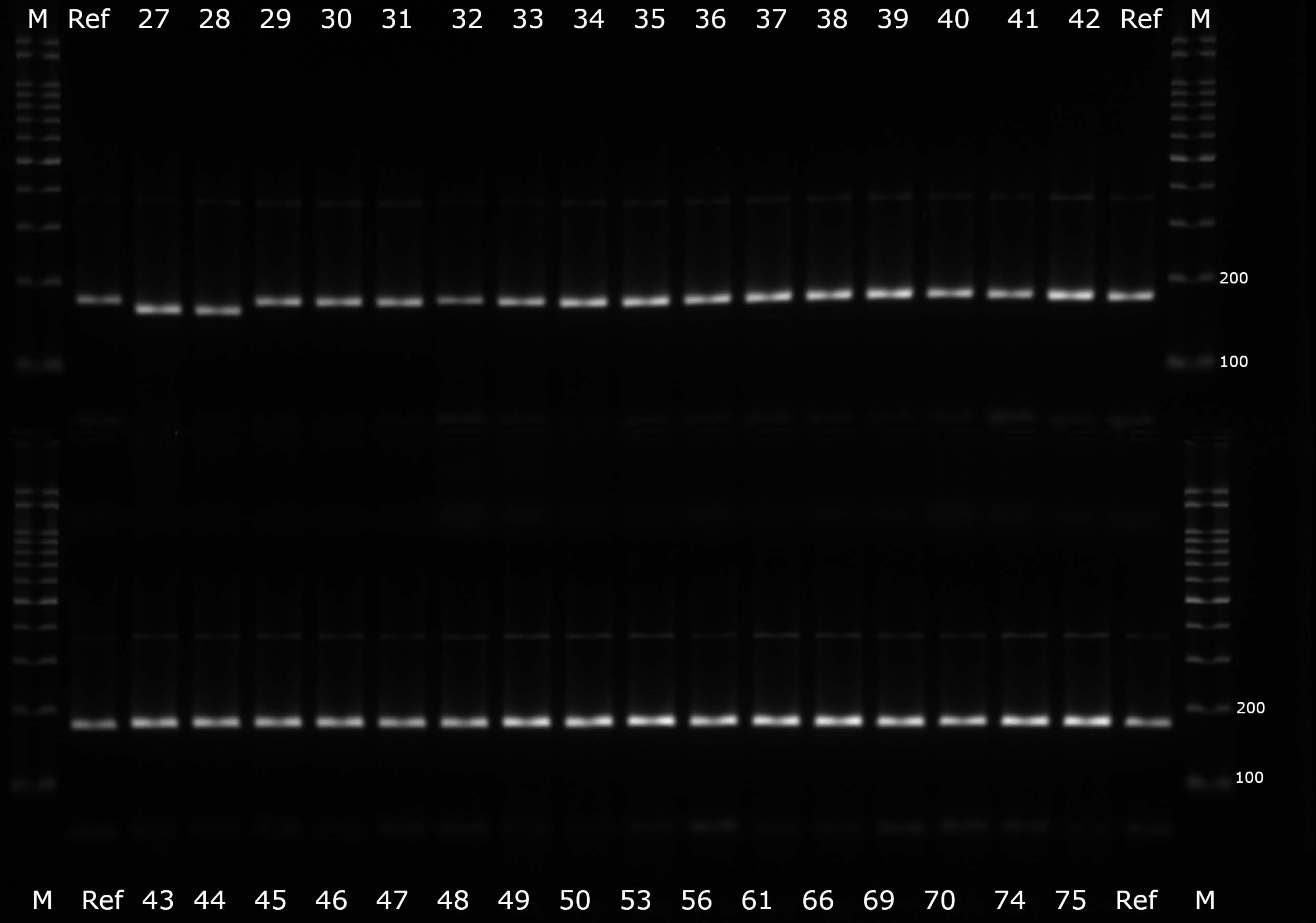

Marker Name: TB30

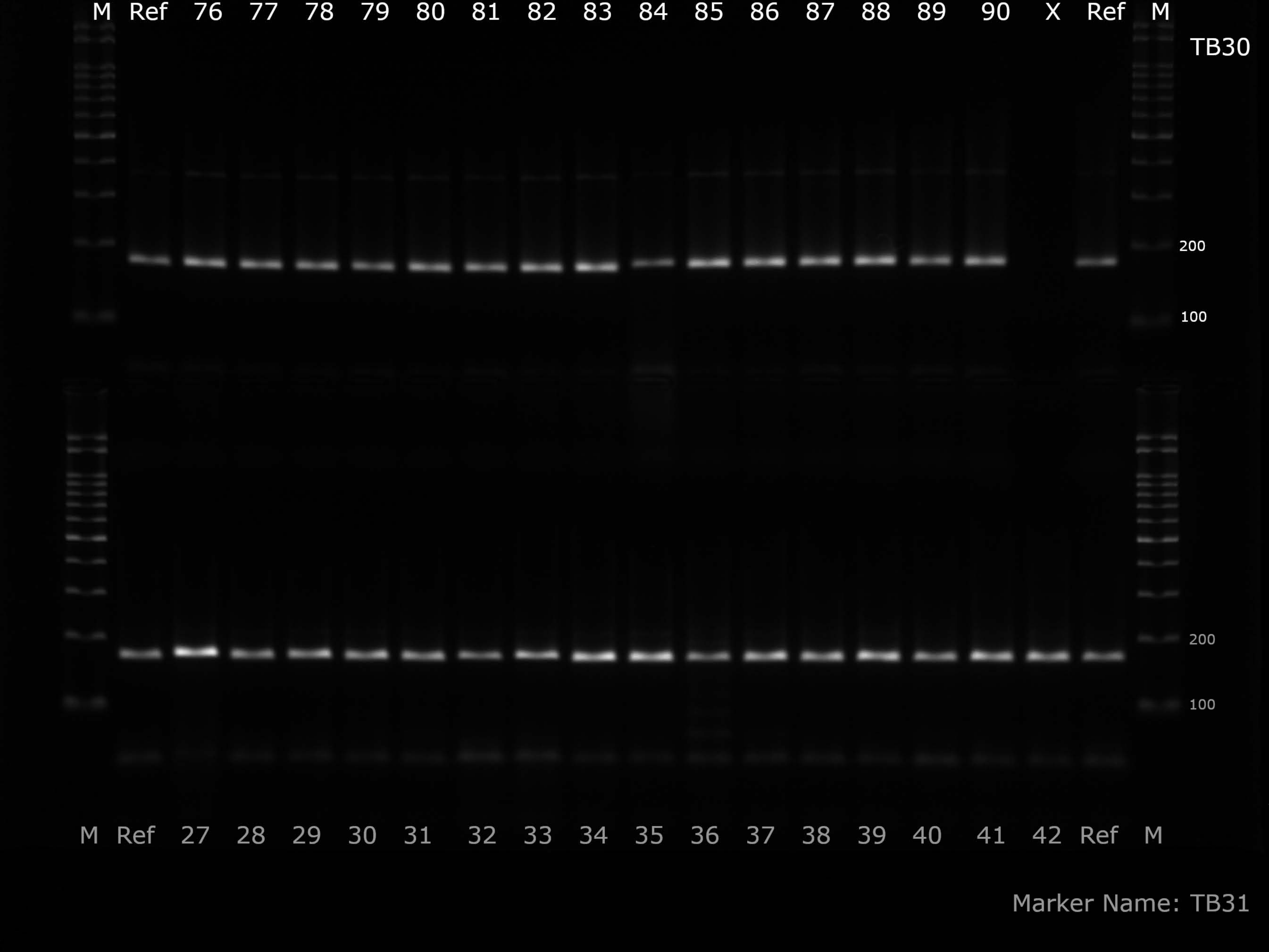
M Ref 76 77 78 79 80 81 82 83 84 85 86 87 88 89 90 X Ref M
TB30
200
100
200
100
M Ref 27 28 29 30 31 32 33 34 35 36 37 38 39 40 41 42 Ref M
Marker Name: TB31

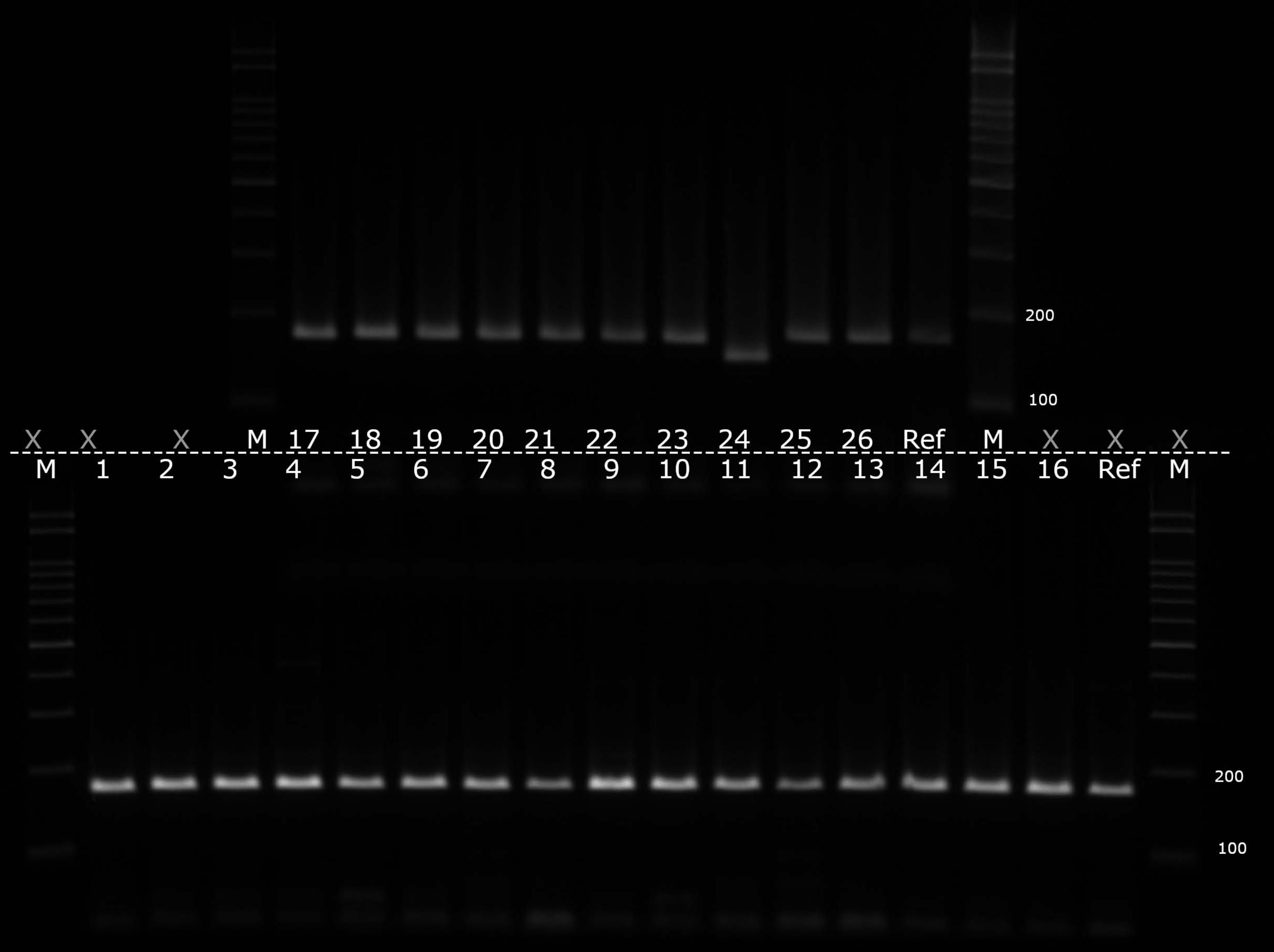

Marker Name: TB31

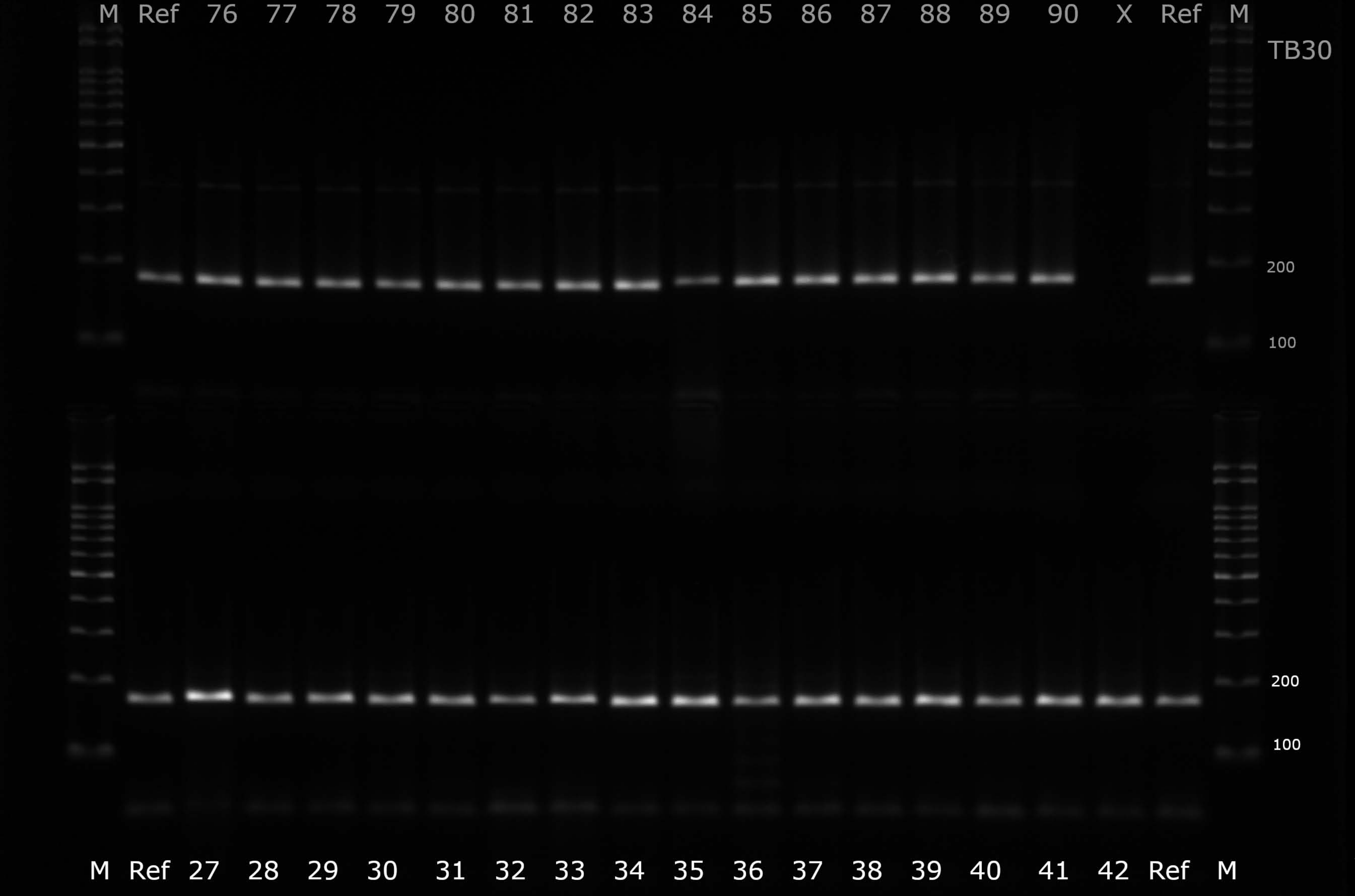

Marker Name: TB31

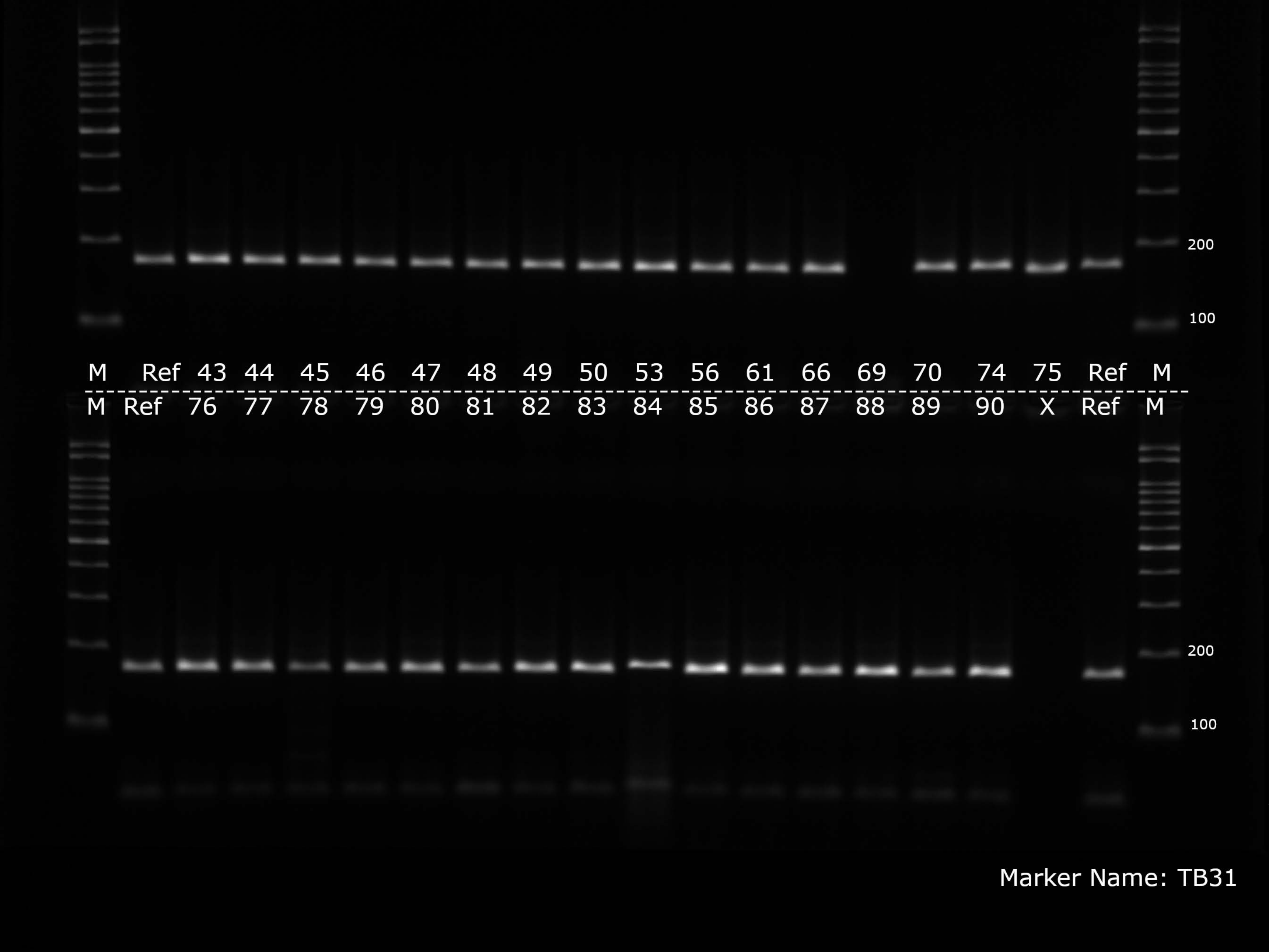
M Ref 43 44 45 46 47 48 49 50 53 56 61 66 69 70 74 75 Ref M
M Ref 76 77 78 79 80 81 82 83 84 85 86 87 88 89 90 X Ref M
200
100
200
100
Marker Name: TB31

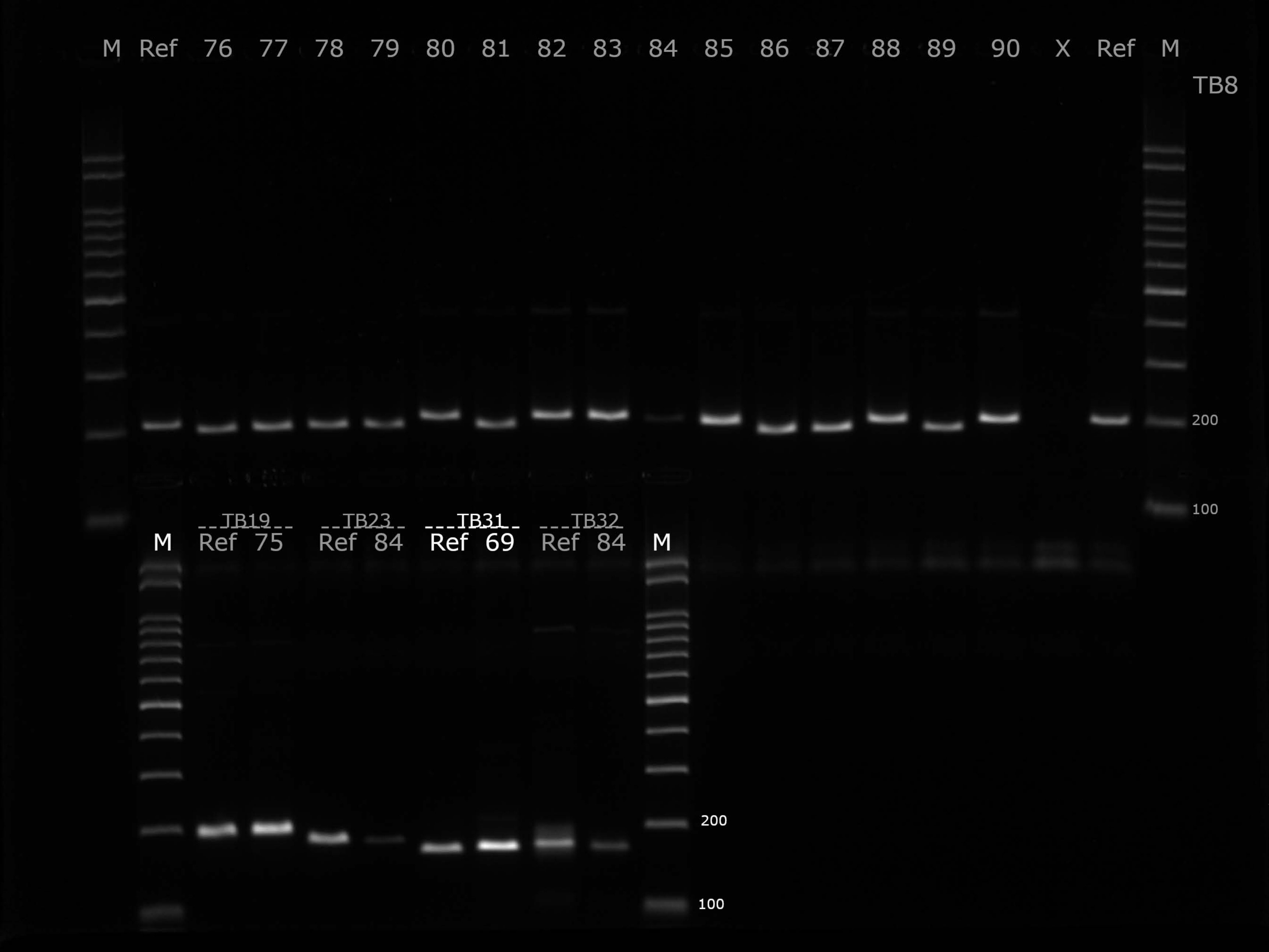

M Ref 76 77 78 79 80 81 82 83 84 85 86 87 88 89 90 X Ref M
TB8
200
100
TB19
TB23
TB31
TB32
M Ref 75 Ref 84 Ref 69 Ref 84 M
200
100

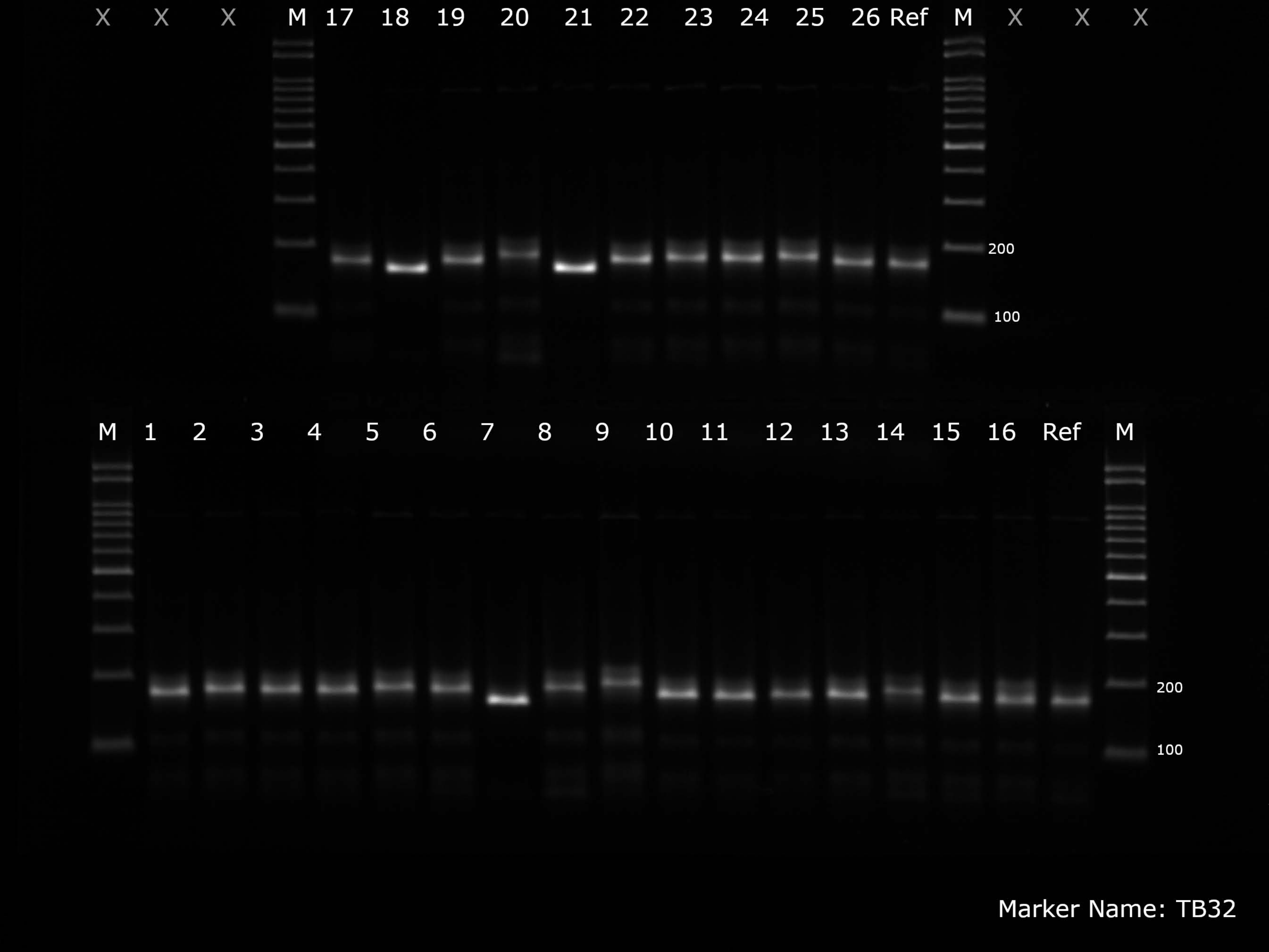
X X X M 17 18 19 20 21 22 23 24 25 26 Ref M X X X
200
100
M 1 2 3 4 5 6 7 8 9 10 11 12 13 14 15 16 Ref M
200
100
Marker Name: TB32

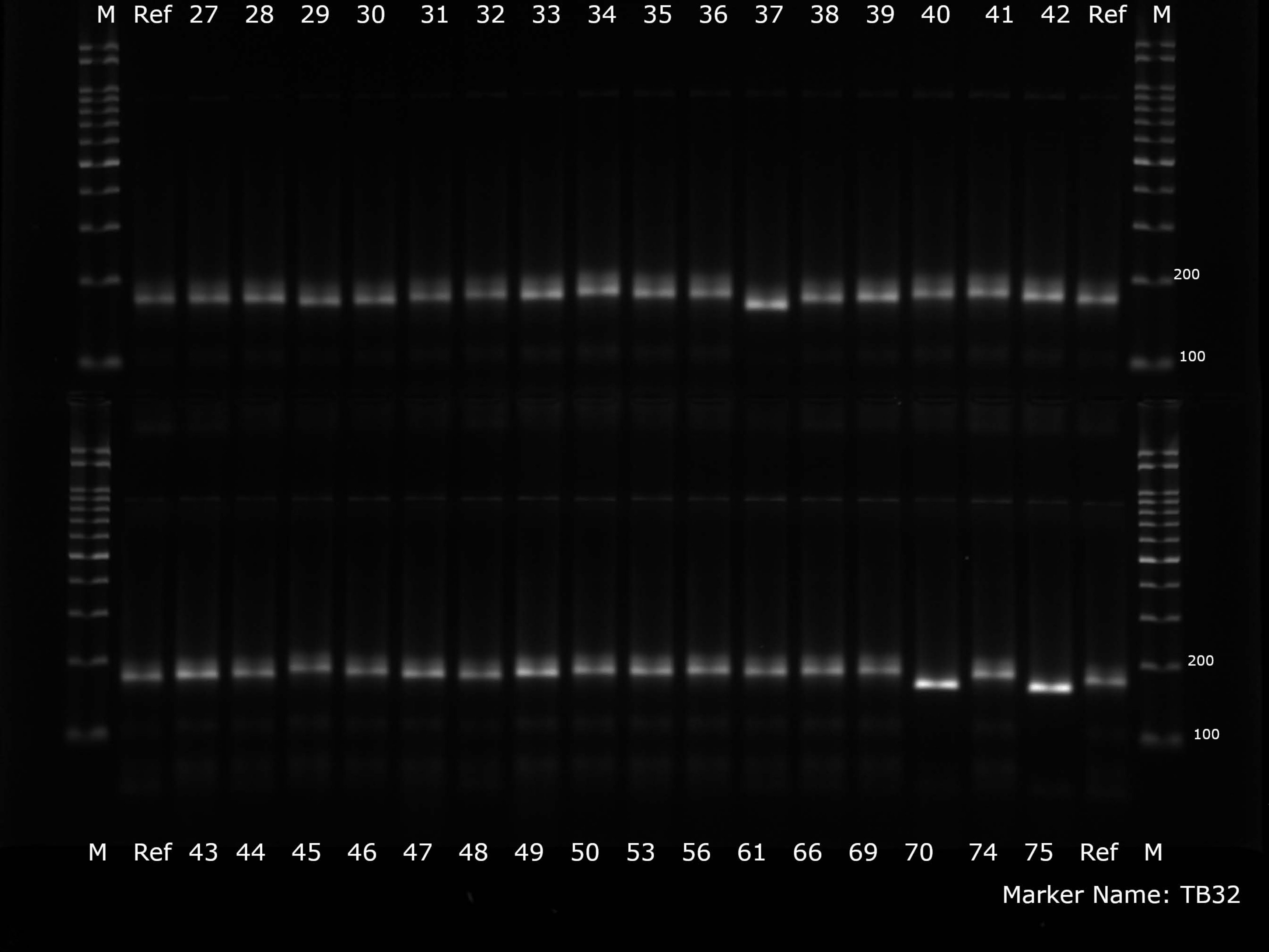
M Ref 27 28 29 30 31 32 33 34 35 36 37 38 39 40 41 42 Ref M
200
100
200
100
M Ref 43 44 45 46 47 48 49 50 53 56 61 66 69 70 74 75 Ref M
Marker Name: TB32

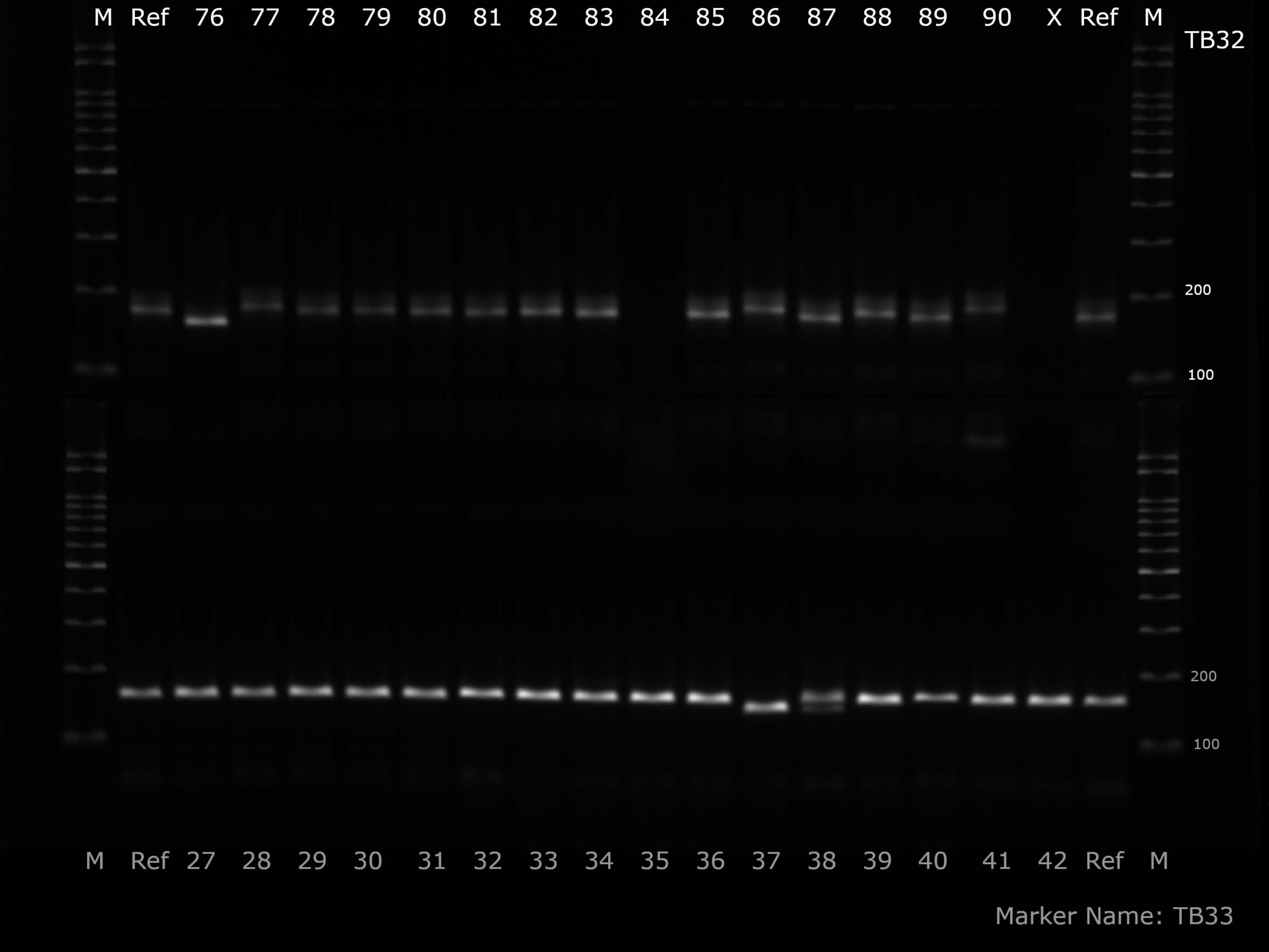
M Ref 76 77 78 79 80 81 82 83 84 85 86 87 88 89 90 X Ref M
TB32
200
100
200
100
M Ref 27 28 29 30 31 32 33 34 35 36 37 38 39 40 41 42 Ref M
Marker Name: TB33

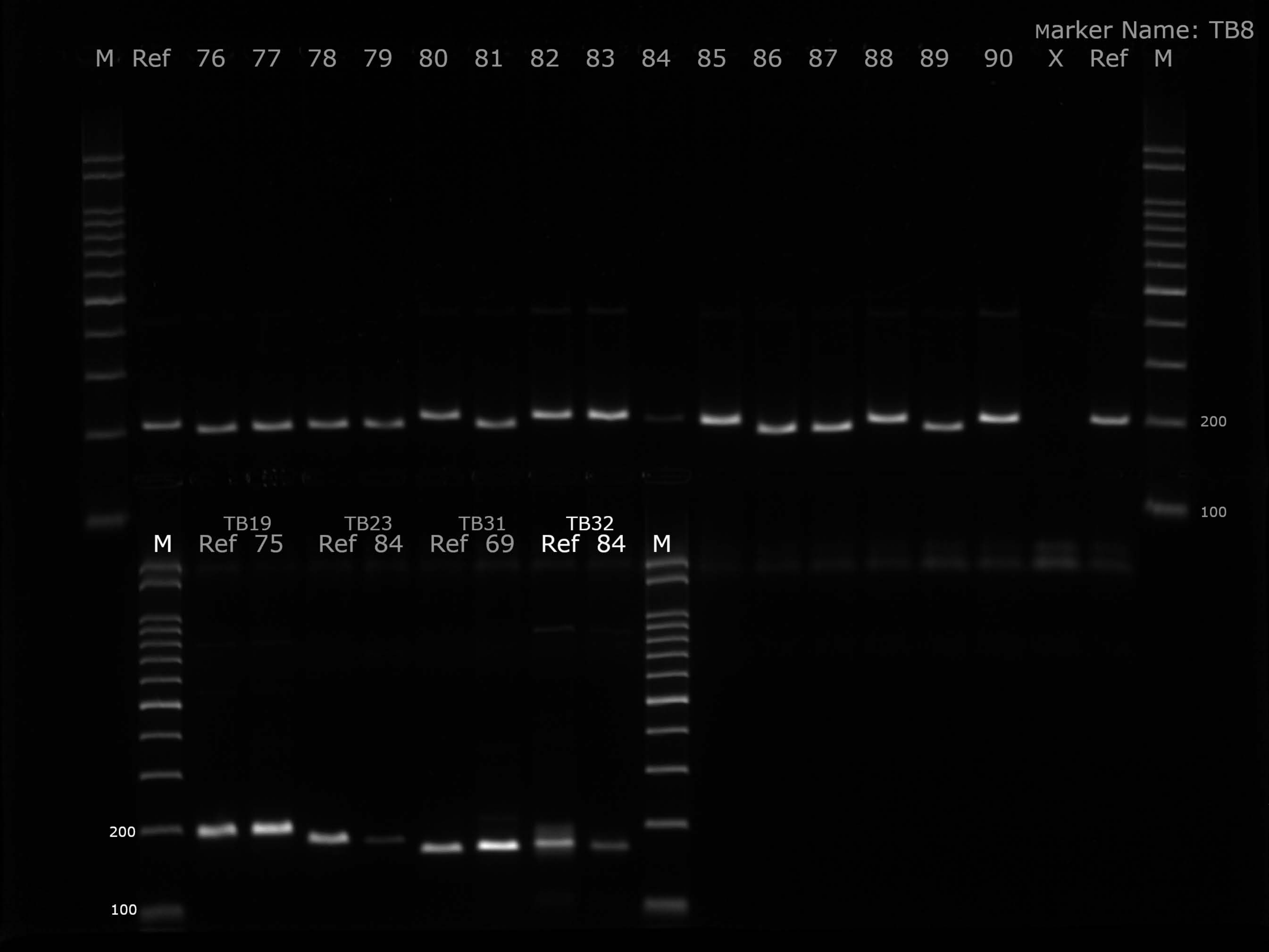
Marker Name: TB8
M Ref 76 77 78 79 80 81 82 83 84 85 86 87 88 89 90 X Ref M
200
100
TB19 TB23 TB31 TB32
M Ref 75 Ref 84 Ref 69 Ref 84 M
200
100

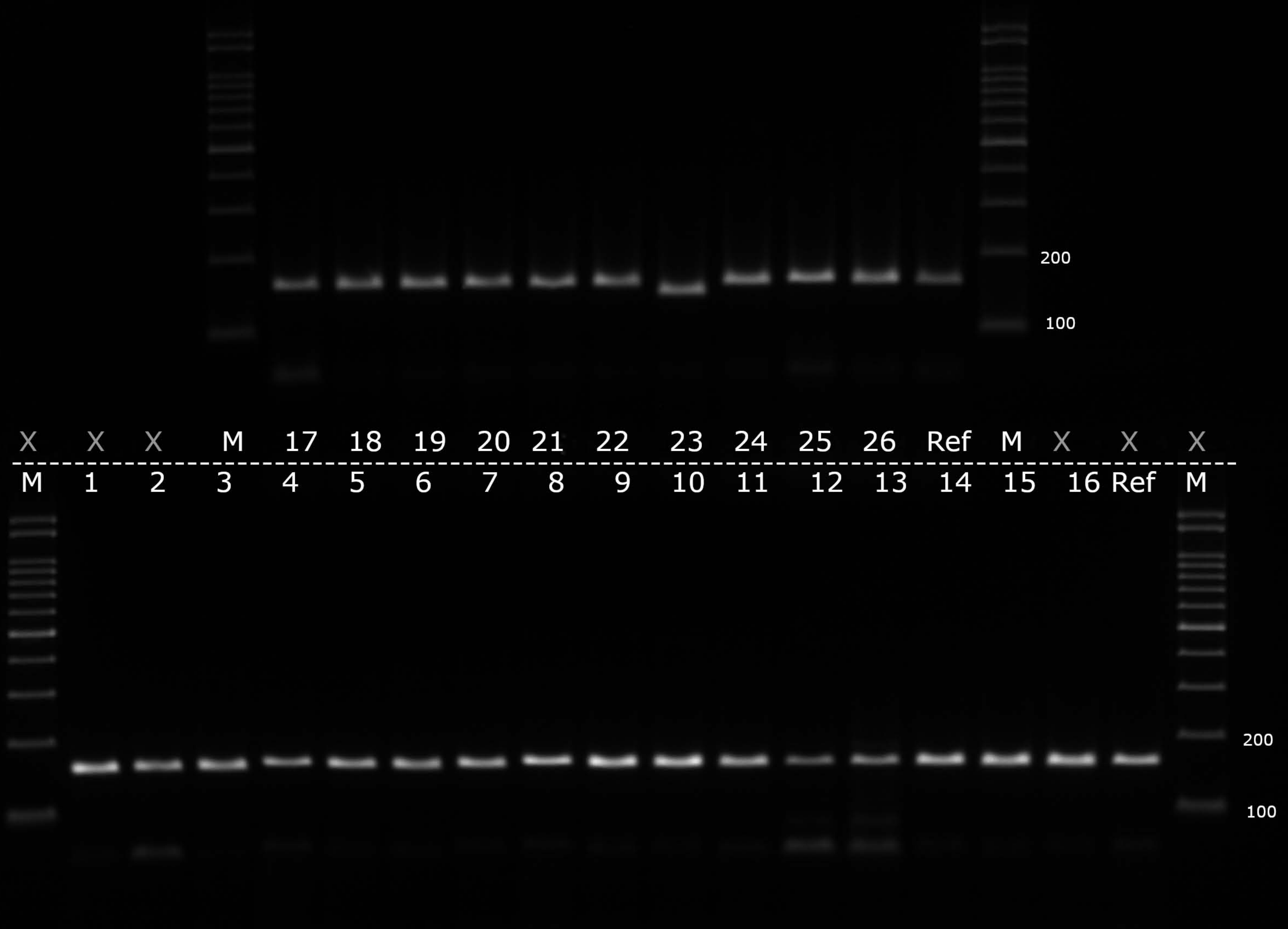

Marker Name: TB33

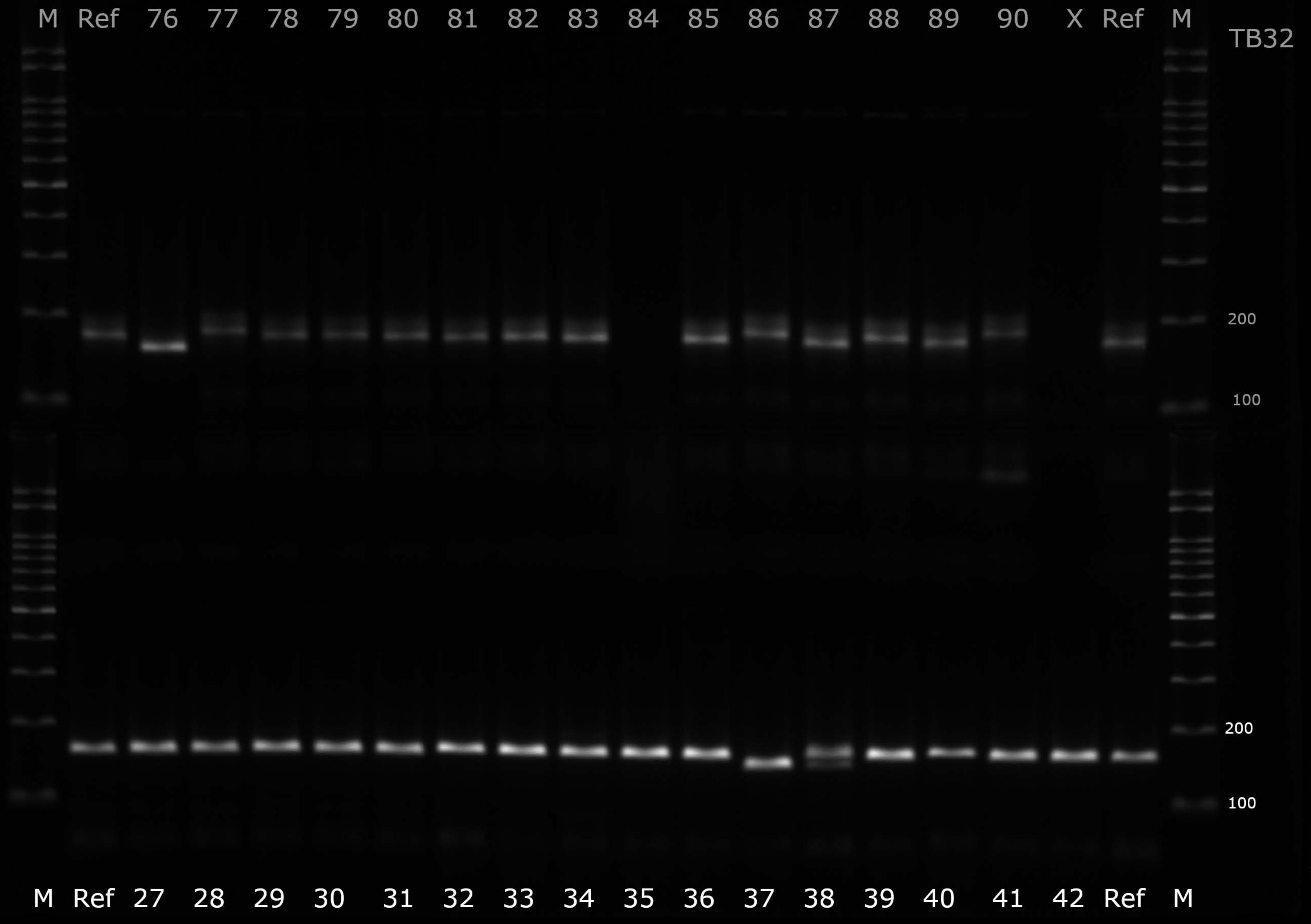

Marker Name: TB33

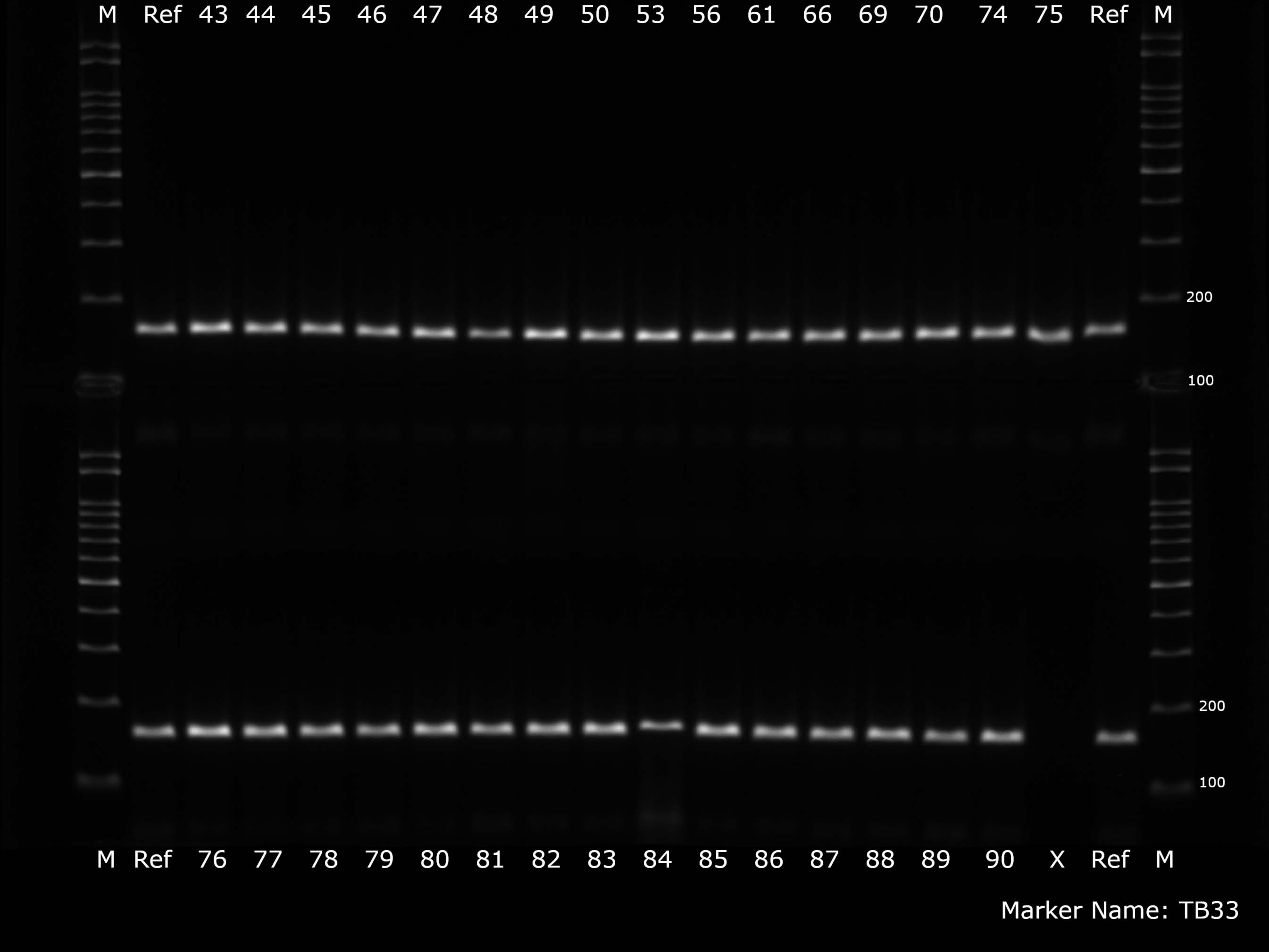
M Ref 43 44 45 46 47 48 49 50 53 56 61 66 69 70 74 75 Ref M
200
100
200
100
M Ref 76 77 78 79 80 81 82 83 84 85 86 87 88 89 90 X Ref M
Marker Name: TB33

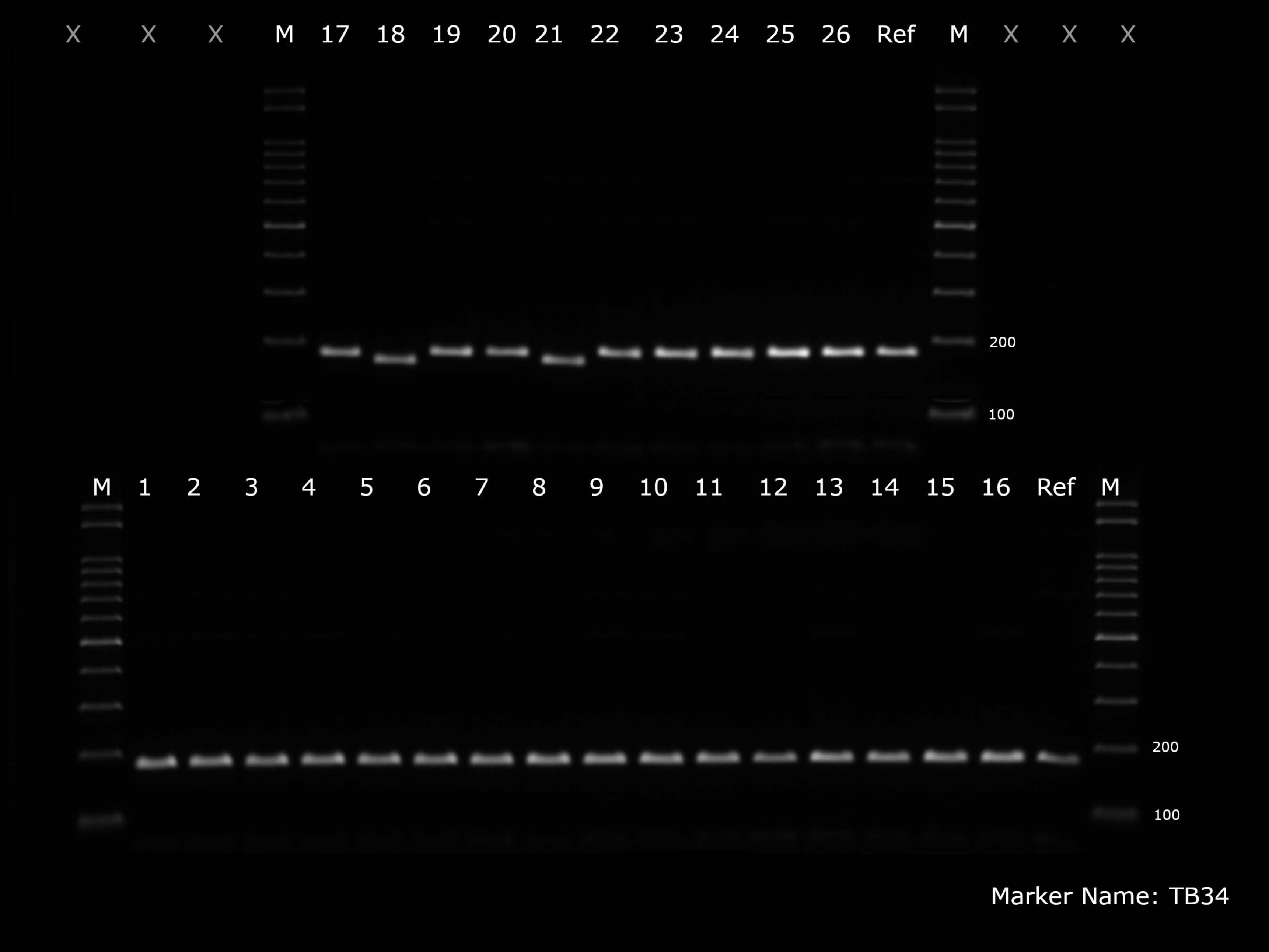
X X X M 17 18 19 20 21 22 23 24 25 26 Ref M X X X
200
100
M 1 2 3 4 5 6 7 8 9 10 11 12 13 14 15 16 Ref M
200
100
Marker Name: TB34

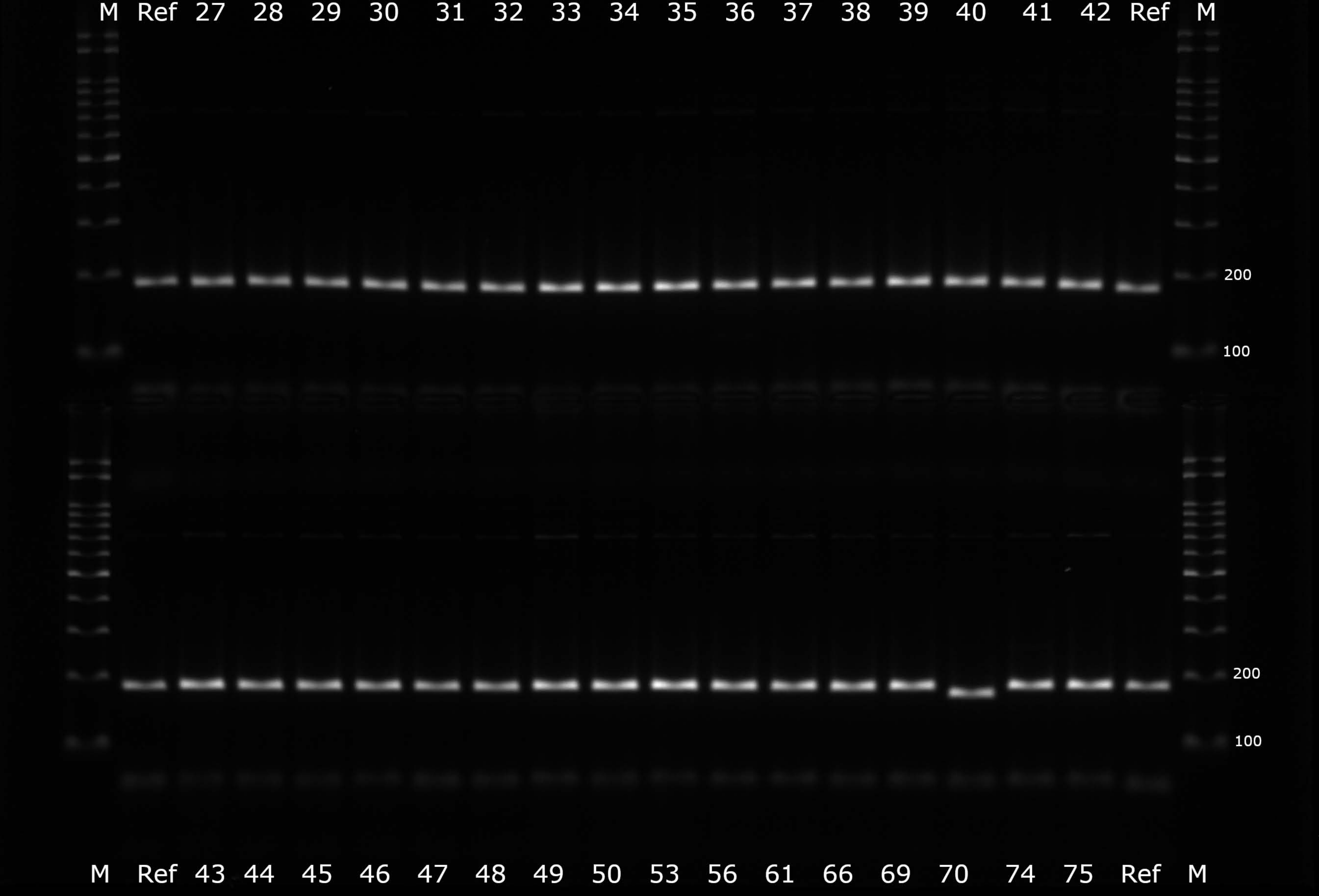

Marker Name: TB34

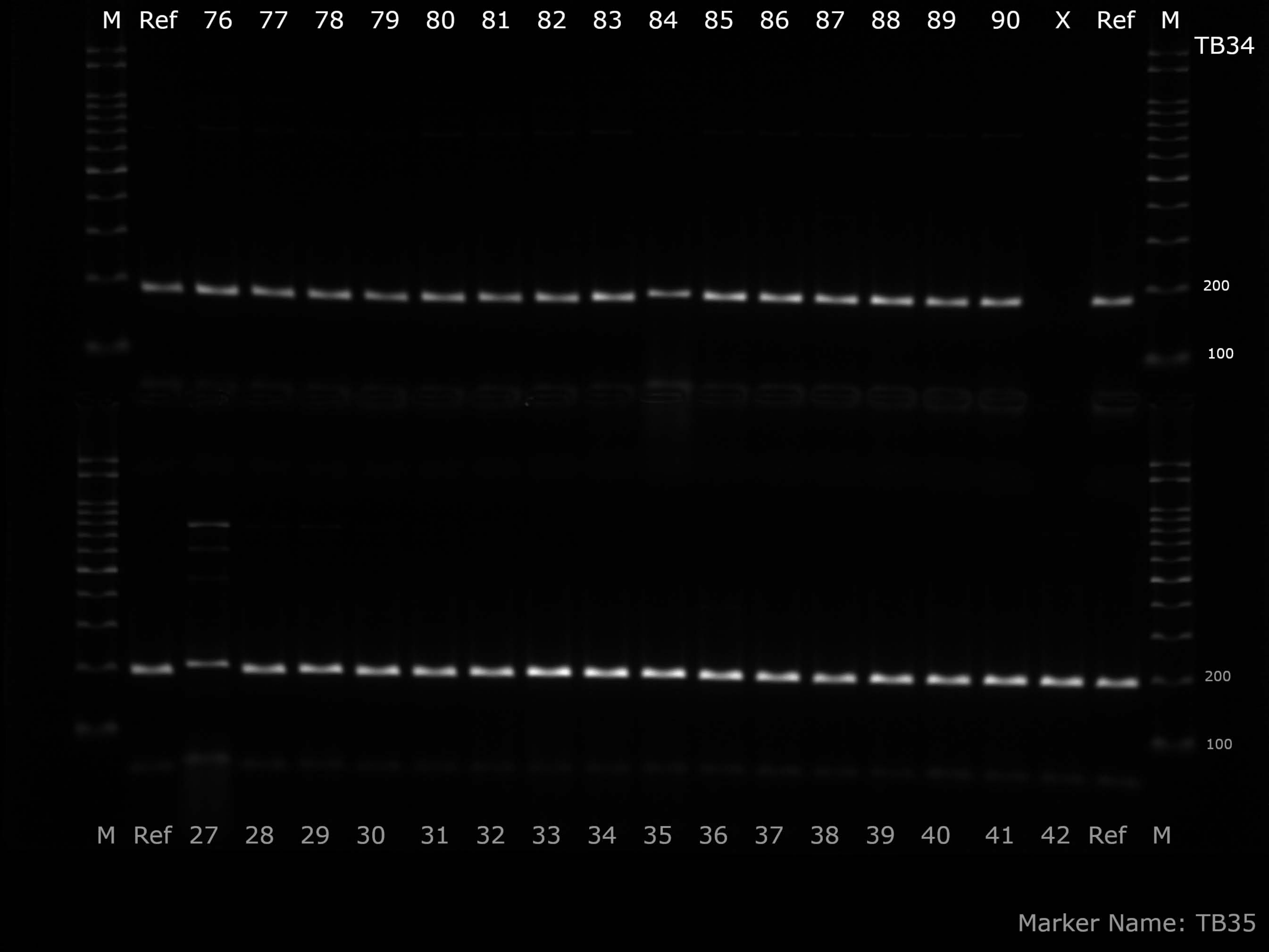
M Ref 76 77 78 79 80 81 82 83 84 85 86 87 88 89 90 X Ref M
TB34
200
100
200
100
M Ref 27 28 29 30 31 32 33 34 35 36 37 38 39 40 41 42 Ref M
Marker Name: TB35

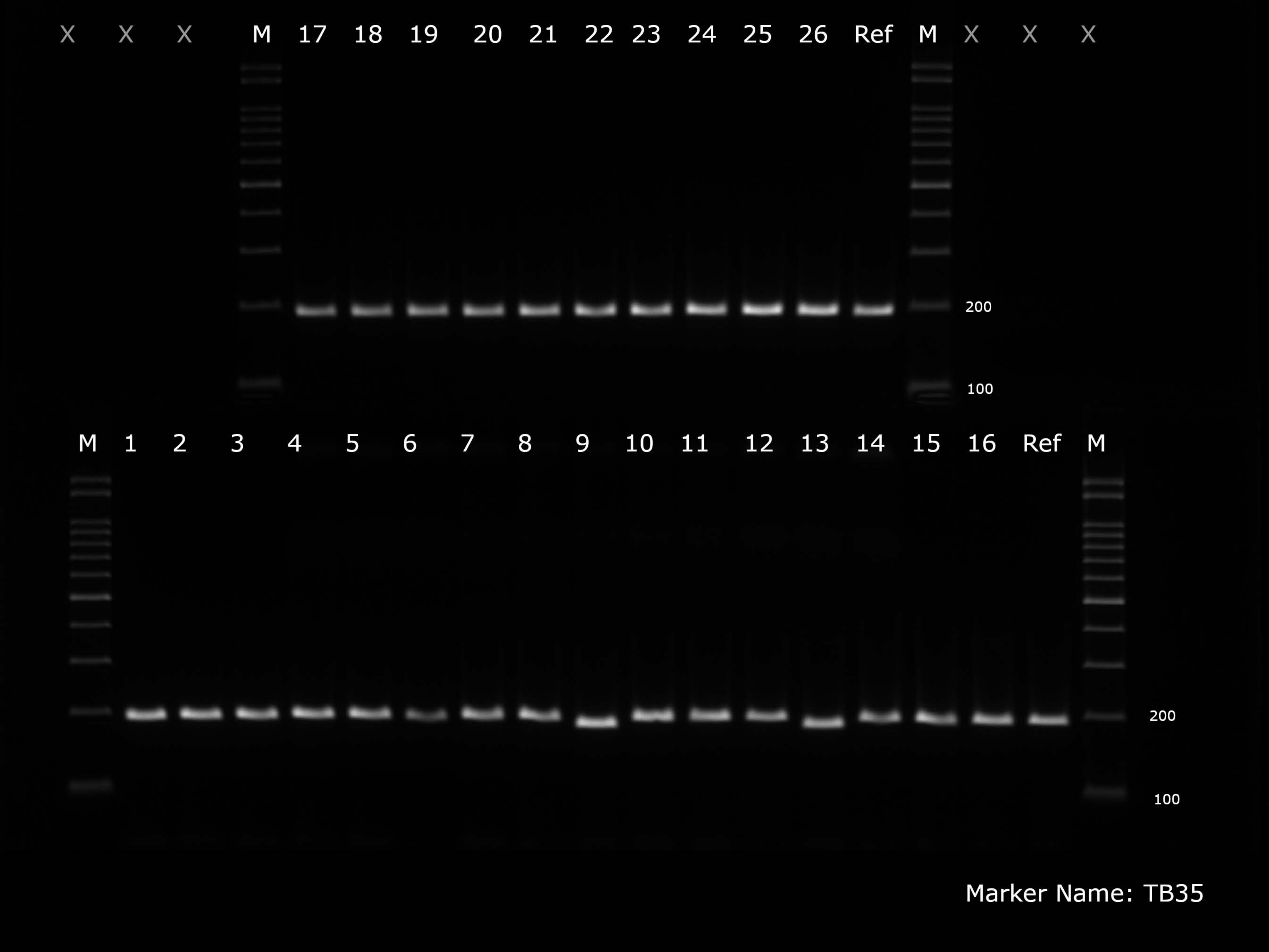
X X X M 17 18 19 20 21 22 23 24 25 26 Ref M X X X
200
100
M 1 2 3 4 5 6 7 8 9 10 11 12 13 14 15 16 Ref M
200
100
Marker Name: TB35

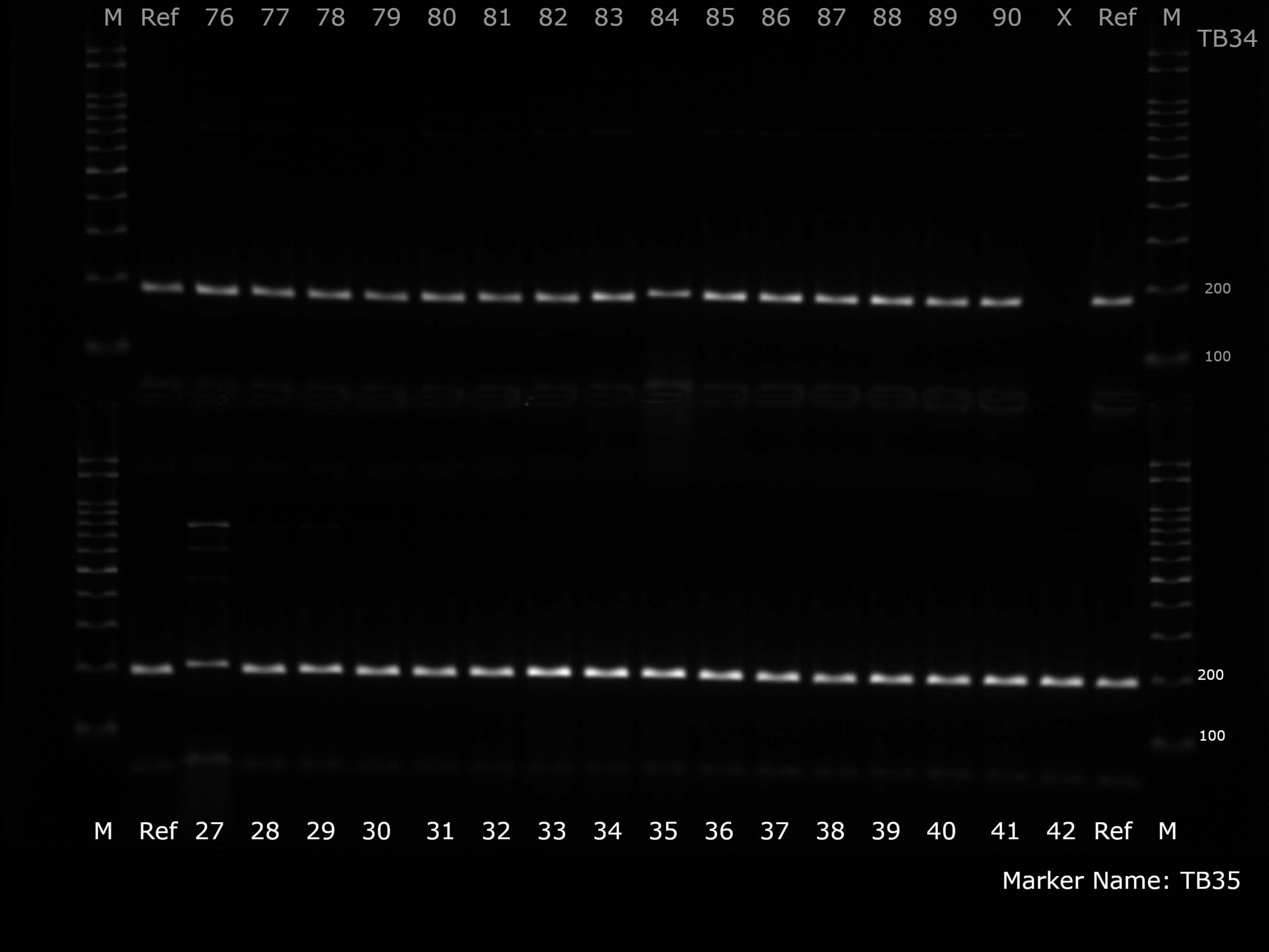
M Ref 76 77 78 79 80 81 82 83 84 85 86 87 88 89 90 X Ref M
TB34
200
100
200
100
M Ref 27 28 29 30 31 32 33 34 35 36 37 38 39 40 41 42 Ref M
Marker Name: TB35

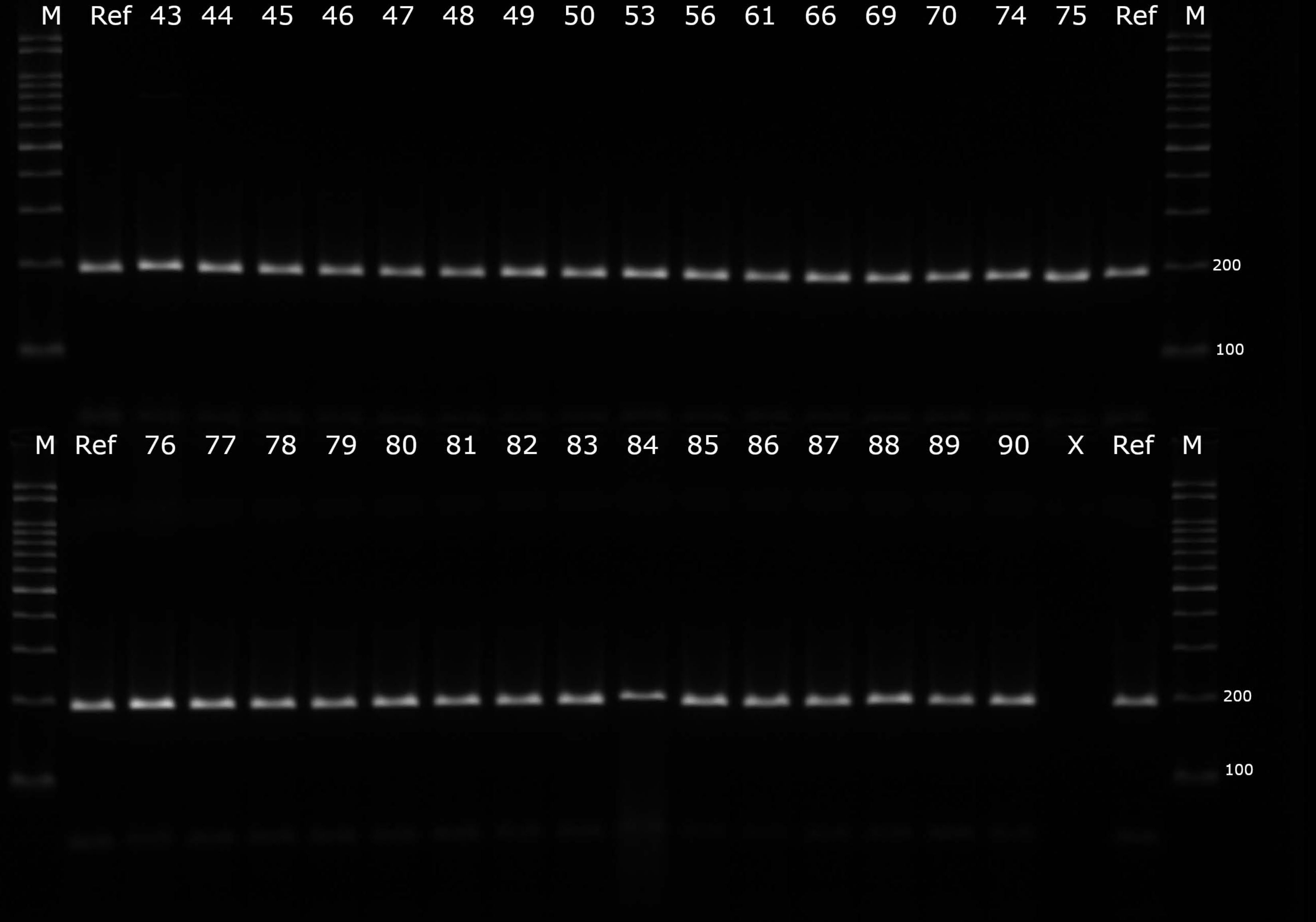

Marker Name: TB35

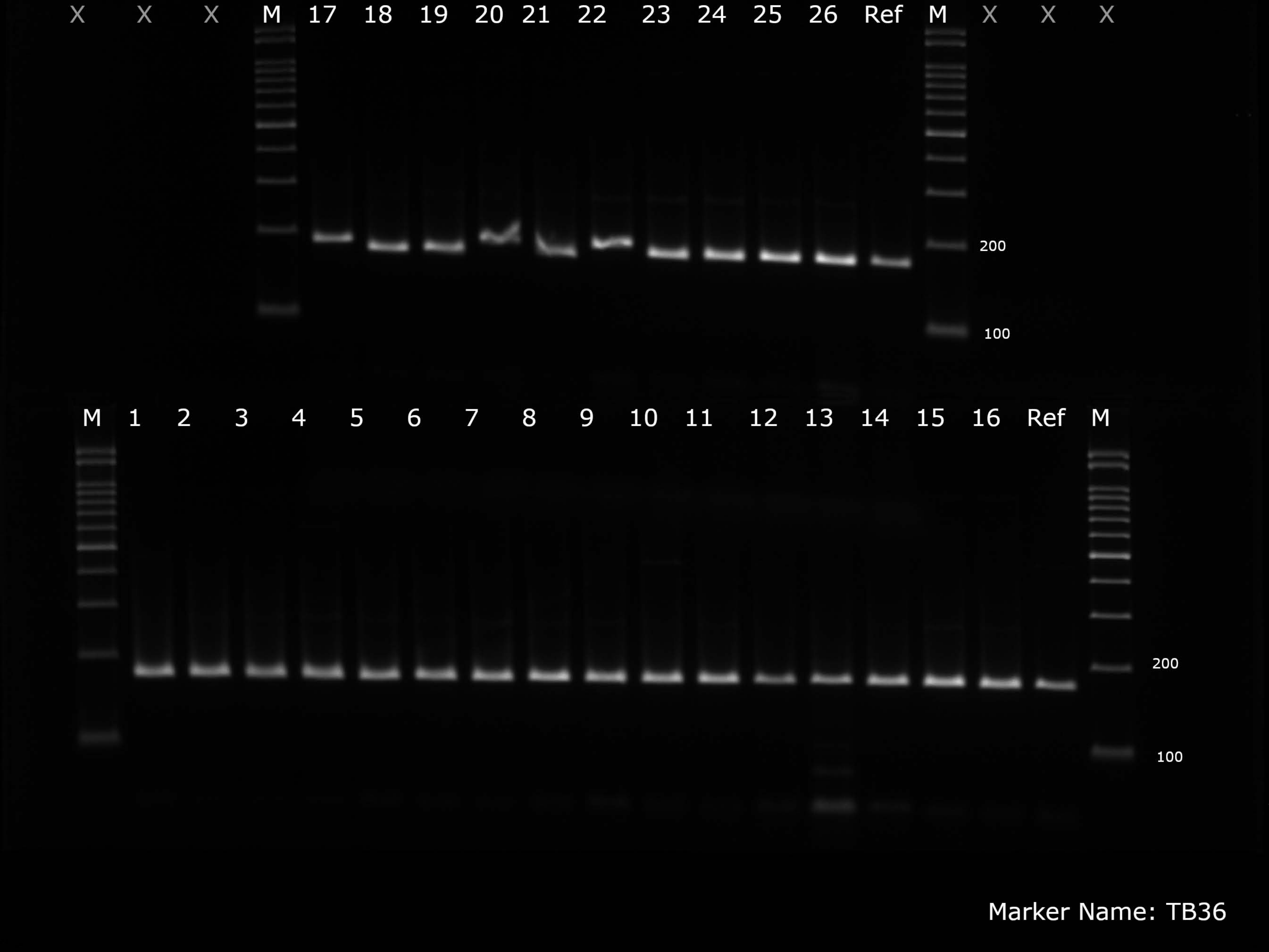
X X X M 17 18 19 20 21 22 23 24 25 26 Ref M X X X
200
100
M 1 2 3 4 5 6 7 8 9 10 11 12 13 14 15 16 Ref M
200
100
Marker Name: TB36

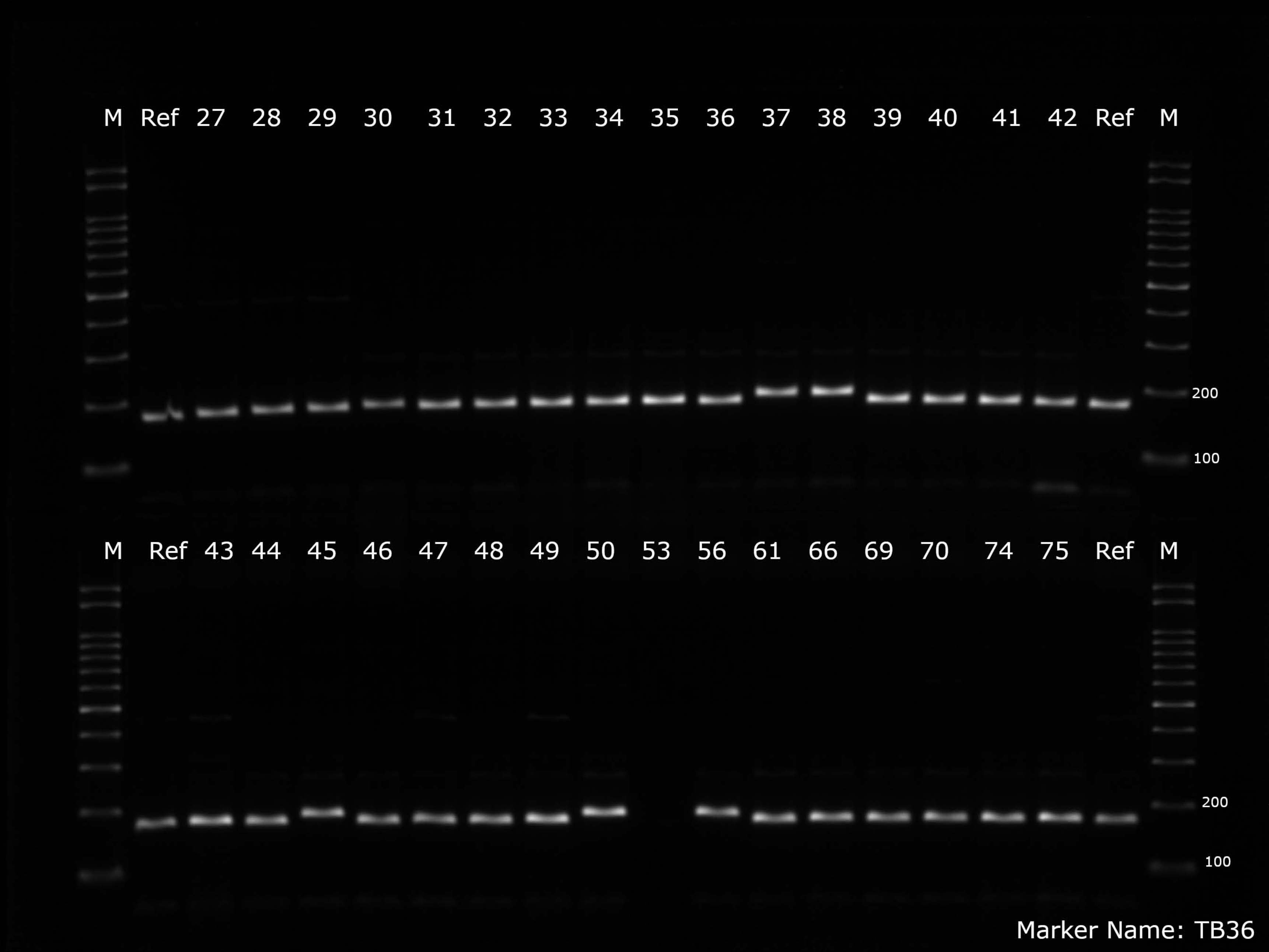
M Ref 27 28 29 30 31 32 33 34 35 36 37 38 39 40 41 42 Ref M
200
100
M Ref 43 44 45 46 47 48 49 50 53 56 61 66 69 70 74 75 Ref M
200
100
Marker Name: TB36

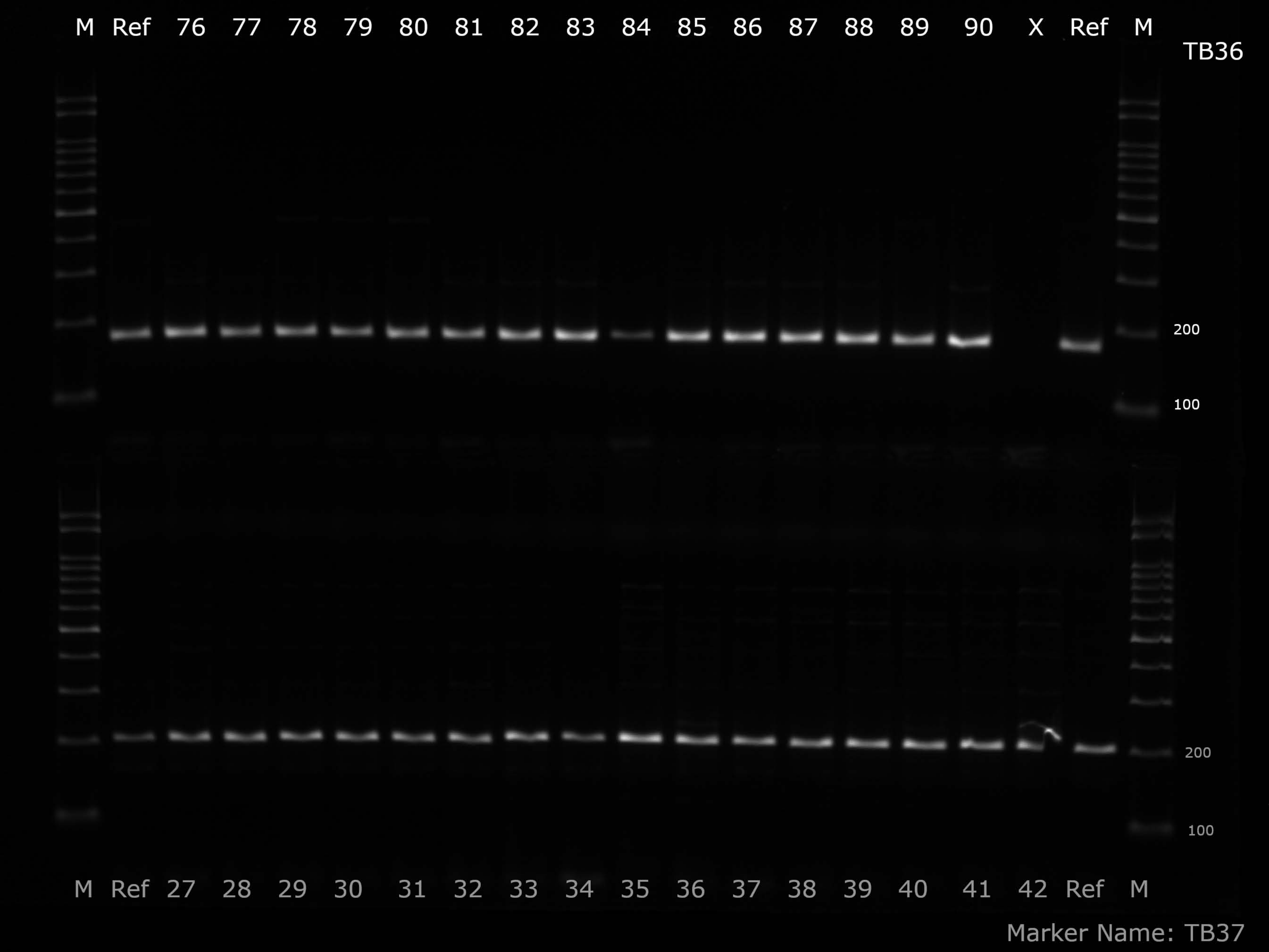
M Ref 76 77 78 79 80 81 82 83 84 85 86 87 88 89 90 X Ref M
TB36
200
100
200
100
M Ref 27 28 29 30 31 32 33 34 35 36 37 38 39 40 41 42 Ref M
Marker Name: TB37

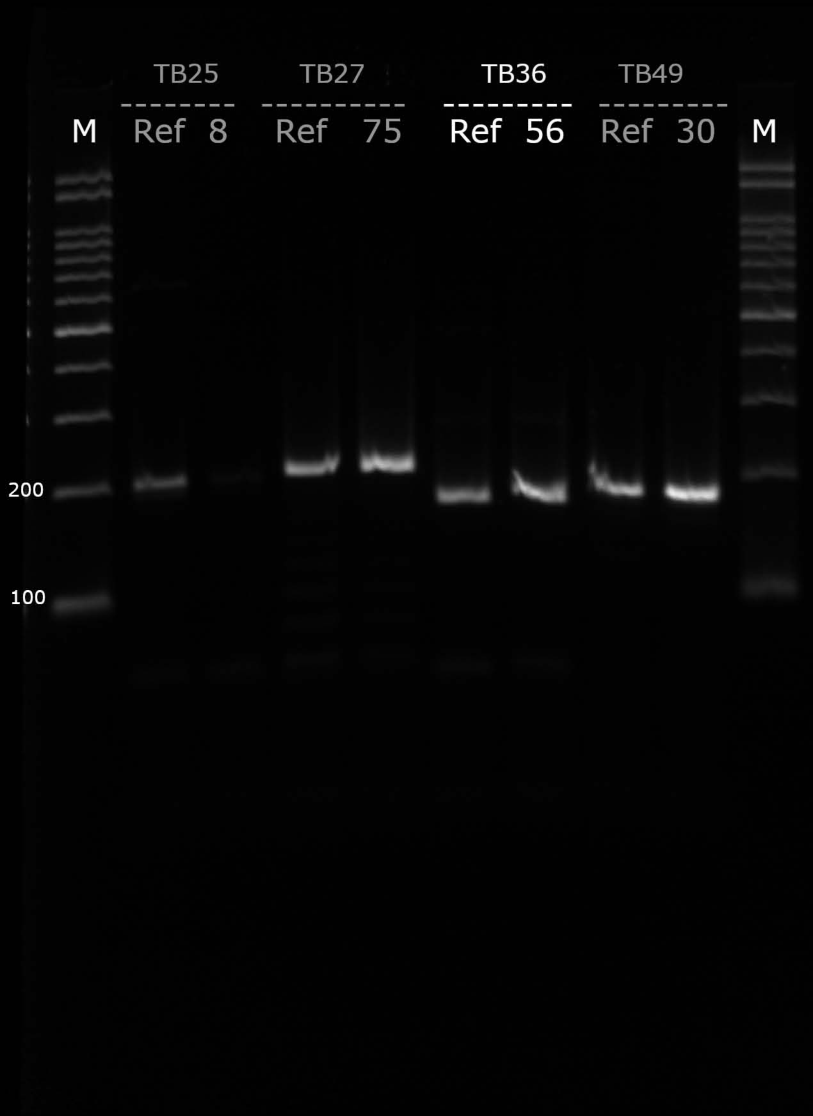
TB25
TB27
TB36
TB49
M
Ref
8
Ref
75
Ref
56
Ref
30
M
200
100

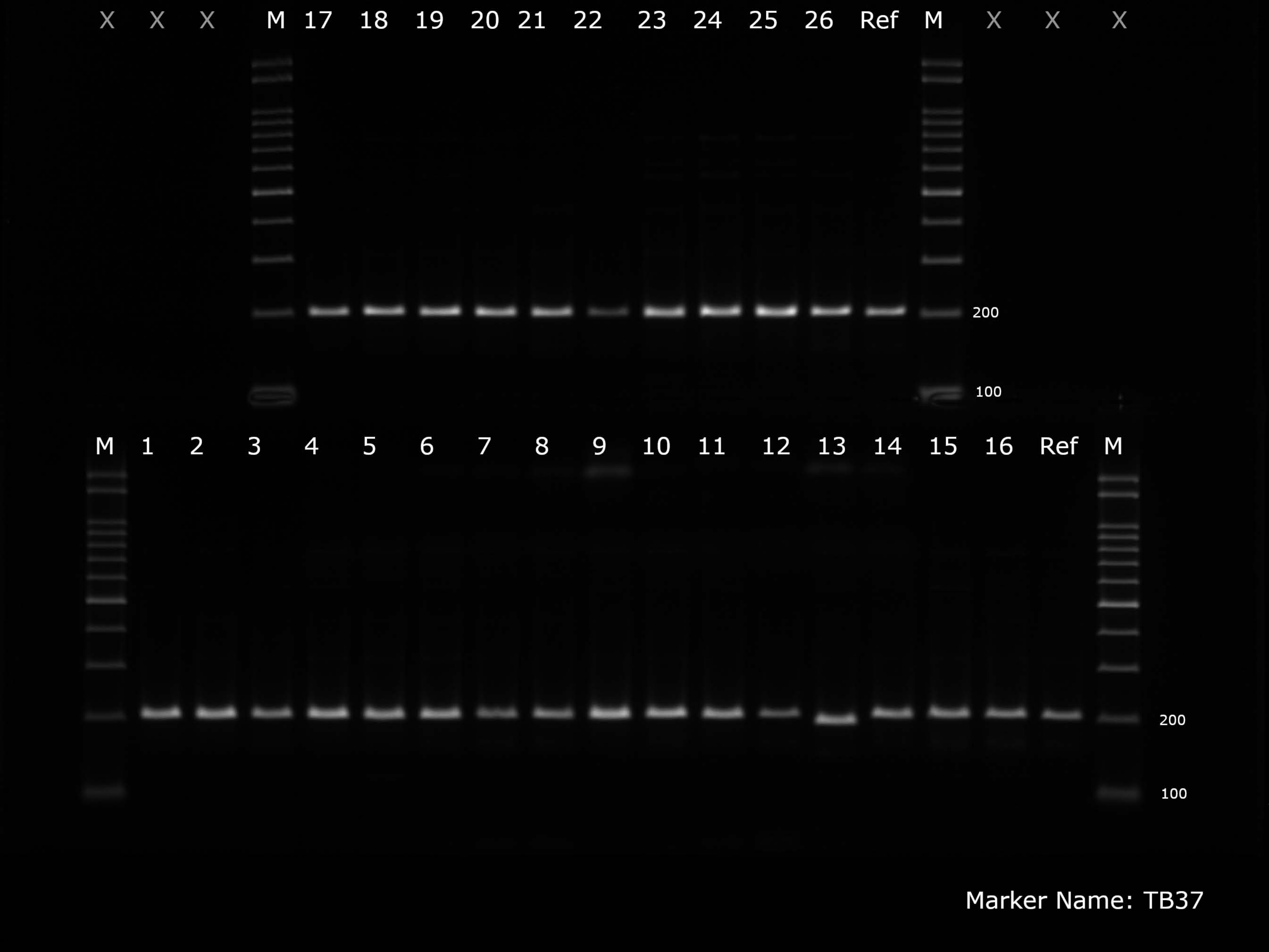
X X X M 17 18 19 20 21 22 23 24 25 26 Ref M X X X
200
100
M 1 2 3 4 5 6 7 8 9 10 11 12 13 14 15 16 Ref M
200
100
Marker Name: TB37

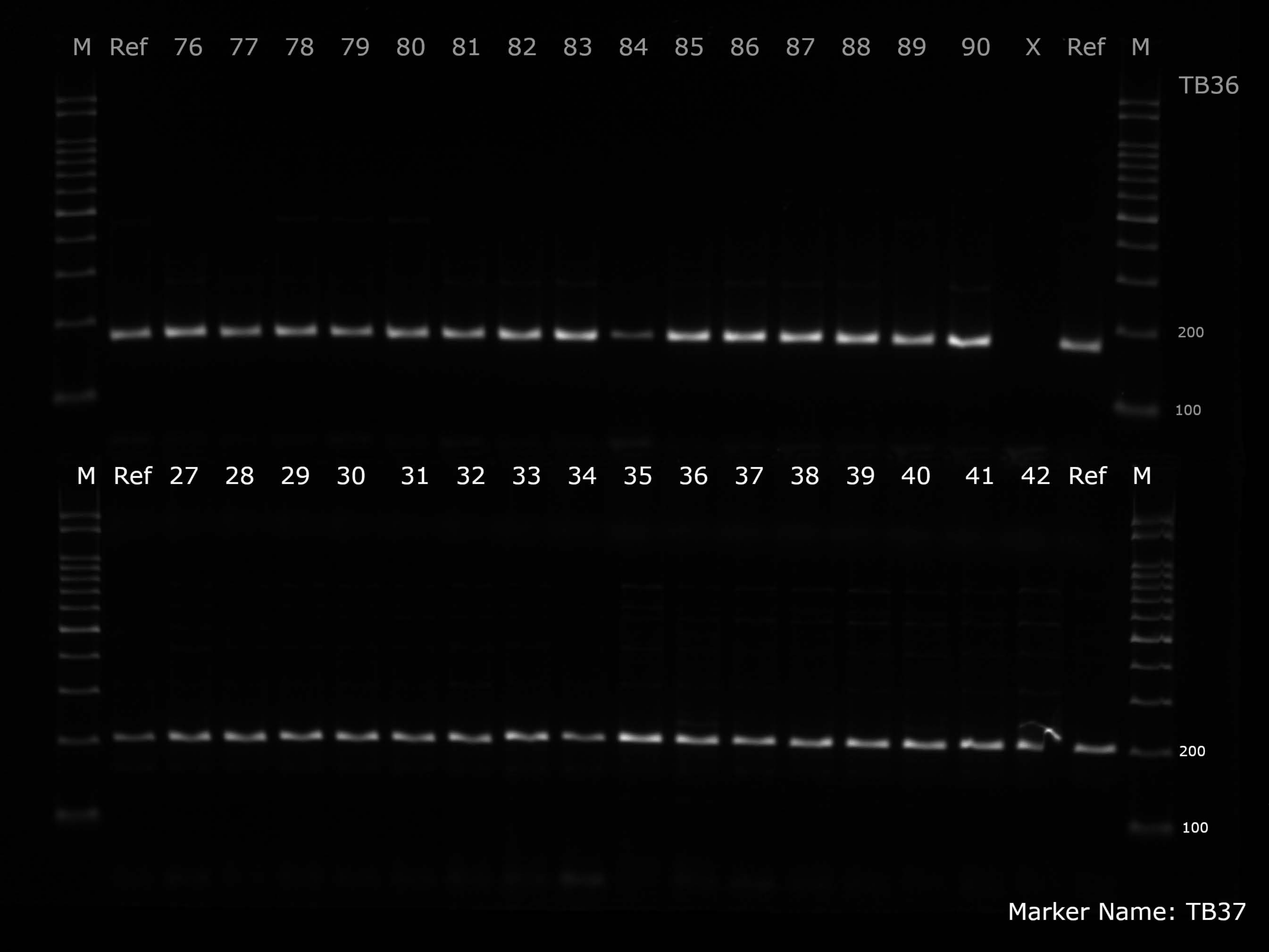
M Ref 76 77 78 79 80 81 82 83 84 85 86 87 88 89 90 X Ref M
TB36
200
100
M Ref 27 28 29 30 31 32 33 34 35 36 37 38 39 40 41 42 Ref M
200
100
Marker Name: TB37

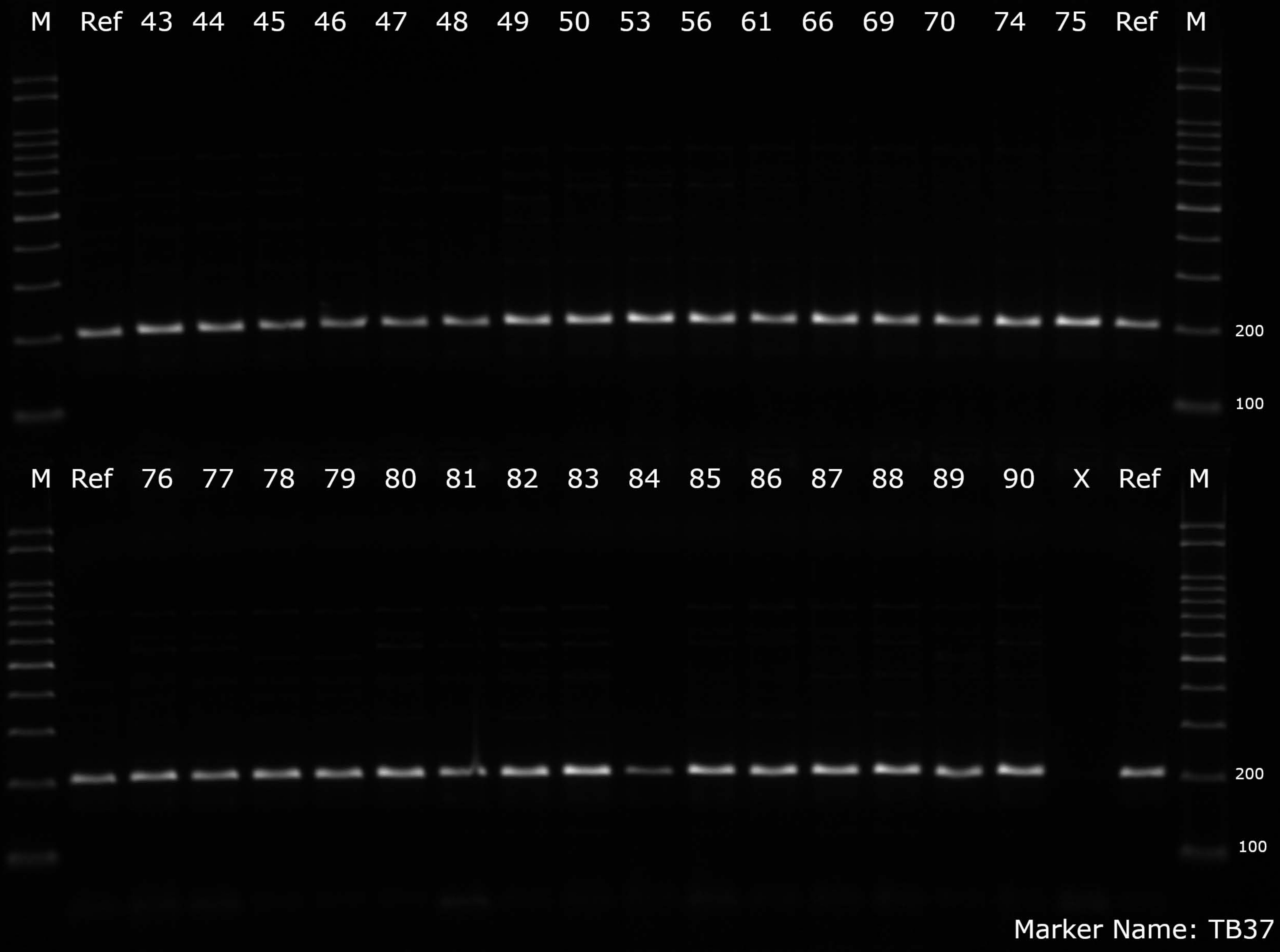
M Ref 43 44 45 46 47 48 49 50 53 56 61 66 69 70 74 75 Ref M
200
100
M Ref 76 77 78 79 80 81 82 83 84 85 86 87 88 89 90 X Ref M
200
100
Marker Name: TB37

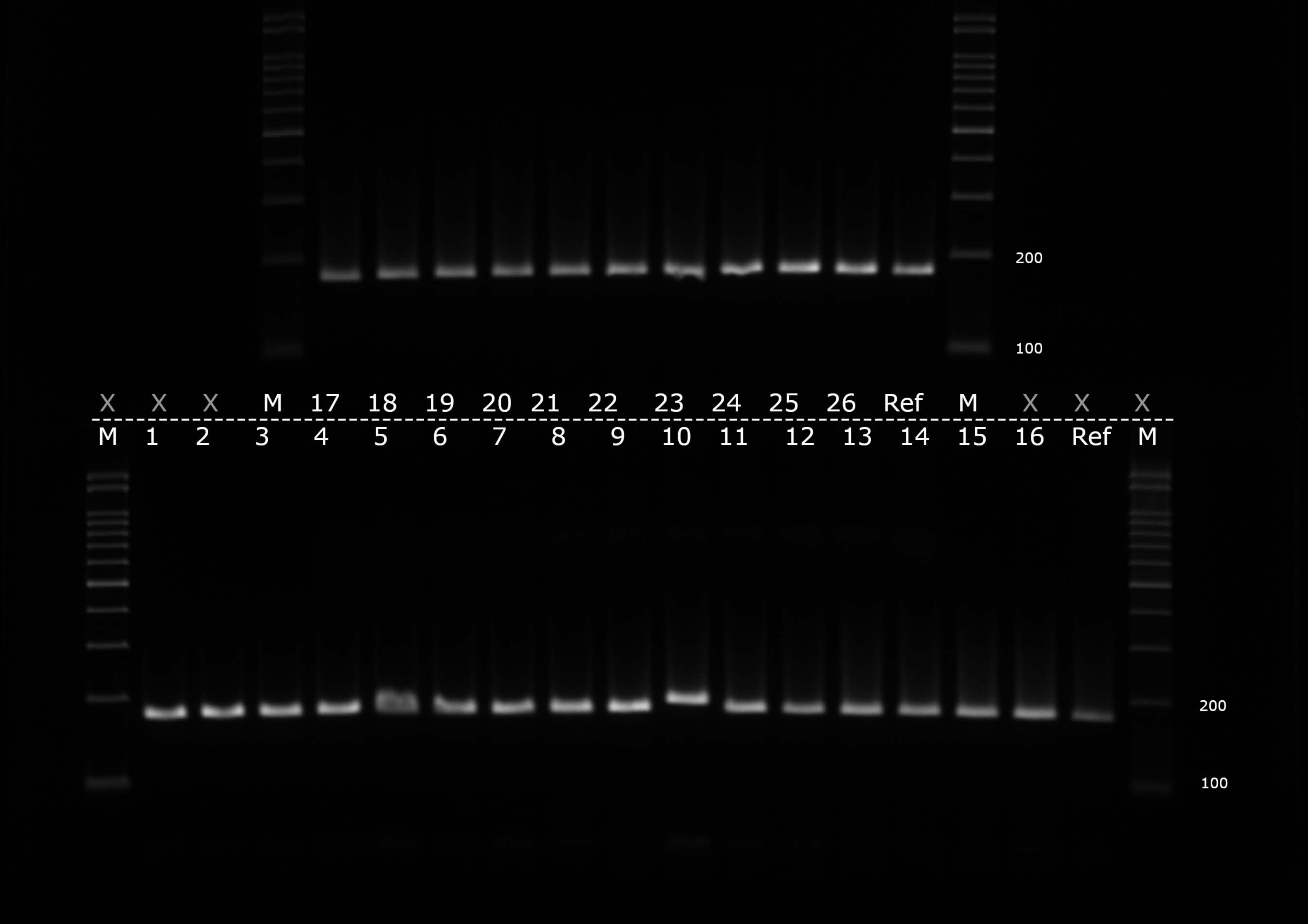

Marker Name: TB38

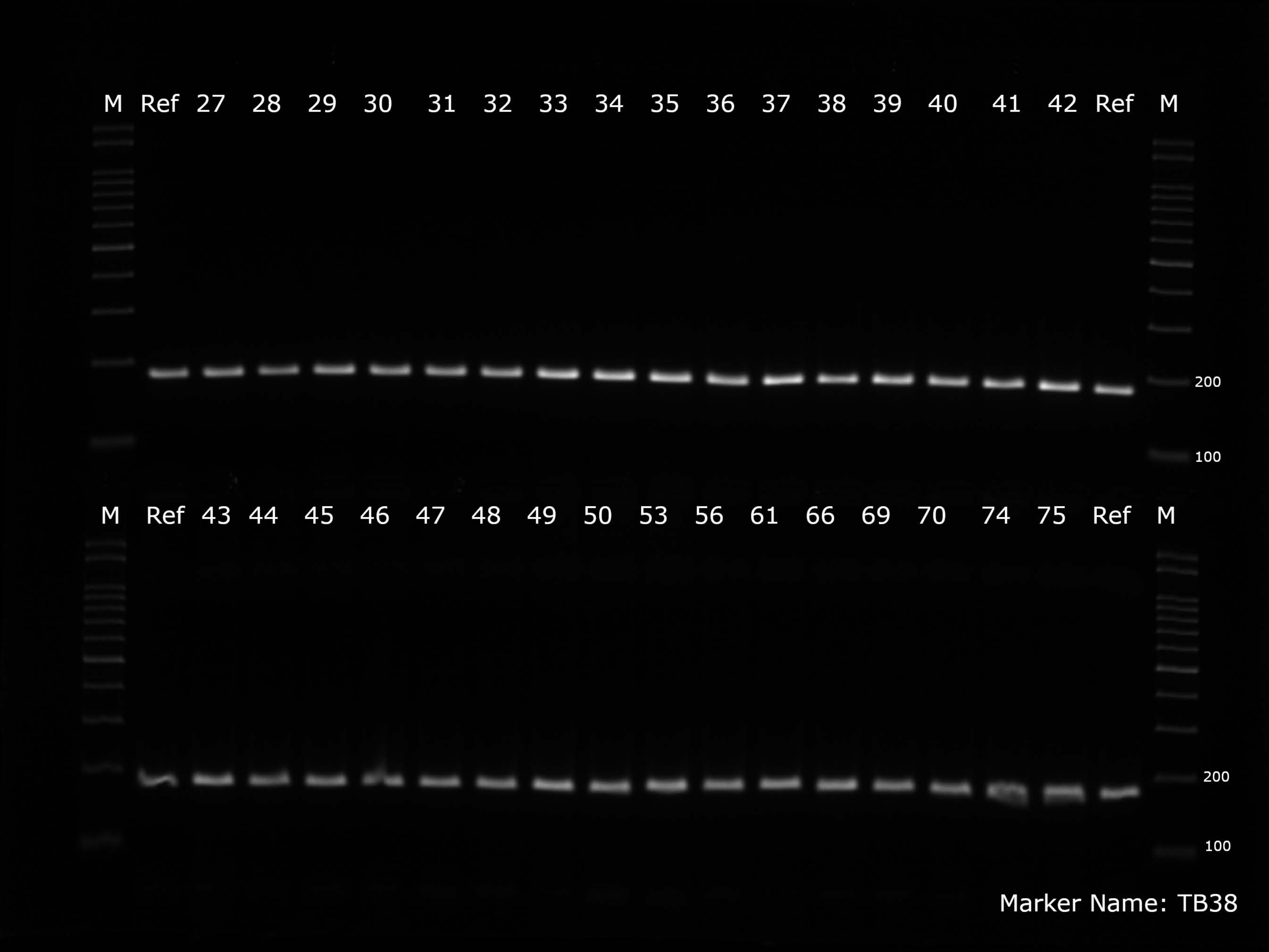
M Ref 27 28 29 30 31 32 33 34 35 36 37 38 39 40 41 42 Ref M
200
100
M Ref 43 44 45 46 47 48 49 50 53 56 61 66 69 70 74 75 Ref M
200
100
Marker Name: TB38

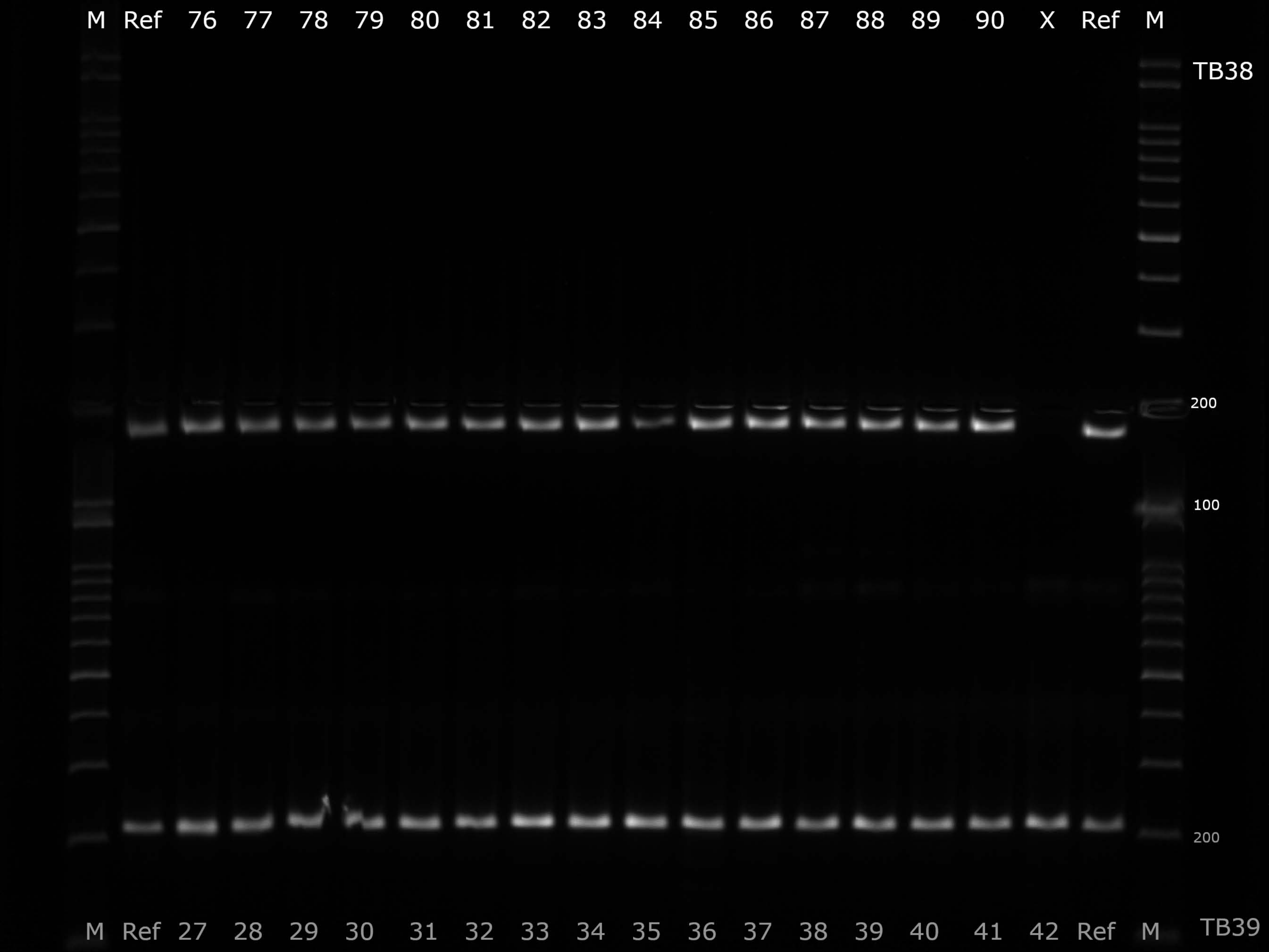

M Ref 76 77 78 79 80 81 82 83 84 85 86 87 88 89 90 X Ref M
TB38
200
100
200
M Ref 27 28 29 30 31 32 33 34 35 36 37 38 39 40 41 42 Ref M
TB39

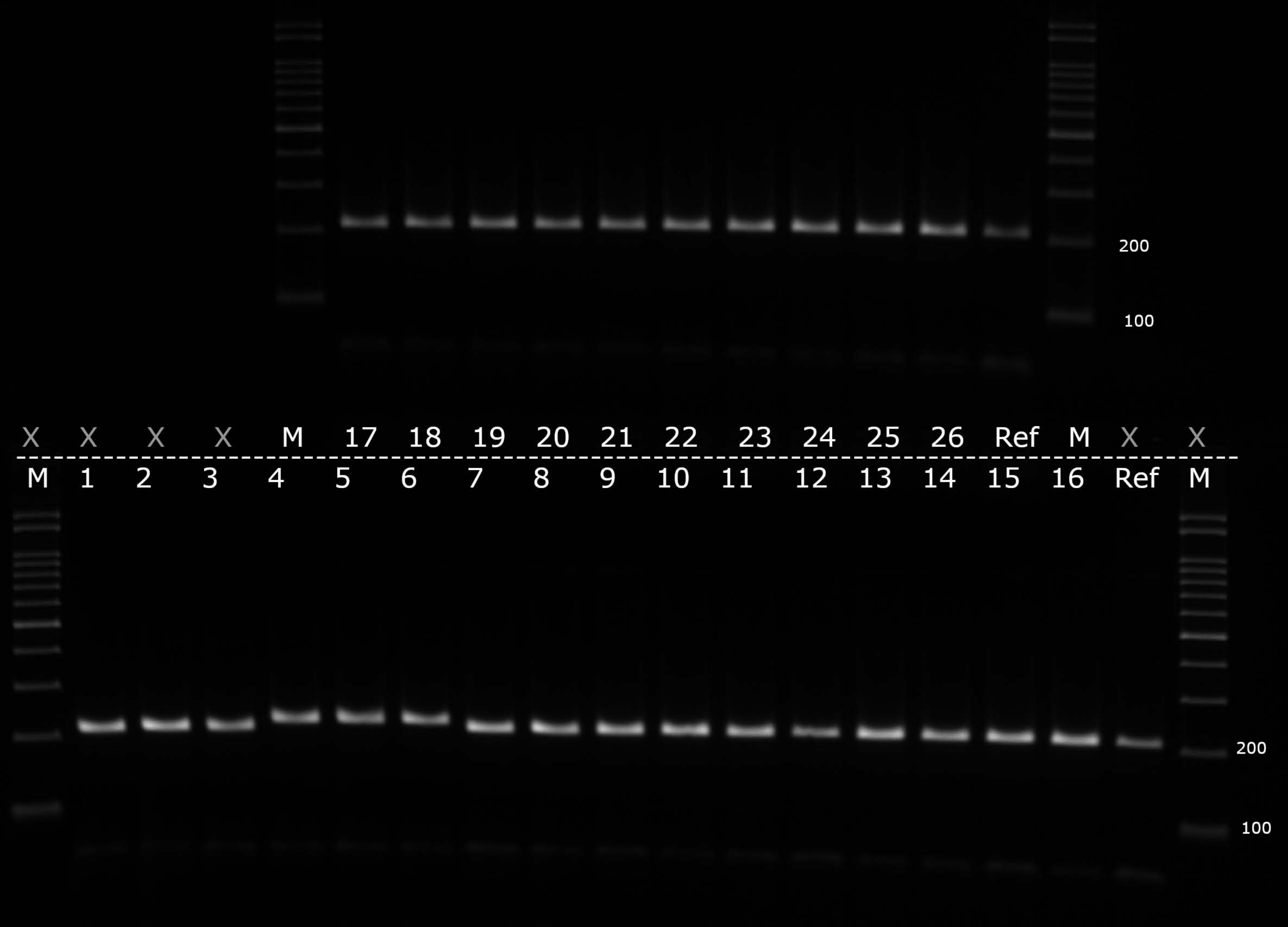

Marker Name: TB39

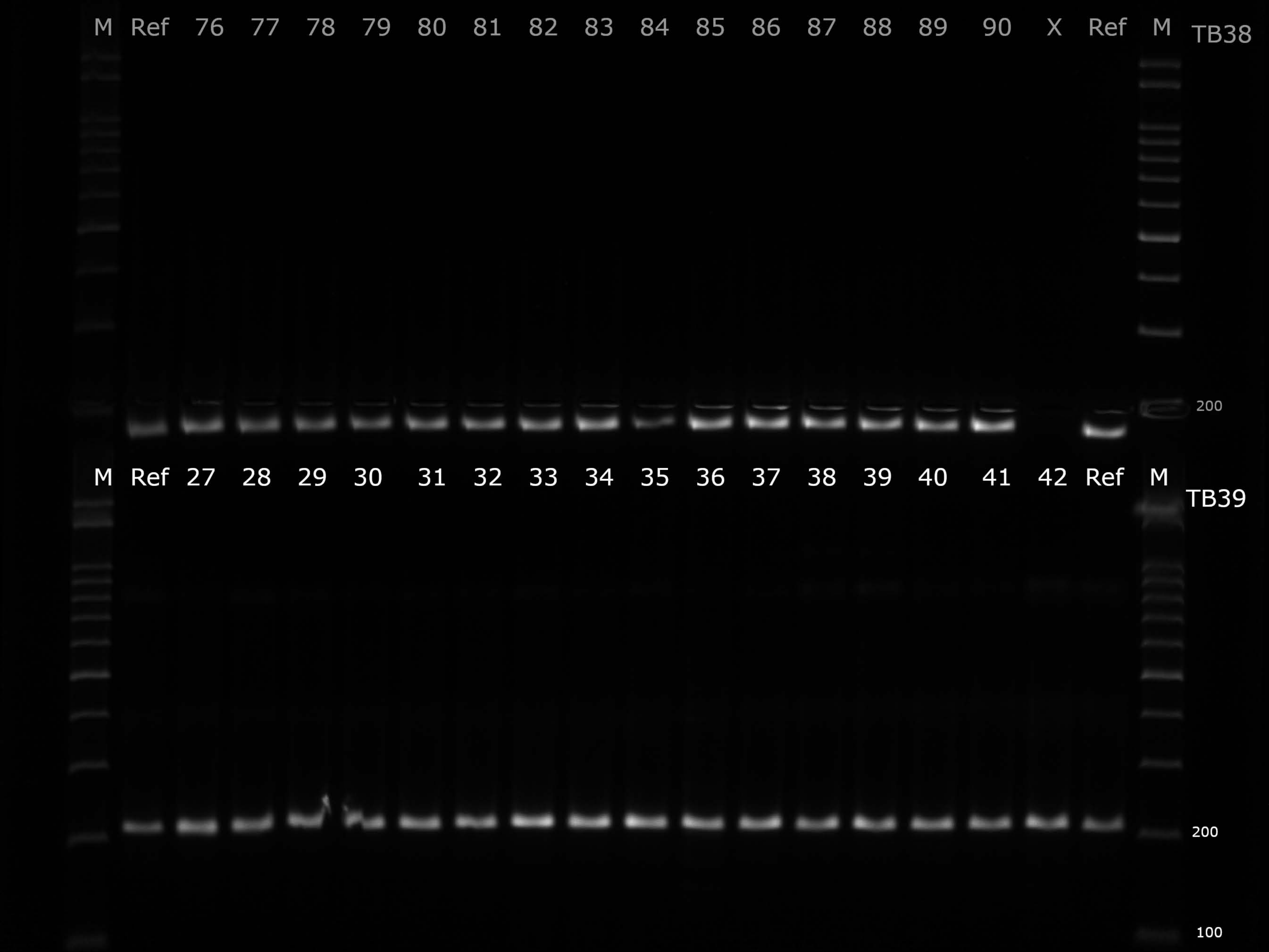
M Ref 76 77 78 79 80 81 82 83 84 85 86 87 88 89 90 X Ref M
TB38
200
M Ref 27 28 29 30 31 32 33 34 35 36 37 38 39 40 41 42 Ref M
TB39
200
100

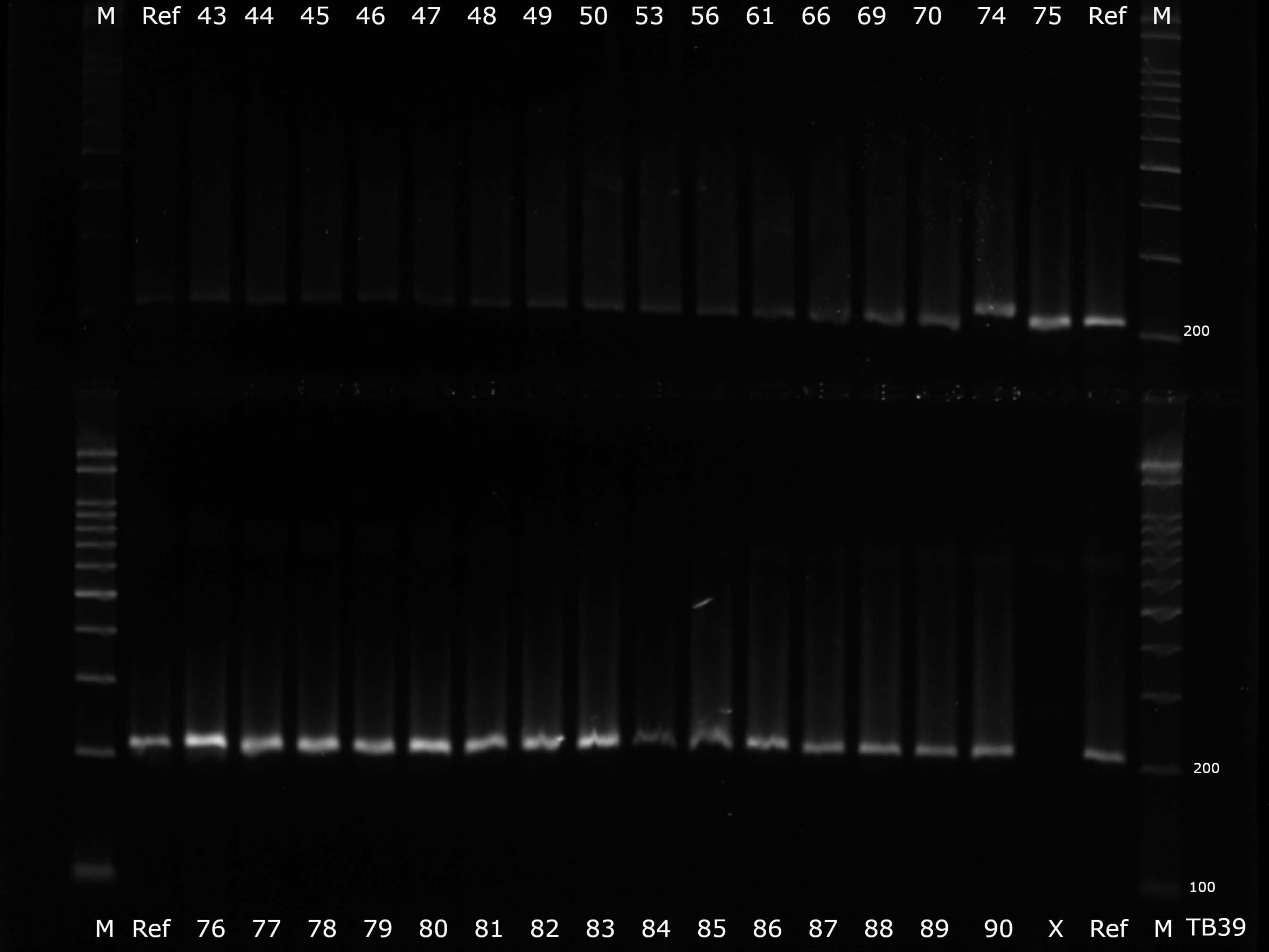
M Ref 43 44 45 46 47 48 49 50 53 56 61 66 69 70 74 75 Ref M
200
200
100
M Ref 76 77 78 79 80 81 82 83 84 85 86 87 88 89 90 X Ref M
TB39

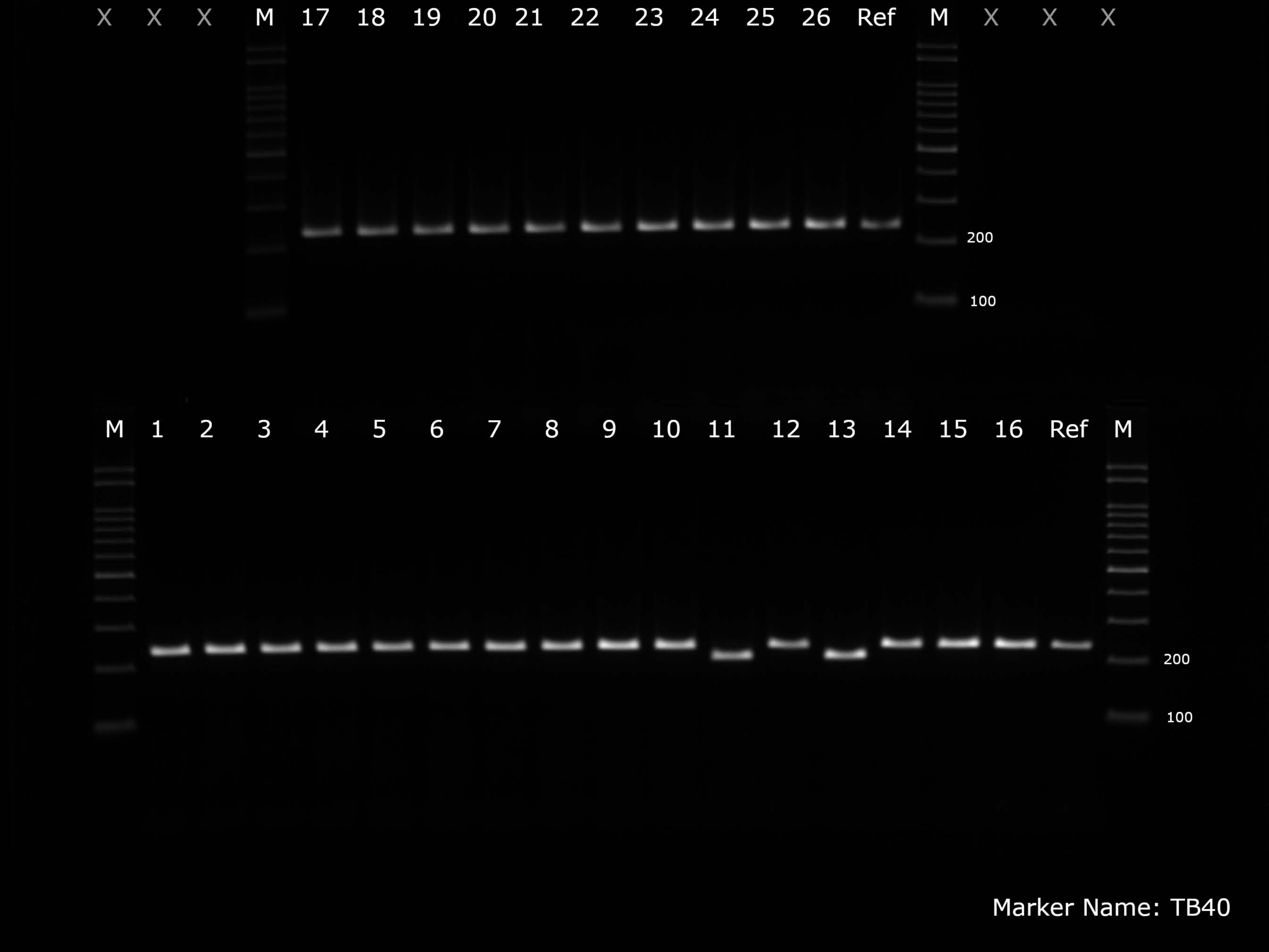
X X X M 17 18 19 20 21 22 23 24 25 26 Ref M X X X
200
100
M 1 2 3 4 5 6 7 8 9 10 11 12 13 14 15 16 Ref M
200
100
Marker Name: TB40

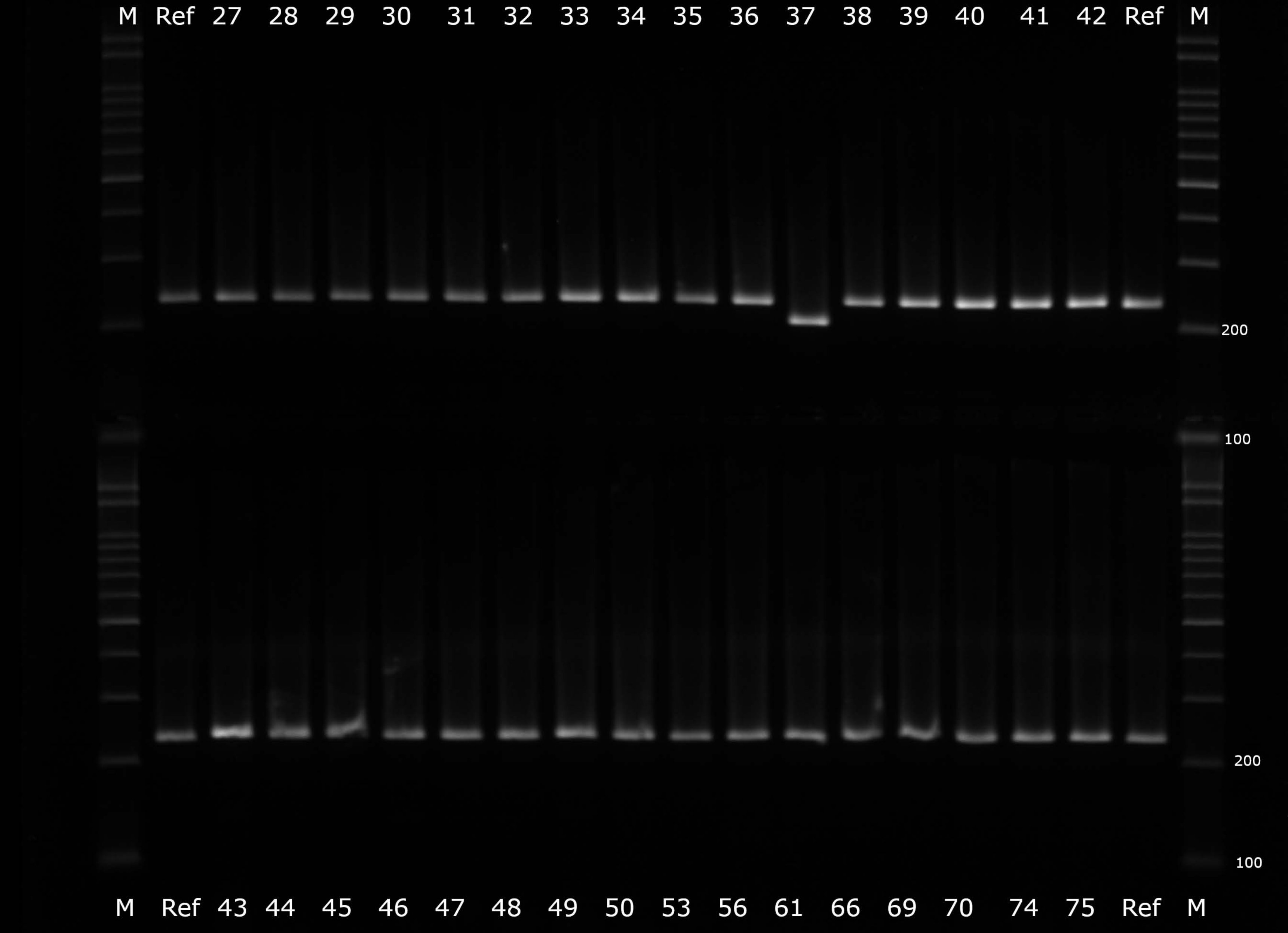

Marker Name: TB40

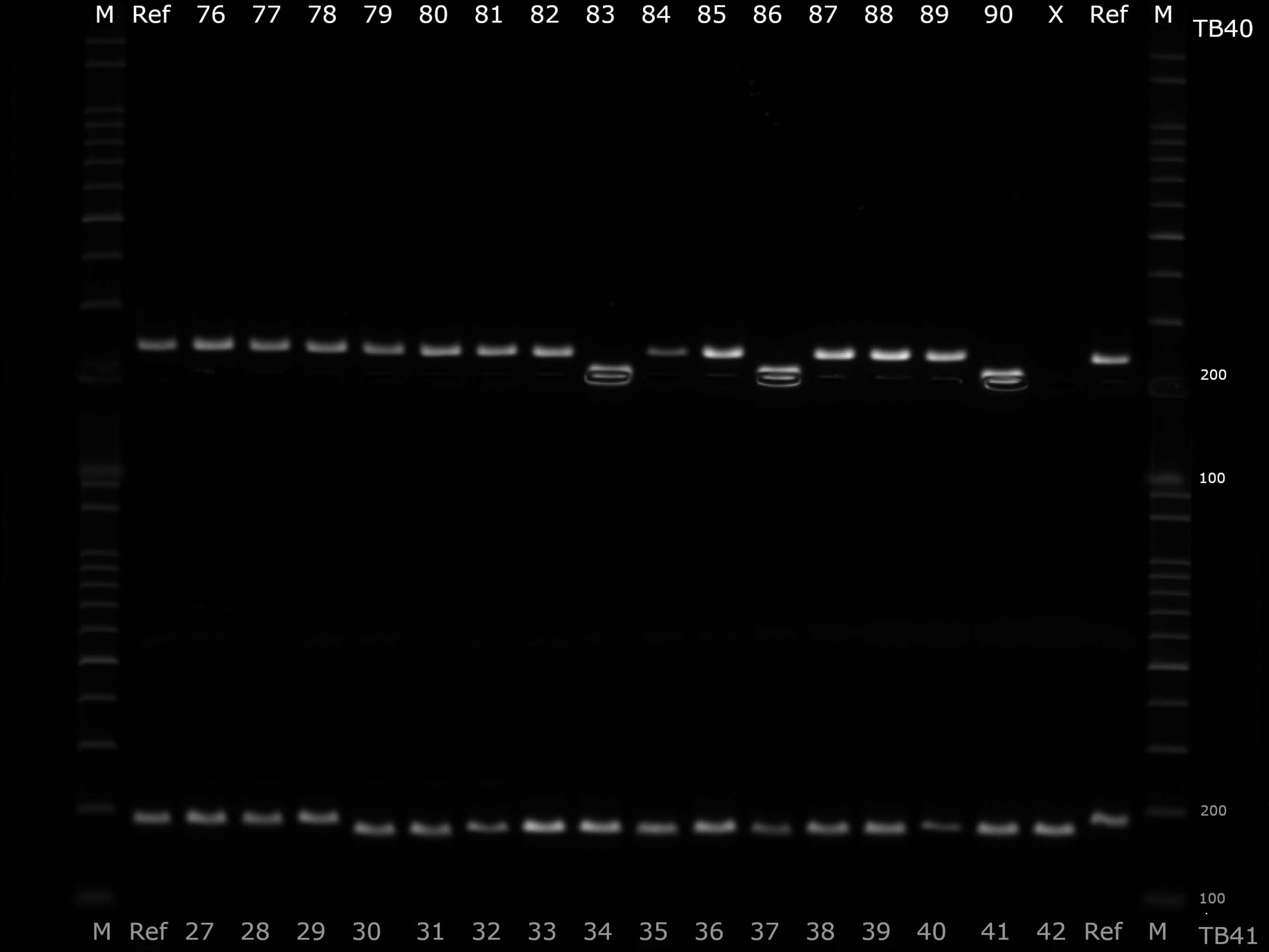

M Ref 76 77 78 79 80 81 82 83 84 85 86 87 88 89 90 X Ref M
TB40
200
100
200
100
M Ref 27 28 29 30 31 32 33 34 35 36 37 38 39 40 41 42 Ref M
TB41

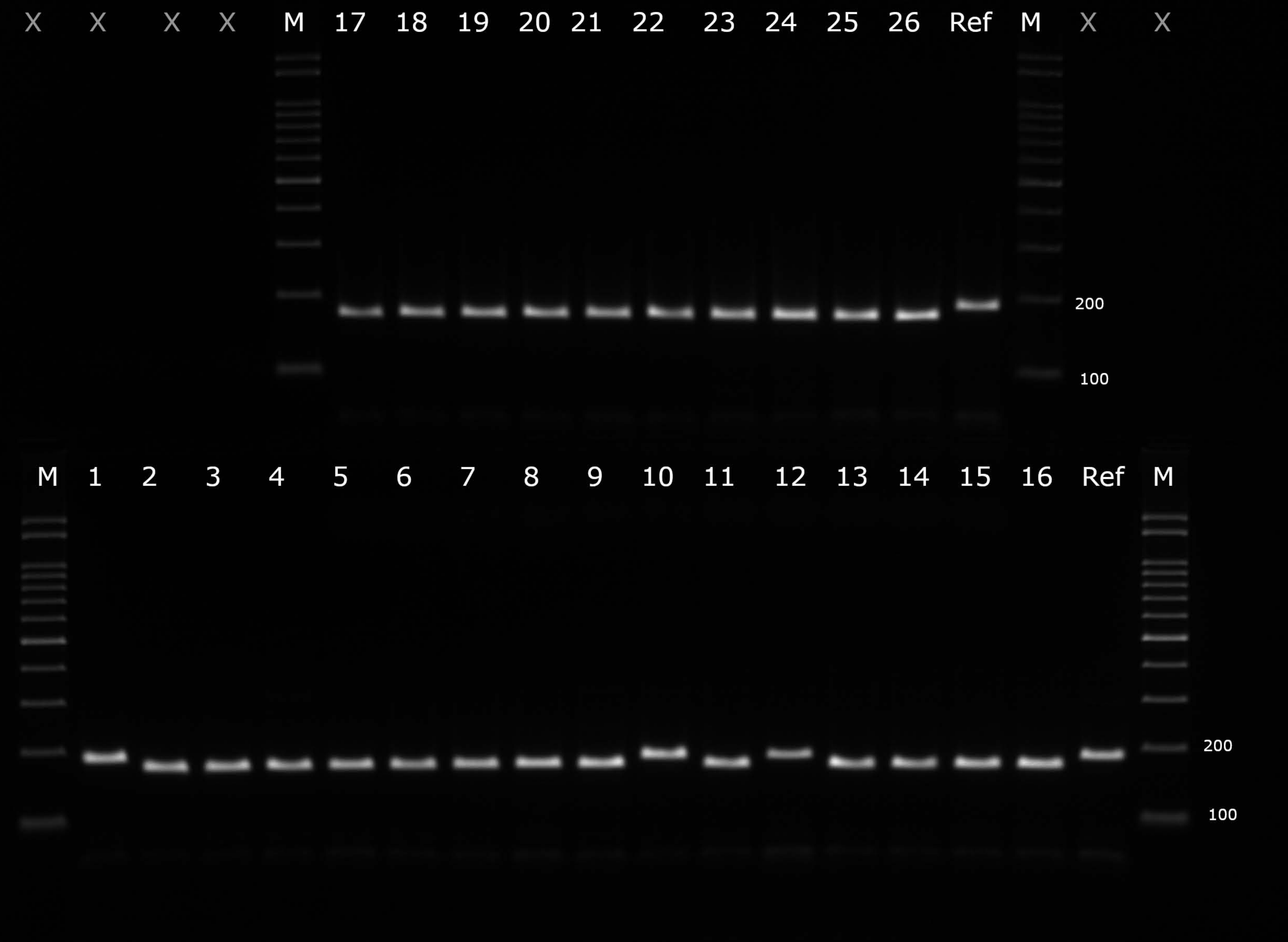

Marker Name: TB41

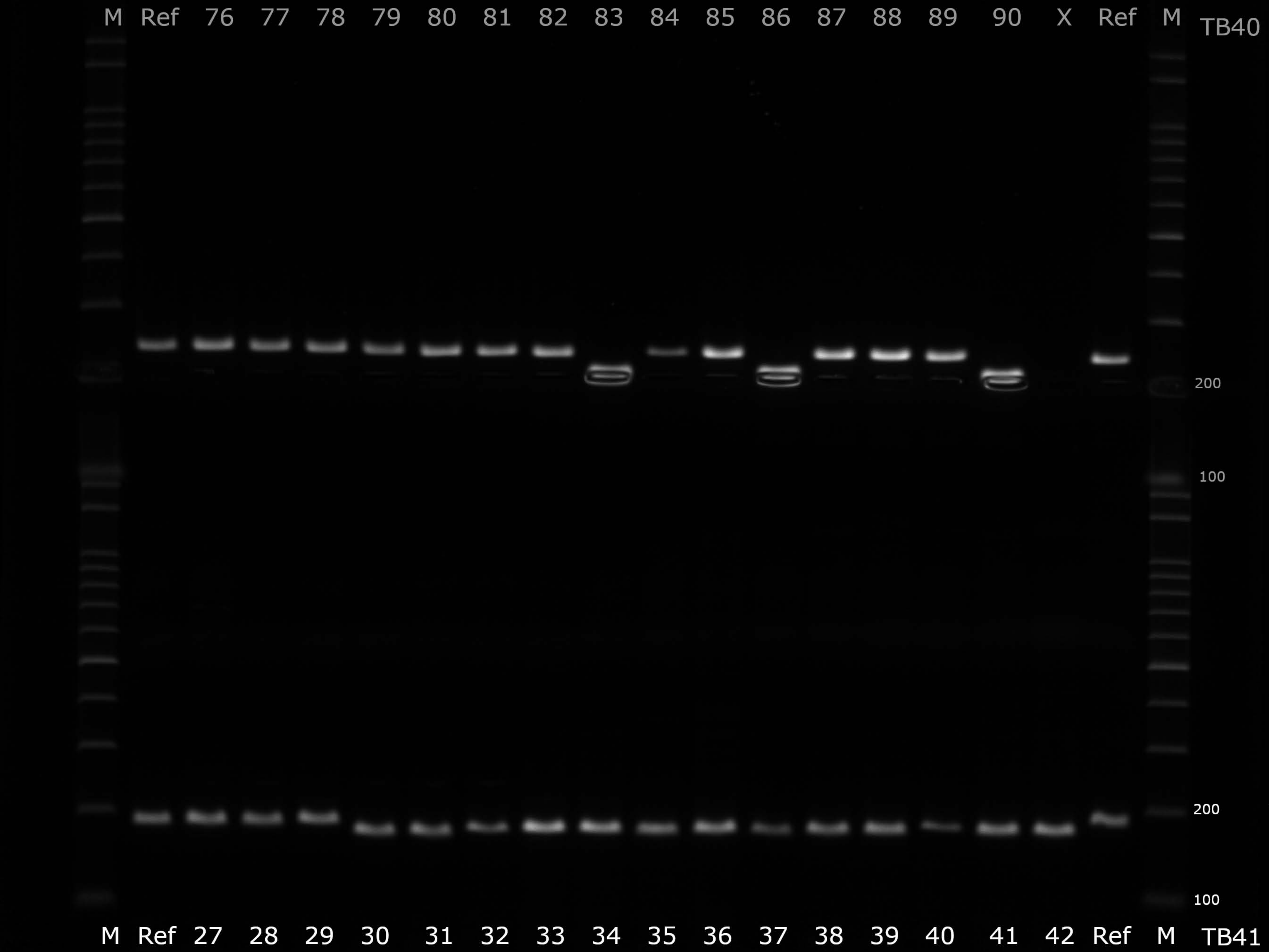

M Ref 76 77 78 79 80 81 82 83 84 85 86 87 88 89 90 X Ref M
TB40
200
100
200
100
M Ref 27 28 29 30 31 32 33 34 35 36 37 38 39 40 41 42 Ref M
TB41

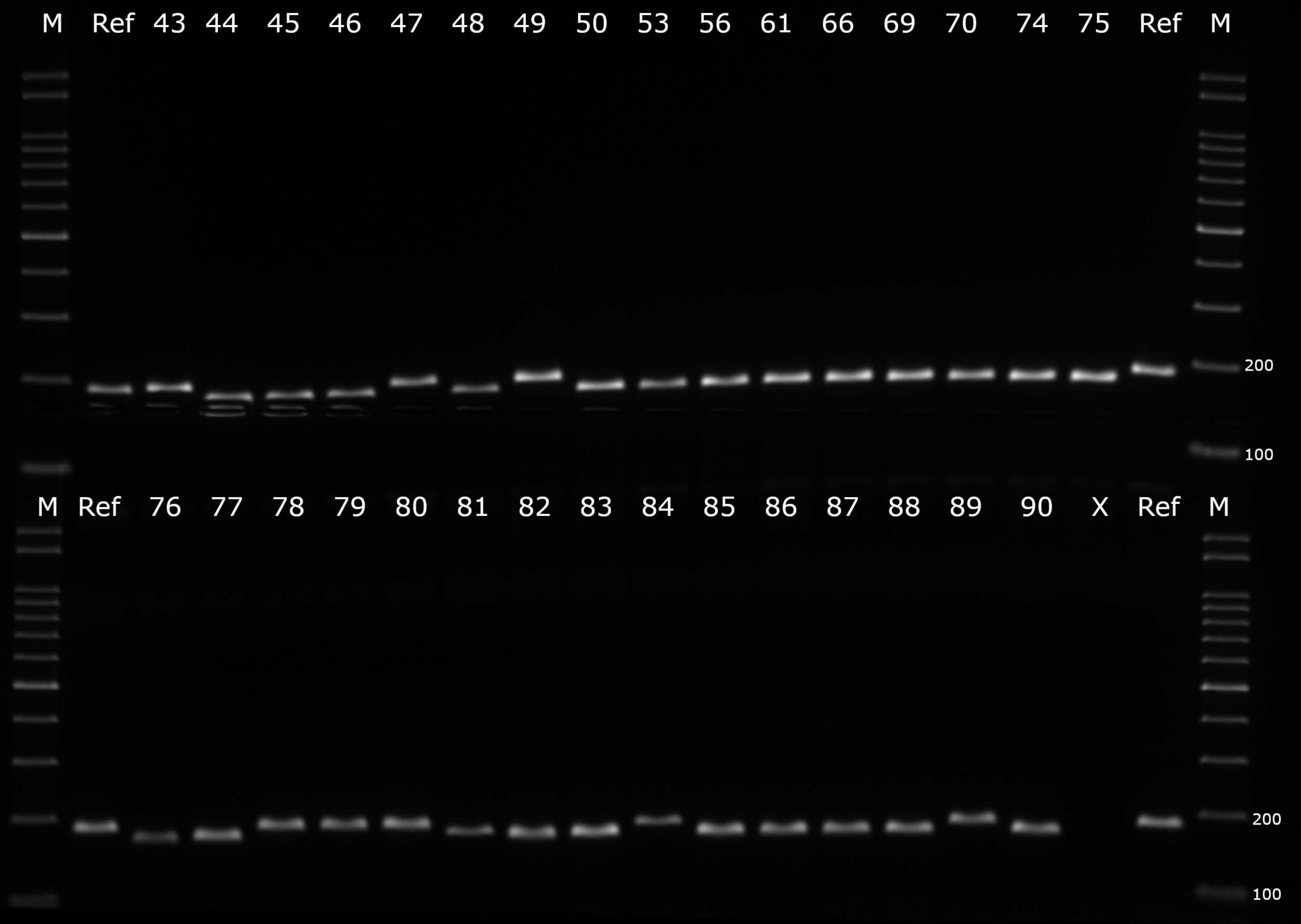

Marker Name: TB41

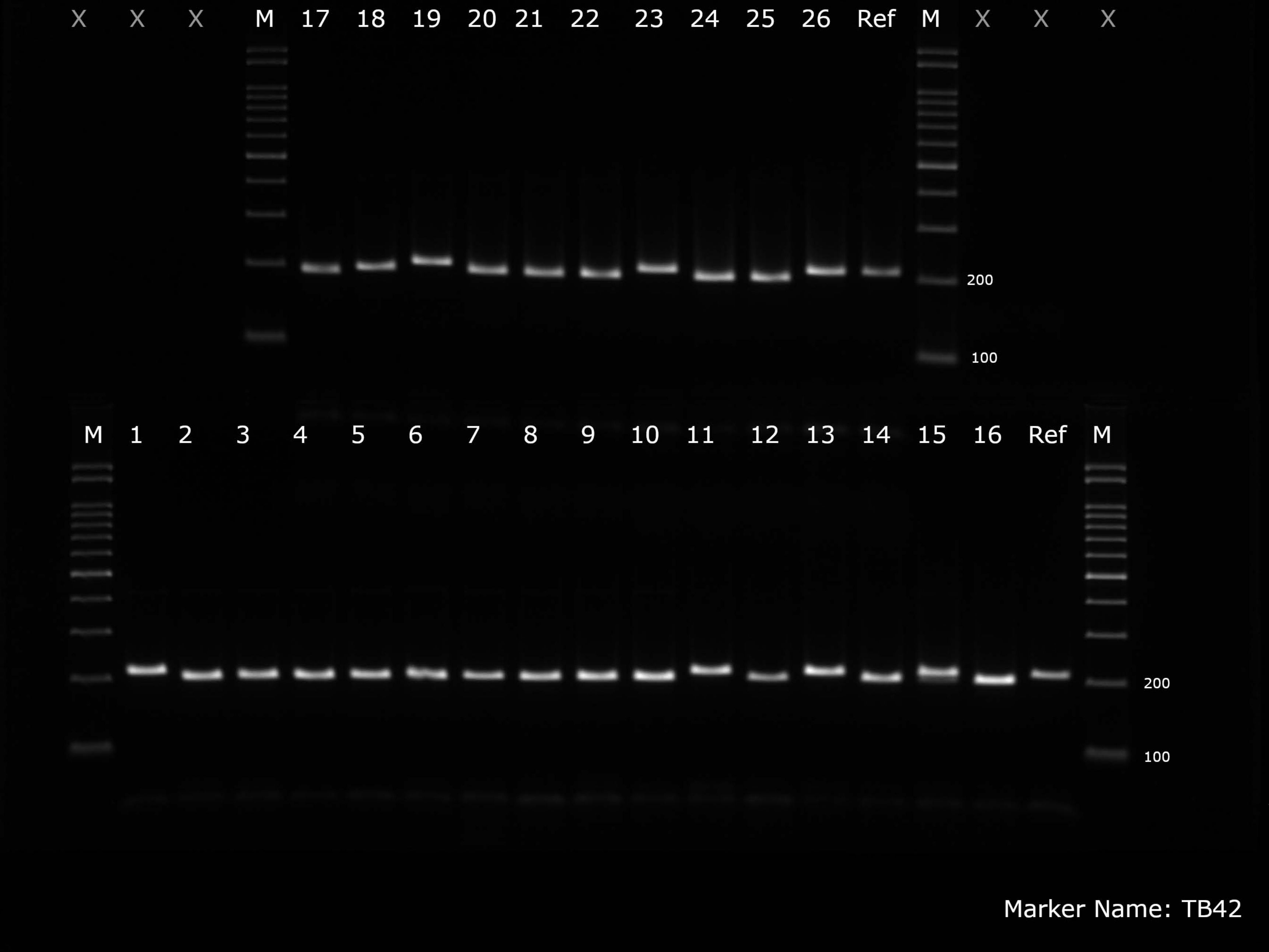
X X X M 17 18 19 20 21 22 23 24 25 26 Ref M X X X
200
100
M 1 2 3 4 5 6 7 8 9 10 11 12 13 14 15 16 Ref M
200
100
Marker Name: TB42

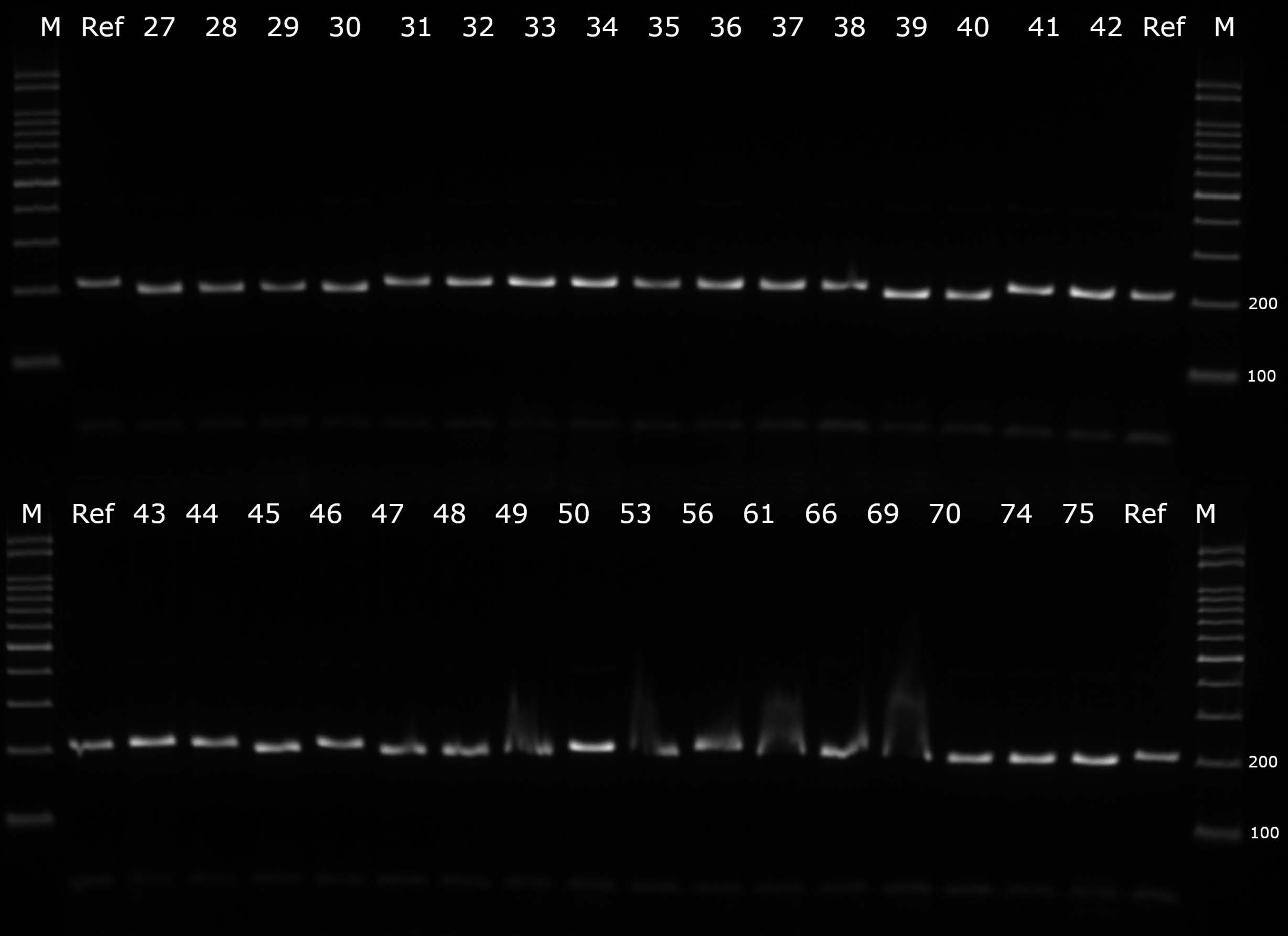

Marker Name: TB42

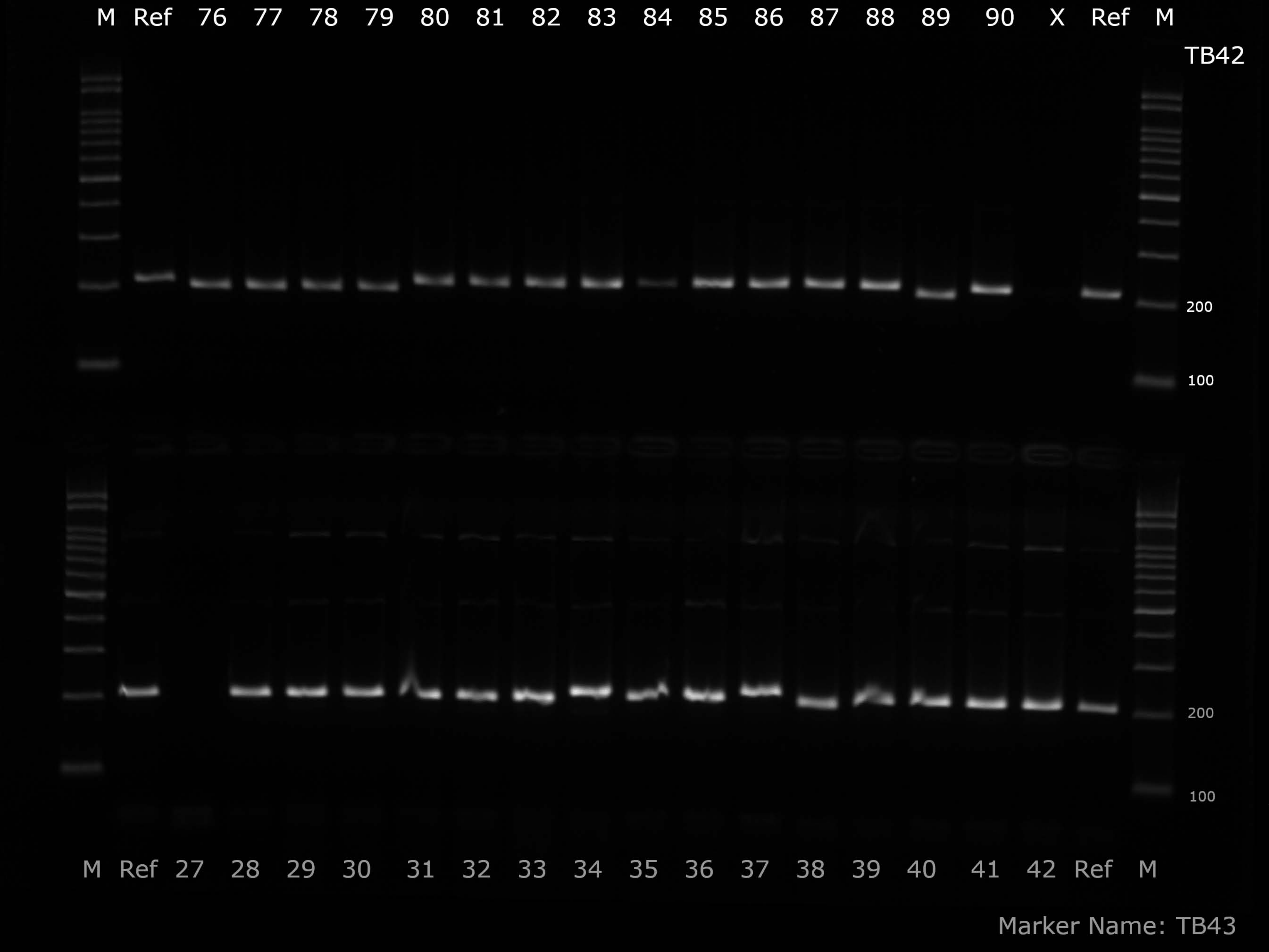
M Ref 76 77 78 79 80 81 82 83 84 85 86 87 88 89 90 X Ref M
TB42
200
100
200
100
M Ref 27 28 29 30 31 32 33 34 35 36 37 38 39 40 41 42 Ref M
Marker Name: TB43

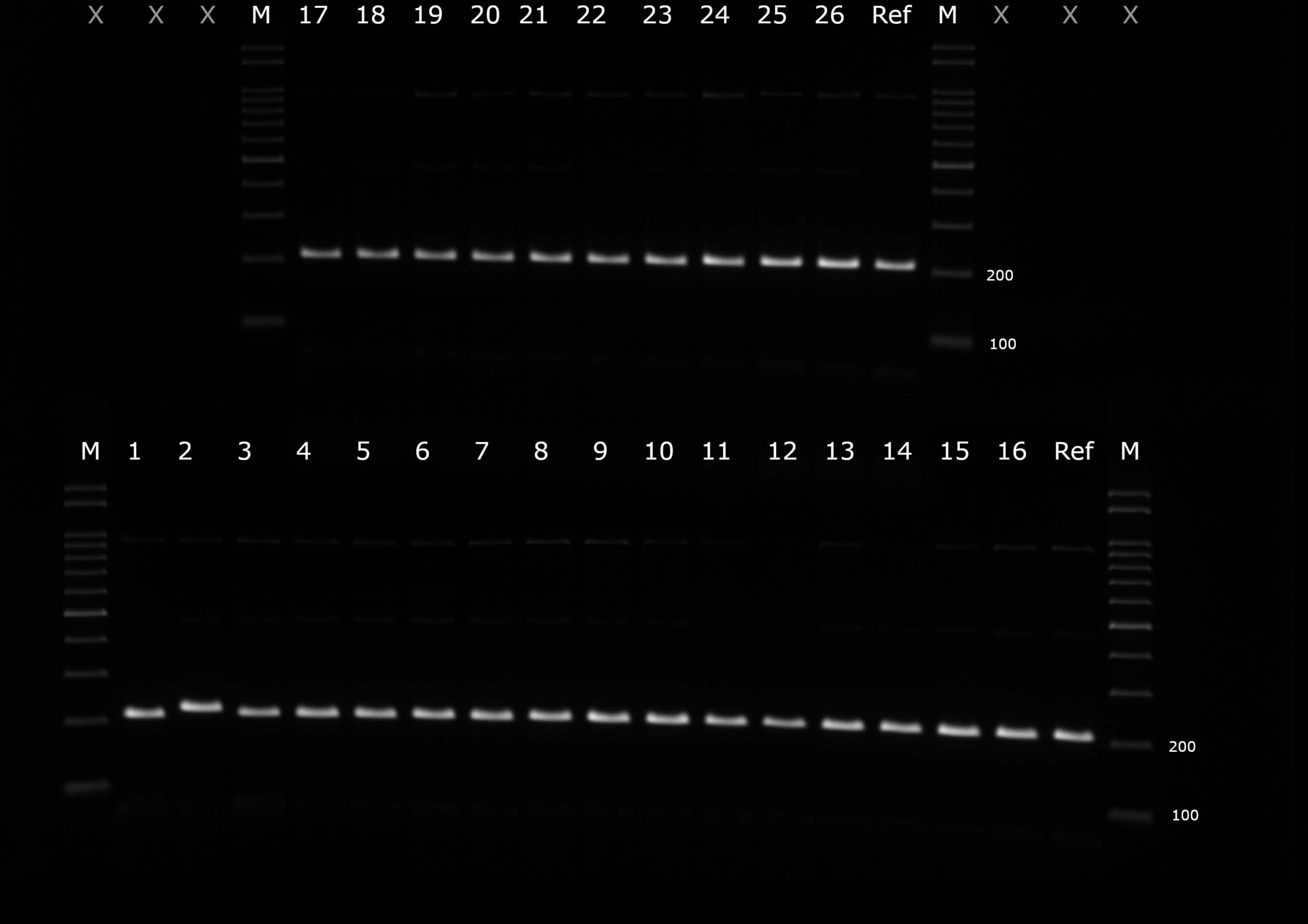

Marker Name: TB43

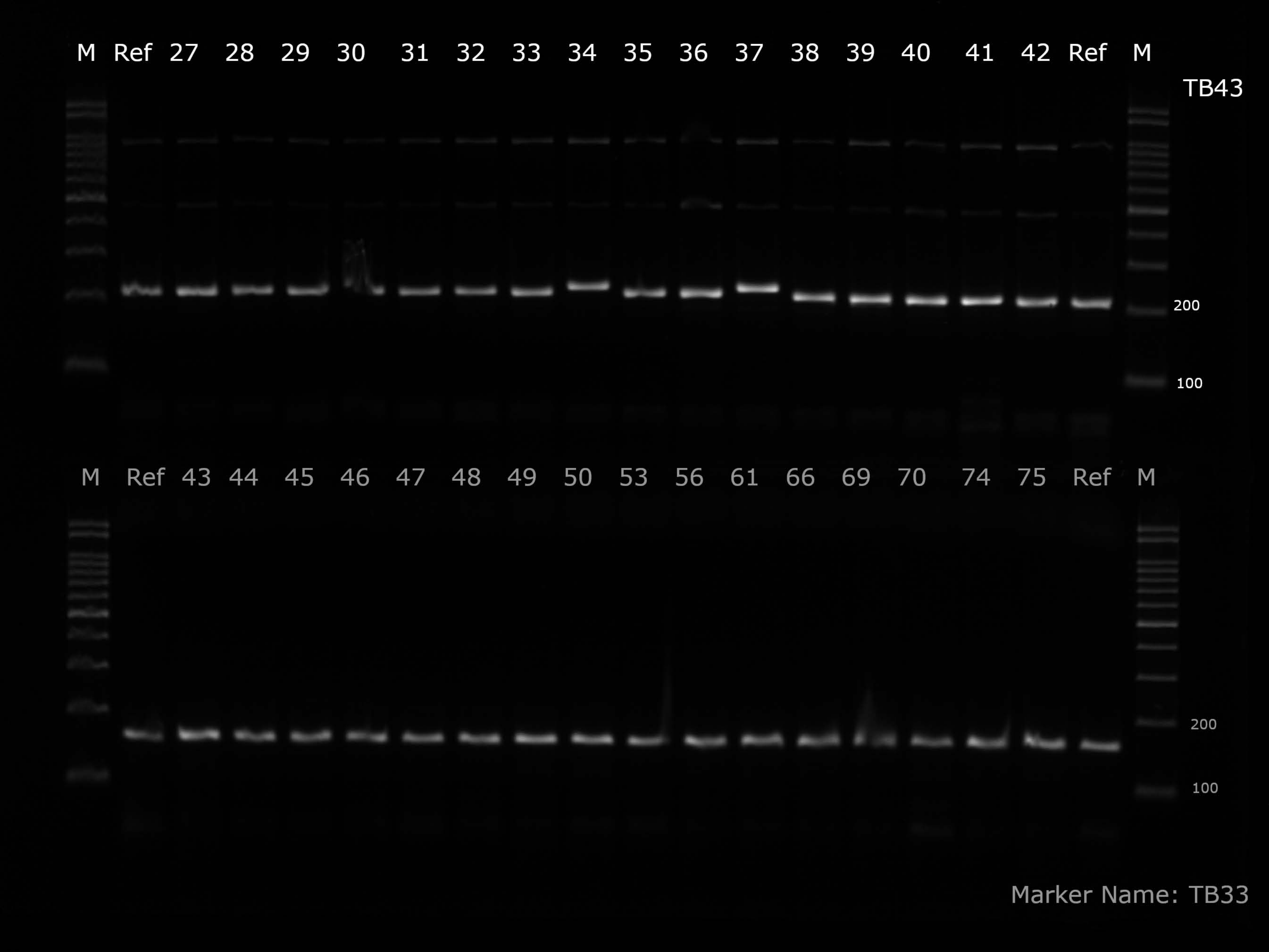
M Ref 27 28 29 30 31 32 33 34 35 36 37 38 39 40 41 42 Ref M
TB43
200
100
M Ref 43 44 45 46 47 48 49 50 53 56 61 66 69 70 74 75 Ref M
200
100
Marker Name: TB33

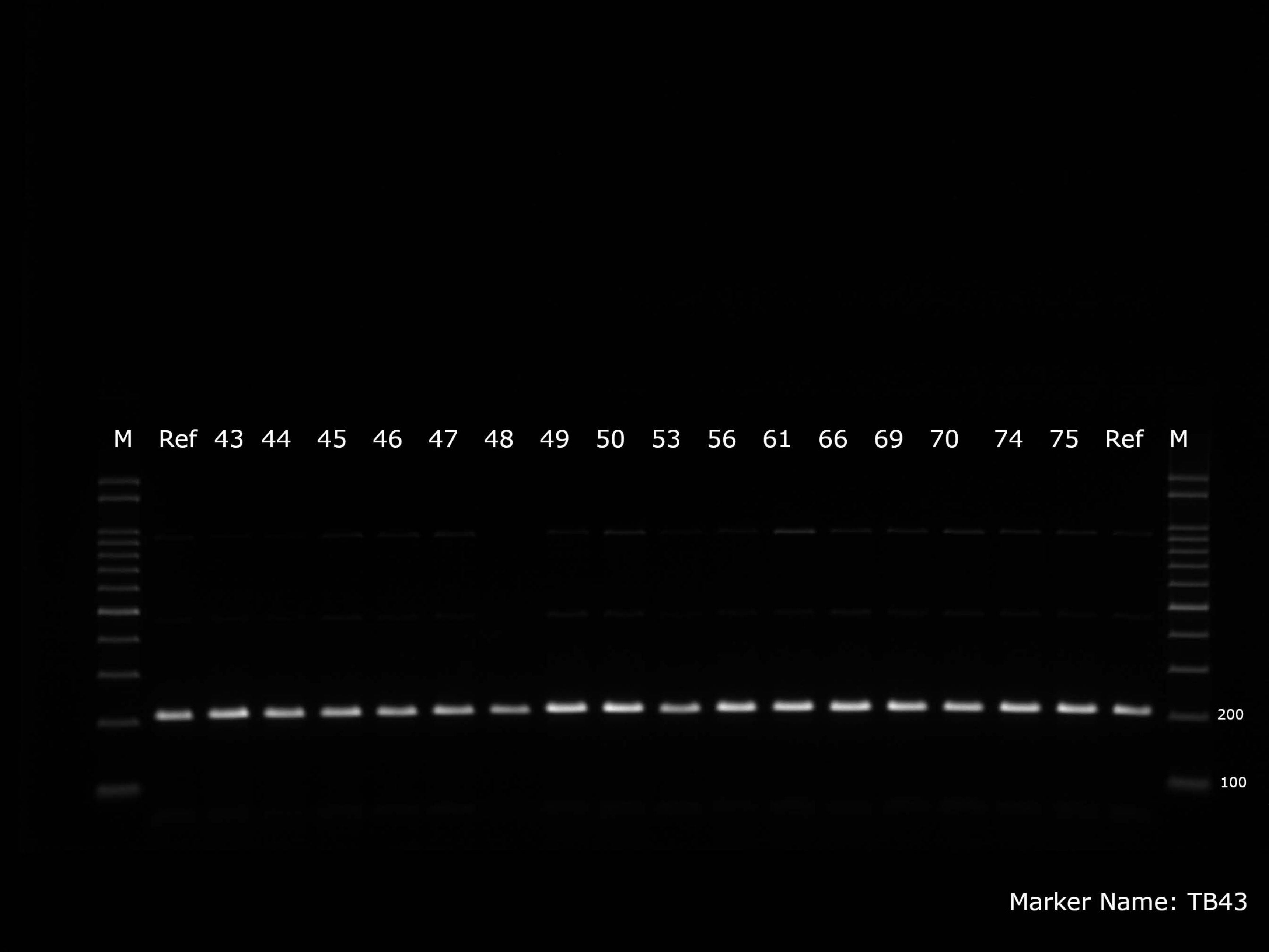
M Ref 43 44 45 46 47 48 49 50 53 56 61 66 69 70 74 75 Ref M
200
100
Marker Name: TB43

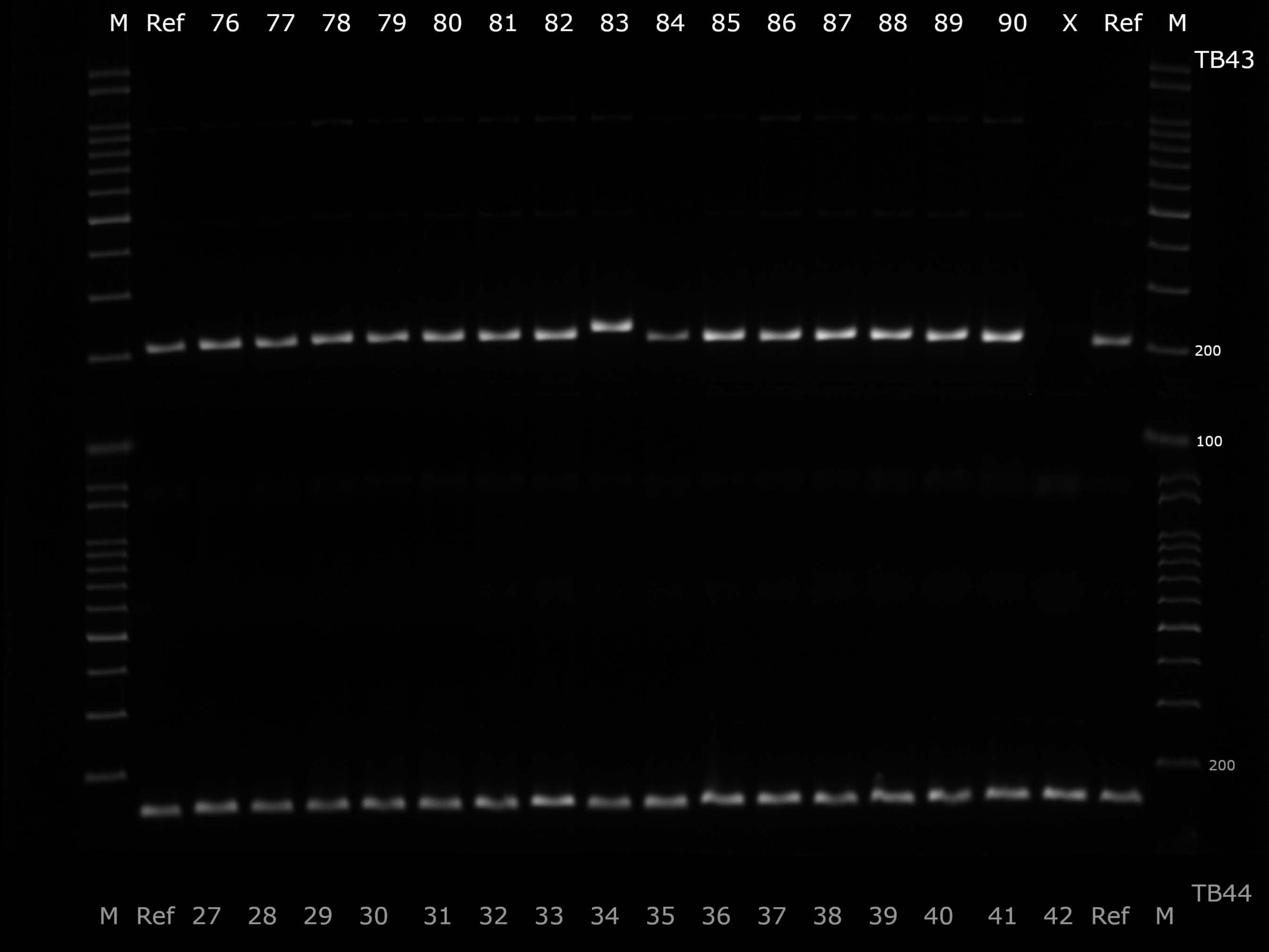
M Ref 76 77 78 79 80 81 82 83 84 85 86 87 88 89 90 X Ref M
TB43
200
100
200
TB44
M Ref 27 28 29 30 31 32 33 34 35 36 37 38 39 40 41 42 Ref M

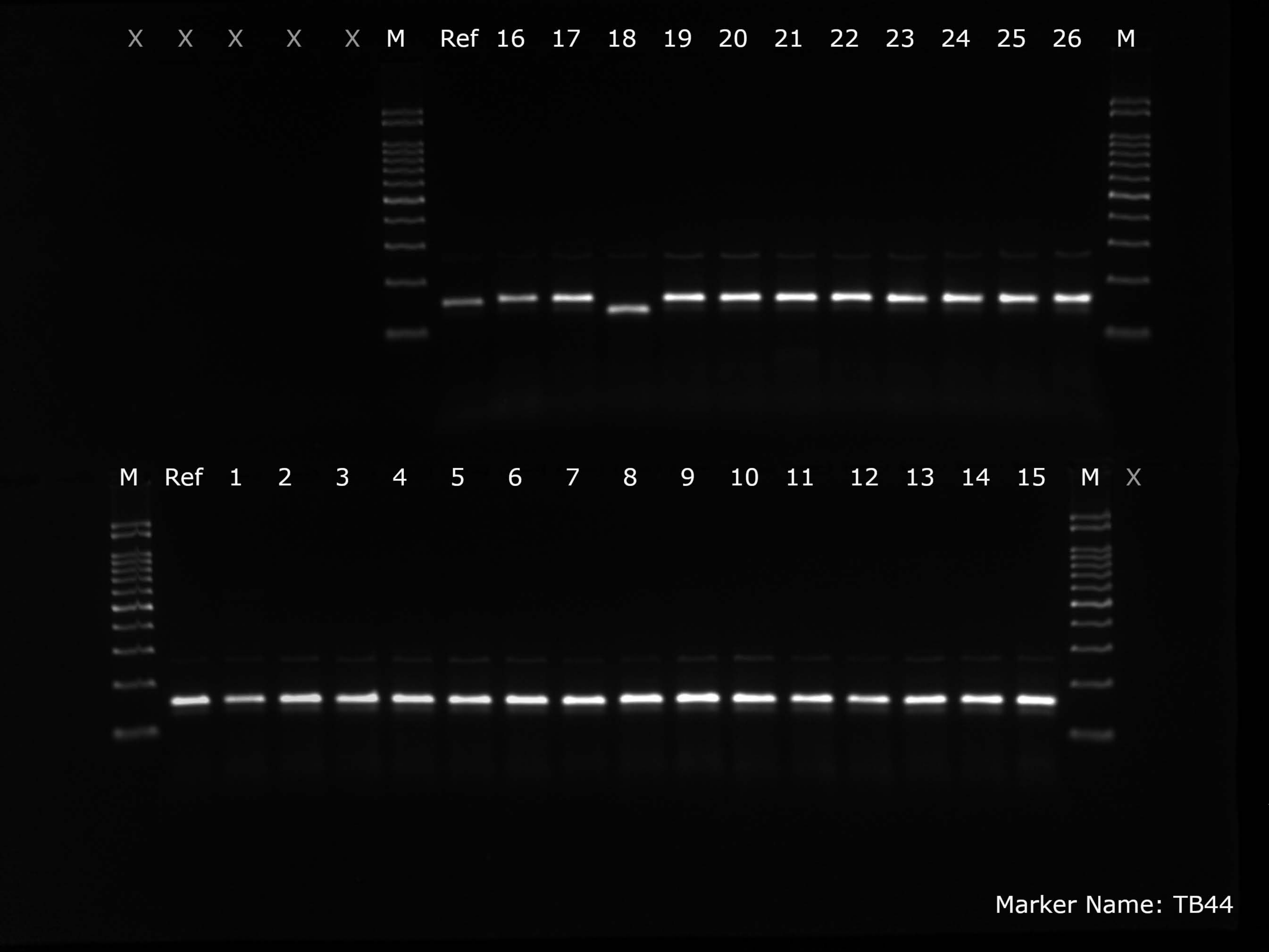
X X X X X M Ref 16 17 18 19 20 21 22 23 24 25 26 M
M Ref 1 2 3 4 5 6 7 8 9 10 11 12 13 14 15 M X
Marker Name: TB44

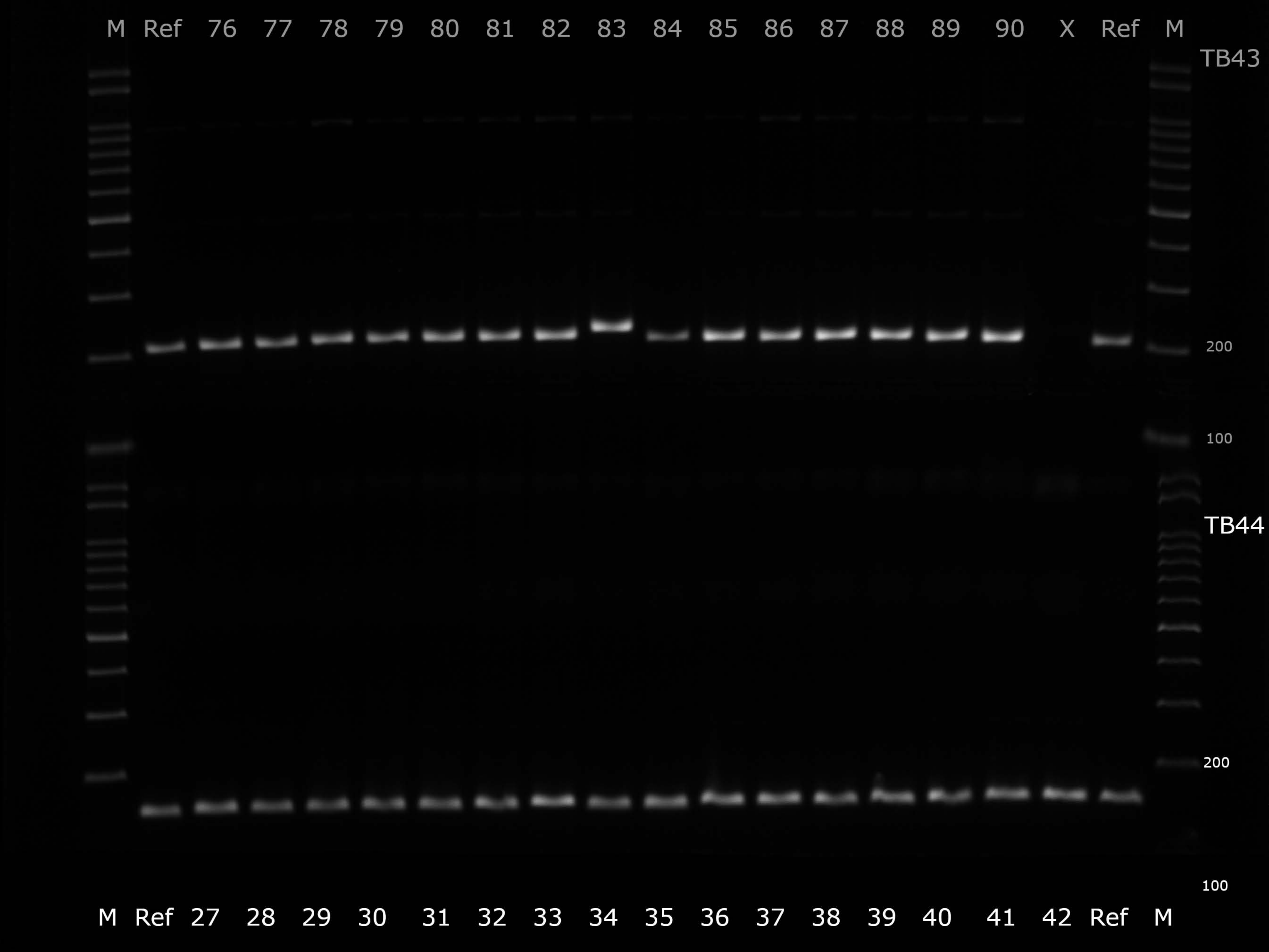
M Ref 76 77 78 79 80 81 82 83 84 85 86 87 88 89 90 X Ref M
TB43
200
100
TB44
200
100
M Ref 27 28 29 30 31 32 33 34 35 36 37 38 39 40 41 42 Ref M

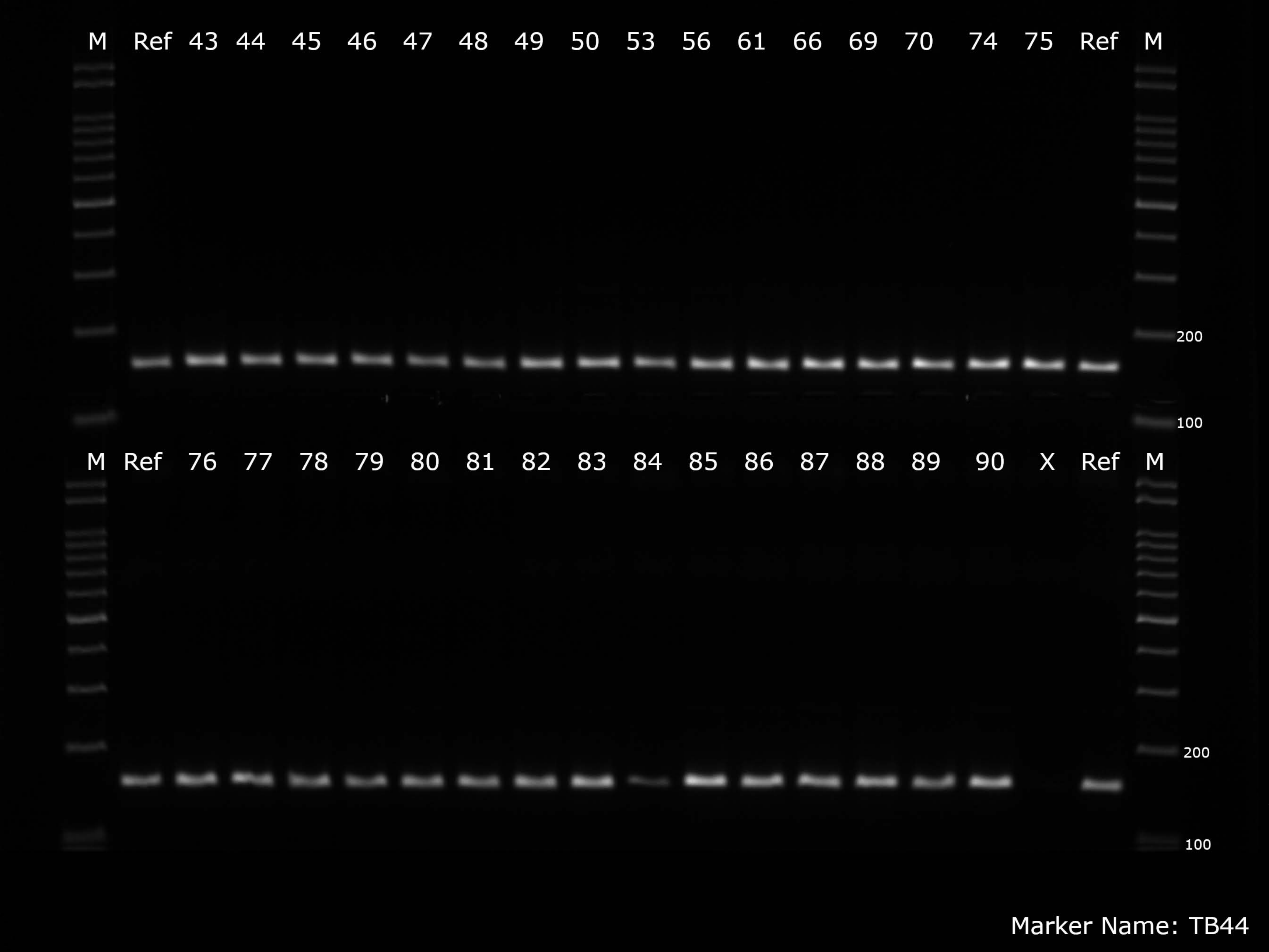
M Ref 43 44 45 46 47 48 49 50 53 56 61 66 69 70 74 75 Ref M
200
100
M Ref 76 77 78 79 80 81 82 83 84 85 86 87 88 89 90 X Ref M
200
100
Marker Name: TB44

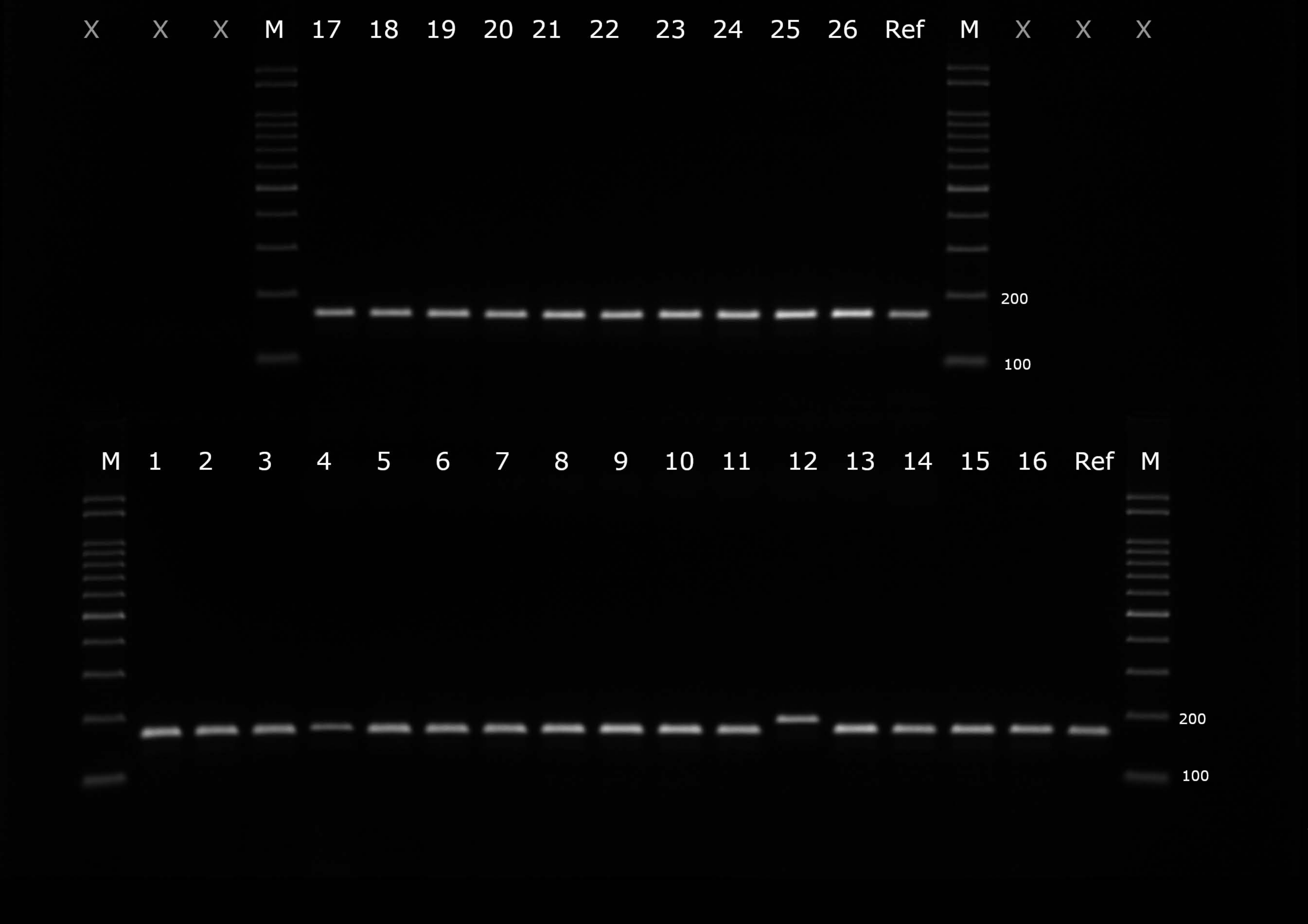

Marker Name: TB45

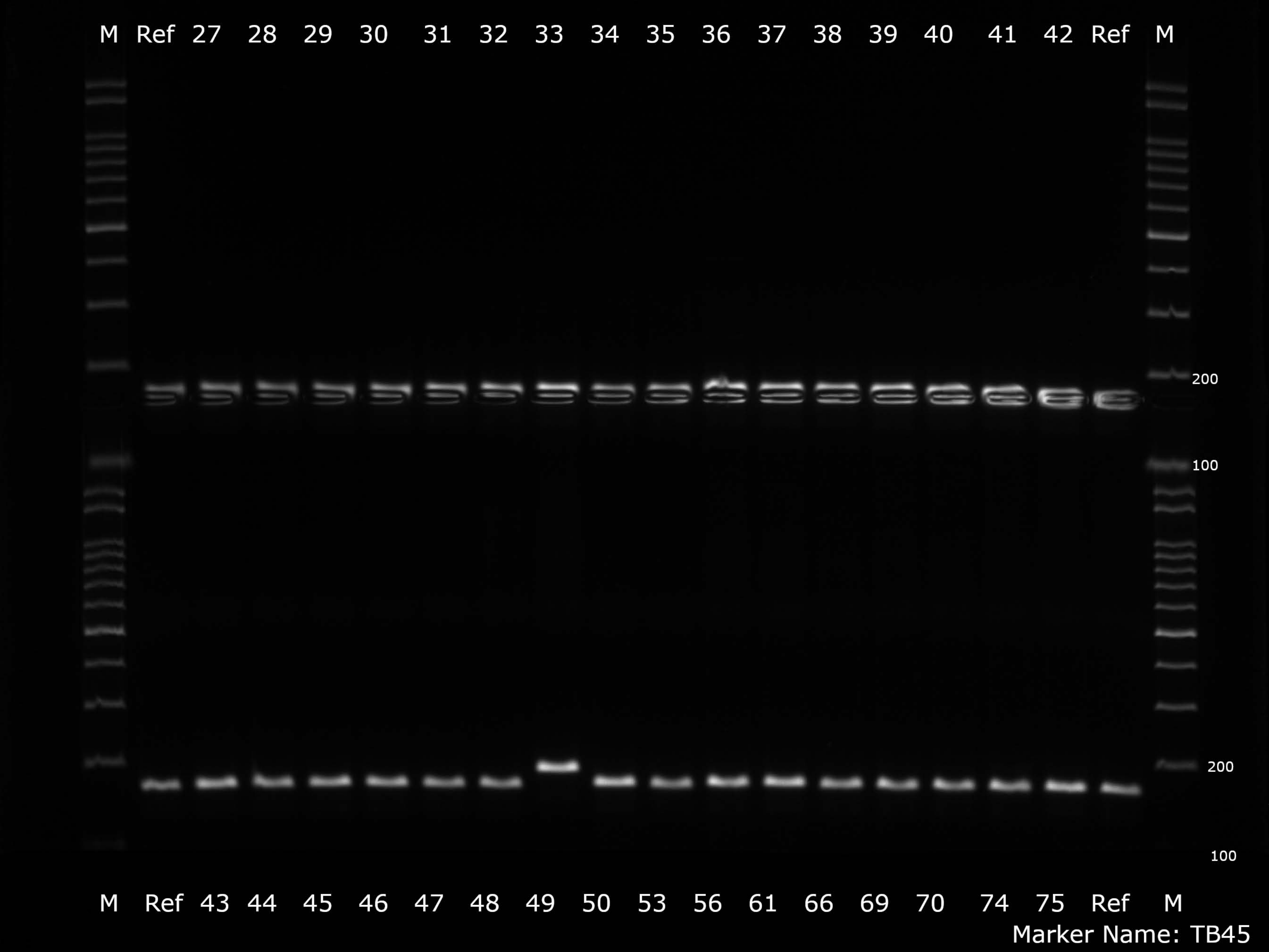
M Ref 27 28 29 30 31 32 33 34 35 36 37 38 39 40 41 42 Ref M
200
100
200
100
M Ref 43 44 45 46 47 48 49 50 53 56 61 66 69 70 74 75 Ref M
Marker Name: TB45

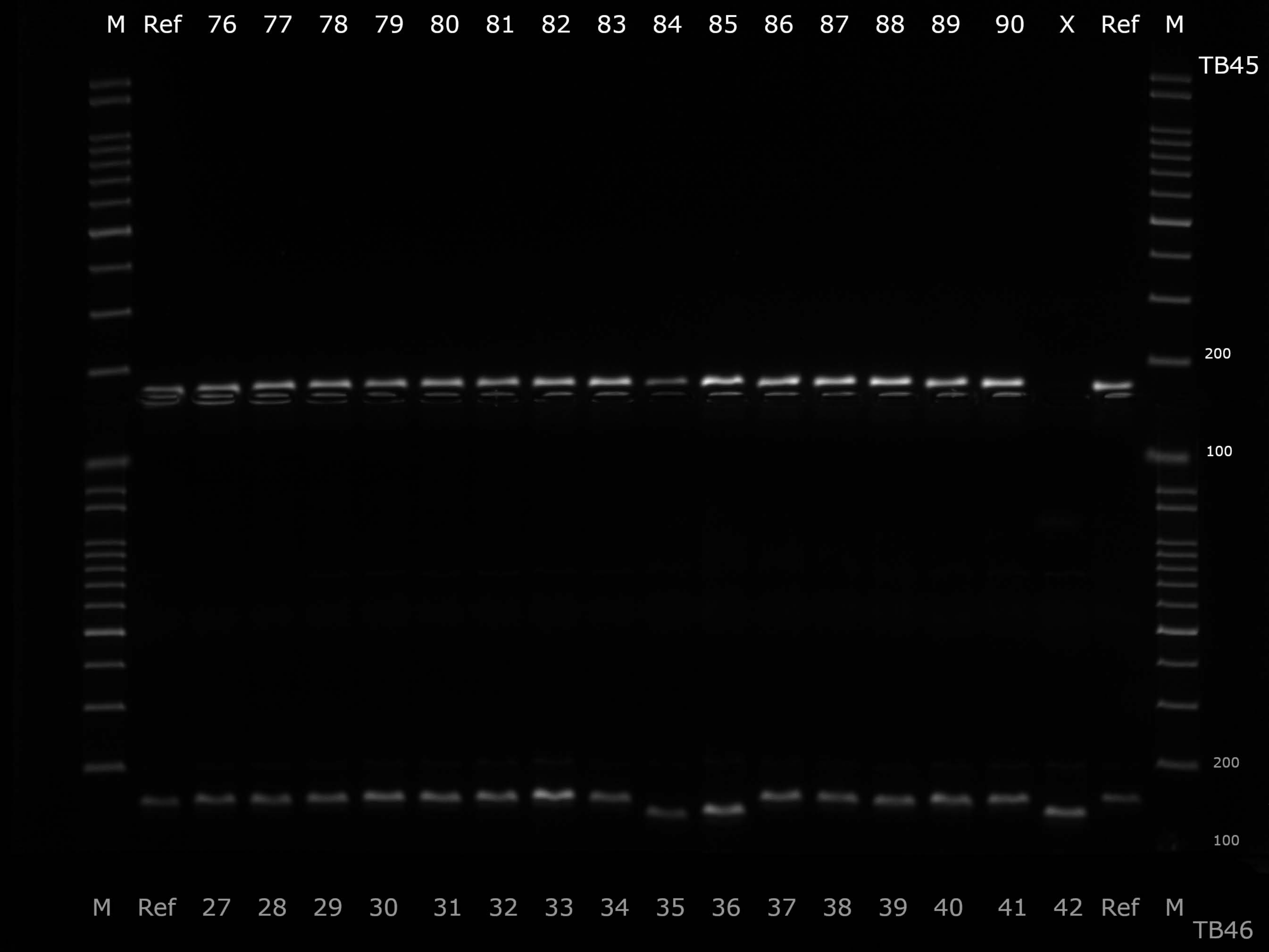
M Ref 76 77 78 79 80 81 82 83 84 85 86 87 88 89 90 X Ref M
TB45
200
100
200
100
M Ref 27 28 29 30 31 32 33 34 35 36 37 38 39 40 41 42 Ref M
TB46

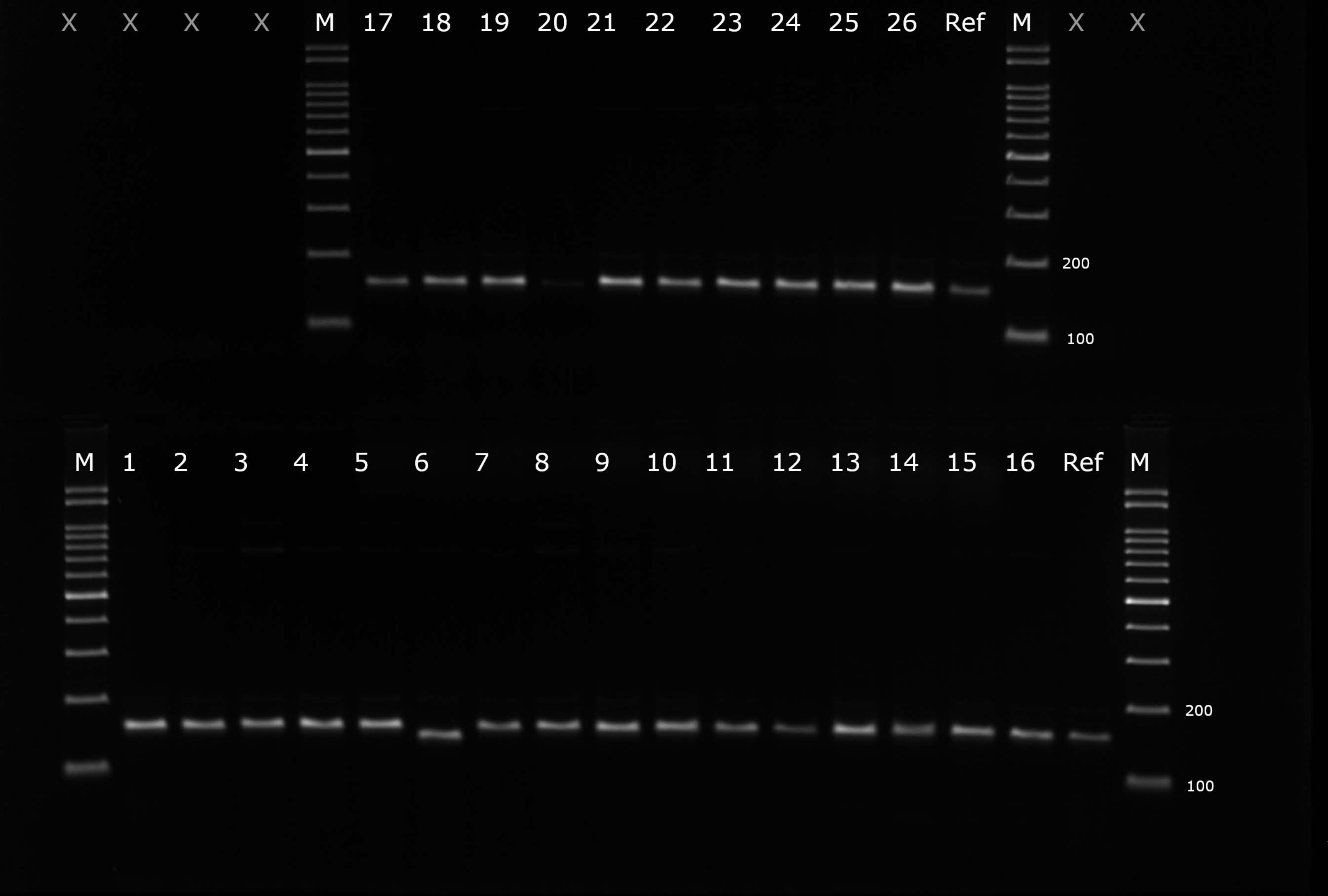

Marker Name: TB46

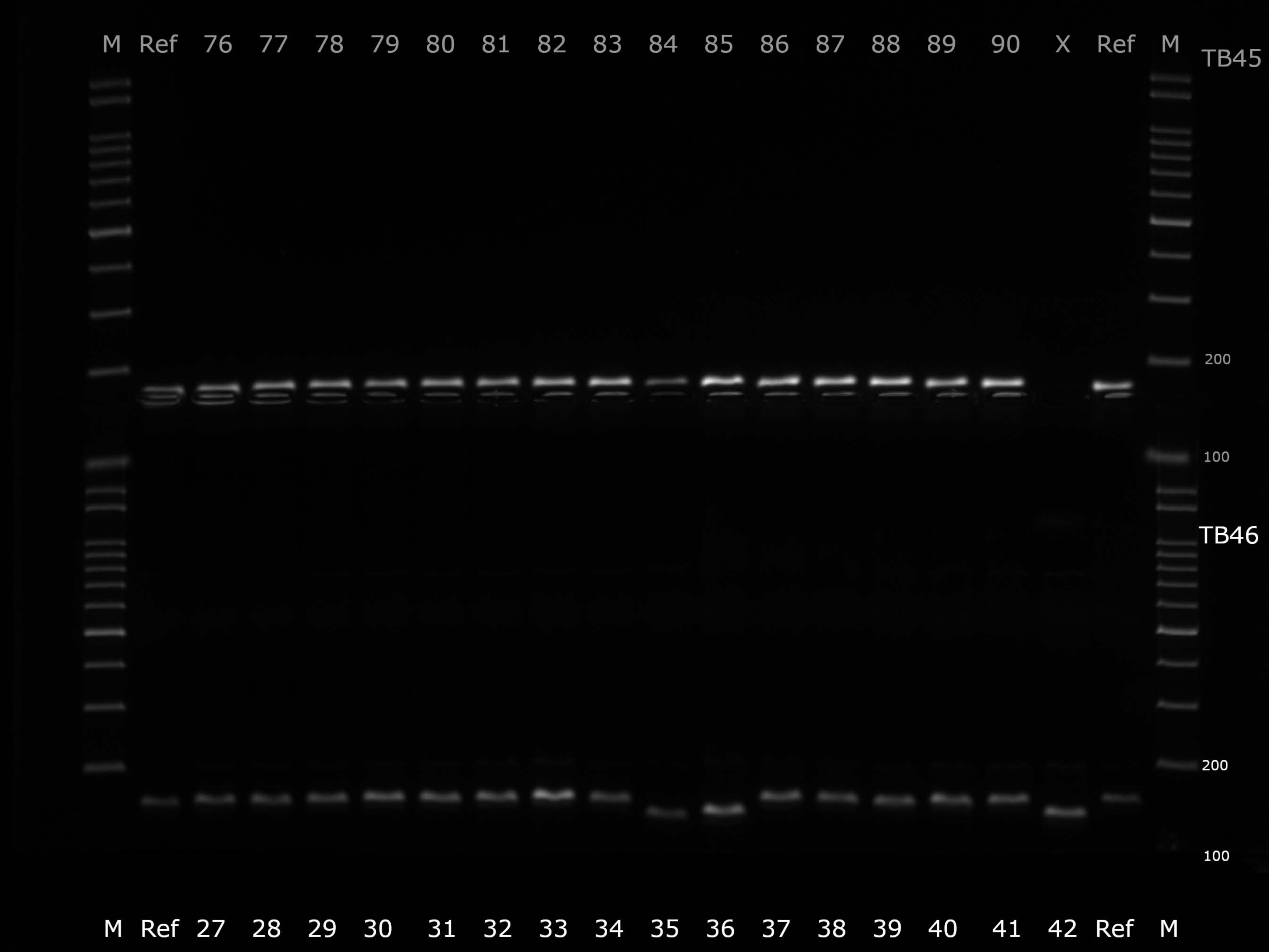
M Ref 76 77 78 79 80 81 82 83 84 85 86 87 88 89 90 X Ref M
TB45
200
100
TB46
200
100
M Ref 27 28 29 30 31 32 33 34 35 36 37 38 39 40 41 42 Ref M

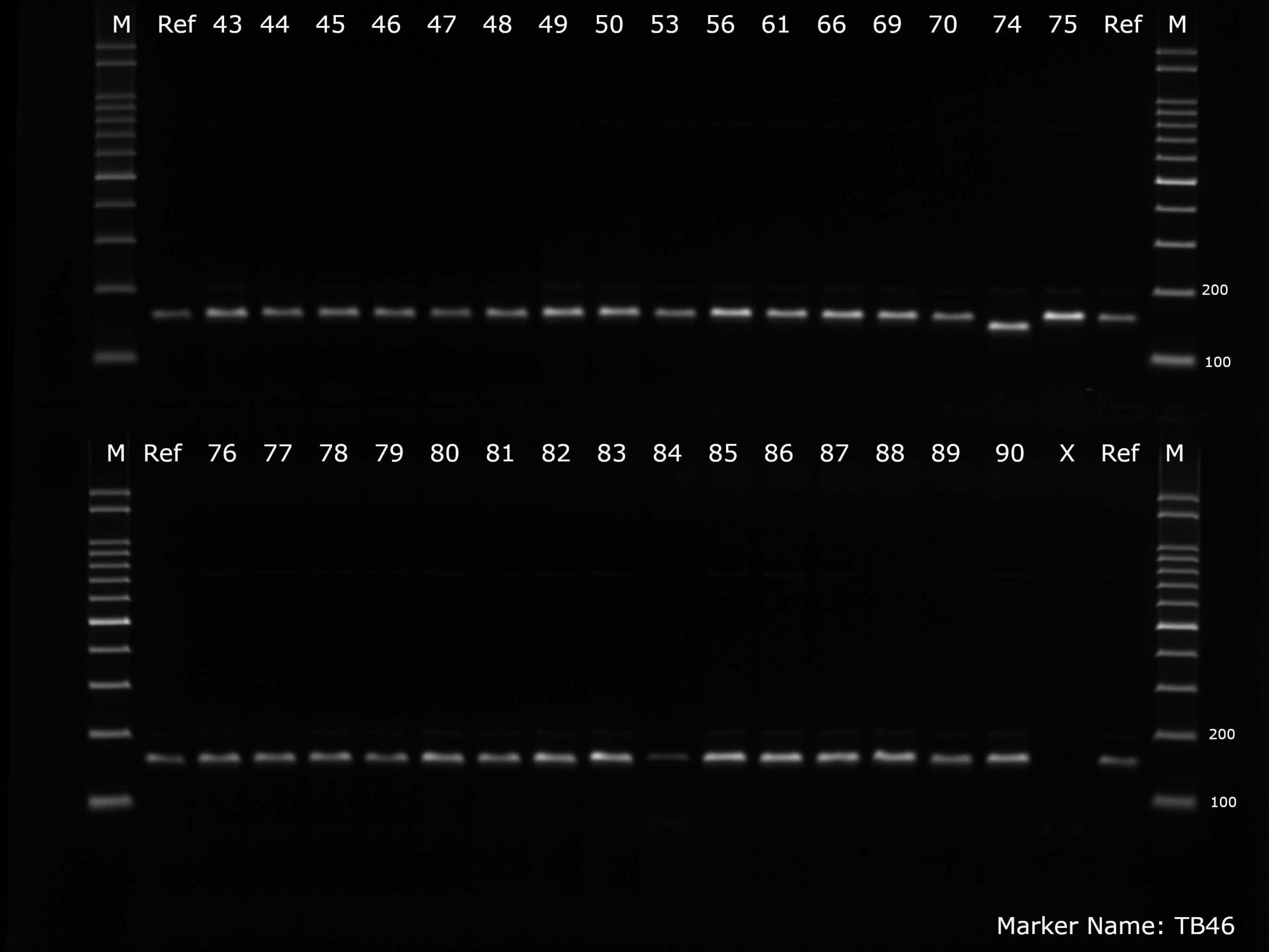
M Ref 43 44 45 46 47 48 49 50 53 56 61 66 69 70 74 75 Ref M
200
100
M Ref 76 77 78 79 80 81 82 83 84 85 86 87 88 89 90 X Ref M
200
100
Marker Name: TB46

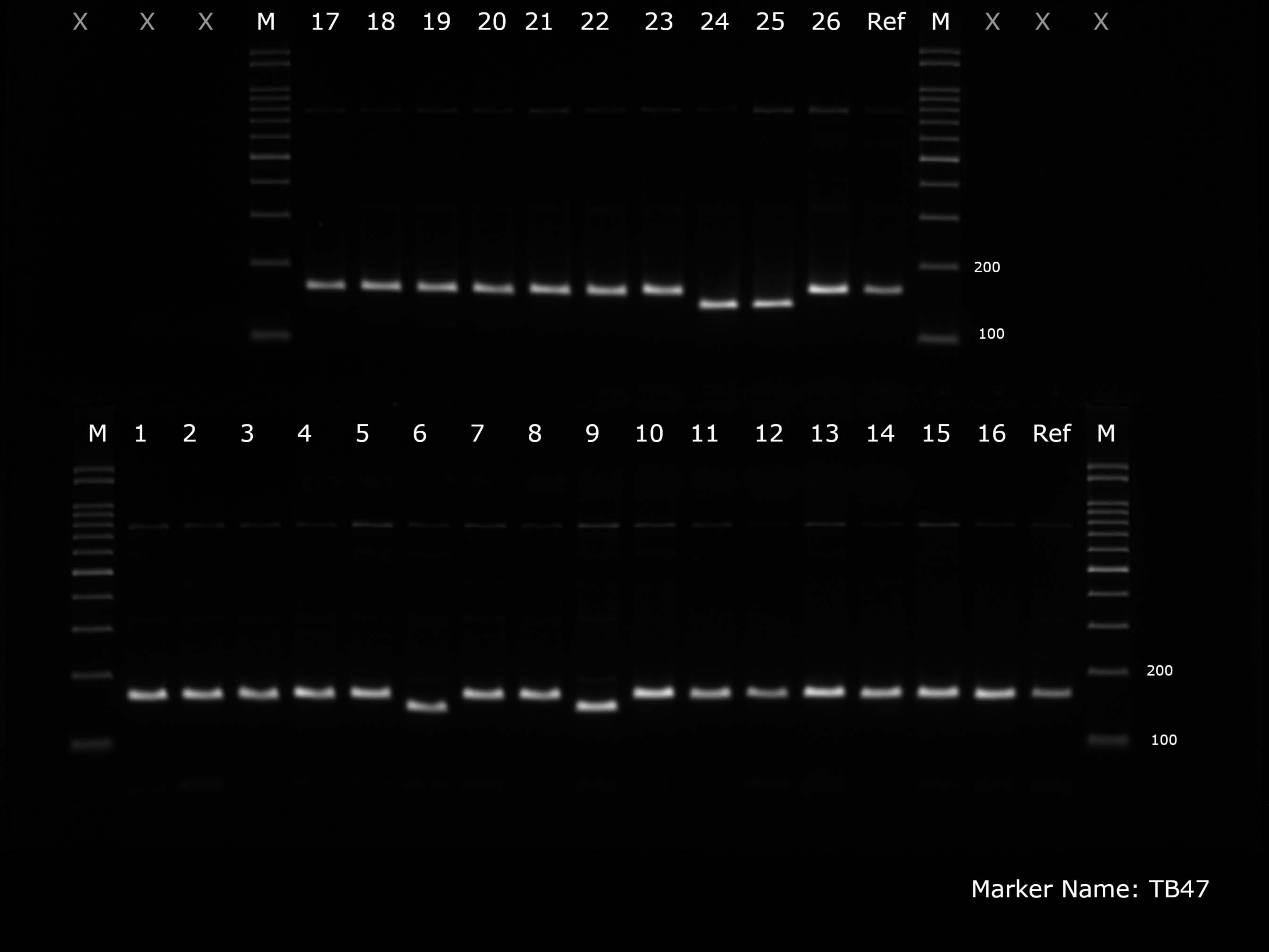
X X X M 17 18 19 20 21 22 23 24 25 26 Ref M X X X
200
100
M 1 2 3 4 5 6 7 8 9 10 11 12 13 14 15 16 Ref M
200
100
Marker Name: TB47

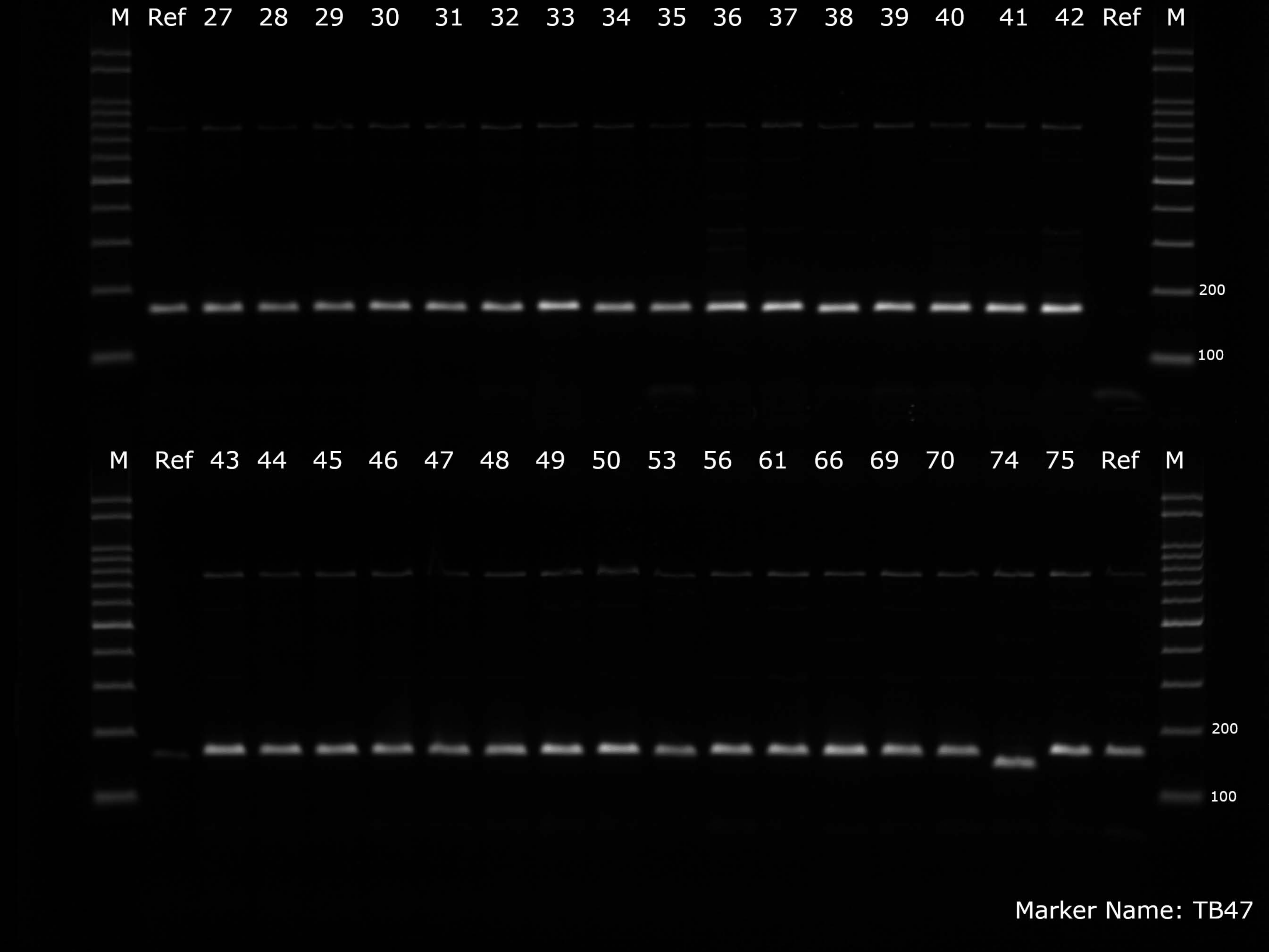
M Ref 27 28 29 30 31 32 33 34 35 36 37 38 39 40 41 42 Ref M
200
100
M Ref 43 44 45 46 47 48 49 50 53 56 61 66 69 70 74 75 Ref M
200
100
Marker Name: TB47

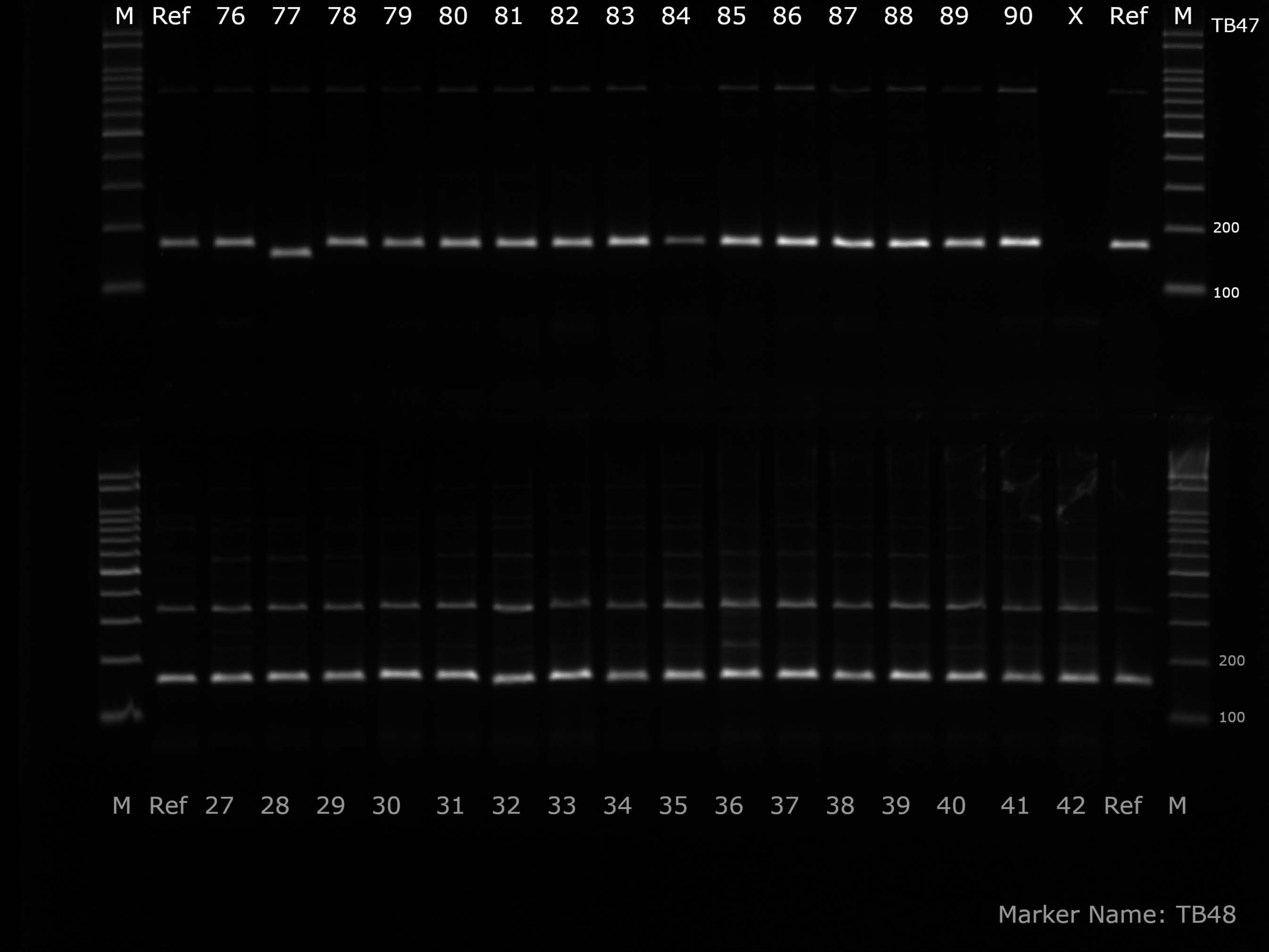
M Ref 76 77 78 79 80 81 82 83 84 85 86 87 88 89 90 X Ref M
TB47
200
100
200
100
M Ref 27 28 29 30 31 32 33 34 35 36 37 38 39 40 41 42 Ref M
Marker Name: TB48

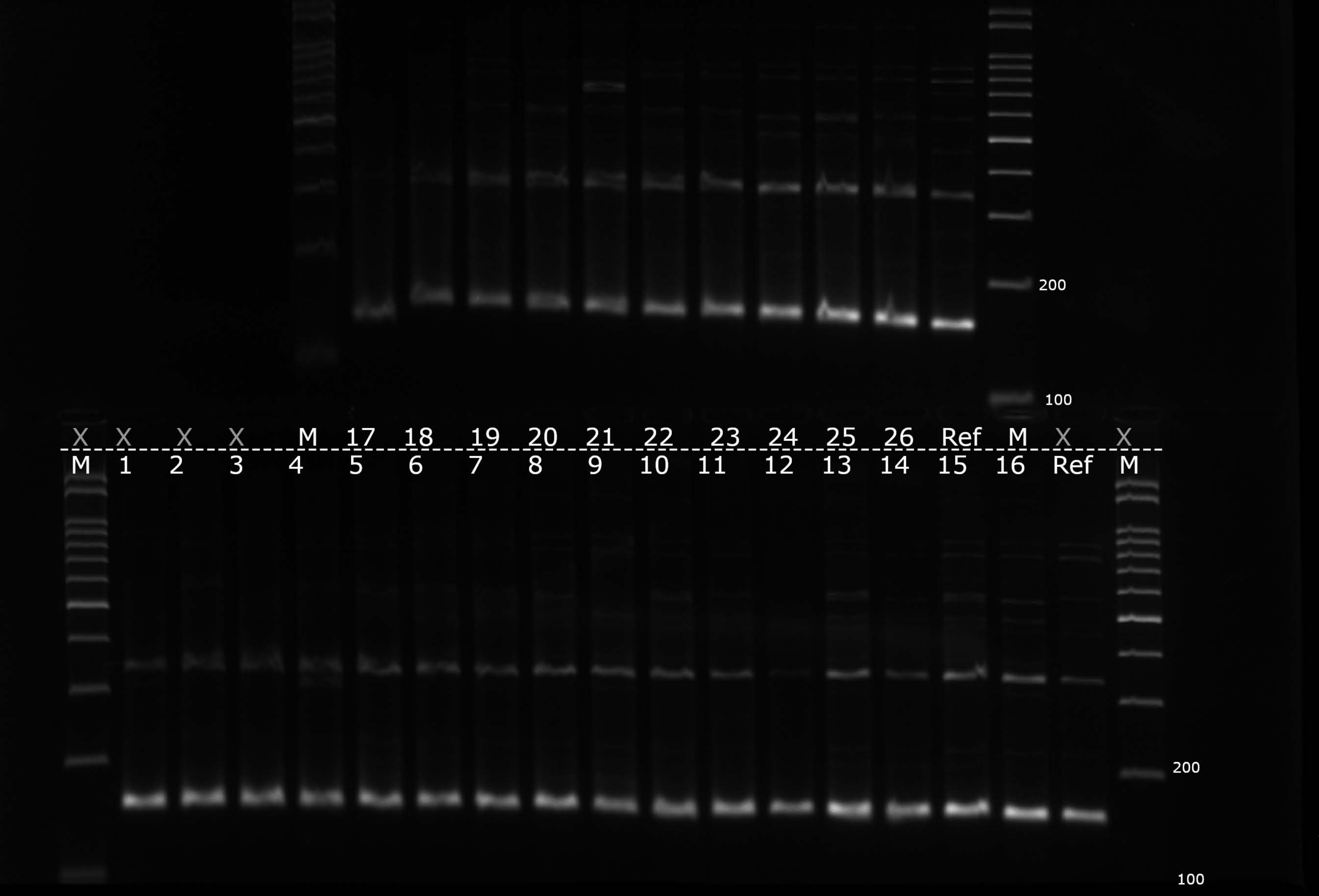

Marker Name: TB48

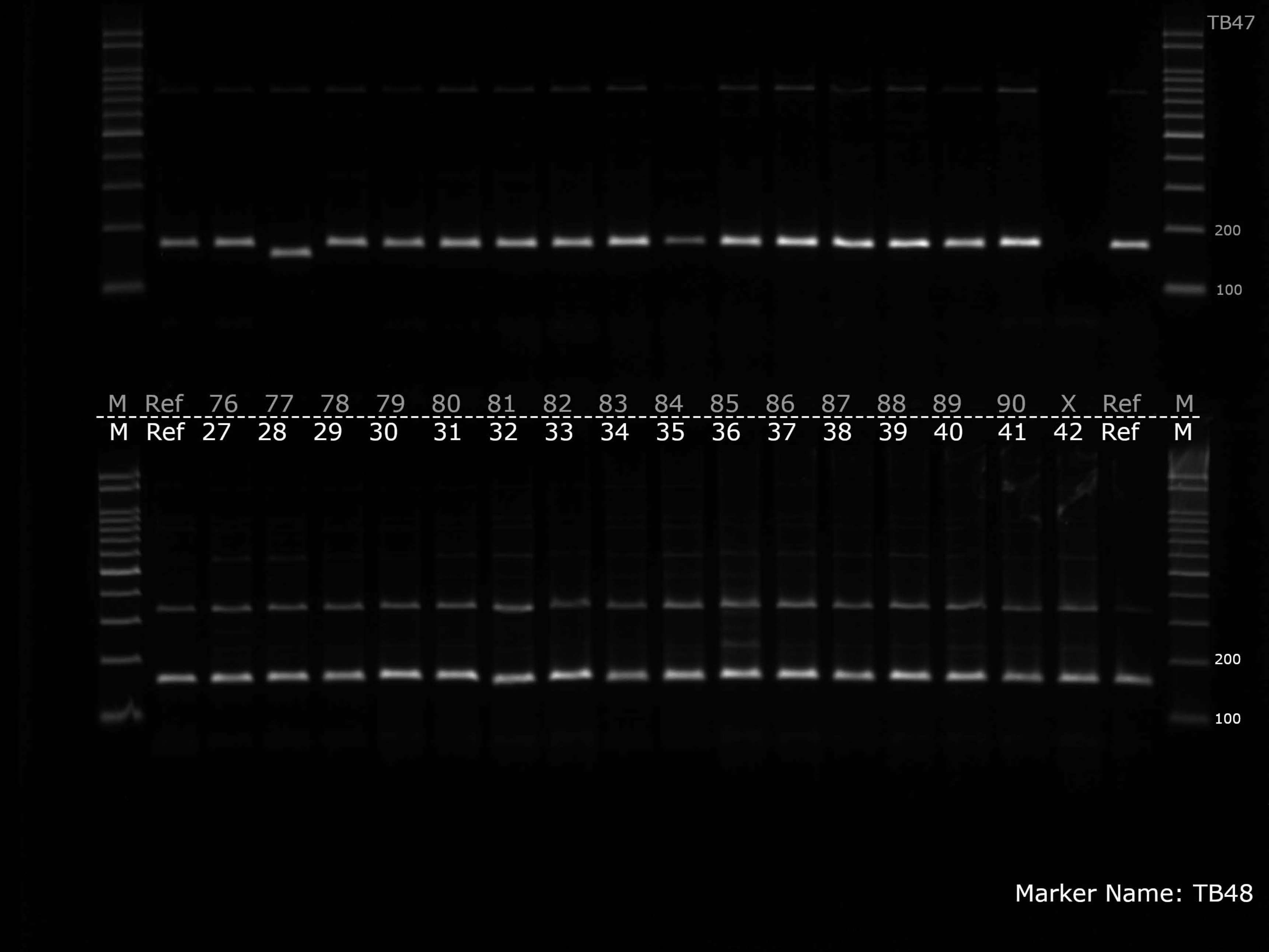
TB47
M Ref 76 77 78 79 80 81 82 83 84 85 86 87 88 89 90 X Ref M
M Ref 27 28 29 30 31 32 33 34 35 36 37 38 39 40 41 42 Ref M
200
100
200
100
Marker Name: TB48

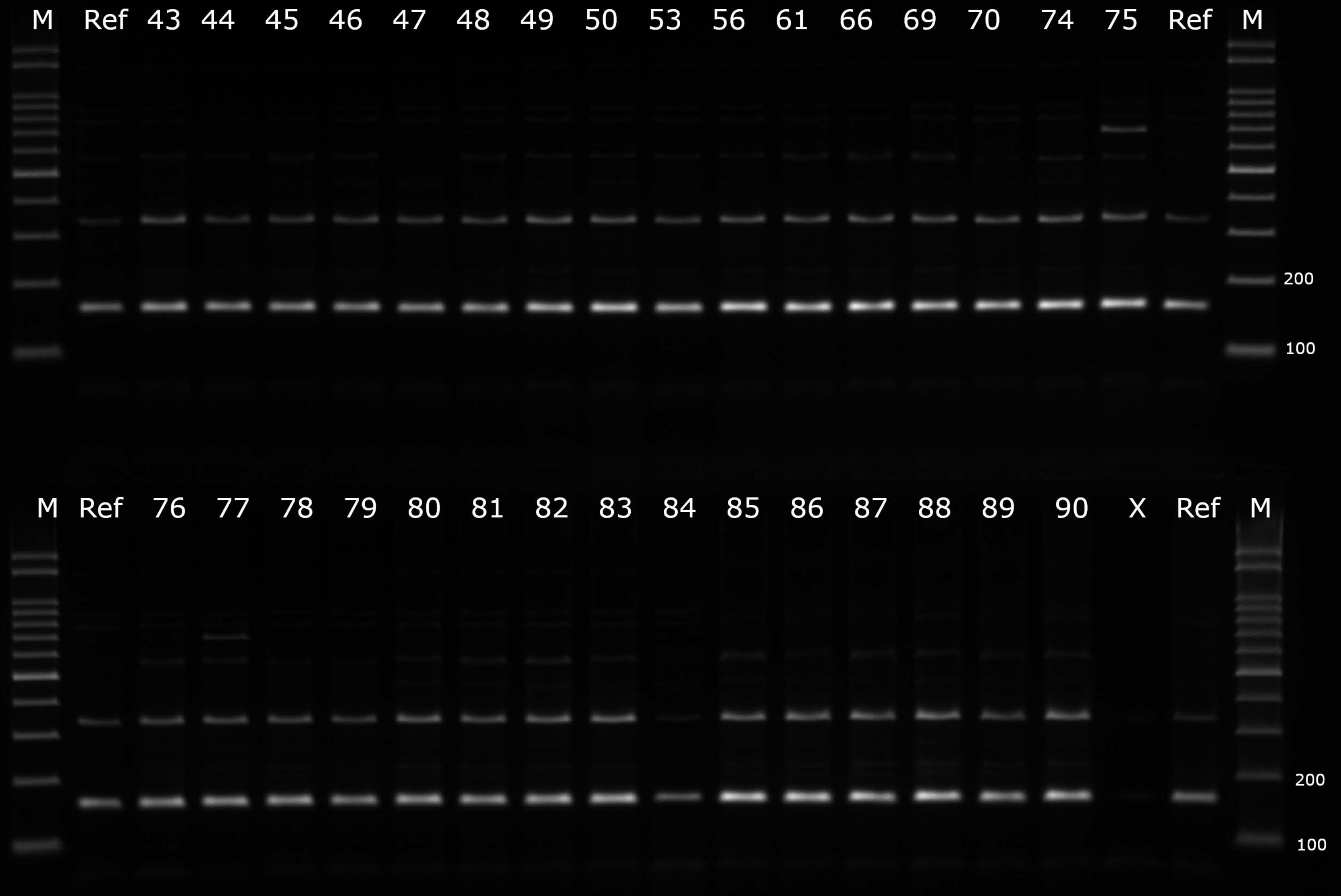

Marker Name: TB48

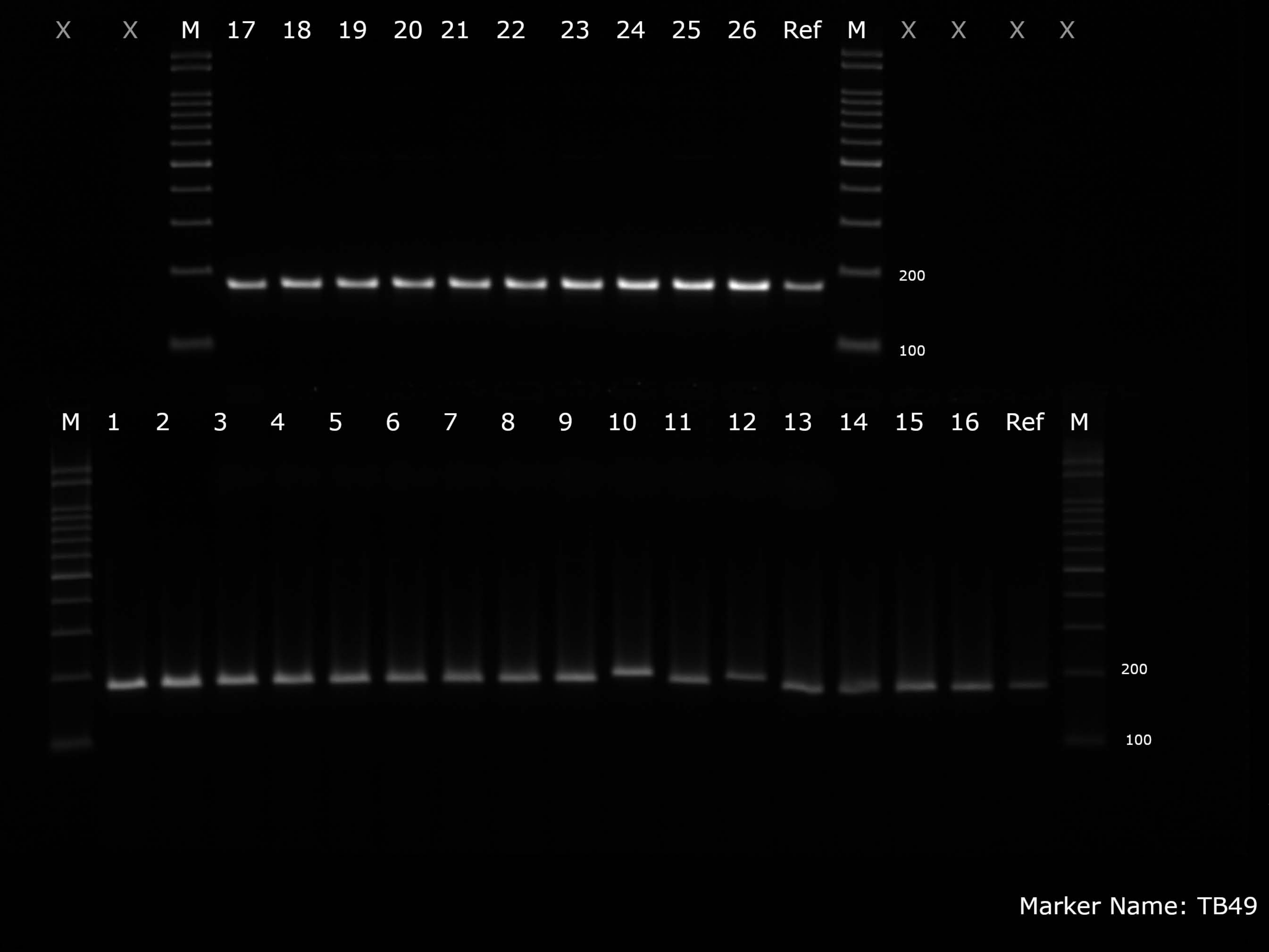
X X M 17 18 19 20 21 22 23 24 25 26 Ref M X X X X
200
100
M 1 2 3 4 5 6 7 8 9 10 11 12 13 14 15 16 Ref M
200
100
Marker Name: TB49

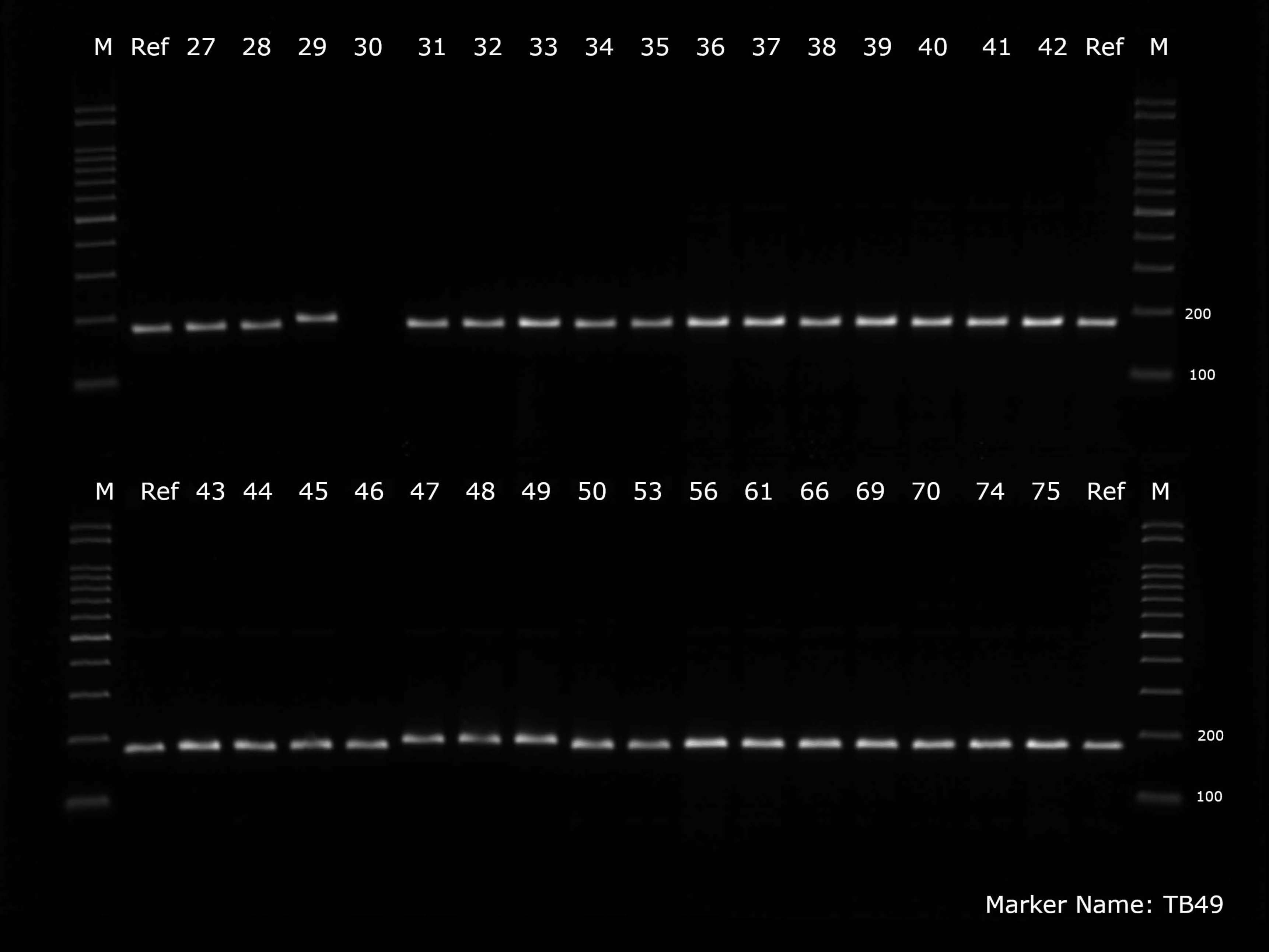
M Ref 27 28 29 30 31 32 33 34 35 36 37 38 39 40 41 42 Ref M
200
100
M Ref 43 44 45 46 47 48 49 50 53 56 61 66 69 70 74 75 Ref M
200
100
Marker Name: TB49

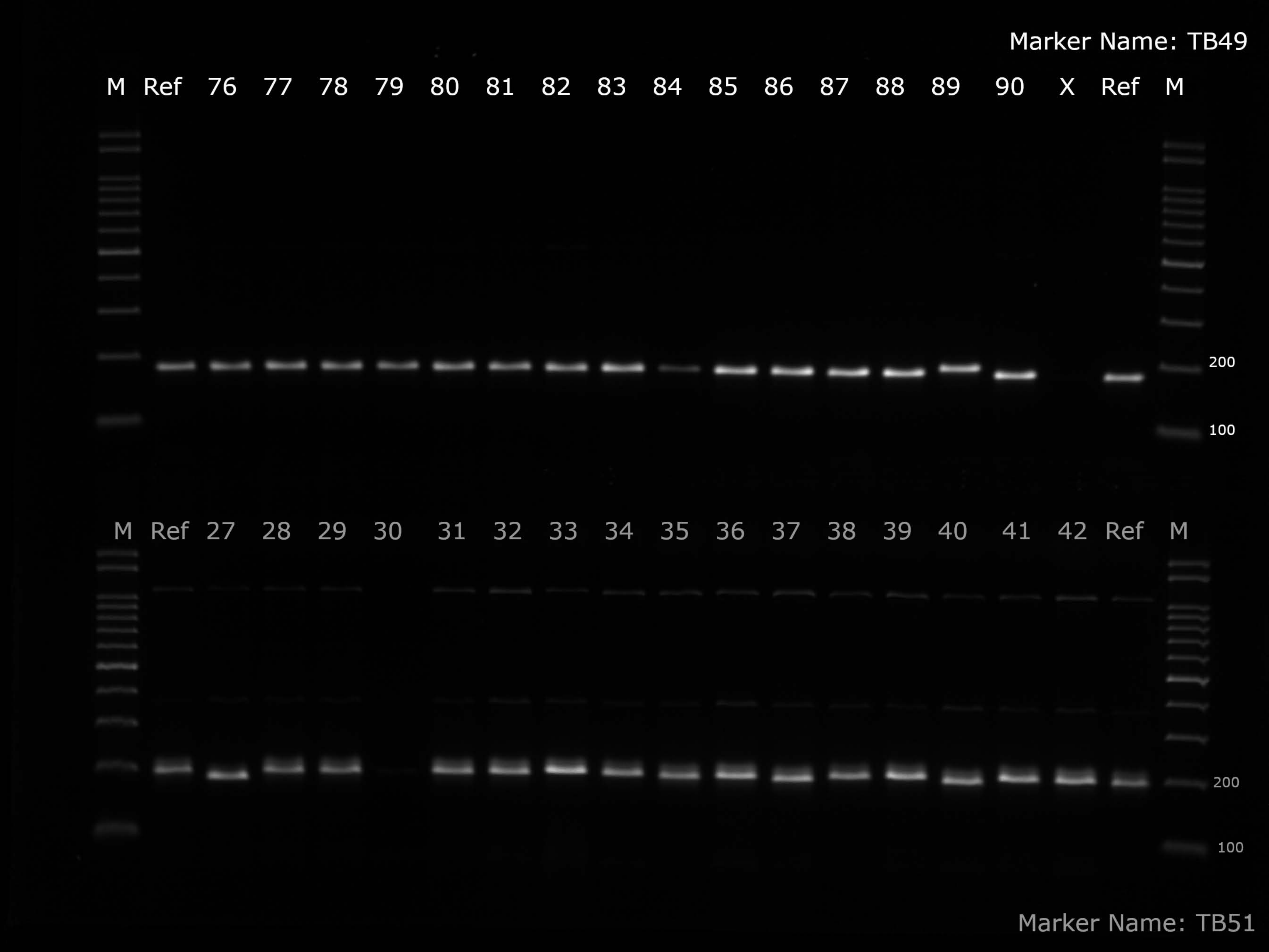

Marker Name: TB49
M Ref 76 77 78 79 80 81 82 83 84 85 86 87 88 89 90 X Ref M
200
100
M Ref 27 28 29 30 31 32 33 34 35 36 37 38 39 40 41 42 Ref M
200
100
Marker Name: TB51

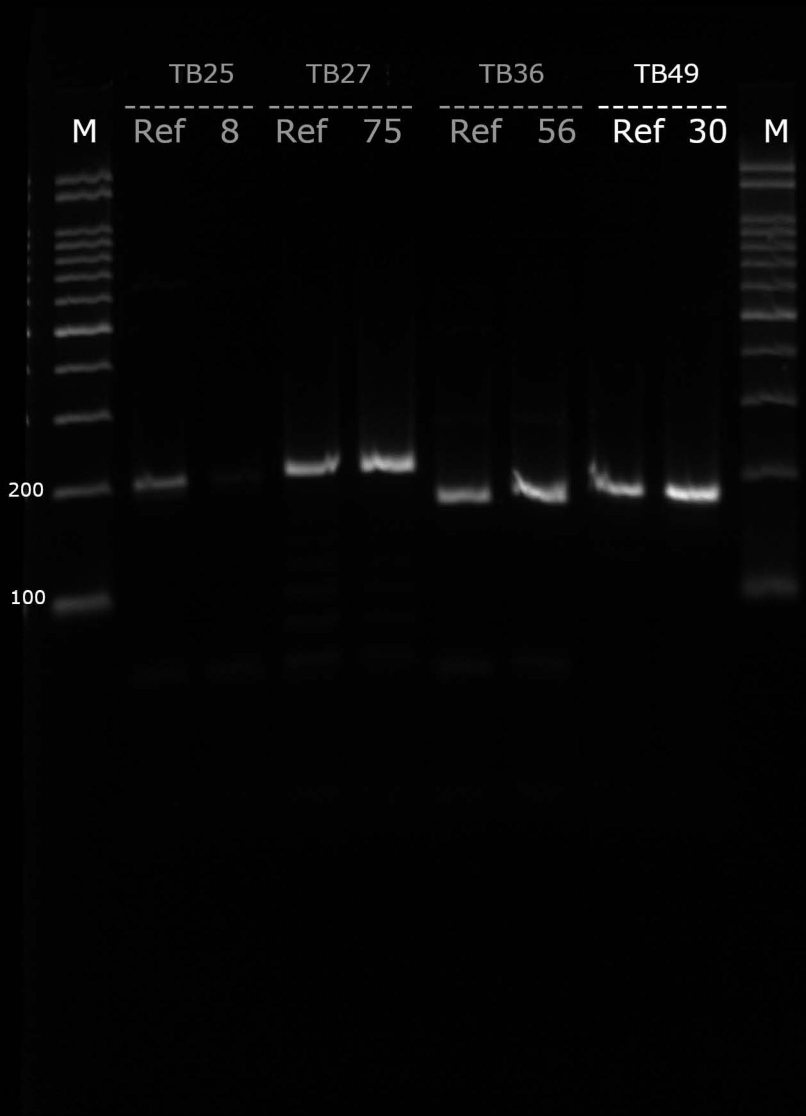

TB25
TB27
TB36
TB49
M
Ref
8
Ref
75
Ref
56
Ref
30
M
200
100

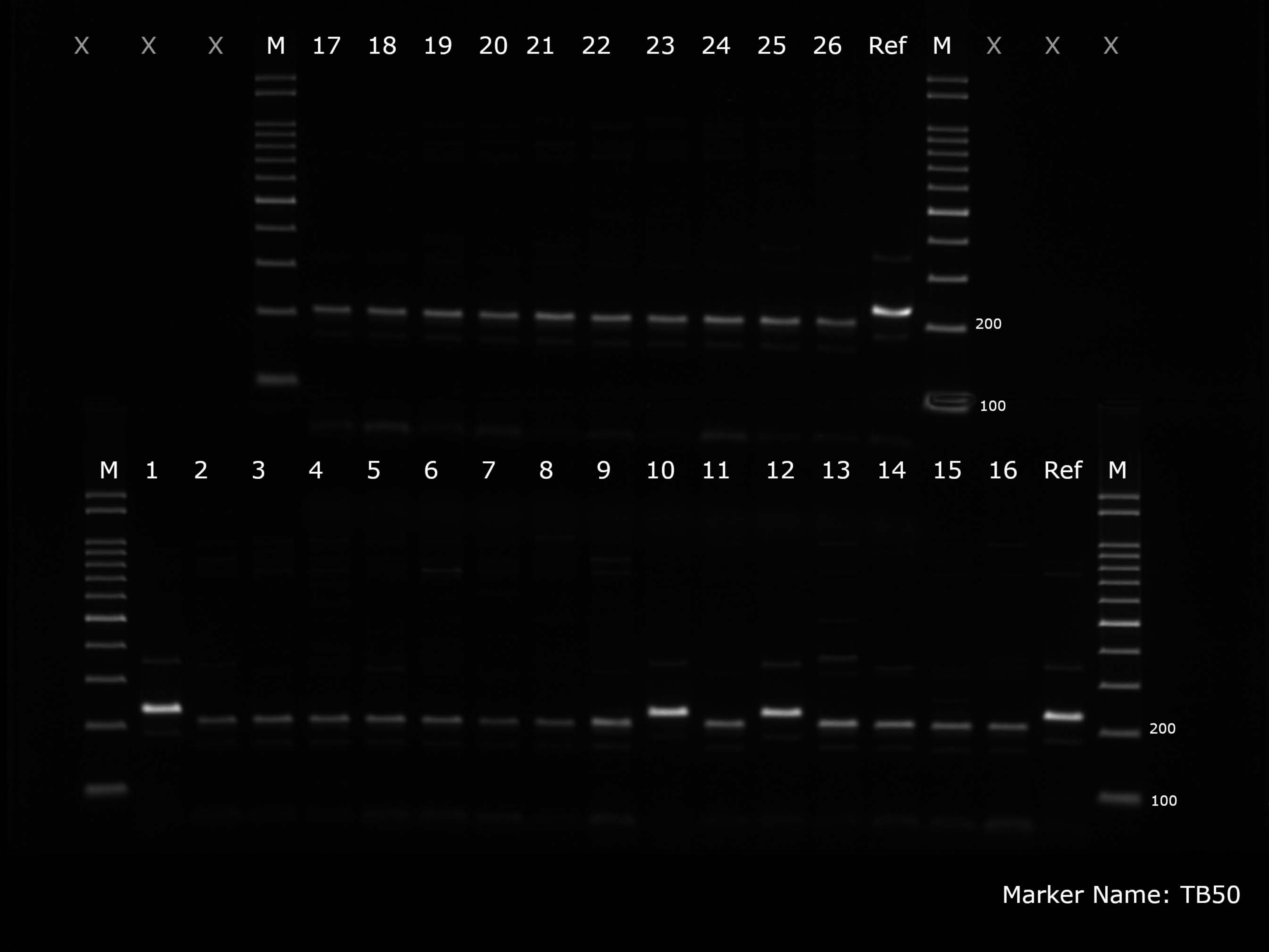
X X X M 17 18 19 20 21 22 23 24 25 26 Ref M X X X
200
100
M 1 2 3 4 5 6 7 8 9 10 11 12 13 14 15 16 Ref M
200
100
Marker Name: TB50

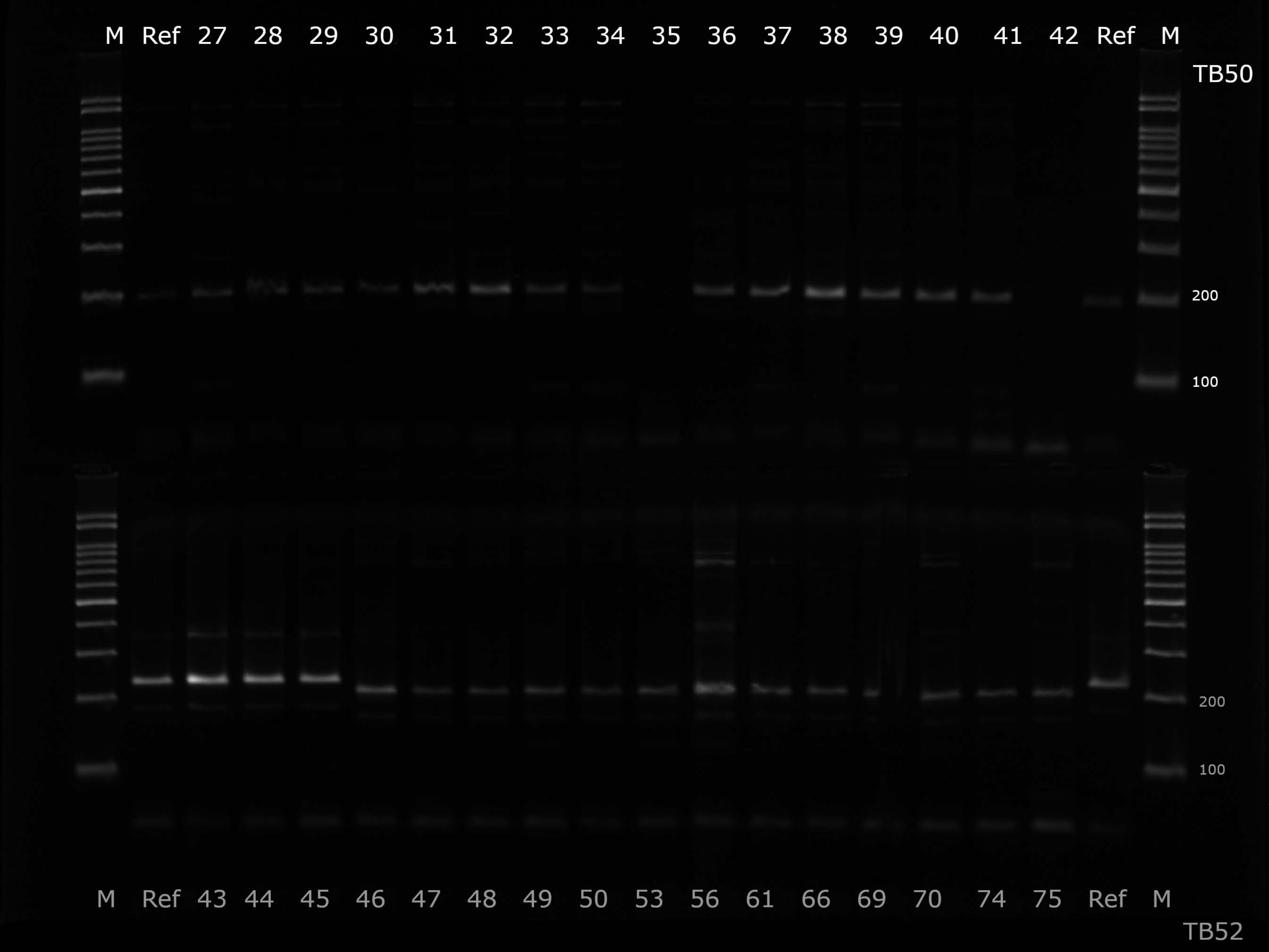
M Ref 27 28 29 30 31 32 33 34 35 36 37 38 39 40 41 42 Ref M
TB50
200
100
200
100
M Ref 43 44 45 46 47 48 49 50 53 56 61 66 69 70 74 75 Ref M
TB52

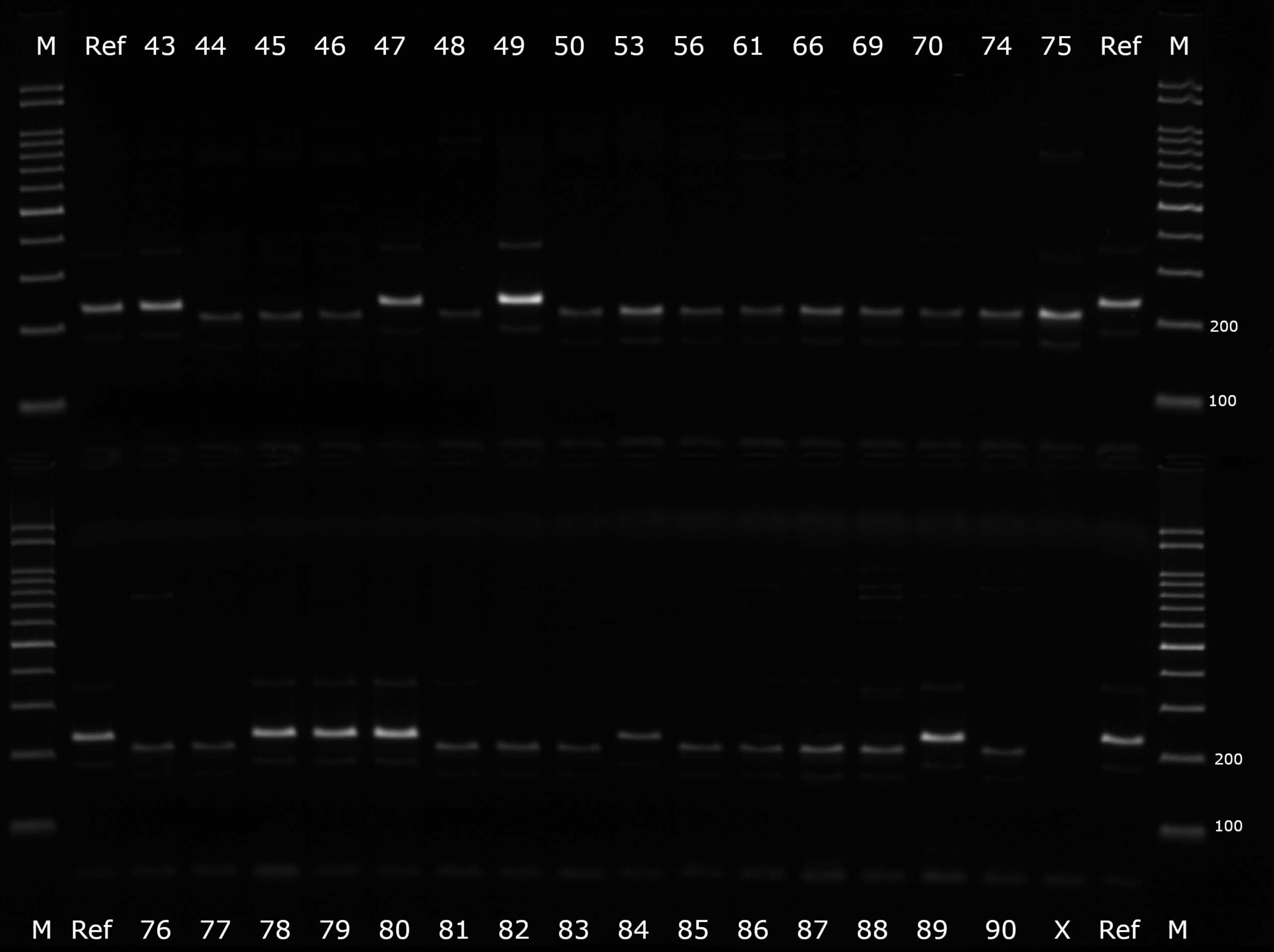

Marker Name: TB50
