# Supplementary material for: Development of 50 InDel-based barcode system for genetic identification of tartary buckwheat resources
Source: PLoS One. 2021 Jun 3;16(6):e0250786. doi: 10.1371/journal.pone.0250786 (PMC8174720; doi:10.1371/journal.pone.0250786)
Supplement: S2 Table — (DOCX) [file pone.0250786.s005.docx]

**S2 Table**. A gradient UPLC method for the simultaneous determination of rutin and quercetin

| T[min] | % A (1% formic acid in water) | % B (0.1% formic acid in ACN) |
| --- | --- | --- |
| 0 | 93 | 7 |
| 2 | 93 | 7 |
| 11 | 83 | 17 |
| 13 | 75 | 25 |
| 19 | 75 | 25 |
| 21 | 93 | 7 |
| 23 | 93 | 7 |
