# Supplementary material for: Development of 50 InDel-based barcode system for genetic identification of tartary buckwheat resources
Source: PLoS One. 2021 Jun 3;16(6):e0250786. doi: 10.1371/journal.pone.0250786 (PMC8174720; doi:10.1371/journal.pone.0250786)
Supplement: S3 Table — (DOCX) [file pone.0250786.s006.docx]

**S3 Table**. **Statistics of filtered raw data for evaluating Illumina paired read quality in 26 tartary buckwheat accessions.**

| Sample ID | Total reads^a^ | Clean reads  (%)^b^ | Total bases  (bp)^c^ | Clean bases  (%)^d^ | Mapped reads  (%)^e^ | Average  depth^f^ |
| --- | --- | --- | --- | --- | --- | --- |
| HLB1001 | 164099694 | 155361196  (94.67) | 16574069094 | 15572610886  ( 93.96) | 138151129  (94.8) | 24.28 |
| HLB1002 | 186094746 | 175797832  (94.47) | 18795569346 | 17615776752  (93.72) | 151555634  (93.64) | 26.6 |
| HLB1003 | 159180372 | 151333186  (95.07) | 16077217572 | 15174458974  (94.38) | 132327270  (94.00) | 23.25 |
| HLB1004 | 164775812 | 154431224  (93.72) | 16642357012 | 15455115671  (92.87) | 135595511  (93.63) | 23.78 |
| HLB1005 | 186932666 | 175495042  (93.88) | 18880199266 | 17569195364  (93.06) | 150573110  (93.36) | 26.4 |
| HLB1006 | 178599736 | 168296360  (94.23) | 18038573336 | 16846326001  (93.39) | 149310147  (94.53) | 26.2 |
| HLB1007 | 172747910 | 161823932  (93.68) | 17447538910 | 16185848703  (92.77) | 140302749  (92.99) | 24.58 |
| HLB1008 | 168618356 | 158710512  (94.12) | 17030453956 | 15882813280  (93.26) | 138511711  (93.47) | 24.28 |
| HLB1009 | 193924662 | 182102182  (93.9) | 19586390862 | 18226383114  (93.06) | 155750516  (93.21) | 27.3 |
| HLB1010 | 172725126 | 162878792  (94.3) | 17445237726 | 16296950440  (93.42) | 140792846  (93.64) | 24.68 |
| HLB1011 | 178054766 | 167616376  (94.14) | 17983531366 | 16767177469  (93.24) | 147684921  (94.22) | 25.89 |
| HLB1012 | 176788766 | 165602604  (93.67) | 17855665366 | 16560451108  (92.75) | 147114644  (94.57) | 25.79 |
| HLB1013 | 169439400 | 159547168  (94.16) | 17113379400 | 15957012345  (93.24) | 139463514  (93.92) | 24.44 |
| HLB1014 | 178269818 | 167554754  (93.99) | 18005251618 | 16752790826  (93.04) | 146930552  (94.06) | 25.74 |
| HLB1015 | 175551290 | 165240678  (94.13) | 17730680290 | 16536039708  (93.26) | 145552229  (94.19) | 25.53 |
| HLB1016 | 172208468 | 161976258  (94.06) | 17393055268 | 16207651023  (93.18) | 142193285  (94.01) | 24.93 |
| HLB1017 | 176795856 | 166406098  (94.12) | 17856381456 | 16651104050  (93.25) | 146341170  (94.18) | 25.66 |
| HLB1018 | 176677004 | 165112744  (93.45) | 17844377404 | 16498464317  (92.46) | 141138829  (93.14) | 24.69 |
| HLB1019 | 173016342 | 162920726  (94.16) | 17474650542 | 16310573893  (93.34) | 143226146  (94.23) | 25.13 |
| HLB1020 | 159435618 | 150468814  (94.38) | 16102997418 | 15054329635  (93.49) | 135115026  (94.89) | 23.7 |
| HLB1021 | 175879534 | 166026208  (94.4) | 17763832934 | 16620034696  (93.56) | 143797566  (93.7) | 25.21 |
| HLB1022 | 167386680 | 157505484  (94.1) | 16906054680 | 15777126558  (93.32) | 139219783  (94.35) | 24.44 |
| HLB1023 | 186724518 | 175871574  (94.19) | 18859176318 | 17613717847  (93.4) | 153781722  (94.05) | 26.99 |
| HLB1024 | 162170430 | 152781158  (94.21) | 16379213430 | 15302156544  (93.42) | 136118375  (94.56) | 23.9 |
| HLB1025 | 175471624 | 165242374  (94.17) | 17722634024 | 16546359223  (93.36) | 147283911  (94.6) | 25.85 |
| HLB1026 | 184489214 | 173450578  (94.02) | 18633410614 | 17359212292  (93.16) | 151985106  (93.98) | 26.65 |

^a^ Total reads: The total number of reads generated.

^b^ The number of reads after trimming with Sickle; (%) = No. of clean reads / No. of total reads.

^c^ Total bases: The total bases of total reads.

^d^ Total bases of clean reads; (%) = No. of clean bases/No. of total bases.

^e^ The number of reads mapped to the reference using BWA mapping tool; (%) = No. of mapped reads / No. of clean reads.

^f^ Average Depth: Mean coverage of all baits in the experiment.
