# Supplementary material for: Development of 50 InDel-based barcode system for genetic identification of tartary buckwheat resources
Source: PLoS One. 2021 Jun 3;16(6):e0250786. doi: 10.1371/journal.pone.0250786 (PMC8174720; doi:10.1371/journal.pone.0250786)
Supplement: S4 Table — (DOCX) [file pone.0250786.s007.docx]

**S4 Table**. **Number of InDels showing alternative genotype in 26 re-sequenced genomes**

| Sample ID | No. of homogenous InDels^a^ | No. of heterogenous InDels^b^ | No. of total InDels^c^ |
| --- | --- | --- | --- |
| HLB1001 | 967018 | 93567 | 1060585 |
| HLB1002 | 1005267 | 101871 | 1107138 |
| HLB1003 | 991512 | 95614 | 1087126 |
| HLB1004 | 998275 | 99067 | 1097342 |
| HLB1005 | 1002356 | 101339 | 1103695 |
| HLB1006 | 1005397 | 101341 | 1106738 |
| HLB1007 | 1008541 | 99702 | 1108243 |
| HLB1008 | 1009309 | 99566 | 1108875 |
| HLB1009 | 1011610 | 102273 | 1113883 |
| HLB1010 | 989688 | 94575 | 1084263 |
| HLB1011 | 1003414 | 101853 | 1105267 |
| HLB1012 | 995975 | 97985 | 1093960 |
| HLB1013 | 998260 | 99467 | 1097727 |
| HLB1014 | 995261 | 113604 | 1108865 |
| HLB1015 | 977278 | 155750 | 1133028 |
| HLB1016 | 984430 | 142252 | 1126682 |
| HLB1017 | 1005979 | 102307 | 1108286 |
| HLB1018 | 1008990 | 100707 | 1109697 |
| HLB1019 | 1002355 | 101677 | 1104032 |
| HLB1020 | 1000579 | 99496 | 1100075 |
| HLB1021 | 1008552 | 100153 | 1108705 |
| HLB1022 | 996111 | 97976 | 1094087 |
| HLB1023 | 1008224 | 102824 | 1111048 |
| HLB1024 | 998055 | 98062 | 1096117 |
| HLB1025 | 1000075 | 101226 | 1101301 |
| HLB1026 | 997547 | 122173 | 1119720 |

^a^ The number of variats homogeneous to alternative genotype; both alleles are same to alternative genotype.

^b^ The number of variants heterogeneous genotype; one allele is same to reference, and another to alternative genotype.

^C^ Total sum of homogenous and heterogeneous variants; not counting multi-allelic variants.
