# Supplementary material for: Development of 50 InDel-based barcode system for genetic identification of tartary buckwheat resources
Source: PLoS One. 2021 Jun 3;16(6):e0250786. doi: 10.1371/journal.pone.0250786 (PMC8174720; doi:10.1371/journal.pone.0250786)
Supplement: S7 Table — (DOCX) [file pone.0250786.s010.docx]

**S7 Table. Statistics of the 50 InDels in tartary buckwheat accessions (n=73)**

| Marker | No of Scaffold | Major Allele Frquency | Gene Diversity | Heterozygosity | PIC |
| --- | --- | --- | --- | --- | --- |
| TB01 | Scaffold 10 | 0.9041 | 0.1734 | 0 | 0.1584 |
| TB02 | Scaffold 83 | 0.8088 | 0.3100 | 0 | 0.2620 |
| TB03 | Scaffold 38 | 0.7397 | 0.3851 | 0 | 0.3109 |
| TB04 | Scaffold 311 | 0.9863 | 0.0270 | 0 | 0.0267 |
| TB05 | Scaffold 813 | 0.9726 | 0.0533 | 0 | 0.0519 |
| TB06 | Scaffold 49 | 0.7397 | 0.3851 | 0 | 0.3109 |
| TB07 | Scaffold 58 | 0.8630 | 0.2364 | 0 | 0.2085 |
| TB08 | Scaffold 331 | 0.7260 | 0.3978 | 0 | 0.3187 |
| TB09 | Scaffold 4 | 0.6986 | 0.4211 | 0 | 0.3324 |
| TB10 | Scaffold 7 | 0.9041 | 0.1734 | 0 | 0.1584 |
| TB11 | Scaffold 57 | 0.8356 | 0.2747 | 0 | 0.2370 |
| TB12 | Scaffold 5 | 0.8219 | 0.2927 | 0 | 0.2499 |
| TB13 | Scaffold 11 | 0.6301 | 0.4661 | 0 | 0.3575 |
| TB14 | Scaffold 77 | 0.7260 | 0.3978 | 0 | 0.3189 |
| TB15 | Scaffold 223 | 0.7260 | 0.3978 | 0 | 0.3187 |
| TB16 | Scaffold 137 | 0.7055 | 0.4156 | 0.0137 | 0.3292 |
| TB17 | Scaffold 32 | 0.8767 | 0.2162 | 0 | 0.1928 |
| TB18 | Scaffold 320 | 0.7671 | 0.3573 | 0 | 0.2935 |
| TB19 | Scaffold 145 | 0.9726 | 0.0533 | 0 | 0.0519 |
| TB20 | Scaffold 1 | 0.9863 | 0.0270 | 0 | 0.0267 |
| TB21 | Scaffold 1 | 0.9658 | 0.0661 | 0.0137 | 0.0640 |
| TB22 | Scaffold 1 | 0.9589 | 0.0788 | 0 | 0.0758 |
| TB23 | Scaffold 4 | 0.9452 | 0.1036 | 0 | 0.0982 |
| TB24 | Scaffold 4 | 0.9589 | 0.0788 | 0 | 0.0757 |
| TB25 | Scaffold 8 | 0.9863 | 0.0270 | 0 | 0.0267 |
| TB26 | Scaffold 16 | 0.9589 | 0.0788 | 0 | 0.0757 |
| TB27 | Scaffold 22 | 0.9863 | 0.0270 | 0 | 0.0267 |
| TB28 | Scaffold 34 | 0.9863 | 0.0270 | 0 | 0.0267 |
| TB29 | Scaffold 34 | 0.8082 | 0.3100 | 0 | 0.2620 |
| TB30 | Scaffold 37 | 0.9589 | 0.0788 | 0 | 0.0757 |
| TB31 | Scaffold 42 | 0.9863 | 0.0270 | 0 | 0.0267 |
| TB32 | Scaffold 42 | 0.9041 | 0.1734 | 0 | 0.1584 |
| TB33 | Scaffold 44 | 0.9658 | 0.0661 | 0.0137 | 0.0640 |
| TB34 | Scaffold 70 | 0.9589 | 0.0788 | 0 | 0.0757 |
| TB35 | Scaffold 87 | 0.9726 | 0.0533 | 0 | 0.0519 |
| TB36 | Scaffold 88 | 0.8904 | 0.1952 | 0 | 0.1761 |
| TB37 | Scaffold 100 | 0.9863 | 0.0270 | 0 | 0.0267 |
| TB38 | Scaffold 118 | 0.9863 | 0.0270 | 0 | 0.0267 |
| TB39 | Scaffold 168 | 0.9452 | 0.1036 | 0 | 0.0982 |
| TB40 | Scaffold 169 | 0.9178 | 0.1509 | 0 | 0.1395 |
| TB41 | Scaffold 197 | 0.8082 | 0.3100 | 0 | 0.2620 |
| TB42 | Scaffold 207 | 0.5616 | 0.4924 | 0 | 0.3712 |
| TB43 | Scaffold 209 | 0.9452 | 0.1036 | 0 | 0.0982 |
| TB44 | Scaffold 230 | 0.9863 | 0.0270 | 0 | 0.0267 |
| TB45 | Scaffold 307 | 0.9726 | 0.0533 | 0 | 0.0519 |
| TB46 | Scaffold 365 | 0.9315 | 0.1276 | 0 | 0.1195 |
| TB47 | Scaffold 488 | 0.9315 | 0.1276 | 0 | 0.1195 |
| TB48 | Scaffold 9 | 0.9863 | 0.0270 | 0 | 0.0267 |
| TB49 | Scaffold 60 | 0.9041 | 0.1734 | 0 | 0.1584 |
| TB50 | Scaffold 61 | 0.8082 | 0.3100 | 0 | 0.2620 |
| Mean |  | 0.8850 | 0.1798 | 0.0008 | 0.1532 |
