# Supplementary material for: Development of 50 InDel-based barcode system for genetic identification of tartary buckwheat resources
Source: PLoS One. 2021 Jun 3;16(6):e0250786. doi: 10.1371/journal.pone.0250786 (PMC8174720; doi:10.1371/journal.pone.0250786)
Supplement: Attachment — Submitted filename: PONE-D-20-30493_R1_Edit.pdf [file pone.0250786.s013.pdf]

PLOS ONE

Development of 50 InDel-based barcode system for genetic identification of tartary buckwheat resources

--Manuscript Draft--

**Manuscript Number:**	PONE-D-20-30493R1
**Article Type:**	Research Article
**Full Title:**	Development of 50 InDel-based barcode system for genetic identification of tartary buckwheat resources
**Short Title:**	Identification of tartary buckwheat resources
**Corresponding Author:**	Yul-Ho Kim<br>National Institute of Crop Science<br>Pyeongchang-Gun, Gangwon KOREA, REPUBLIC OF
**Keywords:**	Tartary buckwheat; rutin; InDel; barcode system; genetic diversity
**Abstract:**	Tartary buckwheat (Fagopyrum tataricum Gartn.) is a highly functional crop that is poised to be the target of many future breeding efforts. The reliable ex situ conservation of various genetic resources is essential for the modern breeding of tartary buckwheat varieties. We developed PCR-based ~~and~~ co-dominant insertion/deletion (InDel) markers to discriminate tartary buckwheat genetic resources. First, we obtained the whole genome from 26 accessions across a superscaffold-scale reference genome of 569.37 Mb for tartary buckwheat cv. “Daegwan 3-7.” Next, 171,926 homogeneous and 53,755 heterogeneous InDels were detected by comparing 26 accessions with the “Daegwan 3-7” reference sequence. Of these, 100 candidate InDels ranging from 5–20 bp in length were chosen for validation, and 50 of them revealed polymorphisms between the 26 accessions and “Daegwan 3-7.” The validated InDels were further tested through the assessment of their likelihood to give rise to a single or a few PCR products in 50 other accessions, covering most tartary buckwheat genome types. The major allele frequencies ranged from 0.5616 at the TB42 locus to 0.9863 at the TB48 locus, with the average PIC value of 0.1532 with a range of 0.0267–0.3712. To create a user-friendly system, the homology of the genotypes between and among the accessions were visualized in both one- (1D) and two-dimensional (2D) barcode types by comparing amplicon polymorphisms with the reference variety, “Daegwan 3-7.” A phylogenetic tree and population structure of the 76 accessions according to amplicon polymorphisms for the 50 InDel markers corresponded to those using non-synonymous single nucleotide polymorphism variants, indicating that the barcode system based on the 50 InDels was a useful tool to improve the reliability of identification of tartary buckwheat accessions in the germplasm stocks.
**Order of Authors:**	Hwang-Bae Sohn
	Su-Jeong Kim
	Su-Young Hong
	Sin-Gi Park
	Dong-Ha Oh
	Sunghoon Lee
	Hwa Yeun Nam
	Jung Hwan Nam
	Yul-Ho Kim
**Response to Reviewers:**	We greatly appreciate the reviewer’s efforts to carefully review our manuscript and the valuable suggestions offered, which were invaluable in helping us to make improvements. We have revised and modified our manuscript to clarify and strengthen its content, after taking into consideration all of the reviewers’ comments. Please find our responses to each of the reviewers’ comments in the uploaded file.

Powered by Editorial Manager® and ProduXion Manager® from Aries Systems Corporation

Additional Information:	
**Question**	**Response**
**Financial Disclosure**<br><br>Enter a financial disclosure statement that describes the sources of funding for the work included in this submission. Review the submission guidelines for detailed requirements. View published research articles from *PLOS ONE* for specific examples.<br><br>This statement is required for submission and **will appear in the published article** if the submission is accepted. Please make sure it is accurate.<br><br>**Unfunded studies**<br>Enter: *The author(s) received no specific funding for this work.*<br><br>**Funded studies**<br>Enter a statement with the following details:<br>• Initials of the authors who received each award<br>• Grant numbers awarded to each author<br>• The full name of each funder<br>• URL of each funder website<br>• Did the sponsors or funders play any role in the study design, data collection and analysis, decision to publish, or preparation of the manuscript?<br>• **NO** - Include this sentence at the end of your statement: *The funders had no role in study design, data collection and analysis, decision to publish, or preparation of the manuscript.*<br>• **YES** - Specify the role(s) played.<br><br>* typeset	This study was financially supported by grants from Co-operative Research Program (Project No. PJ01189401) for Agriculture Science and Technology Development funded by the Rural Development Administration, Republic of Korea.
**Competing Interests**<br><br>Use the instructions below to enter a competing interest statement for this submission. On behalf of all authors, disclose any competing interests that could be perceived to bias this work—acknowledging all financial support and any other relevant financial or non-financial competing interests.	The authors have declared that no competing interests exist.

Powered by Editorial Manager® and ProduXion Manager® from Aries Systems Corporation

This statement **will appear in the published article** if the submission is accepted. Please make sure it is accurate. View published research articles from *PLOS ONE* for specific examples.<br><br>**NO authors have competing interests**<br><br>Enter: *The authors have declared that no competing interests exist.*<br><br>**Authors with competing interests**<br><br>Enter competing interest details beginning with this statement:<br><br>*I have read the journal's policy and the authors of this manuscript have the following competing interests: [insert competing interests here]*<br><br>* typeset	
**Ethics Statement**<br><br>Enter an ethics statement for this submission. This statement is required if the study involved:<br><br>• Human participants<br>• Human specimens or tissue<br>• Vertebrate animals or cephalopods<br>• Vertebrate embryos or tissues<br>• Field research<br><br>Write "N/A" if the submission does not require an ethics statement.<br><br>General guidance is provided below. Consult the submission guidelines for detailed instructions. **Make sure that all information entered here is included in the Methods section of the manuscript.**	N/A

Powered by Editorial Manager® and ProduXion Manager® from Aries Systems Corporation

**Format for specific study types**<br><br>**Human Subject Research (involving human participants and/or tissue)**<br>• Give the name of the institutional review board or ethics committee that approved the study<br>• Include the approval number and/or a statement indicating approval of this research<br>• Indicate the form of consent obtained (written/oral) or the reason that consent was not obtained (e.g. the data were analyzed anonymously)<br><br>**Animal Research (involving vertebrate animals, embryos or tissues)**<br>• Provide the name of the Institutional Animal Care and Use Committee (IACUC) or other relevant ethics board that reviewed the study protocol, and indicate whether they approved this research or granted a formal waiver of ethical approval<br>• Include an approval number if one was obtained<br>• If the study involved *non-human primates*, add *additional details* about animal welfare and steps taken to ameliorate suffering<br>• If anesthesia, euthanasia, or any kind of animal sacrifice is part of the study, include briefly which substances and/or methods were applied<br><br>**Field Research**<br><br>Include the following details if this study involves the collection of plant, animal, or other materials from a natural setting:<br>• Field permit number<br>• Name of the institution or relevant body that granted permission	
**Data Availability**<br><br>Authors are required to make all data underlying the findings described fully available, without restriction, and from the time of publication. PLOS allows rare exceptions to address legal and ethical concerns. See the PLOS Data Policy and FAQ for detailed information.	Yes - all data are fully available without restriction

A Data Availability Statement describing where the data can be found is required at submission. Your answers to this question constitute the Data Availability Statement and **will be published in the article**, if accepted.<br><br>**Important:** Stating 'data available on request from the author' is not sufficient. If your data are only available upon request, select 'No' for the first question and explain your exceptional situation in the text box.<br><br>Do the authors confirm that all data underlying the findings described in their manuscript are fully available without restriction?	
**Describe where the data may be found in full sentences. If you are copying our sample text, replace any instances of XXX with the appropriate details.**<br><br>• If the data are **held or will be held in a public repository**, include URLs, accession numbers or DOIs. If this information will only be available after acceptance, indicate this by ticking the box below. For example: *All XXX files are available from the XXX database (accession number(s) XXX, XXX.*).<br>• If the data are all contained **within the manuscript and/or Supporting Information files**, enter the following: *All relevant data are within the manuscript and its Supporting Information files.*<br>• If neither of these applies but you are able to provide **details of access elsewhere**, with or without limitations, please do so. For example:<br><br>*Data cannot be shared publicly because of [XXX]. Data are available from the XXX Institutional Data Access / Ethics Committee (contact via XXX) for researchers who meet the criteria for access to confidential data.*<br><br>*The data underlying the results presented in the study are available from (include the name of the third party*	All relevant data are within the manuscript and its Supporting Information files.

and contact information or URL).<br>• This text is appropriate if the data are owned by a third party and authors do not have permission to share the data.<br><br>* typeset	
Additional data availability information:	

Powered by Editorial Manager® and ProduXion Manager® from Aries Systems Corporation

Manuscript Click here to access/download;Manuscript;Manuscript.docx

Development of 50 InDel-based barcode system for genetic identification of tartary buckwheat resources

Short title: Identification of tartary buckwheat resources

Hwang-Bae Sohn[1¶], Su-Jeong Kim[1¶], Su-Young Hong[1], Sin-Gi Park[2], Dong-Ha Oh[3], Sunghoon Lee[4], Hwa-Yeun Nam[1], Jung-Hwan Nam[1], Yul-Ho Kim[1*]

[1]Highland Agriculture Research Institute, National Institute of Crop Science, Pyeongchang, Gangwon-do, Republic of Korea

[2]TheragenEtex Bio Institute, TherageneEtex Inc., Suwon, Gyeonggi-do, Republic of Korea

[3]Department of Biological Science, Louisiana State University, Baton Rouge, LA, United States of America

[4]EONE-DIAGNOMICS Genome Center Co. Ltd., Incheon, Republic of Korea

* Corresponding author

E-mail: kimyuh77@korea.kr (YHK)

¶ These authors contributed equally to this work

Abstract

Tartary buckwheat (*Fagopyrum tataricum* Gartn.) is a highly functional crop that is poised to be the target of many future breeding efforts. The reliable *ex situ* conservation of various genetic resources is essential for the modern breeding of tartary buckwheat varieties. We developed PCR-based ~~and~~ co-dominant insertion/deletion (InDel) markers to discriminate tartary buckwheat genetic resources. First, we obtained the whole genome from 26 accessions across a superscaffold-scale reference genome of 569.37 Mb for tartary buckwheat cv. "Daegwan 3-7." Next, 171,926 homogeneous and 53,755 heterogeneous InDels were detected by comparing 26 accessions with the "Daegwan 3-7" reference sequence. Of these, 100 candidate InDels ranging from 5–20 bp in length were chosen for validation, and 50 of them revealed polymorphisms between the 26 accessions and "Daegwan 3-7." The validated InDels were further tested through the assessment of their likelihood to give rise to a single or a few PCR products in 50 other accessions, covering most tartary buckwheat genome types. The major allele frequencies ranged from 0.5616 at the TB42 locus to 0.9863 at the TB48 locus, with the average PIC value of 0.1532 with a range of 0.0267–0.3712. To create a user-friendly system, the homology of the genotypes between and among the accessions were visualized in both one- (1D) and two-dimensional (2D) barcode types by comparing amplicon polymorphisms with the reference variety, "Daegwan 3-7." A phylogenetic tree and population structure of the 76 accessions according to amplicon polymorphisms for the 50 InDel markers corresponded to those using non-synonymous single nucleotide polymorphism variants, indicating that the barcode system based on the 50 InDels was a useful tool to improve the reliability of identification of tartary buckwheat accessions in the germplasm stocks.

Introduction

The eudicot family Polygonaceae consists of 27 reported species, including the 2 cultivated species, common buckwheat (*Fagopyrum esculentum* Moench) and tartary buckwheat (*F. tataricum* Gaert.) [1,2]. Buckwheats (*F. esculentum* and *F. tataricum*) are cultivated, as short-season pseudo-cereals, in many countries and regions, including China, Nepal, Russia, Europe, Korea, and Japan [3, 4]. Following domestication in upland southwestern China, a multitude of tartary buckwheat landraces were created by ancient farmers through both artificial and natural selection [5]. After cultivation in China, these landraces were first dispersed to Nepal, Russia, Europe, Korea, and Japan [5-9]. Several characteristics of tartary buckwheat, such as its self-pollinating nature and ability to adapt to various regions (ranging from high altitude zones to lowland), led to both morphology and genetically divergent pockets of landraces [10-13]. These landraces have been largely replaced by fewer landraces with specific desired characteristics such as high-yield, drought resistance, and flavor [5,14].

Tartary buckwheat is a major food crop in high altitude zones (e.g., high

mountain areas of southern China and the Himalayan hills) due to its frost tolerance under poor soil conditions. Tartary buckwheat has also been used more recently for a variety of purposes, ranging from various transitional food products to medical use [15,16]. The grain of tartary buckwheat is considered to be an ideal functional food source for humans because it is richer in proteins, fats, vitamins, rutin, quercetin, and other flavonoids than common buckwheat [17-23]. The popularity of tartary buckwheat has been steadily increasing, and its cultivation has broadened into areas far outside its original region [10, 11,24,25]. This is a testament to tartary buckwheat's adaptability, which will be crucial in Earth's changing climate [14]. Breeding for commercial varieties of tartary buckwheat improves agricultural traits, such as lodging, late maturity, and low yield [26,27]; however, this cultivation resulted from a narrow genetic base from a few varieties only, which have produced modern varieties that are not well adapted to a variety of growing environment. The narrow genetic diversity within a commercial variety is due to the rigid quality required by farmers and processors, the finite use of exotic germplasm, restricted breeding strategies, and individual plant selection [28-30]. Thus, more genetic variation among tartary buckwheat genotypes could be used to improve a specific trait of interest due to the narrow genetic diversity of modern commercial varieties [14,31,32].

The identification of tartary buckwheat germplasms is vital to the enhancement of genetic diversity. Researchers have been focused on developing molecular markers using germplasms and morphological descriptors to provide high discrimination power. There are many methods for variety discrimination based on DNA polymorphisms, such as random amplification of polymorphic DNA (RAPD), amplified fragment length polymorphism (AFLP), inter-simple sequence repeat (ISSR), simple sequence repeats (SSRs), and sing nucleotide polymorphism (SNP). Insertion-deletion polymorphisms (InDels) are gaining more attention among molecular breeding scientists because they are easy to use, co-dominant (fully informative), and relatively abundant [33-40]. In tartary buckwheat, SSR [9], AFLP [41], RAPD [42,43], ISSR [44] and SNP [45,46] have been used to assess genetic variation, but no study has used a variety of discrimination methods with InDel markers due to the difficulty of finding DNA polymorphisms in tartary buckwheat accessions.

Until recently, a lack of large-scale genome sequencing information made the introduction of InDels as genetic markers in non-model species (e.g., common and tartary buckwheat) difficult. In recent years, published studies have provided the draft genomes of common buckwheat [47], and a tartary buckwheat cultivar "Pinku1" [48] in the family Polygonaceae. More importantly, a wealth of polymorphism data can now be obtained from a massive amount of sequencing data using high-throughput sequencing technologies [49]. In addition, [38] reported that the InDel-based barcode system focuses on usability, and provided an efficient resource management system. In addition to the InDel-based barcode system, morphological descriptors are now emerging that aim to identify tartary buckwheat accessions, which can be used to verify key genomic factors that explain or predict major agronomic traits.

We present a high-quality draft genome assembly and annotation for the tartary

buckwheat. *F. tataricum* cv. “Daegwan 3-7.” Using comparative genomics approaches, we revealed 171,926 homogeneous InDels and 53,755 heterogeneous InDels found in 26 tartary buckwheat accessions, which we compared to the draft genomes of the tartary buckwheat cultivar “Daegwan 3-7.” Among them, 50 polymorphic InDels from the 26 accessions were selected by gel electrophoresis, which were converted to barcodes by comparing amplicon polymorphisms with the reference sequence. We also revealed the geographical distribution of 73 accessions through population structure analysis using the barcodes. For the genetic identification of tartary buckwheat resources, we made a user-friendly barcode system based on 50 InDel-based genotypes of 73 accessions. As a user-friendly system, the homology between the accessions can be predicted in both one (1D) and two-dimensions (2D) as blocks. Our platform could be used in genetic research and breeding programs, as well as for efficient resource management systems for tartary buckwheat.

Materials and methods

Plant Materials

The accessions of 73 tartary buckwheat plants (S1 Table) were provided by the National Agrobiodiversity Center (Jeonju, Republic of Korea) and the Highland Agriculture Research Institute (Pyeongchang, Republic of Korea). The accessions were planted in a greenhouse located at the Highland Agriculture Research Institute (37°68′N and 128°73′E, 779 m altitude), Gangwon Province, Republic of Korea. These were self-pollinated 3 times by the single seed descent (SSD) method by growing the individual plants in flowerpots (10cm in diameter) [50]. We grew an additional four generations of HLB1001 using the SSD method, which resulted in the selection of a progeny, “Daegwan 3-7” for a draft genome of tartary buckwheat. A total of 26 accessions were collected (China, India, Nepal, Bhutan, Pakistan, USA).

To investigate the biodiversity and comparative relationships between the accessions, collected accessions were sown in 2017 and grown in the greenhouse at the Highland Agriculture Research Institute. We separated the plants by developmental stage: the vegetative growth stage (1–40 days after planting, DAP), flowering stage (40–80 DAP), and yield stage (80-90 DAP). At the yield stage, the color of the seed coat turned black, indicating that the seeds were completely maturated.

Measurement of rutin and quercetin content

The yield stage seeds were used to measure their rutin and quercetin content. First, the mature seeds from the 76 accessions were milled into a fine powder, then the samples were immediately flash-frozen in liquid nitrogen and stored at −80°C until further use. A total of 0.10 g dry powder for each sample was mixed with 1 ml methanol, then was extracted at 80°C for 1 hr in a conventional Soxhlet apparatus. The extract was filtered through a 0.20 μm syringe filter (PTFE 13 mm, PALL Life Sciences, Ann Arbor, MI) for rutin content [23]. Ultra-performance liquid chromatography (Waters Corporation, Milford, MA, USA) was performed on an Acquity 1-class using a C18 column (2.1 mm × 100 mm, 1.7 μm) (Waters Corporation, USA) at 30°C. The mobile phase (1% formic acid in water/0.1% formic acid in acetonitrile) flowed by the gradient elution method (S2 Table) at a flow rate of 0.25 ml/min with a total injection volume of 10 $\mu\ell$. Rutin quantity was estimated based on the linear calibration curve of standard rutin and quercetin (Extrasynthese, France) under a detection wavelength of 259 nm. Three independent sample analyses were performed for each sample. All statistical analyses were performed using ver. 3.6.1 of the R software.

DNA sequencing and *de novo* assembly

After 21 DAP, the leaves in the third node of the progeny “Daegwan 3-7” were used to create a draft genome. First, 3-week-old leaves from the bulk of 10 plants were collected for genomic DNA extraction using a standard CTAB (cetyl trimethylammonium bromide) protocol [52, 53]. Both short read (Illumina) and long read (PacBio) libraries were prepared according to the manufacturer’s instructions for the entire genome of *F. tataricum*. A short insert paired-end (PE) library was prepared using a Illumina TruSeq Nano DNA Sample Preparation Kit (Illumina Inc., San Diego, Ca, USA) and 1~3 μg of DNA. A library profile analysis was performed with an Agilent 2100 Bioanalyzer (Agilent Technologies, USA) and qPCR quantification, then the libraries were sequenced to 2 × 100 bp on the Illumina HiSeq 2500 platform (Illumina, USA) from the TheragenEtex Bio Institute (TheragenEtex Inc., Suwon, Republic of Korea). PacBio SMRTbell libraries (2 kb, 5 kb, 10 kb, and 15 kb inserts) were prepared with the standard PacBio library preparation protocols, which are available at http://pacificbiosciences.com/. The sequencing was conducted on a PacBio RS II (Pacific Biosciences, USA) system using C4 chemistry and 240 min movies by the TheragenEtex Bio Institute.

The short and long reads were assembled separately. The de novo assembly of the PacBio long reads was performed using Fast Alignment and CONsensus [54] with default parameters. The short reads were assembled with SOAPdenovo2 [55] with default

parameters. The initial contigs were merged using HaploMerger2 [56]. Both short and long reads were then used to construct scaffolds with SSPACE [57], which led to the superscafold-scale draft genome of tartary buckwheat cv. “Daegwan 3-7.” The N50 length of the final genome assemblies was evaluated.

Identification and validation of the polymorphic InDels

Genomic DNA was extracted for whole-genome resequencing from the 26 accessions that had self-pollinated 3 times using the SSD method. Three-week-old leaves from the bulk of 10 plants were collected for genomic DNA extraction using a standard CTAB protocol [52, 53]. The data consisted of 101-bp reads, which were generated using the HiSeq 2500 sequencer. The Short Oligonucleotide Alignment Program 2 (SOAP2) was used to map the raw pair-end reads onto the reference genome (tartary buckwheat cv. “Daegwan 3-7”). For each accession, more than 93% of the reads were properly aligned to the reference genome. Insert size was estimated by mapping the reads to the reference genome using the Burrows-Wheeler Aligner algorithm (bwa) [58] ver 0.5.9. The aligned reads were realigned at InDel positions using the GATK InDelRealinger algorithm [59] enhance the mapping quality. The base quality scores were recalibrated using the GATK TableRecalibration algorithm. The primer pair used to amplify each of the InDels were selected by the Primer3 software (http://primer3.sourceforge.net). We selected InDels with more than 10 bp and designed the primers accordingly to match the characteristics of each InDel using the Primer 3 software.

Amplicon polymorphism assay and bar-coding process

PCR analysis was performed using 10 $\mu\ell$ reaction mixtures containing 20 ng of total genomic DNA, 2 pM of primer, and 5 $\mu\ell$ of GoTaq Green Master Mix (Promega, Madison, WI, USA). The PCR was performed at 95℃ for 5 min, followed by 35 subsequent rounds at 94℃ for 30 sec, 45°C for 30 sec and 72°C for 30 sec, using a Mastercycler pro 384 (Eppendorf, Germany). The PCR products were separated by electrophoresis in 3% gels of certified low range ultra-agarose (Bio-rad) followed by LoadingSTAR staining (Dynebio, Republic of Korea). For the limitation of the number of candidates, we chose primer pairs that amplified PCR products that were 150–231 bp long. The allelic diversity of the InDels with the e-PCR products in 26 tartary buckwheat genomes were assessed via PIC (Polymorphism Information Content), which was defined as PIC i =1-$\sum_{j=1}^{n} p_{ij}^{2}$, where p_{ij} is the frequency of the *j*th pattern for the *i*th marker [60,61].

The 50 InDel markers were selected according to their genotyping success and PCR band size. The discriminating power of the selected 50 InDel set for tartary buckwheat identification was evaluated using the 76 tartary buckwheat accessions. Primers were designed by targeting the InDel region in such a way that the genotypes of tartary buckwheat accessions would produce the same or different (insertion or deletion) amplicons relative to the reference genome, "Daegwan 3-7." Based on the results of InDel marker amplification, the same result with the reference genome was represented by an "a" while other results were represented by a "b." For bar-coding representation of the results, "a" and "b" were converted to white and black, respectively. The homology of the accessions were calculated after PCR amplification of all 50 InDels.

Phylogenetic analysis and population structure among 73 tartary buckwheat accessions

The discriminating power of the selected 50 InDel set for tartary buckwheat identification were evaluated using the 76 tartary buckwheat accessions. The analysis was carried out with POWERMARKER Ver. 3.23 (Liu and Muse 2005; http//www.poxermarker.net). A phylogenetic tree for the 73 accessions was drawn based on the genotypes defined with the 50 InDels using the weighted neighbor-joining method with simple matching coefficients in the DARwin software [55]. To delineate clusters of individuals on the basis of their genotypes, the model-based STRUCTURE 2.3.4 [63] was used with the admixture model, a burn-in period of 100,000 iterations, and data from the 50 InDel loci. The number of clusters (k) was set to five, as this number maximized the Δk (ad hoc criterion) parameter. We used simulations with k values ranging from 2 to 10 with 5 replications, to calculate the LnP(D) value. The optimal k value can be chosen based on the maximum LnP(D) that provides a distinct population structure. On this basis, we chose K = 3 (three ancestral groups) for the tartary buckwheat populations. To check the quality of these analyses, a phylogenetic tree and a population structure for the 26 re-sequenced accessions were drawn based on the genotypes defined using the 6,622 non-synonymous SNPs.

Results

Genome assembly and whole-genome sequencing of tartary buckwheat accessions

We grew seven generations of tartary buckwheat cv. “Daegwan” using the SSD method and an isolated individual (designated as “Daegwan 3-7”) to exclude any possible heterogeneity in the original seed lot. We produced a total of 43.83 and 32.17 Gb sequences from Illumina PE and Single-Molecule Real-Time (SMRT) sequencing platforms, respectively, which corresponded to 78x (Illumina PE, S2 Table) and 58x (SMRT, S3 Table) coverages (S1 Fig) for the reference genome. Combining the sequencing strategies of PacBio and Illumina Hiseq2500, we obtained a final draft assembly of 569.37 Mb in 2,886 scaffolds with 50% of the total sequence captured in 156 scaffolds larger than 886,968 bps (Table 1, N50).

Table 1. Summary of *Fagopyrum tataricum* draft genome assembly process.

Steps	Type	No. of Sequences	Total bases	Longest sequence	N50 sequence	N90 sequence
Illumina assembly	Contigs	37,711	416,936,361	318,774	29,297	5,269
PacBio assembly	Contigs	2,231	450,944,578	5,290,013	650,667	148,793
Hybrid assembly	Contigs	4,433	565,095,288	5,290,013	463,432	36,547
Scaffolding / Gap-filling	Scaffolds	2,886	569,372,063	5,875,542	798,933	61,611

We selected 26 tartary buckwheat accessions for whole-genome resequencing. These accessions originated or were popularized in different Asian and international regions, including 15 landraces from China, 4 from Nepal, 4 from India, 1 from the USA, 1 from Bhutan and 1 from Pakistan (Table 2). After 3 additional generations were grown using the SSD method, a panel of the 26 genotypes showed a broad extent of rutin content and phenotype variation (Figs 1 and 2). The 26 accessions showed a various distribution of rutin content compared to those of the 73 accessions (Fig 2). In particular, HLB1013 had the highest rutin content (1,921 mg/100g·DW), while HLB1006 had the lowest (1,250 mg/100g·DW) among the 26 accessions. Large differences were found in seed shape and seed coat color among the 26 accessions (Fig 1). Seed information conformed to the known the genetic variation of the 26 accessions.

Table 2.Genome coverage and mean depth after the whole genome resequencing of 26 accessions.

Genotype	IT Number [a]	Country of origin	Genome coverage (%) [b]	Mean depth
HLB 1001	IT 224676	USA	94.80	24.28
HL1B 002	IT 225083	China	93.64	26.60
HLB 1003	IT 225084	China	94.00	23.25
HLB 1004	IT225085	India	93.63	23.78
HLB 1005	IT225086	India	93.36	26.40
HLB 1006	IT225087	India	94.53	26.20
HLB 1007	IT225088	Nepal	92.99	24.58
HLB 1008	IT225089	Nepal	93.47	24.28
HLB 1009	IT225090	Nepal	93.21	27.30
HLB 1010	IT226673	China	93.64	24.68
HLB 1011	IT226674	China	94.22	25.89
HLB 1012	IT226675	China	94.57	25.79
HLB 1013	IT226676	China	93.92	24.44
HLB 1014	IT226677	China	94.06	25.74
HLB 1015	IT226678	China	94.19	25.53
HLB 1016	IT226679	China	94.01	24.93
HLB 1017	IT226680	China	94.18	25.66
HLB 1018	IT226682	Nepal	93.14	24.69
HLB 1019	IT261917	China	94.23	25.13
HLB 1020	IT261922	China	94.89	23.70
HLB 1021	IT261924	Bhutan	93.70	25.21
HLB 1022	IT278316	China	94.35	24.44
HLB 1023	IT278317	China	94.05	26.99
HLB 1024	IT278318	China	94.56	23.90
HLB 1025	IT278319	Pakistan	94.60	25.85
HLB 1026	IT226681	India	93.98	26.65

[a] National registration number managed by Nation Agrodiversity Center in Republic of Korea.
[b] Genome coverage was calculated based on a 569 Mbp genome size and 101 bp paired-end Illumina reads.

Fig 1. The seed morphology of 26 re-sequenced tartary buckwheat accessions and Clfa41.

Fig 2. Rutin content of 73 tartary buckwheat accessions according to country of origin. The phenotypic segregation is shown in box-plot format. The interquartile region, median, and range are indicated by the box, the bold horizontal line, and the vertical line respectively. USA/Japan/Russia includes 4 accessions; 1 from the USA, 1 from Japan, and 2 from Russia. We selected 26 accessions for whole-genome resequencing that originated or were popularized in different Asian and international regions. For expanded details of the 73 tartary buckwheat accessions, see S1 Table.

We produced paired-end DNA reads at 23–27-fold depths (Table 2) for the 26 accession genomes and mapped them to the reference genome (“Daegwan 3-7”). The sequencing qualities of all the samples were high: 93%–95% of the accession sample

reads were mapped to the reference genome, and 96%–97% of the reference genome was covered (S3 Table). The genetic variations (SNPs and small InDels) among the 26 accessions collected from the six countries were individually identified by Samtools and the Genome Analysis Tollkit GATK (see Methods). To increase the accuracy of the prediction, only the variations predicted by both methods were used for subsequent analysis. Bioinformatic analysis revealed 171,926 and 53,755 homogeneous and heterogeneous InDels, respectively (Fig 3). The heterozygous rates of all accessions were around 10%, reflecting the possibility of cross-pollination although self-fertilization was used (S4 Table).

Fig 3. Number of InDels in 26 tartary buckwheat accessions aligned with reference genome, "Daegwan 3-7." The number of variants homogeneous to alternative genotype; both alleles are same to alternative genotype. The number of variants heterogeneous genotype; one allele is same to reference, and another to alternative genotype.

Selection of 50 InDels and constructing barcode-type database of 73 accessions

Sequence comparison at the nucleotide level of the tested genomes with the reference genome revealed InDels that could be easily used to identify tartary buckwheat resources in common laboratories. Consistent with what has been found in other organisms [38,64], the great majority of homogeneous InDels from all accessions were short with a dominance of 4-bp or fewer events (Fig 3). From this polymorphism data, we chose InDels with more than 10-bp for further analysis to help us identify them by PCR and gel-electrophoresis. We tested 100 primer sets, focusing on their ability to amplify PCR products in the 26 genome types, since this study aimed to establish PCR-based markers applicable for all tartary buckwheat accessions. PCR primers were designed to generate amplicons of varying sizes within the 150–231 bp interval (S5 Table). Among them, 50 InDel loci were widely distributed on whole chromosomes in the reference genome ("Pinku 1") reported previously (Fig 4). We selected the 50 InDels to validate the prediction accuracy and found that 98.2% of the PCR results in the 26 accessions were consistent with the predicted results. The results of the InDel analyses were highly accurate (S5 and S6 Tables). This high accuracy provided a solid foundation for our data analyses and provides high-quality data for future data mining.

Fig 4. Schematic of the chromosomal distribution of the tartary buckwheat InDels. The number of the chromosome is shown on each chromosome of a reference genome of "Pinku1."

Primers were designed by targeting the InDel region, in such a way that the

genotypes of the 26 accessions would produce the same or different (insertion or deletion) amplicons relative to the reference genome (Fig 5). By using the previously reported barcode method [38,39], identical results to the reference genome "Daegwan 3-7" were represented by an "a," while the other results were represented by "b," which were presented as "white" and "black" barcodes, respectively. Fig 5 shows the barcode system applied to the tartary buckwheat accessions HLB1007 and HLB1008, which were converted to the standard 1D and widely-used 2D barcode types by comparing their amplicon polymorphisms to the reference genome, "Daegwan 3-7." This result highlights that the barcode types can be effectively used to investigate the degree of marker difference in tartary buckwheat accessions (Fig 6). The creation, stability and quality of the barcode system for genetic identification using the selected 50 InDels is thoroughly evaluated below.

Fig 5. Application of tartary barcode system to tartary accession, HLB1007 and HLB1008. (A) Amplification products and barcode representation of the polymorphisms using InDel marker TB11 on tartary buckwheat accessions. PCR results using the 50 InDels in (B) HLB1007 and (C) HLB1008 were converted to standard 1D and widely-used 2D barcode types in comparison with those in "Daegwan 3-7." Identical results to the reference genome were represented by white, while other results were represented by black.

Fig 6. Comparison of 2-dimensional barcode patterns between HLB1007 and HLB1008. Identical results are represented by white, while the other results are represented by black. Seed morphology of HLB1007 and HLB1008.

By building a database of the 50 InDel polymorphisms for the 73 tartary buckwheat accessions (S2 Fig), we have established a more stable foundation for utilizing the barcode system to provide a promising tool for identifying the genetic resources of tartary buckwheat. Allele frequencies and average PIC value of the 50 InDel loci are shown in Table 1. The major allele frequencies ranged from 0.5616 at the TB42 locus to 0.9863 at the TB48 locus. The average PIC value was 0.1532, with a range of 0.0267–0.3712, which implied that the selected InDels could be applied for investigating polymorphisms of tartary buckwheat accessions (S7 Table). The genotypes of the 73 tartary buckwheat accessions were constructed with a two-dimensional barcode, and genotypic discrimination was analyzed by comparing it with the accessions of the barcode system. The average difference between the analyzed accessions was 7 InDels from a total of 50 InDels, while 6 accessions had differences in 12 InDels compared to the reference genome, "Daegwan 3-7." By comparing the two-dimensional barcodes of the accessions, different blocks could be visually distinguished with ease, so that the genotype differences of the accessions could be determined (Fig 6). The discrimination power of genetic resources was high enough to broaden our understanding of both phylogenetic signals and population-level variation.

Phylogenetic analysis and population structure in tartary buckwheat accessions

Phylogenetic analysis of the 6,622 non-synonymous SNP variants and population structure subdivided the panel into 2 primary populations (Fig 7): the reference group and the non-reference group. A similar result was attained with the SSR data, which indicated two separate group clusters of tartary buckwheat [9]. The 23 accessions had 4 subgroups within the non-reference group, and were classified according to their collection area. The phylogeny resolved subgroup 2 (3 Nepal, 1 Bhutan, and 2 China) as closer to subgroup 1 (8 China and 1 India) than subgroup 3 (3 India, 1 Pakistan, and 1 Nepal).

Fig 7. Diversity analysis of 26 re-sequenced tartary buckwheat accessions. (A) Phylogenetic analysis based on non-synonymous SNPs. (B) STRUCTURE [63] classification and (C) principal component analysis were similar. Each color represents country of origin.

To validate the reliability of the 50 InDel markers for discriminating between tartary buckwheat accessions, bin maps were constructed for 73 tartary buckwheat accessions using the InDels (S7 Fig). We analyzed the population structures of the studied accessions and reference groups. The result are shown in Fig 8. At K = 3, the clusters were anchored to Southeast Asia and China. Among subgroup 1, the accessions in Southeast Asia were almost entirely composed of the red component, while the Chinese accessions were composed of the red, green, and blue components. Subgroup 1 mainly consisted of accessions from Southeast Asia (65.4%), while subgroup 2 was comprised of Chinese accessions (71.4%), and subgroup 3 consisted of Chinese accessions (97.0%). At the same time, the Chinese accessions in subgroup 1 and 3 showed a mixed composition of both red, green, and blue components. A PCA also provided similar results, with the accessions forming a tight cluster separated by collection region.

Fig 8. Diversity analysis of tartary buckwheat accessions. (A) STRUCTURE [63] results based on 50 InDels: 73 accessions were divided in 3 subpopulations. (B) Principal component analysis and STRUCUTURE classification were similar. Each color represents country of origin. (C) Phylogenetic analysis among 73 tartary buckwheat accessions using weighted neighbor-joining analysis in DARwin software [62]. The horizontal bar indicates distance based on the simple matching coefficient. Each subgroup represents a subpopulation based on STRUCTURE results.

Weighted neighbor-joining (NJ tree) relationships with bin maps revealed 3 groups (G1, G2, and G3) that contained 40, 16, and 17 accessions, respectively (Fig 8). The accessions collected in Nepal and Bhutan demonstrated substructure within group 1, being the genotypes closest to the reference group. A different result revealed that two separate groups were clustered by using non-synonymous SNP variants (Fig 7). The color-coded branches supported the classification of the 73 accessions by the collection region (Fig 8). In the phylogenetic analysis, PCA and population structure indicated that the tartary buckwheat accessions segregated into different groups that reflected their

geographical distribution. With the evidence relating the 50 InDels to environmental adaptability, these results are consistent with the segregation of the tartary buckwheat into different groups according to their geographical distribution. These results show the practicability of the barcode system, which can be used to identify genetic variation associated with phenotypic variation of functional relevance, as well as markers that can be used in crop breeding.

Discussion

Tartary buckwheat is an important crop that is poised to be the target of many future breeding efforts. It is a crop species of interest for various reasons, including its higher levels of rutin compared to the common buckwheat [10,23,65]. For breeding commercial varieties of tartary buckwheat, the elite germplasm needs to be expanded by adding various genetic resources. This is a field that is still under-represented at the genetic level, despite its vast germplasm diversity and its importance in stress adapted resources [66-70]. Particularly, since these resources possess many distinct traits, we used 73 genetic resources of tartary buckwheat to create a framework for comparative analysis. These resources differ in seed morphology (Fig 1) and rutin content (Fig 2). These differences might result in distinct responses to UV-B, drought [69], and salt stress [67]. A recent publication of tartary buckwheat genomes provided the reference genome needed to study the molecular markers to distinguish between these genetic resources [48]. The narrow genetic diversity seen within a commercial variety has various causes, including the rigid quality required by farmers and processors [28,30]. Thus, molecular markers are imperative to understand the genetic differences of tartary buckwheat accessions, which could be used to broaden the genetic resources of commercial breeding programs. To address this, we made a user-friendly barcode system using 50 InDel-based genotypes of 73 accessions. Our barcode system could be used in genetic research, breeding programs, and efficient resource management systems for buckwheat.

High-throughput sequencing technologies [49] offer immense possibilities for generating a massive amount of sequencing data from hitherto uncharacterized genomes, which facilitates the introduction of InDels as genetic markers in non-model crops, like tartary buckwheat. Given that the existing reference genome for “Pinku 1” was from China [48], we constructed a new superscaffold-scale reference genome for a variety of “Daegwan 3-7” from the Republic of Korea by combining the sequencing strategies of PacBio and Illumina Hiseq2500. Using this integrated approach, we obtained an N50 scaffold size of 463,432 bps for the draft genome of “Daegwan 3-7” (Table 1). To explore the genomic variation of tartary buckwheat accessions, we performed whole-genome sequencing for 26 accessions across the reference genome (“Daegwan 3-7”) (Table 2). Significantly, the depth (23 × – 27 ×) of sequence coverage indicates that it was well-suited for the sequence analysis of the 26 genetic resources. From these genome comparisons, 171,926 homogeneous InDels and 53,755 heterogeneous InDels were

obtained with an InDel density of 2 for every 1 kb through pairwise comparison with the reference genome (Fig 3 and S5 Table). Among them, 50 polymorphic InDels were selected by gel electrophoresis, which PCR results were 98.2% matched with the predicted results (S6 and S7 Tables). Moreover, the 49 InDels were distributed widely in the whole genome of "Pinku 1." Considering that the TB8 locus was not found in "Pinku 1," the two reference genomes for "Pinku 1" and "Daegwan 3-7" can provide backbones and references for developing the pan-genome to reduce reference bias. We plan to continue improve the draft genome of tartary buckwheat cv. "Daegwan 3-7" for use in the pan-genome. Our results suggest that superscaffold-scale *de novo* draft genomes usually facilitate the introduction of InDels as genetic markers in non-model crops, like tartary buckwheat.

To efficiently identify tartary buckwheat accessions, we developed the barcode system using 50 InDels selected from 26 landrace genomes using a resequencing strategy. The 2D-barcode system (Figs 5 and 6) is a powerful tool to compare the genotypes of tartary buckwheat accessions [38-40]. The landrace genomes collected from six countries, contained a wealth of genetic diversity adapted to wide-ranging environmental conditions (Table 2). Through the application of 50 InDels to this collection, we found that both geographical and environmental factors have shaped the genetic diversity of tartary buckwheat landraces (Fig 7). In the 73 accessions, the population structure analyses using the DNA barcode data revealed their geographical distribution (Fig 8), which is geared for adaptation in the diverse topographic conditions of the agroecosystem [71-74]. In the phylogenetic analysis using non-synonymous SNPs, the 26 accessions showed a close relationship with those from Bhutan and Pakistan. (Fig 7). This observation is consistent with a previous study, which found that population genetic structure analysis using SSR data clustered one group that was mainly distributed in Nepal, Bhutan, and the Yunnan-Guizhou Plateau regions of China and the other group principally derived from the Loess Plateau regions, Hunan and Hubei of China and the USA [9]. These findings were consistent with the previously suggested method of seed exchange within a close geographic distance [5, 8]. Altogether with SSR [9], AFLP [41], RAPD [42,43], and ISSR [44], the barcode system using InDels can complement current research and broaden our understanding of both phylogenetic signals and population-level variations [38-40]. These phylogenetic and structural analyses in the tartary buckwheat accessions will serve as a significant resource to gain more insight into the evolution of lineage-specific gene networks associated with the environmentally-specific developmental and adaptive traits of tartary buckwheat landraces.

According to their position in unigenes, the 50 InDels were divided into five types: coding region, 5′ untranslated region, 3′ untranslated region, splice region and intergenic region (S7 Table). The InDels may be biased toward changes in gene expression via cis-regulatory mutations [75]. The InDels of certain genes, e.g., *RPP4* [76], *UGT73B5* [77], *TPS4* [78], and *DREB3* [79] have been postulated to play a role in different responses to environmental stresses during growth and survival. With the evidence relating the 50 InDels to environmental adaptability, these results are consistent with the segregation of the tartary buckwheat into different groups according to their geographical distribution.

Despite self-fertilizing the tartary buckwheat, through 3 additional generations using the SSD method, around 10% heterogeneous InDels were detected in the genomic DNA from the 26 progenies (S3 Table). Previous studies have shown that farmers cultivated several tartary buckwheat landraces suitable for the multiple local microclimates and environmental conditions [5], which could drive the gene flow of landraces within a region [80]**.** In this study, we uncovered strong support for allogamy among landraces that contributes to heterogeneous InDels in tartary buckwheat, which might drive the genetic diversity of landraces within a region. Considering the effect of heterozygous InDel on the increase of single nucleotide mutation rate [75,81], it might be an important characteristic that allows tartary buckwheat to adjust to the diverse topographic conditions of the agroecosystem. These results suggest that the divergent gene expression of the InDel loci may affect its adaptation to environmental conditions.

We found that tartary buckwheat accessions could be distinguished by 50 InDels based on genotype according to location with the differing environment (Figs 7 and 8). Tartary buckwheat is a diverse crop in which separate domestication events happened in each gene pool followed by race and market class diversification that has resulted in different morphological characteristics in each commercial market class [82]. Combining multiple data types can compensate for missing or unreliable information in any single data type. Importantly, complete resource management is only likely to be discovered if the genotype, phenotype and chemotype of tartary buckwheat accessions are considered in an analysis. To make a user-friendly common platform for genotype, phenotype, and chemotype resources, we plan to incorporate genotypic data with that phenotype and chemotype (rutin content) data for tartary buckwheat accessions (S3 Fig). Our platform could be used in genetic research, breeding programs, and for efficient resource management systems for buckwheat. Increasing tartary buckwheat productivity will require adapting the crop to the agricultural environment of new locations. The barcode system can assist tartary buckwheat breeders in identifying germplasm accessions and can be used as donor parents for breeding tartary buckwheat cultivars.

Author contributions

HBS, SJK, SL and YHK conceptualized the research. HBS, SGP, DHO, and YHK were involved in data curation, and formal analysis. SJK, and YHK strived for funding acquisition, and project administration. YHK supervised the project (Project No. PJ01189401) which was managed by National Institute of Crop Science. HBS, SJK, and YHK took a part in investigation, methodology, validation, visualization. SJK, YHK, SYH and JHN provided resources for the research. HBS and HYN performed the role of writing-original draft preparation. HBS and YHK played the role in writing-review & editing.

Acknowledgements

Sincere thanks to Misook Lee for the cultivation of tartary buckwheat resources, Juyoung Shim for the DNA extractions of samples, and Hyeongjin Shin for the technical assistance in the laboratory. The authors would like to thank Enago (www.enago.co.kr) for the English language review.

References

1. Ohnishi O, Matsuoka H. Search for the wild ancestor of buckwheat II. Taxonomy of *Fagopyrum* (Polygonaceae) species based on morphology, isozymes and cpDNA variability. Genes Genet Syst. 1996;71:383-390. doi: 10.1266/ggs.71.383

2. Yasui Y, Ohnishi O. Phylogenetic relationships among *Fagopyrum* species revealed by the nucleotide sequences of the ITS region of the nuclear rRNA gene. Genes Genet Syst. 1998;73(4):201-210. doi: 10.1266/ggs.73.201

3. Eggum BO, Kreft I, Javornik B. Chemical composition and protein quality of buckwheat (*Fagopyrum esculentum* Moench). Plant Foods Hum Nutr. 1980;30(3):175-179. doi: 10.1007/BF1094020

4. Bonafaccia G, Marocchini M, Kreft I. Composition and technological properties of the flour and bran from common and tartary buckwheat. Food Chem. 2003;80(1):9-15. doi: 10.1016/S0308-8146(02)00228-5

5. Huang W, Jarvis DI, Ahmed S, Long C. Tartary buckwheat genetic diversity in the Himalayas associated with farmer landrace diversity and low dietary dependence. Sustainability. 2017;9(10):1806. doi: 10.3390/su9101806

6. Murai M, Ohnishi O. Population genetics of cultivated common buckwheat, Fagopyrum esculentum Moench. X. Diffusion routes revealed by RAPD markers. Genes Genet. Syst. 1996;71:211-218. Available at http://jstage.jst.go.jp/article/ggs/71/4/71_4_211/_pdf/-char/en. Accessed 18 September 2020

7. Ohnishi O. Search for the wild ancestor of buckwheat III. The wild ancestor of cultivated common buckwheat, and of tartary buckwheat. Econ Bot. 1998;52(2):123-133. Available at http://www.jstor.com/stable/4256049. Accessed 18 September 2020

8. Delêtrea M, McKey DB, Hodkinson TR. Marriage exchanges, seed exchanges, and the dynamics of manioc diversity. Proc Natl Acad Sci U S A. 2011;108(45):18249–18254. doi: 10.1073/pnas.1106259108

9. Hou S, Sun Z, Linghu B, Xu D, Wu B, Zhang B, et al. Genetic diversity of buckwheat cultivars (*Fagopyrum tataricum* Gaertn.) assessed with SSR markers developed from genome survey sequences. Plant Mol Biol Report. 2016;34(1):233–241. doi: 10.1007/s11105-015-0907-5

10. Kreft I, Fabjani N, Germ M. Rutin in buckwheat-protection of plants and its importance for the production of functional food. Fagopyrum. 2003;20:7-11. doi: 10.1.1.563.1011&rep=rep1&type=pdf

11. Bisht IS, Mehta PS, Bhandari DC. Traditional crop diversity and its conservation on-farm for sustainable agricultural production in Kumaon Himalaya of Uttaranchal state: A case study. Genet Resour Crop Evol. 2007;54:345–357. doi: 10.1007/s10722-005-5562-5

12. Liu M, Ma Z, Zheng T, Sun W, Zhang Y, Jin W, et al. Insights into the correlation between physiological changes in and seed development of tartary buckwheat (*Fagopyrum tataricum* Gaertn.). BMC Genomics. 2018;19:648. doi: 10.1186/s12864-018-5036-8

13. Spengler R. (2020). Anthropogenic seed dispersal: Rethinking the origins of plant domestication. Trends Plant Sci. 2020;25(4):340-348. doi: 10.1016/j.tplants.2020.01.05

14. Bevan MW, Uauy C, Wulff BBH, Zhou J, Krasileva K, Clark MD. Genomic innovation for crop improvement. Nature. 2017;543:346-354. doi: 10.1038/nature22011

15. Li SQ, Zhang QH. Advances in the development of functional foods from buckwheat. Crit Rev Food Sci Nutr. 2001;41(6):451-464. doi: 10.1080/20014091091887

16. Jones MK, Liu XY. Origins of agriculture in East Asia. Science. 2009;324(5928):730-731. doi: 10.1126/science.1172082

17. Steadman KJ, Burgoon MS, Lewis BA, Edwardson SE, Obendorf RL. Minerals, phytic acid, tannin and rutin in buckwheat seed milling fractions. J Sci Food Agric. 2001;81(11):1094-1100. doi:10.1002/jsfa.914

18. Fabjan N, Rode J, Košir IJ, Wang Z, Zhang Z, Kreft I. Tartary buckwheat (*Fagopyrum tataricum* Gaertn.) as a source of dietary rutin and quercitrin. J Agric Food Chem. 2003;51(22):6452-6455. doi: 10.1021/jf034543e

19. Kreft I, Fabjan N, Yasumoto K. Rutin content in buckwheat (*Fagopyrum esculentum* Moench) food materials and products. Food Chem. 2006;98(3):508-512. doi: 10.1016/j.foodchem.2005.05.081

20. Gupta N, Sharma SK, Rana JC, Chauhan RS. Expression of flavonoid biosynthesis genes via-**à**-vis rutin content variation in different growth stages of *Fagopyrum* species. J Plant Physiol. 2011;168(17):2117-2123. doi: 10.1016/j.jplph.2011.06.018

21. Wang L, Yang X, Qin P, Shan F, Ren G. Flavonoid composition, antibacterial and antioxidant properties of tartary buckwheat bran extract. Ind Crops Prod. 2013;49:312-317. doi: 10.1016/j.indcrop.2013.04.039

22. Hu Y, Hou Z, Liu D, Yang X. Tartary buckwheat flavonoids protect hepatic cells against high glucose-induced oxidative stress and insulin resistance via MAPK signaling pathways. Food Funct. 2016;7(3):1523-1536. doi: 10.1039/c6fo1467k

23. Kim SJ, Sohn HB, Kim GH, Lee YY, Hong SY, Kim GD, et al. Comparison and validation of rutin and quercetin contents according to the extraction method of tartary buckwheat (*Fagopyrum tataricum* Gaertn.). Korean J Food Sci Technol. 2017;49(3):258-264. doi: 10.9721/KJFST.2017.49.3.258

24. Joshi BK. Correlation, regression and path coefficient analyses for some yield components in common and Tartary buckwheat in Nepal. Fagopyrum. 2005;22:77-82. doi: 10.1.1.524.5293&rep=rep1&type=pdf

25. Wang Y, Campbell C. Tartary buckwheat breeding (*Fagopyrum tataricum* Gaertn.) through hybridization with its rice-tartary type. Euphytica. 2007;156(3):399-405. doi: 10.1007/s10681-007-9839-3

26. Suzuki T, Morshita T, Mukasa Y, Takigawa S, Yokota S, Ishiguro K, Noda T. Breeding of 'Manten-Kirari', a non-bitter and trace-rutinosidase variety of Tartary buckwheat (*Fagopyrum tataricum* Gaertn.). Breed Sci. 2014;64(4):344-350. doi: 10.1270/jsbbs.64.344

27. Li C, Xie Z, Wang Y, Lu W, Yin G, Sun D, et al. Correlation and genetic analysis of seed shell thickness and yield factors in Tartary buckwheat (*Fagopyrum tataricum* (L.) Gaertn.). Breed Sci. 2019;69:464-470. doi:10.1270/jsbbs.18081

28. Singh SP. Broadening the genetic base of common bean cultivars. Crop Sci. 2001;41:1659-1675. doi: 10.2135/cropsci2001.1659

29. Hyten DL, Song Q, Choi IY, Nelson RL, Costa JM, Specht JE, et al. Impacts of genetic bottlenecks on soybean genome diversity. Proc Natl Acad Sci U S A. 2006;103(45):16666–16671. doi: 10.1073/pnas.0604379103

30. Bhandari HR, Bhanu AN, Srivastava K, Singh MN, Shreya, Hemantaranjan A. Assessment of genetic diversity in crop plants. Adv Plants Agric Res. 2017;7(3):279-286. doi: 10.15406/apar.2017.07.00255

31. Ericksen PJ. Conceptualizing food systems for global environmental change research. Glob Environ Change. 2008;18(1):234-245. doi: 10.1016/j.gloenvcha.2007.09.002

32. Hughes AR, Inouye BD, Underwood JN, Vellend M. Ecological consequences of genetic diversity. Ecol Lett. 2008;11(6):609-623. doi: 10.1111/j.1461-0248.01179.x

33. Britten RJ, Rowen L, Williams J, Cameron RA. Majority of divergence between closely related DNA samples is due to indels. Proc Natl Acad Sci U S A.

2003;100(8):4661-4665. doi: 10.1073/pnas.0330964100

34. Vali U, Brandsrom M, Johansson M, Ellegren H. Insertion-deletion polymorphisms (indels) as genetic markers in natural populations. BMC Genet. 2008;9:8. doi: 10.1186/1471-2156-9-8

35. Hou X, Li L, Peng Z, Wei B, Tang S, Ding M, et al. A platform of high-density INDEL/CAPS markers for map-based cloning in Arabidopsis. Plant J. 2010;63(5):880–888. doi: 10.1111/j.1365-313X.2010.04277.x

36. Moghaddam SM, Song Q, Mamidi S, Schmutz J, Lee R, Cregan P, et al. Developing market class specific InDel markers from next generation sequence data in *Phaseolus vulgaris* L. Front Plant Sci. 2014;5:185. doi: 10.3389/fpls.2014.00185

37. Meng HT, Zhang YD, Shen CM, Yuan GL, Yang CH, Jin R, et al. Genetic polymorphism analyses of 30 InDels in Chinese Xibe ethnic group and its population genetic differentiations with other groups. Scientific Reports. 2015;5:8260. doi: 10.1038/srep08260

38. Sohn HB, Kim SJ, Hwang TY, Park HM, Lee YY, Markkandan K, et al. Barcode system for genetic identification of soybean [*Glycine max* (L.) Merrill] cultivars using InDel markers specific to dense variation blocks. Front Plant Sci. 2017;8:520. doi: 10.3389/fpls.2017.00520

39. Sohn HB, Song YH, Kim SJ, Hong SY, Kim KD, Koo BC, Kim YH. Identification and chromosomal reshuffling patterns of soybean cultivars bred in Gwangwon-do using 202 InDel markers specific to variation blocks. Korean J Breed Sci. 2018;50(4):396-405. doi: 10.9787/KJBS.2018.50.4.396

40. Chun JB, Jin M, Jeong N, Cho C, Seo MS, Choi MS, et al. Genetic identification and phylogenetic analysis of new varieties and 149 Korean cultivars using 27 InDel markers selected from dense variation blocks in soybean (*Glycine max* (L.) Merrill). Korean J Plant Res. 2019;32(5):519-542. doi: 10.7732/kjpr.2019.32.5.519

41. Tsuji K, Ohnishi O. Phylogenetic relationships among wild and cultivated tartary buckwheat (*Fagopyrum tataricum* Gaertn.) populations revealed by AFLP analyses. Genes Genet Syst. 2001;76(1):47–52. doi: 10.1266/ggs.76.47

42. Tsuji K, Ohnishi O. Origin of cultivated Tatary buckwheat (*Fagopyrum tataricum* Gaertn.) revealed by RAPD analyses. Genet Resour Crop Evol. 2000;47:431–438. doi: 10.1023/A:1008741513277

43. Tsuji K, Ohnishi O. Phylogenetic position of east Tibetan natural populations in tartary buckwheat (*Fagopyrum tataricum* Gaertn.) revealed by RAPD analyses. Genet Resour Crop Evol. 2001;48:63–67. doi: 10.1023/A:1011286326401

44. Zhang A, Ohnishi O. Molecular characterization of genetic diversity of underutilized crops; Buckwheat as an example. Acta Hortic. 2013;979:407-419. doi: 10.17660/ActaHortic.2013.979.44

45. Katsu K, Suzuki T, Fujino K, Morishita T, Noda T. Development of a DNA marker for variety discrimination specific to ‘Manten-Kirari’ based on the NGS-RNA sequence in Tartary buckwheat (*Fagopyrum tataricum*). Food Chem. 2019;295:51-57. doi: 10.1016/j.foodchem.2019.05.087

46. Zhang L, Ma M, Liu L. Identification of genetic locus underlying easy dehulling in rice-tartary for easy postharvest processing of tartary buckwheat. Genes. 2020;11(4):459. doi: 10.3390/genes11040459

47. Yasui Y, Hirakawa H, Ueno M, Matsui K, Tanaka TK, Yang SJ, et al. Assembly of the draft genome of buckwheat and its applications in identifying agronomically useful genes. DNA Res. 2016;23(3):215-224. doi: 10.1093/dnares/dsw012

48. Zhang L, Li X, Ma B, Gao Q, Du H, Han Y, et al. The Tartary buckwheat genome provides insights into rutin biosynthesis and abiotic stress tolerance. Mol Plant. 2017;10(9):1224-1237. doi: 10.1016/j.molp.2017.08.013

49. Bansal V, Boucher C. Sequencing technologies and analyses: Where have we been and where are we going? iScience. 2019;18:37-41. doi: 10.1016/j.isci.2019.06.035

50. Janwan M, Sreewongchai T, Sripichitt P. Rice breeding for high yield by advanced single seed descent method of selection. J Plant Sci. 2013;8(1):24-30. doi: 10.3923/jps.2013.24.30

51. Halbrecq B, Romedenne P, Ledent JF. Evolution of flowering, ripening and seed set in buckwheat (*Fagopyrum esculentum* Moench): quantitative analysis. Europ J Agronomy. 2005;23:209-224. doi: 10.1016/j.eja.2004.11.006

52. Reyes-Valdés MH, Santacruz-Varela A, Martinez O, Simpson J, Hayano-Kanashiro, C, Cortés-Romero C. Analysis and optimization of bulk DNA sampling with binary scoring for germplasm characterization. PLoS ONE. 2013;8:e79936. doi:10.1371/journal.pone.0079936

53. Rogers SO, Bendich AJ. Extraction of total cellular DNA from plants, algae and fungi. In: Gelvin SB, Schilperoort RA, editors. Plant Molecular Biology Manual. Springer: Dordrecht; 1994. p. 183-190. doi: 10.1007/978-94-011-0511-8_12

54. Chin CS, Peluso P, Sedlazeck FJ, Nattestad M, Concepcion GT, Clum A, et al. Phased diploid genome assembly with single-molecule real-time sequencing. Nat Methods. 2016;13:1050-1054. doi: 10.1038/nmeth.4035

55. Luo R, Liu B, Xie Y, Li Z, Huang W, Yuan J, et al. SOAPdenovo2; an empirically improved memory-efficient short- read *de novo* assembler. Gigascience. 2012;1(1): 18. doi: 10.1186/s13742-015-0069-2

56. Huang S, Kang, M, Xu A. Haplomergers: rebuilding both haploid subassemblies from high-heterozygosity diploid genome assembly. Bioinformatics. 2017;33(16):2577-2579. doi: 10.1093/bioinformatics/btx220

57. Boetzer M, Henkel CV, Jansen HJ, Butler D, Pirovano W. Scaffolding pre-assembled contigs using SSPACE. Bioinformatics. 2011;27(4):578-579. doi: 10.1093/bioinformatics/btq683

58. Li H, Durbin R. Fast and accurate short read alignment with Burrows-Wheeler transform. Bioinformatics. 2009;25(14):1754-1760. doi: 10.1093/bioinformatics/btp324

59. McKenna A, Hanna M, Banks E, Sivachenko A, Cibulskis K, Kernytsky A, et al. The genome analysis toolkit: a MapReduce framework for analyzing next-generation DNA sequencing data. Genome Res. 2010;20(9):1297-1303. doi: 10.1101/gr.107524.110

60. Anderson JA, Churchill GA, Autrigue JE, Tanksley SD, Sorrells ME. Optimizing parental selection for genetic linkage maps. Genome. 1993;36(1):181–186. doi: 10.1139/g93-024

61. Liu K, Muse S. Powermarker: an intergrated analysis environment for genetic marker analysis. Bioinformatics. 2005;21(9):2128-2129. doi: 10.1093/bioinformatics/bti282

62. Perrier X, Jacquemoud-Collet JP. DARwin Software. 2006. Available at http://darwin.cirad.fr/

63. Prichard JK, Stephens M, Rosenberg NA, Donnelly P. Association mapping in structured populations. Am J Hum Genet. 2000;67(1):170-181. doi: 10.1086/302959

64. Kim YH, Park HM, Hwang TY, Lee SK, Choi MS, Jho S, et al. Variation block-based genomics method for crop plants. BMC Genomics 2014;15:477. doi: 10.1186/1471-2164-15-477

65. Jiang P, Burczynski F, Campbell C, Pierce G, Austria J A, Briggs CJ. Rutin and flavonoid contents in three buckwheat species *Fagopyrum esculentum*, *F. tataricum*, and *F. homotropicum* and their protective effects against peroxidation. Food Res Int. 2006;40(3):356-364. doi: 10.1016/j.foodres.200610.009

66. Kreft S, Strukelj B, Gaberscik A, Kreft I. Rutin in buckwheat herbs grown at different UV-B radiation levels: comparison of two UV spectrophotometric and an HPLC method. J Exp Bot. 2002;53(375):1801-1804. doi: 10.1093/jxb/erf032

67. Matsuura H, Inanaga S, Murata K. Differences in the vegetative growth between common and tartary buckwheat in saline hydroponic culture. Plant Prod Sci. 2005;8(5):533–538. doi: 10.1626/pps.8.533

68. Owens DK, Alerding AB, Crosby KC, Bandara AB, Westwood JH, Winkel BSJ. Functional analysis of a predicted flavonol synthase gene family in Arabidopsis. Plant Physiol. 2008;147:1046-1061. doi: 10.1104/pp.108.117457

69. Germ M, Brenznik B, Dolinar N, Kreft I, Gaberščik A. The combined effect of water limitation and UV-B radiation on common and tartary buckwheat. Cereal Res.

Commun. 2013;41:97-105. doi: 10.1556/CRC.2012.031

70. Pandey V, Niranjan A, Atri N, Chandrashekhar K, Misha MK, Trivedi PK, Misra P. WsSGTL1 gene from *Withania somnifera*, modulates glycosylation profile, antioxidant system and confers biotic and salt stress tolerance in transgenic tobacco. Planta. 2014;239(6):1217-1231. doi: 10.1007/s00425-014-2046-x

71. Jackson L, van Noordwijk M, Bentsson J, Foster W, Lipper L, Pulleman M, et al. Biodiversity and agricultural sustainability: from assessment to adaptive management. Curr Opin Environ Sustain. 2010;2:80-87. doi: 10.1016/j.cosust.2010.02.007

72. Vigouroux Y, Barnaud A, Scarcelli N, Thuillet AC. Biodiversity, evolution and adaptation in cultivated crops. C R Biol. 2011;334:450–457. doi: 10.1016/j.crvi.2011.03.003

73. Ahmed S, Peters CM, Long CL, Meyer R, Unachukwu U, Litt A, et al. Biodiversity and phytochemical quality in indigenous and state-supported tea management systems of Yunnan, China. Conserv Lett. 2013;6:28–36. doi: 10.1111.j.1755-263x.2012.00269.x

74. Qu Y, Zhou Y, Wang Z, Wang Z, Wang P, Gao J, et al. Analysis of genetic diversity and structure of Tartary buckwheat resources from production regions. Scientia Agricultura Sinica. 2016;49(11):2049-2062. doi: 10.3864/j.issn.0578-1752.2016.11.002

75. Tian D, Wang Q, Zhang P, Araki H, Yang S, Kreitman M, et al. Single-nucleotide mutation rate increases close to insertion/deletions in eukaryotes. Nature. 2008;455(7209):105-108. doi: 10.1038/nature07175

76. van der Biezen EA, Freddie CT, Kahn K, Parker JE, Jones JDG. Arabidopsis RPP4 is a member of the RPP5 multigene family of TIR-NB-LRR genes and confers downy mildew resistance through multiple signaling components. Plant J. 2002;29(4):439-451. doi; 10.1046/j.0960-7412.2001.01229.x

77. Langlois-Meurinne M, Gachon CM, Saindrenan P. Pathogen-responsive expression of glycosyltransferase genes UGT73B3 and UGT73B5 is necessary for resistance to *Psudomonas syringae* pv tomato in Arabidopsis. Plant Physiol. 2005;139(4):1890-1901. doi: 10.1104/pp.105.067223

78. Attaran E, Rostás M, Zeier J. *Pseudomonas syringae* elicits emission of the terpenoid (E,E)-4,8,12-trimethnyl-1,3,7,11-tridecatetraene in Arabidopsis leaves via jasmonate signaling and expression of the terpene synthase TPS4. Mol Plant Microbe Interact. 2008;21(11):1482-1497. doi: 10.1094/MPMI-21-11-1482

79. Islam MS, Wang MH. Expression of dehydration responsive element-binding protein-3 (DREB3) under different abiotic stresses in tomato. BMB Rep. 2009;42(9):611-616. doi: 10.5483/bmbrep.2009.42.9.611

80. Bellucci E, Bitocchi E, Rau D, Nanni L, Ferradini N, Giardini A, et al. Population

structure of barley landrace populations and gene-flow with modern varieties. PLoS One. 2013;8:e83891. doi: 10.1371/journal.pone.0083891

81. Hollister JD, Ross-Ibara J, Gaut BS. Indel-associated mutation rate varies with mating system in flowering plant. Mol Biol Evol. 2010;27(2):409-416. doi: 10.1093/molbev/msp249

82. Leishman MR, Westoby M, Jurado E. Correlates of seed size variation: a comparison among five temperate floras. J Ecol. 1995;83(3):517-530. doi: 10.2307/2261604

Supporting information

S1 Fig. The draft genome assembly process using *Fagopyrum tataricum* cv. "Daegwan 3-7."

S2 Fig. Agarose gels showing the separation of alleles of 50 InDels in 73 tartary buckwheat accessions.

S3 Fig. Application of barcode system to tartary buckwheat accession, HLB1003.

S1 Table. **List of 73 tartary buckwheat accessions used for evaluating the selected InDels.**

S2 Table. A gradient UPLC method for the simultaneous determination of rutin and quercetin.

S3 Table. Statistics of filtered raw data for evaluating Illumina paired read quality in 26 tartary buckwheat accessions.

S4 Table. Number of InDels showing alternative genotype in 26 re-sequenced genomes.

S5 Table. **The 50 InDel markers developed to discriminate tartary buckwheat accessions.**

S6 Table. A diagram of PCR band patterns of 73 tartary buckwheat accessions obtained using 50 InDel markers.

S7 Table. Statistics of the 50 InDels in tartary buckwheat accessions (n=73).

Figure 1

Click here to access/download;Figure;Fig1.tif

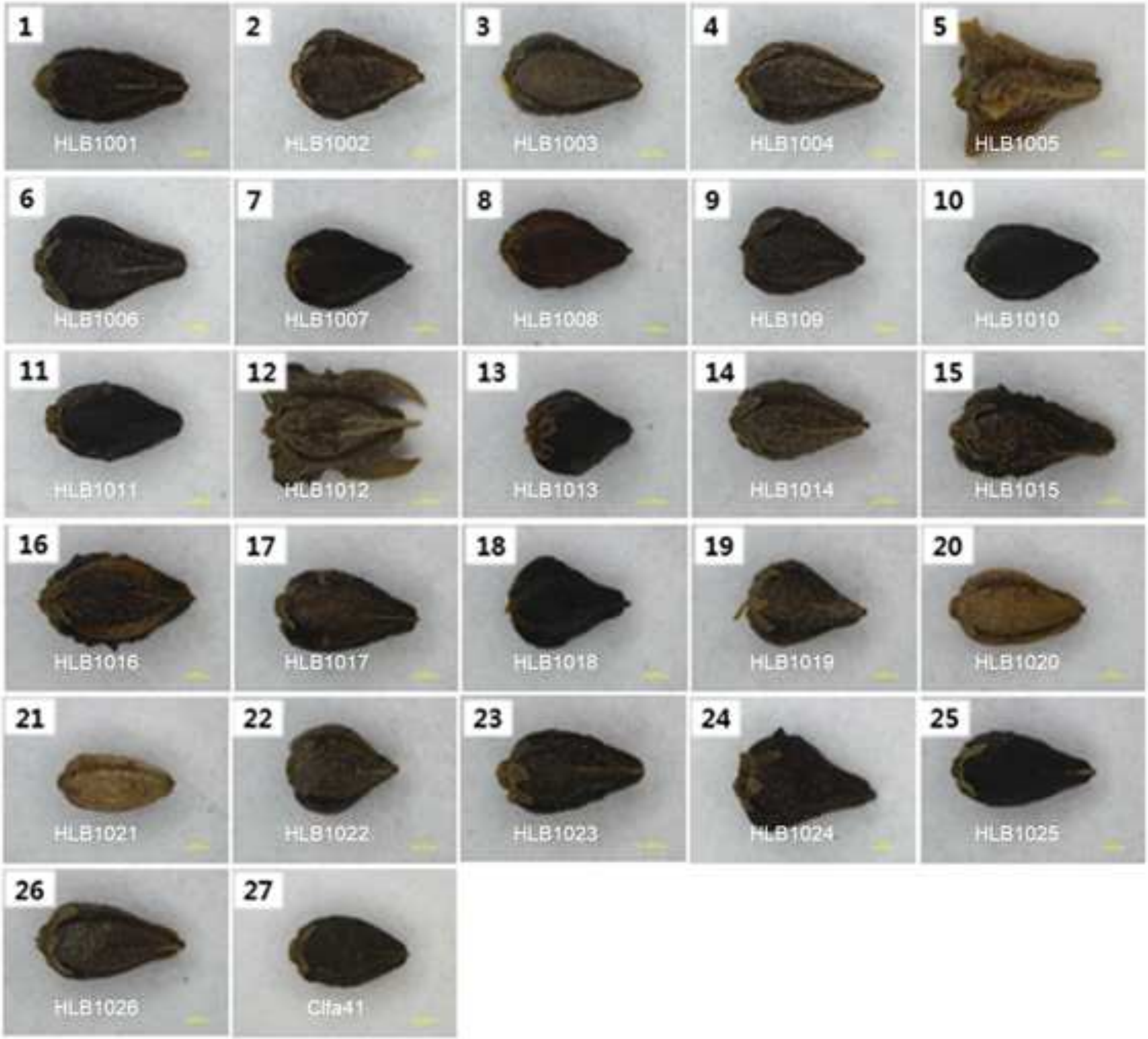

Figure 2

Click here to access/download;Figure;Fig2.tif

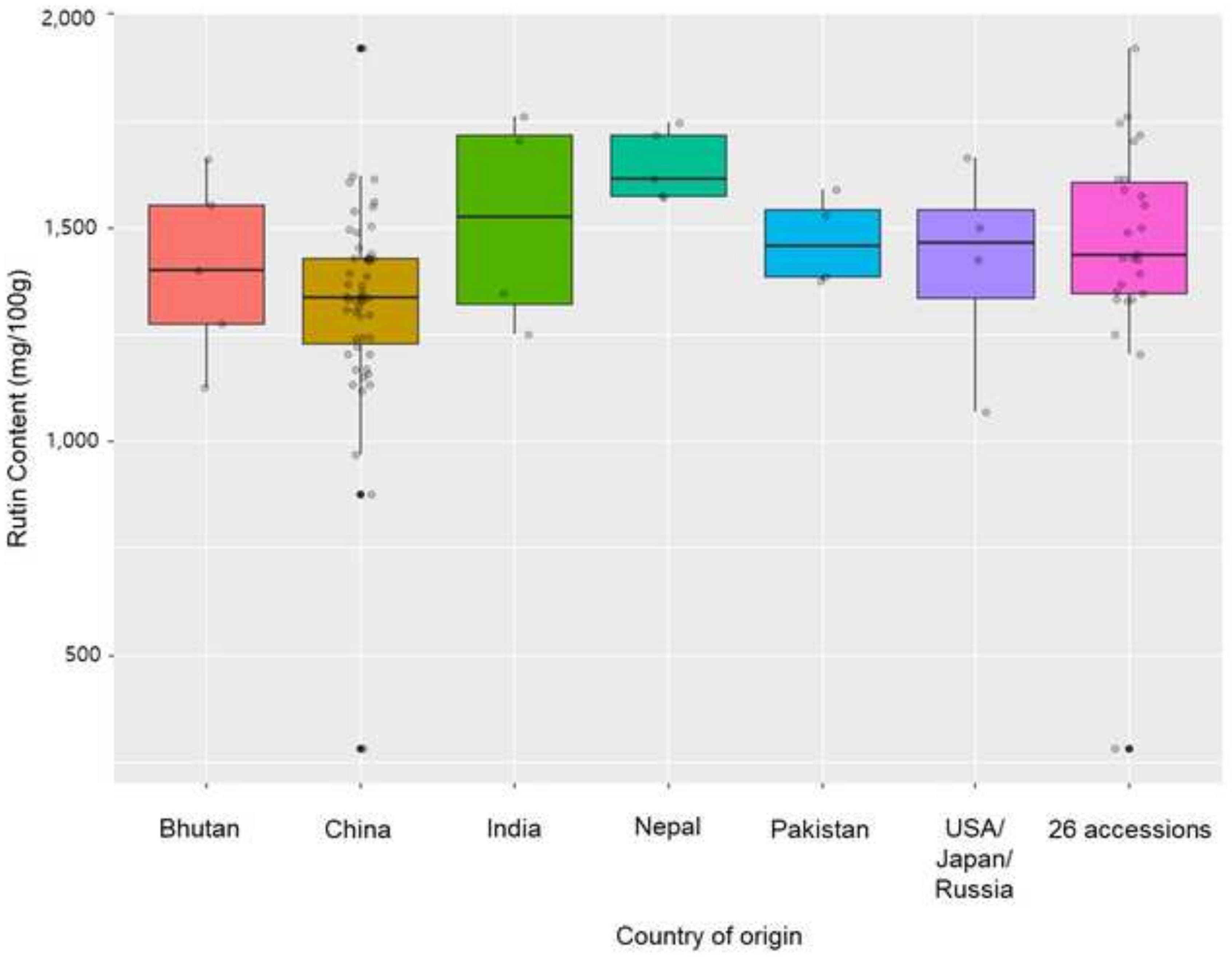

Figure 3 Click here to access/download;Figure;Fig3.tif

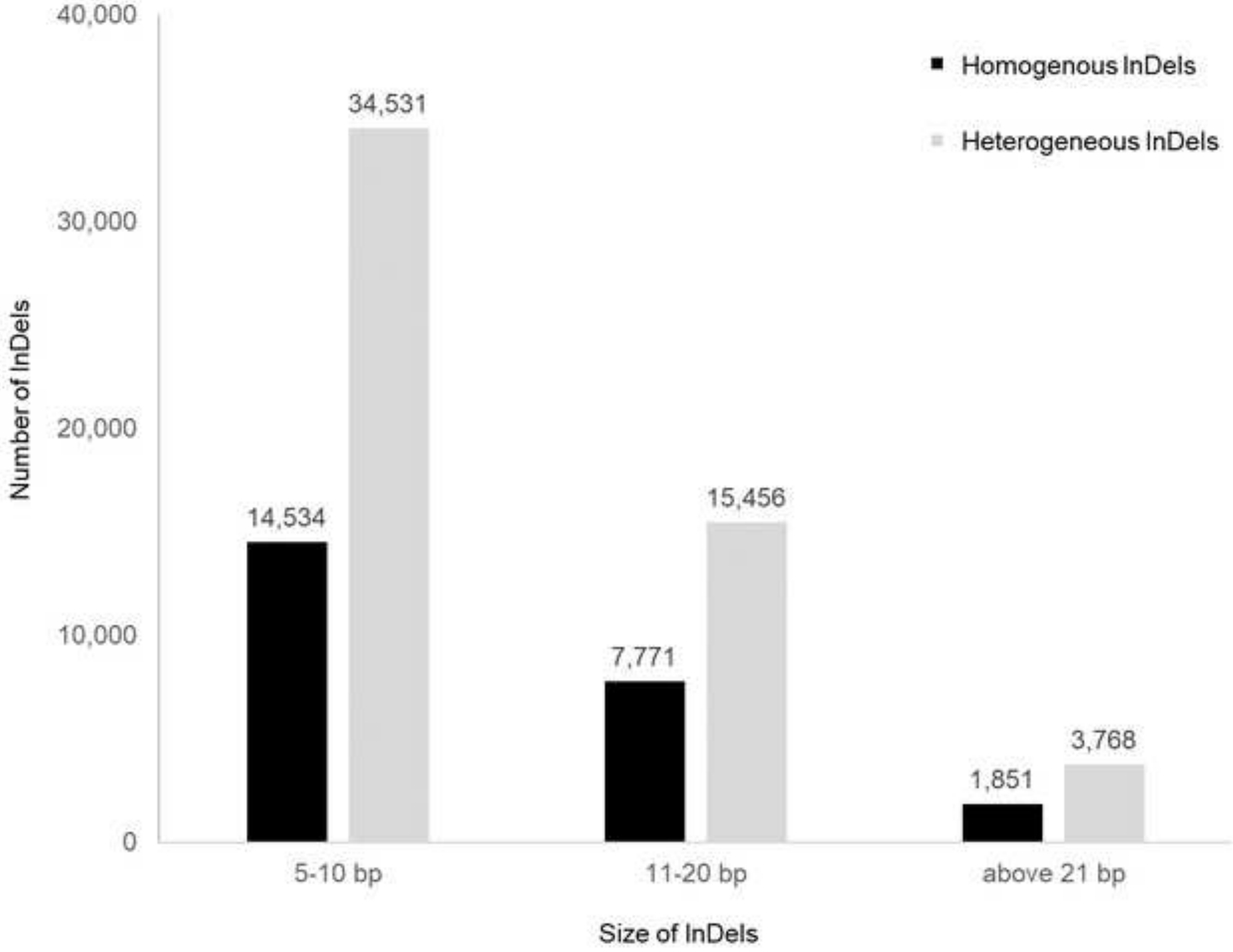

Figure 4

Click here to access/download;Figure;Fig4.tif

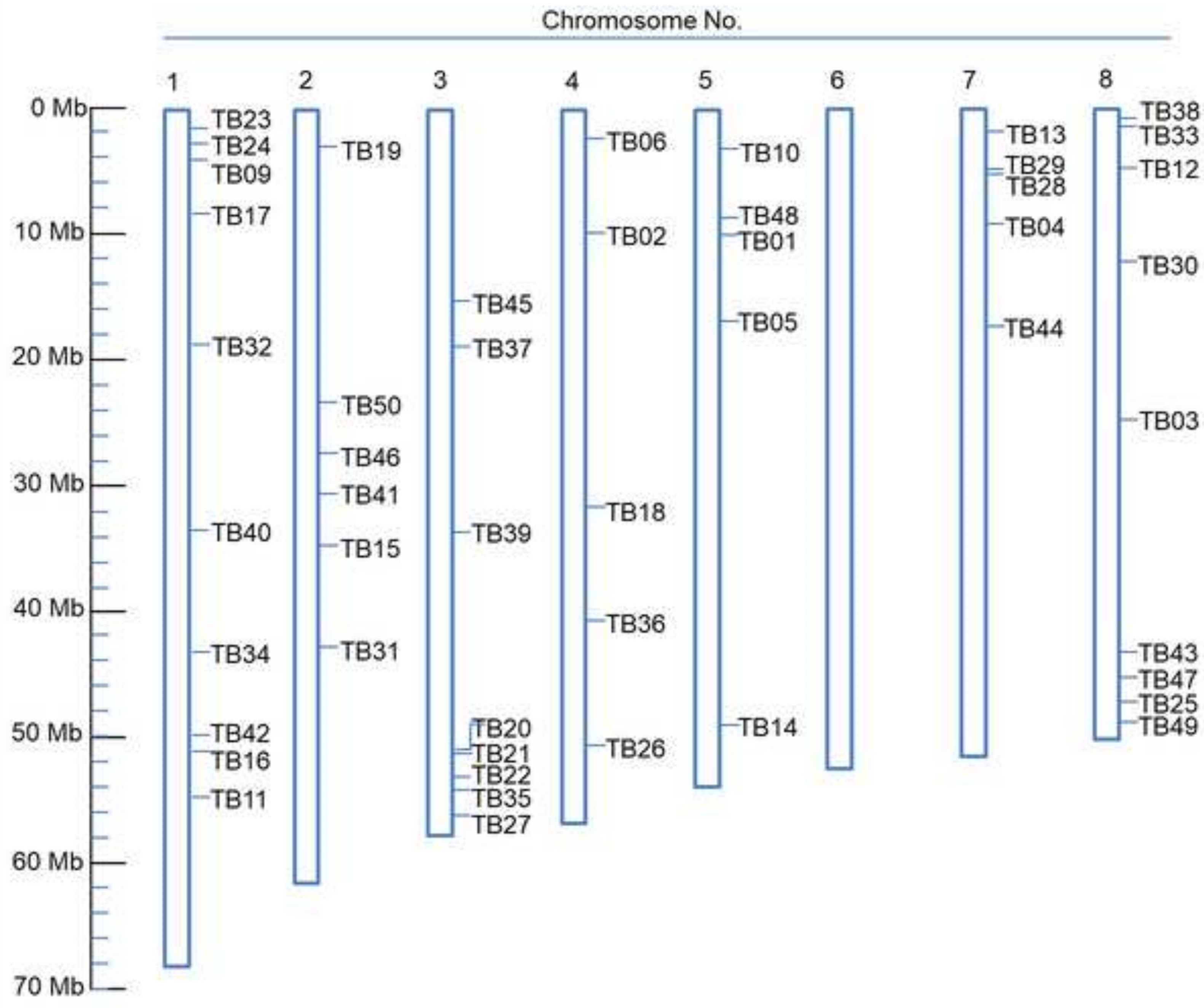

Figure 5

Click here to access/download;Figure;Fig5.tif

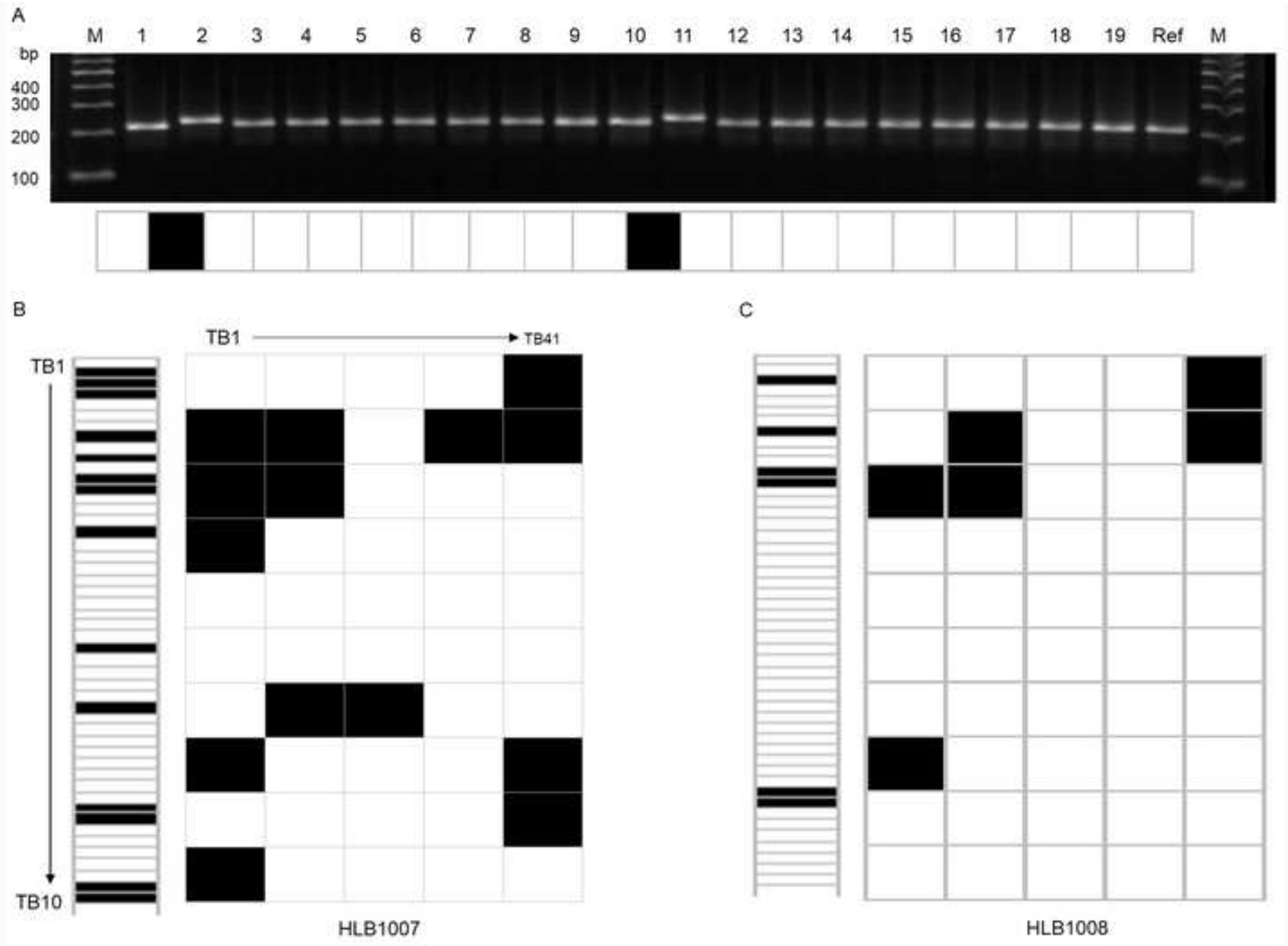

Figure 6

Click here to access/download;Figure;Fig6.tif

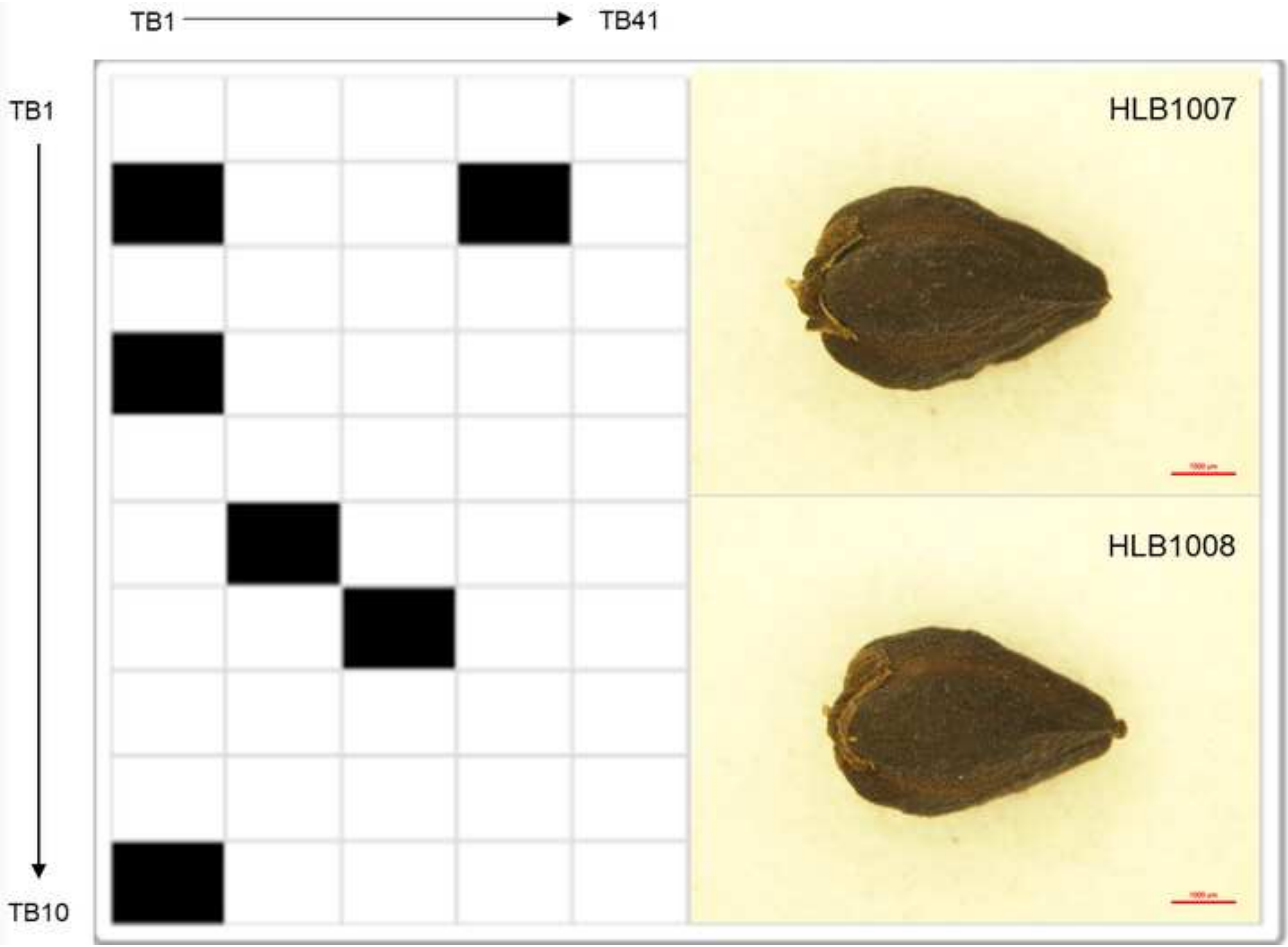

Figure 7

Click here to access/download;Figure;Fig7.tif

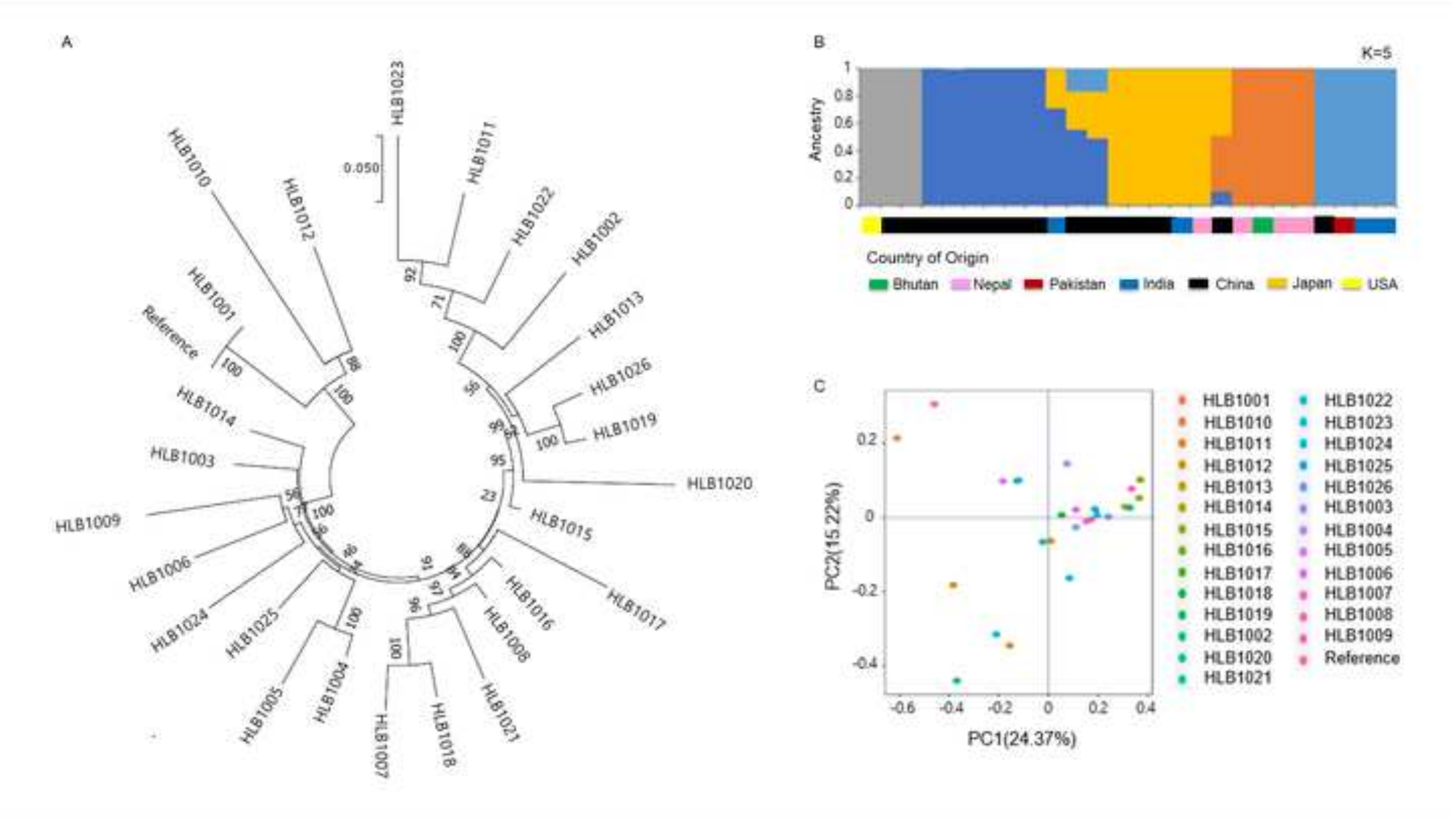

Figure 8

Click here to access/download;Figure;Fig8.tif

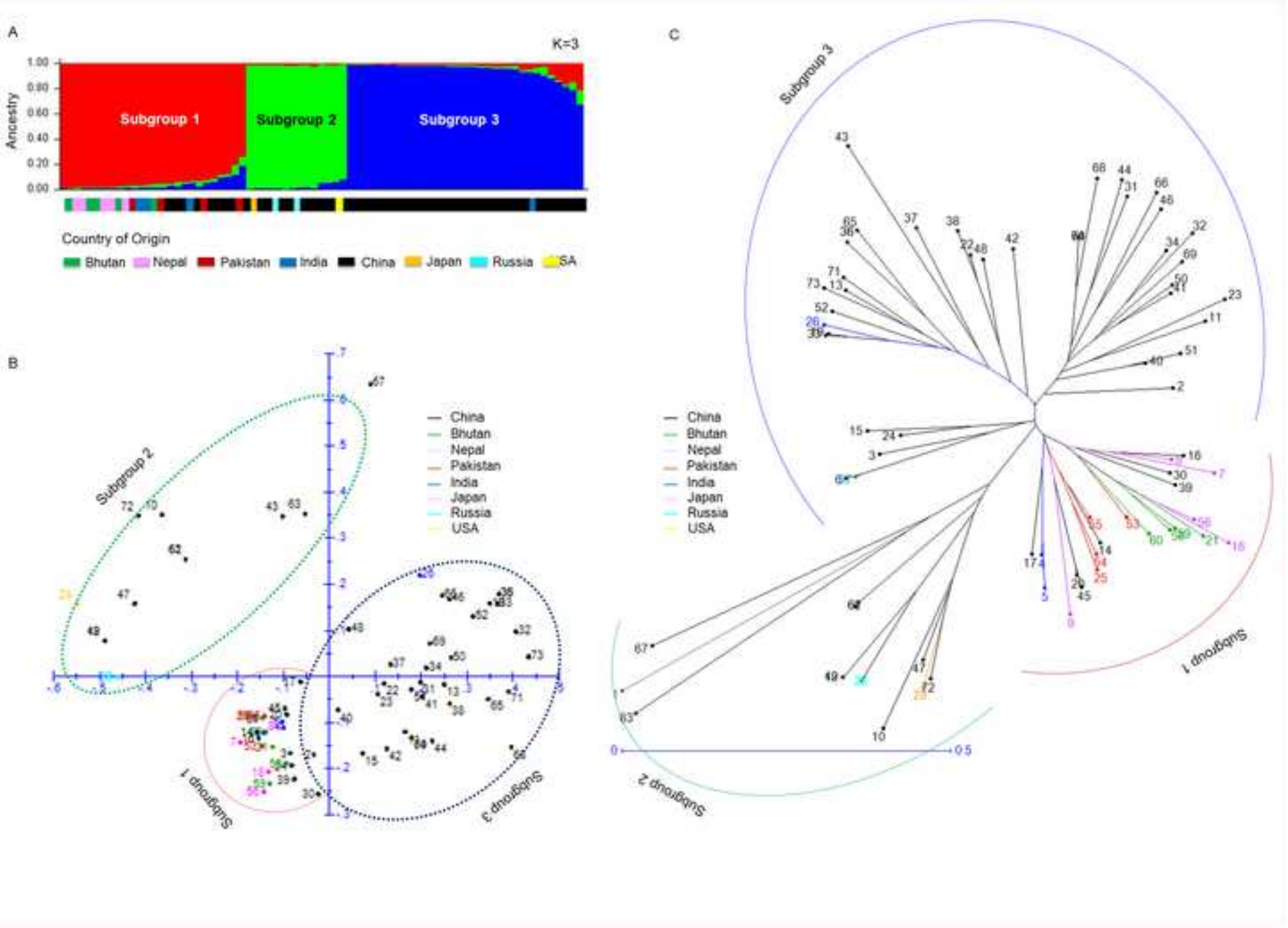

Supporting Information Figure 1

Click here to access/download

Supporting Information

S1 Fig.tif

Supporting Information Figure 2

Click here to access/download
Supporting Information
S2 Fig_raw_images.pdf

Supporting Information Figure 3

Click here to access/download
Supporting Information
S3 Fig.tif

Supporting Information Table 2

Click here to access/download
Supporting Information
S2 Table.docx

Supporting Information Table 3

Click here to access/download
Supporting Information
S3 Table.docx

Supporting Information Table 4

Click here to access/download
Supporting Information
S4 Table.docx

Supporting Information Table 5

Click here to access/download
Supporting Information
S5 Table.xlsx

Supporting Information Table 6

Click here to access/download
Supporting Information
S6 Table.xlsx

Supporting Information Table 7

Click here to access/download
Supporting Information
S7 Table.docx

Development of 50 InDel-based barcode system for genetic identification of tartary buckwheat resources

Short title: Identification of tartary buckwheat resources

Hwang-Bae Sohn[1¶], Su-Jeong Kim[1¶], Su-Young Hong[1], Sin-Gi Park[2], Dong-Ha Oh[3], Sunghoon Lee[4], Hwa Yeun Nam[1], Jung Hwan Nam[1], Yul-Ho Kim[1*]

[1]Highland Agriculture Research Institute, National Institute of Crop Science, Pyeongchang, Gangwon-do, Republic of Korea

[2]TheragenEtex Bio Institute, TherageneEtex Inc., Suwon, Gyeonggi-do, Republic of Korea

[3]Department of Biological Science, Louisiana State University, Baton Rouge, LA, United States of America

[4]EONE-DIAGNOMICS Genome Center Co. Ltd., Incheon, Republic of Korea

*** Corresponding author**

E-mail: kimyuh77@korea.kr (YHK)

¶ These authors contributed equally to this work

Formatted: Font color: Green

Abstract

Tartary buckwheat (*Fagopyrum tataricum* Gartn~~.),~~.) is a highly functional crop~~,~~ that is poised to be the target of many future breeding efforts. The reliable *ex situ* conservation of various genetic resources ~~are~~is essential for the modern breeding of tartary buckwheat varieties. ~~For this purpose, we~~We developed PCR-based and co-dominant insertion/deletion (InDel) markers to discriminate tartary buckwheat genetic resources. First, we obtained the whole genome ~~of~~from 26 accessions across a superscaffold-scale reference genome of 569.~~37Mb~~37 Mb for tartary buckwheat cv. ~~"~~"Daegwan 3-7."~~".~~ Next, 171,926 homogeneous and 53,755 heterogeneous InDels were detected by comparing 26 accessions with the ~~"~~"Daegwan 3-7"~~"~~ reference sequence. Of these, 100 candidate InDels ranging from 5~~–~~–20~~–~~ bp in length were chosen for validation, and 50 of them revealed polymorphisms between the 26 accessions and ~~"~~"Daegwan 3-7."~~".~~ The validated InDels were ~~also~~further tested through the assessment of their likelihood to give rise to a single or a few PCR products in 50 other accessions, covering most tartary buckwheat genome types. The major allele frequencies ranged from 0.5616 at the TB42 locus to 0.9863 at the TB48 locus~~ and~~, with the average PIC value ~~was~~of 0.1532 with a range of 0.0267~~–~~–0.3712. ~~Then, as~~To create a user~~–~~-friendly system, the homology of ~~genotype~~the genotypes between and among the accessions ~~can be~~were visualized in both one- (1D) and two-dimensional (2D)~~ as~~ barcode types by comparing amplicon polymorphisms with the reference variety, ~~"~~"Daegwan 3-7."~~".~~ A phylogenetic tree and population structure of the 76 accessions according to amplicon polymorphisms for the 50 InDel markers corresponded to those using non-synonymous sing nucleotide polymorphism~~SNP~~ variants, indicating that the barcode system based on the 50 InDels ~~were~~was a useful tool to improve the reliability of identification of tartary buckwheat accessions in the ~~germplsm~~germplasm stocks.~~–~~

Introduction

The eudicot family Polygonaceae consists of 27 reported species, including the 2~~two~~ cultivated species, common buckwheat (*Fagopyrum esculentum* Moench) and tartary buckwheat (*F. tataricum* Gaert.) [1,2]. Buckwheats (*F. esculentum* and *F. tataricum*) are cultivated~~,~~ as short-season pseudo-cereals~~,~~ in many countries and regions, including China, Nepal, Russia, Europe, Korea, and Japan [3,~~–~~4]. Following domestication in upland southwestern China, a multitude of tartary buckwheat landraces ~~was~~were created by ancient farmers through both artificial and natural selection [5]. ~~These~~After cultivation in China, these landraces were first ~~cultivated in China before~~ dispersed to ~~the neighboring regions of~~ Nepal, Russia, Europe, Korea, and Japan [5-9]. Several ~~factors~~characteristics of tartary buckwheat, such as ~~the~~ its self-pollinating nature ~~of~~

Formatted: Font color: Green

Commented [Editor1]: Tip: American English Style: Double quotation marks are used in American English; to denote a quote within a quote, single quotation marks are used.

Commented [Editor2]: Tip: American English Style: In American English, the closing quotation mark is placed after a comma or a period.

Commented [Editor3]: Tip: En Dashes: En dashes are preferred over hyphens for ranges. In this case, you have provided a range "5" to "20."

Commented [Editor4]: Remark: "Neighboring region" does not fit here, since Europe is not normally considered a neighbor to China.

~~tartary buckwheat~~ and ~~its adaptation~~ability to adapt to various regions (ranging from high altitude zones to lowland), led to both morphology and genetically ~~diverged~~divergent pockets of landraces [10-13]. These landraces ~~largely~~ have been largely replaced by ~~a few~~fewer landraces with specific desired characteristics, such as high-yield, drought resistance, and flavor [5,14].

Tartary buckwheat ~~has been~~ is a major food crop in high altitude zones~~, such as the~~ (e.g., high mountain areas of southern China and the Himalayan hills) due to its frost tolerance under poor soil conditions. ~~Recently, further uses~~Tartary buckwheat has also been used more recently for a variety of ~~tartary buckwheat vary widely~~purposes, ranging from various transitional food products to medical use [15,16]. The grain of tartary buckwheat is considered ~~as~~to be an ideal functional food source for humans because it is ~~rich~~richer in proteins, fats, vitamins, ~~as well as~~ rutin, quercetin, and other flavonoids ~~compared to~~ than common buckwheat [17-23]. The popularity of tartary buckwheat has been steadily increasing, and ~~cultivating~~its cultivation has ~~been~~ broadened ~~in places~~into areas far outside ~~the~~its original region ~~of tartary buckwheat cultivation, including reaching from high altitude zones to lowland~~ [10,~~-~~11,24,25]. This is a testament to tartary ~~buckwheat~~buckwheat's adaptability, which will be ~~crucially needed for agriculture in the face of various cultivation area and a~~crucial in Earth's changing climate [14]. Breeding for commercial varieties ~~in~~of tartary buckwheat ~~occurs to improve~~improves agricultural traits, such as lodging, late maturity, and low yield [26,27~~]. However,~~]; however, this cultivation resulted from ~~the~~a narrow genetic base ~~of the~~from a few varieties~~, these~~ only, which have produced modern varieties ~~bred via individual plant selection and hybridization breeding~~that are not well adapted to ~~the various local~~a variety of growing environments. The narrow genetic diversity within a commercial variety is due to the rigid quality required by farmers and processors, the finite use of exotic germplasm, ~~and the~~ restricted breeding strategies~~ likewise~~, and individual plant selection [28-30]. Thus, ~~it is important to have~~more genetic variation among tartary buckwheat genotypes ~~that can~~could be used ~~for improving a~~to improve a specific trait of interest due to the narrow genetic diversity of modern commercial varieties [14,31,32].

The identification of tartary buckwheat germplasms is vital ~~in~~to the enhancement of genetic diversity. ~~Altogether with morphological descriptors and researchers~~Researchers have been focused on developing molecular markers using germplasms and morphological descriptors to provide ~~a~~ high discrimination power. There are many methods for variety discrimination based on DNA ~~polymorphism~~polymorphisms, such as random amplification of polymorphic DNA (RAPD), amplified fragment length polymorphism (AFLP), inter-simple sequence repeat (ISSR), simple sequence repeats (SSRs~~)~~), and sing nucleotide polymorphism (SNP). Insertion-deletion polymorphisms (InDels) are gaining more attention among ~~the~~ molecular breeding scientists because they are easy to use, co-dominant (fully informative), and relatively abundant [33-40]. In tartary buckwheat, SSR [9], AFLP [41], RAPD [42,43], ISSR [44] and SNP [45,46] have been used to assess ~~the~~ genetic variation~~ of genetic resources. However,~~ but no study has ~~reported~~used a variety of discrimination ~~using~~methods with InDel markers ~~because of~~due to the difficulty ~~in~~ of finding DNA polymorphisms ~~among~~in tartary

Commented [Editor5]: Remark: This has already been stated and does not need to be repeated here.

Commented [Editor6]: Remark: I have shortened this sentence some to make it stronger.

Commented [Editor7]: Remark: Ensure that your meaning has been accurately retained here as this has been edited for clarity and better readability.

Commented [Editor8]: **Tip:** American English; Oxford comma: In American English, a comma is inserted before the coordinating conjunction preceding the last item in a list of three or more items. This comma, which was introduced by the Oxford University Press (hence called Oxford comma), is referred to as a serial comma.

buckwheat accessions.

~~A~~Until recently, a lack of large-scale genome ~~sequence~~sequencing information ~~has up till now has been under-represented in~~made the introduction of InDels as genetic markers in non-model species ~~such as~~(e.g., common ~~buckwheat~~ and tartary buckwheat~~.~~ ~~Recent~~) difficult. In recent years, published studies have ~~seen publications, including~~provided the draft genomes of common buckwheat [47], and a tartary buckwheat cultivar “Pinku1” [48] in the family Polygonaceae. More importantly, a wealth of polymorphism data can now be obtained from a massive amount of ~~sequence~~sequencing data ~~with~~using high-throughput sequencing technologies [49]. ~~Also~~In addition, [38] reported that the InDel-based barcode system ~~focused~~focuses on usability~~, providing~~ and provided an efficient resource management system. In addition to the InDel-based barcode system, ~~the integration of~~ morphological descriptors are now emerging that aim to identify tartary buckwheat accessions, ~~and~~which can be used to verify key genomic factors that explain or predict major agronomic traits.

We present a high-quality draft genome assembly and annotation for ~~a~~the tartary buckwheat~~.~~ *F. tataricum* cv. “Daegwan.” Using comparative genomics approaches, we revealed 171,926 homogeneous InDels and 53,755 heterogeneous InDels found in 26 tartary buckwheat accessions, which we compared to the draft genomes of the tartary buckwheat cultivar “Daegwan 3-7.” Among them, 50 polymorphic InDels from the 26 accessions were selected by gel electrophoresis, which ~~was~~were converted to ~~barcode types~~barcodes by comparing amplicon polymorphisms with the reference sequence. We also revealed the geographical distribution of 73 accessions through population structure analysis using the ~~barcode types.~~barcodes. For the genetic identification of tartary buckwheat resources, we made a user-friendly barcode system ~~using~~based on 50 InDel-based genotypes of 73 accessions. As a user-friendly system, the homology between the accessions can be predicted in both one (1D) and two-~~dimension~~dimensions (2D) as blocks. Our platform could be ~~not only~~ used in genetic research and breeding programs~~ but also used~~, as well as for efficient resource management ~~system in~~systems for tartary buckwheat.

Materials and methods

Plant m~~M~~aterials

~~Accessions~~ The accessions of 73 tartary buckwheat plants (S1 Table) were provided by the National Agrobiodiversity Center (Jeonju, Republic of Korea) and the Highland Agriculture Research Institute (Pyeongchang, Republic of Korea~~) and~~). The accessions were planted in a greenhouse located ~~in~~at the Highland Agriculture Research Institute (37°68′N and 128°73′E, 779 m altitude), Gangwon Province, Republic of Korea. These were self-pollinated 3~~three~~ times by the single seed descent (SSD) method by growing the individual plants in ~~flower pots~~flowerpots ~~as 10cm~~until they were 10 cm in diameter [50]. ~~Of these, we performed~~We grew an additional four generations of HLB1001 ~~with~~using the SSD method, which resulted in the selection of a progeny, "Daegwan 3-7" for a draft genome of tartary buckwheat. ~~For whole-genome resequencing, the plants of the~~A total of 26 accessions were collected from six countries ~~including~~ (China, India, and Nepal~~.~~) for whole-genome resequencing.

To investigate the biodiversity and comparative relationships~~, these~~ between the accessions, collected accessions were sown in 2017~~,~~ and grown in the ~~greeinhouse in~~ greenhouse at the Highland Agriculture Research Institute. We separated the ~~development stage of tartary buckwheat~~ plants ~~into three different stages~~by developmental stage: the vegetative growth stage (1–40 days after planting, DAP), ~~the~~ flowering stage (40–80 DAP), and ~~the~~ yield stage (80–90 DAP). At the yield stage, the color of the seed coat turned ~~into~~ black, ~~which means~~indicating that the seeds were ~~maturated~~ completely maturated.

Measurement of rutin and quercetin content

The yield stage seeds were ~~applied~~used to ~~measurement of~~measure their rutin and quercetin content. ~~The~~ First, the mature seeds ~~of~~from the 76 accessions were milled into a fine powder~~. The~~, then the samples were immediately flash-frozen in liquid nitrogen and stored at −80°C ~~for~~until further use. A total of 0.~~10g~~10 g dry powder for each tissue was mixed with 1 ml ~~of~~ methanol ~~and~~, then was extracted at 80°C ~~°C~~ for 1 hr in a conventional Soxhlet apparatus. The extract was filtered through a 0.20 ~~μm~~ μm syringe filter (PTFE 13 mm, PALL Life Sciences, Ann Arbor, MI) for rutin content [23]. Ultra-performance liquid chromatography (Waters Corporation, Milford, MA, USA) was performed on an Acquity 1-class using a C18 column (2.1 mm × ~~x~~ 100 mm, 1.7 ~~μm~~ μm) (Waters Corporation, USA) at 30°C ~~°C~~. The mobile phase (1% formic acid in water/0.1% formic acid in acetonitrile) flowed by the gradient elution method (S2 Table~~),~~) at a flow rate of 0.25 ml/min with a total injection volume of 10 μℓ. Rutin quantity was estimated based on the linear calibration curve of standard rutin and quercetin (Extrasynthese, France) under a detection wavelength of 259 nm. Three

Commented [Editor9]: Remark: Please list all 6 countries.

Formatted: Font color: Green

Commented [Editor10]: Remark: Would "sample" be a better word here? Please clarify.

Commented [Editor11]: Tip: Scientific notation and convention: Use a multiplication sign (×) instead of the letter x at such instances. Note that a space should be inserted before and after the sign.

independent sample analyses were performed for each tissue. All statistical ~~analysis~~analyses ~~was~~were performed using ver. 3.6.1 of the R software [R].

DNA sequencing and de novo assembly

After 21 DAP, the leaves in the third node of the progeny "Daegwan 3-7" were ~~applied~~used to create a draft genome. ~~Three~~First, 3-week-old leaves ~~of~~from the bulk of 10 plants were collected for genomic DNA extraction using a standard CTAB (cetyl trimethylammonium bromide) protocol [52,53]. ~~For the entire genome of *F. tataricum*, both~~Both short read (Illumina) and long read (PacBio) libraries were prepared according to the manufacturer's instructions~~.~~ for the entire genome of *F. tataricum*. A short insert paired-end (PE) ~~libraries were~~library was prepared using a Illumina TruSeq Nano DNA Sample Preparation Kit (Illumina Inc., San Diego, Ca, USA) and 1~~~~~–3 µg of DNA. ~~After~~A library profile analysis ~~with~~was performed using an Agilent 2100 Bioanalyzer (Agilent Technologies, USA) and qPCR quantification, then the libraries were sequenced to 2 × ~~x~~ 100 bp on the Illumina HiSeq 2500 platform (Illumina, USA) ~~by~~from the TheragenEtex Bio Institute (TheragenEtex Inc., Suwon, Republic of Korea). PacBio SMRTbell libraries (~~2kb, 5kb, 10kb~~2 kb, 5 kb, 10 kb, and ~~15kb~~15 kb inserts) were prepared with the standard PacBio library preparation protocols, which ~~is~~are available at http://pacificbiosciences.com/. The sequencing was conducted on a PacBio RS II (Pacific Biosciences, USA) system using C4 chemistry and 240 min movies by the TheragenEtex Bio Institute.

The short and long reads were assembled separately. The de novo assembly of the PacBio long reads was performed using Fast Alignment and CONsensus [54] with default parameters. The short reads ~~was~~were assembled with SOAPdenovo2 [55] with default parameters. The initial contigs were merged using HaploMerger2 [Huang et al.~~,~~ 2017]. Both short and long reads were then used to construct scaffolds with SSPACE [Boetzer et al.~~,~~ 2011] and GapFiller [Ref], which led to the superscafold-scale draft genome of tartary buckwheat cv. "Daegwan 3-7~~"~~." The N50 length of the final genome assemblies was evaluated. To check the quality of the assembly, BAC-based sequences of *FtFLS1* (*F. tataricum* Flavonol Synthase 1) and *FtFLS2* were aligned against the reference genome of tartary buckwheat cv. "Daegwan 3-7~~"~~." The number of errors per base ~~was~~was estimated according to the sequence variants between them.

Identification and validation of the polymorphic InDels

Commented [Editor12]: Remark: Consider replacing with "sample" or "tissue sample."

Formatted: Font color: Green

Formatted: Font color: Green

For Genomic DNA was extracted for whole-genome resequencing, genomic DNA was extracted – from the 26 accessions that had self-pollinated 3three times by using the SSD method. Three-week-old leaves offrom the bulk of 10 plants were collected for genomic DNA extraction using a standard CTAB (cetyl trimethylammonium bromide) protocol [52,-53]. The data consisted of 101-bp reads, which were generated using the HiSeq 2500 sequencer. The Short Oligonucleotide Alignment Program 2 (SOAP2) was used to map the raw pair-endsend reads onto the reference genome (tartary buckwheat cv. ""Daegwan 3-7""). For each accession, more than 93% of the reads were properly aligned to the reference genome. Insert size was estimated by mapping the reads to the reference genome using the Burrows-Wheeler Aligner algorithm (bwa) [58] ver 0.5.9. The aligned reads were realigned at InDel positions withusing the GATK InDelRealinger algorithm [59] for enhancingto enhance the mapping quality. The base quality scores were recalibrated by using the GATK TableRecalibration algorithm. The primer pair used to amplify each of the InDels were selected by the Primer3 software (http://primer3.sourceforge.net). We selected InDels of abovewith more than 10– bp and designed the primers, according accordingly to match the characteristics of each InDel by using the Primer 3 software.–

Formatted: Font color: Green

Amplicon polymorphism assay and bar-coding process

The PCR analysis was performed using 10– $\mu\ell$ of $\mu\ell$ reaction mixtures containing 20– ng of total genomic DNA, 2– pM of primer, and 5– $\mu\ell$ of GoTaq Green Master Mix (Promega, Madison, WI, USA). The PCR was performed under conditions ofat 95°C℃ for 5– min and s, followed by 35 subsequent 35 rounds ofat 94°C ℃ for 30– sec, 45°C ℃ for 30– sec and 72°C ℃ for 30– sec, using a Mastercycler pro 384 (Eppendorf, Germany). The PCR products were separated by electrophoresis in 3% gels of certified low range ultra-agarose (Bio-rad) followed by LoadingSTAR staining (Dynebio, Republic of Korea). For the limitation of the number of candidates, we chose primer pairs that amplified PCR products that were 150~–231– bp long. The allelic diversity of the InDels with the e-PCR products in 26 tartary buckwheat genomes waswere assessed byvia PIC (Polymorphism Information Content), which was defined as PIC $i = 1 - \sum_{j=1}^{n} p_{ij}^2$, where p $_{ij}$ is the frequency of the jth pattern for the ith marker [60,61].

Commented [Editor13]: Remark: Please note that some journals require equations to be given on a separate line. Please check with your journal for their requirements.

The 50 InDel markers were selected according to their genotyping success and PCR band size. The discriminating power of the selected 50 InDel set for tartary buckwheat identification was evaluated withusing the 76 tartary buckwheat accessions. Primers were designed by targeting the InDel region, in such a way that the genotypes of tartary buckwheat accessions would produce the same or different (insertion or deletion) amplicons relative to the reference genome, ""Daegwan 3-7."". Based on the results of InDel marker amplification, the same result with the reference genome was represented by an ""a,"", thewhile other results were represented by a ""b."". For bar-coding

Formatted: Font color: Green

representation of the results, “a” and “b” were converted to white and black, respectively. The homology of the accessions ~~was~~were calculated after~~ the~~ PCR amplification of all 50 InDels.

Phylogenetic analysis and population structure among 73 tartary buckwheat accessions

The discriminating power of the selected 50 InDel set for tartary buckwheat identification ~~was~~were evaluated ~~with~~using the 76 tartary buckwheat accessions. The analysis was carried out with POWERMARKER Ver. 3.23 (Liu and Muse 2005; http//www.poxermarker.net). A phylogenetic tree for the 73 accessions was drawn based on the genotypes defined ~~using~~with the 50 InDels using the weighted neighbor-joining method with simple matching coefficients~~ implemented~~ in the DARwin software [55]. To delineate clusters of individuals on the basis of their genotypes, the model-based STRUCTURE 2.3.4 [63] was used with the admixture model~~;~~, a burn-in period of 100,000 iterations, and ~~the~~ data from the 50 InDel loci. The number of clusters (k) was set to five, as this number maximized the △k (ad hoc criterion) parameter (Evanno et al. 2005). We used simulations with k values ranging from 2 to 10~~,~~ with 5~~five~~ replications~~,~~ to calculate the LnP(D) value. The optimal k value can be chosen based on the maximum LnP(D) that provides a distinct population structure. On this basis, we chose K~~=~~ = 3 (three ancestral groups) for the tartary buckwheat populations. To check the quality of these analyses, a phylogenetic tree and a population structure for the 26 ~~resequenced~~re-sequenced accessions ~~was~~were drawn based on the genotypes defined using the 6,622 non-synonymous~~synonymous~~ SNPs.

Formatted: Font color: Green

Results

Genome assembly and whole-genome sequencing of tartary buckwheat accessions

~~To exclude any possible heterogeneity in the original seed lot, we performed~~We grew seven generations of tartary buckwheat cv. “Daegwan” ~~with~~ using the SSD~~single seed descent (SSD)~~ method and an isolated ~~an~~ individual (designated as “Daegwan 3-7”~~).~~

Formatted: Font color: Green

Genotype	IT Number [a]	Country of origin	Genome coverage (%) [b]	Mean depth

~~For a reference genome, we~~) to exclude any possible heterogeneity in the original seed lot. We produced a total of 43.83 and 32.~~17Gb~~17 Gb sequences from Illumina PE~~paired ends (PE)~~ and Single-Molecule Real-Time (SMRT) sequencing platforms, respectively, which corresponded to 78x (Illumina PE, S2 Table) and 58x (SMRT, S3 Table) coverages (S1 Fig~~).~~) for the reference genome. Combining the sequencing strategies of PacBio and Illumina Hiseq2500, we obtained a final draft assembly of 569.37~~–~~ Mb in 2,886 scaffolds with 50% of the total sequence captured in 156 scaffolds larger than 886,~~968bps~~968 bps (Table 1, N50).

Table 1. Summary of *Fagopyrum tataricum* draft genome assembly process~~.~~

Steps	Type	No. of Sequences	Total bases	Longest sequence	N50 sequence	N90 sequence
Illumina assembly	Contigs	37,711	416,936,361	318,774	29,297	5,269
PacBio assembly	Contigs	2,231	450,944,578	5,290,013	650,667	148,793
Hybrid assembly	Contigs	4,433	565,095,288	5,290,013	463,432	36,547
Scaffolding~~–~~/~~-~~Gap-filling	Scaffolds	2,886	569,372,063	5,875,542	798,933	61,611

~~For whole genome resequencing, we~~We selected ~~the~~ 26 tartary buckwheat accessions ~~that were~~for whole-genome resequencing. These accessions originated or were popularized in different Asian and international regions, including ~~fifteen Chinese, four~~15 landraces from China, 4 from Nepal, ~~four~~4 from India, ~~one~~ 1 from the USA, ~~one~~1 from Bhutan and ~~one~~1 from Pakistan ~~landraces~~ (Table 2). After 3~~three~~ additional generations ~~with~~were grown using the SSD method, a panel of the 26 genotypes showed a broad extent of rutin content and phenotype variation (Figs 1 and 2). The 26 accessions showed a various distribution of rutin content compared to those of the 73 accessions (Fig 2). In particular, HLB1013 had the highest rutin content (1,921~~–~~ mg/100g·DW), while "~~"~~HLB1006"~~"~~ had the lowest (1,250~~–~~ mg/100g·DW) among the 26 accessions. ~~Among the accessions, large~~Large differences ~~showed~~were found in seed shape and seed coat color~~.~~ among the 26 accessions (Fig 1). Seed information ~~complied~~conformed to ~~know~~ the known genetic variation of the 26 accessions.~~ Fig 1 shows large differences in seed shape and seed coat color among the accessions.~~

Table 2.Genome coverage and mean depth after the whole~~-~~ genome resequencing of 26 accessions~~.~~

Formatted: Font color: Green

HLB 1001	IT 224676	USA	94.80	24.28
HL1B 002	IT 225083	China	93.64	26.60
HLB 1003	IT 225084	China	94.00	23.25
HLB 1004	IT225085	India	93.63	23.78
HLB 1005	IT225086	India	93.36	26.40
HLB 1006	IT225087	India	94.53	26.20
HLB 1007	IT225088	Nepal	92.99	24.58
HLB 1008	IT225089	Nepal	93.47	24.28
HLB 1009	IT225090	Nepal	93.21	27.30
HLB 1010	IT226673	China	93.64	24.68
HLB 1011	IT226674	China	94.22	25.89
HLB 1012	IT226675	China	94.57	25.79
HLB 1013	IT226676	China	93.92	24.44
HLB 1014	IT226677	China	94.06	25.74
HLB 1015	IT226678	China	94.19	25.53
HLB 1016	IT226679	China	94.01	24.93
HLB 1017	IT226680	China	94.18	25.66
HLB 1018	IT226682	Nepal	93.14	24.69
HLB 1019	IT261917	China	94.23	25.13
HLB 1020	IT261922	China	94.89	23.70
HLB 1021	IT261924	Bhutan	93.70	25.21
HLB 1022	IT278316	China	94.35	24.44
HLB 1023	IT278317	China	94.05	26.99
HLB 1024	IT278318	China	94.56	23.90
HLB 1025	IT278319	Pakistan	94.60	25.85
HLB 1026	IT226681	India	93.98	26.65

[a] National registration number managed by Nation Agrodiversity Center in Republic of Korea.
[b] Genome coverage was calculated based on a 569 Mbp genome size and 101 bp paired-end Illumina reads.

Fig 1. The seed morphology of 26 re-sequenced tartary buckwheat accessions and Clfa41.

Fig 2. Rutin content of 73 tartary buckwheat accessions according to country of origin. The phenotypic segregation is shown in box-plot format. The interquartile region, median, and range are indicated by the box, the bold horizontal line, and the vertical line respectively. Etc. includes 4~~four~~ accessions~~ came~~; 1 from ~~one~~the USA, ~~one~~1 from Japan, ~~two~~and 2 from Russia. ~~For~~We selected 26 accessions for whole-genome resequencing~~, we selected 26 accessions~~ that originated or were popularized in different Asian and international regions. For expanded details of the 73 tartary buckwheat accessions, see ~~S1~~ Table S1.

We produced paired-end DNA reads ~~of~~at 23–27-fold depths (Table 2) for the 26

Commented [Editor14]: Remark: I'm not sure what this is referring to. Please note that this was pointed out by a reviewer as well. Make sure to change this prior to re-submission.

~~accessions~~ accession genomes and mapped them to the reference genome (“~~“~~Daegwan 3-7”~~”~~). The sequencing qualities of all the samples were high: 93~~%~~ %–95% of the accession sample reads were mapped to the reference genome, and 96~~%~~ %–97% of the reference genome was covered (~~S3~~ Table S3). The genetic variations (SNPs and small InDels) among the 26 accessions collected from the six countries were individually identified by Samtools and the Genome Analysis Tollkit GATK (see Methods). To increase the accuracy of the prediction, only the variations predicted by both methods were used for subsequent analysis. Bioinformatic analysis revealed 171,926 and 53,755 homogeneous and heterogeneous InDels, respectively (Fig 3). The heterozygous rates of all accessions were around 10%, reflecting the possibility of cross-pollination although self-fertilization ~~(S4~~ was used (Table S4).–

Fig 3. Number of InDels in 26 tartary buckwheat accessions aligned with reference genome, “~~“~~Daegwan 3-7.”~~”.~~ The number of variants homogeneous to the alternative genotype; both alleles are ~~same~~identical to the alternative genotype. The number of variants heterogeneous to the genotype; one allele is ~~same~~identical to the reference genome, and another is identical to the alternative genotype.

Commented [Editor15]: Remark: This was not originally clear. Please confirm the accuracy of the changes made.

Selection of 50 InDels and constructing barcode-type database of 73 accessions

Sequence comparison at the nucleotide level of the tested genomes with the reference genome revealed InDels ~~which can~~that could be easily used ~~for identifying~~to identify tartary buckwheat resources in common laboratories. Consistent with what has been found in other organisms [38,64], the great majority of homogeneous InDels ~~in~~from all accessions were short with a dominance of 4-bp or fewer events (Fig 3). From ~~these~~this polymorphism data, we chose ~~above~~InDels with more than 10-bp ~~InDels~~ for further analysis ~~that can~~to help us identify them ~~by~~via PCR and gel -electrophoresis. We tested ~~the~~ 100 primer sets, focusing on their ability to amplify PCR products in the 26 genome types, since this study aimed to establish PCR-based markers applicable for all tartary buckwheat accessions. PCR primers were designed to generate amplicons of varying sizes within the 150~~~~~–231– bp interval (~~S5~~ Table S5). Among them, 50 InDel loci were widely distributed on whole chromosomes in the reference genome (“~~“~~Pinku 1”~~”~~) reported previously (Fig 4). We selected the 50 InDels to validate the prediction accuracy and found that 98.2% of the PCR results in the 26 accessions were consistent with the predicted results. The results of the InDel analyses were highly accurate (Tables S5 and S6 ~~Tables~~). This high accuracy provided a solid foundation for our data analyses and ~~makes available~~provides high-quality data for future data mining.

Fig 4. Schematic of the chromosomal distribution of the tartary buckwheat InDels~~.~~ The number of ~~the chromosome~~chromosomes is shown on each chromosome of ~~a~~ the

reference genome ~~of~~from “~~“~~Pinku1.”~~”.~~

Primers were designed by targeting the InDel region~~,~~ in such a way that the genotypes of the 26 accessions would produce the same or different (insertion or deletion) amplicons relative to the reference genome (Fig 5). By using the previously reported barcode method [38,39], ~~the same~~identical results ~~with~~to the reference genome “~~“~~Daegwan 3-7”~~”~~ were represented by an “a” ~~and the~~,” while other results were represented by “b~~”,~~,” which were presented as “white” and “black” ~~barcode~~barcodes, respectively. Fig 5 shows ~~that~~ the barcode system ~~was~~ applied to the tartary buckwheat ~~accession~~accessions “~~“~~HLB1007”~~”~~ and “~~“~~HLB1008,”~~”,~~ which were converted to the standard 1D and widely~~-~~-used 2D barcode types ~~according to~~by comparing their amplicon ~~polymorphism~~polymorphisms to the reference genome, “~~“~~Daegwan 3-7.”~~”.~~ This result highlights that the barcode types can be effectively used to investigate the degree of marker difference ~~of~~in tartary buckwheat accessions (Fig 6). The ~~barcode system with the selected 50 InDel set was built, and their~~creation, stability and quality of the barcode system for genetic identification ~~were~~using the selected 50 InDel is thoroughly evaluated below.~~-~~

Fig 5. Application of tartary barcode system to tartary accession, “~~“~~HLB1007”~~”~~ and “~~“~~HLB1008.”~~”.~~ (A) Amplification products and barcode~~-coding~~ representation of the polymorphisms using InDel marker TB4 on 26 tartary buckwheat accessions. PCR results using the 50 InDels in (B) “~~“~~HLB1007”~~”~~ and (C) “~~“~~HLB1008”~~”~~ were converted to the standard 1D and widely~~-~~-used 2D barcode types in comparison with those in “~~“~~Daegwan 3-7.”~~”.~~ ~~The same~~Identical results ~~with~~to the reference genome were represented by white, ~~the~~ while other results were represented by blue.

Fig6. Comparison of 2-dimensional barcode patterns between “~~“~~HLB1007”~~”~~ and “~~“~~HLB1008.”~~”.~~ ~~The same~~Identical results ~~with each other were~~are represented by white, while the other results are represented by blue. Seed morphology of “~~“~~HLB1007”~~”~~ and “~~“~~HLB1008.”~~”.~~

By building a database of the 50 InDel polymorphisms ~~in~~for the 73 tartary buckwheat accessions, we have established a more stable foundation for utilizing the barcode system to provide a promising tool for identifying the genetic resources of tartary buckwheat. Allele frequencies and average PIC value of the 50 InDel loci ~~were~~are shown in Table 1. The major allele frequencies ranged from 0.5616 at the TB42 locus to 0.9863 at the TB48 locus. The average PIC value was 0.1532, with a range of 0.0267~~-~~–0.3712, which implied that the selected InDels could be applied for investigating ~~polymorphism~~the polymorphisms of tartary buckwheat accessions (~~S7~~ Table S7). The ~~genotype~~genotypes of the 73 tartary buckwheat accessions ~~was~~were constructed with a two-dimensional barcode, and genotypic discrimination was analyzed by comparing it with the accessions of the barcode system. The average difference between the analyzed accessions was 7 InDels from a total of 50 InDels, while 6~~six~~ accessions had differences in 12 InDels compared to ~~a~~ the reference genome, “~~“~~Daegwan 3-7.”~~”.~~ By comparing the

> **Commented [Editor16]:** Remark: Please confirm. Elsewhere you state that white and black were used. Be sure to adhere to consistency throughout your entire manuscript.

two-dimensional barcodes of the accessions, different blocks ~~can~~could be visually ~~easily~~ distinguished with ease, so that the genotype differences of the accessions ~~can~~could be determined (Fig 6). The discrimination power of genetic resources was high enough to broaden our understanding of both phylogenetic signals and population-level variation.

Phylogenetic analysis and population structure in tartary buckwheat accessions

Phylogenetic analysis of the 6,622 non-synonymous SNP variants and population structure subdivided the panel into 2~~two~~ primary populations (Fig 7): the reference group and the non-reference group. ~~And a~~A similar result was ~~given that~~attained with the SSR data, which indicated ~~–~~two separate ~~groups~~group clusters of tartary buckwheat ~~were clustered by using SSR data~~ [9]. The 23 accessions ~~demonstrated by~~had 4 subgroups within the non-reference group, and were classified according to ~~the~~ their collection area. The phylogeny resolved subgroup 2 (~~three~~ 3 Nepal, ~~one~~ 1 Bhutan, and ~~two~~2 China) as closer to subgroup 1 (~~eight~~8 China and ~~one~~1 India) than subgroup 3 (~~three~~3 India, ~~one~~1 Pakistan, and ~~one~~1 Nepal).~~–~~

Fig 7. Diversity analysis of 26 re-sequenced tartary buckwheat accessions. (A) Phylogenetic analysis based on non-synonymous SNPs. (B) STRUCTURE [63] classification and (C) ~~Principal~~principal component analysis were similar. Each color represents country of origin.

To validate the reliability of the 50 InDel markers for discriminating between tartary buckwheat accessions, bin maps ~~with the InDels~~ were constructed for 73 tartary buckwheat accessions ~~(S7~~ using the InDels (Fig S7). We analyzed the population structures of the studied ~~accession~~accessions and ~~referenced group.~~reference groups. The result ~~showed~~are shown in Fig 8. At K~~=~~ = 3, the clusters were anchored to Southeast Asia and China. Among subgroup 1, the accessions in Southeast Asia were ~~constituted~~ almost entirely ~~by~~composed of the red component, while the Chinese accessions were composed of the red, green, and ~~and~~ blue ~~component.~~components. Subgroup 1 mainly consisted of accessions ~~in~~from Southeast Asia (65.4%), while subgroup 2 was comprised of Chinese accessions (71.4%), and subgroup 3 consisted of Chinese accessions (97.0%). At the same time, the Chinese ~~accession~~ accessions in subgroup 1 and 3 showed a mixed ~~constitution~~composition of both red, green, and ~~and~~ blue components. ~~Also, a principal component analysis (~~A PCA~~)~~ also provided similar results, with the accessions forming a tight cluster ~~that is separate~~separated by ~~the~~ collection region.~~–~~

Fig 8. Diversity analysis of tartary buckwheat accessions. (A) STRUCTURE [63] results based on 50 InDels: 73 accessions were divided in 3~~three~~ subpopulations. (B)

Principal component analysis and STRUCUTURE classification were similar. Each color represents country of origin. (C) Phylogenetic analysis among 73 tartary buckwheat accessions ~~by~~ using weighted neighbor-joining analysis in DARwin software [62]. The horizontal bar indicates distance based on the simple matching coefficient. Each subgroup represents a subpopulation based on STRUCTURE results.

Weighted neighbor-joining (NJ tree) ~~relationship~~relationships with ~~the~~ bin maps revealed 3~~three~~ groups (G1, G2, and G3) that contained 40, 16, and 17 accessions, respectively (Fig 8). The accessions collected in Nepal and Bhutan demonstrated substructure within group 1, being the genotypes closest to the reference group~~, a~~. A different result revealed that two separate groups were clustered by using non-synonymous SNP variants (Fig 7). The color-coded branches supported the classification of the 73 accessions by the collection region (Fig 8). In the phylogenetic analysis, PCA and population structure indicated that the tartary buckwheat accessions segregated into different groups that reflected their geographical distribution. With the evidence relating ~~of~~ the 50 InDels to environmental adaptability, these results are consistent with the segregation of the tartary buckwheat into different groups according to their geographical distribution. These results show the practicability of the barcode system, which can ~~then~~ be used to identify genetic variation ~~that is~~ associated with phenotypic variation of functional relevance, as well as markers that can be used in crop breeding.

Discussion

~~Tartary buckwheat has been a crop species of interest for various reasons including the higher levels of rutin compared to the common buckwheat [10,23,65]. Thus, tartary~~Tartary buckwheat is an important crop that is poised to be the target of many future breeding efforts. It is a crop species of interest for various reasons, including its higher levels of rutin compared to the common buckwheat [10,23,65]. For breeding commercial varieties of tartary buckwheat, the elite germplasm needs to be expanded by adding ~~the~~ various genetic ~~resource,~~ resources. This is a field that is still under-represented ~~in~~ at the genetic level, despite its vast germplasm diversity ~~as well as~~ and its importance ~~as a field enriched~~ in stress~~-~~-adapted resources [66-70]. Particularly, since these resources possess many distinct traits, we used 73 genetic resources of tartary buckwheat ~~constitute~~to create a framework for comparative ~~analyses.~~analysis. These resources ~~are different~~differ in seed morphology (Fig 1~~). Most of all, there is various distribution in~~) and rutin content ~~among the genetic resources~~ (Fig 2). ~~Also, these~~ These differences might result in distinct responses to UV-B~~ and~~ , drought [69~~]~~], and salt ~~stresses~~stress [67]. A recent publication of tartary buckwheat genomes provided the reference genome needed to study the molecular markers ~~for distinguishing the~~ to distinguish between these genetic resources [48]. The narrow genetic diversity seen within a commercial variety ~~is due to~~has various ~~reasons~~causes, including the rigid quality required by farmers and processors [28,30]. Thus, molecular markers are

Commented [Editor17]: Remark: This sentence fits better here.

imperative to understand the genetic differences of tartary buckwheat accessions~~ that may lead~~, which could be used to ~~broadening~~broaden the genetic resources of commercial breeding programs. To address this, we made a user-friendly barcode system using 50 InDel-based genotypes of 73 accessions. Our barcode system could be ~~not only~~ used in genetic research~~ and~~, breeding programs~~ but also used for~~, and efficient resource management ~~system in~~systems for buckwheat.

High-throughput sequencing technologies [49] ~~offers~~offer immense possibilities for generating a massive amount of ~~sequence~~sequencing data from hitherto uncharacterized genomes, which facilitates the introduction of InDels as genetic markers in non-model crops, like tartary buckwheat. Given that the existing reference genome for" Pinku 1" was from China [48], we constructed a new superscaffold-scale reference genome for a variety of "Daegwan 3-7" from the Republic of Korea~~,~~ by combining the sequencing strategies of PacBio and Illumina Hiseq2500. Using this integrated approach, we obtained an N50 scaffold size of 463,~~432bps~~432 bps for the draft genome of "Daegwan 3-7" (Table 1). To explore the genomic variation of tartary buckwheat accessions, we performed ~~the~~ whole-genome sequencing ~~of~~for 26 accessions across the reference genome ("Daegwan 3-7") (Table 2). Significantly, the depth (23~~×~~ × 27 ×) of sequence coverage ~~means~~indicates that it was well-suited for the sequence analysis of the 26 genetic resources. From these genome comparisons, 171,926 homogeneous InDels and 53,755 heterogeneous InDels were obtained with an InDel density of 2 for every 1 kb through pairwise comparison with the reference genome (Fig 3 and ~~S5~~ Table S5). Among them, 50 polymorphic InDels were selected by gel electrophoresis, which PCR results were 98.2% matched with the predicted results (Tables S6 and S7 ~~Tables~~). Moreover, the 49 InDels were distributed widely in the whole genome of "Pinku 1." ~~Especially, consider as~~ Considering that the TB8 locus ~~unfound~~was not found in "Pinku 1," the two reference genomes for "Pinku 1" and "Daegwan 3-7" ~~will~~can provide backbones and ~~the~~ references for developing the pan-genome to reduce reference bias. We ~~will work~~plan to continue to improve the draft genome of tartary buckwheat cv. "Daegwan 3-7" for use in the pan-genome. Our results suggest that superscaffold-scale *de novo* draft genomes usually ~~facilitates~~facilitate the introduction of InDels as genetic markers in non-model crops, like tartary buckwheat.

To efficiently identify tartary buckwheat accessions, we developed the barcode system using ~~the~~ 50 InDels ~~which were~~ selected from ~~the~~ 26 landrace ~~genome~~genomes using a resequencing~~ sequencing~~ strategy. The 2D-barcode system (Figs 5 and 6) is a powerful tool to compare the genotypes of tartary buckwheat accessions [38-40]. The landrace ~~collections~~genomes collected from six countries~~, including China, India, and Nepal, and contains~~ contained a wealth of genetic diversity adapted to wide-ranging environmental conditions (Table 2). Through the application of 50 InDels to this collection, we found that both geographical and environmental factors have shaped the genetic diversity of tartary buckwheat landraces (Fig 7). In the 73 accessions, the population structure analyses using the DNA barcode data revealed their geographical distribution (Fig 8), which is geared for adaptation in the diverse topographic conditions of the agroecosystem [71-74]. In the phylogenetic analysis using non-synonymous SNPs, the 26 accessions showed a close relationship with those from Bhutan and Pakistan. (Fig 7).

Commented [Editor18]: Remark: Is there a missing number after the last "×"? Please clarify.

This observation is consistent with a previous study, which found that ~~the~~ population genetic structure analysis ~~by~~ using SSR data clustered one group that was mainly distributed in Nepal, Bhutan, and the Yunnan-Guizhou Plateau regions of China and ~~the other~~a second group that was principally derived from the Loess Plateau regions, Hunan and Hubei of China and the USA [9]. These findings were consistent with the previously suggested method of seed exchange within a close geographic distance [5,~~-~~8]. Altogether with SSR [9], AFLP [41], RAPD [42,43], and ISSR [44], the barcode system using InDels can complement ~~the~~ current research and broaden our understanding of both phylogenetic ~~signal~~signals and population-level ~~variation~~variations [38-40]. These phylogenetic and structural analyses in the tartary buckwheat accessions will serve as a significant resource to gain more insight into the evolution of lineage-specific gene networks associated with the environmentally-specific developmental and adaptive ~~trait in~~traits of tartary buckwheat landraces.

According to their position in unigenes, the 50 InDels ~~are~~were divided into five types~~,~~: coding region, 5′ untranslated region, 3′ untranslated region, splice region, and intergenic region (~~S7~~ Table S7). The InDels may be biased toward changes in gene expression via cis-regulatory mutations [75]. The InDels ~~in~~of certain genes, e.g., *RPP4* [76], *UGT73B5* [77], *TPS4* [78], and *DREB3* [79] have been postulated to play a role in different responses to environmental stresses during growth and survival. With the evidence relating ~~of~~ the 50 InDels to environmental adaptability, these results are consistent with the segregation of the tartary buckwheat into different groups according to their geographical distribution.~~–~~

Despite self-fertilizing the tartary buckwheat~~,~~ through 3~~three~~ additional generations ~~with~~using the SSD method, around 10% heterogeneous InDels were detected in the genomic DNA from the 26 progenies (~~S3~~ Table S3). Previous studies have shown that farmers cultivated several tartary buckwheat landraces suitable for the multiple local microclimates and environmental conditions [5], which might drive the gene flow of landraces within a region [80]**.** In this study, we uncovered strong support for allogamy among landraces that contributes to heterogeneous InDels in tartary buckwheat, which ~~might~~could drive the genetic diversity of landraces within a region. ~~Besides, considering~~Considering the effect of heterozygous InDel on the increase of single nucleotide mutation rate [75,81], it might be an important ~~how~~characteristic that allows tartary buckwheat ~~can~~to adjust to the diverse topographic conditions of the agroecosystem. These results suggest that the divergent gene expression of the InDel loci may affect ~~the~~its adaptation to environmental conditions.

We found that ~~the~~ tartary buckwheat accessions ~~were~~could be distinguished by ~~the~~ 50 InDels based on genotype according to ~~locations~~location with the differing environment (Figs 7 and 8). Tartary buckwheat is a diverse crop in which separate domestication events happened in each gene pool, followed by race and market class diversification that has resulted in different morphological characteristics in each commercial market class [82]. Combining multiple data types can compensate for missing or unreliable information in any single data type. Importantly, ~~the~~ complete resource management is

only likely to be discovered if the genotype, phenotype, and chemotype of tartary buckwheat accessions are considered in an analysis. To make a user-friendly common platform for genotype, phenotype, and chemotype resources, we ~~will~~plan to incorporate genotypic data with ~~that~~ phenotype and chemotype (rutin content) data ~~of~~ for tartary buckwheat accessions (~~S2~~ Fig S2). Our platform could be ~~not only~~ used in genetic research~~ and~~, breeding programs~~ but also used~~, and for efficient resource management ~~system in~~systems for buckwheat. ~~Especially, increasing~~Increasing tartary buckwheat productivity will require adapting the crop to the agricultural environment of new locations. The barcode system can assist tartary buckwheat breeders in identifying germplasm accessions and can be used as donor parents for breeding tartary buckwheat cultivars.

Author contributions

HBS, SJK, SL and YHK conceptualized the research. HBS, SGP, DHO, and YHK were involved in data curation, and formal analysis. SJK, and YHK strived for funding acquisition, and project administration. YHK supervised the project (Project No. PJ01189401) which was managed by National Institute of Crop Science. HBS, SJK, and YHK took a part in investigation, methodology, validation, visualization. SJK, YHK, SYH and JHN provided resources for the research. HBS and HYN performed the role of writing-original draft preparation. HBS and YHK played the role in writing-review & editing.

~~HBS, SJK, SGP, and and YHK performed the experiments. HBS, SJK, DHO, SL, JHN, and and YHK analyzed the data. HBS, HYN, and and YHK drafted the manuscript. HBS, SJK, and and YHK designed the project. YHK supervised the project and complemented the writing.~~

Acknowledg~~ements~~ments

Sincere thanks to Misook Lee for the cultivation of tartary buckwheat resources, Juyoung Shim for the DNA extractions of samples, and Hyeongjin Shin for the technical assistance in the laboratory.

References

1. Ohnishi O, Matsuoka H. Search for the wild ancestor of buckwheat II. Taxonomy of

Formatted: Font color: Auto

Fagopyrum (Polygonaceae) species based on morphology, isozymes and cpDNA variability. Genes Genet Syst. 1996;71:383-390. doi: 10.1266/ggs.71.383

2. Yasui Y, Ohnishi O. Phylogenetic relationships among *Fagopyrum* species revealed by the nucleotide sequences of the ITS region of the nuclear rRNA gene. Genes Genet Syst. 1998;73(4):201-210. doi: 10.1266/ggs.73.201

3. Eggum BO, Kreft I, Javornik B. Chemical composition and protein quality of buckwheat (*Fagopyrum esculentum* Moench). Plant Foods Hum Nutr. 1980;30(3):175-179. doi: 10.1007/BF1094020

4. Bonafaccia G, Marocchini M, Kreft I. Composition and technological properties of the flour and bran from common and tartary buckwheat. Food Chem. 2003;80(1):9-15. doi: 10.1016/S0308-8146(02)00228-5

5. Huang W, Jarvis DI, Ahmed S, Long C. Tartary buckwheat genetic diversity in the Himalayas associated with farmer landrace diversity and low dietary dependence. Sustainability. 2017;9(10):1806. doi: 10.3390/su9101806

6. Murai M, Ohnishi O. Population genetics of cultivated common buckwheat, Fagopyrum esculentum Moench. X. Diffusion routes revealed by RAPD markers. Genes Genet. Syst. 1996;71:211-218. Available at http://jstage.jst.go.jp/article/ggs/71/4/71_4_211/_pdf/-char/en. Accessed 18 September 2020

7. Ohnishi O. Search for the wild ancestor of buckwheat III. The wild ancestor of cultivated common buckwheat, and of tartary buckwheat. Econ Bot. 1998;52(2):123-133. Available at http://www.jstor.com/stable/4256049. Accessed 18 September 2020

8. Delêtrea M, McKey DB, Hodkinson TR. Marriage exchanges, seed exchanges, and the dynamics of manioc diversity. Proc Natl Acad Sci U S A. 2011;108(45):18249–18254. doi: 10.1073/pnas.1106259108

9. Hou S, Sun Z, Linghu B, Xu D, Wu B, Zhang B, et al. Genetic diversity of buckwheat cultivars (*Fagopyrum tataricum* Gaertn.) assessed with SSR markers developed from genome survey sequences. Plant Mol Biol Report. 2016;34(1):233–241. doi: 10.1007/s11105-015-0907-5

10. Kreft I, Fabjani N, Germ M. Rutin in buckwheat-protection of plants and its importance for the production of functional food. Fagopyrum. 2003;20:7-11. doi: 10.1.1.563.1011&rep=rep1&type=pdf

11. Bisht IS, Mehta PS, Bhandari DC. Traditional crop diversity and its conservation on-farm for sustainable agricultural production in Kumaon Himalaya of Uttaranchal state: A case study. Genet Resour Crop Evol. 2007;54:345–357. doi: 10.1007/s10722-005-5562-5

12. Liu M, Ma Z, Zheng T, Sun W, Zhang Y, Jin W, et al. Insights into the correlation

Formatted: Font color: Blue

Formatted: Font color: Auto

Formatted: Font color: Blue

Formatted: Font color: Auto

between physiological changes in and seed development of tartary buckwheat (*Fagopyrum tataricum* Gaertn.). BMC Genomics. 2018;19:648. doi: 10.1186/s12864-018-5036-8

13. Spengler R. (2020). Anthropogenic seed dispersal: Rethinking the origins of plant domestication. Trends Plant Sci. 2020;25(4):340-348. doi: 10.1016/j.tplants.2020.01.05

14. Bevan MW, Uauy C, Wulff BBH, Zhou J, Krasileva K, Clark MD. Genomic innovation for crop improvement. Nature. 2017;543:346-354. doi: 10.1038/nature22011

15. Li SQ, Zhang QH. Advances in the development of functional foods from buckwheat. Crit Rev Food Sci Nutr. 2001;41(6):451-464. doi: 10.1080/20014091091887

16. Jones MK, Liu XY. Origins of agriculture in East Asia. Science. 2009;324(5928):730-731. doi: 10.1126/science.1172082

17. Steadman KJ, Burgoon MS, Lewis BA, Edwardson SE, Obendorf RL. Minerals, phytic acid, tannin and rutin in buckwheat seed milling fractions. J Sci Food Agric. 2001;81(11):1094-1100. doi:10.1002/jsfa.914

18. Fabjan N, Rode J, Košir IJ, Wang Z, Zhang Z, Kreft I. Tartary buckwheat (*Fagopyrum tataricum* Gaertn.) as a source of dietary rutin and quercitrin. J Agric Food Chem. 2003;51(22):6452-6455. doi: 10.1021/jf034543e

19. Kreft I, Fabjan N, Yasumoto K. Rutin content in buckwheat (*Fagopyrum esculentum* Moench) food materials and products. Food Chem. 2006;98(3):508-512. doi: 10.1016/j.foodchem.2005.05.081

20. Gupta N, Sharma SK, Rana JC, Chauhan RS. Expression of flavonoid biosynthesis genes via-**à**-vis rutin content variation in different growth stages of *Fagopyrum* species. J Plant Physiol. 2011;168(17):2117-2123. doi: 10.1016/j.jplph.2011.06.018

21. Wang L, Yang X, Qin P, Shan F, Ren G. Flavonoid composition, antibacterial and antioxidant properties of tartary buckwheat bran extract. Ind Crops Prod. 2013;49:312-317. doi: 10.1016/j.indcrop.2013.04.039

22. Hu Y, Hou Z, Liu D, Yang X. Tartary buckwheat flavonoids protect hepatic cells against high glucose-induced oxidative stress and insulin resistance via MAPK signaling pathways. Food Funct. 2016;7(3):1523-1536. doi: 10.1039/c6fo1467k

23. Kim SJ, Sohn HB, Kim GH, Lee YY, Hong SY, Kim GD, et al. Comparison and validation of rutin and quercetin contents according to the extraction method of tartary buckwheat (*Fagopyrum tataricum* Gaertn.). Korean J Food Sci Technol. 2017;49(3):258-264. doi: 10.9721/KJFST.2017.49.3.258

24. Joshi BK. Correlation, regression and path coefficient analyses for some yield

Field Code Changed

Formatted: Font color: Auto

components in common and Tartary buckwheat in Nepal. Fagopyrum. 2005;22:77-82. doi: 10.1.1.524.5293&rep=rep1&type=pdf

25. Wang Y, Campbell C. Tartary buckwheat breeding (*Fagopyrum tataricum* Gaertn.) through hybridization with its rice-tartary type. Euphytica. 2007;156(3):399-405. doi: 10.1007/s10681-007-9839-3

26. Suzuki T, Morshita T, Mukasa Y, Takigawa S, Yokota S, Ishiguro K, Noda T. Breeding of 'Manten-Kirari', a non-bitter and trace-rutinosidase variety of Tartary buckwheat (*Fagopyrum tataricum* Gaertn.). Breed Sci. 2014;64(4):344-350. doi: 10.1270/jsbbs.64.344

27. Li C, Xie Z, Wang Y, Lu W, Yin G, Sun D, et al. Correlation and genetic analysis of seed shell thickness and yield factors in Tartary buckwheat (*Fagopyrum tataricum* (L.) Gaertn.). Breed Sci. 2019;69:464-470. doi:10.1270/jsbbs.18081

28. Singh SP. Broadening the genetic base of common bean cultivars. Crop Sci. 2001;41:1659-1675. doi: 10.2135/cropsci2001.1659

29. Hyten DL, Song Q, Choi IY, Nelson RL, Costa JM, Specht JE, et al. Impacts of genetic bottlenecks on soybean genome diversity. Proc Natl Acad Sci U S A. 2006;103(45):16666–16671. doi: 10.1073/pnas.0604379103

30. Bhandari HR, Bhanu AN, Srivastava K, Singh MN, Shreya, Hemantaranjan A. Assessment of genetic diversity in crop plants. Adv Plants Agric Res. 2017;7(3):279-286. doi: 10.15406/apar.2017.07.00255

31. Ericksen PJ. Conceptualizing food systems for global environmental change research. Glob Environ Change. 2008;18(1):234-245. doi: 10.1016/j.gloenvcha.2007.09.002

32. Hughes AR, Inouye BD, Underwood JN, Vellend M. Ecological consequences of genetic diversity. Ecol Lett. 2008;11(6):609-623. doi: 10.1111/j.1461-0248.01179.x

33. Britten RJ, Rowen L, Williams J, Cameron RA. Majority of divergence between closely related DNA samples is due to indels. Proc Natl Acad Sci U S A. 2003;100(8):4661-4665. doi: 10.1073/pnas.0330964100

34. Vali U, Brandsrom M, Johansson M, Ellegren H. Insertion-deletion polymorphisms (indels) as genetic markers in natural populations. BMC Genet. 2008;9:8. doi: 10.1186/1471-2156-9-8

35. Hou X, Li L, Peng Z, Wei B, Tang S, Ding M, et al. A platform of high-density INDEL/CAPS markers for map-based cloning in Arabidopsis. Plant J. 2010;63(5):880–888. doi: 10.1111/j.1365-313X.2010.04277.x

36. Moghaddam SM, Song Q, Mamidi S, Schmutz J, Lee R, Cregan P, et al. Developing market class specific InDel markers from next generation sequence data in *Phaseolus vulgaris* L. Front Plant Sci. 2014;5:185. doi: 10.3389/fpls.2014.00185

Formatted: Font color: Blue

Formatted: Font color: Auto

37. Meng HT, Zhang YD, Shen CM, Yuan GL, Yang CH, Jin R, et al. Genetic polymorphism analyses of 30 InDels in Chinese Xibe ethnic group and its population genetic differentiations with other groups. Scientific Reports. 2015;5:8260. doi: 10.1038/srep08260

38. Sohn HB, Kim SJ, Hwang TY, Park HM, Lee YY, Markkandan K, et al. Barcode system for genetic identification of soybean [*Glycine max* (L.) Merrill] cultivars using InDel markers specific to dense variation blocks. Front Plant Sci. 2017;8:520. doi: 10.3389/fpls.2017.00520

39. Sohn HB, Song YH, Kim SJ, Hong SY, Kim KD, Koo BC, Kim YH. Identification and chromosomal reshuffling patterns of soybean cultivars bred in Gwangwon-do using 202 InDel markers specific to variation blocks. Korean J Breed Sci. 2018;50(4):396-405. doi: 10.9787/KJBS.2018.50.4.396

40. Chun JB, Jin M, Jeong N, Cho C, Seo MS, Choi MS, et al. Genetic identification and phylogenetic analysis of new varieties and 149 Korean cultivars using 27 InDel markers selected from dense variation blocks in soybean (*Glycine max* (L.) Merrill). Korean J Plant Res. 2019;32(5):519-542. doi: 10.7732/kjpr.2019.32.5.519

41. Tsuji K, Ohnishi O. Phylogenetic relationships among wild and cultivated tartary buckwheat (*Fagopyrum tataricum* Gaertn.) populations revealed by AFLP analyses. Genes Genet Syst. 2001;76(1):47–52. doi: 10.1266/ggs.76.47

42. Tsuji K, Ohnishi O. Origin of cultivated Tatary buckwheat (*Fagopyrum tataricum* Gaertn.) revealed by RAPD analyses. Genet Resour Crop Evol. 2000;47:431–438. doi: 10.1023/A:1008741513277

43. Tsuji K, Ohnishi O. Phylogenetic position of east Tibetan natural populations in tartary buckwheat (*Fagopyrum tataricum* Gaertn.) revealed by RAPD analyses. Genet Resour Crop Evol. 2001;48:63–67. doi: 10.1023/A:1011286326401

44. Zhang A, Ohnishi O. Molecular characterization of genetic diversity of underutilized crops; Buckwheat as an example. Acta Hortic. 2013;979:407-419. doi: 10.17660/ActaHortic.2013.979.44

45. Katsu K, Suzuki T, Fujino K, Morishita T, Noda T. Development of a DNA marker for variety discrimination specific to 'Manten-Kirari' based on the NGS-RNA sequence in Tartary buckwheat (*Fagopyrum tataricum*). Food Chem. 2019;295:51-57. doi: 10.1016/j.foodchem.2019.05.087

46. Zhang L, Ma M, Liu L. Identification of genetic locus underlying easy dehulling in rice-tartary for easy postharvest processing of tartary buckwheat. Genes. 2020;11(4):459. doi: 10.3390/genes11040459

47. Yasui Y, Hirakawa H, Ueno M, Matsui K, Tanaka TK, Yang SJ, et al. Assembly of the draft genome of buckwheat and its applications in identifying agronomically useful genes. DNA Res. 2016;23(3):215-224. doi: 10.1093/dnares/dsw012

48. Zhang L, Li X, Ma B, Gao Q, Du H, Han Y, et al. The Tartary buckwheat genome provides insights into rutin biosynthesis and abiotic stress tolerance. Mol Plant. 2017;10(9):1224-1237. doi: 10.1016/j.molp.2017.08.013

49. Bansal V, Boucher C. Sequencing technologies and analyses: Where have we been and where are we going? iScience. 2019;18:37-41. doi: 10.1016/j.isci.2019.06.035

50. Janwan M, Sreewongchai T, Sripichitt P. Rice breeding for high yield by advanced single seed descent method of selection. J Plant Sci. 2013;8(1):24-30. doi: 10.3923/jps.2013.24.30

51. Halbrecq B, Romedenne P, Ledent JF. Evolution of flowering, ripening and seed set in buckwheat (*Fagopyrum esculentum* Moench): quantitative analysis. Europ J Agronomy. 2005;23:209-224. doi: 10.1016/j.eja.2004.11.006

52. Reyes-Valdés MH, Santacruz-Varela A, Martinez O, Simpson J, Hayano-Kanashiro, C, Cortés-Romero C. Analysis and optimization of bulk DNA sampling with binary scoring for germplasm characterization. PLoS ONE. 2013;8:e79936. doi:10.1371/journal.pone.0079936

53. Rogers SO, Bendich AJ. Extraction of total cellular DNA from plants, algae and fungi. In: Gelvin SB, Schilperoort RA, editors. Plant Molecular Biology Manual. Springer: Dordrecht; 1994. p. 183-190. doi: 10.1007/978-94-011-0511-8_12

54. Chin CS, Peluso P, Sedlazeck FJ, Nattestad M, Concepcion GT, Clum A, et al. Phased diploid genome assembly with single-molecule real-time sequencing. Nat Methods. 2016;13:1050-1054. doi: 10.1038/nmeth.4035

55. Luo R, Liu B, Xie Y, Li Z, Huang W, Yuan J, et al. SOAPdenovo2; an empirically improved memory-efficient short- read *de novo* assembler. Gigascience. 2012;1(1):18. doi: 10.1186/s13742-015-0069-2

56. Huang S, Kang, M, Xu A. Haplomergers: rebuilding both haploid subassemblies from high-heterozygosity diploid genome assembly. Bioinformatics. 2017;33(16):2577-2579. doi: 10.1093/bioinformatics/btx220

57. Boetzer M, Henkel CV, Jansen HJ, Butler D, Pirovano W. Scaffolding pre-assembled contigs using SSPACE. Bioinformatics. 2011;27(4):578-579. doi: 10.1093/bioinformatics/btq683

58. Li H, Durbin R. Fast and accurate short read alignment with Burrows-Wheeler transform. Bioinformatics. 2009;25(14):1754-1760. doi: 10.1093/bioinformatics/btp324

59. McKenna A, Hanna M, Banks E, Sivachenko A, Cibulskis K, Kernytsky A, et al. The genome analysis toolkit: a MapReduce framework for analyzing next-generation DNA sequencing data. Genome Res. 2010;20(9):1297-1303. doi: 10.1101/gr.107524.110

Formatted: Font color: Blue

Formatted: Font color: Auto

Formatted: Font color: Blue

Formatted: Font color: Auto

Formatted: Font color: Blue

Formatted: Font color: Auto

Formatted: Font color: Blue

Formatted: Font color: Auto

60. Anderson JA, Churchill GA, Autrigue JE, Tanksley SD, Sorrells ME. Optimizing parental selection for genetic linkage maps. Genome. 1993;36(1):181–186. doi: 10.1139/g93-024

61. Liu K, Muse S. Powermarker: an intergrated analysis environment for genetic marker analysis. Bioinformatics. 2005;21(9):2128-2129. doi: 10.1093/bioinformatics/bti282

62. Perrier X, Jacquemoud-Collet JP. DARwin Software. 2006. Available at http://darwin.cirad.fr/

63. Prichard JK, Stephens M, Rosenberg NA, Donnelly P. Association mapping in structured populations. Am J Hum Genet. 2000;67(1):170-181. doi: 10.1086/302959

64. Kim YH, Park HM, Hwang TY, Lee SK, Choi MS, Jho S, et al. Variation block-based genomics method for crop plants. BMC Genomics 2014;15:477. doi: 10.1186/1471-2164-15-477

65. Jiang P, Burczynski F, Campbell C, Pierce G, Austria J A, Briggs CJ. Rutin and flavonoid contents in three buckwheat species *Fagopyrum esculentum*, *F. tataricum*, and *F. homotropicum* and their protective effects against peroxidation. Food Res Int. 2006;40(3):356-364. doi: 10.1016/j.foodres.200610.009

66. Kreft S, Strukelj B, Gaberscik A, Kreft I. Rutin in buckwheat herbs grown at different UV-B radiation levels: comparison of two UV spectrophotometric and an HPLC method. J Exp Bot. 2002;53(375):1801-1804. doi: 10.1093/jxb/erf032

67. Matsuura H, Inanaga S, Murata K. Differences in the vegetative growth between common and tartary buckwheat in saline hydroponic culture. Plant Prod Sci. 2005;8(5):533–538. doi: 10.1626/pps.8.533

68. Owens DK, Alerding AB, Crosby KC, Bandara AB, Westwood JH, Winkel BSJ. Functional analysis of a predicted flavonol synthase gene family in Arabidopsis. Plant Physiol. 2008;147:1046-1061. doi: 10.1104/pp.108.117457

69. Germ M, Brenznik B, Dolinar N, Kreft I, Gaberščik A. The combined effect of water limitation and UV-B radiation on common and tartary buckwheat. Cereal Res. Commun. 2013;41:97-105. doi: 10.1556/CRC.2012.031

70. Pandey V, Niranjan A, Atri N, Chandrashekhar K, Misha MK, Trivedi PK, Misra P. WsSGTL1 gene from *Withania somnifera*, modulates glycosylation profile, antioxidant system and confers biotic and salt stress tolerance in transgenic tobacco. Planta. 2014;239(6):1217-1231. doi: 10.1007/s00425-014-2046-x

71. Jackson L, van Noordwijk M, Bentsson J, Foster W, Lipper L, Pulleman M, et al. Biodiversity and agricultural sustainability: from assessment to adaptive management. Curr Opin Environ Sustain. 2010;2:80-87. doi: 10.1016/j.cosust.2010.02.007

Formatted: No underline, Font color: Blue

Formatted: Font color: Blue

Formatted: Font color: Auto

72. Vigouroux Y, Barnaud A, Scarcelli N, Thuillet AC. Biodiversity, evolution and adaptation in cultivated crops. C R Biol. 2011;334:450–457. doi: 10.1016/j.crvi.2011.03.003

73. Ahmed S, Peters CM, Long CL, Meyer R, Unachukwu U, Litt A, et al. Biodiversity and phytochemical quality in indigenous and state-supported tea management systems of Yunnan, China. Conserv Lett. 2013;6:28–36. doi: 10.1111.j.1755-263x.2012.00269.x

74. Qu Y, Zhou Y, Wang Z, Wang Z, Wang P, Gao J, et al. Analysis of genetic diversity and structure of Tartary buckwheat resources from production regions. Scientia Agricultura Sinica. 2016;49(11):2049-2062. doi: 10.3864/j.issn.0578-1752.2016.11.002

75. Tian D, Wang Q, Zhang P, Araki H, Yang S, Kreitman M, et al. Single-nucleotide mutation rate increases close to insertion/deletions in eukaryotes. Nature. 2008;455(7209):105-108. doi: 10.1038/nature07175

76. van der Biezen EA, Freddie CT, Kahn K, Parker JE, Jones JDG. Arabidopsis RPP4 is a member of the RPP5 multigene family of TIR-NB-LRR genes and confers downy mildew resistance through multiple signaling components. Plant J. 2002;29(4):439-451. doi; 10.1046/j.0960-7412.2001.01229.x

77. Langlois-Meurinne M, Gachon CM, Saindrenan P. Pathogen-responsive expression of glycosyltransferase genes UGT73B3 and UGT73B5 is necessary for resistance to *Psudomonas syringae* pv tomato in Arabidopsis. Plant Physiol. 2005;139(4):1890-1901. doi: 10.1104/pp.105.067223

78. Attaran E, Rostás M, Zeier J. *Pseudomonas syringae* elicits emission of the terpenoid (E,E)-4,8,12-trimethnyl-1,3,7,11-tridecatetraene in Arabidopsis leaves via jasmonate signaling and expression of the terpene synthase TPS4. Mol Plant Microbe Interact. 2008;21(11):1482-1497. doi: 10.1094/MPMI-21-11-1482

79. Islam MS, Wang MH. Expression of dehydration responsive element-binding protein-3 (DREB3) under different abiotic stresses in tomato. BMB Rep. 2009;42(9):611-616. doi: 10.5483/bmbrep.2009.42.9.611

80. Bellucci E, Bitocchi E, Rau D, Nanni L, Ferradini N, Giardini A, et al. Population structure of barley landrace populations and gene-flow with modern varieties. PLoS One. 2013;8:e83891. doi: 10.1371/journal.pone.0083891

81. Hollister JD, Ross-Ibara J, Gaut BS. Indel-associated mutation rate varies with mating system in flowering plant. Mol Biol Evol. 2010;27(2):409-416. doi: 10.1093/molbev/msp249

82. Leishman MR, Westoby M, Jurado E. Correlates of seed size variation: a comparison among five temperate floras. J Ecol. 1995;83(3):517-530. doi: 10.2307/2261604

Supporting information

S1 Fig. The draft genome assembly process using *Fagopyrum tataricum* cv. “"Daegwan 3-7.”'.

S2 Fig. Gel images using 50 InDel polymorphisms for 73 tartary buckwheat accessions.

Formatted: Font color: Red

S23 Fig. Application of barcode system to tartary buckwheat accession, “"HLB1003.”'.

S1 Table. List of 73 tartary buckwheat accessions used for evaluating the selected InDels.

S2 Table. A gradient UPLC method for the simultaneous determination of rutin and quercetin.

S3 Table. Statistics of filtered raw data for evaluating Illumina paired read quality in 26 tartary buckwheat accessions.

S4 Table. Number of InDels showing alternative genotype in 26 re-sequenced genomes.

S5 Table. The 50 InDel markers developed to discriminate tartary buckwheat accessions.

S6 Table. A diagram of PCR band patterns of 73 tartary buckwheat accessions obtained using 50 InDel markers.

S7 Table. Statistics of the 50 InDels in tartary buckwheat accessions (n = 73).

Dear Reviewers,

We greatly appreciate the reviewer's efforts to carefully review our manuscript and the valuable suggestions offered, which were invaluable in helping us to make improvements. We have revised and modified our manuscript to clarify and strengthen its content, after taking into consideration all of the reviewers' comments. Please find our responses to each of the reviewers' comments below.

Responses to the reviewer's comments

Development of 50 InDel-based barcode system for genetic identification of tartary buckwheat resources

Submitted: 28 Sep. 2020
Revised: 20 Dec. 2020

Reviewer # 1

1. **The language of this manuscript should be improved by a native English speaker, or a professional language editing service. There are a lot of grammar errors in this manuscript. There are a lot of grammar errors in this manuscript, such as Lines 94-97.**

Thank you and we are pleased to inform you that this manuscript has been improved by a professional language editing service. We have now provided this information in acknowledgements, as below.

Line 521 to 522:
The authors would like thank Enago (www.enago.co.kr) for the English language review.

2. **The 50 InDel-based barcode system has a bias for genetic identification of tartary buckwheat resources. The amount of 50 InDel-based genotypes of 73 accessions is not enough.**

We agree with the reviewer that allelic information derived from high-density markers can reduce a bias for genetic studies of tartary buckwheat accessions. To define the haplotype polymorphisms with high accuracy, the length of the haplotype blocks should be smaller than the length of the LD blocks. Several measurements of LD have been reported for major crop species, e.g., rapeseed (465.5 kb) [1], foxtail millet (*Setaria italic*, 100kb) [2], pigeon pea (*Cajanus cajan*, 70 kb) [3], soybean (150 kb) [4], and rice (200 kb) [5]. In tartary buckwheat, the short breeding history and low selection intensity could result in rapid LD decay compared to other major crop species. For our research, we identified 171,926 homogenous InDel markers from 26 resources and validated them as reliable, transferable and cost-effective markers in 73 accessions.

We questioned how many markers would be appropriate to develop a barcode system for tartary buckwheat identification. Along with the cost of genotyping, user-friendly technology became the key factor to determine the intensity of marker application. Recently, we developed soybean genetic studies using InDels, which were good sources of reference. After an extensive survey of 73,327 InDels by Kim et al. [6], other studies aimed to adopt InDels as tool in soybean genetic studies [7-9]. For this purpose, Sohn et al. [7] showed 202 unique InDel polymorphisms in 147 soybean cultivars, with an average density of one InDel per 5.5 Mb. Recent follow-up studies have reported that genetic diversity studies of Korean soybean cultivars are possible along with the soybean identification using the 27 markers selected from the 202 InDels [8-9]. Although comparisons must be regarded with care due to the different numbers of markers and accessions and the selection intensity, the genetic studies of tartary buckwheat accessions could be performed using the 50 InDels selected from 26 tartary buckwheat whole-genomes.

In the present work, we evaluated the usefulness of the 50-InDel-based barcode system applied to tartary buckwheat classification. Each of the following sections were developed: 1. Putative InDel markers using next generation sequencing; 2. Selection of 50 InDels and bar-coding process; 3. Evaluation of the selected InDels for genetic identification; 4. Constructing a database using expanded InDels in 73 tartary buckwheat accessions.

Phylogenetic analysis of 6,622 non-synonymous SNP variants and population structure subdivided the panel into 2 primary populations (Fig 7 in the manuscript): a reference group and a non-reference group. A similar result was found with the 50 InDels, which clustered the tartary buckwheat into 2 separate groups [9]. Together with the evidence relating the 6,622 SNPs and the 50 InDels to environmental adaptability, these results are consistent with the segregation of the tartary buckwheat into different groups according to their geographical distribution. Particularly because the barcode system could easily identify tartary buckwheat accessions using PCR and gel electrophoresis compared to other molecular markers, such as SSR and SNP. This approach is efficient, rapid and low-cost for the genetic identification of tartary buckwheat accessions in common laboratories.

However, some accessions in subgroup 2 had identical genotypes using 50 InDels (Fig 1). They showed obvious differences in seed shape and seed volume (Fig 1), suggesting that InDel density has not been as well characterized in natural populations as genetic diversity. We tested

additional 86 primer sets in which there was no marker to distinguish the resources with identical genotypes using 50 InDels. The results indicate that some accessions could be genetically quite similar.

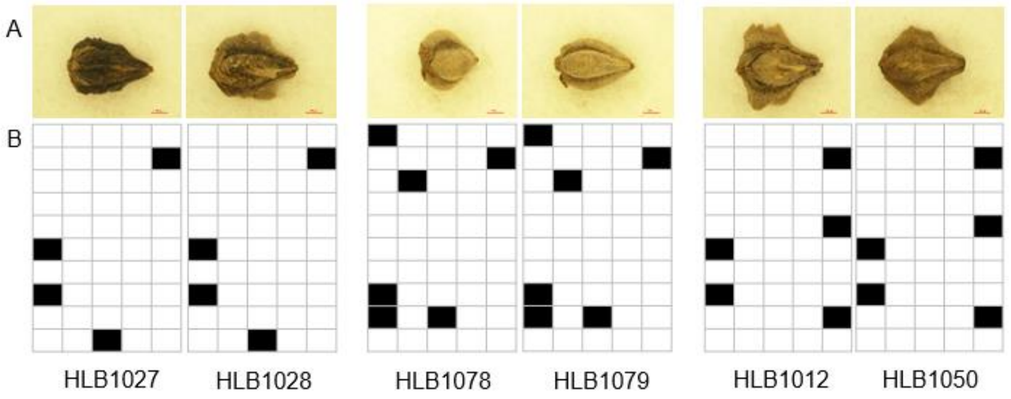

Fig 1. Seed shapes (A) and barcodes (B) of accessions with the same genotype.

In order to distinguish the identical genotypes, we have been approaching two strategies. First, we have been developing a user-friendly program using a database comprising genotypes based on 50 InDels, seed shapes, and functional substances. The 50 InDels could be used as a complement for classification by seed shapes in resource management (Fig 2). Specifically we are trying to develop additional markers to establish PCR-based markers applicable for all tartary buckwheat accessions. We are currently improving the draft genome of *F. tataricum* cv. “Daegwan 3-7” to reduce the number of annotated genes and duplicated BUSCO. In the future, we will present Fta Daegwan 3-7 gene models and each of the following sections: 1. High-quality draft genome of *F. tataricum*; 2. Comparison to other Carophyllales genomes; 3. Comparison with the draft genome of tartary buckwheat cv. “Pinku1”; 4. Fta pan-genome through SoapDenovo2 assembly of the 26 accessions. These results will lead to the discovery of a larger number of genome-wide markers which will help researchers to genetically characterize the tartary buckwheat genome at high resolution.

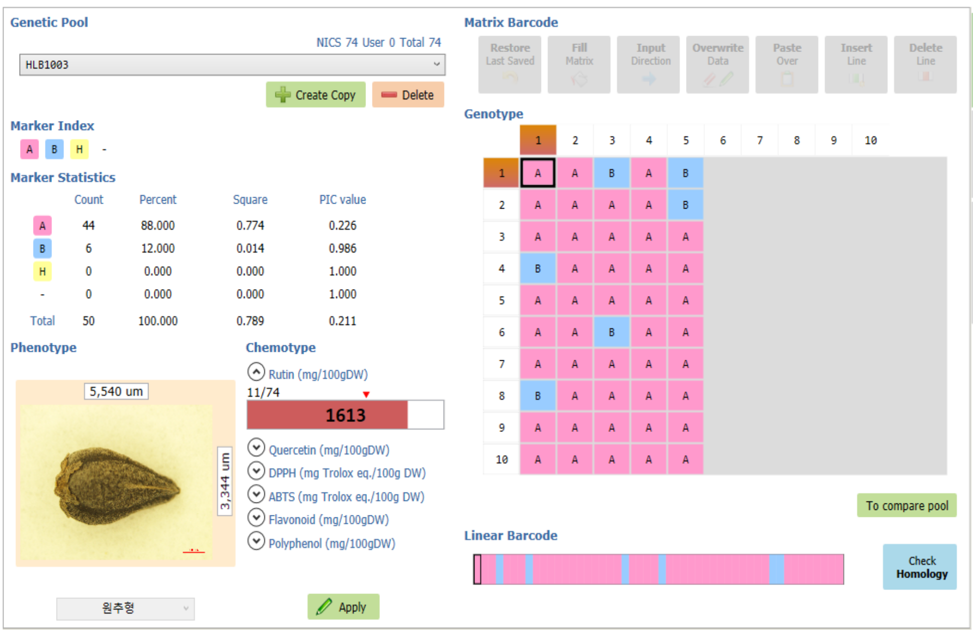

Fig 2. The database compiling genotype, seed shape, and functional substances.

References

1. Wu D, et al. Whole-genome resequencing of a worldwide collection of rapeseed accessions reveals the genetic basis of ecotype divergence. Molecular Plant. 2019: 12: 30-43.
2. Jia J, et al. A haplotype map of genomic variations and genome-wide association studies of agronomic traits in foxtail millet (*Setaria italic*).Nature genetics. 2013:49:957-961.
3. Varshney RK, et al. Whole-genome resequencing of 292 pigeonpea accessions identifies genomic regions associated with domestication and genomic traits. Nature Genetics. 2017:49:1082.
4. Zhou Z, et al. Resequencing 302 wild and cultivated accessions identifies genes related to domestication and improvement in soybean. Nature biotechnology. 2015:33:408-414.
5. Mather KA, et al. The extent of linkage disquilibrium in rice (*Oryza sativa* L.). Genetics. 2007:177:2223-2232.
6. Kim YH, et al. Variation block-based genomics method for crop plants. BMC Genomics. 2014:15:477.
7. Sohn HB, et al. Barcode system for genetic identification of soybean [*Glycine max* (L.) Merrill] cultivars using InDel markers specific to dense variation blocks. Front Plant Sci. 2017;8: 520.
8. Sohn HB, et al. Identification and chromosomal reshuffling patterns of soybean cultivars bred in Gwangwon-do using 202 InDel markers specific to variation blocks. Korean J Breed Sci. 2018;50(4): 396-405.
9. Chun JB, et al. Genetic identification and phylogenetic analysis of new varieties and 149 Korean cultivars using 27 InDel markers selected from dense variation blocks in soybean (*Glycine max* (L.) Merrill). Korean J Plant Res. 2019;32(5): 519-542.

Reviewer # 2

1. **Abstract has been casually written in terms of language used, especially lines 41-45. Please modify accordingly.**

Your assessment has been of great help to us. We have edited the abstract, as below.

Line 27 to 48:
Tartary buckwheat (*Fagopyrum tataricum* Gartn.) is a highly functional crop that is poised to be the target of many future breeding efforts. The reliable *ex situ* conservation of various genetic resources is essential for the modern breeding of tartary buckwheat varieties. We developed PCR-based and co-dominant insertion/deletion (InDel) markers to discriminate tartary buckwheat genetic resources. First, we obtained the whole genome from 26 accessions across a superscaffold-scale reference genome of 569.37 Mb for tartary buckwheat cv. "Daegwan 3-7." Next, 171,926 homogeneous and 53,755 heterogeneous InDels were detected by comparing 26 accessions with the "Daegwan 3-7" reference sequence. Of these, 100 candidate InDels ranging from 5–20 bp in length were chosen for validation, and 50 of them revealed polymorphisms between the 26 accessions and "Daegwan 3-7." The validated InDels were further tested through the assessment of their likelihood to give rise to a single or a few PCR products in 50 other accessions, covering most tartary buckwheat genome types. The major allele frequencies ranged from 0.5616 at the TB42 locus to 0.9863 at the TB48 locus, with the average PIC value of 0.1532 with a range of 0.0267–0.3712. To create a user-friendly system, the homology of the genotypes between and among the accessions were visualized in both one- (1D) and two-dimensional (2D) barcode types by comparing amplicon polymorphisms with the reference variety, "Daegwan 3-7." A phylogenetic tree and population structure of the 76 accessions according to amplicon polymorphisms for the 50 InDel markers corresponded to those using non-synonymous single nucleotide polymorphism variants, indicating that the barcode system based on the 50 InDels was a useful tool to improve the reliability of identification of tartary buckwheat accessions in the germplasm stocks.

2. **What age plant material was used for measuring the rutin and quercetin contents? Mature…how many years?**

We have now provided this information, as below.

Line 135 to 140:
To investigate the biodiversity and comparative relationships between the accessions, collected accessions were sown in 2017 and grown in the greenhouse at the Highland Agriculture Research Institute. We separated the plants by developmental stage: the vegetative growth stage (1–40 days after planting, DAP), flowering stage (40–80 DAP), and yield stage (80-90 DAP). At the yield stage, the color of the seed coat turned black, indicating that the seeds were completely maturated.

Line 142 to 144:
The yield stage seeds were used to measure their rutin and quercetin content. First, the mature seeds from the 76 accessions were milled into a fine powder, then the samples were immediately flash-frozen in liquid nitrogen and stored at −80°C until further use.

3. **What do you mean by 'etc.' in Fig. 2? I am sorry but you can't use such terms especially describing a figure. Correct it in figure legend as well as in the figure itself. You can add country names as and what available.**

We thank for the reviewer comment. We revised "etc." in Fig. 2 to USA/Japan/Russia.

4. **In Fig.3, replace the word 'InDel' by 'InDels' as you dealing with a large no.**

According to the reviewer's suggestion, InDel in Figure 3 was modified to InDels.

5. **References need thorough rechecking as per journal standards. Maintain uniformity while citing edited books, chapters, chapters and/or proceedings.**

Thank you for calling this to our attention. The Reference section of this manuscript has now been edited to match the journal's requirements.

6. **Lastly, recently the grammatical mistakes and sentence framing. Authors seemed to be in a hurry while compiling this analysis. It will be better if help of some native English speaker could be taken.**

Thank you for calling this to our attention. Everything you have pointed out has been corrected in the main text. In order to reduce mistakes in grammar/typos, all authors have tried to proofread the manuscript. In addition, this manuscript has been improved by a professional language editing service. We have now provided this information in acknowledgements, as below.

Line 521 to 522:
The authors would like thank Enago (www.enago.co.kr) for the English language review.

Reviewer # 3

1. **The authors write that they have represented a high quality draft genome assembly and annotation for tartary buckwheat. They do not provide the needed analysis results to backup this assertion, as they have not compared their assembly with previously available assemblies.**

To obtain a high-quality draft genome assembly, we produced a total of 43.83 and 32.17 Gb sequences from Illumina paired-ends (PE) and Single-Molecule Real-Time (SMRT) platforms, respectively, which corresponded to 70x (Illumina PE) and 52x (SMART) coverages. A hybrid assembly followed by scaffolding, gap-filling, and cleaning of redundancy, which resulted in a final draft assembly of 569.37 Mb in 2,886 scaffolds with 50% of the total sequence captured in 156 scaffolds larger than 886,968 bps (N50). We predicted a total of 43,771 putative protein-coding gene models occupying 19.33% of the genome, while 52.00% consisted of repetitive sequences and transposable elements, with Gypsy family long terminal repeat (LTR) retrotransposons being the most abundant class.

The problem with *F. tataricum* cv. “Daegwan 3-7” was that the number of annotated genes and duplicated BUSCO was high compared to the draft genome of *F. tataricum* cv. “Pinku 1.” Thus, we are currently working with Professor Dong-Ha Oh of Louisiana State University to improve the draft genome of *F. tataricum* cv. “Daegwan 3-7.” The table below shows the progress to date.

Table. The number of annotated genes and duplicated BUSCO to improve the draft genome of *Fagopyrum tataricum* cv. “Daegwan 3-7.”

Genome annotation	# representative gene models	% out of total 1,375 BUSCOs[1]			Note
		Complete (Single, Duplicate)	Fragmented	Missing	
Ath	27,445	99.9 (99.1, 0.8)	0.0	0.1	*Arabidopsis thaliana* (Araport11)
Vvi	26,346	95.2 (93.7, 1.5)	3.3	1.5	*Vitis vinifera* (Phytozome version 315)
Sly	34,727	97.6 (97.2, 0.4)	2.0	0.4	*Solanum lycopersicum* (Phytozome v. 390)
Ahy	23,033	92.9 (90.6, 2.3)	5.2	1.9	*Amaranthus hypochondriacus* (Phytozome v. 315)
Bvu	27,421	88.8 (86.8, 2.0)	6.7	4.5	*Beta vulgaris* (v. 1.2)
Cqu	44,776	95.4 (26.3, 69.1)	2.6	2.0	*Chenopodium quinoa* (Phytozome v. 392)
Fta PK	30,386	**89.4** (85.1, 4.3)	5.3	5.3	*Fagopyrum tartaricum* cv. Pinku1 (Zhang et al., 2017)
Fta DG 54 (v5.4)	43,771	80.0 (69.4, 10.6)	11.3	8.7	Fta DG 53 + redundancy filter (used in the 1st submission)
Fta DG 54.ac.s1	293,211	92.0 (17.0, 75.0)	4.7	3.3	Fta DG 54 + new annotation (redundancy and TE removed)
Fta DG 54.ac.s4a	122,527	89.8 (76.9, 12.9)	6.1	4.1	Fta DG 54 + new anno. (consolidated)
Fta DG 54.ac.s4a.TE3.s3	54,794	89.8 (77.2, 12.6)	6.1	4.1	Fta DG 54 + new anno. (LS TE removed) Ks=0 syntelogs > 550
Fta DG 55.a1	53,648	89.6 (78.6, 11.0)	6.3	4.1	Fta DG 54 + redundancy filter (1st round) Ks=0 syntelogs < 450
Fta DG 55.a3 (**v5.5a3**)	53,091	89.6 (79.3, 10.3)	6.3	4.1	Fta DG 54 + redundancy filter (3rd round) **Ks=0 syntelogs < 70**
Fta DG 55.b					Fta DG 54 + redundancy filter (4th round) **Ks=0 syntelogs < 40**
Fta DG 55.b1	44,200	89.2 (79.1, 10.1)	6.3	4.5	Fta DG 55.b + TEs further removed using RepeatModeler
Fta DG 55.b1b (**v5.5b1b**)	**36,675**	**89.2** (79.1, 10.1)	6.3	4.5	Fta DG 55.b1 + OrthFinder singlets with no expression removed
Fes R1 Yasui	286,768	76.8 (71.9, 4.9)	12.6	10.6	*F. esculentum* (Yasui et al., 2016)
Fes R1 TeraBIO	62,831	81.6 (76.3, 5.3)	9.5	8.9	*Fagopyrum esculentum* (used in the 1st submission)

[1] All BUSCO analyses were performed on representative deduced protein sequences using the “embryophyte odb10” database.

In the future, we will present *F. tataricum* “DG” gene model and each of the following sections: 1. High-quality draft genome of *F. tataricum*; 2. Comparison to other Carophyllales genomes; 3. Comparison with the draft genome of tartary buckwheat cv. “Pinku1”; 4. *F. tataricum* pan-genome through SoapDenovo2 assembly of the 26 accessions. Genome sequences, as well as orthologous genes and gene families identified as uniquely expanded in tartary buckwheat through comparative analysis, could provide an essential resource for studying the adaptation, specialized metabolism, and the genomic basis of the strikingly high rutin content in this interesting crop species.

2. **The material and methods section of the manuscript is not precise enough and does not contain all the processing steps presented in the result section. Such as the conditions for structure analysis should be detailed, with parameter settings.**

The material and methods section has now been modified per the reviewer’s suggestion. The blue marked portion has been added.

Line 124 to 241:
Plant Materials

The accessions of 73 tartary buckwheat plants (S1 Table) were provided by the National Agrobiodiversity Center (Jeonju, Republic of Korea) and the Highland Agriculture Research Institute (Pyeongchang, Republic of Korea). The accessions were planted in a greenhouse located at the Highland Agriculture Research Institute (37°68′N and 128°73′E, 779 m altitude), Gangwon Province, Republic of Korea. These were self-pollinated 3 times by the single seed descent (SSD) method by growing the individual plants in flowerpots (10cm in diameter) [50]. We grew an additional four generations of HLB1001 using the SSD method, which resulted in the selection of a progeny, “Daegwan 3-7” for a draft genome of tartary buckwheat. A total of 26 accessions were collected (China, India, Nepal, Bhutan, Pakistan, USA).
To investigate the biodiversity and comparative relationships between the accessions, collected accessions were sown in 2017 and grown in the greenhouse at the Highland Agriculture Research Institute. We separated the plants by developmental stage: the vegetative growth stage (1–40 days after planting, DAP), flowering stage (40–80 DAP), and yield stage

(80-90 DAP). At the yield stage, the color of the seed coat turned black, indicating that the seeds were completely maturated.

Measurement of rutin and quercetin content

The yield stage seeds were used to measure their rutin and quercetin content. First, the mature seeds from the 76 accessions were milled into a fine powder, then the samples were immediately flash-frozen in liquid nitrogen and stored at −80°C until further use. A total of 0.10 g dry powder for each sample was mixed with 1 ml methanol, then was extracted at 80°C for 1 hr in a conventional Soxhlet apparatus. The extract was filtered through a 0.20 μm syringe filter (PTFE 13 mm, PALL Life Sciences, Ann Arbor, MI) for rutin content [23]. Ultra-performance liquid chromatography (Waters Corporation, Milford, MA, USA) was performed on an Acquity 1-class using a C18 column (2.1 mm × 100 mm, 1.7 μm) (Waters Corporation, USA) at 30°C. The mobile phase (1% formic acid in water/0.1% formic acid in acetonitrile) flowed by the gradient elution method (S2 Table) at a flow rate of 0.25 ml/min with a total injection volume of 10 $\mu\ell$. Rutin quantity was estimated based on the linear calibration curve of standard rutin and quercetin (Extrasynthese, France) under a detection wavelength of 259 nm. Three independent sample analyses were performed for each sample. All statistical analyses were performed using ver. 3.6.1 of the R software.

DNA sequencing and *de novo* assembly

After 21 DAP, the leaves in the third node of the progeny "Daegwan 3-7" were used to create a draft genome. First, 3-week-old leaves from the bulk of 10 plants were collected for genomic DNA extraction using a standard CTAB (cetyl trimethylammonium bromide) protocol [52, 53]. Both short read (Illumina) and long read (PacBio) libraries were prepared according to the manufacturer's instructions for the entire genome of *F. tataricum*. A short insert paired-end (PE) library was prepared using a Illumina TruSeq Nano DNA Sample Preparation Kit (Illumina Inc., San Diego, Ca, USA) and 1~3 μg of DNA. A library profile analysis was performed with an Agilent 2100 Bioanalyzer (Agilent Technologies, USA) and qPCR quantification, then the libraries were sequenced to 2 × 100 bp on the Illumina HiSeq 2500 platform (Illumina, USA) from the TheragenEtex Bio Institute (TheragenEtex Inc., Suwon, Republic of Korea). PacBio SMRTbell libraries (2 kb, 5 kb, 10 kb, and 15 kb inserts) were prepared with the standard PacBio library preparation protocols, which are available at http://pacificbiosciences.com/. The sequencing was conducted on a PacBio RS II (Pacific Biosciences, USA) system using C4 chemistry and 240 min movies by the TheragenEtex Bio Institute.

The short and long reads were assembled separately. The de novo assembly of the PacBio long reads was performed using Fast Alignment and CONsensus [54] with default parameters. The short reads were assembled with SOAPdenovo2 [55] with default parameters. The initial contigs were merged using HaploMerger2 [56]. Both short and long reads were then used to construct scaffolds with SSPACE [57], which led to the superscafold-scale draft genome of tartary buckwheat cv. "Daegwan 3-7." The N50 length of the final genome assemblies was evaluated.

Identification and validation of the polymorphic InDels

Genomic DNA was extracted for whole-genome resequencing from the 26 accessions that had self-pollinated 3 times using the SSD method. Three-week-old leaves from the bulk of 10 plants were collected for genomic DNA extraction using a standard CTAB protocol [52, 53]. The data consisted of 101-bp reads, which were generated using the HiSeq 2500 sequencer. The Short Oligonucleotide Alignment Program 2 (SOAP2) was used to map the raw pair-end reads onto the reference genome (tartary buckwheat cv. "Daegwan 3-7"). For each accession, more than 93% of the reads were properly aligned to the reference genome. Insert size was estimated by mapping the reads to the reference genome using the Burrows-Wheeler Aligner algorithm (bwa) [58] ver 0.5.9. The aligned reads were realigned at InDel positions using the GATK InDelRealinger algorithm [59] enhance the mapping quality. The base quality scores were recalibrated using the GATK TableRecalibration algorithm. The primer pair used to amplify each of the InDels were selected by the Primer3 software (http://primer3.sourceforge.net). We selected InDels with more than 10 bp and designed the primers accordingly to match the characteristics of each InDel using the Primer 3 software.

Amplicon polymorphism assay and bar-coding process

PCR analysis was performed using 10 $\mu\ell$ reaction mixtures containing 20 ng of total genomic DNA, 2 pM of primer, and 5 $\mu\ell$ of GoTaq Green Master Mix (Promega, Madison, WI, USA). The PCR was performed at 95℃ for 5 min, followed by 35 subsequent rounds at 94℃ for 30 sec, 45°C for 30 sec and 72°C for 30 sec, using a Mastercycler pro 384 (Eppendorf, Germany). The PCR products were separated by electrophoresis in 3% gels of certified low range ultra-agarose (Bio-rad) followed by LoadingSTAR staining (Dynebio, Republic of Korea). For the limitation of the number of candidates, we chose primer pairs that amplified PCR products that were 150–231 bp long. The allelic diversity of the InDels with the e-PCR products in 26 tartary buckwheat genomes were assessed via PIC (Polymorphism Information Content), which was defined as PIC i =1-$\sum_{j=1}^{n} p_{ij}^2$, where p_{ij} is the frequency of the jth pattern for the ith marker [60,61].

The 50 InDel markers were selected according to their genotyping success and PCR band size. The discriminating power of the selected 50 InDel set for tartary buckwheat identification was evaluated using the 76 tartary buckwheat accessions. Primers were designed by targeting the InDel region in such a way that the genotypes of tartary buckwheat accessions would produce the same or different (insertion or deletion) amplicons relative to the reference genome, "Daegwan 3-7." Based on the results of InDel marker amplification, the same result with the reference genome was represented by an "a" while other results were represented by a "b." For bar-coding representation of the results, "a" and "b" were converted to white and black, respectively. The homology of the accessions were calculated after PCR amplification of all 50 InDels.

Phylogenetic analysis and population structure among 73 tartary buckwheat accessions
The discriminating power of the selected 50 InDel set for tartary buckwheat identification were evaluated using the 76 tartary buckwheat accessions. The analysis was carried out with POWERMARKER Ver. 3.23 (Liu and Muse 2005; http//www.poxermarker.net). A phylogenetic tree for the 73 accessions was drawn based on the genotypes defined with the 50 InDels using the weighted neighbor-joining method with simple matching coefficients in the DARwin software [55]. To delineate clusters of individuals on the basis of their genotypes, the model-based STRUCTURE 2.3.4 [63] was used with the admixture model, a burn-in period of 100,000 iterations, and data from the 50 InDel loci. The number of clusters (k) was set to five, as this number maximized the Δk (ad hoc criterion) parameter. We used simulations with k values ranging from 2 to 10 with 5 replications, to calculate the LnP(D) value. The optimal k value can be chosen based on the maximum LnP(D) that provides a distinct population structure. On this basis, we chose K = 3 (three ancestral groups) for the tartary buckwheat populations. To check the quality of these analyses, a phylogenetic tree and a population structure for the 26 re-sequenced accessions were drawn based on the genotypes defined using the 6,622 non-synonymous SNPs.

3. **The manuscript needs to be rechecked for grammatical errors and spelling mistakes.**

Thank you for calling this to our attention. Everything you have pointed out has been corrected in the main text. In order to reduce mistakes in grammar/typos, all authors have tried to proofread the manuscript. In addition, this manuscript has been improved by a professional language editing service. We have now provided this information in acknowledgements, as below.

Line 521 to 522:
The authors would like thank Enago (www.enago.co.kr) for the English language review.

4. **Resolution of the all the figures should be checked to comply quality parameters of the journal.**

Thank you for your review. We modified the resolution of all the figures to comply with the quality parameters of the journal.

5. **In Figure 5A author must include a DNA base pair ladder.**

We added a DNA base pair ladder to Figure 5A.

6. **In Figure 8B there is a need to represent the differentiate accessions in their respective subgroups by encircling them.**

We modified Figure 8B according to your suggestion.
