# Supplementary material for: Development of 50 InDel-based barcode system for genetic identification of tartary buckwheat resources
Source: PLoS One. 2021 Jun 3;16(6):e0250786. doi: 10.1371/journal.pone.0250786 (PMC8174720; doi:10.1371/journal.pone.0250786)
Supplement: Attachment — Submitted filename: Response to reviewers.docx [file pone.0250786.s011.docx]

Dear Reviewers,

We greatly appreciate the reviewer’s efforts to carefully review our manuscript and the valuable suggestions offered, which were invaluable in helping us to make improvements. We have revised and modified our manuscript to clarify and strengthen its content, after taking into consideration all of the reviewers’ comments. Please find our responses to each of the reviewers’ comments below.

**Responses to the reviewer’s comments**

**Development of 50 InDel-based barcode system for genetic identification of tartary buckwheat resources**

Submitted: 28 Sep. 2020

Revised: 20 Dec. 2020

**Reviewer # 1**

1. **The language of this manuscript should be improved by a native English speaker, or a professional language editing service. There are a lot of grammar errors in this manuscript. There are a lot of grammar errors in this manuscript, such as Lines 94-97.**

Thank you and we are pleased to inform you that this manuscript has been improved by a professional language editing service. We have now provided this information in acknowledgements, as below.

**Line 521 to 522:**

The authors would like thank Enago ([www.enago.co.kr](http://www.enago.co.kr)) for the English language review.

2. **The 50 InDel-based barcode system has a bias for genetic identification of tartary buckwheat resources. The amount of 50 InDel-based genotypes of 73 accessions is not enough.**

We agree with the reviewer that allelic information derived from high-density markers can reduce a bias for genetic studies of tartary buckwheat accessions. To define the haplotype polymorphisms with high accuracy, the length of the haplotype blocks should be smaller than the length of the LD blocks. Several measurements of LD have been reported for major crop species, e.g., rapeseed (465.5 kb) [1], foxtail millet (*Setaria italic*, 100kb) [2], pigeon pea (*Cajanus cajan*, 70 kb) [3], soybean (150 kb) [4], and rice (200 kb) [5]. In tartary buckwheat, the short breeding history and low selection intensity could result in rapid LD decay compared to other major crop species. For our research, we identified 171,926 homogenous InDel markers from 26 resources and validated them as reliable, transferable and cost-effective markers in 73 accessions.

We questioned how many markers would be appropriate to develop a barcode system for tartary buckwheat identification. Along with the cost of genotyping, user-friendly technology became the key factor to determine the intensity of marker application. Recently, we developed soybean genetic studies using InDels, which were good sources of reference. After an extensive survey of 73,327 InDels by Kim et al. [6], other studies aimed to adopt InDels as tool in soybean genetic studies [7-9]. For this purpose, Sohn et al. [7] showed 202 unique InDel polymorphisms in 147 soybean cultivars, with an average density of one InDel per 5.5 Mb. Recent follow-up studies have reported that genetic diversity studies of Korean soybean cultivars are possible along with the soybean identification using the 27 markers selected from the 202 InDels [8-9]. Although comparisons must be regarded with care due to the different numbers of markers and accessions and the selection intensity, the genetic studies of tartary buckwheat accessions could be performed using the 50 InDels selected from 26 tartary buckwheat whole-genomes.

In the present work, we evaluated the usefulness of the 50-InDel-based barcode system applied to tartary buckwheat classification. Each of the following sections were developed: 1. Putative InDel markers using next generation sequencing; 2. Selection of 50 InDels and bar-coding process; 3. Evaluation of the selected InDels for genetic identification; 4. Constructing a database using expanded InDels in 73 tartary buckwheat accessions.

Phylogenetic analysis of 6,622 non-synonymous SNP variants and population structure subdivided the panel into 2 primary populations (Fig 7 in the manuscript): a reference group and a non-reference group. A similar result was found with the 50 InDels, which clustered the tartary buckwheat into 2 separate groups [9]. Together with the evidence relating the 6,622 SNPs and the 50 InDels to environmental adaptability, these results are consistent with the segregation of the tartary buckwheat into different groups according to their geographical distribution. Particularly because the barcode system could easily identify tartary buckwheat accessions using PCR and gel electrophoresis compared to other molecular markers, such as SSR and SNP. This approach is efficient, rapid and low-cost for the genetic identification of tartary buckwheat accessions in common laboratories.

However, some accessions in subgroup 2 had identical genotypes using 50 InDels (Fig 1). They showed obvious differences in seed shape and seed volume (Fig 1), suggesting that InDel density has not been as well characterized in natural populations as genetic diversity. We tested additional 86 primer sets in which there was no marker to distinguish the resources with identical genotypes using 50 InDels. The results indicate that some accessions could be genetically quite similar.

Fig 1. Seed shapes (A) and barcodes (B) of accessions with the same genotype.

In order to distinguish the identical genotypes, we have been approaching two strategies. First, we have been developing a user-friendly program using a database comprising genotypes based on 50 InDels, seed shapes, and functional substances. The 50 InDels could be used as a complement for classification by seed shapes in resource management (Fig 2). Specifically we are trying to develop additional markers to establish PCR-based markers applicable for all tartary buckwheat accessions. We are currently improving the draft genome of *F. tataricum* cv. “Daegwan 3-7” to reduce the number of annotated genes and duplicated BUSCO. In the future, we will present Fta Daegwan 3-7 gene models and each of the following sections: 1. High-quality draft genome of *F. tataricum*; 2. Comparison to other Carophyllales genomes; 3. Comparison with the draft genome of tartary buckwheat cv. “Pinku1”; 4. Fta pan-genome through SoapDenovo2 assembly of the 26 accessions. These results will lead to the discovery of a larger number of genome-wide markers which will help researchers to genetically characterize the tartary buckwheat genome at high resolution.

Fig 2. The database compiling genotype, seed shape, and functional substances.

**References**

1. Wu D, et al. Whole-genome resequencing of a worldwide collection of rapeseed accessions reveals the genetic basis of ecotype divergence. Molecular Plant. 2019: 12: 30-43.
2. Jia J, et al. A haplotype map of genomic variations and genome-wide association studies of agronomic traits in foxtail millet (*Setaria italic*).Nature genetics. 2013:49:957-961.
3. Varshney RK, et al. Whole-genome resequencing of 292 pigeonpea accessions identifies genomic regions associated with domestication and genomic traits. Nature Genetics. 2017:49:1082.
4. Zhou Z, et al. Resequencing 302 wild and cultivated accessions identifies genes related to domestication and improvement in soybean. Nature biotechnology. 2015:33:408-414.
5. Mather KA, et al. The extent of linkage disquilibrium in rice (*Oryza sativa* L.). Genetics. 2007:177:2223-2232.
6. Kim YH, et al. Variation block-based genomics method for crop plants. BMC Genomics. 2014:15:477.
7. Sohn HB, et al. Barcode system for genetic identification of soybean [*Glycine max* (L.) Merrill] cultivars using InDel markers specific to dense variation blocks. Front Plant Sci*.* 2017;8: 520.
8. Sohn HB, et al. Identification and chromosomal reshuffling patterns of soybean cultivars bred in Gwangwon-do using 202 InDel markers specific to variation blocks. Korean J Breed Sci*.* 2018;50(4): 396-405.
9. Chun JB, et al. Genetic identification and phylogenetic analysis of new varieties and 149 Korean cultivars using 27 InDel markers selected from dense variation blocks in soybean (*Glycine max* (L.) Merrill). Korean J Plant Res. 2019;32(5): 519-542.

**Reviewer # 2**

1. **Abstract has been casually written in terms of language used, especially lines 41-45. Please modify accordingly.**

Your assessment has been of great help to us. We have edited the abstract, as below.

**Line 27 to 48:**

Tartary buckwheat (*Fagopyrum tataricum* Gartn.) is a highly functional crop that is poised to be the target of many future breeding efforts. The reliable *ex situ* conservation of various genetic resources is essential for the modern breeding of tartary buckwheat varieties. We developed PCR-based and co-dominant insertion/deletion (InDel) markers to discriminate tartary buckwheat genetic resources. First, we obtained the whole genome from 26 accessions across a superscaffold-scale reference genome of 569.37 Mb for tartary buckwheat cv. “Daegwan 3-7.” Next, 171,926 homogeneous and 53,755 heterogeneous InDels were detected by comparing 26 accessions with the “Daegwan 3-7” reference sequence. Of these, 100 candidate InDels ranging from 5–20 bp in length were chosen for validation, and 50 of them revealed polymorphisms between the 26 accessions and “Daegwan 3-7.” The validated InDels were further tested through the assessment of their likelihood to give rise to a single or a few PCR products in 50 other accessions, covering most tartary buckwheat genome types. The major allele frequencies ranged from 0.5616 at the TB42 locus to 0.9863 at the TB48 locus, with the average PIC value of 0.1532 with a range of 0.0267–0.3712. To create a user-friendly system, the homology of the genotypes between and among the accessions were visualized in both one- (1D) and two-dimensional (2D) barcode types by comparing amplicon polymorphisms with the reference variety, “Daegwan 3-7.” A phylogenetic tree and population structure of the 76 accessions according to amplicon polymorphisms for the 50 InDel markers corresponded to those using non-synonymous single nucleotide polymorphism variants, indicating that the barcode system based on the 50 InDels was a useful tool to improve the reliability of identification of tartary buckwheat accessions in the germplasm stocks.

2. **What age plant material was used for measuring the rutin and quercetin contents? Mature…how many years?**

We have now provided this information, as below.

**Line 135 to 140:**

To investigate the biodiversity and comparative relationships between the accessions, collected accessions were sown in 2017 and grown in the greenhouse at the Highland Agriculture Research Institute. We separated the plants by developmental stage: the vegetative growth stage (1–40 days after planting, DAP), flowering stage (40–80 DAP), and yield stage (80-90 DAP). At the yield stage, the color of the seed coat turned black, indicating that the seeds were completely maturated.

**Line 142 to 144:**

The yield stage seeds were used to measure their rutin and quercetin content. First, the mature seeds from the 76 accessions were milled into a fine powder, then the samples were immediately flash-frozen in liquid nitrogen and stored at −80°C until further use.

3. **What do you mean by ‘etc.’ in Fig. 2? I am sorry but you can’t use such terms especially describing a figure. Correct it in figure legend as well as in the figure itself. You can add country names as and what available.**

We thank for the reviewer comment. We revised “etc.” in Fig. 2 to USA/Japan/Russia.

4. **In Fig.3, replace the word ‘InDel’ by ‘InDels’ as you dealing with a large no.**

According to the reviewer’s suggestion, InDel in Figure 3 was modified to InDels.

5. **References need thorough rechecking as per journal standards. Maintain uniformity while citing edited books, chapters, chapters and/or proceedings.**

Thank you for calling this to our attention. The Reference section of this manuscript has now been edited to match the journal’s requirements.

6. **Lastly, recently the grammatical mistakes and sentence framing. Authors seemed to be in a hurry while compiling this analysis. It will be better if help of some native English speaker could be taken.**

Thank you for calling this to our attention. Everything you have pointed out has been corrected in the main text. In order to reduce mistakes in grammar/typos, all authors have tried to proofread the manuscript. In addition, this manuscript has been improved by a professional language editing service. We have now provided this information in acknowledgements, as below.

**Line 521 to 522:**

The authors would like thank Enago ([www.enago.co.kr](http://www.enago.co.kr)) for the English language review.

**Reviewer # 3**

1. **The authors write that they have represented a high quality draft genome assembly and annotation for tartary buckwheat. They do not provide the needed analysis results to backup this assertion, as they have not compared their assembly with previously available assemblies.**

To obtain a high-quality draft genome assembly, we produced a total of 43.83 and 32.17 Gb sequences from Illumina paired-ends (PE) and Single-Molecule Real-Time (SMRT) platforms, respectively, which corresponded to 70x (Illumina PE) and 52x (SMART) coverages. A hybrid assembly followed by scaffolding, gap-filling, and cleaning of redundancy, which resulted in a final draft assembly of 569.37 Mb in 2,886 scaffolds with 50% of the total sequence captured in 156 scaffolds larger than 886,968 bps (N50). We predicted a total of 43,771 putative protein-coding gene models occupying 19.33% of the genome, while 52.00% consisted of repetitive sequences and transposable elements, with Gypsy family long terminal repeat (LTR) retrotransposons being the most abundant class.

The problem with *F. tataricum* cv. “Daegwan 3-7” was that the number of annotated genes and duplicated BUSCO was high compared to the draft genome of *F. tataricum* cv. “Pinku 1.” Thus, we are currently working with Professor Dong-Ha Oh of Louisiana State University to improve the draft genome of *F. tataricum* cv. “Daegwan 3-7.” The table below shows the progress to date.

Table. The number of annotated genes and duplicated BUSCO to improve the draft genome of *Fagopyrum tataricum* cv. “Daegwan 3-7.”

In the future, we will present *F. tataricum* “DG” gene model and each of the following sections: 1. High-quality draft genome of *F. tataricum*; 2. Comparison to other Carophyllales genomes; 3. Comparison with the draft genome of tartary buckwheat cv. “Pinku1”; 4. *F. tataricum* pan-genome through SoapDenovo2 assembly of the 26 accessions. Genome sequences, as well as orthologous genes and gene families identified as uniquely expanded in tartary buckwheat through comparative analysis, could provide an essential resource for studying the adaptation, specialized metabolism, and the genomic basis of the strikingly high rutin content in this interesting crop species.

2. **The material and methods section of the manuscript is not precise enough and does not contain all the processing steps presented in the result section. Such as the conditions for structure analysis should be detailed, with parameter settings.**

The material and methods section has now been modified per the reviewer’s suggestion. The blue marked portion has been added.

**Line 124 to 241:**

**Plant Materials**

The accessions of 73 tartary buckwheat plants (S1 Table) were provided by the National Agrobiodiversity Center (Jeonju, Republic of Korea) and the Highland Agriculture Research Institute (Pyeongchang, Republic of Korea). The accessions were planted in a greenhouse located at the Highland Agriculture Research Institute (37°68′N and 128°73′E, 779 m altitude), Gangwon Province, Republic of Korea. These were self-pollinated 3 times by the single seed descent (SSD) method by growing the individual plants in flowerpots (10cm in diameter) [50]. We grew an additional four generations of HLB1001 using the SSD method, which resulted in the selection of a progeny, “Daegwan 3-7” for a draft genome of tartary buckwheat. A total of 26 accessions were collected (China, India, Nepal, Bhutan, Pakistan, USA).

To investigate the biodiversity and comparative relationships between the accessions, collected accessions were sown in 2017 and grown in the greenhouse at the Highland Agriculture Research Institute. We separated the plants by developmental stage: the vegetative growth stage (1–40 days after planting, DAP), flowering stage (40–80 DAP), and yield stage (80-90 DAP). At the yield stage, the color of the seed coat turned black, indicating that the seeds were completely maturated.

**Measurement of rutin and quercetin content**

The yield stage seeds were used to measure their rutin and quercetin content. First, the mature seeds from the 76 accessions were milled into a fine powder, then the samples were immediately flash-frozen in liquid nitrogen and stored at −80°C until further use. A total of 0.10 g dry powder for each sample was mixed with 1 ml methanol, then was extracted at 80°C for 1 hr in a conventional Soxhlet apparatus. The extract was filtered through a 0.20 μm syringe filter (PTFE 13 mm, PALL Life Sciences, Ann Arbor, MI) for rutin content [23]. Ultra-performance liquid chromatography (Waters Corporation, Milford, MA, USA) was performed on an Acquity 1-class using a C18 column (2.1 mm × 100 mm, 1.7 μm) (Waters Corporation, USA) at 30°C. The mobile phase (1% formic acid in water/0.1% formic acid in acetonitrile) flowed by the gradient elution method (S2 Table) at a flow rate of 0.25 ml/min with a total injection volume of 10 ㎕. Rutin quantity was estimated based on the linear calibration curve of standard rutin and quercetin (Extrasynthese, France) under a detection wavelength of 259 nm. Three independent sample analyses were performed for each sample. All statistical analyses were performed using ver. 3.6.1 of the R software.

**DNA sequencing and *de novo* assembly**

After 21 DAP, the leaves in the third node of the progeny “Daegwan 3-7” were used to create a draft genome. First, 3-week-old leaves from the bulk of 10 plants were collected for genomic DNA extraction using a standard CTAB (cetyl trimethylammonium bromide) protocol [52, 53]. Both short read (Illumina) and long read (PacBio) libraries were prepared according to the manufacturer’s instructions for the entire genome of *F. tataricum*. A short insert paired-end (PE) library was prepared using a Illumina TruSeq Nano DNA Sample Preparation Kit (Illumina Inc., San Diego, Ca, USA) and 1~3 ㎍ of DNA. A library profile analysis was performed with an Agilent 2100 Bioanalyzer (Agilent Technologies, USA) and qPCR quantification, then the libraries were sequenced to 2 × 100 bp on the Illumina HiSeq 2500 platform (Illumina, USA) from the TheragenEtex Bio Institute (TheragenEtex Inc., Suwon, Republic of Korea). PacBio SMRTbell libraries (2 kb, 5 kb, 10 kb, and 15 kb inserts) were prepared with the standard PacBio library preparation protocols, which are available at http://pacificbiosciences.com/. The sequencing was conducted on a PacBio RS II (Pacific Biosciences, USA) system using C4 chemistry and 240 min movies by the TheragenEtex Bio Institute.

The short and long reads were assembled separately. The de novo assembly of the PacBio long reads was performed using Fast Alignment and CONsensus [54] with default parameters. The short reads were assembled with SOAPdenovo2 [55] with default parameters. The initial contigs were merged using HaploMerger2 [56]. Both short and long reads were then used to construct scaffolds with SSPACE [57], which led to the superscafold-scale draft genome of tartary buckwheat cv. “Daegwan 3-7.” The N50 length of the final genome assemblies was evaluated.

**Identification and validation of the polymorphic InDels**

Genomic DNA was extracted for whole-genome resequencing from the 26 accessions that had self-pollinated 3 times using the SSD method. Three-week-old leaves from the bulk of 10 plants were collected for genomic DNA extraction using a standard CTAB protocol [52, 53]. The data consisted of 101-bp reads, which were generated using the HiSeq 2500 sequencer. The Short Oligonucleotide Alignment Program 2 (SOAP2) was used to map the raw pair-end reads onto the reference genome (tartary buckwheat cv. “Daegwan 3-7”). For each accession, more than 93% of the reads were properly aligned to the reference genome. Insert size was estimated by mapping the reads to the reference genome using the Burrows-Wheeler Aligner algorithm (bwa) [58] ver 0.5.9. The aligned reads were realigned at InDel positions using the GATK InDelRealinger algorithm [59] enhance the mapping quality. The base quality scores were recalibrated using the GATK TableRecalibration algorithm. The primer pair used to amplify each of the InDels were selected by the Primer3 software (<http://primer3.sourceforge.net>). We selected InDels with more than 10 bp and designed the primers accordingly to match the characteristics of each InDel using the Primer 3 software.

**Amplicon polymorphism assay and bar-coding process**

PCR analysis was performed using 10 ㎕ reaction mixtures containing 20 ng of total genomic DNA, 2 pM of primer, and 5 ㎕ of GoTaq Green Master Mix (Promega, Madison, WI, USA). The PCR was performed at 95℃ for 5 min, followed by 35 subsequent rounds at 94℃ for 30 sec, 45°C for 30 sec and 72°C for 30 sec, using a Mastercycler pro 384 (Eppendorf, Germany). The PCR products were separated by electrophoresis in 3% gels of certified low range ultra-agarose (Bio-rad) followed by LoadingSTAR staining (Dynebio, Republic of Korea). For the limitation of the number of candidates, we chose primer pairs that amplified PCR products that were 150–231 bp long. The allelic diversity of the InDels with the e-PCR products in 26 tartary buckwheat genomes were assessed via PIC (Polymorphism Information Content), which was defined as PIC *i* =1-$\sum_{j=1}^{n} p_{ij}^{2}$, where p _ij_ is the frequency of the jth pattern for the ith marker [60,61].

The 50 InDel markers were selected according to their genotyping success and PCR band size. The discriminating power of the selected 50 InDel set for tartary buckwheat identification was evaluated using the 76 tartary buckwheat accessions. Primers were designed by targeting the InDel region in such a way that the genotypes of tartary buckwheat accessions would produce the same or different (insertion or deletion) amplicons relative to the reference genome, “Daegwan 3-7.” Based on the results of InDel marker amplification, the same result with the reference genome was represented by an “a” while other results were represented by a “b.” For bar-coding representation of the results, “a” and “b” were converted to white and black, respectively. The homology of the accessions were calculated after PCR amplification of all 50 InDels.

**Phylogenetic analysis and population structure among 73 tartary buckwheat accessions**

The discriminating power of the selected 50 InDel set for tartary buckwheat identification were evaluated using the 76 tartary buckwheat accessions. The analysis was carried out with POWERMARKER Ver. 3.23 (Liu and Muse 2005; http//www.poxermarker.net). A phylogenetic tree for the 73 accessions was drawn based on the genotypes defined with the 50 InDels using the weighted neighbor-joining method with simple matching coefficients in the DARwin software [55]. To delineate clusters of individuals on the basis of their genotypes, the model-based STRUCTURE 2.3.4 [63] was used with the admixture model, a burn-in period of 100,000 iterations, and data from the 50 InDel loci. The number of clusters (k) was set to five, as this number maximized the △k (ad hoc criterion) parameter. We used simulations with k values ranging from 2 to 10 with 5 replications, to calculate the LnP(D) value. The optimal k value can be chosen based on the maximum LnP(D) that provides a distinct population structure. On this basis, we chose K = 3 (three ancestral groups) for the tartary buckwheat populations. To check the quality of these analyses, a phylogenetic tree and a population structure for the 26 re-sequenced accessions were drawn based on the genotypes defined using the 6,622 non-synonymous SNPs*.*

3. **The manuscript needs to be rechecked for grammatical errors and spelling mistakes.**

Thank you for calling this to our attention. Everything you have pointed out has been corrected in the main text. In order to reduce mistakes in grammar/typos, all authors have tried to proofread the manuscript. In addition, this manuscript has been improved by a professional language editing service. We have now provided this information in acknowledgements, as below.

**Line 521 to 522:**

The authors would like thank Enago ([www.enago.co.kr](http://www.enago.co.kr)) for the English language review.

4. **Resolution of the all the figures should be checked to comply quality parameters of the journal.**

Thank you for your review. We modified the resolution of all the figures to comply with the quality parameters of the journal.

5. **In Figure 5A author must include a DNA base pair ladder.**

We added a DNA base pair ladder to Figure 5A.

6. **In Figure 8B there is a need to represent the differentiate accessions in their respective subgroups by encircling them.**

We modified Figure 8B according to your suggestion.
