# Supplementary material for: Development of 50 InDel-based barcode system for genetic identification of tartary buckwheat resources
Source: PLoS One. 2021 Jun 3;16(6):e0250786. doi: 10.1371/journal.pone.0250786 (PMC8174720; doi:10.1371/journal.pone.0250786)
Supplement: Attachment — Submitted filename: Comments_PLoS Revision.docx [file pone.0250786.s012.docx]

Dear Editor,

All queries have been answered satisfactorily in the revised version of this manuscript. The language has also been improved.

There is just a very minor mistake in the abstract portion which I suppose should be corrected. Rest all seems fine now.

Regards
