# Supplementary material for: Development of 50 InDel-based barcode system for genetic identification of tartary buckwheat resources
Source: PLoS One. 2021 Jun 3;16(6):e0250786. doi: 10.1371/journal.pone.0250786 (PMC8174720; doi:10.1371/journal.pone.0250786)
Supplement: Attachment — Submitted filename: Response to editor.docx [file pone.0250786.s014.docx]

Dear Editor,

We greatly appreciate the reviewer’s efforts to carefully review our manuscript and the valuable suggestions offered, which were invaluable in helping us to make improvements. We have revised and modified our manuscript to clarify and strengthen its content, after taking into consideration all of the Editor’ comments. Please find our responses to each of the Editor’ comments below.

**Responses to the editor’s comments**

**Development of 50 InDel-based barcode system for genetic identification of tartary buckwheat resources (PONE-D-20-30493R1)**

Submitted: 28 Sep. 2020

Revised: 21 March 2020

**Please review your reference list to ensure that it is complete and correct. If you have cited papers that have been retracted. Please include the rationale for doing so in the manuscript text, or remove these references and replace them with relevant current references. Any changes to the reference list should be mentioned in the rebuttal letter that accompanies your revised manuscript. If you need to cite a retracted article, indicate the article’s retracted status in the References list and also include a citation and full reference for the retraction notice.**

The changed references are as follows. In addition, the changes have been indicated in the file (Revised Manuscript with Track Changes.docx).

**Line 30 to 31:**

We developed PCR-based ~~and~~ co-dominant insertion/deletion (InDel) markers to discriminate tartary buckwheat genetic resources.

**Line 101 to 103:**

In addition, Sohn et al. [38] reported that the InDel-based barcode system focuses on usability, and provided an efficient resource management system.

**Line 130 to 133:**

We grew an additional four generations of HLB1001 using the SSD method, which resulted in the selection of a progeny, “Daegwan 3-7” for a draft genome of tartary buckwheat. A total of 26 accessions were collected (China, India, Nepal, Bhutan, Pakistan, and USA).

**Line 528 to 530:**

Ohnishi O, Matsuoka Y. Search for the wild ancestor of buckwheat II. Taxonomy of *Fagopyrum* (Polygonaceae) species based on morphology, isozymes and cpDNA variability. Genes Genet Syst. 1996;71:383-390. doi: 10.1266/ggs.71.383

**Line 540 to 542:**

Huang W, Jarvis D, Ahmed S, Long C. Tartary buckwheat genetic diversity in the Himalayas associated with farmer landrace diversity and low dietary dependence. Sustainability. 2017;9(10):1806. doi: 10.3390/su9101806

**Line 543 to 545:**

*Fagopyrum esculentum* Moench. X. Diffusion routes revealed by RAPD markers. Genes Genet Syst. 1996;71:211-218. doi: 10.1266/ggs.71.211

**Line 546 to 549:**

Ohnishi O. Search for the wild ancestor of buckwheat III. The wild ancestor of cultivated common buckwheat, and of tatary buckwheat. Econ Bot. 1998;52(2):123-133. Available at [http://www.jstor.com/stable/4256049.pdf. Accessed 18 September 2020](http://www.jstor.com/stable/4256049.pdf.%20Accessed%2018%20September%202020)

**Line 553 to 556:**

Hou S, Sun Z, Linghu B, Xu D, Wu B, Zhang B, et al. Genetic diversity of buckwheat cultivars (*Fagopyrum tartaricum* Gaertn.) assessed with SSR markers developed from genome survey sequences. Plant Mol Biol Report. 2016;34(1):233–241. doi: 10.1007/s11105-015-0907-5

**Line 565 to 568:**

Liu M, Ma Z, Zheng T, Sun W, Zhang Y, Jin W, et al. Insights into the correlation between physiological changes in and seed development of tartary buckwheat (*Fagopyrum tataricum* Gaertn.). BMC Genomics. 2018;19(1):648. doi: 10.1186/s12864-018-5036-8

**Line 569 to 571:**

Spengler R. Anthropogenic seed dispersal: Rethinking the origins of plant domestication. Trends Plant Sci. 2020;25(4):340-348. doi: 10.1016/j.tplants.2020.01.005

**Line 582 to 584:**

Fabjan N, Rode J, [Košir](https://pubs.acs.org/action/doSearch?field1=Contrib&text1=Iztok+Jo%C5%BEe++Ko%C5%A1ir) IJ, Wang Z, Zhang Z, Kreft I. Tartary buckwheat (*Fagopyrum tataricum* Gaertn.) as a source of dietary rutin and quercitrin. J Agric Food Chem. 2003;51(22):6452-6455. doi: 10.1021/jf034543e

**Line 604 to 606:**

Wang Y, Campbell CG. Tartary buckwheat breeding (*Fagopyrum tataricum* L. Gaertn.) through hybridization with its rice-tartary type. Euphytica. 2007;156(3):399-405. doi: 10.1007/s10681-007-9389-3

**Line 616 to 618:**

Hyten DL, Song Q, Zhu Y, Choi IY, Nelson RL, Costa JM, et al. Impacts of genetic bottlenecks on soybean genome diversity. Proc Natl Acad Sci U S A. 2006;103(45):16666–16671. doi: 10.1073/pnas.0604379103

**Line 619 to 621:**

Bhandari HR, Bhanu AN, Srivastava K, Singh MN, Shreya, Hemantaranjan A. Assessment of genetic diversity in crop plants – an overview. Adv Plants Agric Res. 2017;7(3):279-286. doi: 10.15406/apar.2017.07.00255

**Line 624 to 626:**

Hughes AR, Inouye BD, Johnson MTJ, Underwood N, Vellend M. Ecological consequences of genetic diversity. Ecol Lett. 2008;11(6):609-623. doi: 10.1111/j.1461-0248.2008.01179.x

**Line 630 to 632:**

Väli Ü, Brandström M, Johansson M, Ellegren H. Insertion-deletion polymorphisms (indels) as genetic markers in natural populations. BMC Genet. 2008;9(1):8. doi: 10.1186/1471-2156-9-8

**Line 639 to 642:**

Meng HT, Zhang YD, Shen CM, Yuan GL, Yang CH, Jin R, et al. Genetic polymorphism analyses of 30 InDels in Chinese Xibe ethnic group and its population genetic differentiations with other groups. Scientific Reports. 2015;5(5):8260. doi: 10.1038/srep08260

**Line 639 to 642:**

Tsuji K, Ohnishi O. Phylogenetic position of east Tibetan natural populations in tartary buckwheat (*Fagopyrum tataricum* Gaertn.) revealed by RAPD analyses. Genet Resour Crop Evol*.* 2001;48(1):63–67. doi: 10.1023/A:1011286326401

**Line 639 to 642:**

Zhang A, Zhao L. Molecular characterization of genetic diversity of underutilized crops; Buckwheat as an example. Acta Hortic. 2013;979:407-419. doi: 10.17660/ActaHortic.2013.979.44

**Line 718 to 720:**

Liu K, Muse SV. PowerMarker: an integrated analysis environment for genetic marker analysis. Bioinformatics. 2005;21(9):2128-2129. doi: 10.1093/bioinformatics/bti282

**Line 721 to 722:**

Perrier X, Jacquemoud-Collet JP. DARwin software. 2006. Available at http://darwin.cirad.fr/

**Line 728 to 731:**

Jiang P, Burczynski F, Campbell C, Pierce G, Austria JA, Briggs CJ. Rutin and flavonoid contents in three buckwheat species *Fagopyrum esculentum*, *F. tataricum*, and *F. homotropicum* and their protective effects against peroxidation. Food Res Int. 2007;40(3):356-364. doi: 10.1016/j.foodres.2006.10.009

**Line 741 to 743:**

Germ M, Brenznik B, Dolinar N, Kreft I, Gaberščik A. The combined effect of water limitation and UV-B radiation on common and tartary buckwheat. Cereal Res Commun. 2013;41(1):97-105. doi: 10.1556/CRC.2012.031

**Line 744 to 747:**

Pandey V, Niranjan A, Atri N, Chandrashekhar K, Mishra MK, Trivedi PK, Misra P. *WsSGTL1* gene from *Withania somnifera*, modulates glycosylation profile, antioxidant system and confers biotic and salt stress tolerance in transgenic tobacco. Planta. 2014;239(6):1217-1231. doi: 10.1007/s00425-014-2046-x

**Line 762 to 764:**

Tian D, Wang Q, Zhang P, Araki H, Yang S, Kreitman M, et al. Single-nucleotide mutation rate increases close to insertion/deletions in eukaryotes. Nature. 2008;455:105-108. doi: 10.1038/nature07175

**Line 765 to 768:**

van der Biezen EA, Freddie CT, Kahn K, Parker JE, Jones JDG. *Arabidopsis RPP4* is a member of the *RPP5* multigene family of TIR-NB-LRR genes and confers downy mildew resistance through multiple signaling components. Plant J. 2002;29(4):439-451. doi; 10.1046/j.0960-7412.2001.01229.x

**Line 769 to 772:**

Langlois-Meurinne M, Gachon CM, Saindrenan P. Pathogen-responsive expression of glycosyltransferase genes *UGT73B3* and *UGT73B5* is necessary for resistance to *Psudomonas syringae* pv *tomato* in Arabidopsis. Plant Physiol. 2005;139(4):1890-1901. doi: 10.1104/pp.105.067223

**Line 773 to 776:**

Attaran E, Rostás M, Zeier J. *Pseudomonas syringae* elicits emission of the terpenoid (E,E)-4,8,12-trimethnyl-1,3,7,11-tridecatetraene in *Arabidopsis* leaves via jasmonate signaling and expression of the terpene synthase TPS4. Mol Plant Microbe Interact. 2008;21(11):1482-1497. doi: 10.1094/MPMI-21-11-1482

**Line 777 to 779:**

Islam MS, Wang MH. Expression of dehydration responsive element-binding protein-3 (*DREB3*) under different abiotic stresses in tomato. BMB Rep*.* 2009;42(9):611-616. doi: 10.5483/bmbrep.2009.42.9.611

**Line 780 to 782:**

Bellucci E, Bitocchi E, Rau D, Nanni L, Ferradini N, Giardini A, et al. Population structure of barley landrace populations and gene-flow with modern varieties. PLoS ONE. 2013;8(12):e83891. doi: 10.1371/journal.pone.0083891

**Line 783 to 785:**

Hollister JD, Ross-Ibara J, Gaut BS. Indel-associated mutation rate varies with mating system in flowering plants. Mol Biol Evol. 2010;27(2):409-416. doi: 10.1093/molbev/msp249

**Line 786 to 787:**

Leishman MR, Westoby M, Jurado E. Correlates of seed size variation: a comparison among five temperate floras. J Ecol. 1995;83(3):517-529. doi: 10.2307/2261604

**My question is that authors have not deposit the sequencing data in Public repositories like NCBI or any other. Firstly authors submit data into NCBI and put SRA number or any ID. Because I don’t get the details for the same in the manuscript and I am not satisfied with this, I only accept the manuscript if authors provide these details.**

I agree with your opinion. But, the draft genome has limitations. First, the draft genome of tartary buckwheat cv. “Daegwan 3-7” showed 43,771 gene models compared to 30,386 in “PinKu1.” Second, “Daegwan3-7” matched 80% of showed 80% gene matches compared to 89% in “Pinku1” out of 1,375 BUSCOs. Thus, we’re currently working with Professor Dong-Ha Oh of Louisiana State University to improve gene models and BUSCOs of the draft genome (Table 1). In addition, we will try to present *de novo* assembly of the 26 genomes by using the improved gene models and BUSCOs of the draft genome. It would be possible to perform pan-genome analysis of the 26 genomes, enabling identification of the core and expanded subgenomes of tartary buckwheat.

Table 1. Improving gene models of *Fagopyrum tataricum* “Daegwan3-7”

In the future, we will deposit the improved sequencing data in public repositories. These results will lead to the discovery of a larger number of genome-wide markers which will help researchers to genetically characterize the tartary buckwheat genome at high resolution. Furthermore, genome sequences, as well as orthologous genes and gene families identified as uniquely in tartary buckwheat through comparative analysis, provide an essential resource for studying the adaptation, specialized metabolism, and the genomic basis of the strikingly high rutin content in this interesting crop species.

It is clear that the 50-InDel based barcode system should be efficient, fast and low-cost approach for the genetic identification of tartary buckwheat accessions. We believe that these modifications have strengthened the manuscript and hope that the revised manuscript is suitable for publication in PLOS ONE.
